# Supplementary material for: STARD3 regulates lysosome positioning and contacts via a GSK3-controlled phosphorylation switch
Source: EMBO J. 2026 Feb 25;45(7):2239–77. doi: 10.1038/s44318-026-00705-3 (PMC13044316; doi:10.1038/s44318-026-00705-3)
Supplement: Supplementary file 1 — Appendix [file 44318_2026_705_MOESM1_ESM.pdf]

Appendix for "STARD3 regulates lysosome positioning and contacts **via a GSK3-controlled phosphorylation switch"**

Table of contents

Appendix Figure S1: MS/MS analysis of S_{213} phosphorylation on recombinant pS_{213} cSTD3 protein 2

Appendix Figure S2: In HeLa cells, GSK3 activity regulates the interaction between STARD3 and VAPs and the establishment of ER-endosome contacts 3

Appendix Figure S3: *In vivo*, GSK3 activity regulates the establishment of ER-endosome contacts mediated by STARD3 and VAP-B 4

Appendix Figure S4: Schematic representation of the method used to quantify the clustering index of LE/Lys 5

Appendix Figure S5: Transmission Electron Microscopy (TEM) analysis of STARD3-induced LE/Lys clusters 6

Appendix Figure S6: The START domain of STARD3 mediates LE/Lys clustering 7

Appendix Figure S7: Proteins with a potential Phospho-FFAT possibly activated by GSK3 8

A S_{213} phosphorylation identification

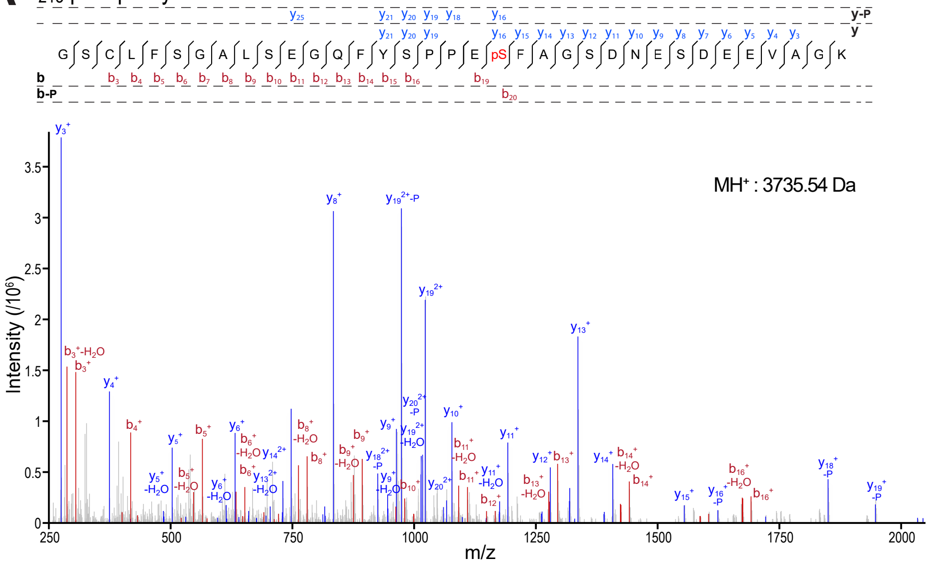

Appendix Figure S1: MS/MS analysis of S_{213} phosphorylation on recombinant pS_{213} cSTD3 protein

MS/MS spectrum of a peptide phosphorylated on S_{213}. The molecular weight of the peptide is indicated on the right. The spectrum is an assembly of ions produced by collision-induced dissociation of the precursor peptides. Fragmentation occurs preferentially at peptide bonds to generate b and y ions, which extend from the amino and carboxy terminus, respectively. The precursor peptide sequence and the different b and y ions identified in the spectrum are shown above. b and y ion peaks are shown in red and blue, respectively. b and y ions with the highest intensity are labeled on the spectrum. Neutral mass losses of H_2O and H_3PO_4 (P) are indicated. Analysis of y and b ion fragmentation patterns showed that S_{213} is phosphorylated.

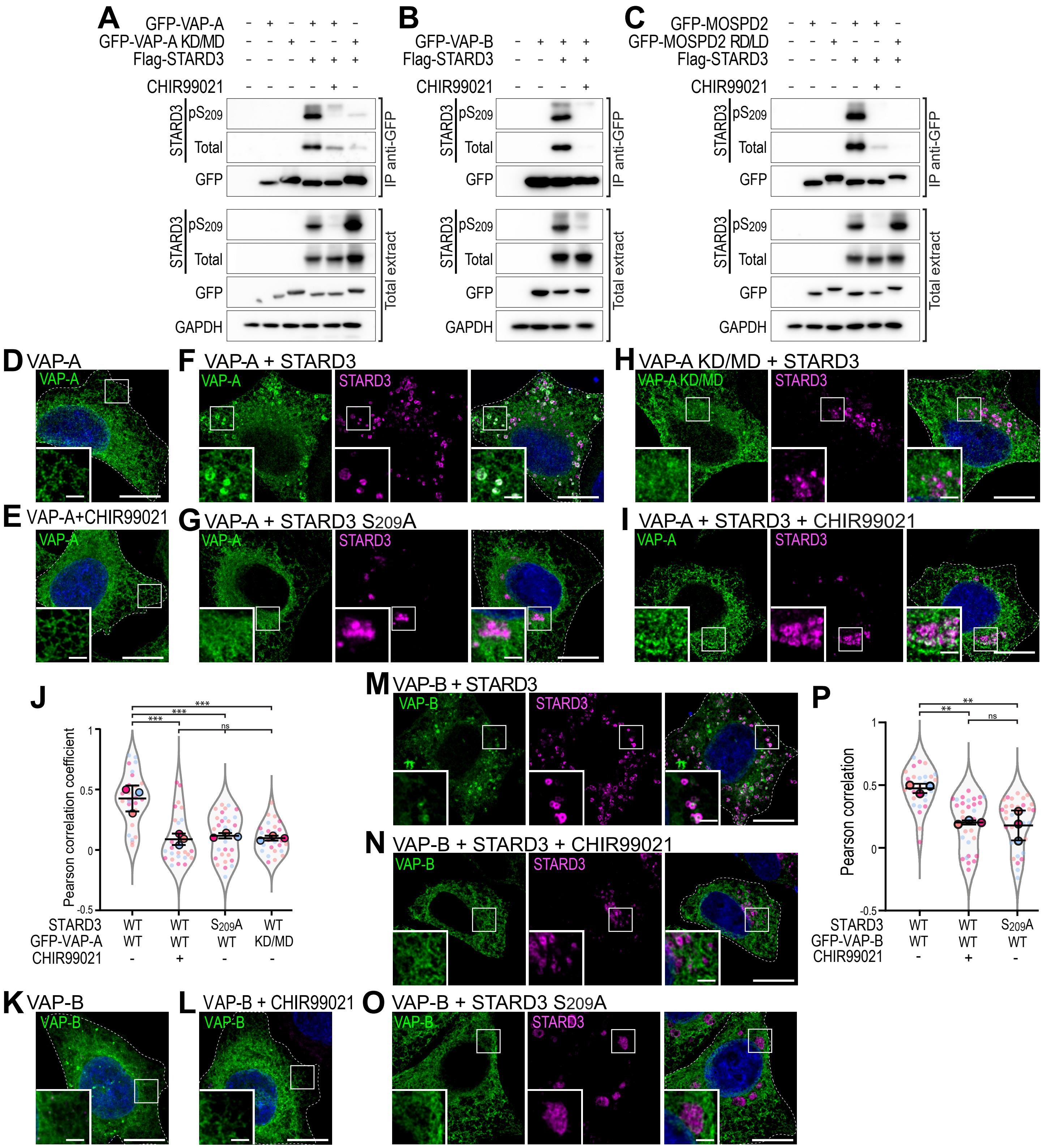

Appendix Figure S2: In HeLa cells, GSK3 activity regulates the interaction between STARD3 and VAPs and the establishment of ER-endosome contacts

A-C: Immunoprecipitation (GFP-Trap) experiments between GFP-VAP-A (A), GFP-VAP-B (B) or GFP-MOSPD2 (C) and Flag-tagged STARD3 in HeLa cells. Approximatively 5 µg of total protein extract was analyzed by Western blot using anti-STARD3, anti-pS$_{209}$-STARD3, anti-GFP and anti-GAPDH antibodies. Immunoprecipitated proteins were analyzed using anti-STARD3, anti-pS$_{209}$-STARD3 and anti-GFP antibodies.

D-I: HeLa cells expressing GFP-VAP-A (D-G, J) and GFP-VAP-A KD/MD (H) (green) were either untransfected (D-E) or transfected with STARD3 WT (F, H, I) or STARD3 S$_{209}$A (G). Cells were left untreated (D, F-H) or treated with CHIR99021 (E, I). STARD3 was labeled using anti-STARD3 antibodies (F-I, magenta), and nuclei stained with Hoechst (blue). Insets show higher magnification images of the areas outlined in white. Scale bars: 10 µm. Inset scale bars: 2 µm. The overlay panels show merged green, magenta and blue images. In D and E, endogenous STARD3 levels were too low to be detected with anti-STARD3 antibodies.

J: Pearson's correlation coefficients between VAP-A (WT or KD/MD mutant) and STARD3 (WT or STARD3 S$_{209}$A) in cells treated or not with CHIR99021. Data are displayed as Superplots with Pearson's correlation coefficient for individual cells (small dots) and the mean per independent experiment (large dots). Number of cells: VAP-A–STARD3: 28; VAP-A–STARD3 treated with CHIR99021: 28; VAP-A–STARD3 S$_{209}$A: 30, VAP-A KD/MD–STARD3: 27, from three independent experiments. Independent experiments are color-coded. Means ± SD. ANOVA with Tukey's multiple comparison test (***, $P < 0.001$).

K-O: HeLa cells expressing GFP-VAP-B (K-O; green) were either untransfected (K, L) or transfected with STARD3 WT (M, N) or STARD3 S$_{209}$A (O). Cells were left untreated (K, M, O) or treated with CHIR99021 (L, N). STARD3 was labeled using anti-STARD3 antibodies (magenta), and nuclei were stained with Hoechst (blue). Insets show higher magnification images of the areas outlined in white. Scale bars: 10 µm. Inset scale bars: 2 µm. The overlay panels show merged green, magenta and blue images. In K and L, endogenous STARD3 levels were too low to be detected with anti-STARD3 antibodies.

P: Pearson's correlation coefficients between VAP-B and STARD3 (WT or S$_{209}$A) in cells treated or not with CHIR99021. Each dot represents a single cell (number of cells: VAP-B-STARD3: 26; VAP-B-STARD3 treated with CHIR99021: 30; VAP-B-STARD3 S$_{209}$A: 33, from three independent experiments). Means and error bars (SD) are shown. ANOVA with Tukey's multiple comparison test (**, $P < 0.01$).

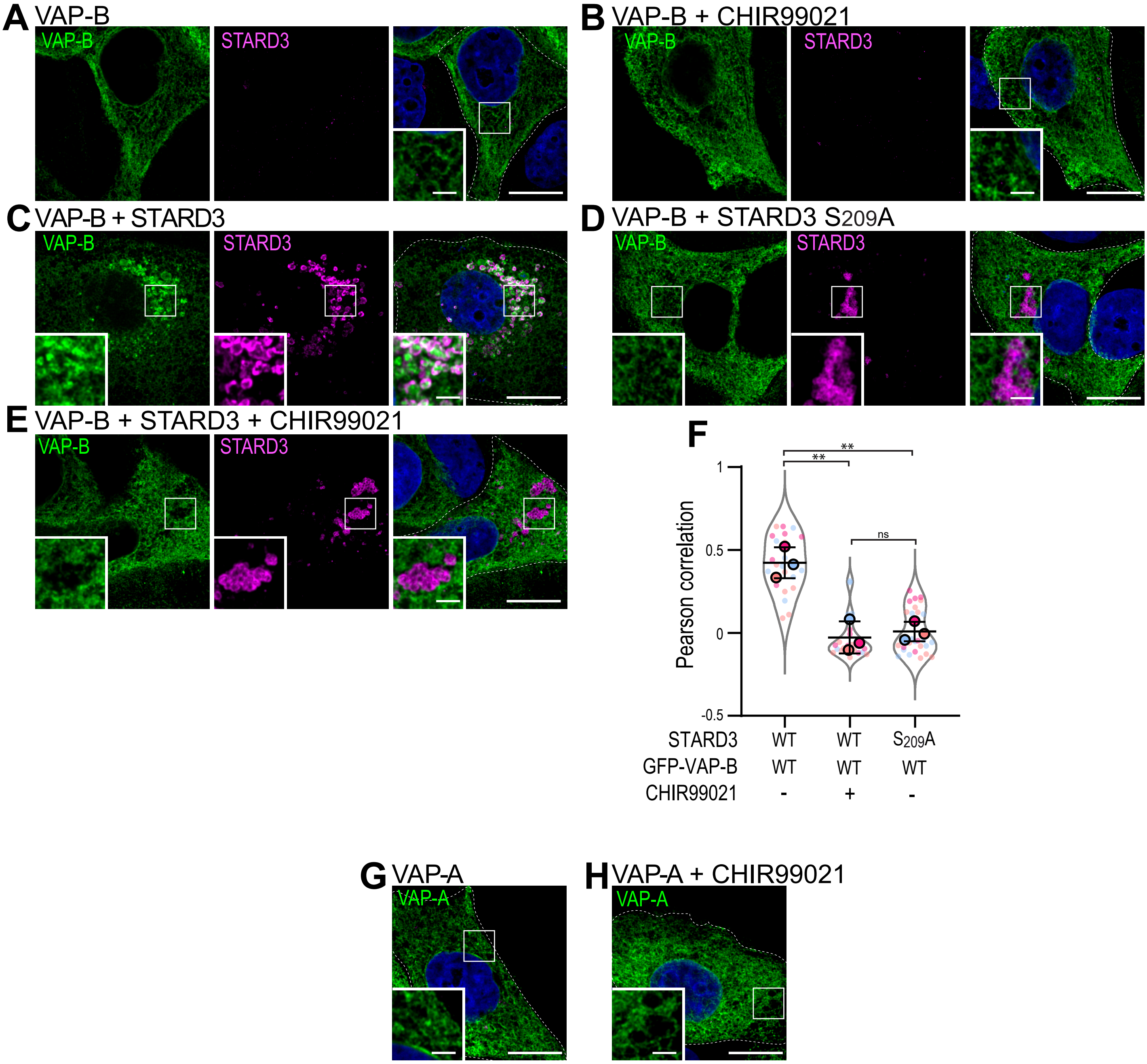

Appendix Figure S3: In vivo, GSK3 activity regulates the establishment of ER-endosome contacts mediated by STARD3 and VAP-B

A-E, G-H: MCF7 cells expressing GFP-VAP-B (A-E; green) or GFP-VAP-A (G, H) were either untransfected (A-G) or transfected with STARD3 WT (C, E) or STARD3 S_{209}A (D). Cells were left untreated (A, C, D, G) or treated with CHIR99021 (B, E, H). STARD3 was labeled using anti-STARD3 antibodies (magenta), and nuclei were stained with Hoechst (blue). Insets show higher magnification images of the areas outlined in white. Scale bars: 10 µm. Inset scale bars: 2 µm. The overlay panels show merged green, magenta and blue images. In A-D, endogenous STARD3 levels were too low to be detected with anti-STARD3 antibodies.

F: Pearson's correlation coefficients between VAP-B and STARD3 (WT or S_{209}A) in cells treated or not with CHIR99021. Each dot represents a single cell (number of cells: VAP-B-STARD3: 22; VAP-B-STARD3 treated with CHIR99021: 23; VAP-B-STARD3 S_{209}A: 28, from three independent experiments). Means and error bars (SD) are shown. ANOVA with Tukey's multiple comparison test (**, $P < 0.01$).

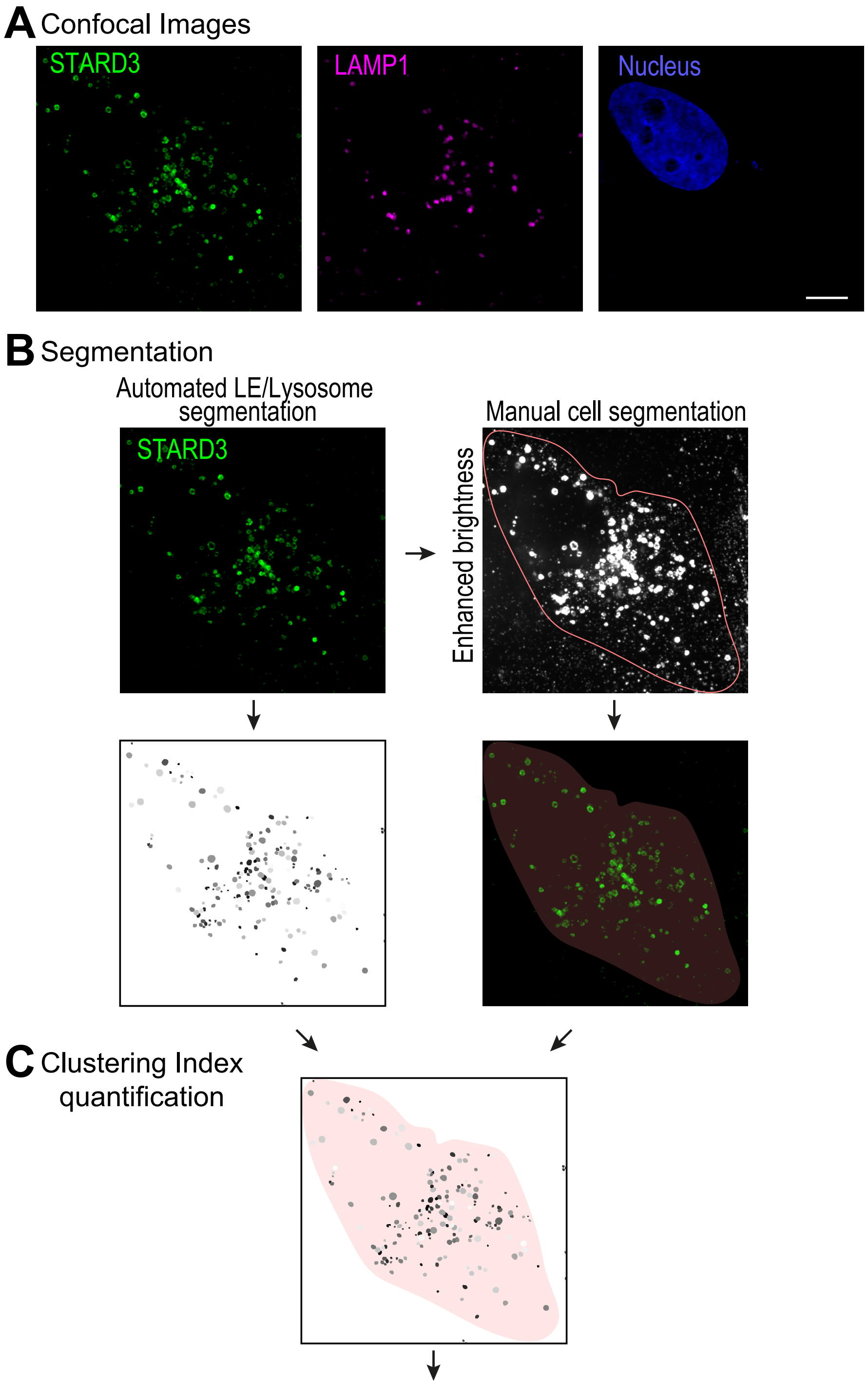

$$\text{Clustering Index} = \frac{\text{Number of ROIs in contact}}{\text{Total number of ROIs}}$$

Appendix Figure S4: Schematic representation of the method used to quantify the clustering index of LE/Lys

A: Cells were labelled with anti-STARD3 (green), anti-LAMP1 antibodies (magenta) with nuclei stained with Hoechst (blue). Images were acquired with a confocal Spinning Disk microscope with a Live-SR Super Resolution module and analyzed with CellProfiler. Scale bar: 10 µm.

B: Image segmentation: LE/Lys were segmented based on STARD3 staining (or LAMP1 staining in Figure 4 A-C) using StarDist, trained on a custom dataset. Cell contours were manually segmented.

C: Clustering Index quantification: using the segmentation data of cells and LE/Lys, the proportion of LE/Lys in contact with at least one other LE/Lys per cell was determined (Cellprofiler, Module: MeasureObjectNeighbors, module method: Adjacent). The clustering index ranges from 0 (all LE/Lys are isolated) to 1 (all LE/Lys are in contact with at least one other).

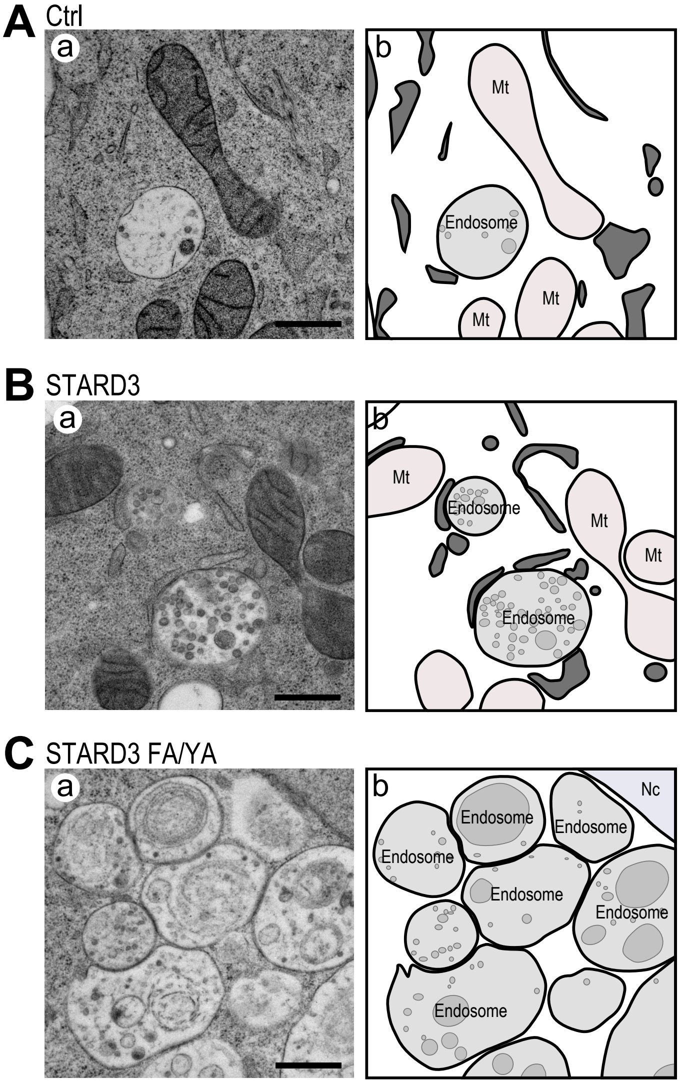

Appendix Figure S5: Transmission Electron Microscopy (TEM) analysis of STARD3-induced LE/Lys clusters
TEM images (a) of control HeLa cells (A), HeLa cells expressing STARD3 (B), and HeLa cells expressing STARD3 FA/YA (C). Scale bars: 500 nm. Schematic representation (b) of images shown in (a) indicate the ER, endosomes, and intraluminal membranes in dark, light, and medium gray, respectively. Mt: mitochondria; Nc: nucleus.

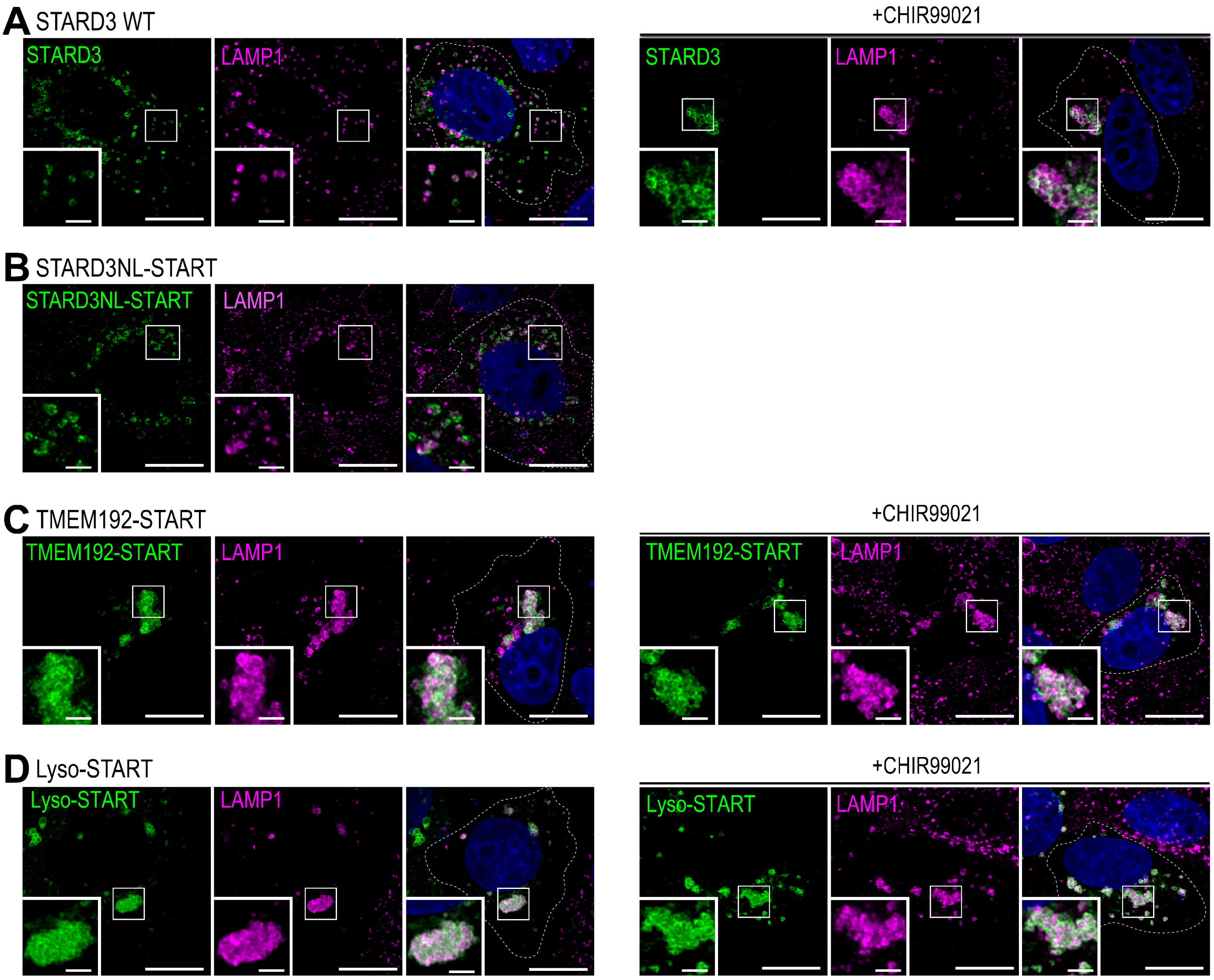

Appendix Figure S6: The START domain of STARD3 mediates LE/Lys clustering
MCF7 cells expressing STARD3 WT left untreated (left) or treated with CHIR99021 (5 µM, overnight; right) (A) and MCF7 cells expressing STARD3NL-START (B), TMEM192-START (C), and Lyso-START (D) were labelled with anti-STARD3 antibodies (green) and with anti-LAMP1 antibodies to label LE/Lys (magenta). Nuclei were stained with Hoechst (blue). Subpanels show higher magnification images of the area outlined in white. Scale bars: 10 µm. Inset scale bars: 2 µm.

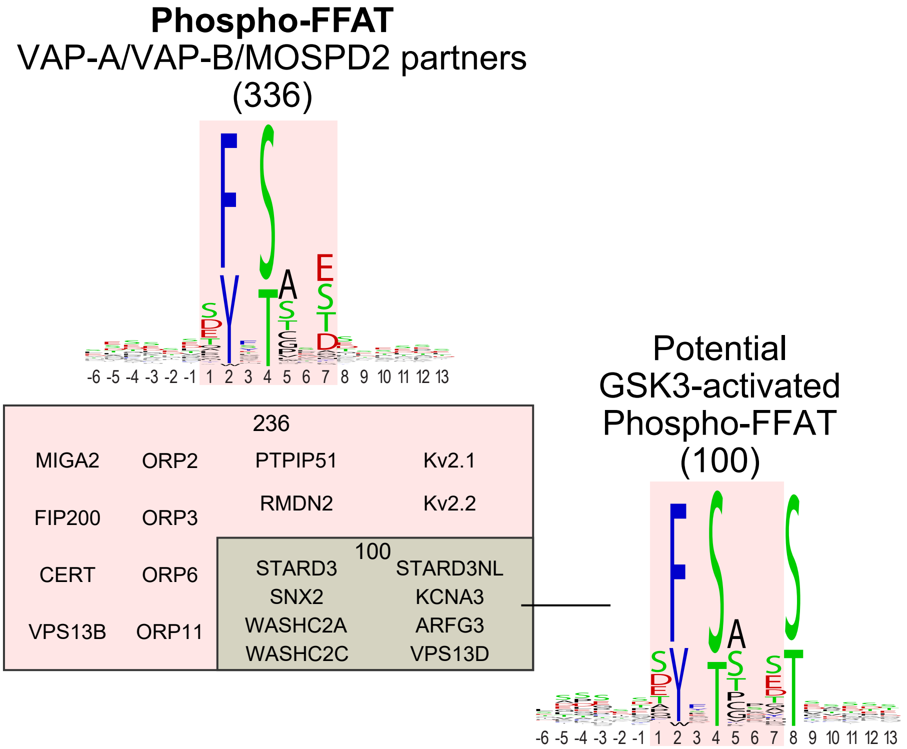

Appendix Figure S7: Proteins with a potential Phospho-FFAT possibly activated by GSK3

List of selected proteins among the 336 identified as VAPs and/or MOSPD2 partners that contain a potential Phospho-FFAT motif (see Table 1). Sequence logos (Crooks et al., 2004) represent the consensus of all 336 Phospho-FFAT motifs (top) and the subset of 100 motifs with a serine or threonine at position 8 (right).
