# Supplementary material for: STARD3 regulates lysosome positioning and contacts via a GSK3-controlled phosphorylation switch
Source: EMBO J. 2026 Feb 25;45(7):2239–77. doi: 10.1038/s44318-026-00705-3 (PMC13044316; doi:10.1038/s44318-026-00705-3)
Supplement: Supplementary file 4 — Movie EV1 [file 44318_2026_705_MOESM4_ESM.zip › Movie EV1 Legend.pdf]

Movie Legend

MovieEV1: 3D visualization of ER and LE/Lys organization in a control HeLa cell imaged by FIB-SEM. FIB-SEM volume and 3D rendering of a control HeLa cell. Segmentation highlights LE/Lys (magenta), ER (green), and mitochondria (brown).
