# Supplementary material for: STARD3 regulates lysosome positioning and contacts via a GSK3-controlled phosphorylation switch
Source: EMBO J. 2026 Feb 25;45(7):2239–77. doi: 10.1038/s44318-026-00705-3 (PMC13044316; doi:10.1038/s44318-026-00705-3)
Supplement: Supplementary file 5 — Movie EV2 [file 44318_2026_705_MOESM5_ESM.zip › Movie EV2 Legend.pdf]

Movie Legend

MovieEV2: 3D visualization of ER and LE/Lys organization in a cell expressing STARD3 imaged by FIB-SEM.

FIB-SEM volume and 3D rendering of a HeLa cells expressing WT STARD3. Segmentation highlights LE/Lys (magenta), ER (green), and mitochondria (brown).
