# Supplementary figures and images for: STARD3 regulates lysosome positioning and contacts via a GSK3-controlled phosphorylation switch (part 1 of 7)
Source: EMBO J. 2026 Feb 25;45(7):2239–77. doi: 10.1038/s44318-026-00705-3 (PMC13044316; doi:10.1038/s44318-026-00705-3)

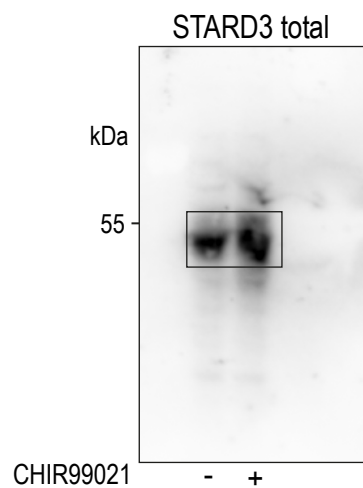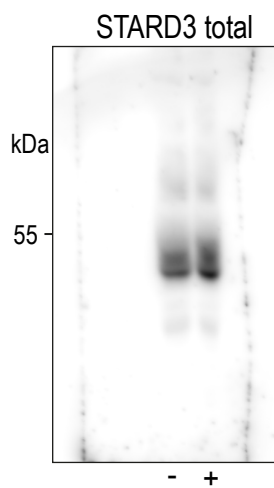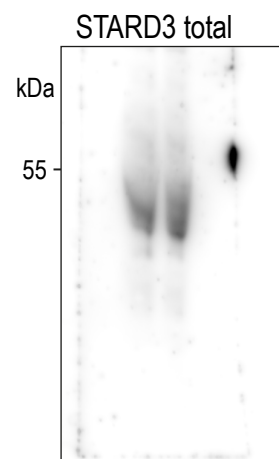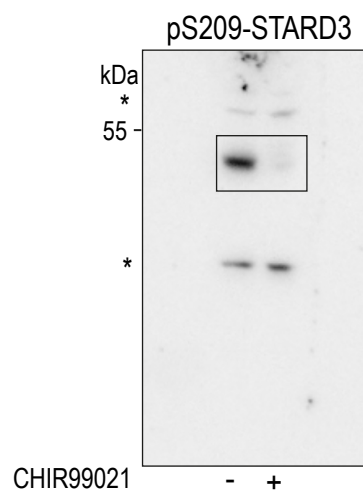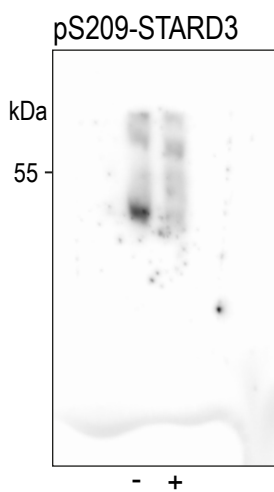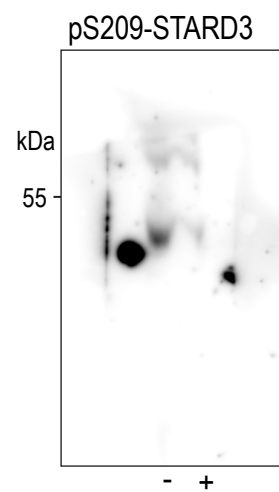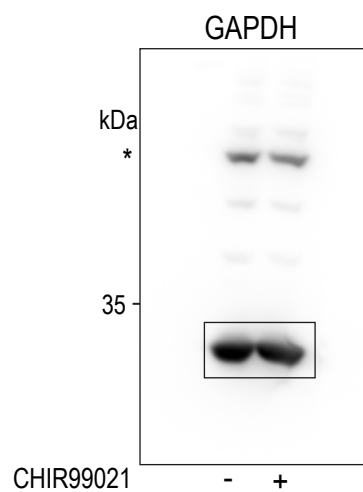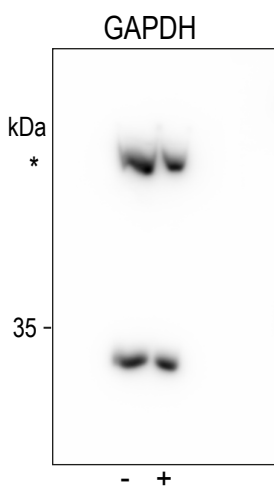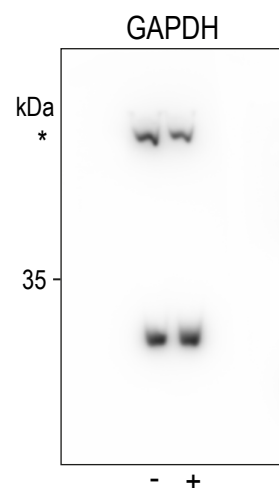

Supplement: Supplementary file 7 — Source data Fig. 1 [file 44318_2026_705_MOESM7_ESM.zip › Figure 1/B/HCC1954_STARD3_CHIR99021_WB.pdf]

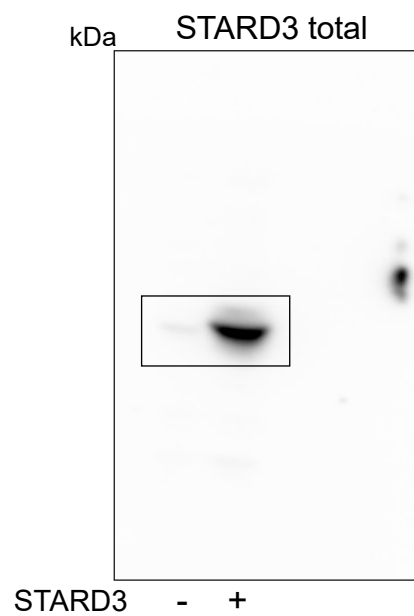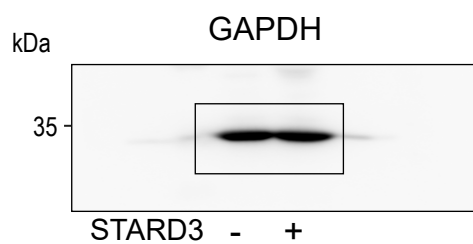

Supplement: Supplementary file 7 — Source data Fig. 1 [file 44318_2026_705_MOESM7_ESM.zip › Figure 1/C/MCF7_STARD3_expression_WB.pdf]

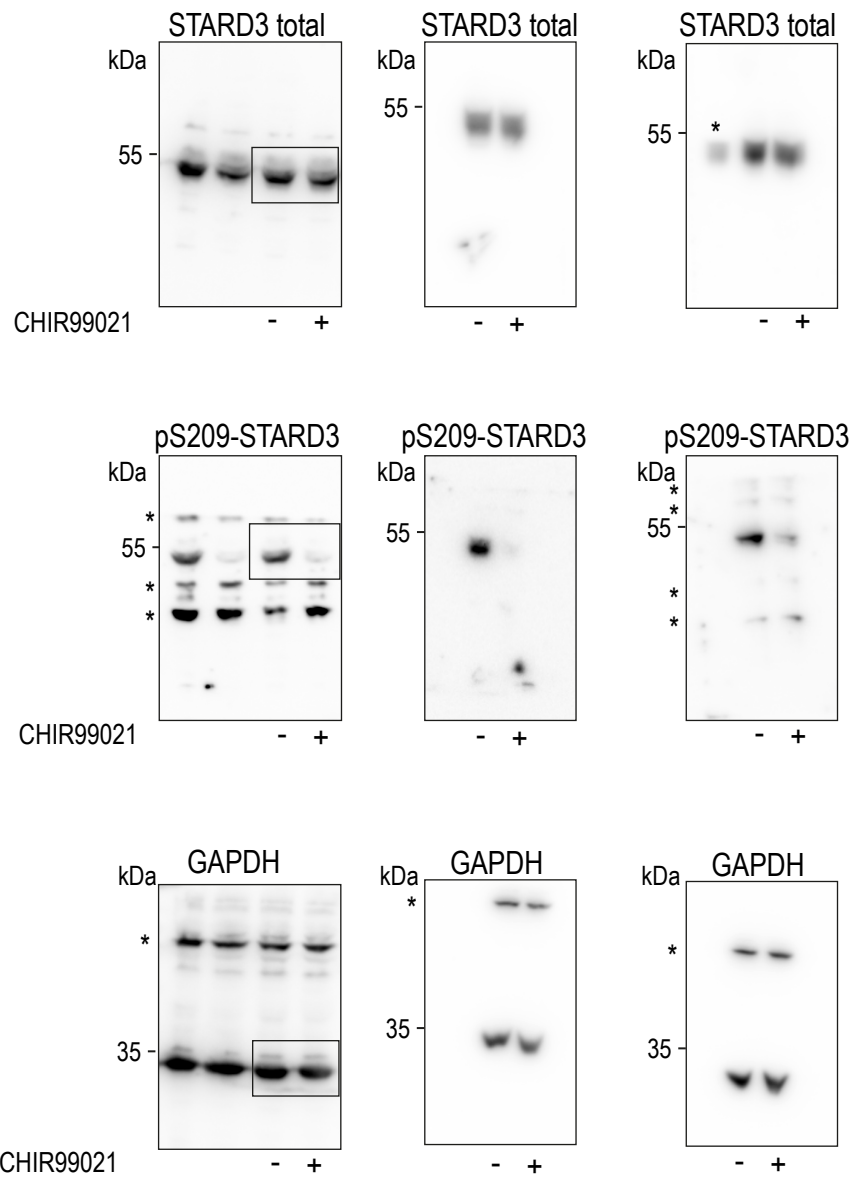

Supplement: Supplementary file 7 — Source data Fig. 1 [file 44318_2026_705_MOESM7_ESM.zip › Figure 1/D/MCF7_STARD3_CHIR99021_WB.pdf]

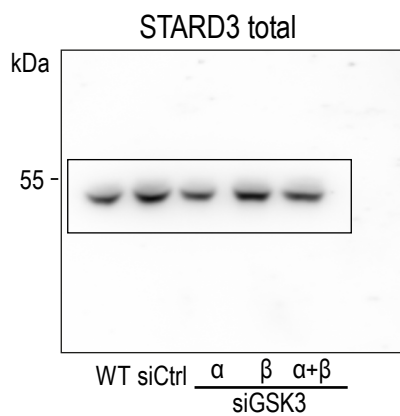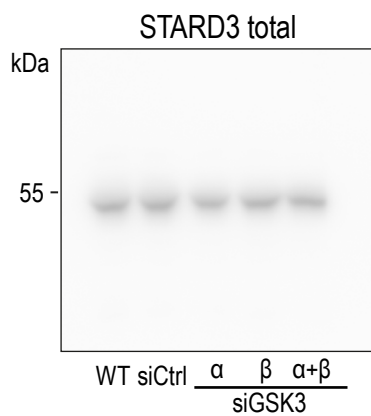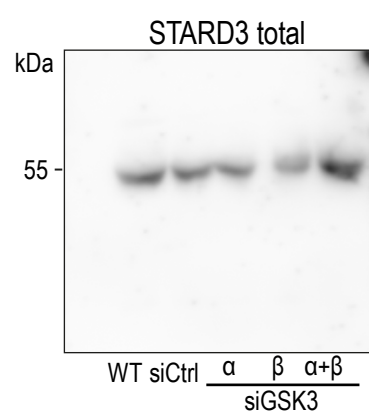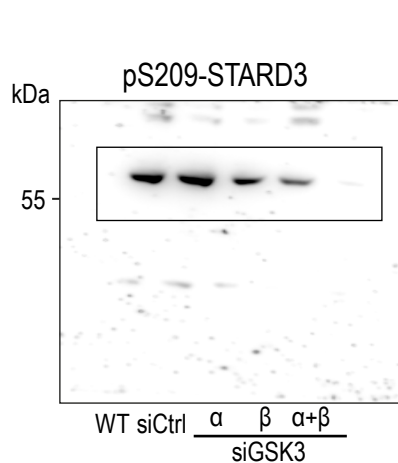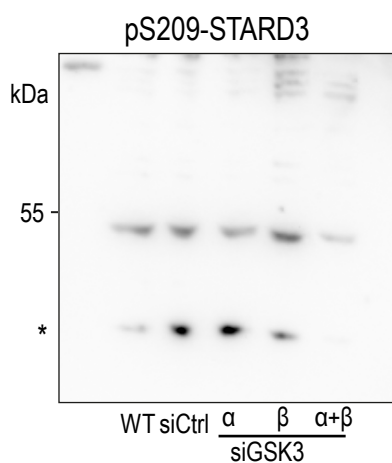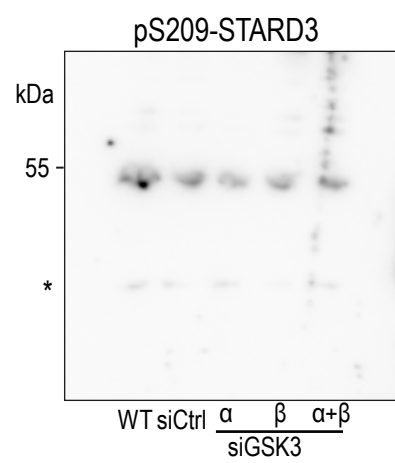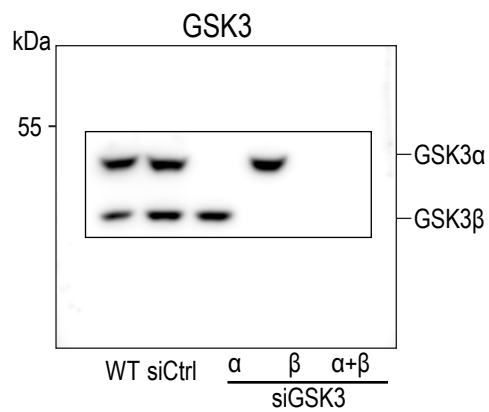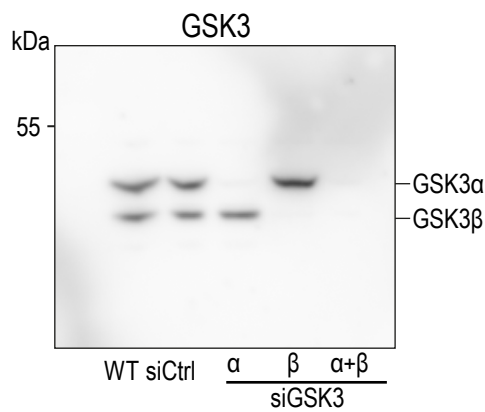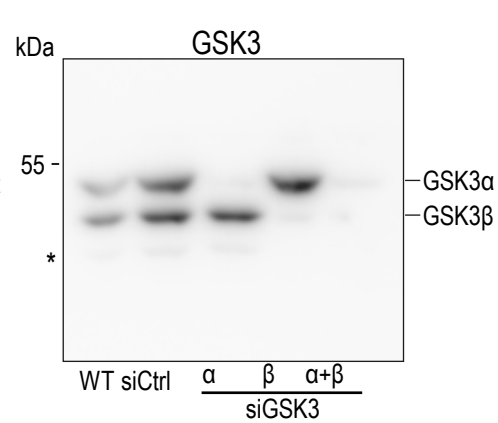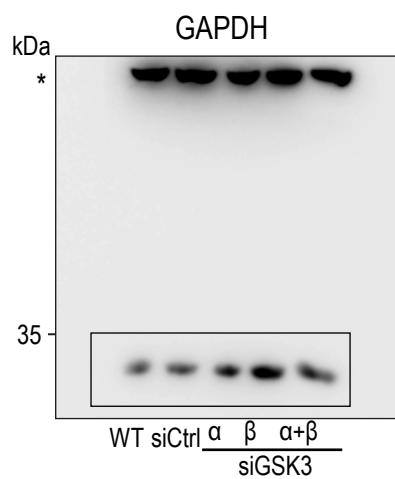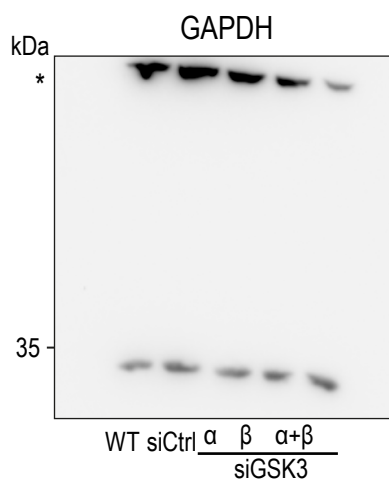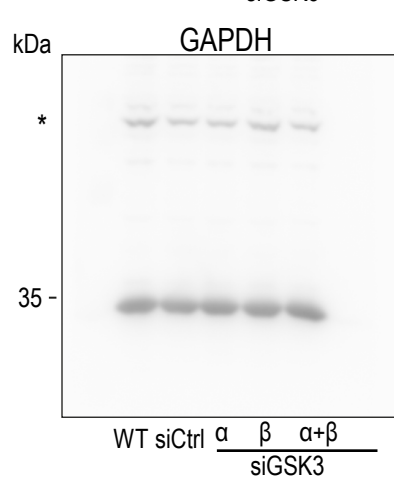

Supplement: Supplementary file 7 — Source data Fig. 1 [file 44318_2026_705_MOESM7_ESM.zip › Figure 1/E/HCC1954_siRNA_WB.pdf]

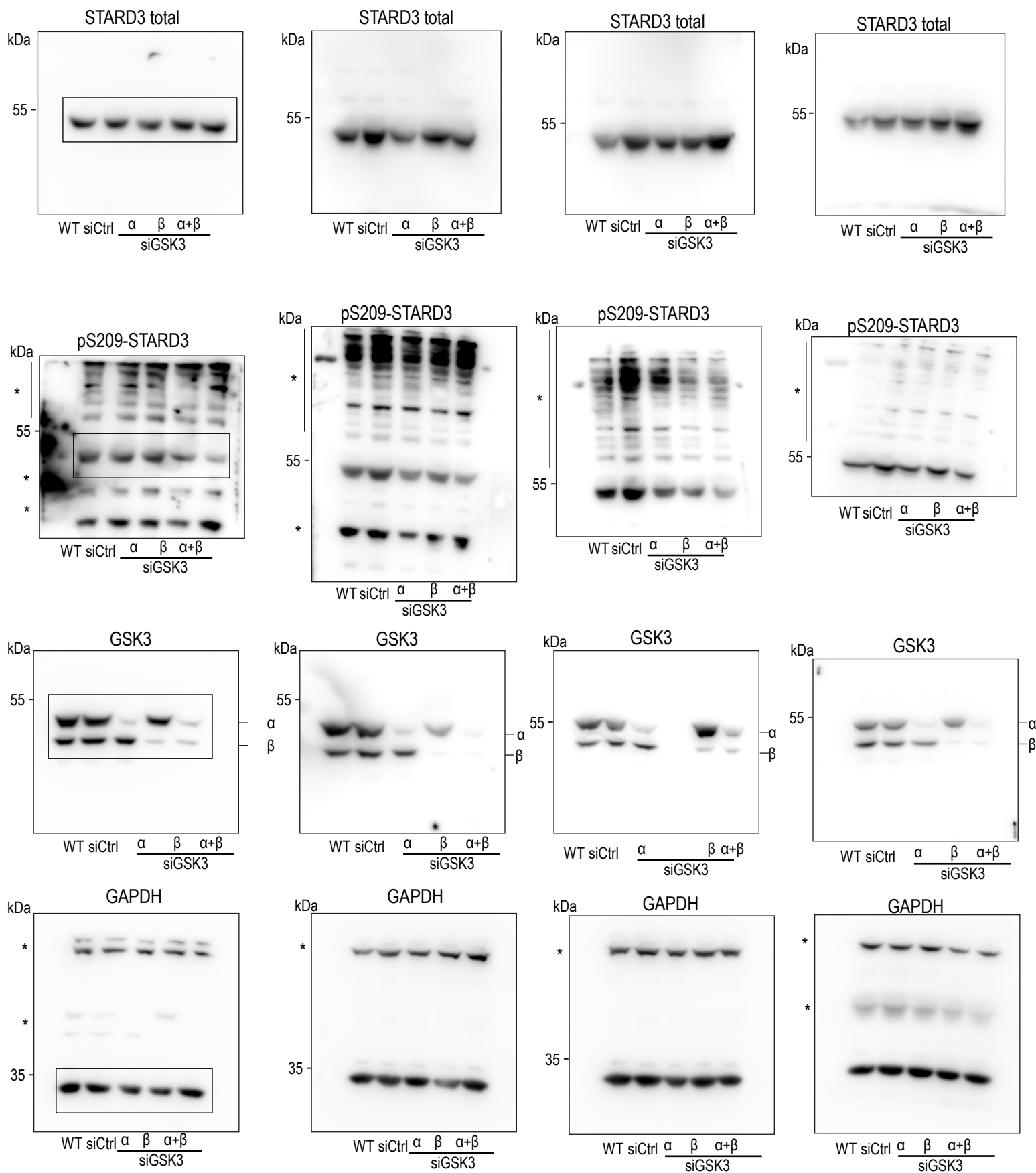

Supplement: Supplementary file 7 — Source data Fig. 1 [file 44318_2026_705_MOESM7_ESM.zip › Figure 1/F/MCF7_siRNA_WB.pdf]

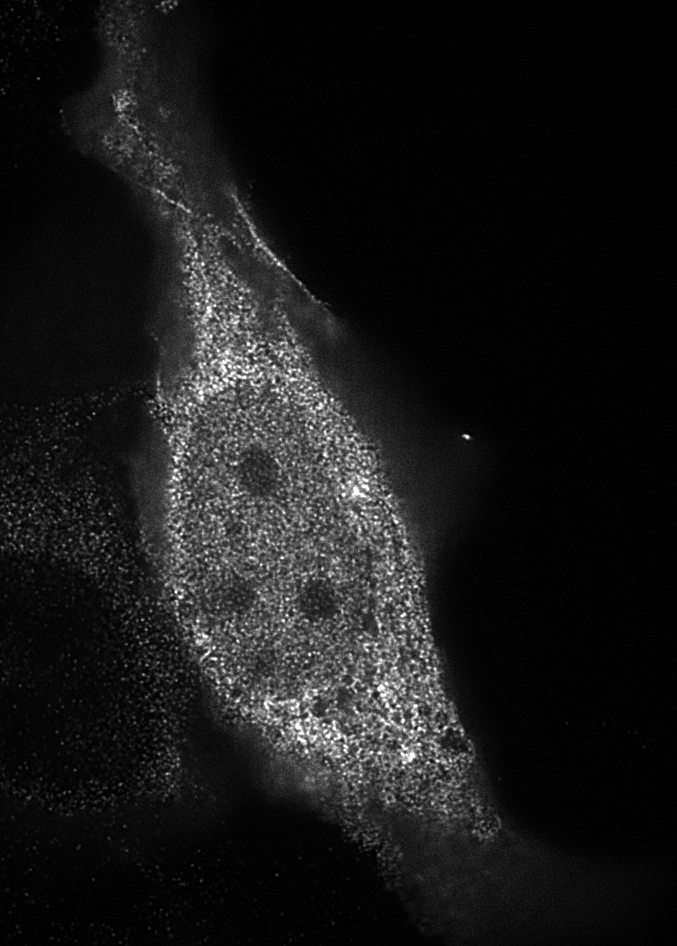

Supplement: Supplementary file 7 — Source data Fig. 1 [file 44318_2026_705_MOESM7_ESM.zip › Figure 1/G/20240506_MCF7WTGSK3_NT_2_SR_w1SPI 491 GFP.TIF]

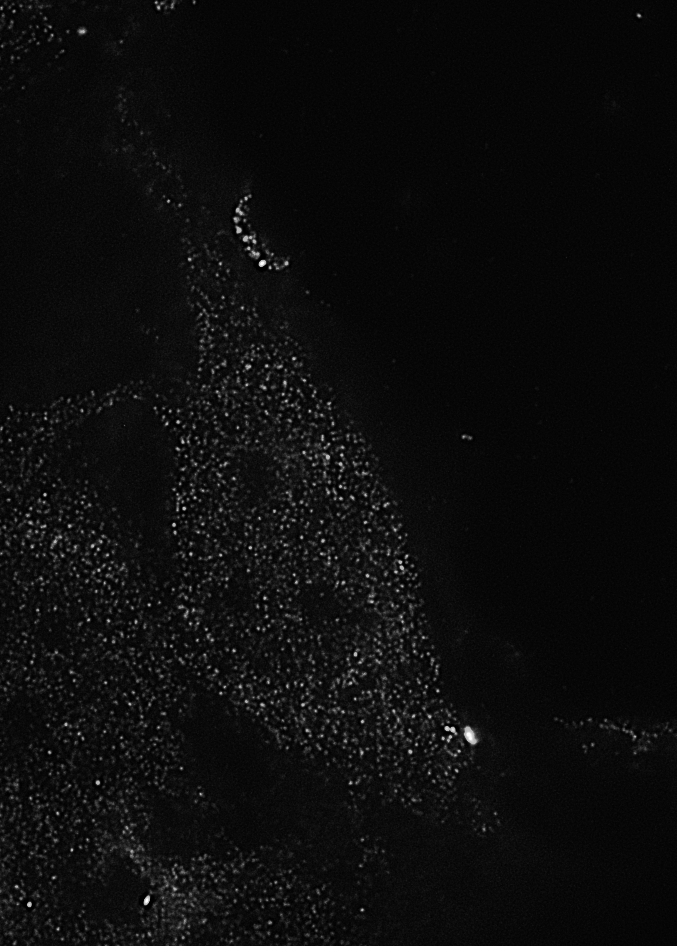

Supplement: Supplementary file 7 — Source data Fig. 1 [file 44318_2026_705_MOESM7_ESM.zip › Figure 1/G/20240506_MCF7WTGSK3_NT_2_SR_w2SPI 561 mCherry.TIF]

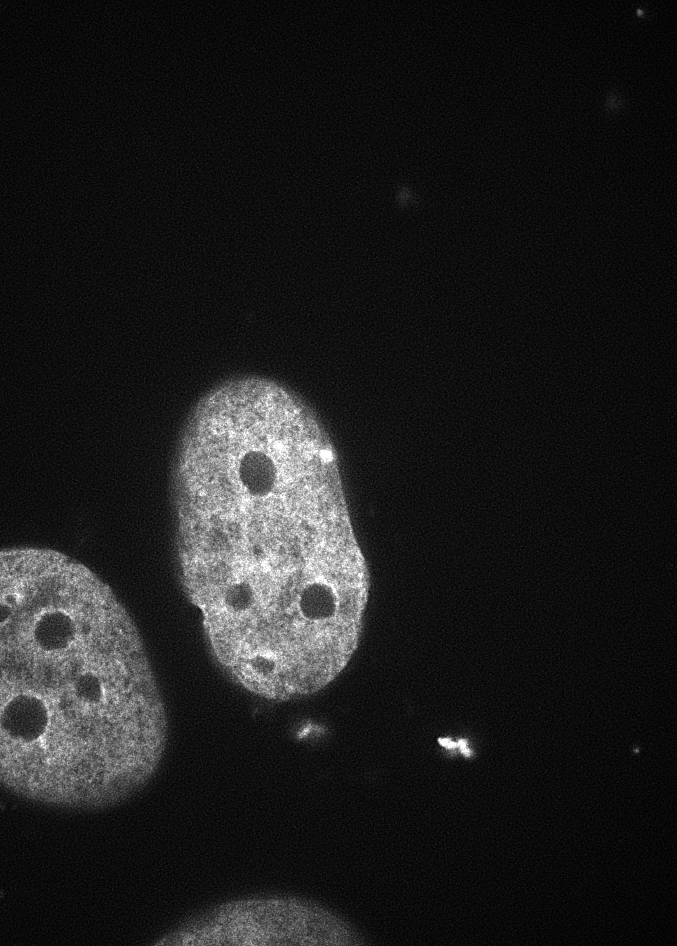

Supplement: Supplementary file 7 — Source data Fig. 1 [file 44318_2026_705_MOESM7_ESM.zip › Figure 1/G/20240506_MCF7WTGSK3_NT_2_SR_w3SPI 405 DAPI.TIF]

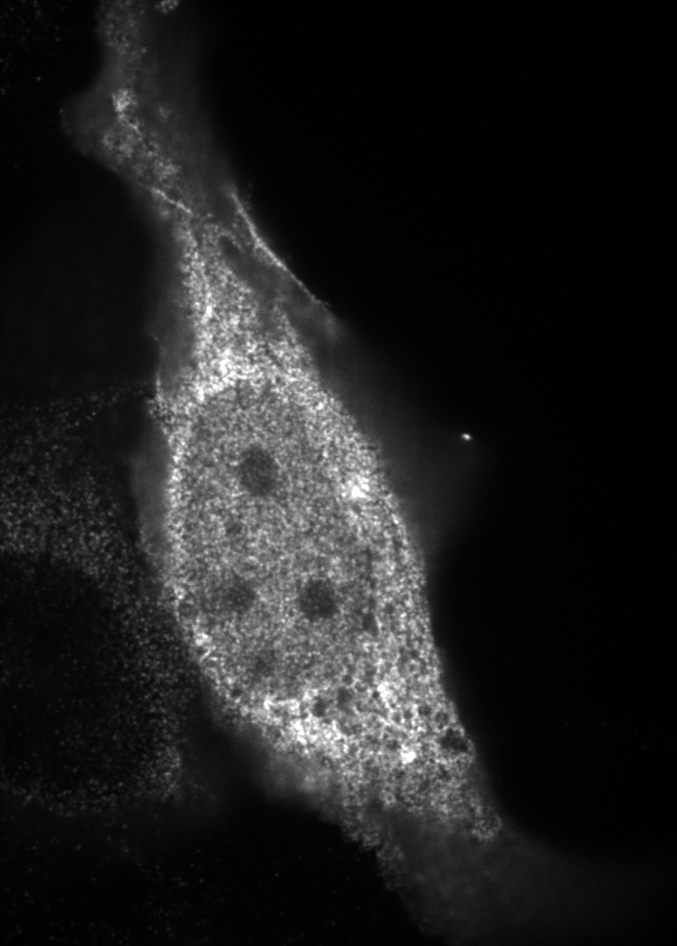

Supplement: Supplementary file 7 — Source data Fig. 1 [file 44318_2026_705_MOESM7_ESM.zip › Figure 1/G/20240506_MCF7WTGSK3_NT_2_w1SPI 491 GFP.TIF]

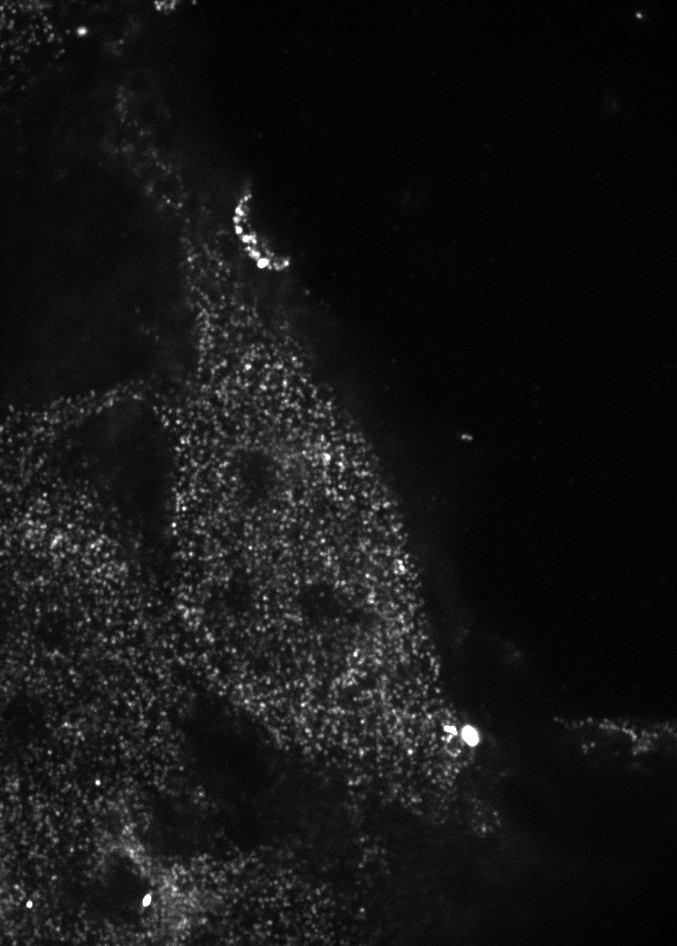

Supplement: Supplementary file 7 — Source data Fig. 1 [file 44318_2026_705_MOESM7_ESM.zip › Figure 1/G/20240506_MCF7WTGSK3_NT_2_w2SPI 561 mCherry.TIF]

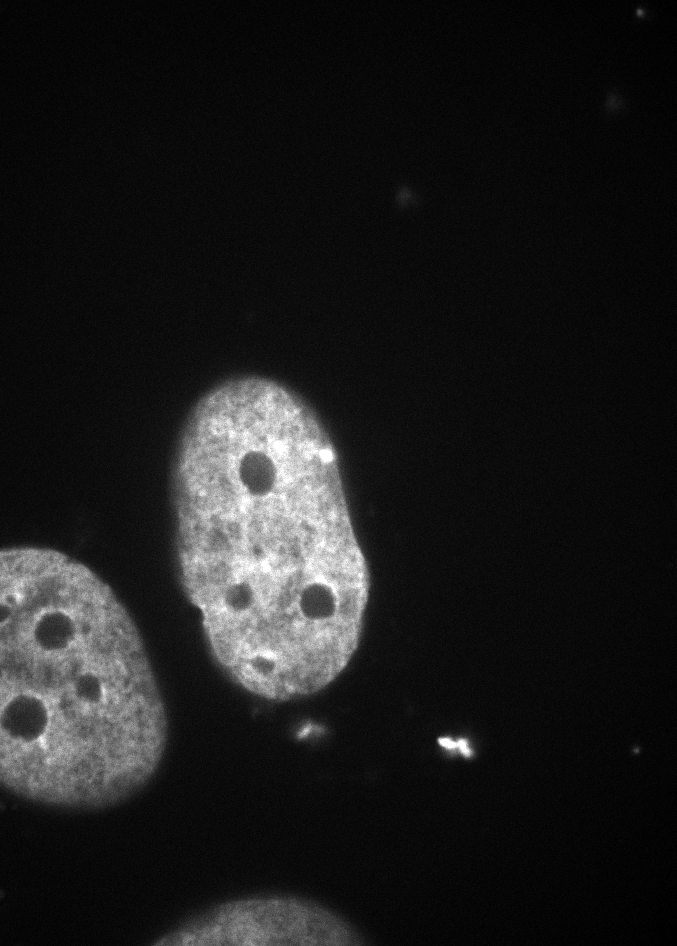

Supplement: Supplementary file 7 — Source data Fig. 1 [file 44318_2026_705_MOESM7_ESM.zip › Figure 1/G/20240506_MCF7WTGSK3_NT_2_w3SPI 405 DAPI.TIF]

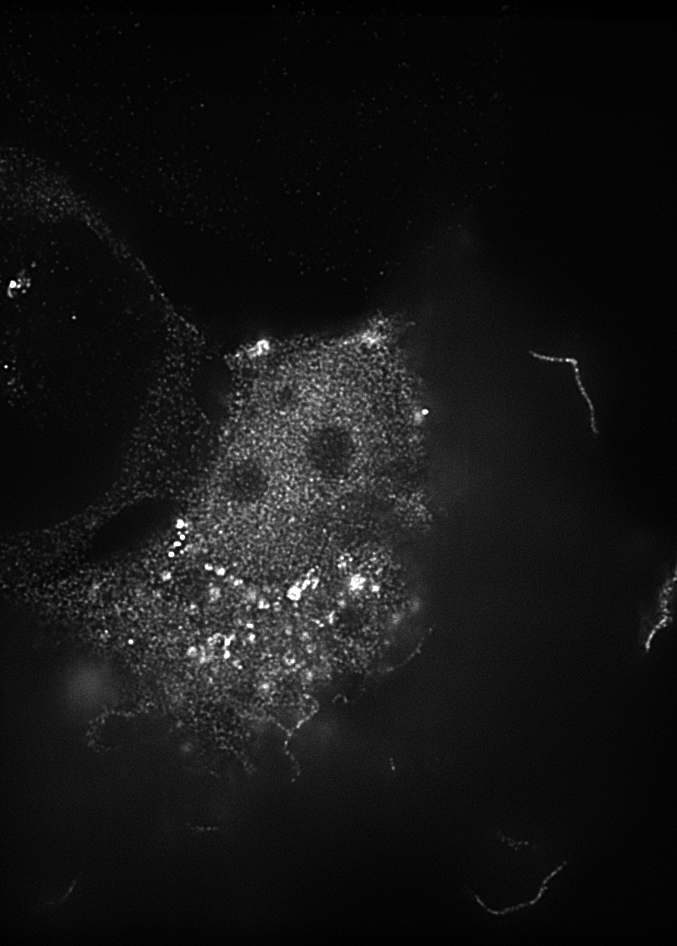

Supplement: Supplementary file 7 — Source data Fig. 1 [file 44318_2026_705_MOESM7_ESM.zip › Figure 1/H/20240201_GSK3WT_S_antiHAold_STARD3WT_5_SR_w1SPI 491 GFP.TIF]

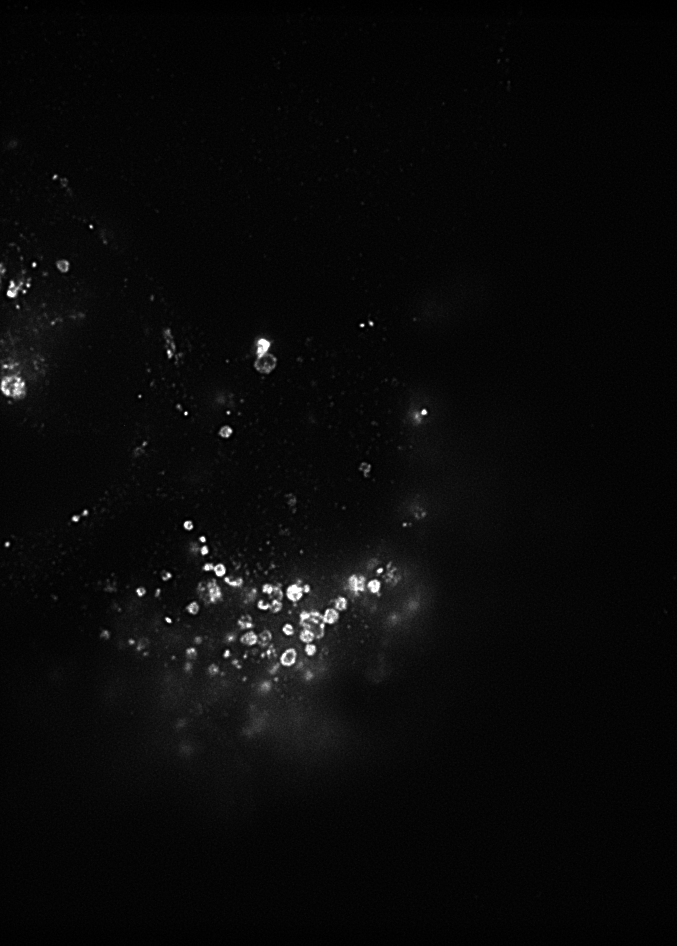

Supplement: Supplementary file 7 — Source data Fig. 1 [file 44318_2026_705_MOESM7_ESM.zip › Figure 1/H/20240201_GSK3WT_S_antiHAold_STARD3WT_5_SR_w2SPI 561 mCherry.TIF]

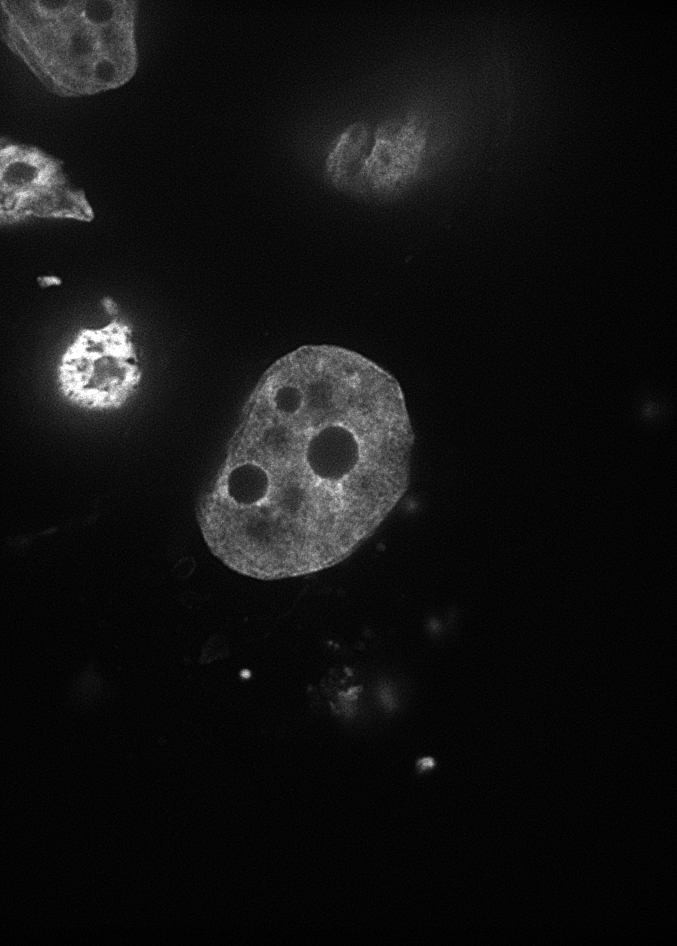

Supplement: Supplementary file 7 — Source data Fig. 1 [file 44318_2026_705_MOESM7_ESM.zip › Figure 1/H/20240201_GSK3WT_S_antiHAold_STARD3WT_5_SR_w3SPI 405 DAPI.TIF]

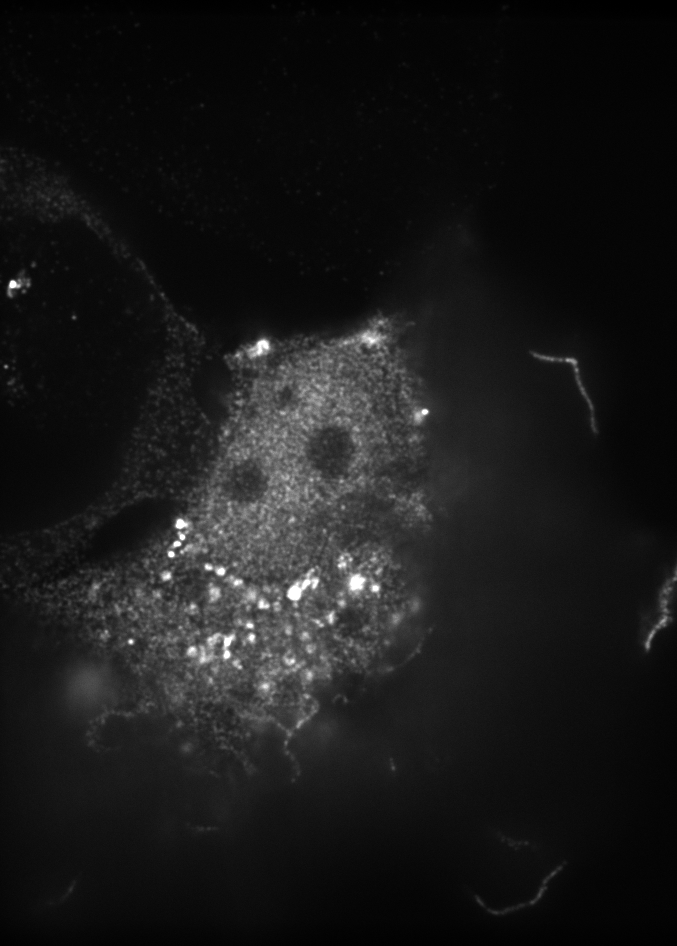

Supplement: Supplementary file 7 — Source data Fig. 1 [file 44318_2026_705_MOESM7_ESM.zip › Figure 1/H/20240201_GSK3WT_S_antiHAold_STARD3WT_5_w1SPI 491 GFP.TIF]

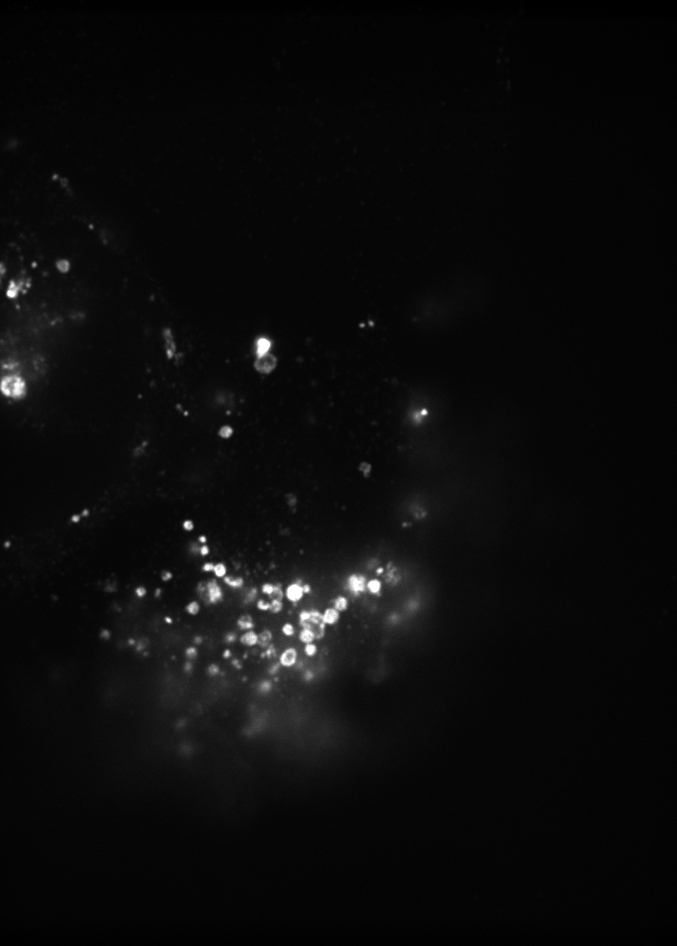

Supplement: Supplementary file 7 — Source data Fig. 1 [file 44318_2026_705_MOESM7_ESM.zip › Figure 1/H/20240201_GSK3WT_S_antiHAold_STARD3WT_5_w2SPI 561 mCherry.TIF]

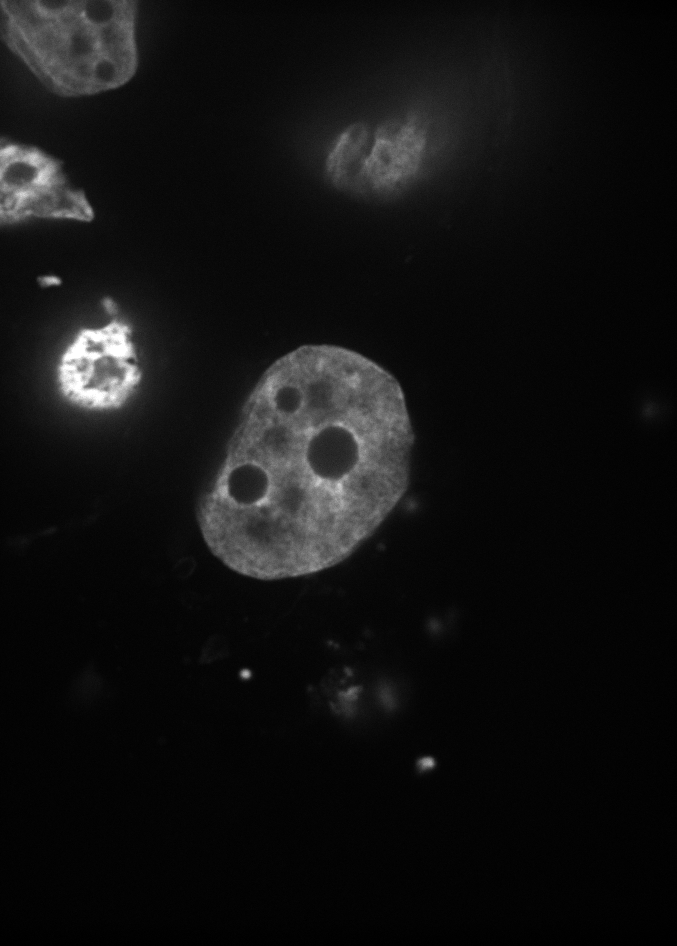

Supplement: Supplementary file 7 — Source data Fig. 1 [file 44318_2026_705_MOESM7_ESM.zip › Figure 1/H/20240201_GSK3WT_S_antiHAold_STARD3WT_5_w3SPI 405 DAPI.TIF]

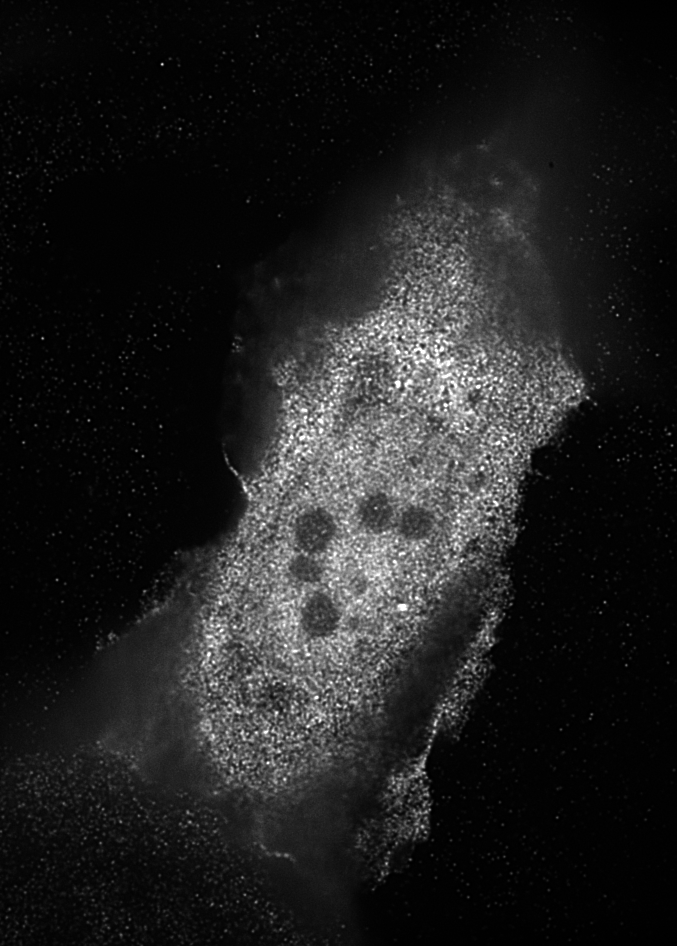

Supplement: Supplementary file 7 — Source data Fig. 1 [file 44318_2026_705_MOESM7_ESM.zip › Figure 1/I/20240202_MCF7GSK3WT_STARD3S209A_5_SR_w1SPI 491 GFP.TIF]

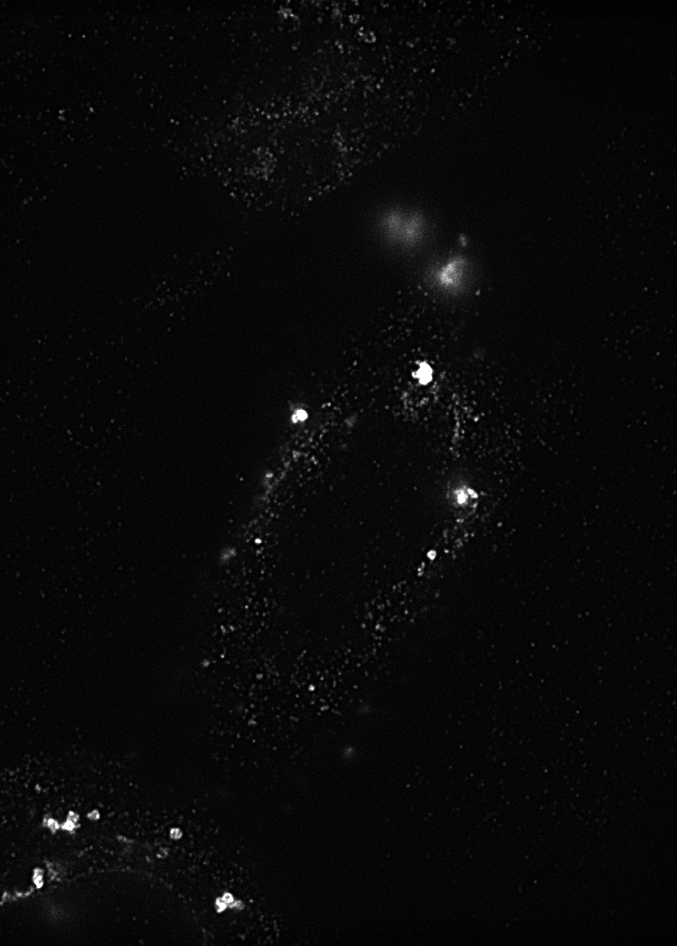

Supplement: Supplementary file 7 — Source data Fig. 1 [file 44318_2026_705_MOESM7_ESM.zip › Figure 1/I/20240202_MCF7GSK3WT_STARD3S209A_5_SR_w2SPI 561 mCherry.TIF]

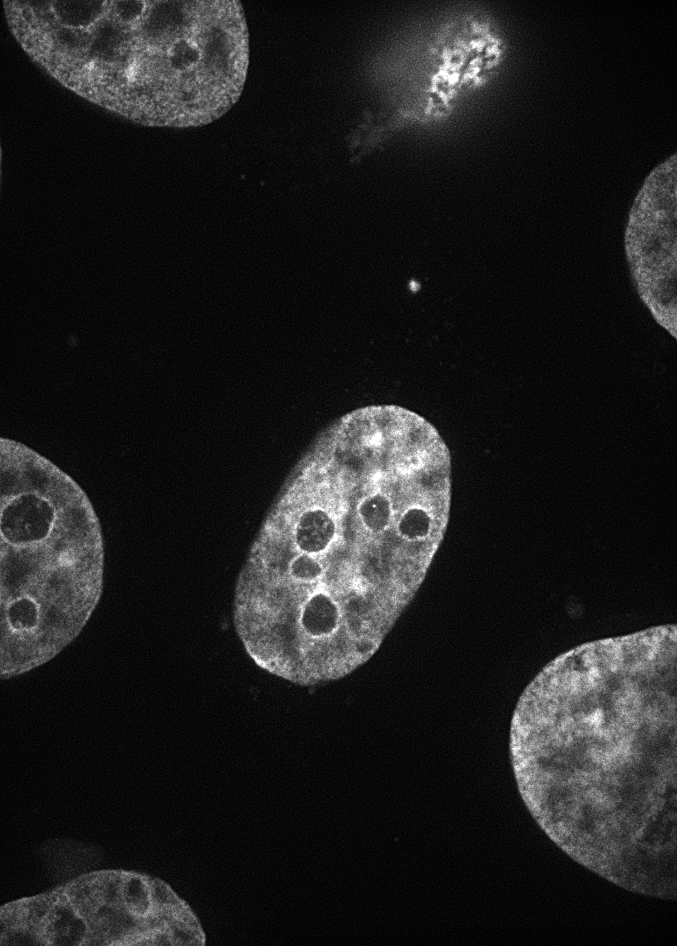

Supplement: Supplementary file 7 — Source data Fig. 1 [file 44318_2026_705_MOESM7_ESM.zip › Figure 1/I/20240202_MCF7GSK3WT_STARD3S209A_5_SR_w3SPI 405 DAPI.TIF]

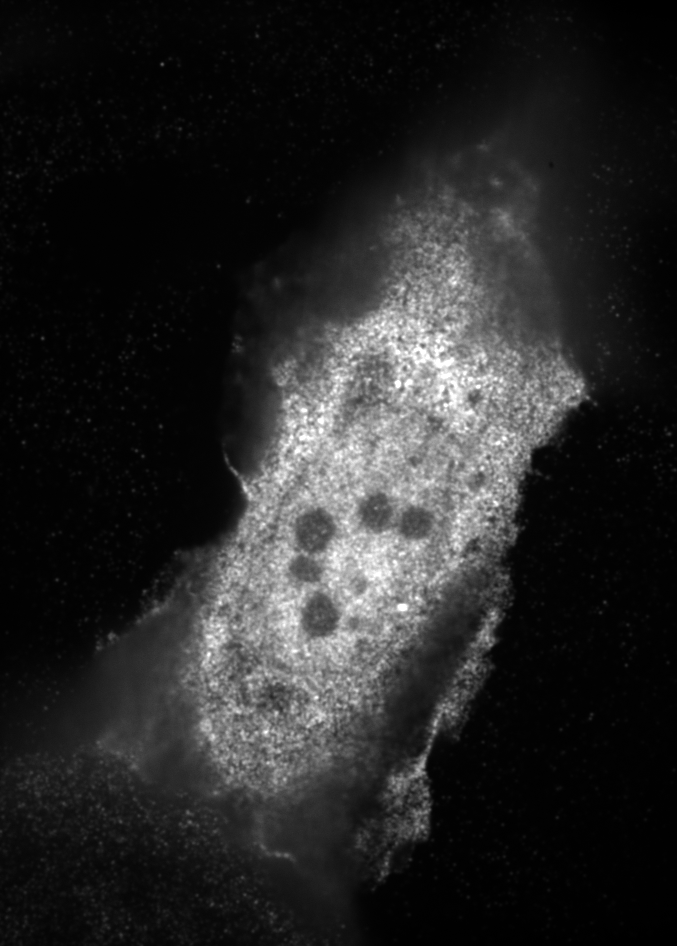

Supplement: Supplementary file 7 — Source data Fig. 1 [file 44318_2026_705_MOESM7_ESM.zip › Figure 1/I/20240202_MCF7GSK3WT_STARD3S209A_5_w1SPI 491 GFP.TIF]

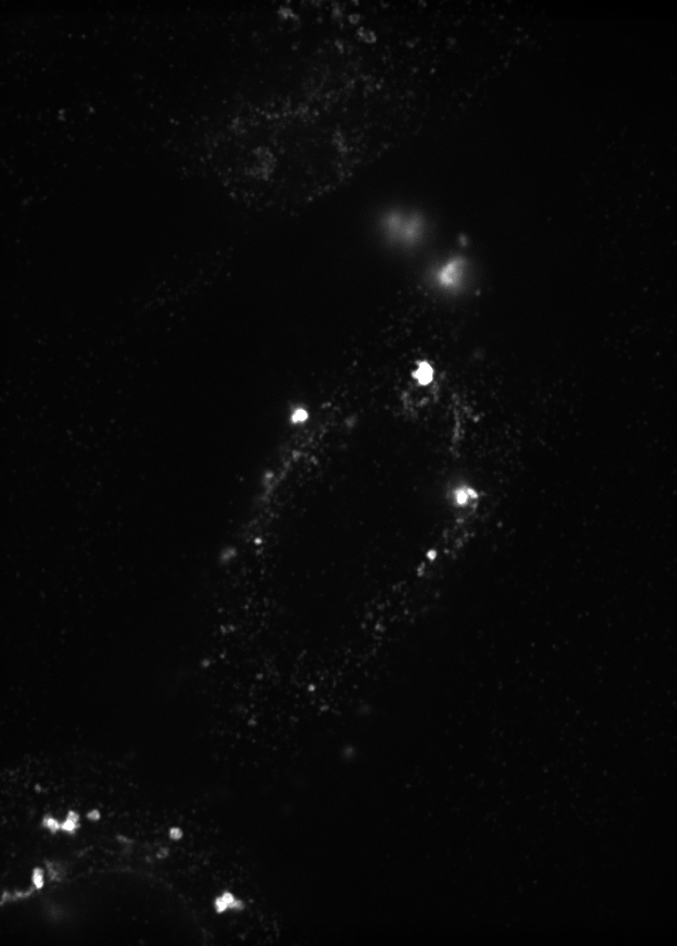

Supplement: Supplementary file 7 — Source data Fig. 1 [file 44318_2026_705_MOESM7_ESM.zip › Figure 1/I/20240202_MCF7GSK3WT_STARD3S209A_5_w2SPI 561 mCherry.TIF]

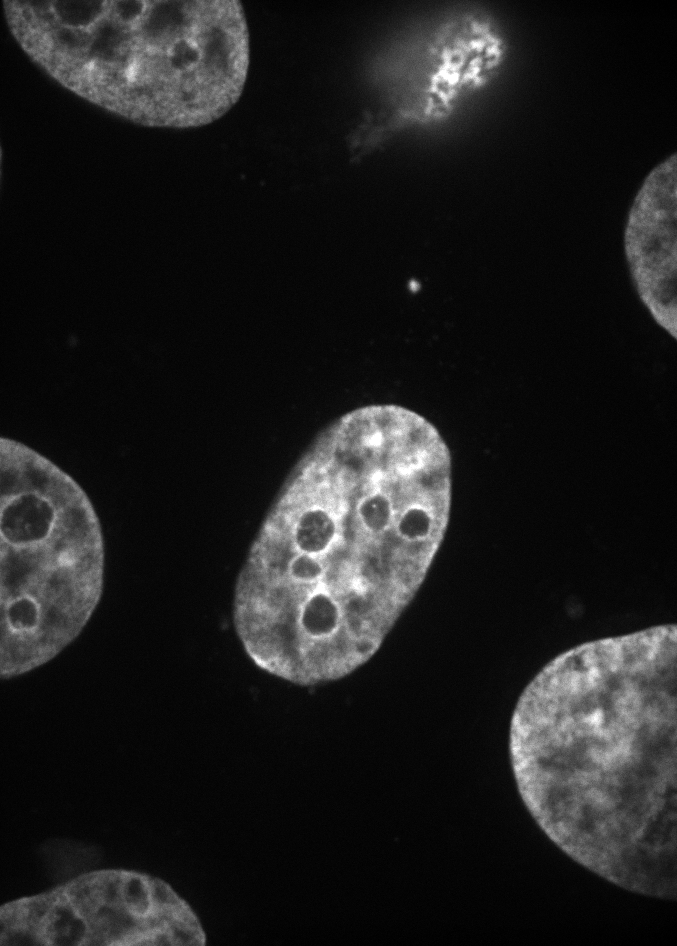

Supplement: Supplementary file 7 — Source data Fig. 1 [file 44318_2026_705_MOESM7_ESM.zip › Figure 1/I/20240202_MCF7GSK3WT_STARD3S209A_5_w3SPI 405 DAPI.TIF]

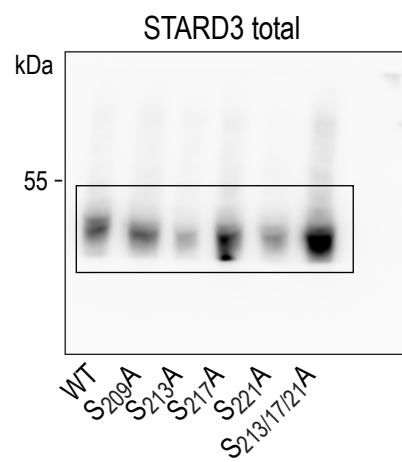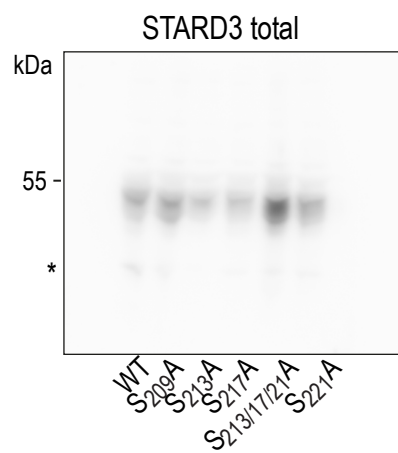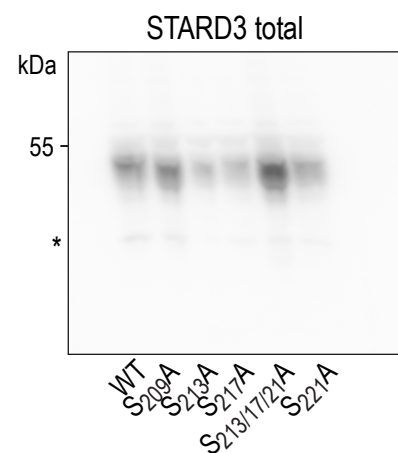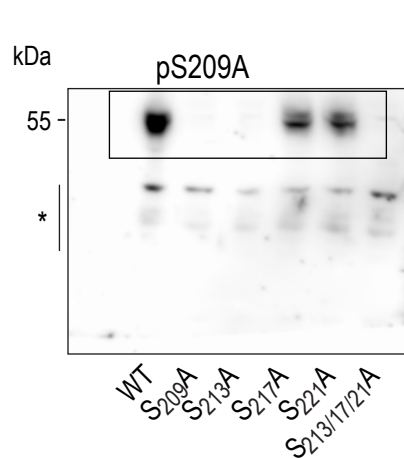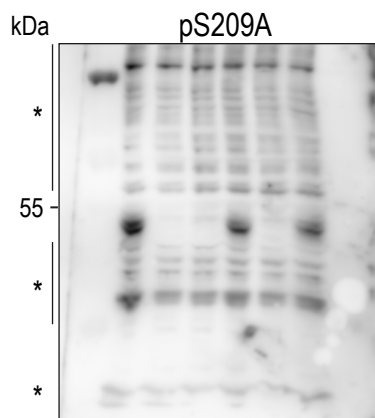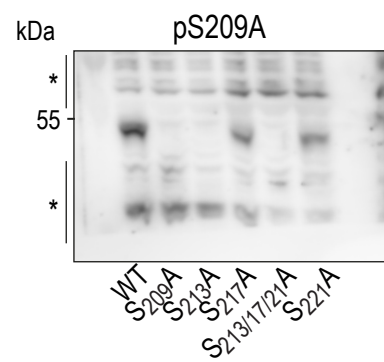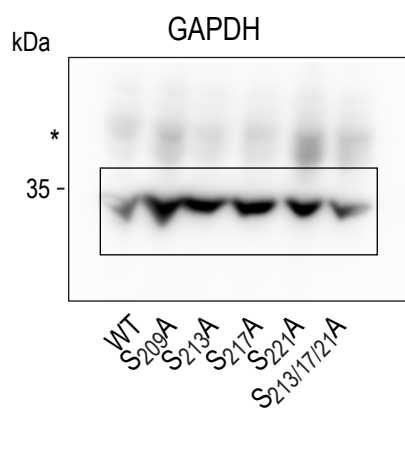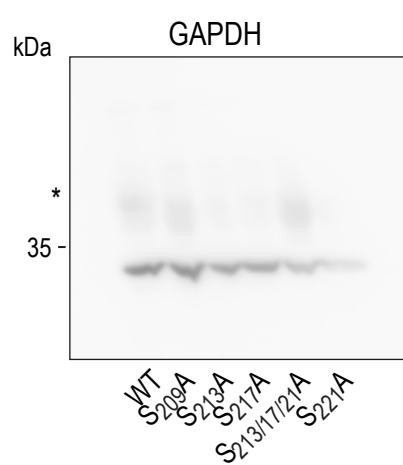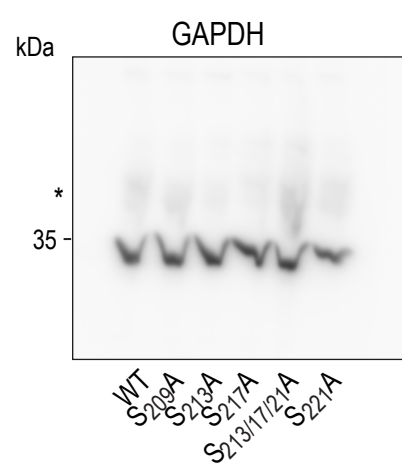

Supplement: Supplementary file 8 — Source data Fig. 2 [file 44318_2026_705_MOESM8_ESM.zip › Figure 2/B/MCF7_priming_WB.pdf]

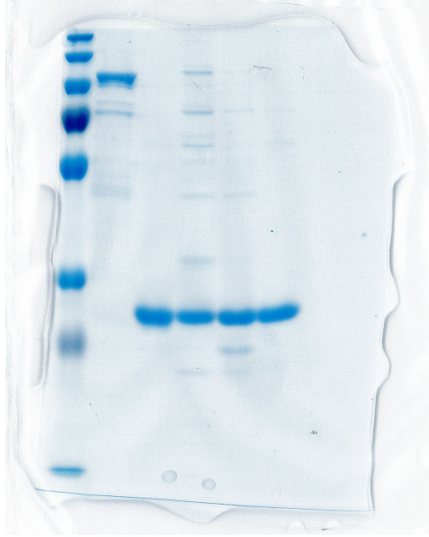

Supplement: Supplementary file 8 — Source data Fig. 2 [file 44318_2026_705_MOESM8_ESM.zip › Figure 2/E/Coomassie blue.pdf]

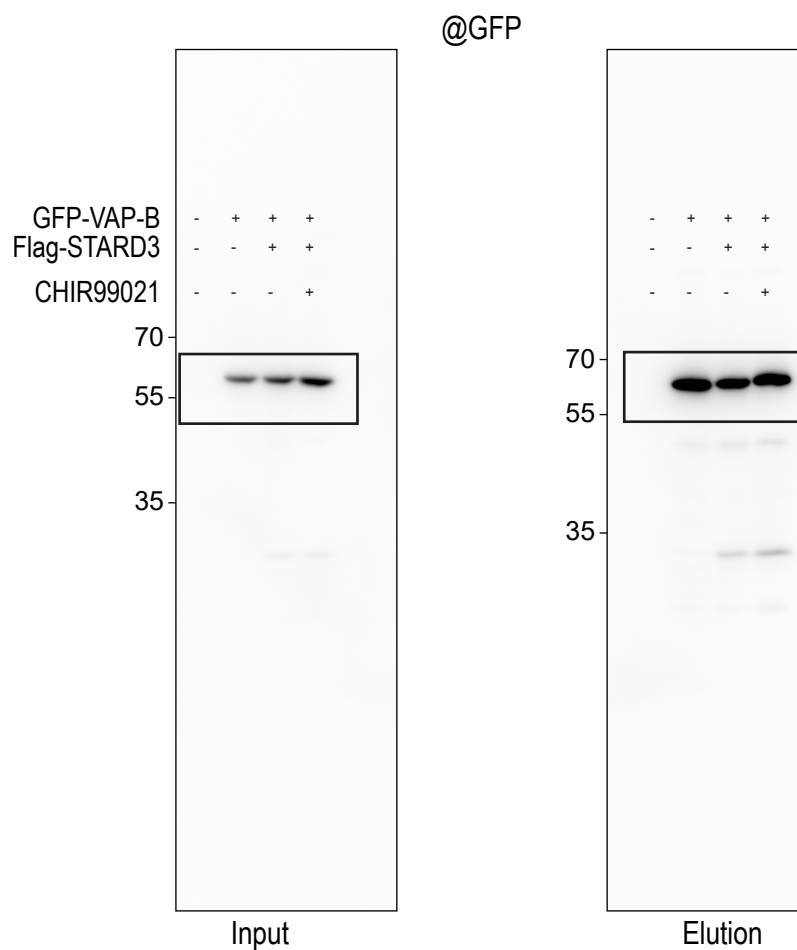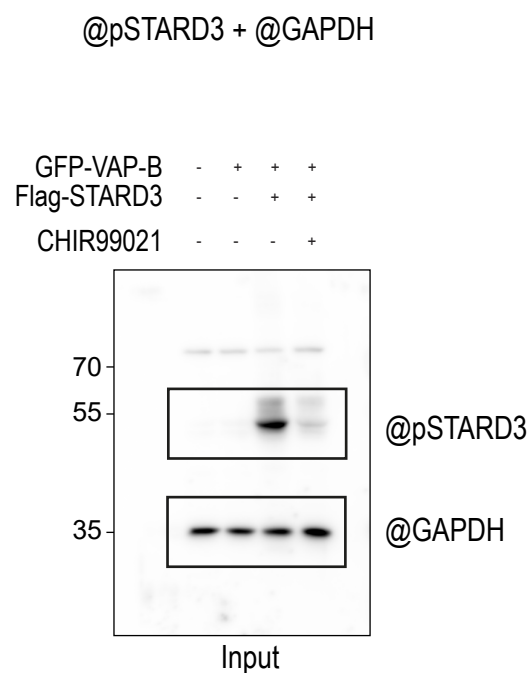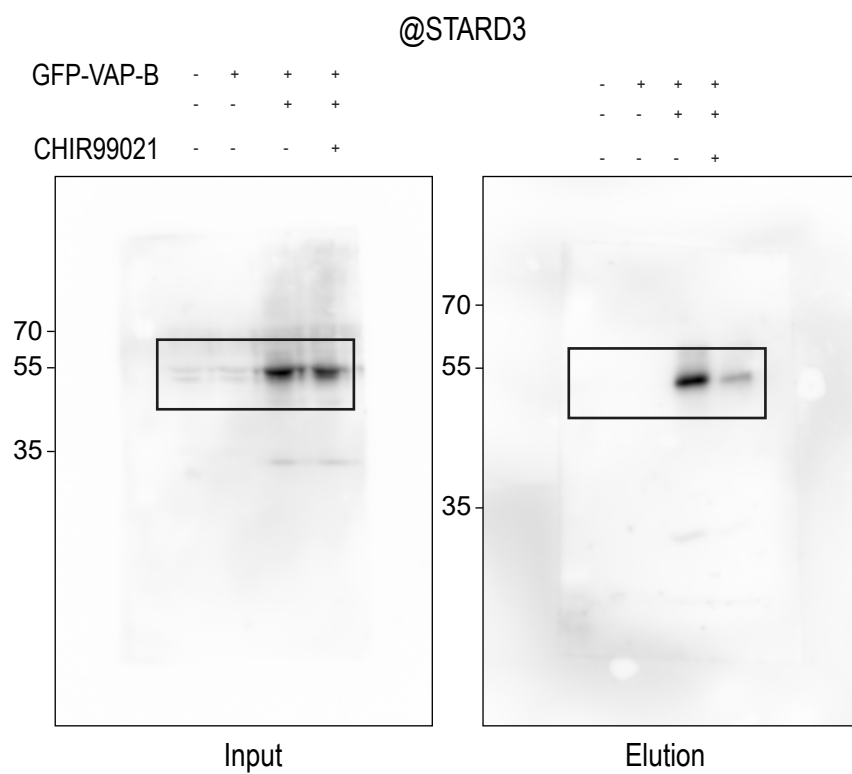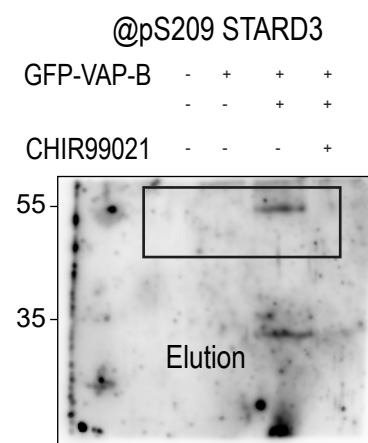

Supplement: Supplementary file 9 — Source data Fig. 3 [file 44318_2026_705_MOESM9_ESM.zip › Figure 3/B/GFP Trap-VAPB_WB.pdf]

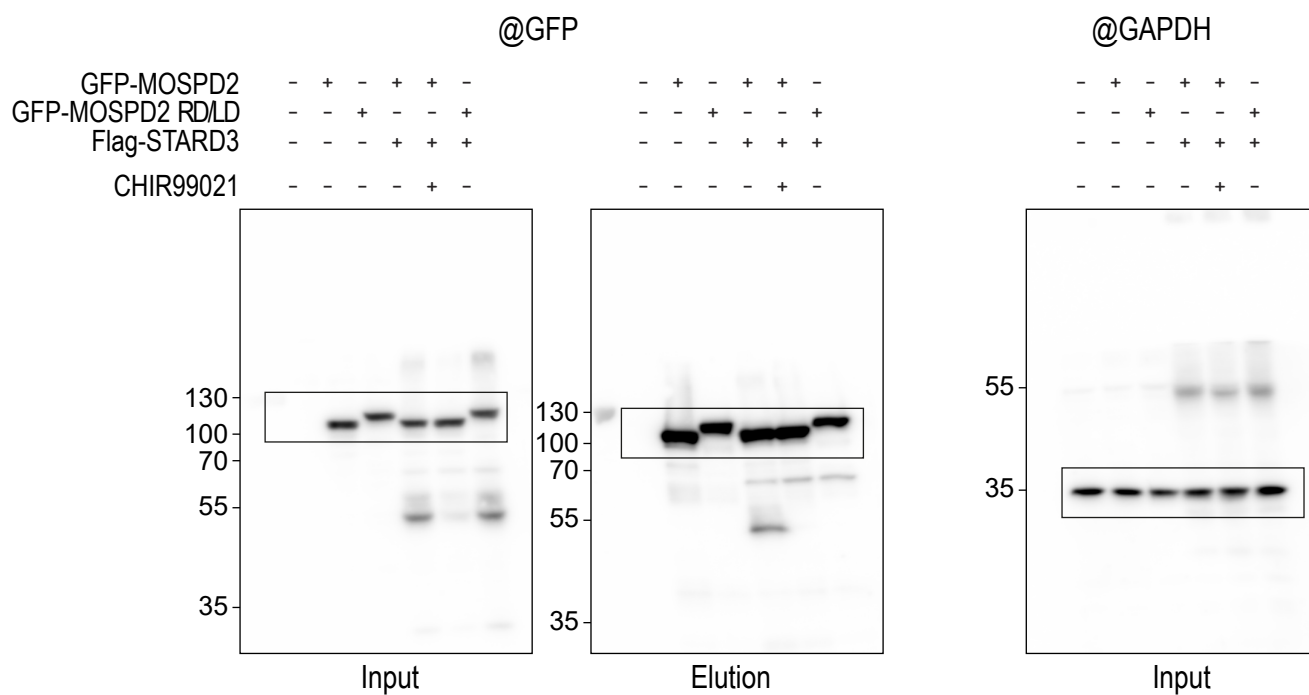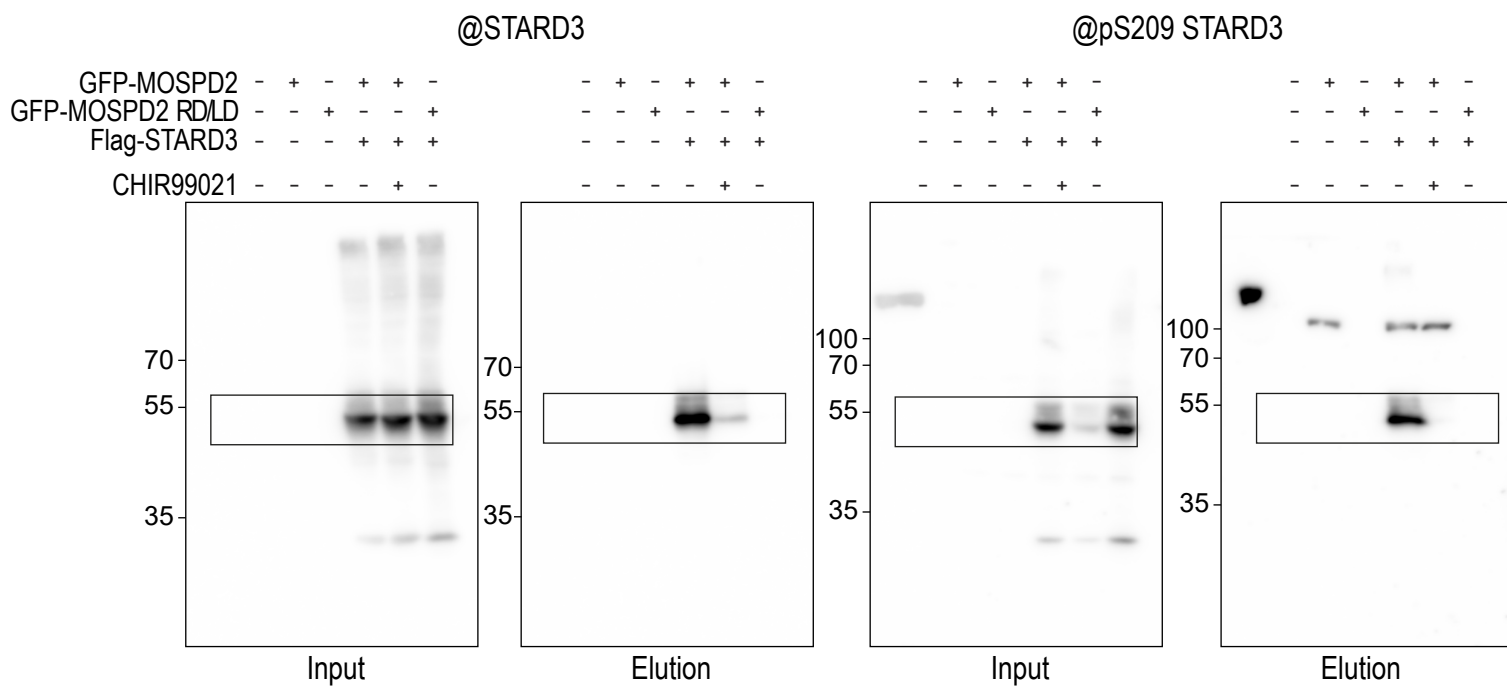

Supplement: Supplementary file 9 — Source data Fig. 3 [file 44318_2026_705_MOESM9_ESM.zip › Figure 3/C/GFP Trap-MOSPD2_WB.pdf]

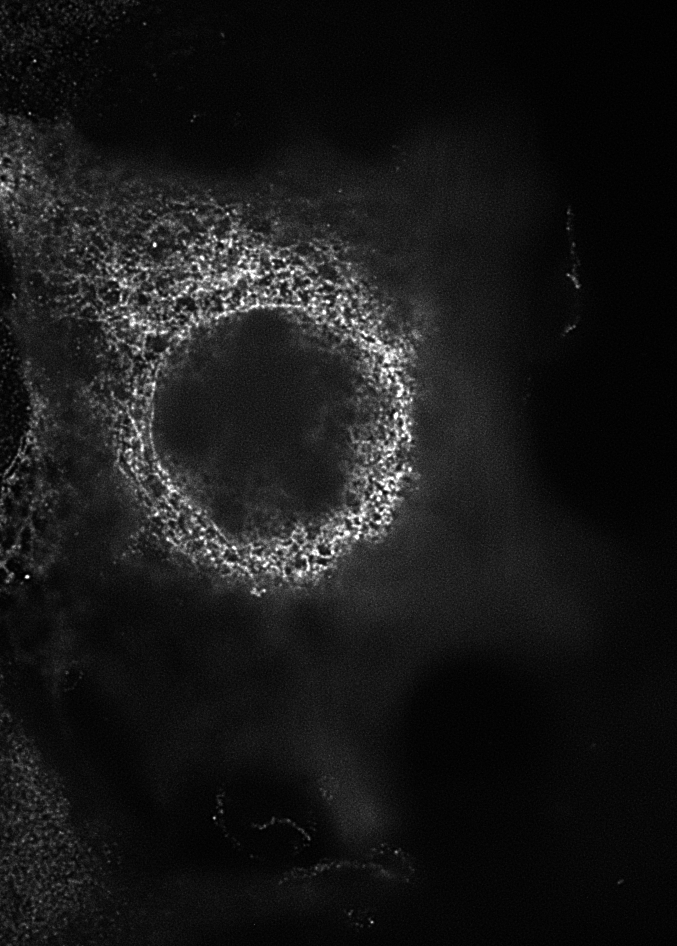

Supplement: Supplementary file 9 — Source data Fig. 3 [file 44318_2026_705_MOESM9_ESM.zip › Figure 3/F/20250821_MCF7VAPAGFP_NT_1_SR_w1SPI 491 GFP.TIF]

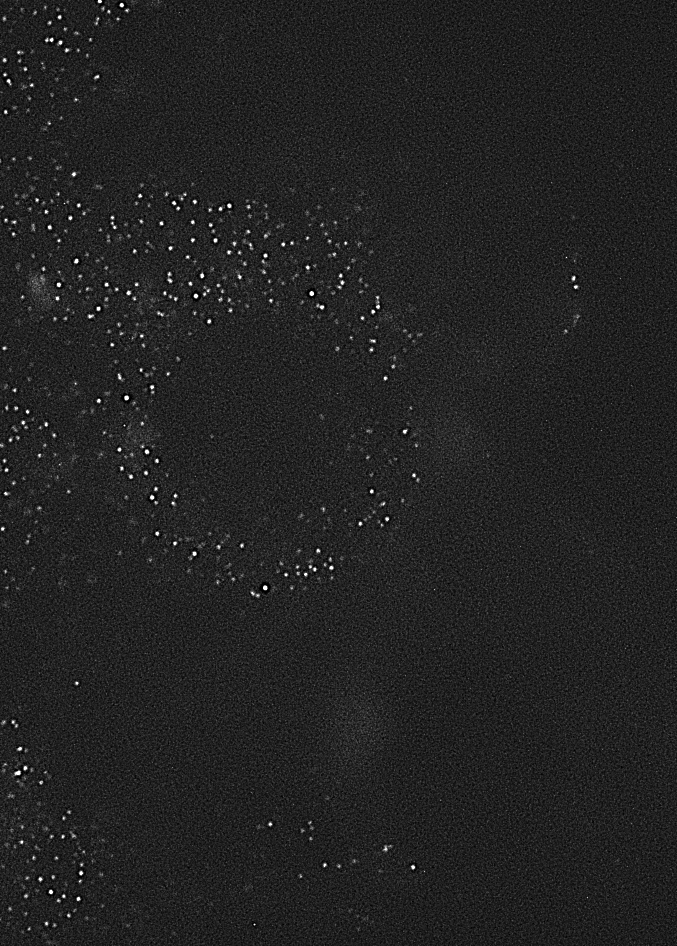

Supplement: Supplementary file 9 — Source data Fig. 3 [file 44318_2026_705_MOESM9_ESM.zip › Figure 3/F/20250821_MCF7VAPAGFP_NT_1_SR_w2SPI 561 mCherry.TIF]

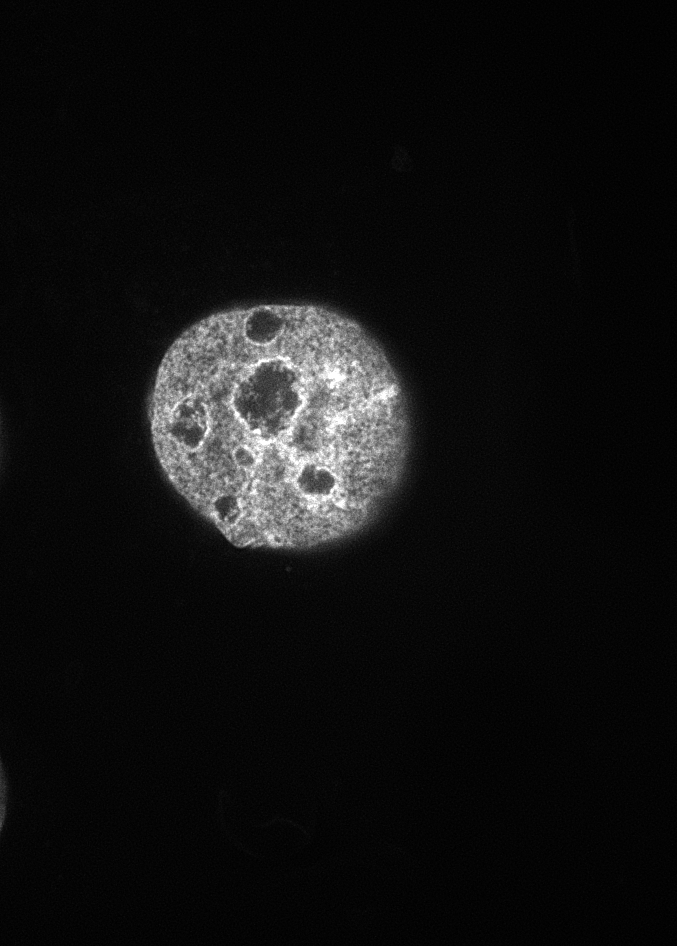

Supplement: Supplementary file 9 — Source data Fig. 3 [file 44318_2026_705_MOESM9_ESM.zip › Figure 3/F/20250821_MCF7VAPAGFP_NT_1_SR_w3SPI 405 DAPI.TIF]

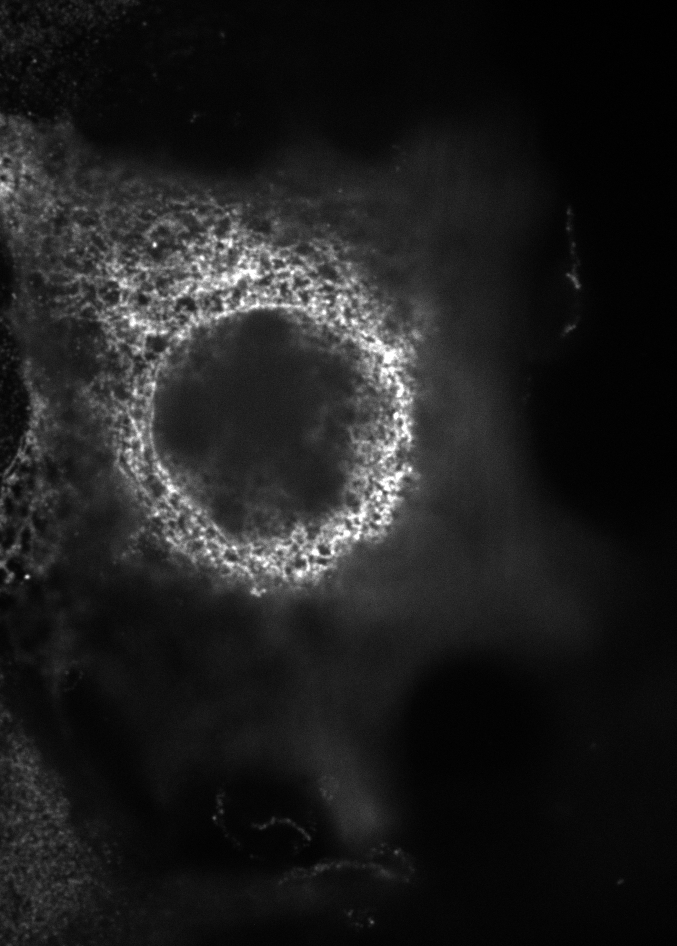

Supplement: Supplementary file 9 — Source data Fig. 3 [file 44318_2026_705_MOESM9_ESM.zip › Figure 3/F/20250821_MCF7VAPAGFP_NT_1_w1SPI 491 GFP.TIF]

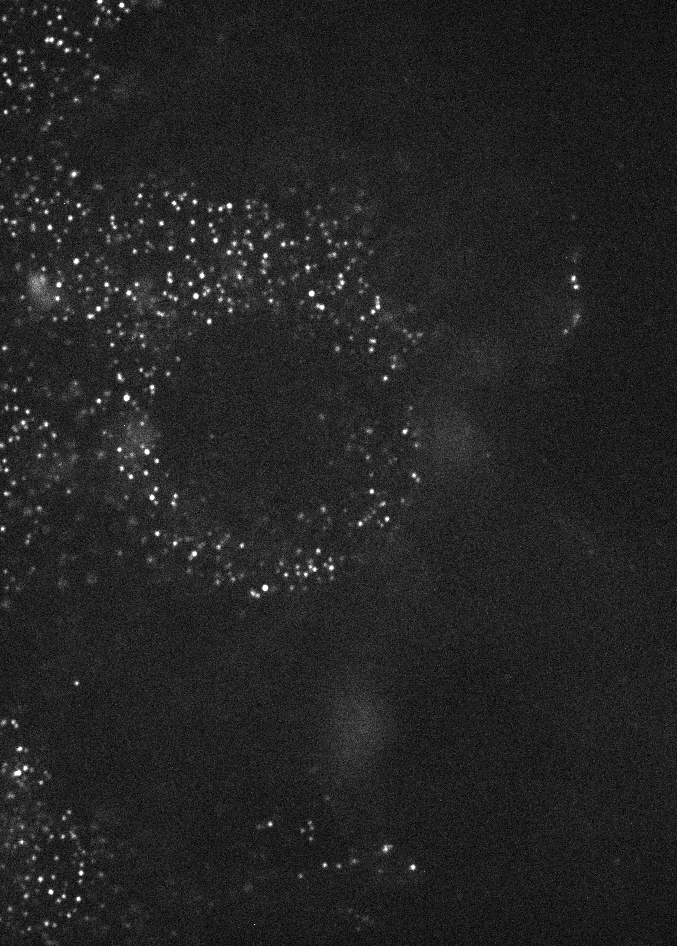

Supplement: Supplementary file 9 — Source data Fig. 3 [file 44318_2026_705_MOESM9_ESM.zip › Figure 3/F/20250821_MCF7VAPAGFP_NT_1_w2SPI 561 mCherry.TIF]

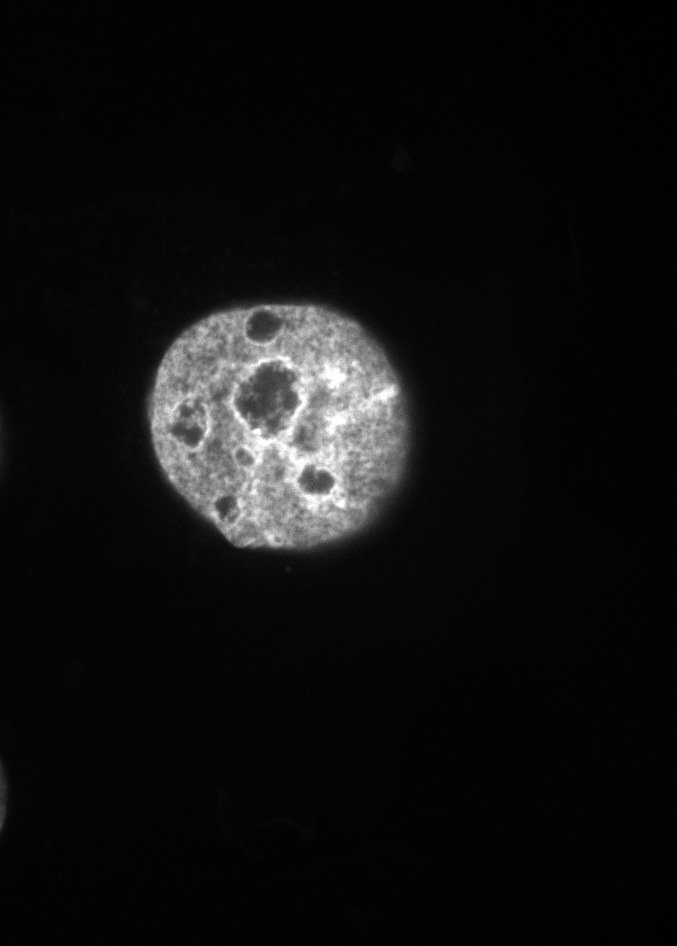

Supplement: Supplementary file 9 — Source data Fig. 3 [file 44318_2026_705_MOESM9_ESM.zip › Figure 3/F/20250821_MCF7VAPAGFP_NT_1_w3SPI 405 DAPI.TIF]

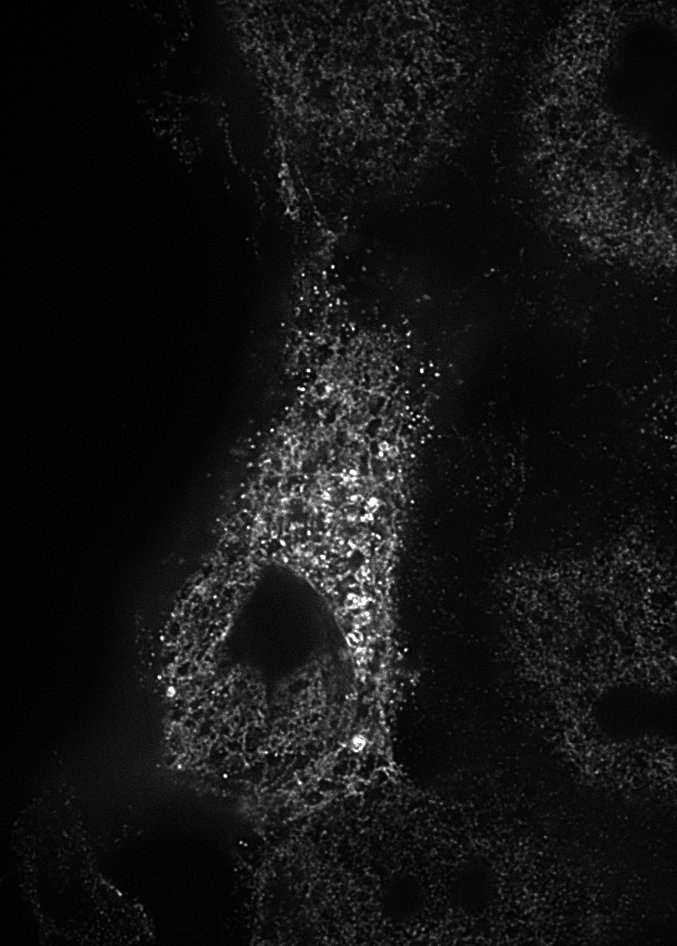

Supplement: Supplementary file 9 — Source data Fig. 3 [file 44318_2026_705_MOESM9_ESM.zip › Figure 3/G/20250823_MCF7VAPAGFPSTARD3_NT_3_SR_w1SPI 491 GFP.TIF]

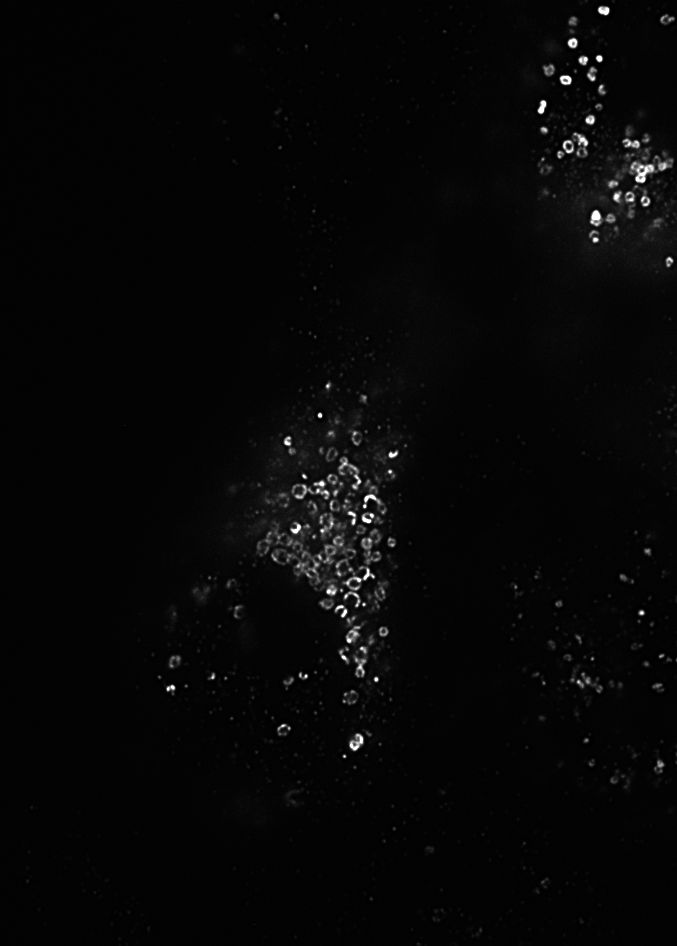

Supplement: Supplementary file 9 — Source data Fig. 3 [file 44318_2026_705_MOESM9_ESM.zip › Figure 3/G/20250823_MCF7VAPAGFPSTARD3_NT_3_SR_w2SPI 561 mCherry.TIF]

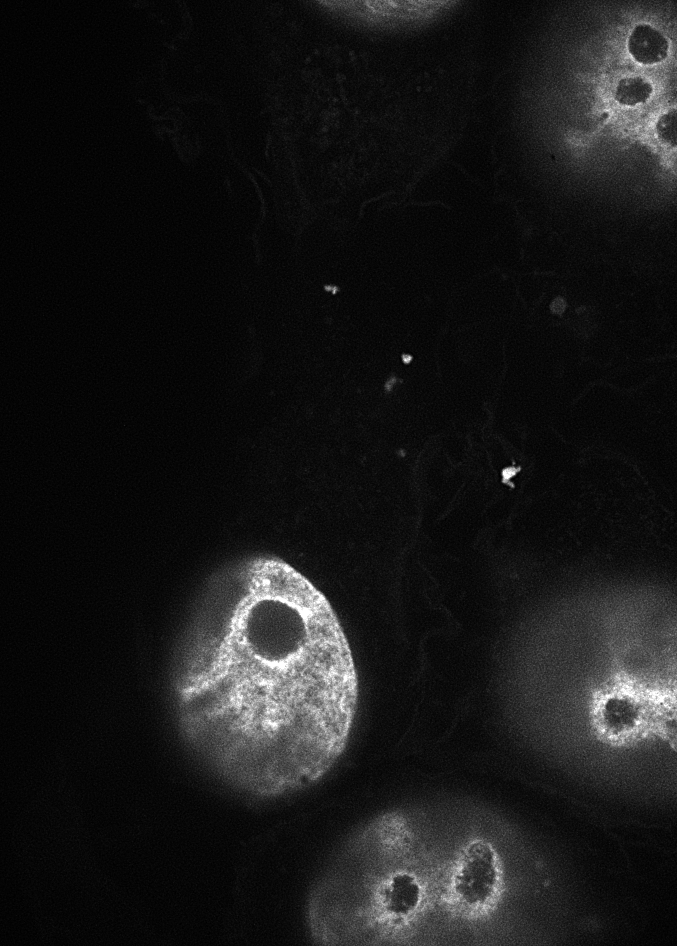

Supplement: Supplementary file 9 — Source data Fig. 3 [file 44318_2026_705_MOESM9_ESM.zip › Figure 3/G/20250823_MCF7VAPAGFPSTARD3_NT_3_SR_w3SPI 405 DAPI.TIF]

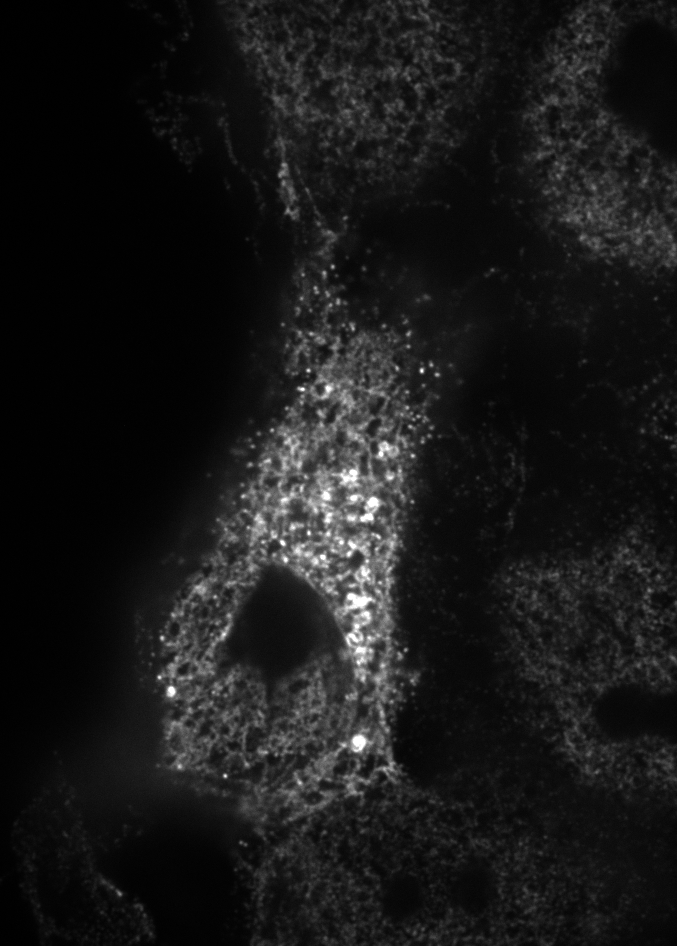

Supplement: Supplementary file 9 — Source data Fig. 3 [file 44318_2026_705_MOESM9_ESM.zip › Figure 3/G/20250823_MCF7VAPAGFPSTARD3_NT_3_w1SPI 491 GFP.TIF]

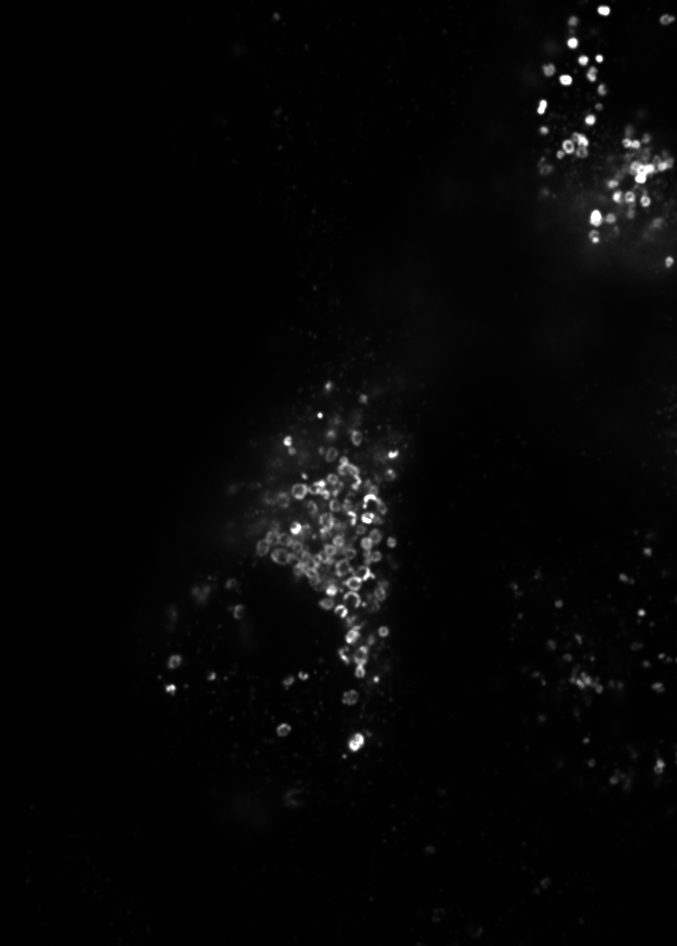

Supplement: Supplementary file 9 — Source data Fig. 3 [file 44318_2026_705_MOESM9_ESM.zip › Figure 3/G/20250823_MCF7VAPAGFPSTARD3_NT_3_w2SPI 561 mCherry.TIF]

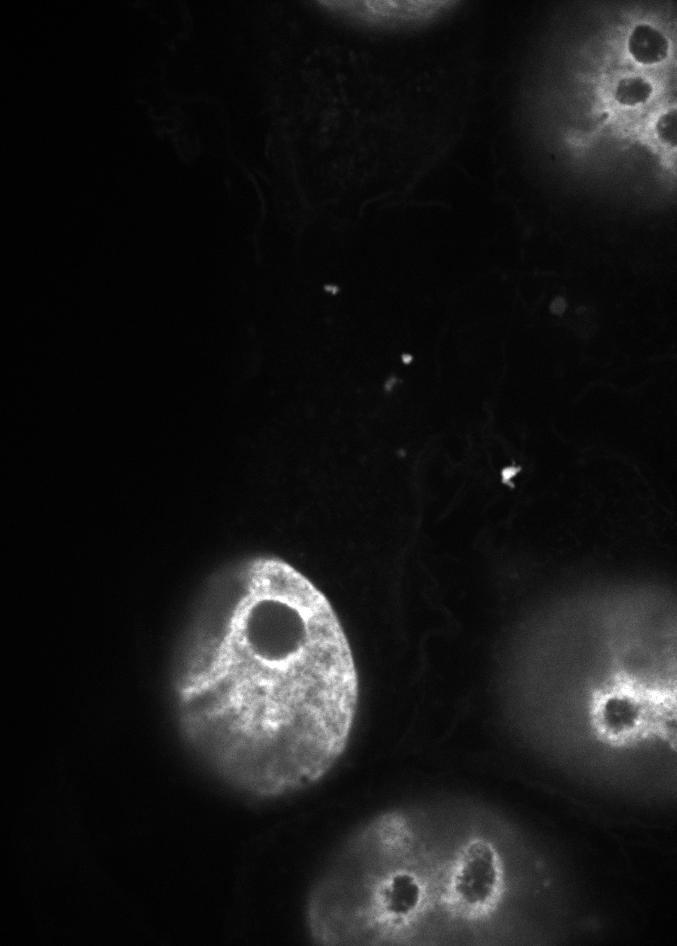

Supplement: Supplementary file 9 — Source data Fig. 3 [file 44318_2026_705_MOESM9_ESM.zip › Figure 3/G/20250823_MCF7VAPAGFPSTARD3_NT_3_w3SPI 405 DAPI.TIF]

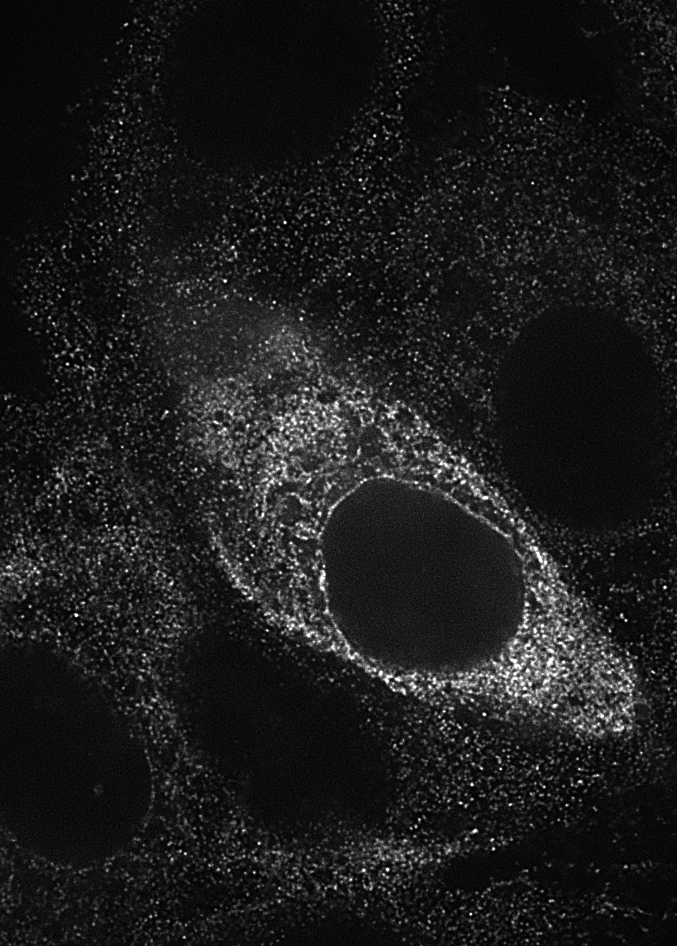

Supplement: Supplementary file 9 — Source data Fig. 3 [file 44318_2026_705_MOESM9_ESM.zip › Figure 3/H/20250821_MCF7GFPVAPASTARD3S209A_NT_6_SR_w1SPI 491 GFP.TIF]

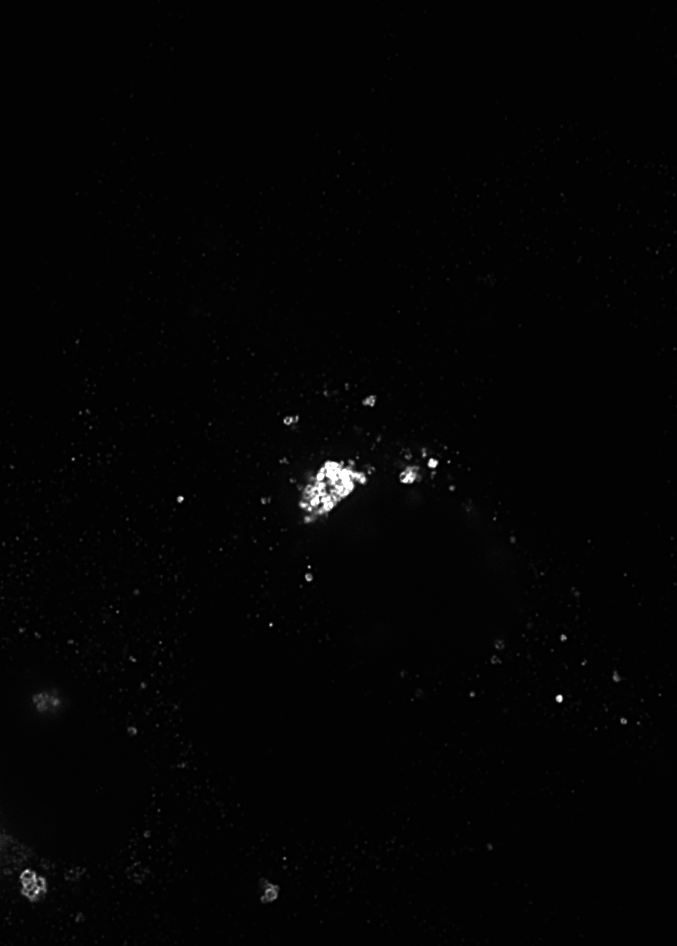

Supplement: Supplementary file 9 — Source data Fig. 3 [file 44318_2026_705_MOESM9_ESM.zip › Figure 3/H/20250821_MCF7GFPVAPASTARD3S209A_NT_6_SR_w2SPI 561 mCherry.TIF]

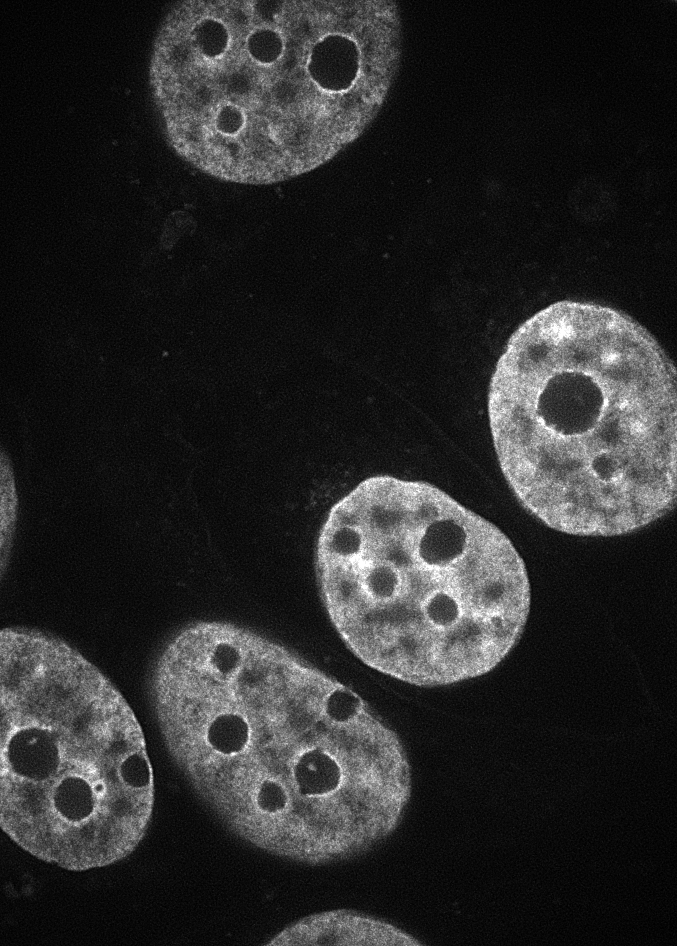

Supplement: Supplementary file 9 — Source data Fig. 3 [file 44318_2026_705_MOESM9_ESM.zip › Figure 3/H/20250821_MCF7GFPVAPASTARD3S209A_NT_6_SR_w3SPI 405 DAPI.TIF]

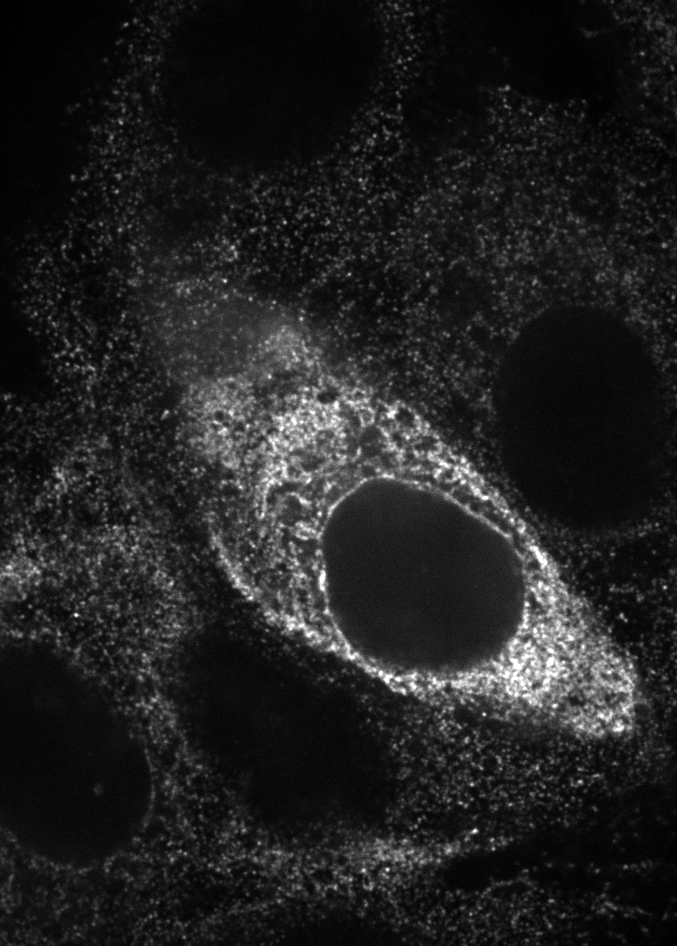

Supplement: Supplementary file 9 — Source data Fig. 3 [file 44318_2026_705_MOESM9_ESM.zip › Figure 3/H/20250821_MCF7GFPVAPASTARD3S209A_NT_6_w1SPI 491 GFP.TIF]

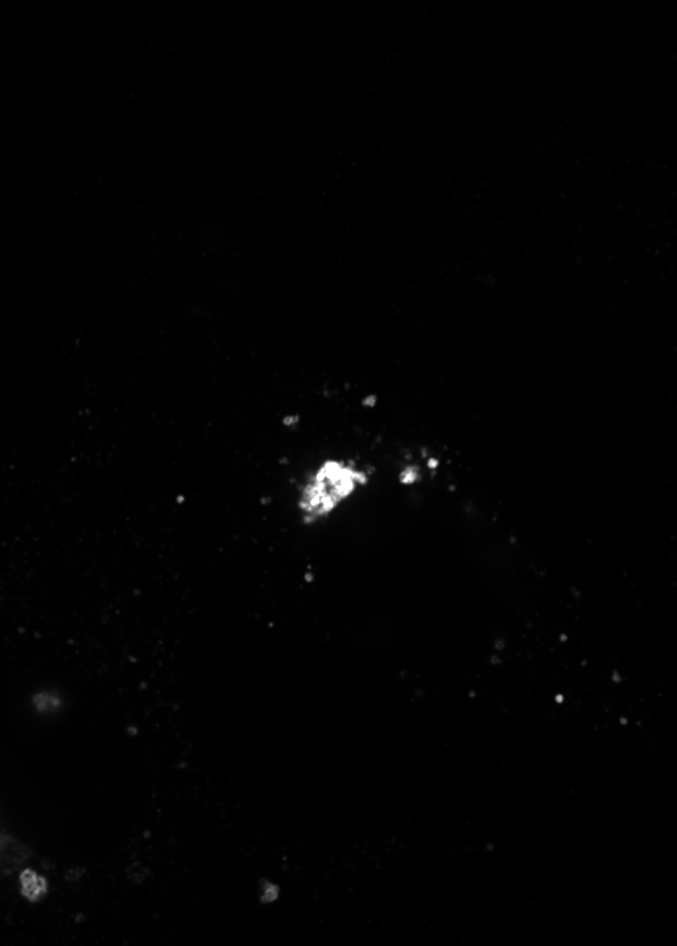

Supplement: Supplementary file 9 — Source data Fig. 3 [file 44318_2026_705_MOESM9_ESM.zip › Figure 3/H/20250821_MCF7GFPVAPASTARD3S209A_NT_6_w2SPI 561 mCherry.TIF]

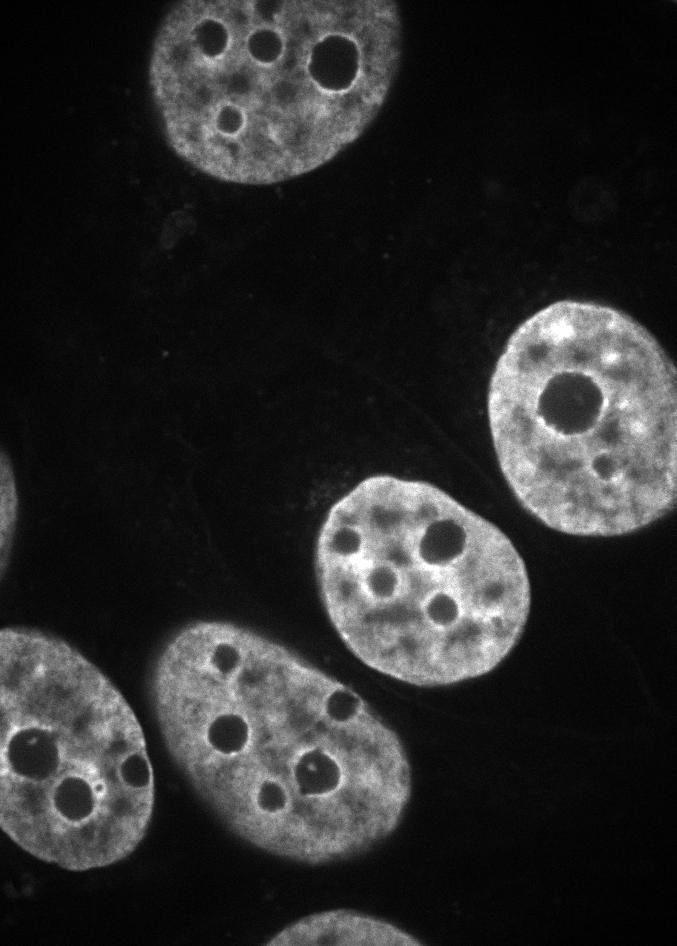

Supplement: Supplementary file 9 — Source data Fig. 3 [file 44318_2026_705_MOESM9_ESM.zip › Figure 3/H/20250821_MCF7GFPVAPASTARD3S209A_NT_6_w3SPI 405 DAPI.TIF]

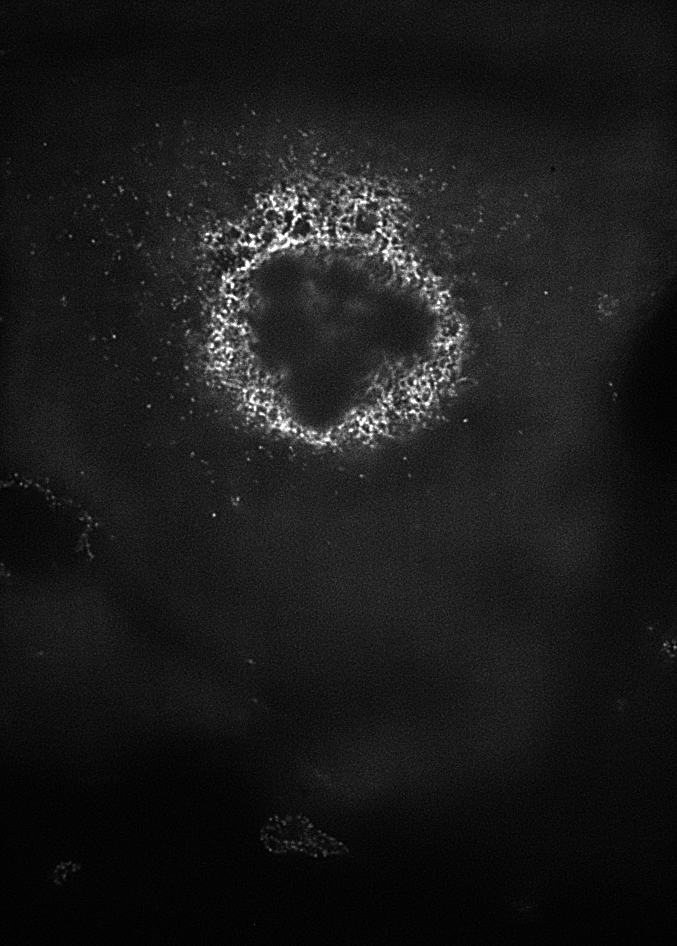

Supplement: Supplementary file 9 — Source data Fig. 3 [file 44318_2026_705_MOESM9_ESM.zip › Figure 3/I/20250823_MCF7GFPVAPAKDMDSTARD3_NT_2_SR_w1SPI 491 GFP.TIF]

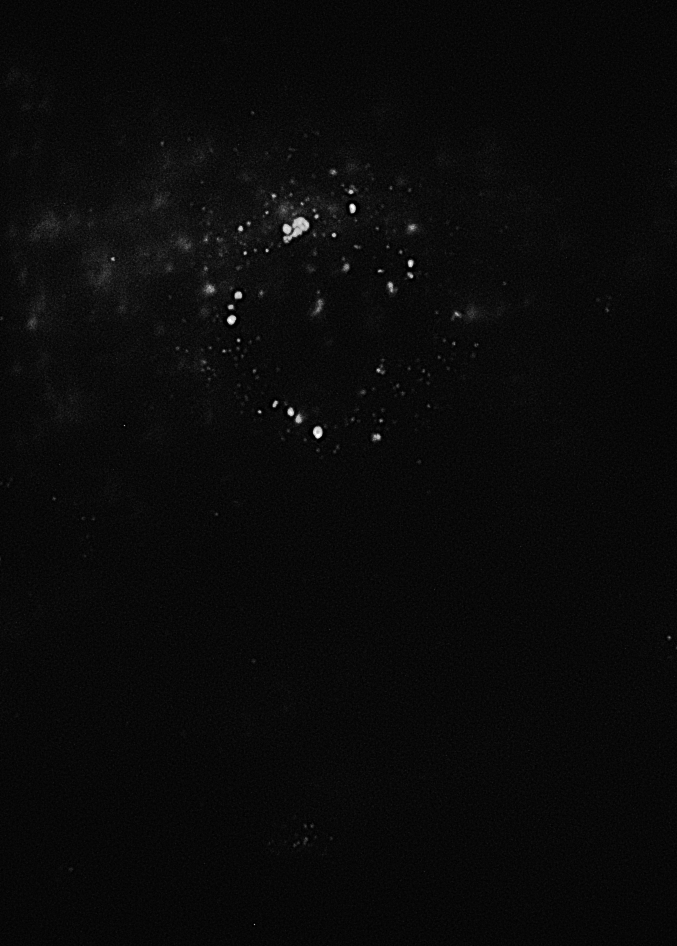

Supplement: Supplementary file 9 — Source data Fig. 3 [file 44318_2026_705_MOESM9_ESM.zip › Figure 3/I/20250823_MCF7GFPVAPAKDMDSTARD3_NT_2_SR_w2SPI 561 mCherry.TIF]

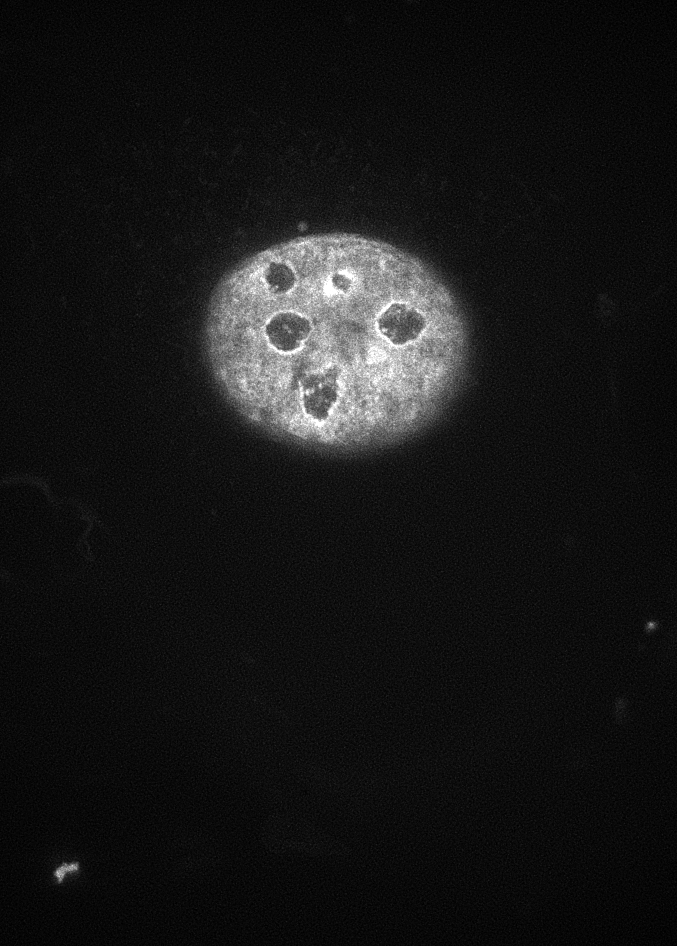

Supplement: Supplementary file 9 — Source data Fig. 3 [file 44318_2026_705_MOESM9_ESM.zip › Figure 3/I/20250823_MCF7GFPVAPAKDMDSTARD3_NT_2_SR_w3SPI 405 DAPI.TIF]

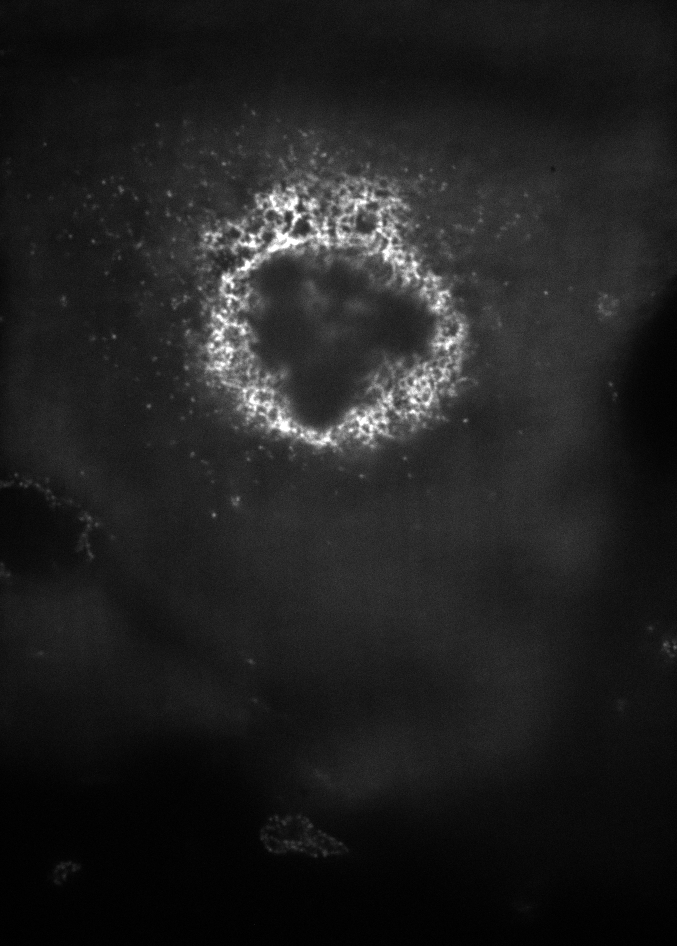

Supplement: Supplementary file 9 — Source data Fig. 3 [file 44318_2026_705_MOESM9_ESM.zip › Figure 3/I/20250823_MCF7GFPVAPAKDMDSTARD3_NT_2_w1SPI 491 GFP.TIF]

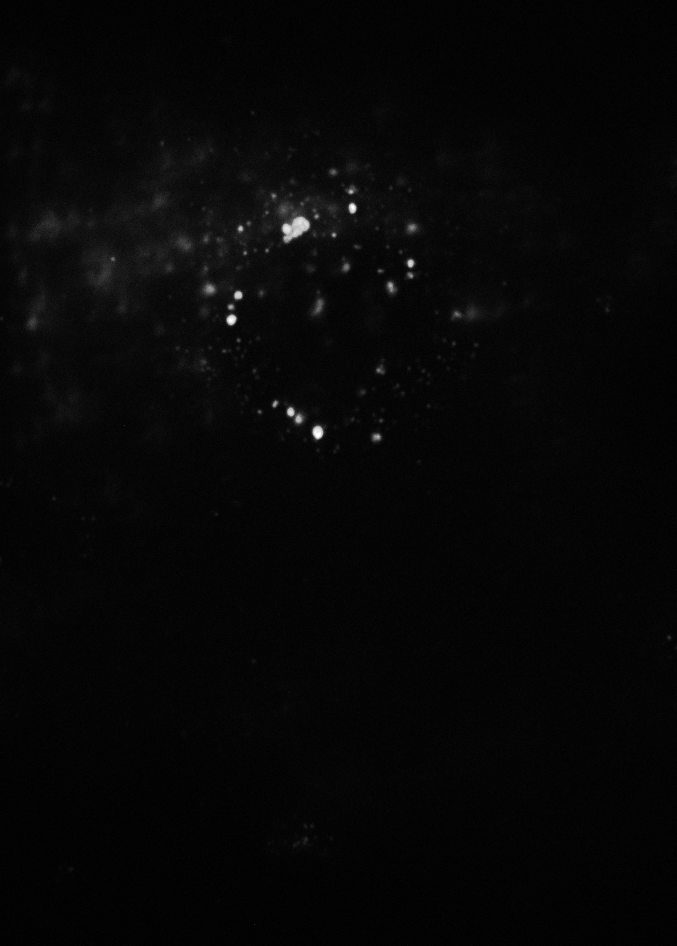

Supplement: Supplementary file 9 — Source data Fig. 3 [file 44318_2026_705_MOESM9_ESM.zip › Figure 3/I/20250823_MCF7GFPVAPAKDMDSTARD3_NT_2_w2SPI 561 mCherry.TIF]

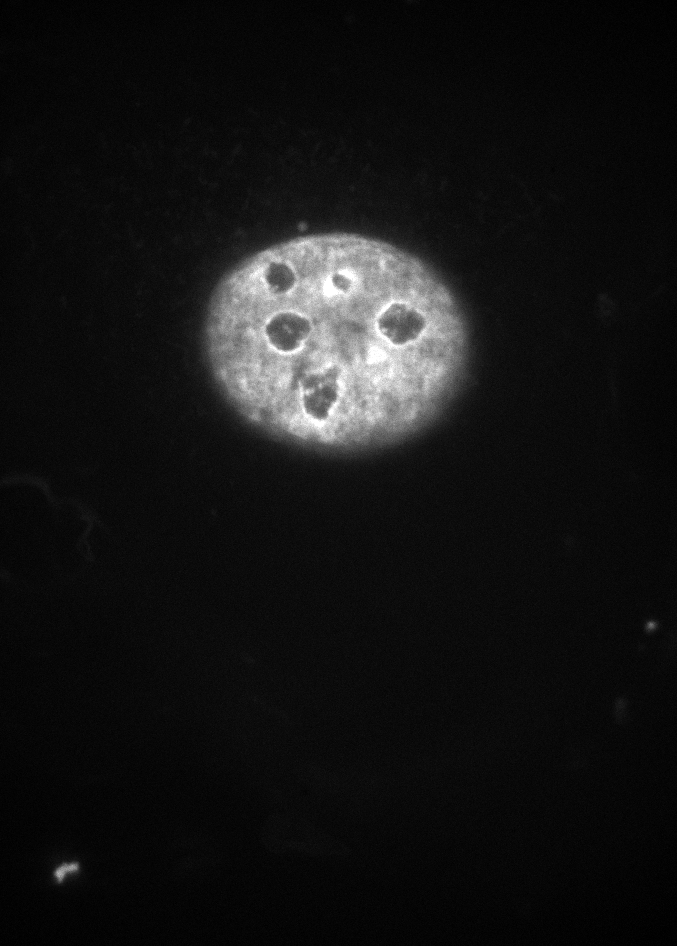

Supplement: Supplementary file 9 — Source data Fig. 3 [file 44318_2026_705_MOESM9_ESM.zip › Figure 3/I/20250823_MCF7GFPVAPAKDMDSTARD3_NT_2_w3SPI 405 DAPI.TIF]

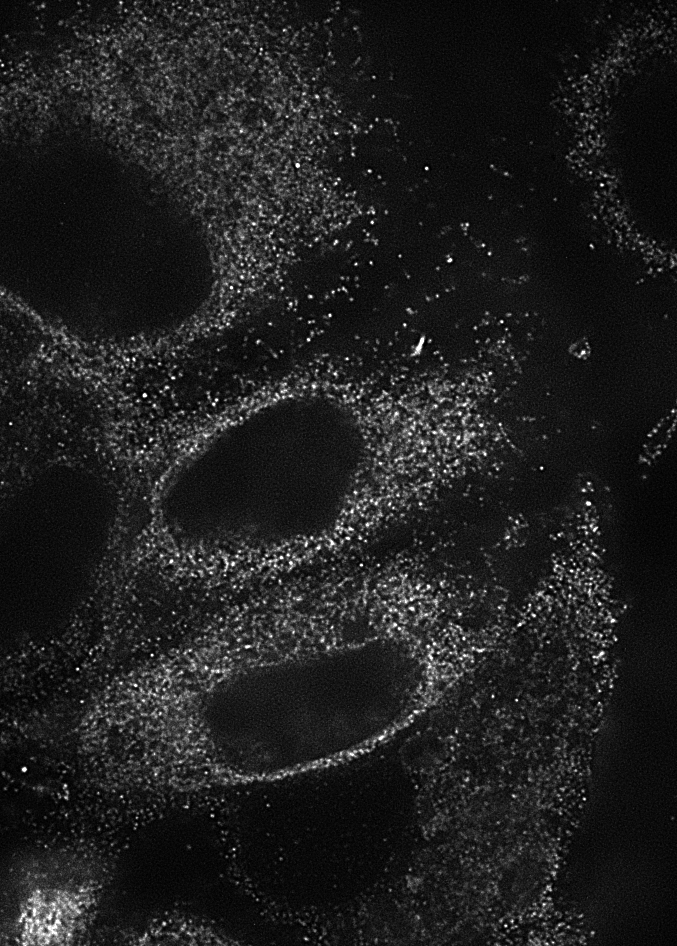

Supplement: Supplementary file 9 — Source data Fig. 3 [file 44318_2026_705_MOESM9_ESM.zip › Figure 3/J/20250821_MCF7VAPAGFPSTARD3_CHIR_3_SR_w1SPI 491 GFP.TIF]

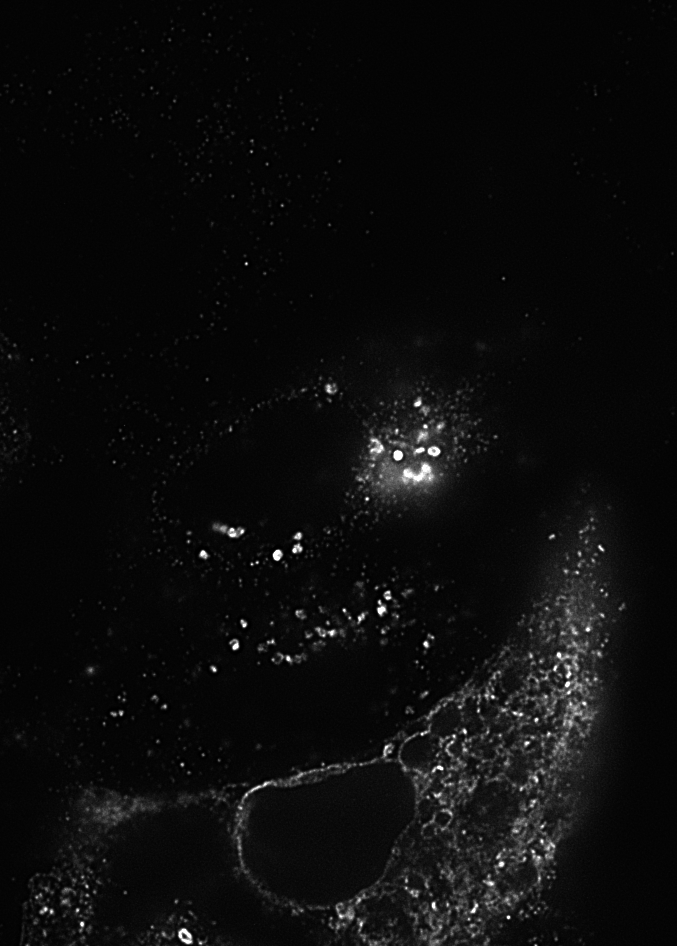

Supplement: Supplementary file 9 — Source data Fig. 3 [file 44318_2026_705_MOESM9_ESM.zip › Figure 3/J/20250821_MCF7VAPAGFPSTARD3_CHIR_3_SR_w2SPI 561 mCherry.TIF]

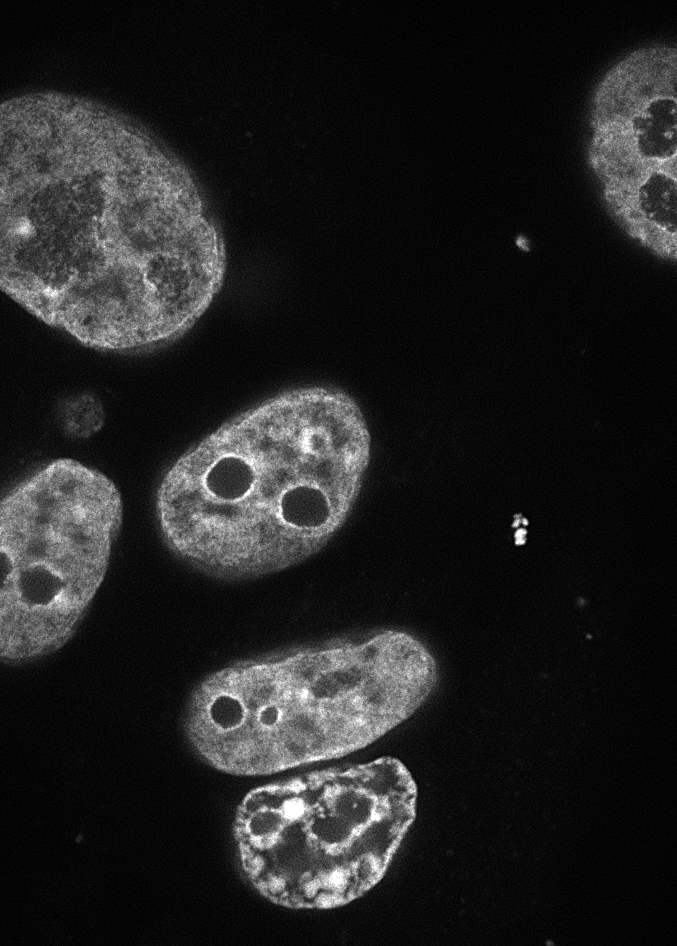

Supplement: Supplementary file 9 — Source data Fig. 3 [file 44318_2026_705_MOESM9_ESM.zip › Figure 3/J/20250821_MCF7VAPAGFPSTARD3_CHIR_3_SR_w3SPI 405 DAPI.TIF]

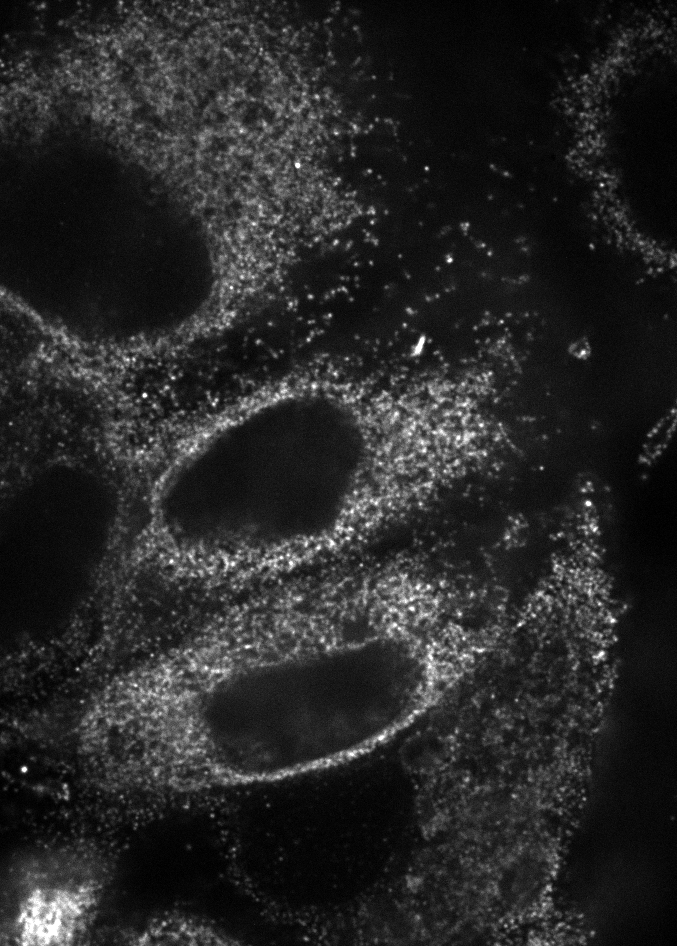

Supplement: Supplementary file 9 — Source data Fig. 3 [file 44318_2026_705_MOESM9_ESM.zip › Figure 3/J/20250821_MCF7VAPAGFPSTARD3_CHIR_3_w1SPI 491 GFP.TIF]

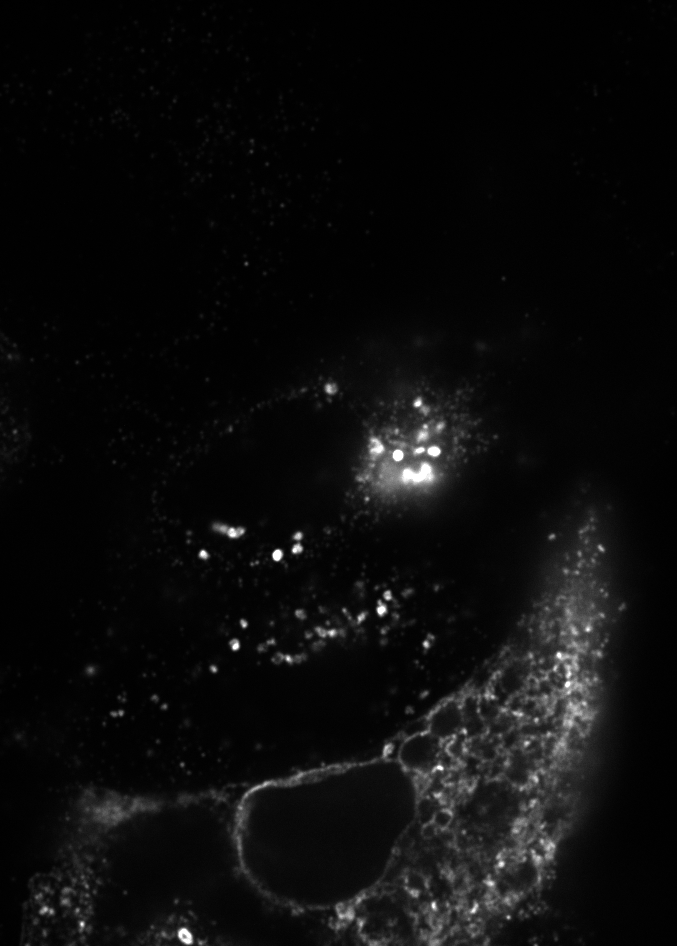

Supplement: Supplementary file 9 — Source data Fig. 3 [file 44318_2026_705_MOESM9_ESM.zip › Figure 3/J/20250821_MCF7VAPAGFPSTARD3_CHIR_3_w2SPI 561 mCherry.TIF]

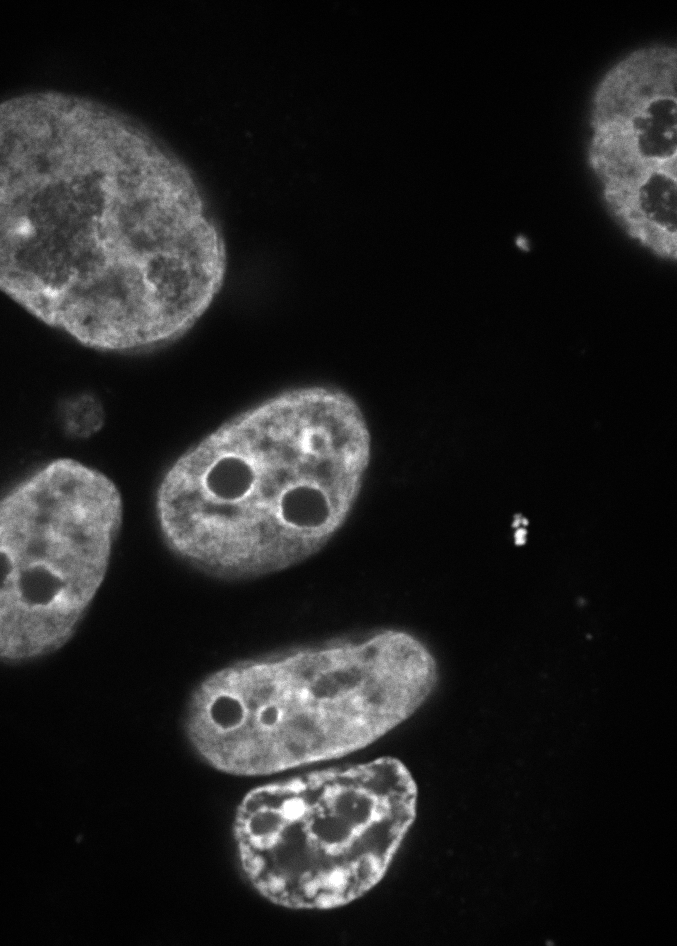

Supplement: Supplementary file 9 — Source data Fig. 3 [file 44318_2026_705_MOESM9_ESM.zip › Figure 3/J/20250821_MCF7VAPAGFPSTARD3_CHIR_3_w3SPI 405 DAPI.TIF]

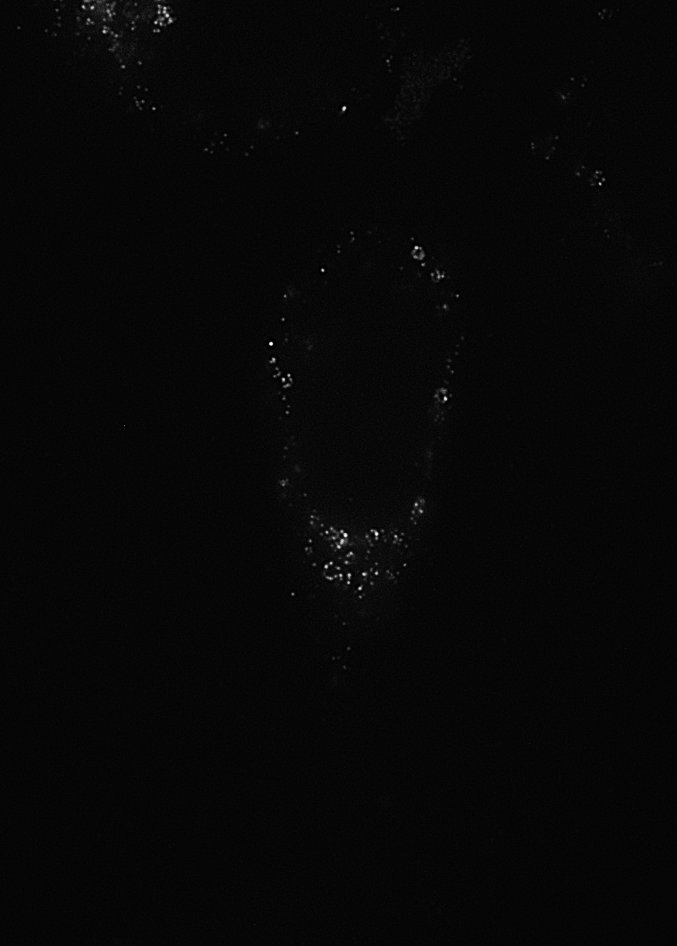

Supplement: Supplementary file 10 — Source data Fig. 4-1 [file 44318_2026_705_MOESM10_ESM.zip › Figure 4-1/A/HCC1954_CHIR99021/20240607_HCCSTARD3WT_CHIR_2_SR_w1SPI 491 GFP.TIF]

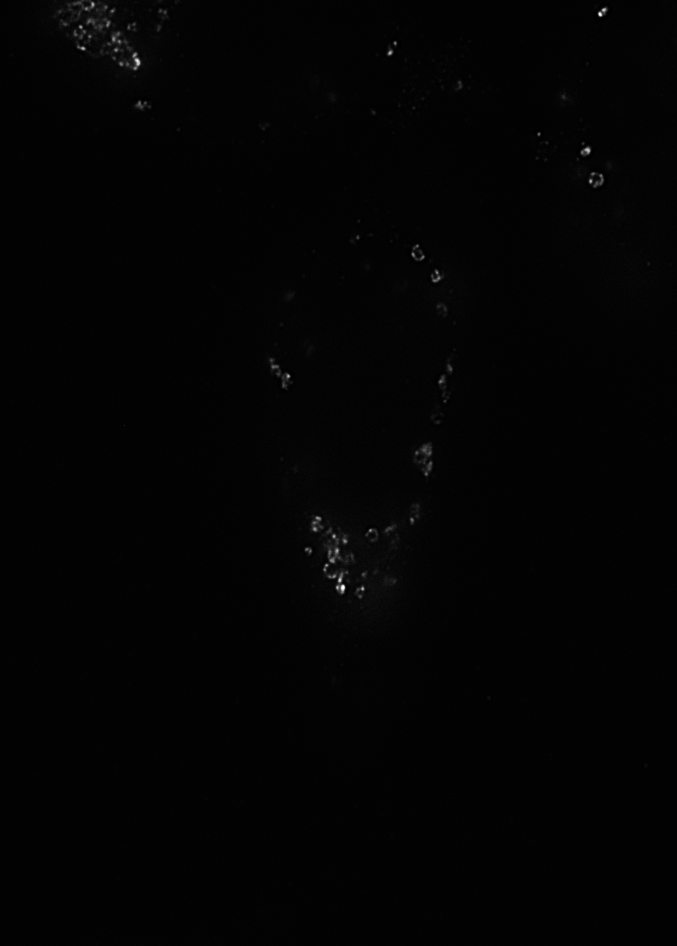

Supplement: Supplementary file 10 — Source data Fig. 4-1 [file 44318_2026_705_MOESM10_ESM.zip › Figure 4-1/A/HCC1954_CHIR99021/20240607_HCCSTARD3WT_CHIR_2_SR_w2SPI 561 mCherry.TIF]

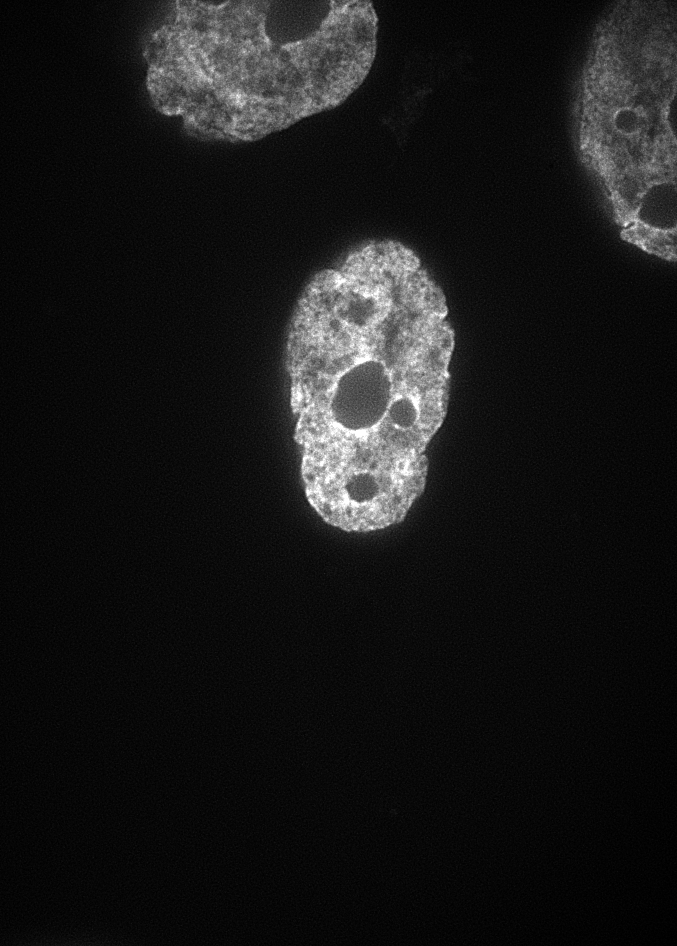

Supplement: Supplementary file 10 — Source data Fig. 4-1 [file 44318_2026_705_MOESM10_ESM.zip › Figure 4-1/A/HCC1954_CHIR99021/20240607_HCCSTARD3WT_CHIR_2_SR_w3SPI 405 DAPI.TIF]

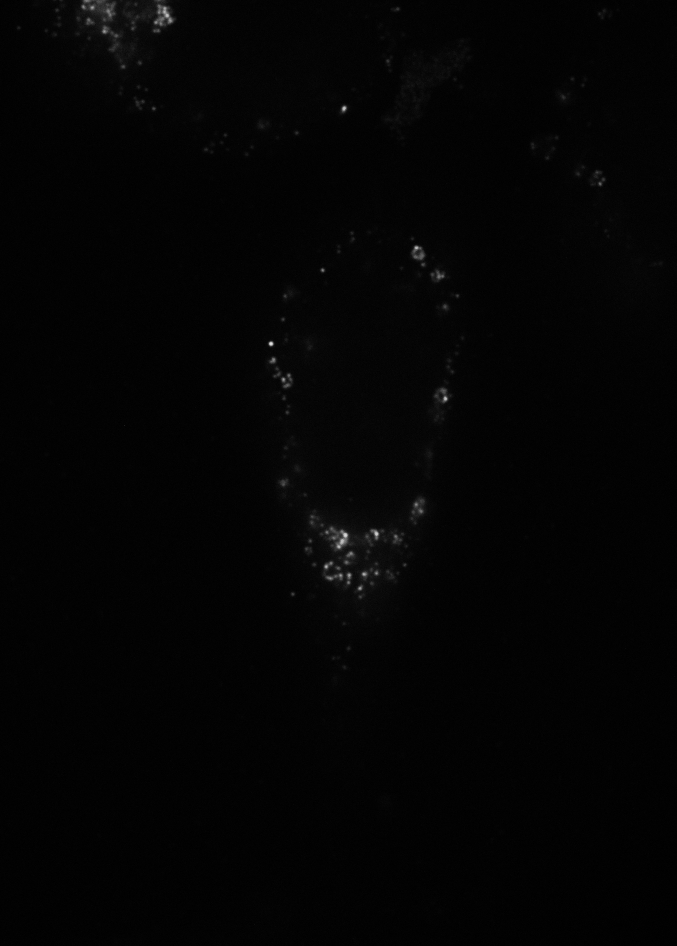

Supplement: Supplementary file 10 — Source data Fig. 4-1 [file 44318_2026_705_MOESM10_ESM.zip › Figure 4-1/A/HCC1954_CHIR99021/20240607_HCCSTARD3WT_CHIR_2_w1SPI 491 GFP.TIF]

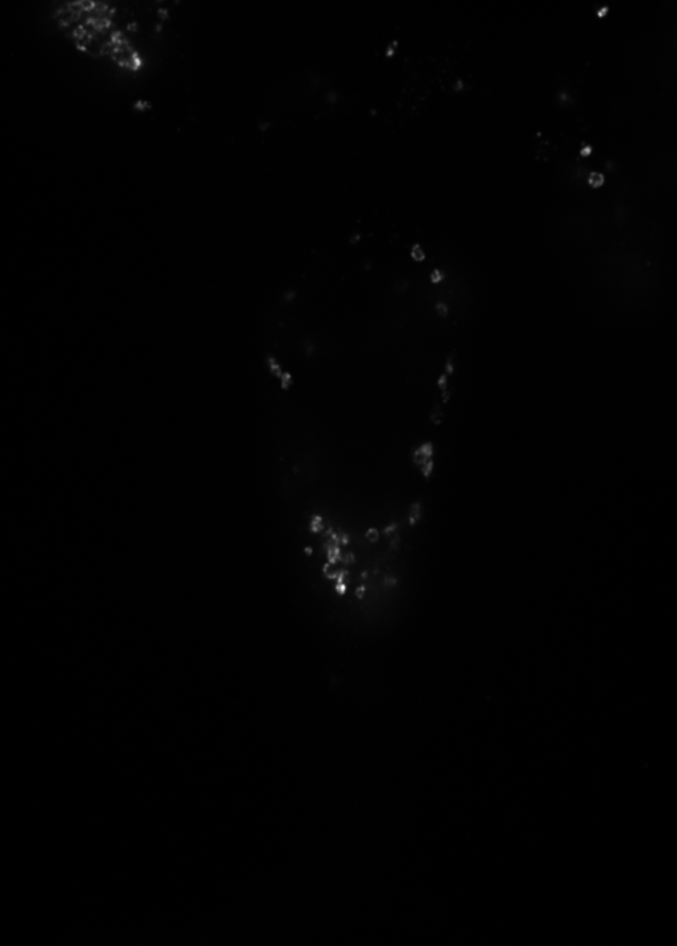

Supplement: Supplementary file 10 — Source data Fig. 4-1 [file 44318_2026_705_MOESM10_ESM.zip › Figure 4-1/A/HCC1954_CHIR99021/20240607_HCCSTARD3WT_CHIR_2_w2SPI 561 mCherry.TIF]

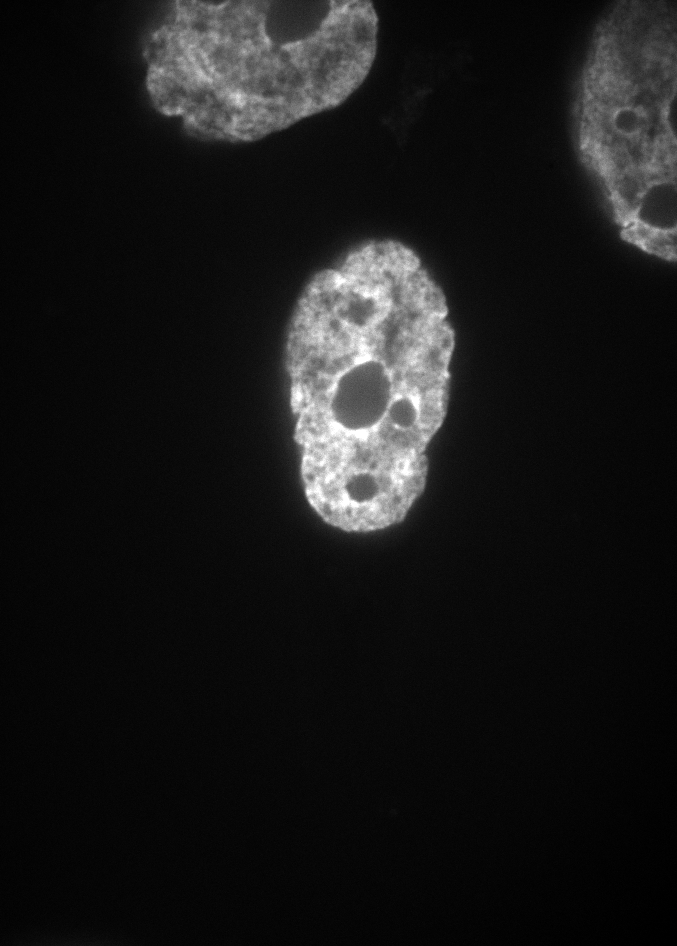

Supplement: Supplementary file 10 — Source data Fig. 4-1 [file 44318_2026_705_MOESM10_ESM.zip › Figure 4-1/A/HCC1954_CHIR99021/20240607_HCCSTARD3WT_CHIR_2_w3SPI 405 DAPI.TIF]

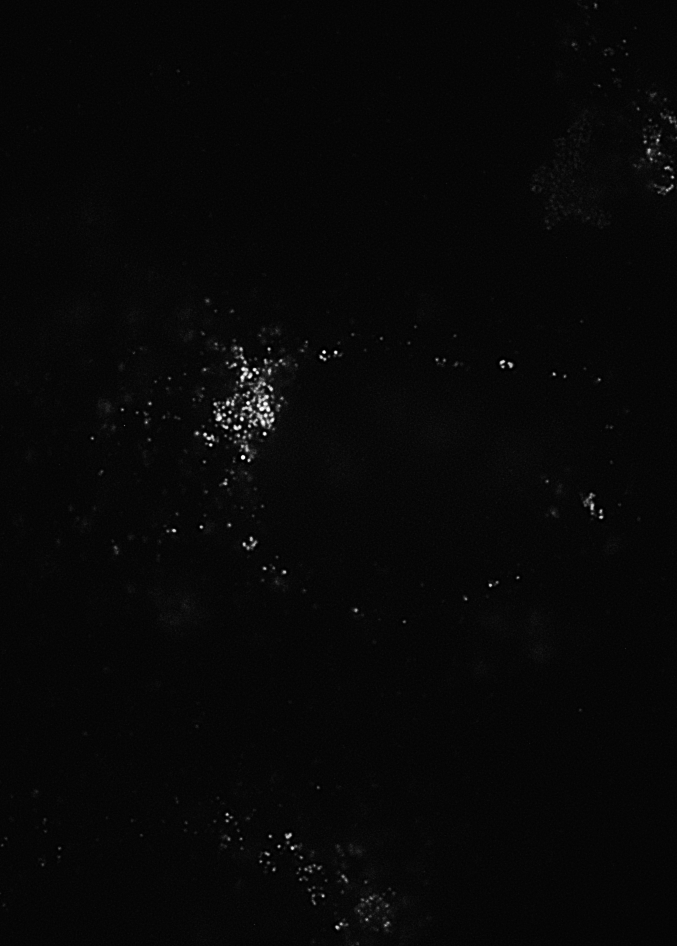

Supplement: Supplementary file 10 — Source data Fig. 4-1 [file 44318_2026_705_MOESM10_ESM.zip › Figure 4-1/A/HCC1954_NT/20240607_HCCSTARD3WT_NT_2_SR_w1SPI 491 GFP.TIF]

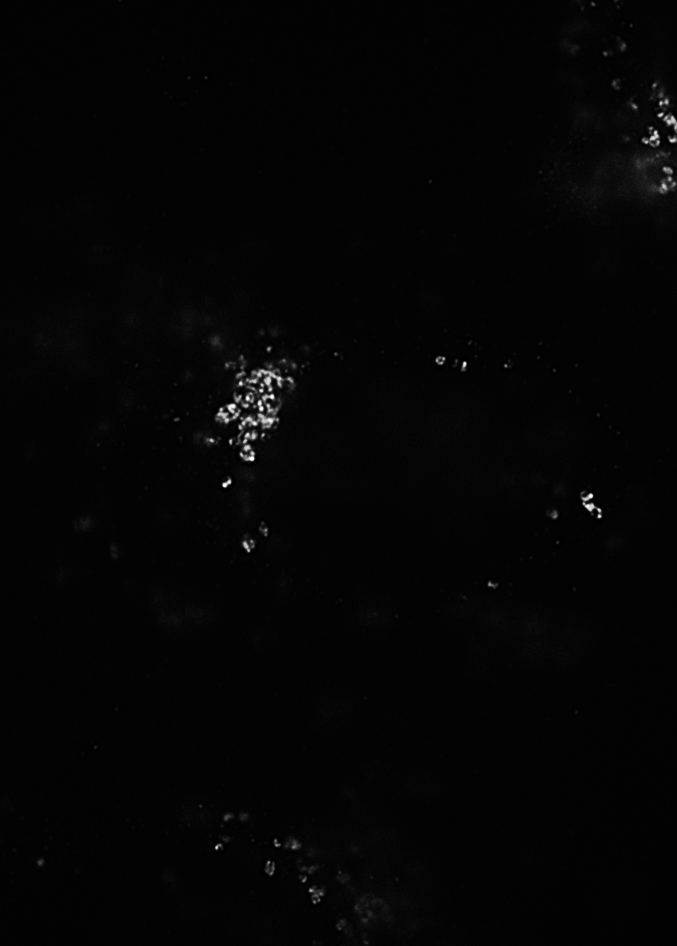

Supplement: Supplementary file 10 — Source data Fig. 4-1 [file 44318_2026_705_MOESM10_ESM.zip › Figure 4-1/A/HCC1954_NT/20240607_HCCSTARD3WT_NT_2_SR_w2SPI 561 mCherry.TIF]

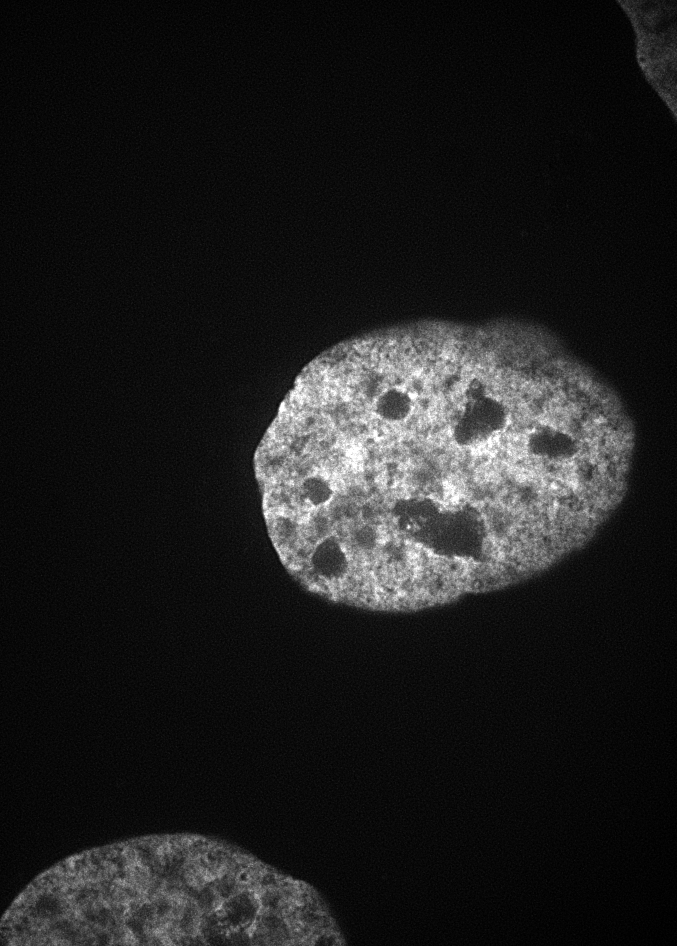

Supplement: Supplementary file 10 — Source data Fig. 4-1 [file 44318_2026_705_MOESM10_ESM.zip › Figure 4-1/A/HCC1954_NT/20240607_HCCSTARD3WT_NT_2_SR_w3SPI 405 DAPI.TIF]

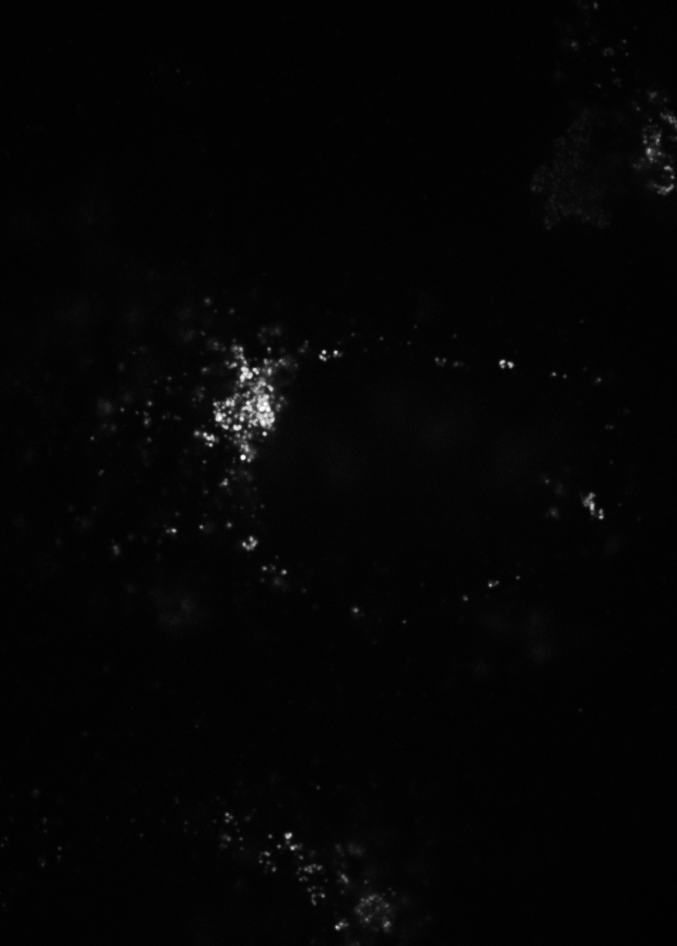

Supplement: Supplementary file 10 — Source data Fig. 4-1 [file 44318_2026_705_MOESM10_ESM.zip › Figure 4-1/A/HCC1954_NT/20240607_HCCSTARD3WT_NT_2_w1SPI 491 GFP.TIF]

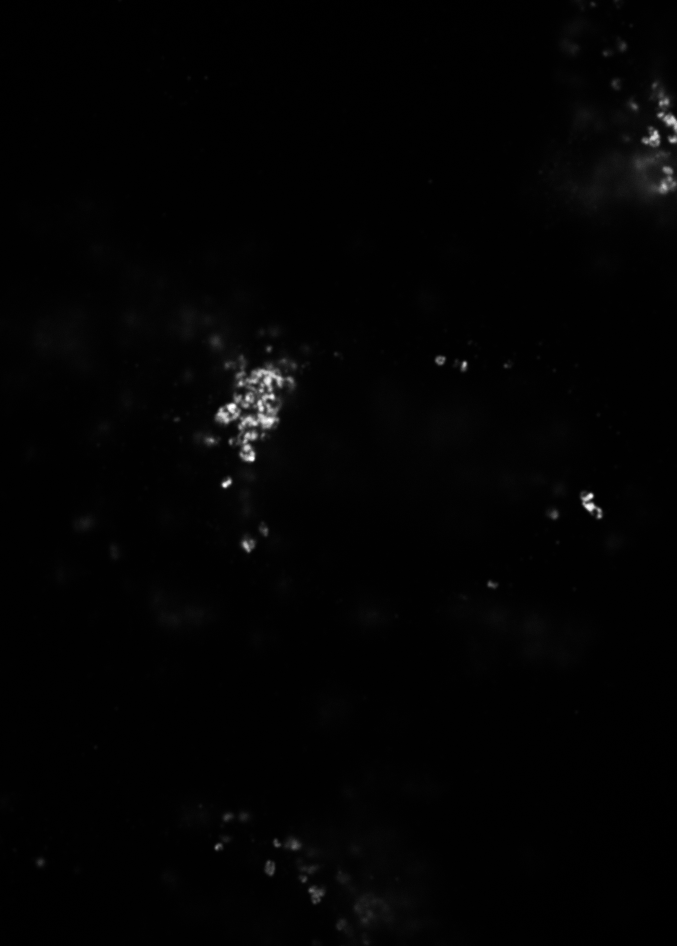

Supplement: Supplementary file 10 — Source data Fig. 4-1 [file 44318_2026_705_MOESM10_ESM.zip › Figure 4-1/A/HCC1954_NT/20240607_HCCSTARD3WT_NT_2_w2SPI 561 mCherry.TIF]

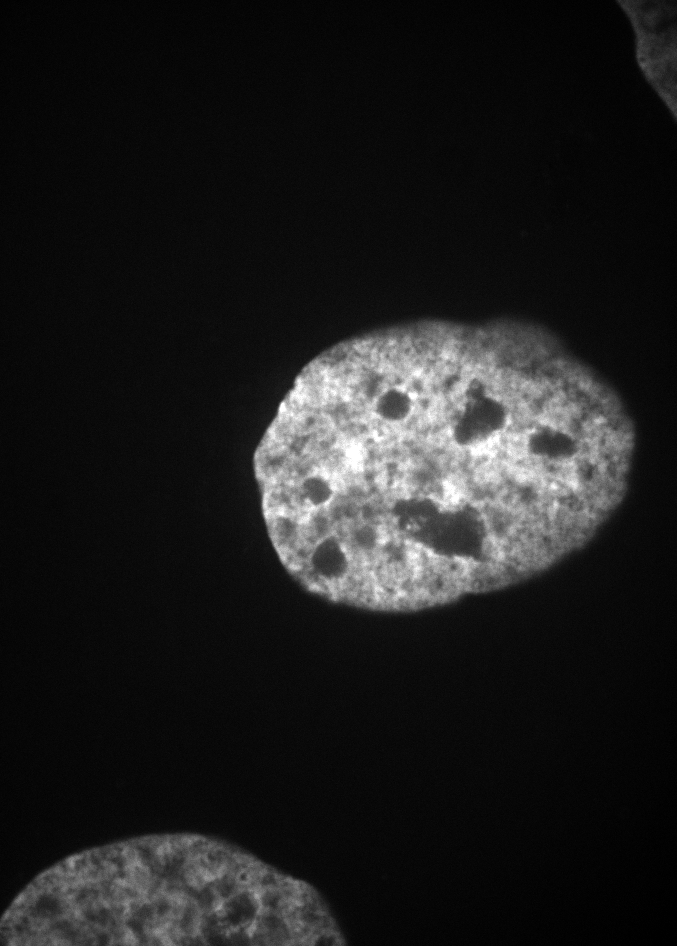

Supplement: Supplementary file 10 — Source data Fig. 4-1 [file 44318_2026_705_MOESM10_ESM.zip › Figure 4-1/A/HCC1954_NT/20240607_HCCSTARD3WT_NT_2_w3SPI 405 DAPI.TIF]

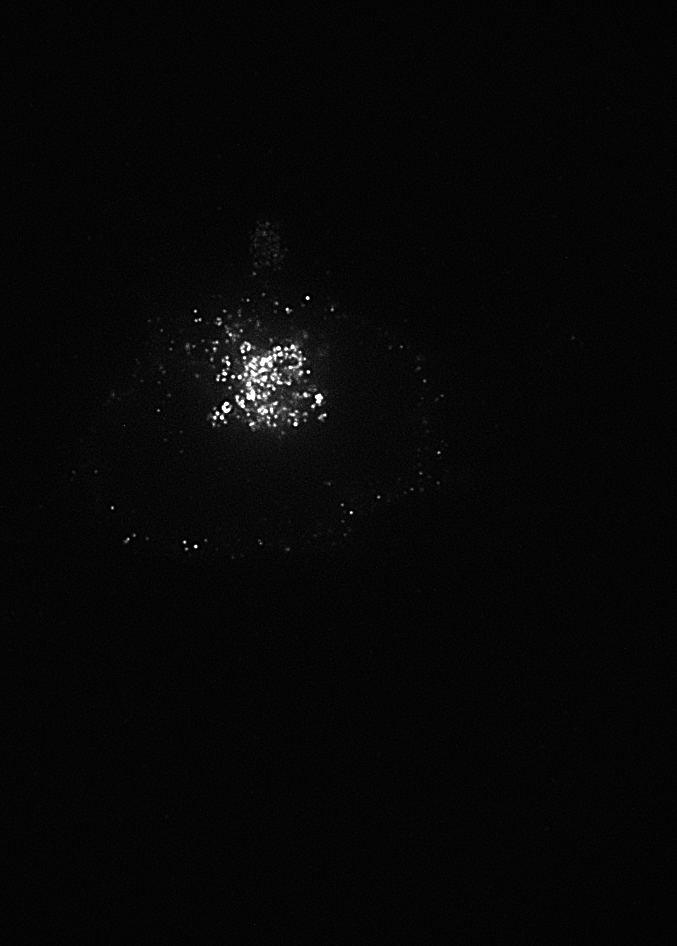

Supplement: Supplementary file 10 — Source data Fig. 4-1 [file 44318_2026_705_MOESM10_ESM.zip › Figure 4-1/B/HCC1954_siCT_CHIR99021/20240603_HCCSTARD3sictrl_CHIR_5_SR_w1SPI 491 GFP.TIF]

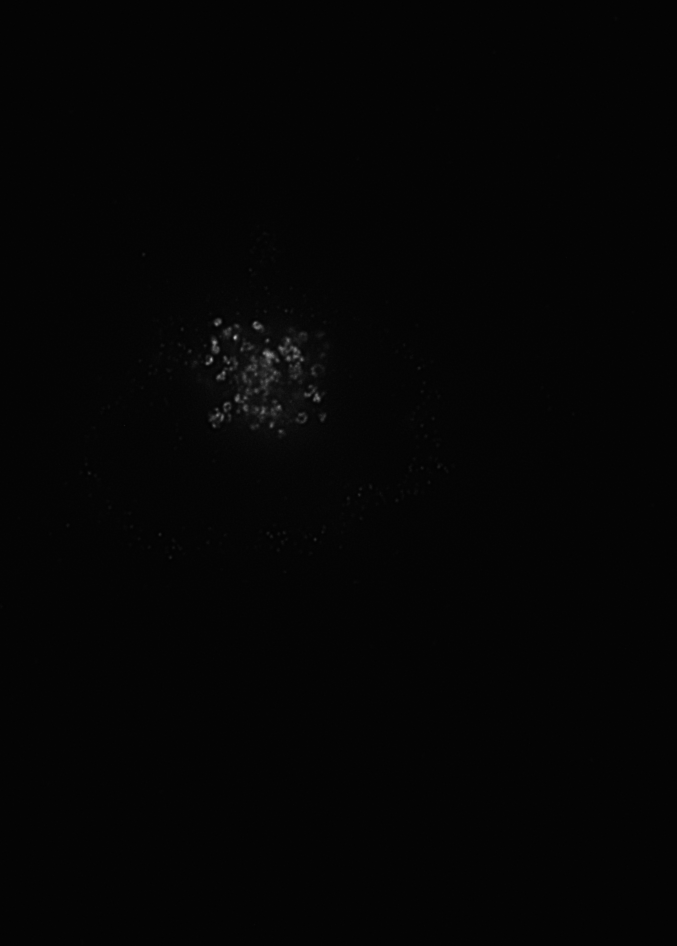

Supplement: Supplementary file 10 — Source data Fig. 4-1 [file 44318_2026_705_MOESM10_ESM.zip › Figure 4-1/B/HCC1954_siCT_CHIR99021/20240603_HCCSTARD3sictrl_CHIR_5_SR_w2SPI 561 mCherry.TIF]

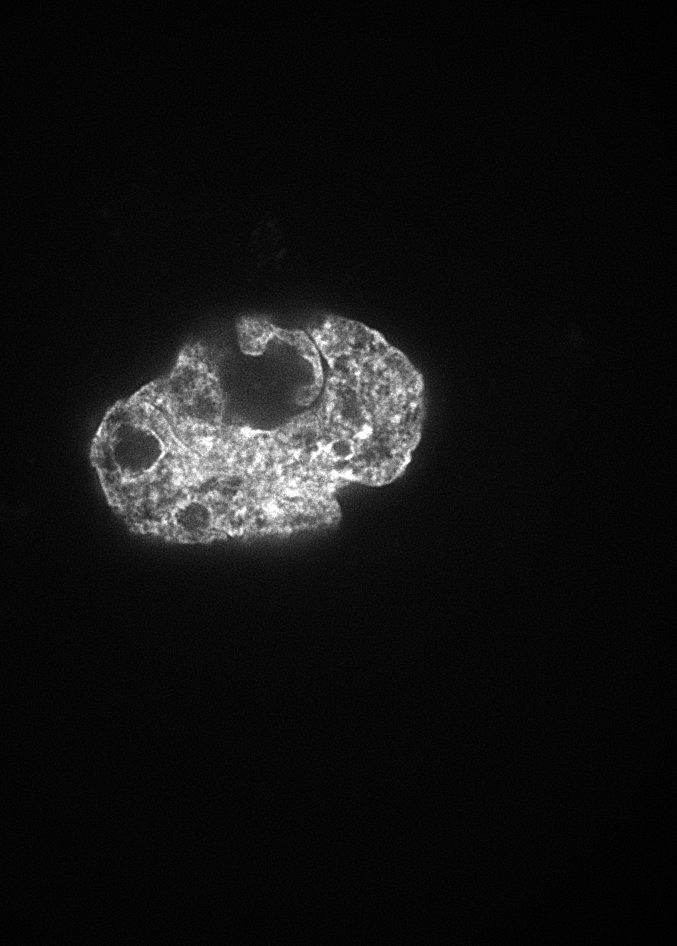

Supplement: Supplementary file 10 — Source data Fig. 4-1 [file 44318_2026_705_MOESM10_ESM.zip › Figure 4-1/B/HCC1954_siCT_CHIR99021/20240603_HCCSTARD3sictrl_CHIR_5_SR_w3SPI 405 DAPI.TIF]

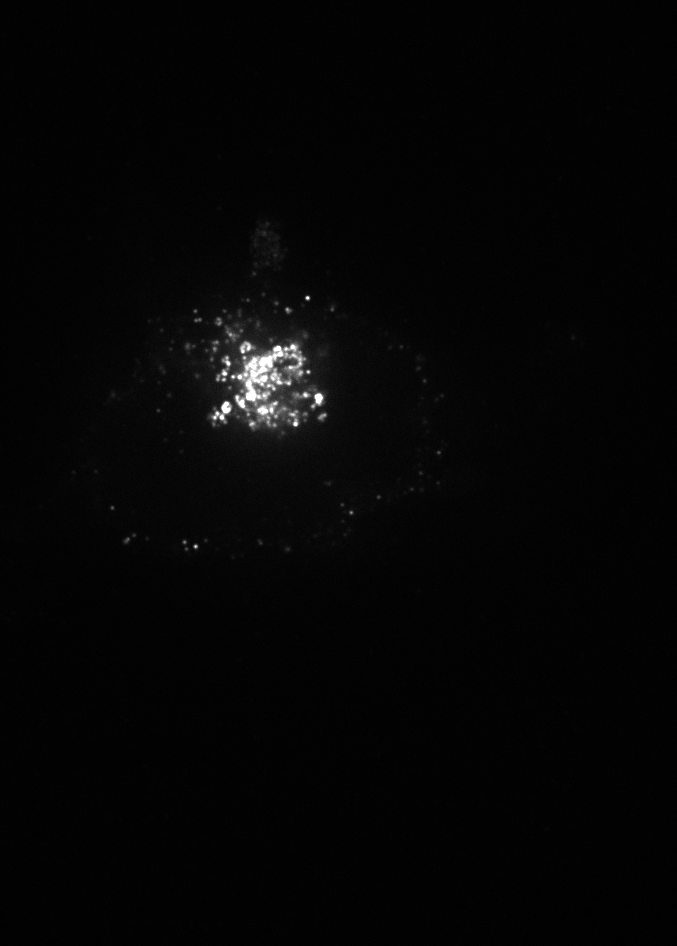

Supplement: Supplementary file 10 — Source data Fig. 4-1 [file 44318_2026_705_MOESM10_ESM.zip › Figure 4-1/B/HCC1954_siCT_CHIR99021/20240603_HCCSTARD3sictrl_CHIR_5_w1SPI 491 GFP.TIF]

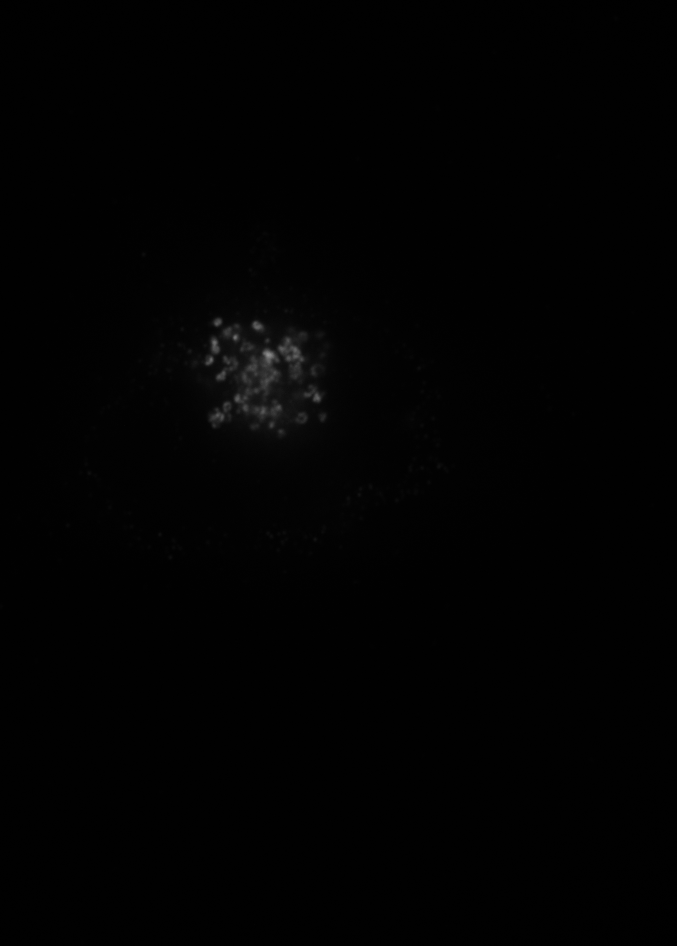

Supplement: Supplementary file 10 — Source data Fig. 4-1 [file 44318_2026_705_MOESM10_ESM.zip › Figure 4-1/B/HCC1954_siCT_CHIR99021/20240603_HCCSTARD3sictrl_CHIR_5_w2SPI 561 mCherry.TIF]

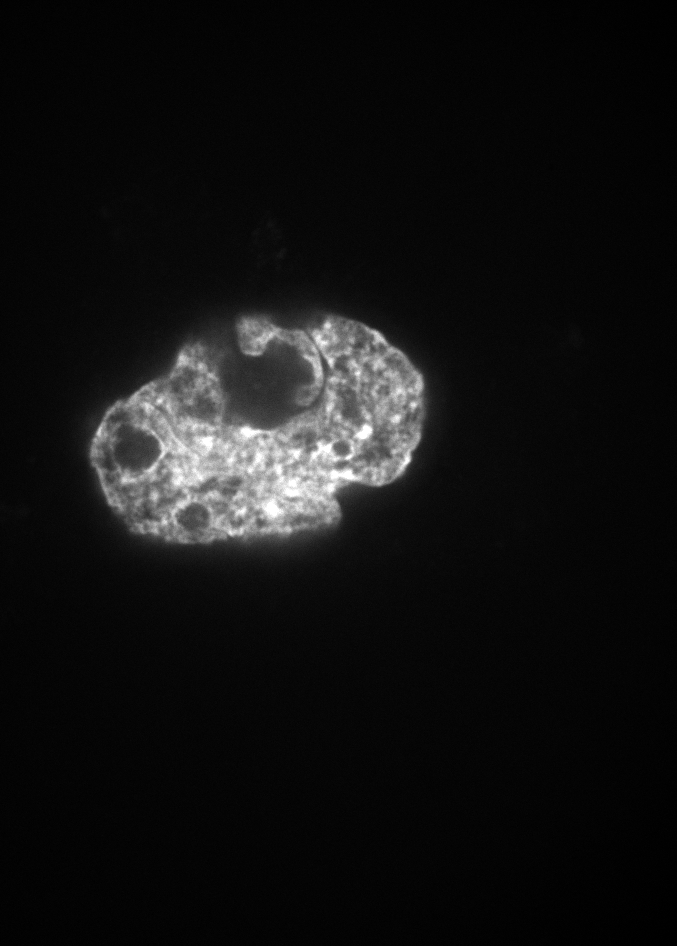

Supplement: Supplementary file 10 — Source data Fig. 4-1 [file 44318_2026_705_MOESM10_ESM.zip › Figure 4-1/B/HCC1954_siCT_CHIR99021/20240603_HCCSTARD3sictrl_CHIR_5_w3SPI 405 DAPI.TIF]

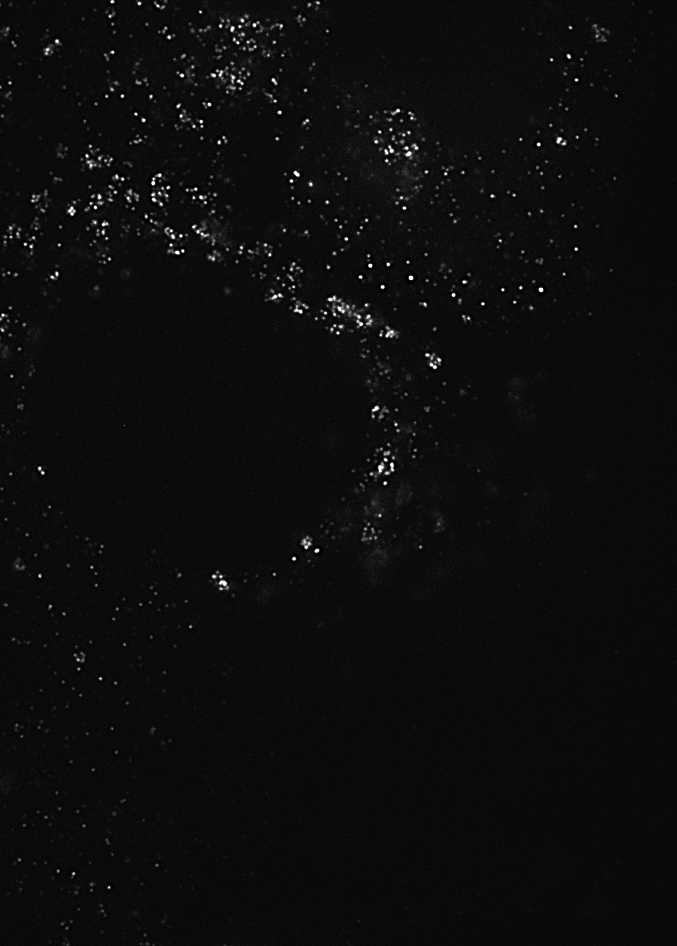

Supplement: Supplementary file 10 — Source data Fig. 4-1 [file 44318_2026_705_MOESM10_ESM.zip › Figure 4-1/B/HCC1954_siCT_NT/20240603_HCCSTARD3sictrl_NT_1_SR_w1SPI 491 GFP.TIF]

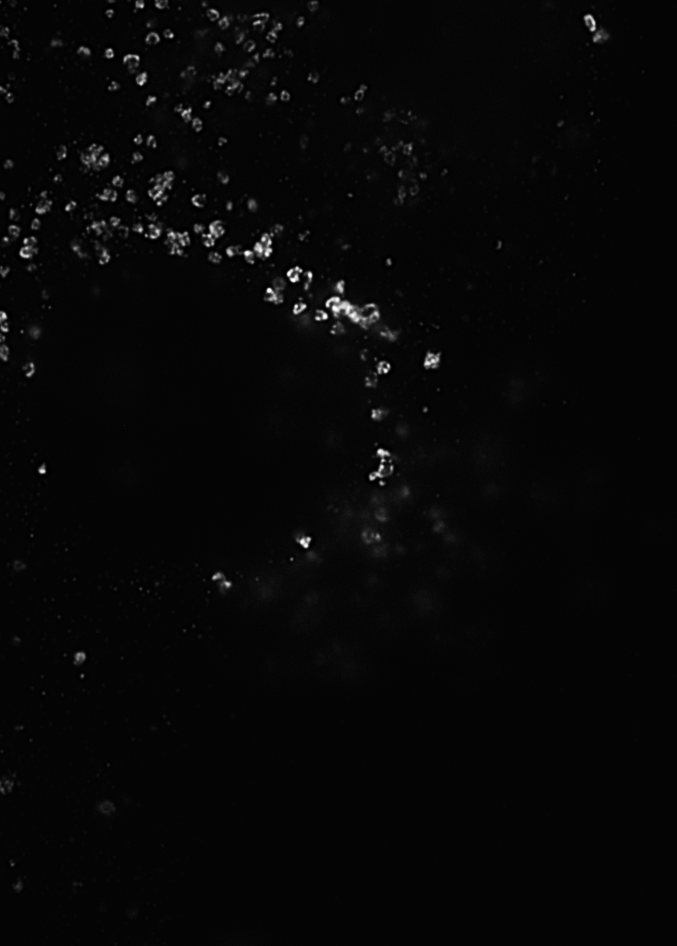

Supplement: Supplementary file 10 — Source data Fig. 4-1 [file 44318_2026_705_MOESM10_ESM.zip › Figure 4-1/B/HCC1954_siCT_NT/20240603_HCCSTARD3sictrl_NT_1_SR_w2SPI 561 mCherry.TIF]

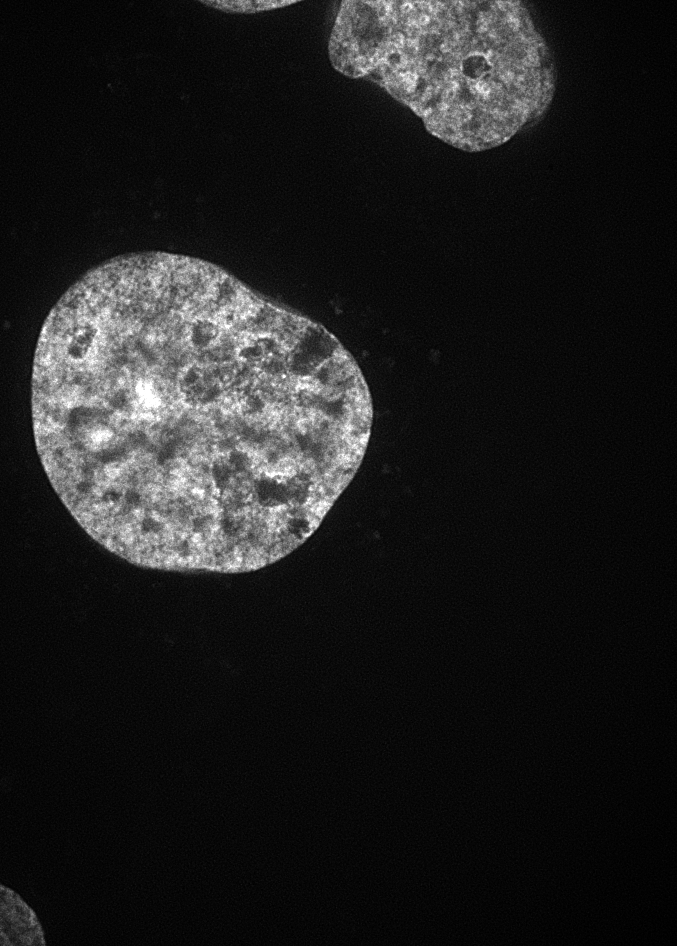

Supplement: Supplementary file 10 — Source data Fig. 4-1 [file 44318_2026_705_MOESM10_ESM.zip › Figure 4-1/B/HCC1954_siCT_NT/20240603_HCCSTARD3sictrl_NT_1_SR_w3SPI 405 DAPI.TIF]

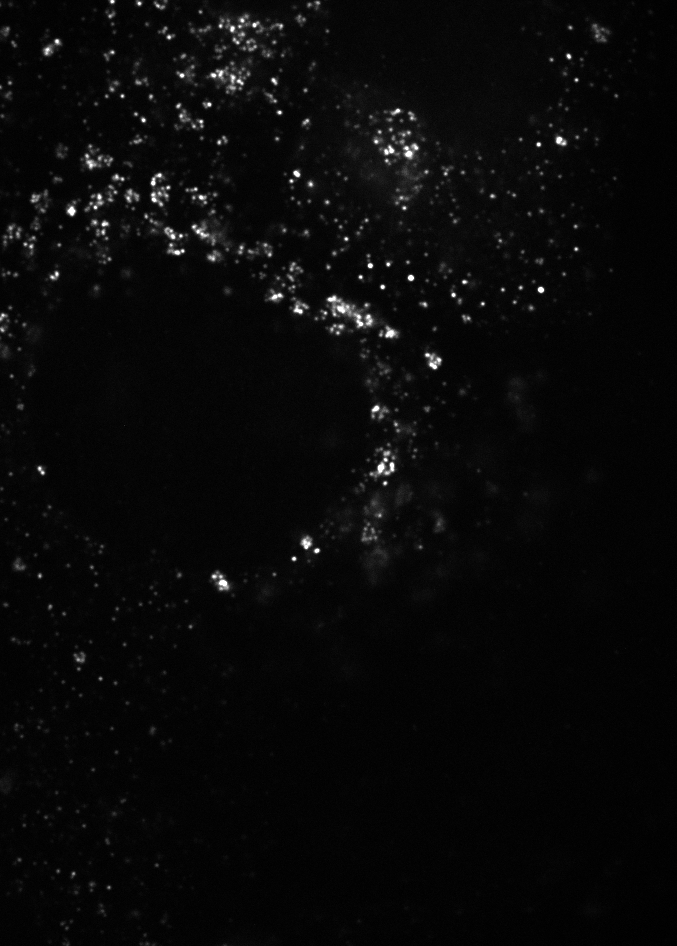

Supplement: Supplementary file 10 — Source data Fig. 4-1 [file 44318_2026_705_MOESM10_ESM.zip › Figure 4-1/B/HCC1954_siCT_NT/20240603_HCCSTARD3sictrl_NT_1_w1SPI 491 GFP.TIF]

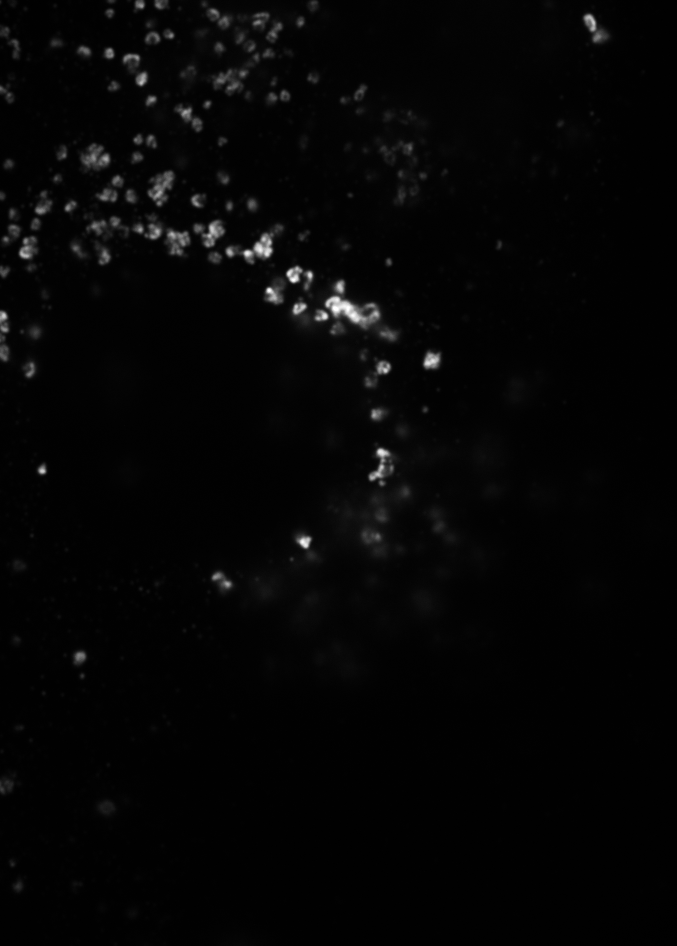

Supplement: Supplementary file 10 — Source data Fig. 4-1 [file 44318_2026_705_MOESM10_ESM.zip › Figure 4-1/B/HCC1954_siCT_NT/20240603_HCCSTARD3sictrl_NT_1_w2SPI 561 mCherry.TIF]

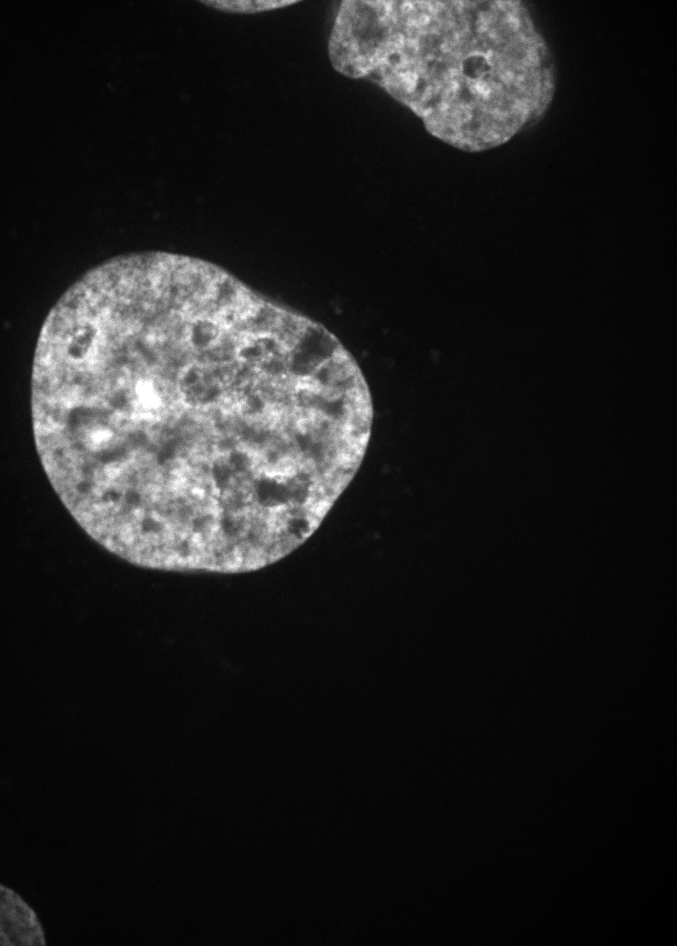

Supplement: Supplementary file 10 — Source data Fig. 4-1 [file 44318_2026_705_MOESM10_ESM.zip › Figure 4-1/B/HCC1954_siCT_NT/20240603_HCCSTARD3sictrl_NT_1_w3SPI 405 DAPI.TIF]

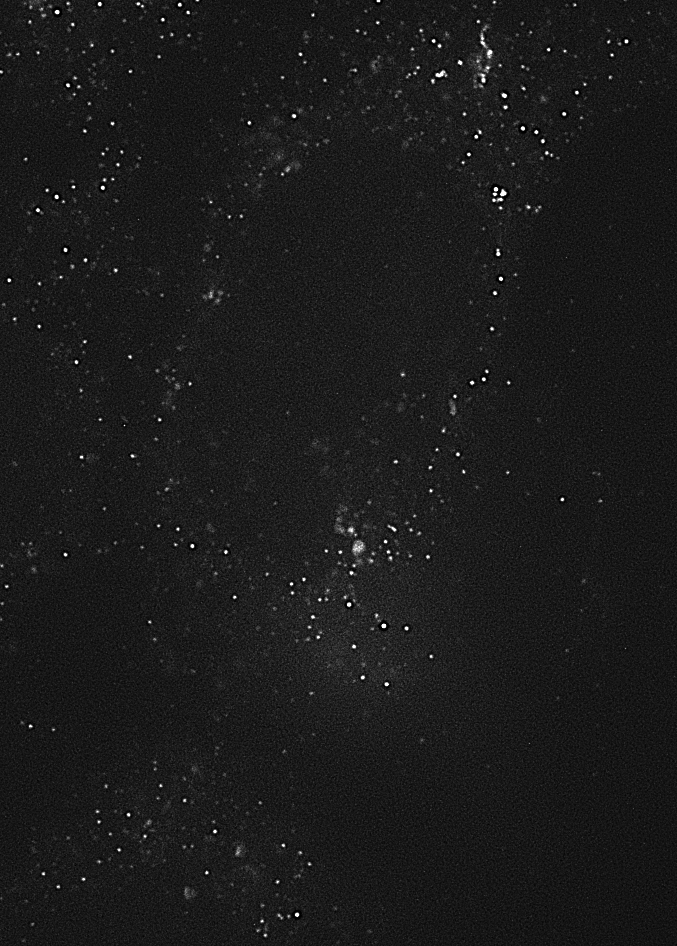

Supplement: Supplementary file 11 — Source data Fig. 4-2 [file 44318_2026_705_MOESM11_ESM.zip › Figure 4-2/C/HCC1954_siSTARD3_CHIR99021/20240603_HCCSTARD3siSTARD3_CHIR_6_SR_w1SPI 491 GFP.TIF]

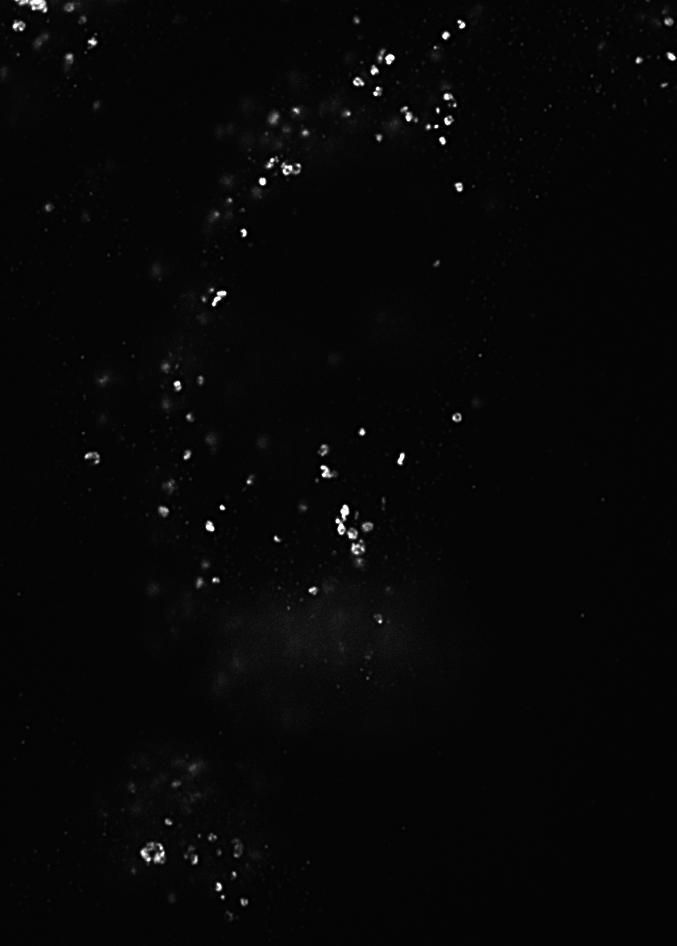

Supplement: Supplementary file 11 — Source data Fig. 4-2 [file 44318_2026_705_MOESM11_ESM.zip › Figure 4-2/C/HCC1954_siSTARD3_CHIR99021/20240603_HCCSTARD3siSTARD3_CHIR_6_SR_w2SPI 561 mCherry.TIF]

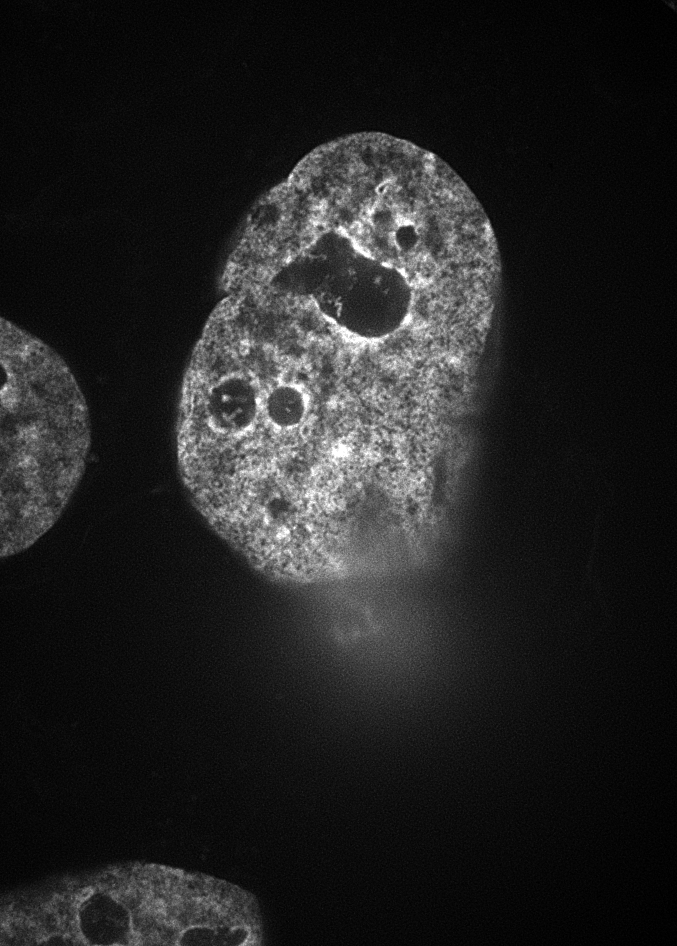

Supplement: Supplementary file 11 — Source data Fig. 4-2 [file 44318_2026_705_MOESM11_ESM.zip › Figure 4-2/C/HCC1954_siSTARD3_CHIR99021/20240603_HCCSTARD3siSTARD3_CHIR_6_SR_w3SPI 405 DAPI.TIF]

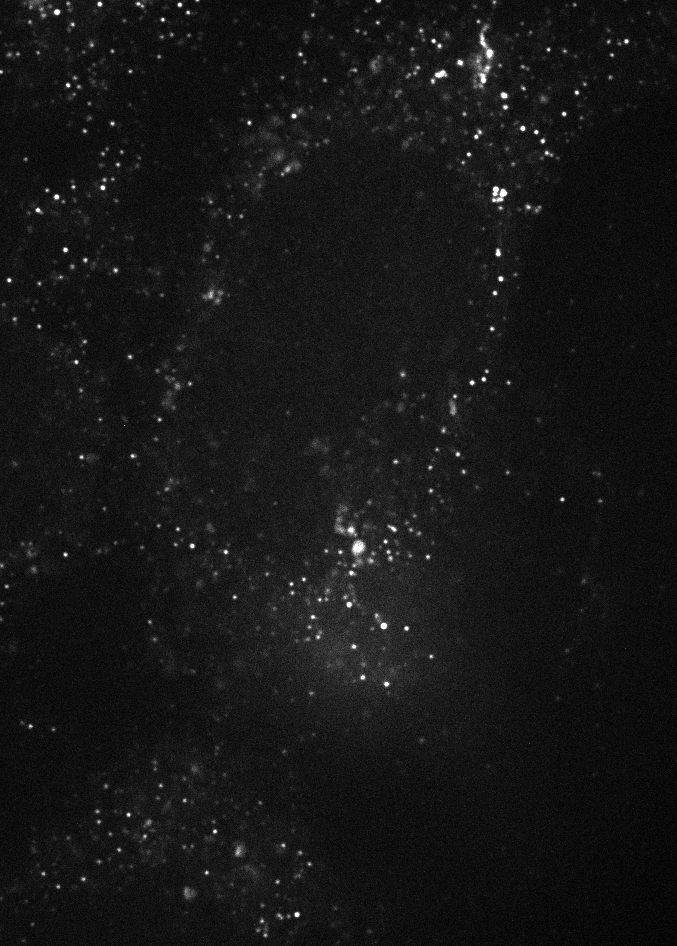

Supplement: Supplementary file 11 — Source data Fig. 4-2 [file 44318_2026_705_MOESM11_ESM.zip › Figure 4-2/C/HCC1954_siSTARD3_CHIR99021/20240603_HCCSTARD3siSTARD3_CHIR_6_w1SPI 491 GFP.TIF]

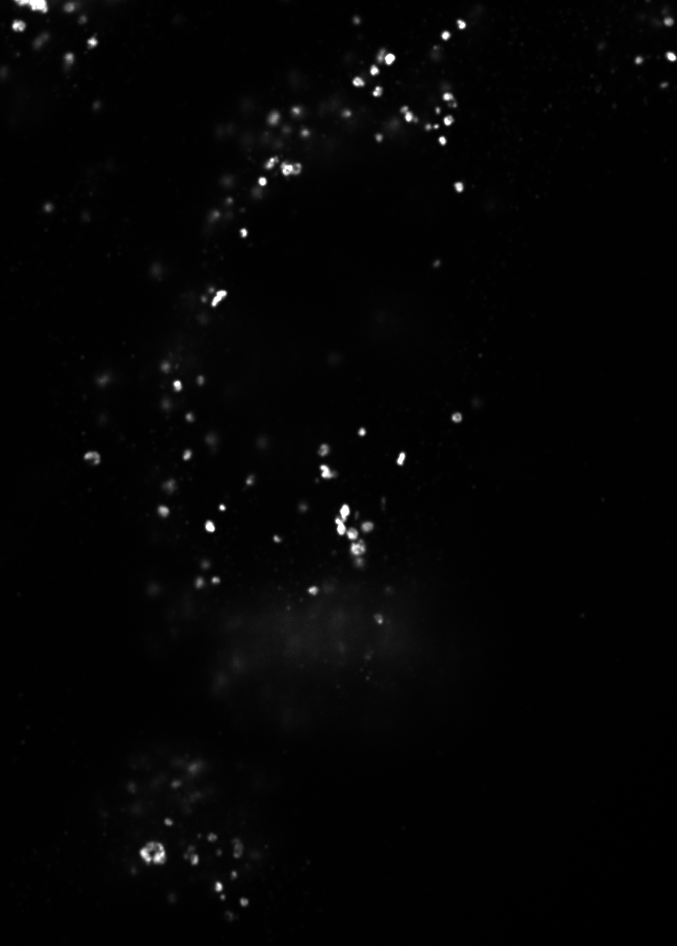

Supplement: Supplementary file 11 — Source data Fig. 4-2 [file 44318_2026_705_MOESM11_ESM.zip › Figure 4-2/C/HCC1954_siSTARD3_CHIR99021/20240603_HCCSTARD3siSTARD3_CHIR_6_w2SPI 561 mCherry.TIF]

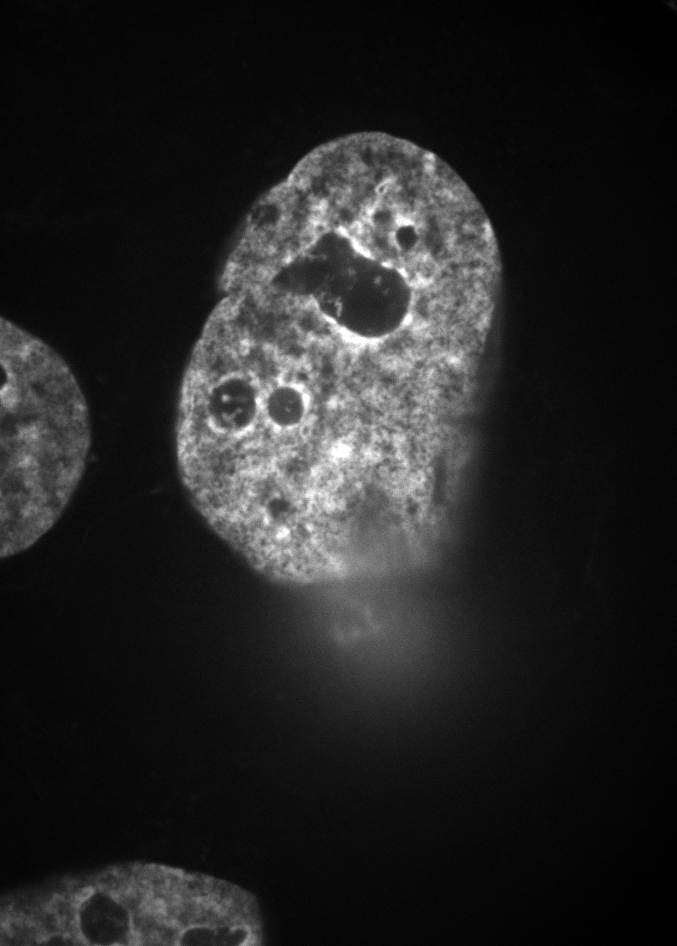

Supplement: Supplementary file 11 — Source data Fig. 4-2 [file 44318_2026_705_MOESM11_ESM.zip › Figure 4-2/C/HCC1954_siSTARD3_CHIR99021/20240603_HCCSTARD3siSTARD3_CHIR_6_w3SPI 405 DAPI.TIF]

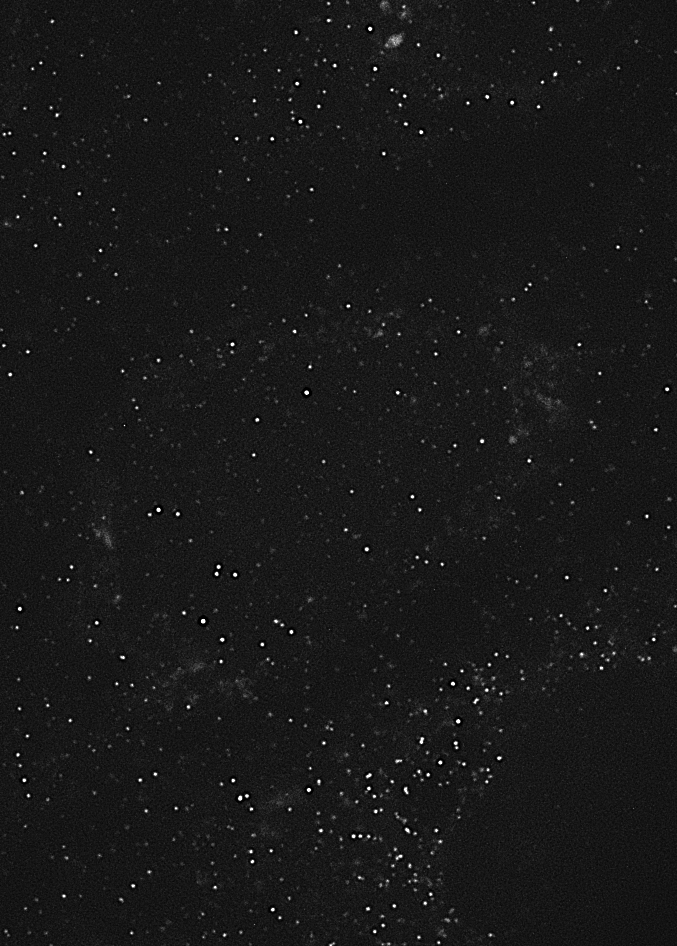

Supplement: Supplementary file 11 — Source data Fig. 4-2 [file 44318_2026_705_MOESM11_ESM.zip › Figure 4-2/C/HCC1954_siSTARD3_NT/20240603_HCCSTARD3siSTARD3_NT_1_SR_w1SPI 491 GFP.TIF]

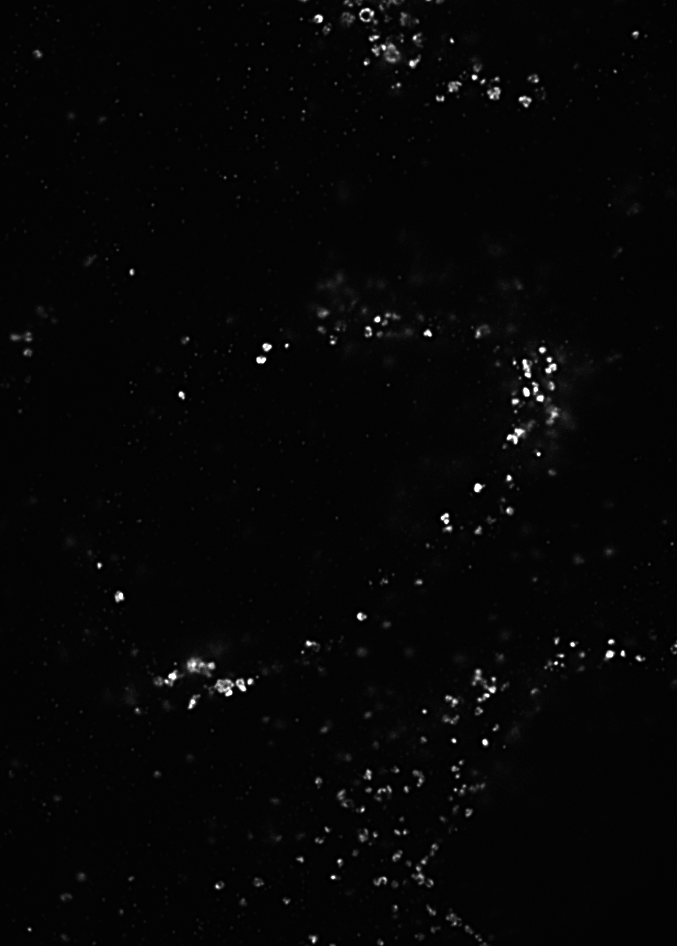

Supplement: Supplementary file 11 — Source data Fig. 4-2 [file 44318_2026_705_MOESM11_ESM.zip › Figure 4-2/C/HCC1954_siSTARD3_NT/20240603_HCCSTARD3siSTARD3_NT_1_SR_w2SPI 561 mCherry.TIF]

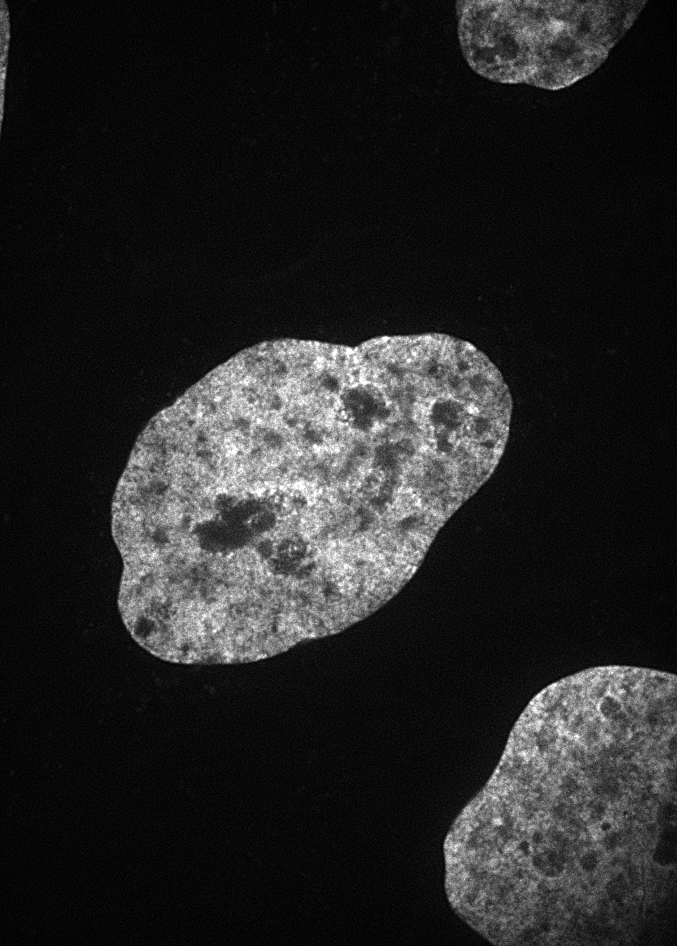

Supplement: Supplementary file 11 — Source data Fig. 4-2 [file 44318_2026_705_MOESM11_ESM.zip › Figure 4-2/C/HCC1954_siSTARD3_NT/20240603_HCCSTARD3siSTARD3_NT_1_SR_w3SPI 405 DAPI.TIF]

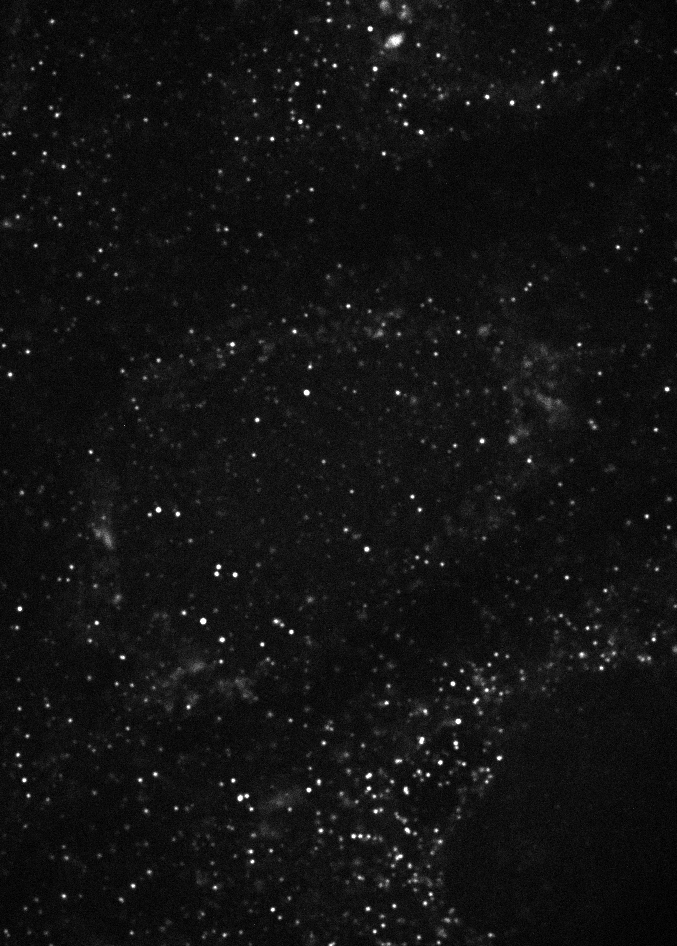

Supplement: Supplementary file 11 — Source data Fig. 4-2 [file 44318_2026_705_MOESM11_ESM.zip › Figure 4-2/C/HCC1954_siSTARD3_NT/20240603_HCCSTARD3siSTARD3_NT_1_w1SPI 491 GFP.TIF]

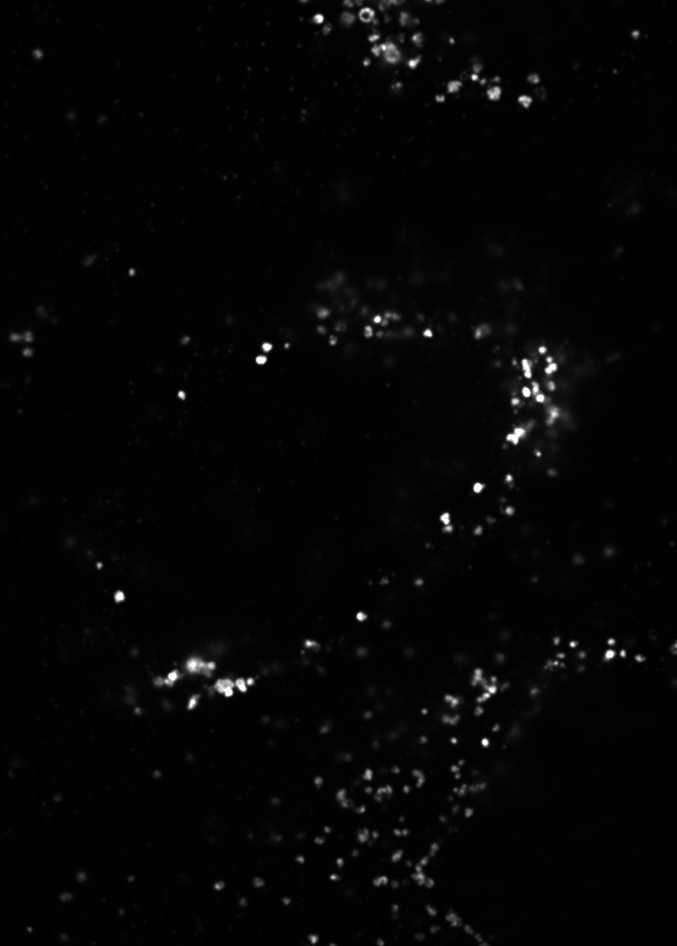

Supplement: Supplementary file 11 — Source data Fig. 4-2 [file 44318_2026_705_MOESM11_ESM.zip › Figure 4-2/C/HCC1954_siSTARD3_NT/20240603_HCCSTARD3siSTARD3_NT_1_w2SPI 561 mCherry.TIF]

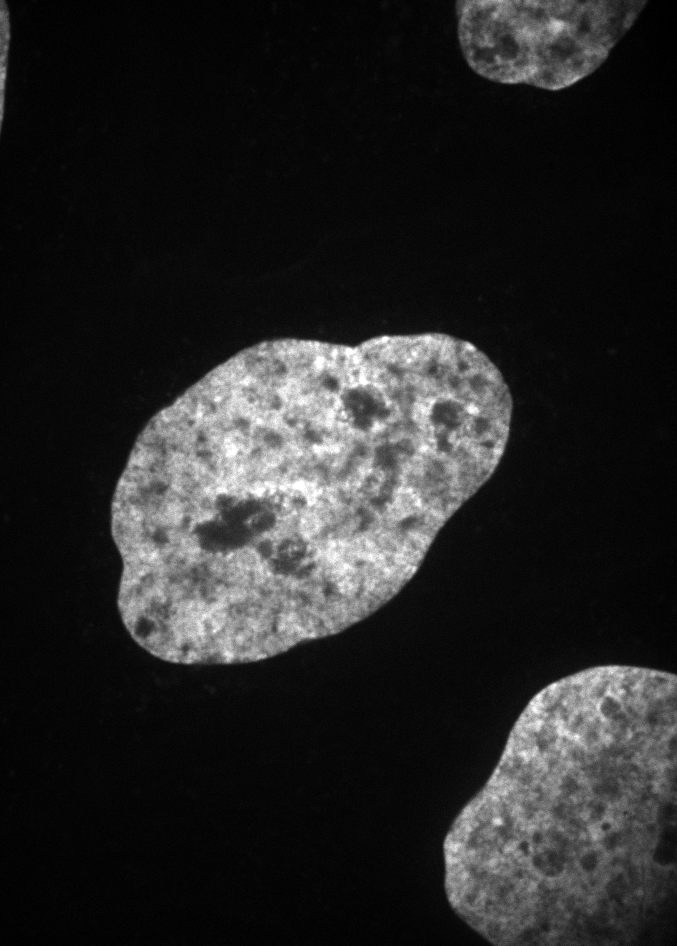

Supplement: Supplementary file 11 — Source data Fig. 4-2 [file 44318_2026_705_MOESM11_ESM.zip › Figure 4-2/C/HCC1954_siSTARD3_NT/20240603_HCCSTARD3siSTARD3_NT_1_w3SPI 405 DAPI.TIF]

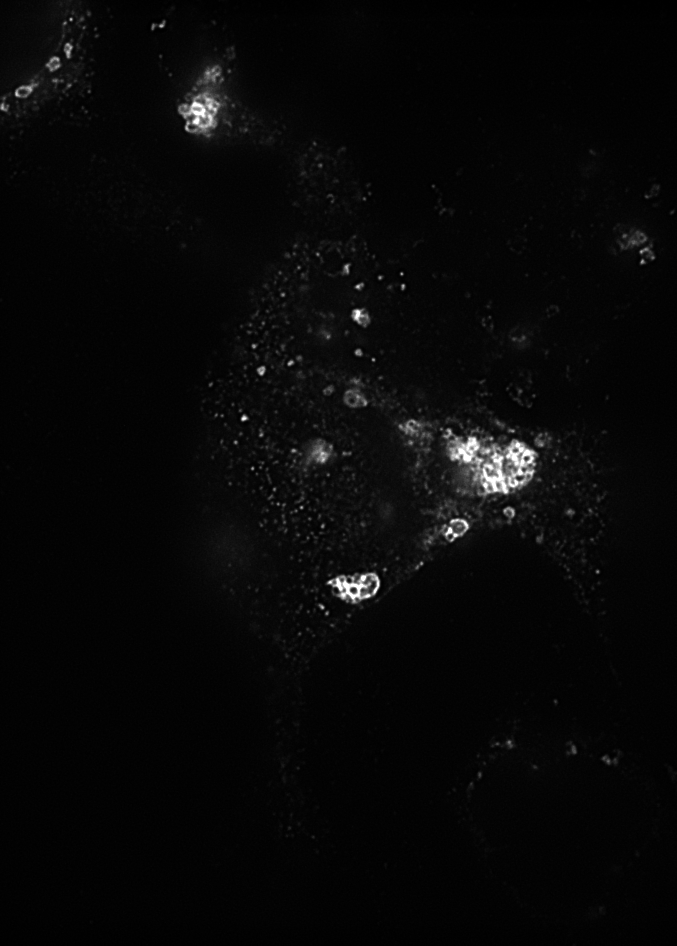

Supplement: Supplementary file 11 — Source data Fig. 4-2 [file 44318_2026_705_MOESM11_ESM.zip › Figure 4-2/E/MCF7_STARD3WT_CHIR99021/20230310_MCF7STARD3WT_GSK3i_2_SR_w1SPI 561 mCherry.TIF]

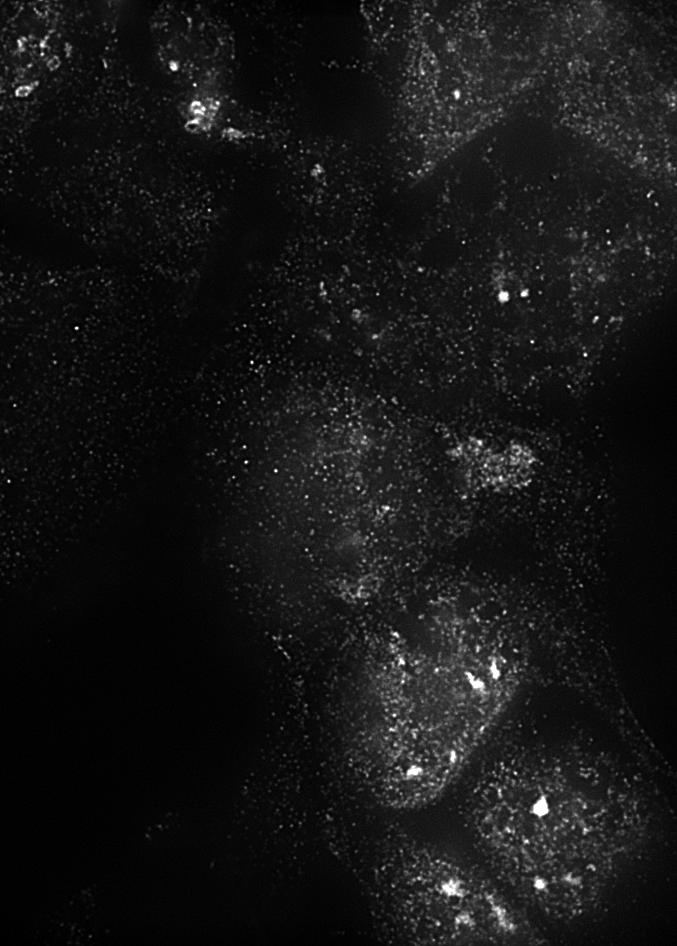

Supplement: Supplementary file 11 — Source data Fig. 4-2 [file 44318_2026_705_MOESM11_ESM.zip › Figure 4-2/E/MCF7_STARD3WT_CHIR99021/20230310_MCF7STARD3WT_GSK3i_2_SR_w2SPI 491 GFP.TIF]

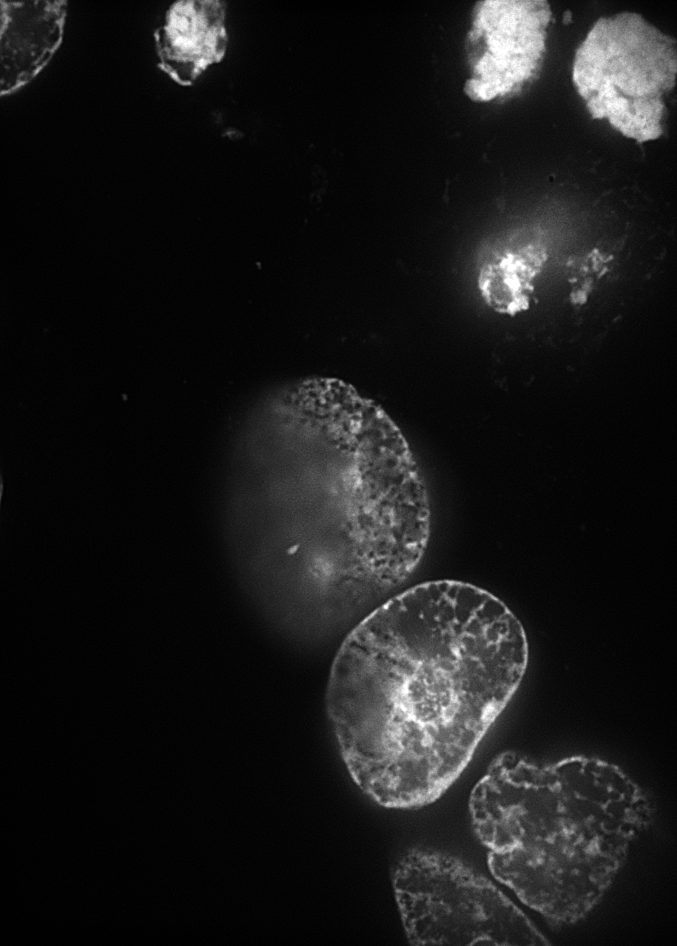

Supplement: Supplementary file 11 — Source data Fig. 4-2 [file 44318_2026_705_MOESM11_ESM.zip › Figure 4-2/E/MCF7_STARD3WT_CHIR99021/20230310_MCF7STARD3WT_GSK3i_2_SR_w3SPI 405 DAPI.TIF]

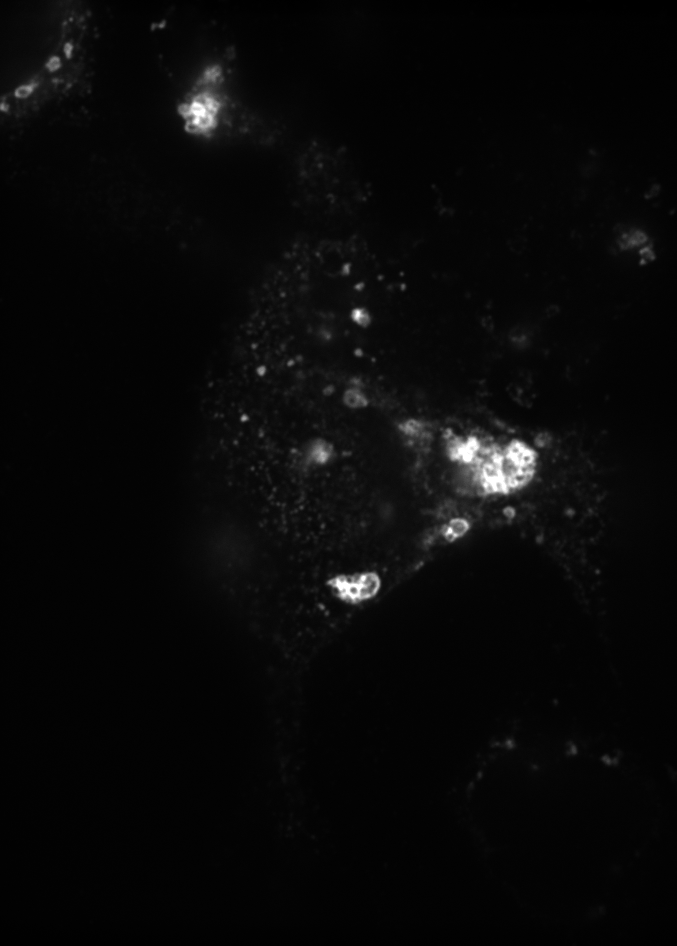

Supplement: Supplementary file 11 — Source data Fig. 4-2 [file 44318_2026_705_MOESM11_ESM.zip › Figure 4-2/E/MCF7_STARD3WT_CHIR99021/20230310_MCF7STARD3WT_GSK3i_2_w1SPI 561 mCherry.TIF]

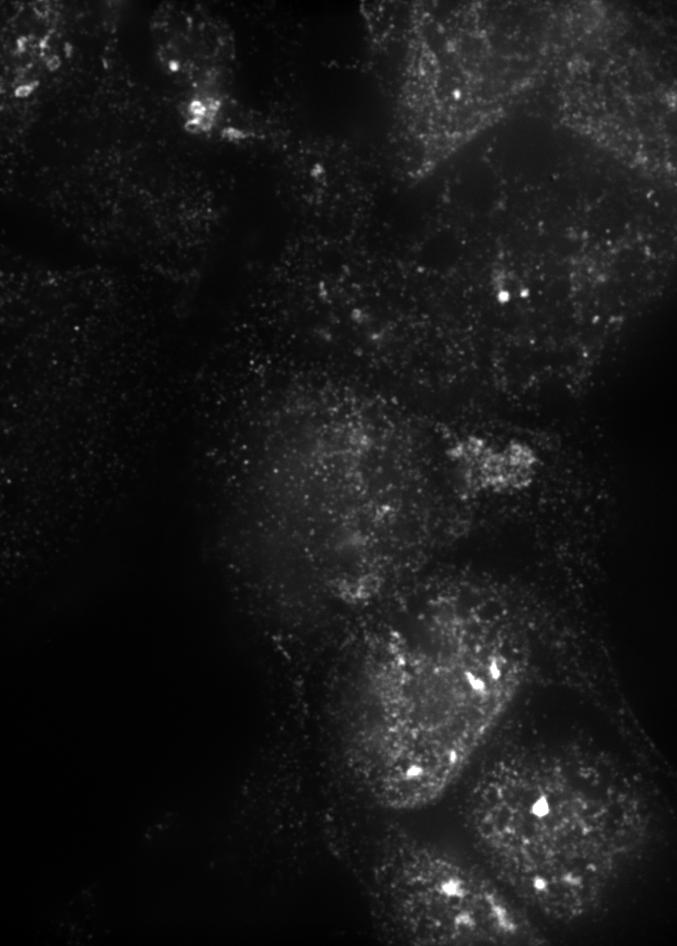

Supplement: Supplementary file 11 — Source data Fig. 4-2 [file 44318_2026_705_MOESM11_ESM.zip › Figure 4-2/E/MCF7_STARD3WT_CHIR99021/20230310_MCF7STARD3WT_GSK3i_2_w2SPI 491 GFP.TIF]

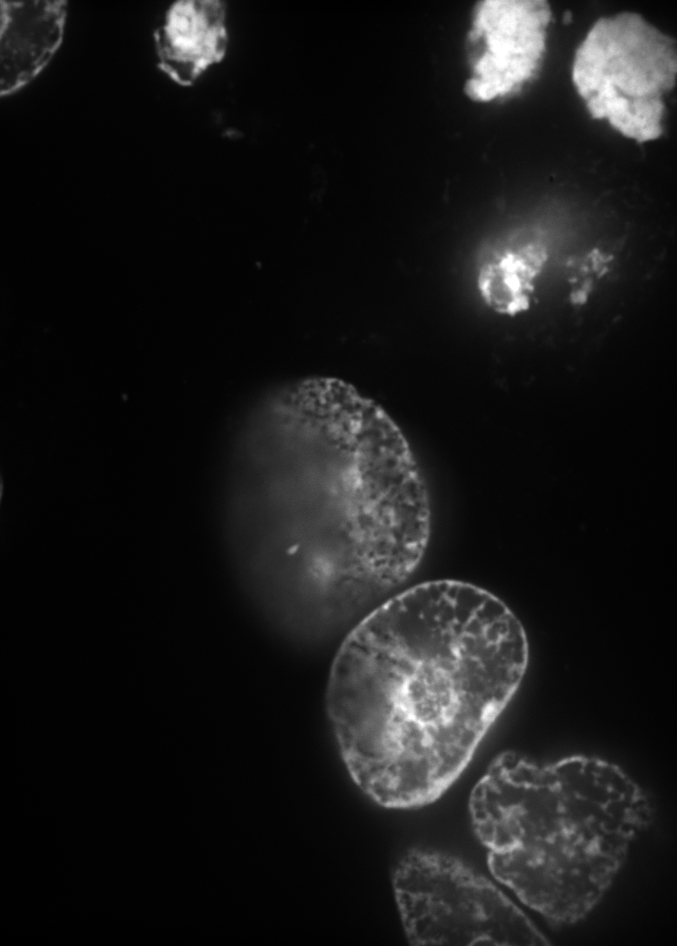

Supplement: Supplementary file 11 — Source data Fig. 4-2 [file 44318_2026_705_MOESM11_ESM.zip › Figure 4-2/E/MCF7_STARD3WT_CHIR99021/20230310_MCF7STARD3WT_GSK3i_2_w3SPI 405 DAPI.TIF]

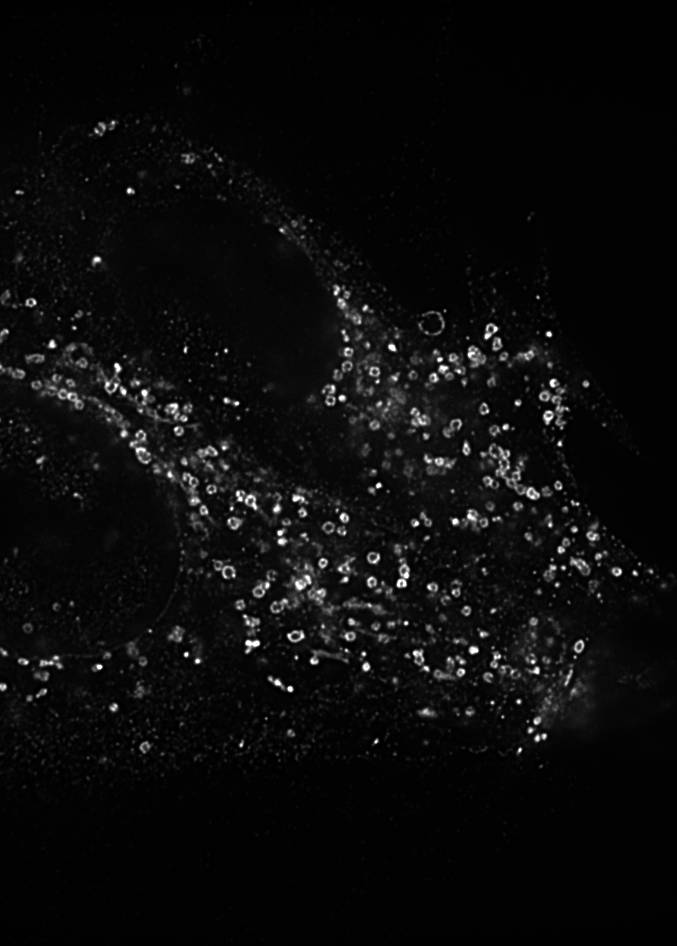

Supplement: Supplementary file 11 — Source data Fig. 4-2 [file 44318_2026_705_MOESM11_ESM.zip › Figure 4-2/E/MCF7_STARD3WT_NT/20230310_MCF7STARD3WT_NT_3_SR_w1SPI 561 mCherry.TIF]
