# Supplementary material for: STARD3 regulates lysosome positioning and contacts via a GSK3-controlled phosphorylation switch
Source: EMBO J. 2026 Feb 25;45(7):2239–77. doi: 10.1038/s44318-026-00705-3 (PMC13044316; doi:10.1038/s44318-026-00705-3)

Exp 1

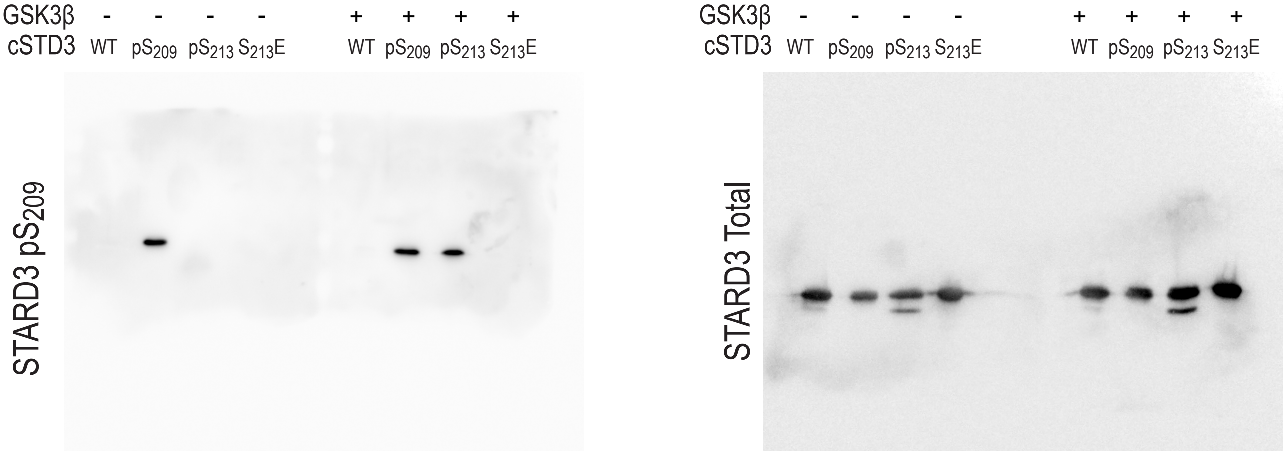

Exp 2

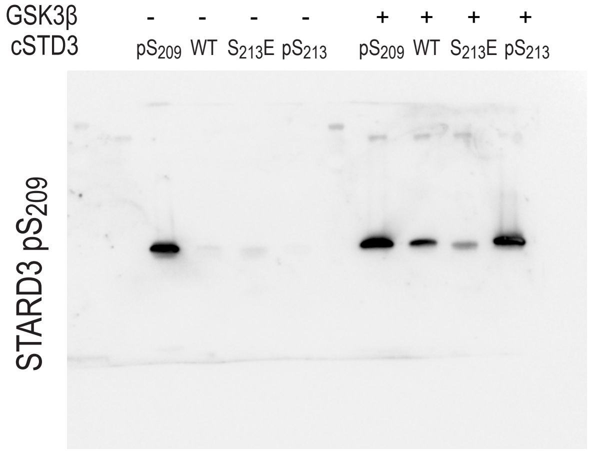

Exp 3

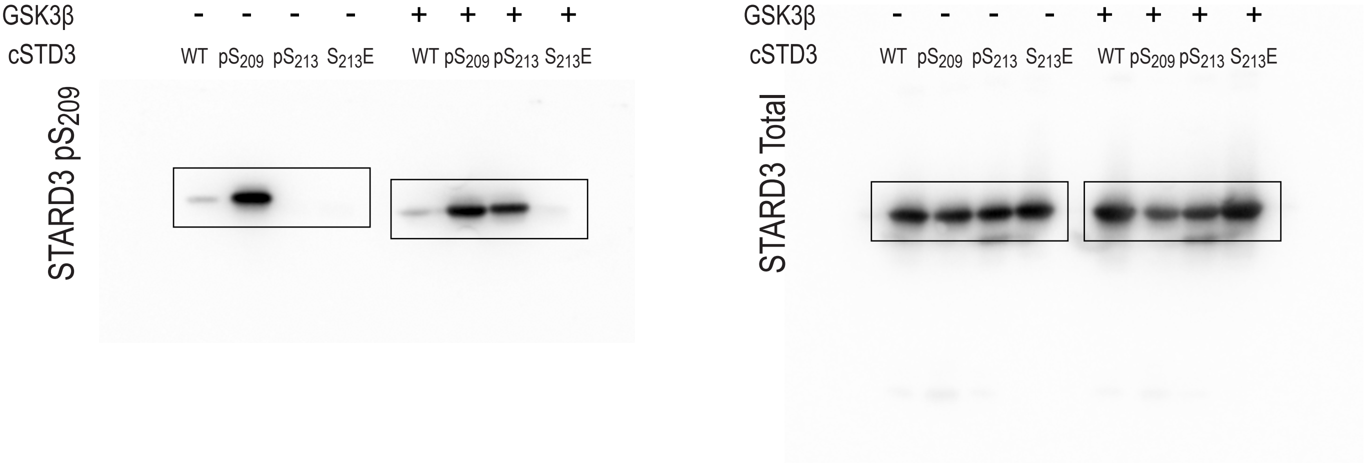

Supplement: Supplementary file 8 — Source data Fig. 2 [file 44318_2026_705_MOESM8_ESM.zip › Figure 2/F/Kinase assay_WB.pdf]
