# Supplementary material for: STARD3 regulates lysosome positioning and contacts via a GSK3-controlled phosphorylation switch
Source: EMBO J. 2026 Feb 25;45(7):2239–77. doi: 10.1038/s44318-026-00705-3 (PMC13044316; doi:10.1038/s44318-026-00705-3)
Supplement: Supplementary file 9 — Source data Fig. 3 [file 44318_2026_705_MOESM9_ESM.zip › Figure 3/A/GFP Trap-VAPA_WB.pdf]

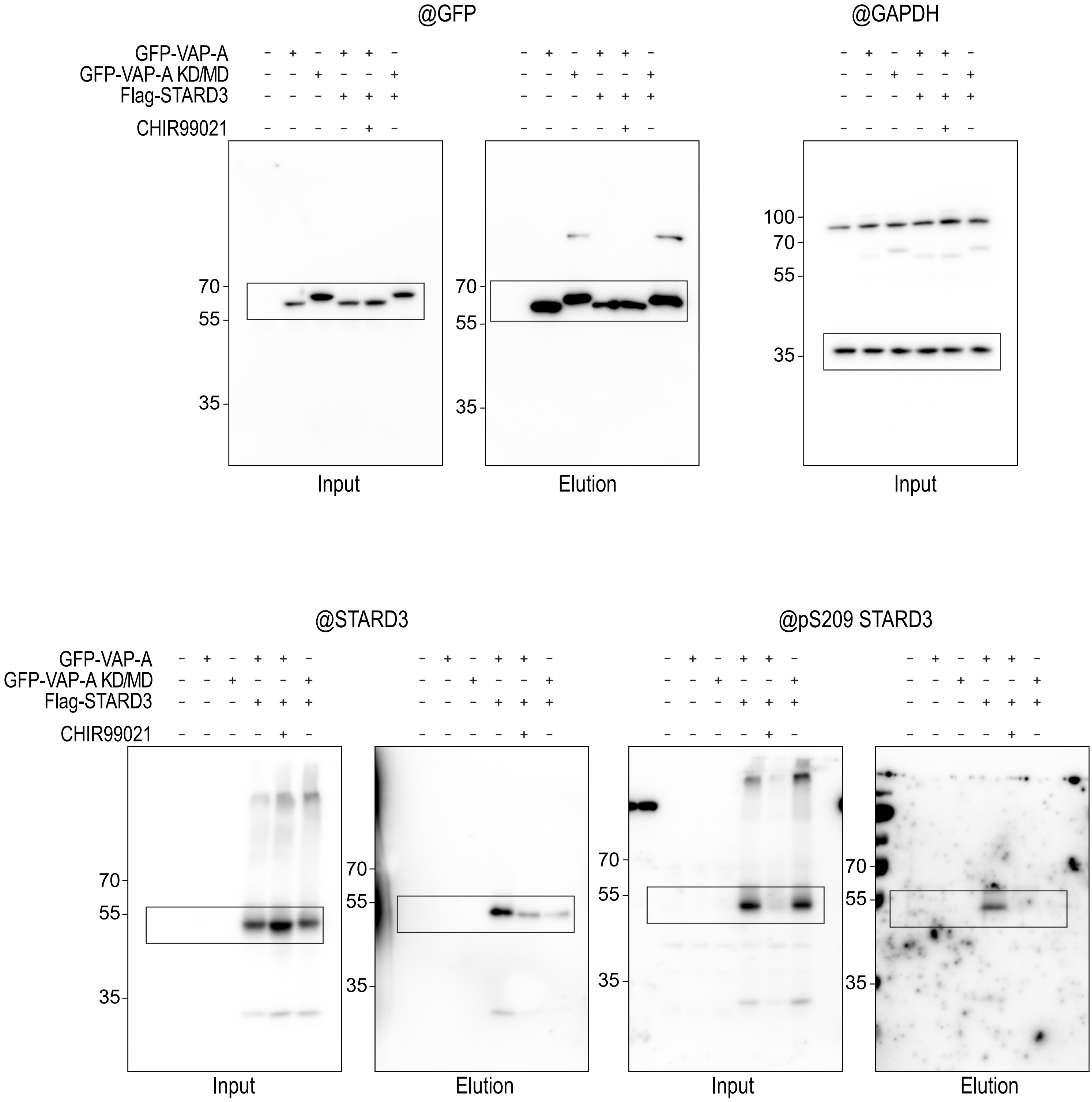
@GFP
@GAPDH
GFP-VAP-A
GFP-VAP-A KD/MD
Flag-STARD3
CHIR99021
100
70
55
35
Input
Elution
@STARD3
@pS209 STARD3
