# Supplementary material for: STARD3 regulates lysosome positioning and contacts via a GSK3-controlled phosphorylation switch
Source: EMBO J. 2026 Feb 25;45(7):2239–77. doi: 10.1038/s44318-026-00705-3 (PMC13044316; doi:10.1038/s44318-026-00705-3)
Supplement: Supplementary file 9 — Source data Fig. 3 [file 44318_2026_705_MOESM9_ESM.zip › Figure 3/D/IP-STARD3_WB.pdf]

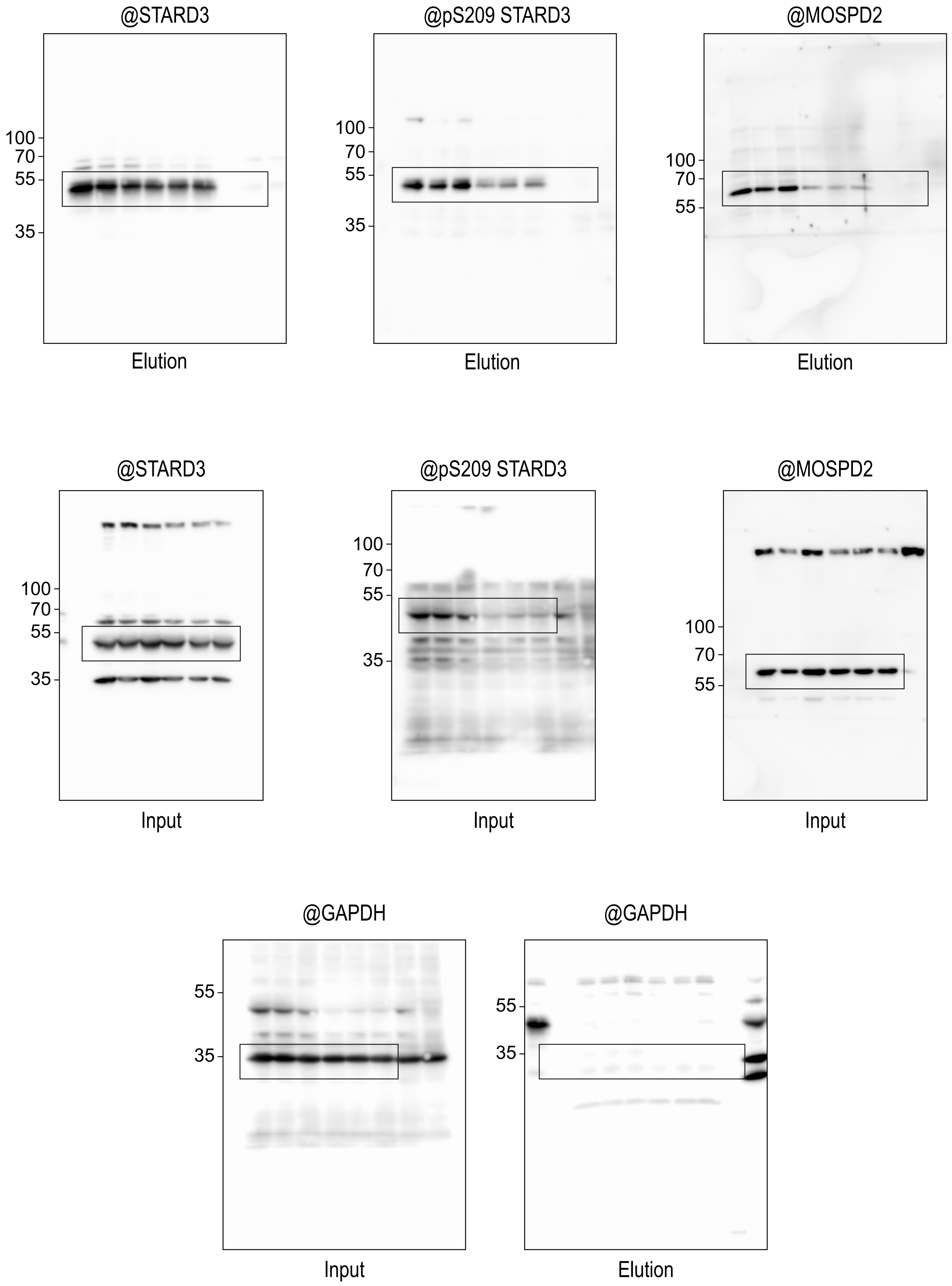
@STARD3
100
70
55
35
Elution
@pS209 STARD3
100
70
55
35
Elution
@MOSPD2
100
70
55
Elution
@STARD3
100
70
55
35
Input
@pS209 STARD3
100
70
55
35
Input
@MOSPD2
100
70
55
Input
@GAPDH
55
35
Input
@GAPDH
55
35
Elution
