# Supplementary figures and images for: STARD3 regulates lysosome positioning and contacts via a GSK3-controlled phosphorylation switch (part 2 of 7)
Source: EMBO J. 2026 Feb 25;45(7):2239–77. doi: 10.1038/s44318-026-00705-3 (PMC13044316; doi:10.1038/s44318-026-00705-3)

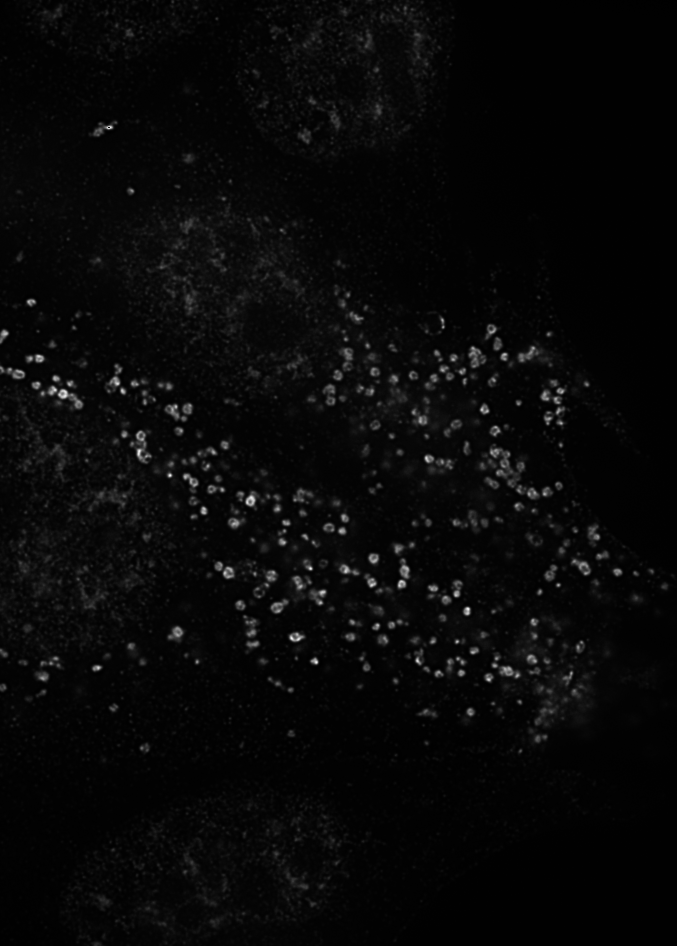

Supplement: Supplementary file 11 — Source data Fig. 4-2 [file 44318_2026_705_MOESM11_ESM.zip › Figure 4-2/E/MCF7_STARD3WT_NT/20230310_MCF7STARD3WT_NT_3_SR_w2SPI 491 GFP.TIF]

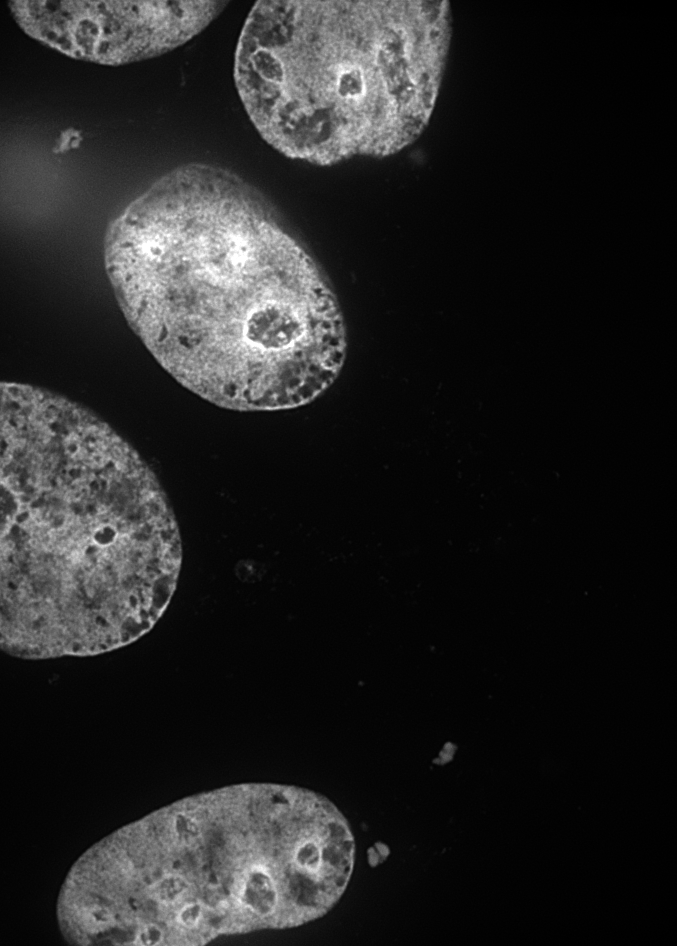

Supplement: Supplementary file 11 — Source data Fig. 4-2 [file 44318_2026_705_MOESM11_ESM.zip › Figure 4-2/E/MCF7_STARD3WT_NT/20230310_MCF7STARD3WT_NT_3_SR_w3SPI 405 DAPI.TIF]

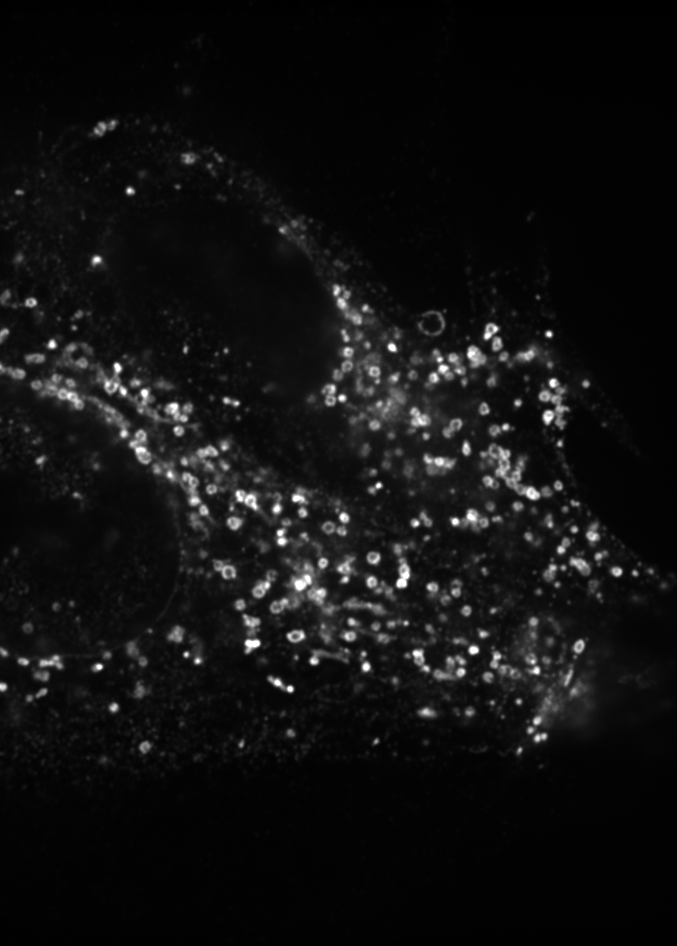

Supplement: Supplementary file 11 — Source data Fig. 4-2 [file 44318_2026_705_MOESM11_ESM.zip › Figure 4-2/E/MCF7_STARD3WT_NT/20230310_MCF7STARD3WT_NT_3_w1SPI 561 mCherry.TIF]

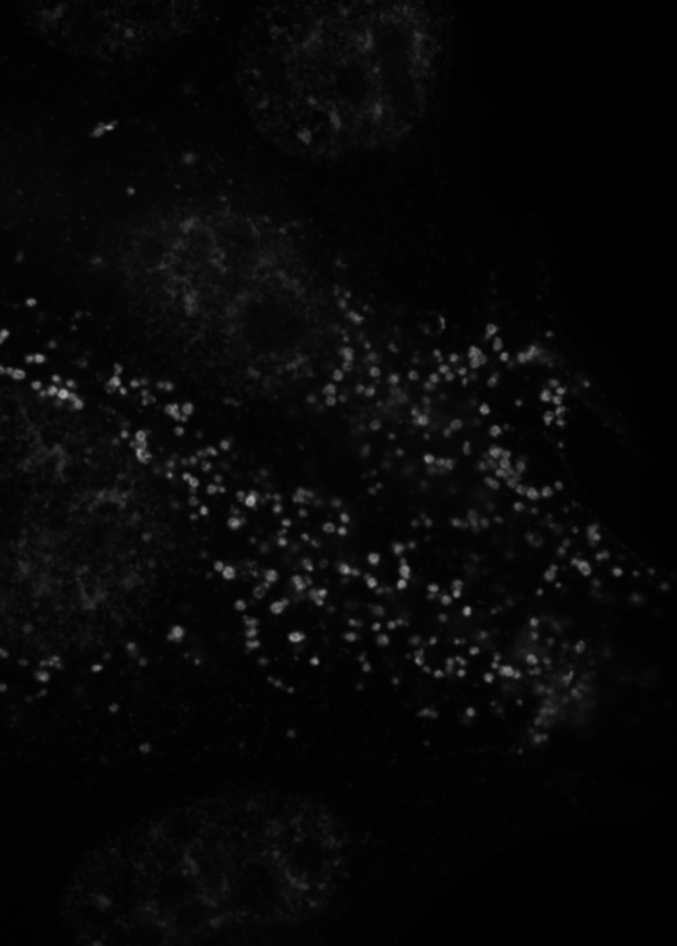

Supplement: Supplementary file 11 — Source data Fig. 4-2 [file 44318_2026_705_MOESM11_ESM.zip › Figure 4-2/E/MCF7_STARD3WT_NT/20230310_MCF7STARD3WT_NT_3_w2SPI 491 GFP.TIF]

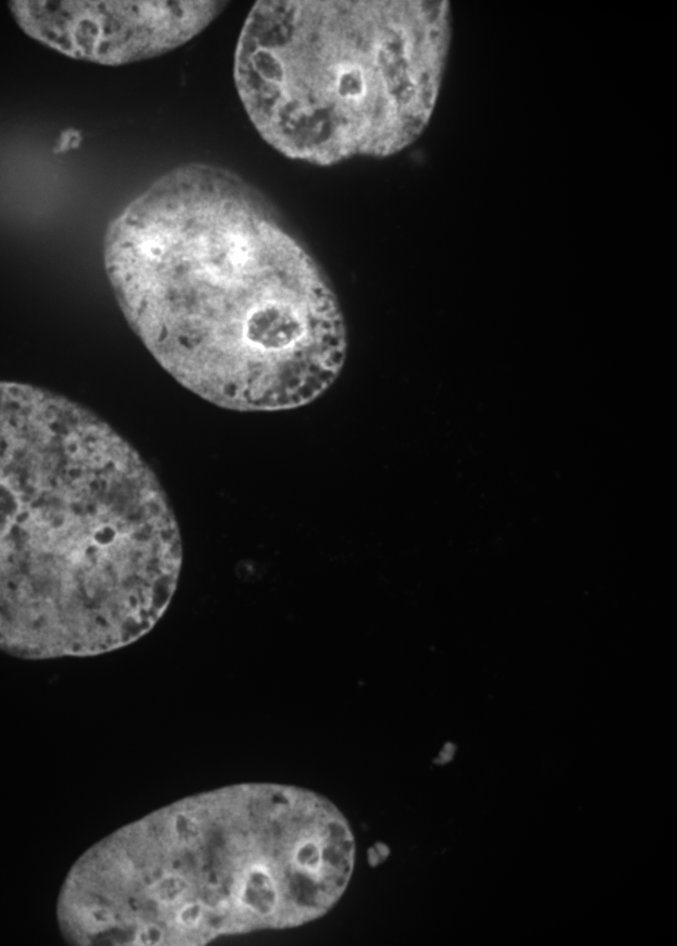

Supplement: Supplementary file 11 — Source data Fig. 4-2 [file 44318_2026_705_MOESM11_ESM.zip › Figure 4-2/E/MCF7_STARD3WT_NT/20230310_MCF7STARD3WT_NT_3_w3SPI 405 DAPI.TIF]

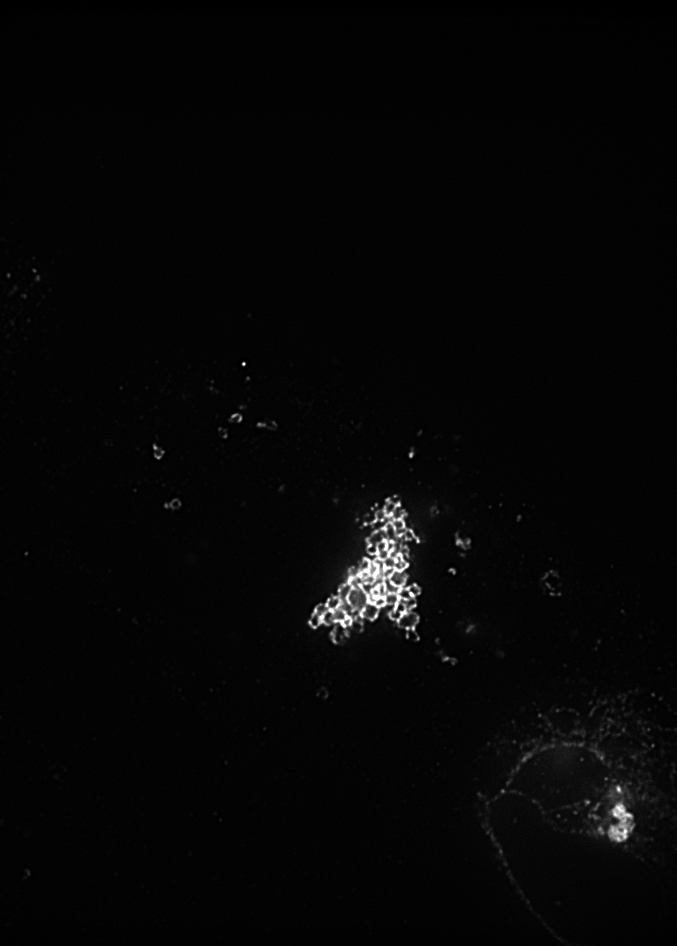

Supplement: Supplementary file 11 — Source data Fig. 4-2 [file 44318_2026_705_MOESM11_ESM.zip › Figure 4-2/F/MCF7STARD3S209A_CHIR99021/20230310_MCF7STARD3S209A_GSK3i_1_SR_w1SPI 561 mCherry.TIF]

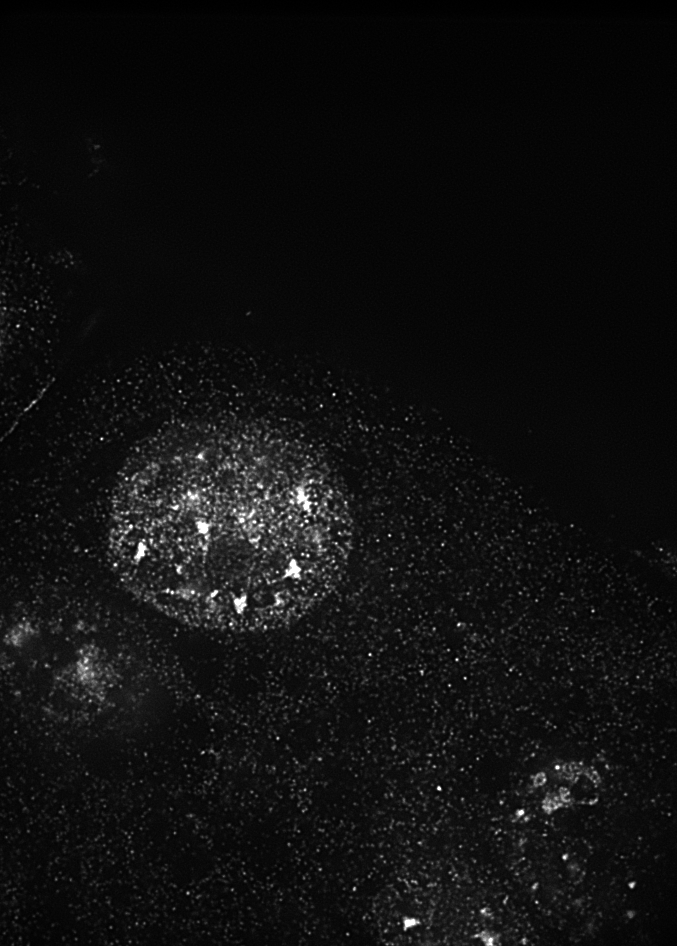

Supplement: Supplementary file 11 — Source data Fig. 4-2 [file 44318_2026_705_MOESM11_ESM.zip › Figure 4-2/F/MCF7STARD3S209A_CHIR99021/20230310_MCF7STARD3S209A_GSK3i_1_SR_w2SPI 491 GFP.TIF]

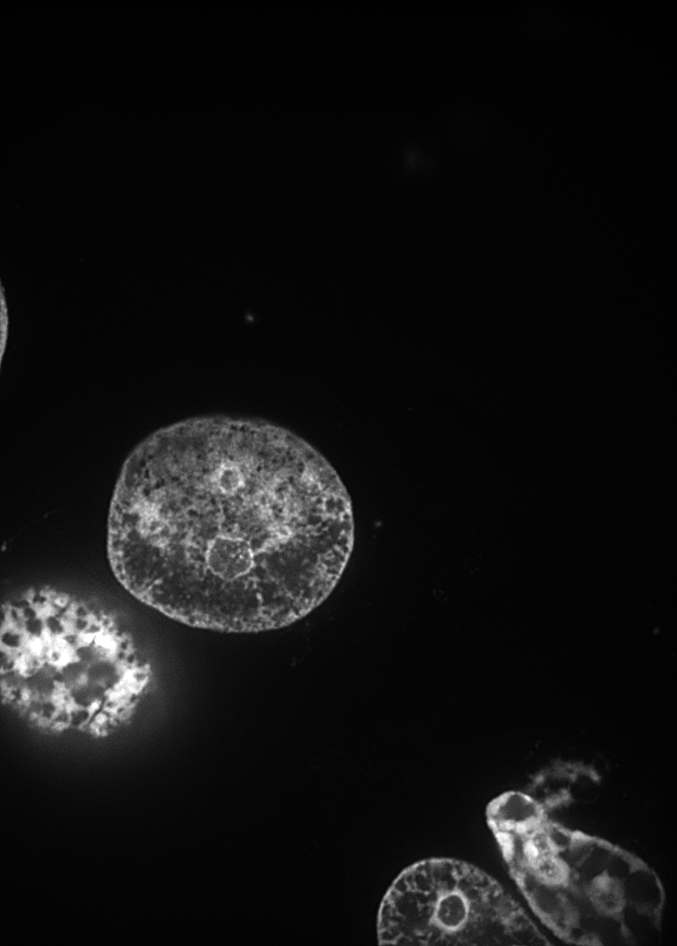

Supplement: Supplementary file 11 — Source data Fig. 4-2 [file 44318_2026_705_MOESM11_ESM.zip › Figure 4-2/F/MCF7STARD3S209A_CHIR99021/20230310_MCF7STARD3S209A_GSK3i_1_SR_w3SPI 405 DAPI.TIF]

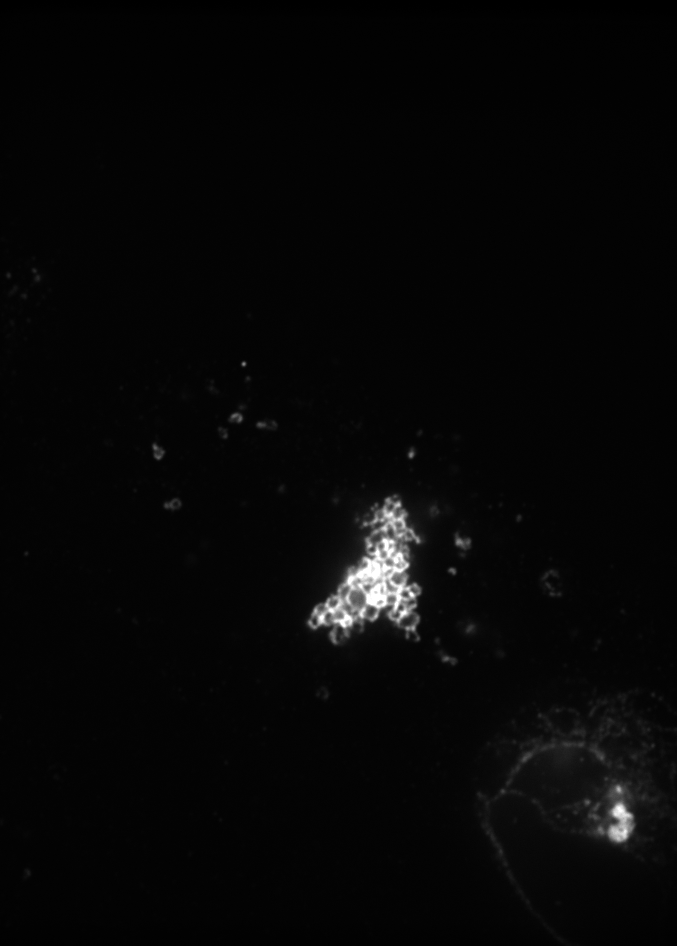

Supplement: Supplementary file 11 — Source data Fig. 4-2 [file 44318_2026_705_MOESM11_ESM.zip › Figure 4-2/F/MCF7STARD3S209A_CHIR99021/20230310_MCF7STARD3S209A_GSK3i_1_w1SPI 561 mCherry.TIF]

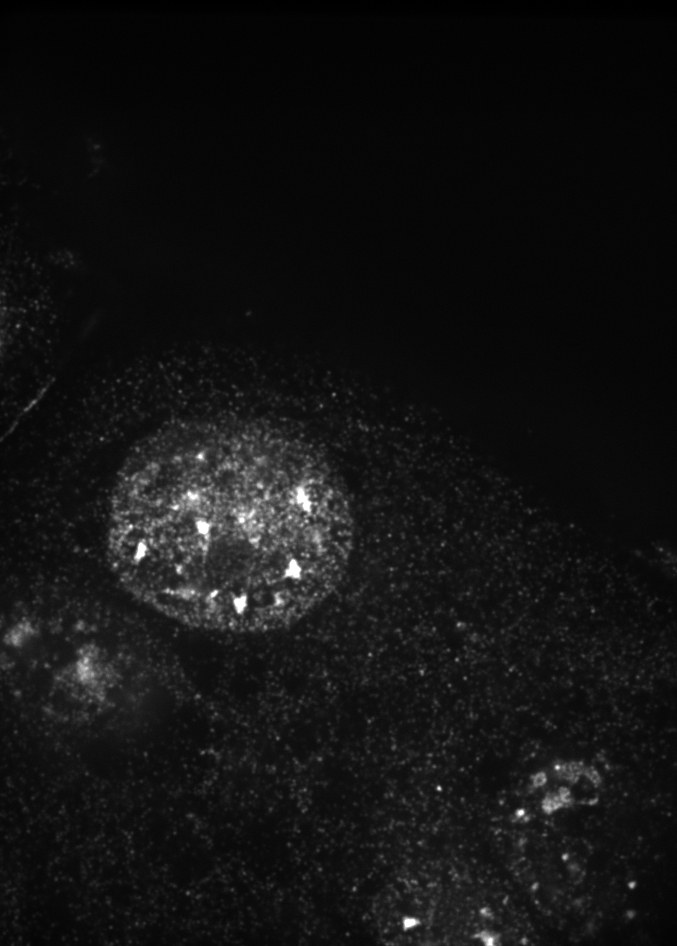

Supplement: Supplementary file 11 — Source data Fig. 4-2 [file 44318_2026_705_MOESM11_ESM.zip › Figure 4-2/F/MCF7STARD3S209A_CHIR99021/20230310_MCF7STARD3S209A_GSK3i_1_w2SPI 491 GFP.TIF]

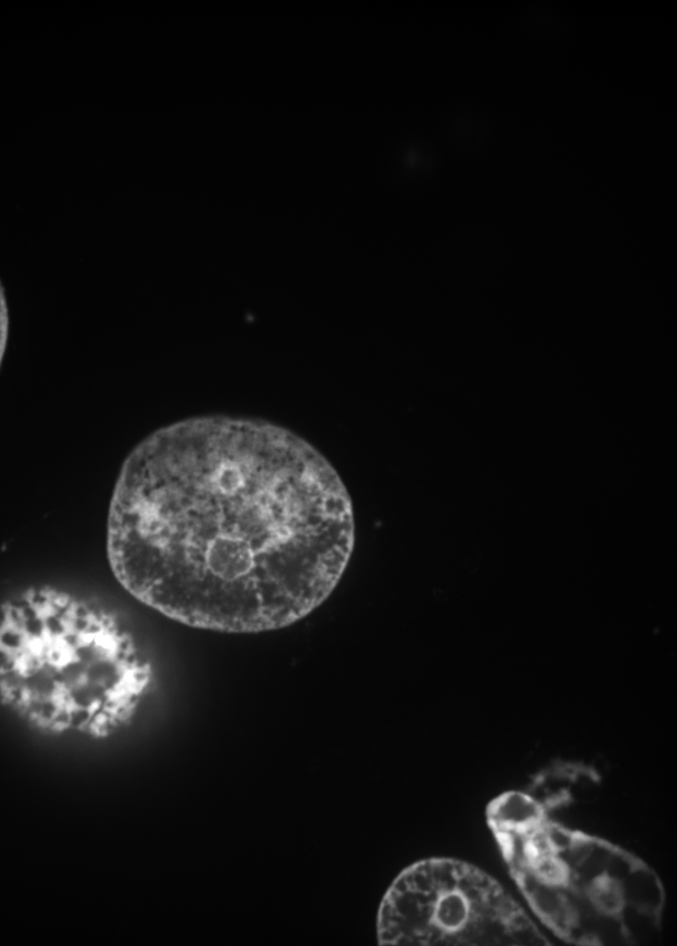

Supplement: Supplementary file 11 — Source data Fig. 4-2 [file 44318_2026_705_MOESM11_ESM.zip › Figure 4-2/F/MCF7STARD3S209A_CHIR99021/20230310_MCF7STARD3S209A_GSK3i_1_w3SPI 405 DAPI.TIF]

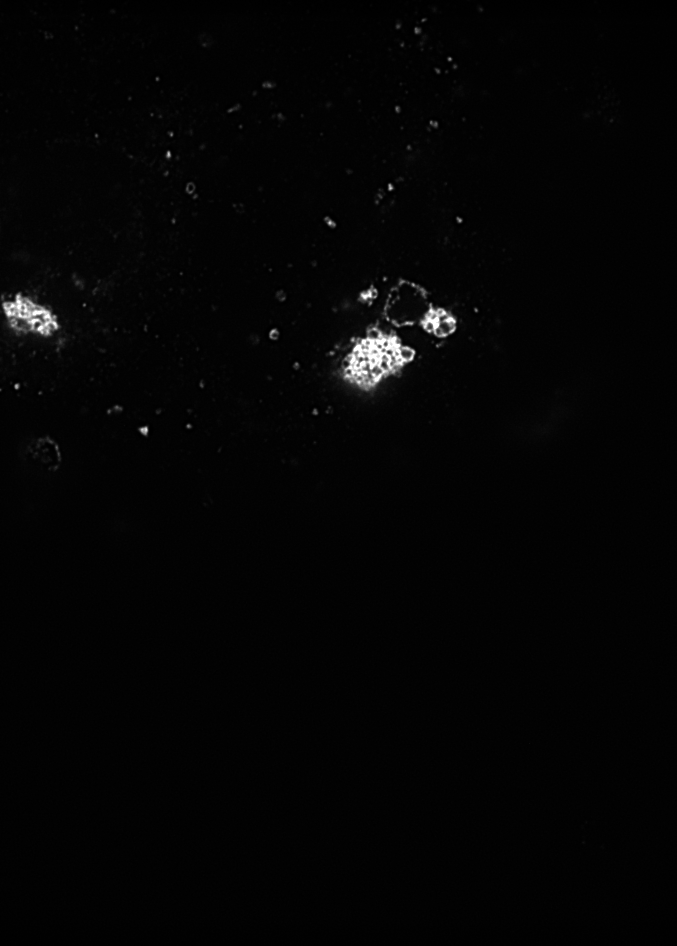

Supplement: Supplementary file 11 — Source data Fig. 4-2 [file 44318_2026_705_MOESM11_ESM.zip › Figure 4-2/F/MCF7_STARD3S209A_NT/20230310_MCF7STARD3S209A_NT_3_SR_w1SPI 561 mCherry.TIF]

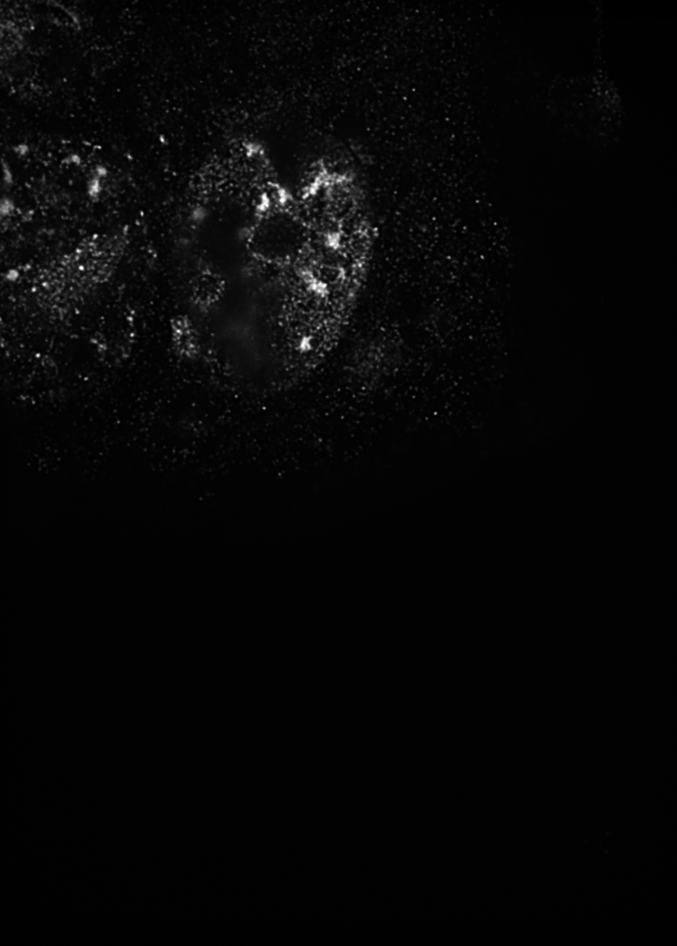

Supplement: Supplementary file 11 — Source data Fig. 4-2 [file 44318_2026_705_MOESM11_ESM.zip › Figure 4-2/F/MCF7_STARD3S209A_NT/20230310_MCF7STARD3S209A_NT_3_SR_w2SPI 491 GFP.TIF]

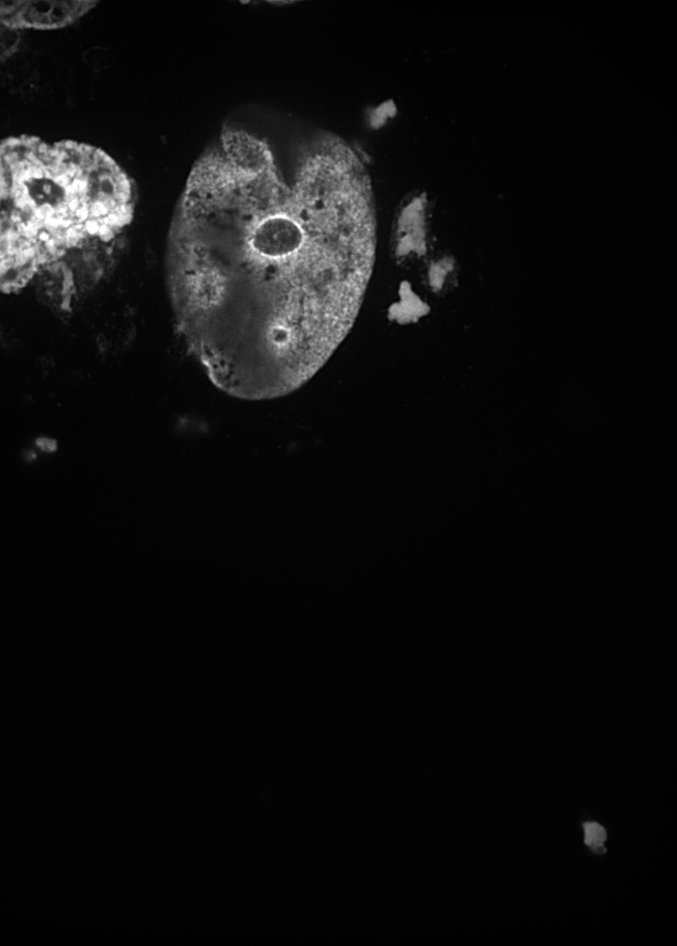

Supplement: Supplementary file 11 — Source data Fig. 4-2 [file 44318_2026_705_MOESM11_ESM.zip › Figure 4-2/F/MCF7_STARD3S209A_NT/20230310_MCF7STARD3S209A_NT_3_SR_w3SPI 405 DAPI.TIF]

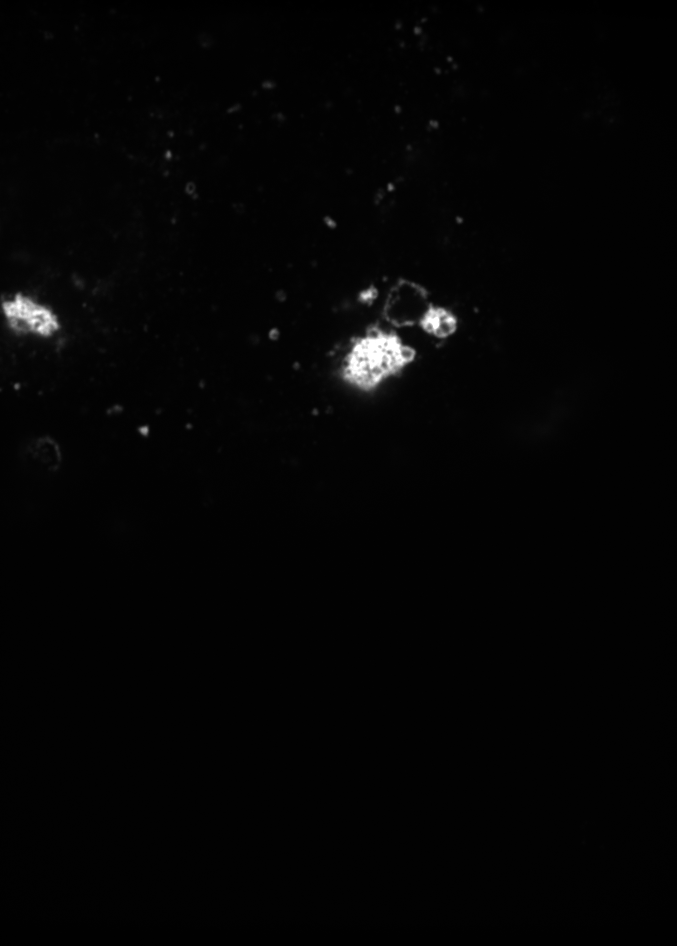

Supplement: Supplementary file 11 — Source data Fig. 4-2 [file 44318_2026_705_MOESM11_ESM.zip › Figure 4-2/F/MCF7_STARD3S209A_NT/20230310_MCF7STARD3S209A_NT_3_w1SPI 561 mCherry.TIF]

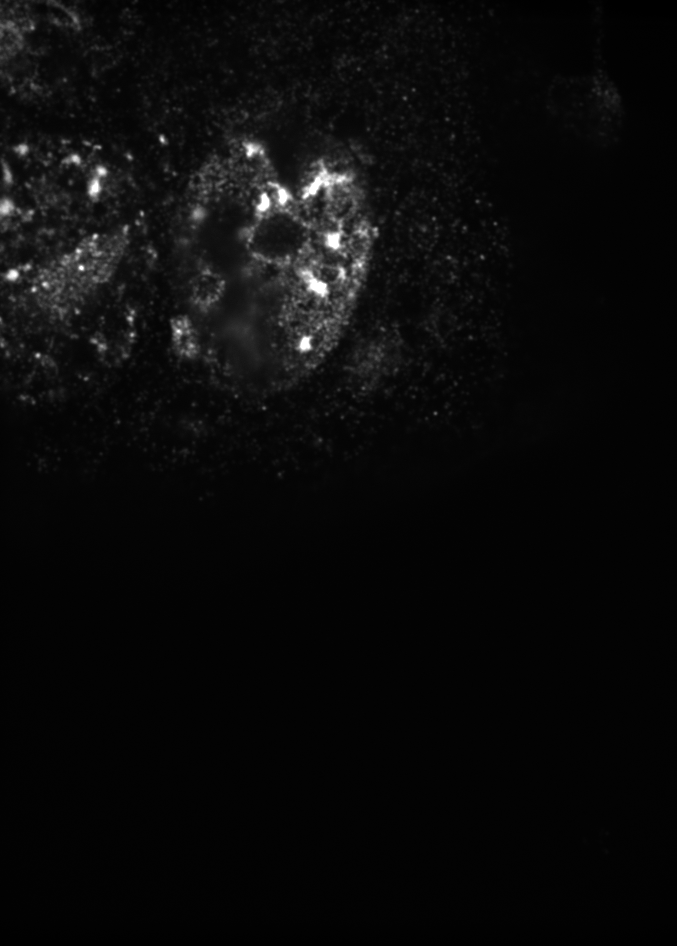

Supplement: Supplementary file 11 — Source data Fig. 4-2 [file 44318_2026_705_MOESM11_ESM.zip › Figure 4-2/F/MCF7_STARD3S209A_NT/20230310_MCF7STARD3S209A_NT_3_w2SPI 491 GFP.TIF]

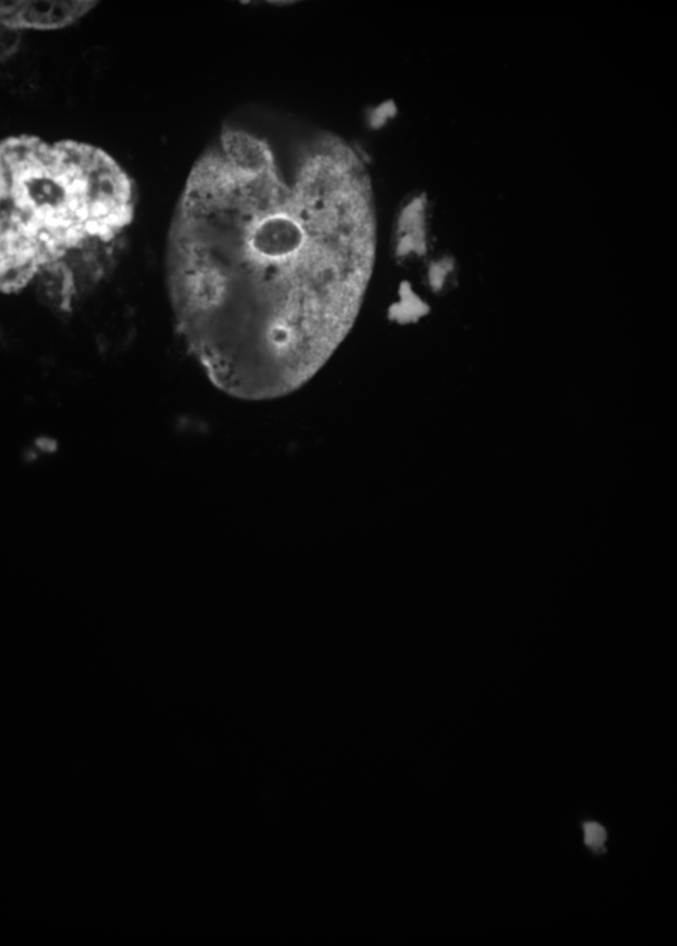

Supplement: Supplementary file 11 — Source data Fig. 4-2 [file 44318_2026_705_MOESM11_ESM.zip › Figure 4-2/F/MCF7_STARD3S209A_NT/20230310_MCF7STARD3S209A_NT_3_w3SPI 405 DAPI.TIF]

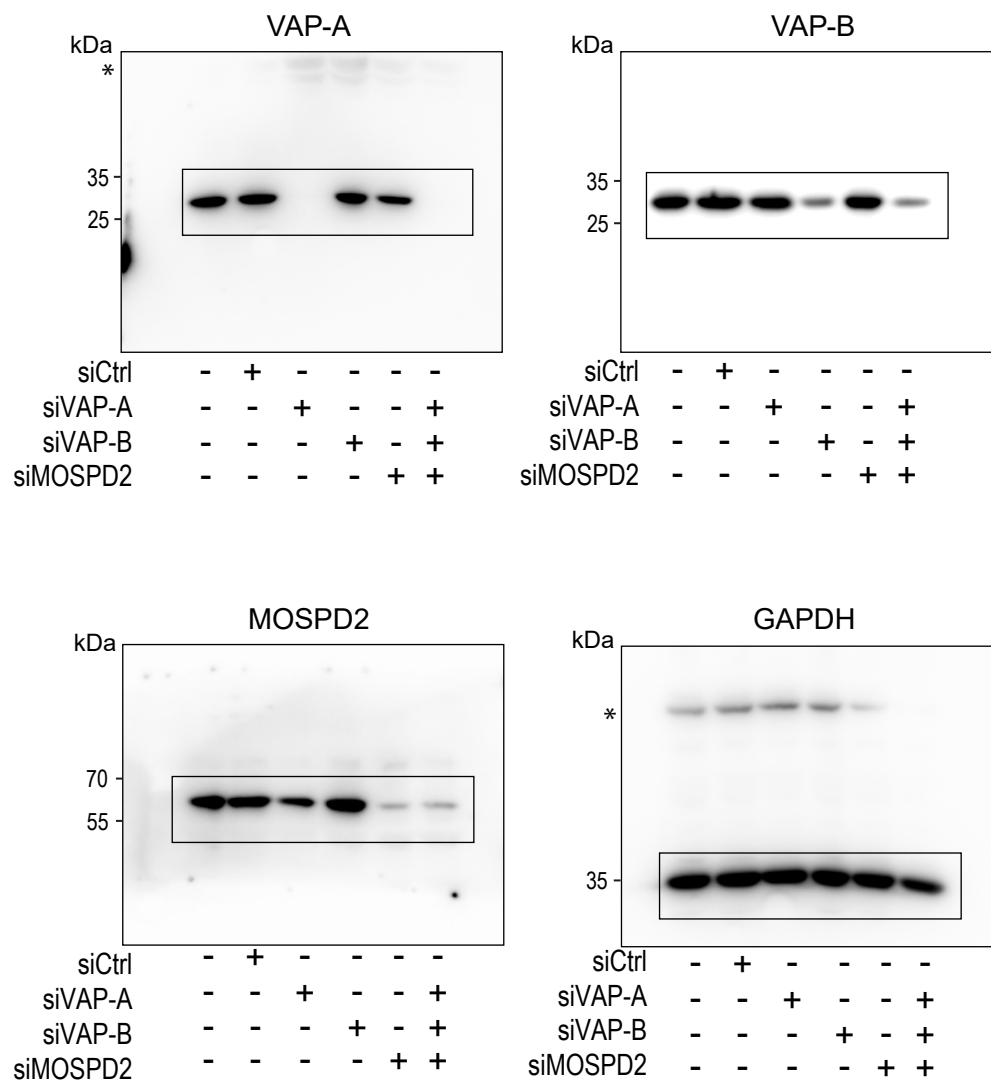

Supplement: Supplementary file 12 — Source data Fig. 5-1 [file 44318_2026_705_MOESM12_ESM.zip › Figure 5-1/A/MCF7_siVAPs_WB.pdf]

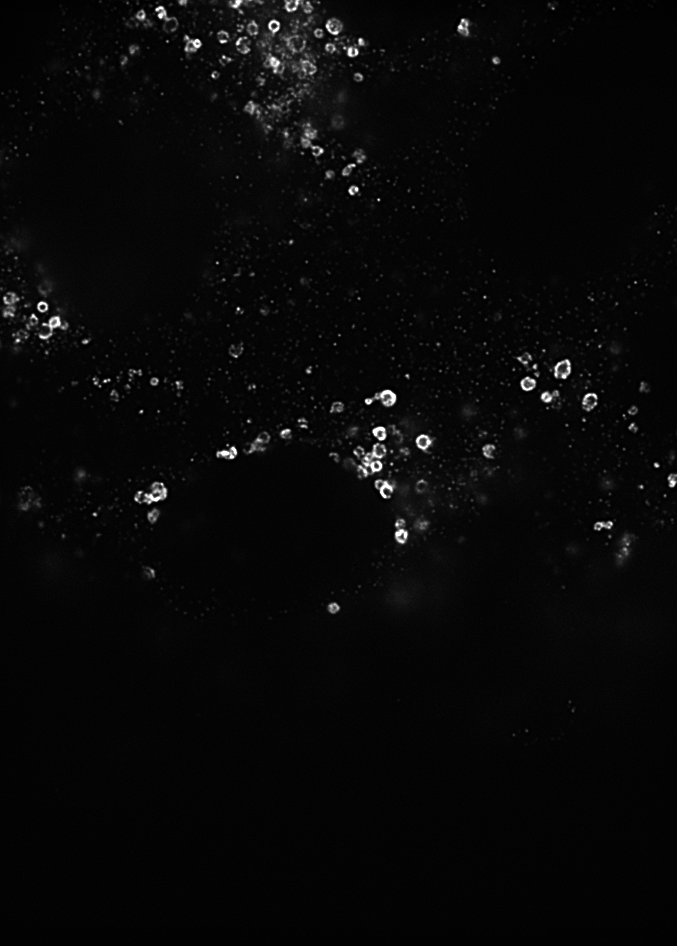

Supplement: Supplementary file 12 — Source data Fig. 5-1 [file 44318_2026_705_MOESM12_ESM.zip › Figure 5-1/B-1/MCF7STARD3/20240528_MCF7STARD3_4_SR_w1SPI 491 GFP.TIF]

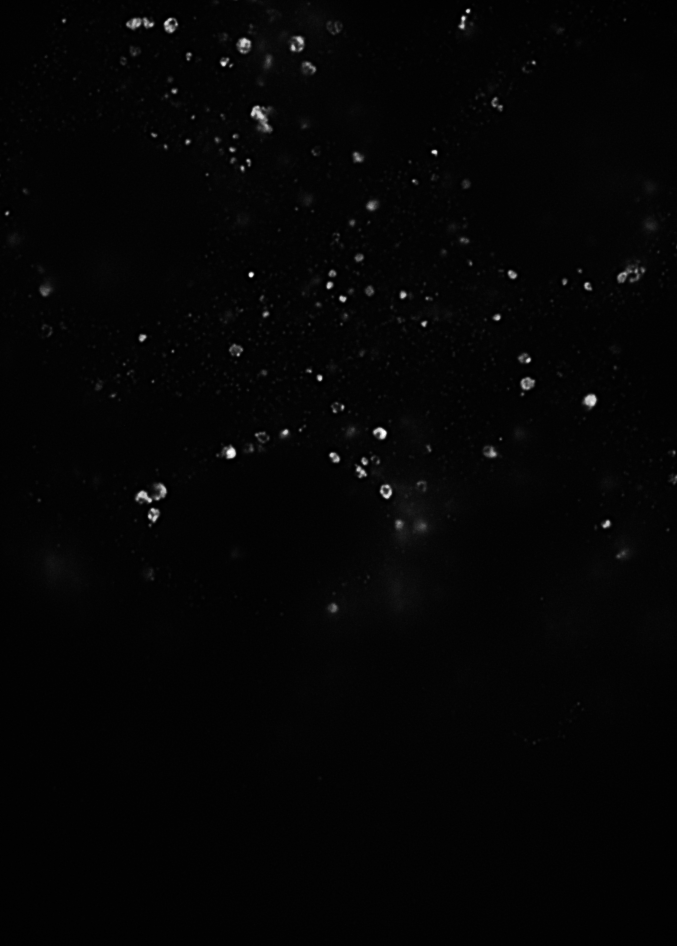

Supplement: Supplementary file 12 — Source data Fig. 5-1 [file 44318_2026_705_MOESM12_ESM.zip › Figure 5-1/B-1/MCF7STARD3/20240528_MCF7STARD3_4_SR_w2SPI 561 mCherry.TIF]

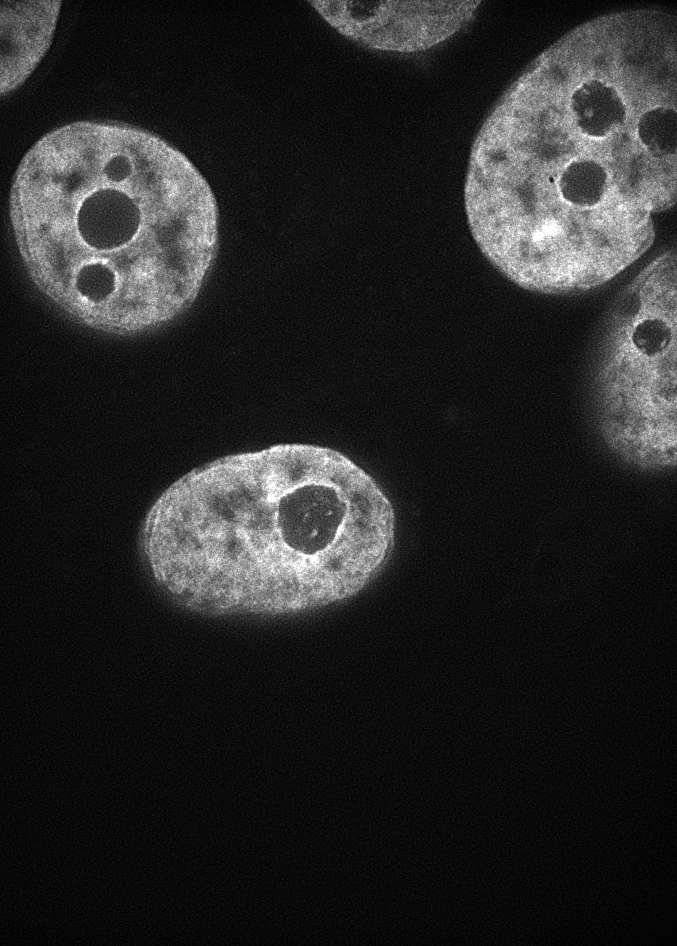

Supplement: Supplementary file 12 — Source data Fig. 5-1 [file 44318_2026_705_MOESM12_ESM.zip › Figure 5-1/B-1/MCF7STARD3/20240528_MCF7STARD3_4_SR_w3SPI 405 DAPI.TIF]

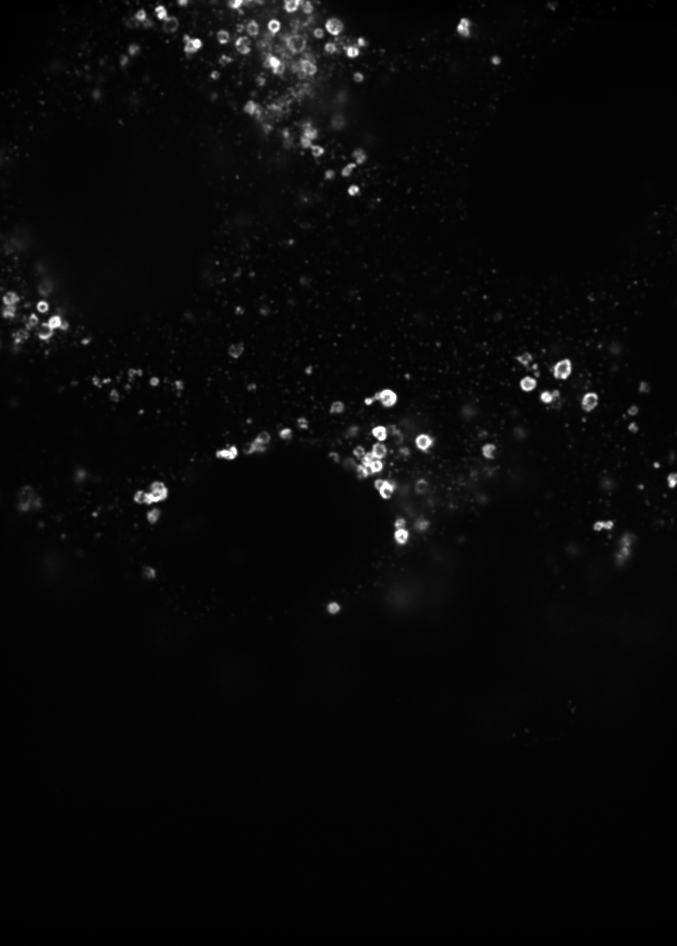

Supplement: Supplementary file 12 — Source data Fig. 5-1 [file 44318_2026_705_MOESM12_ESM.zip › Figure 5-1/B-1/MCF7STARD3/20240528_MCF7STARD3_4_w1SPI 491 GFP.TIF]

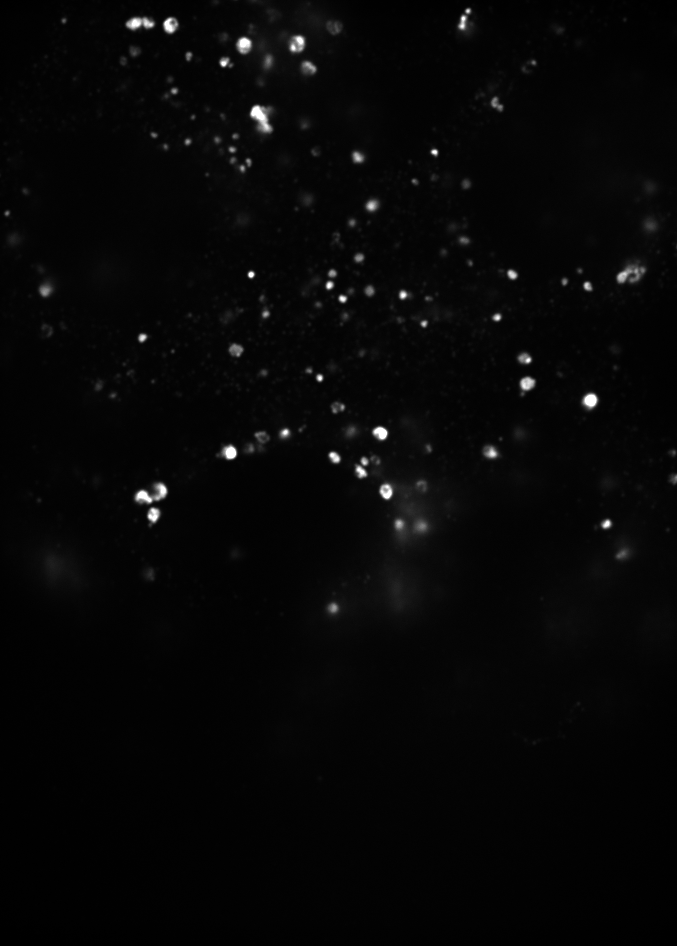

Supplement: Supplementary file 12 — Source data Fig. 5-1 [file 44318_2026_705_MOESM12_ESM.zip › Figure 5-1/B-1/MCF7STARD3/20240528_MCF7STARD3_4_w2SPI 561 mCherry.TIF]

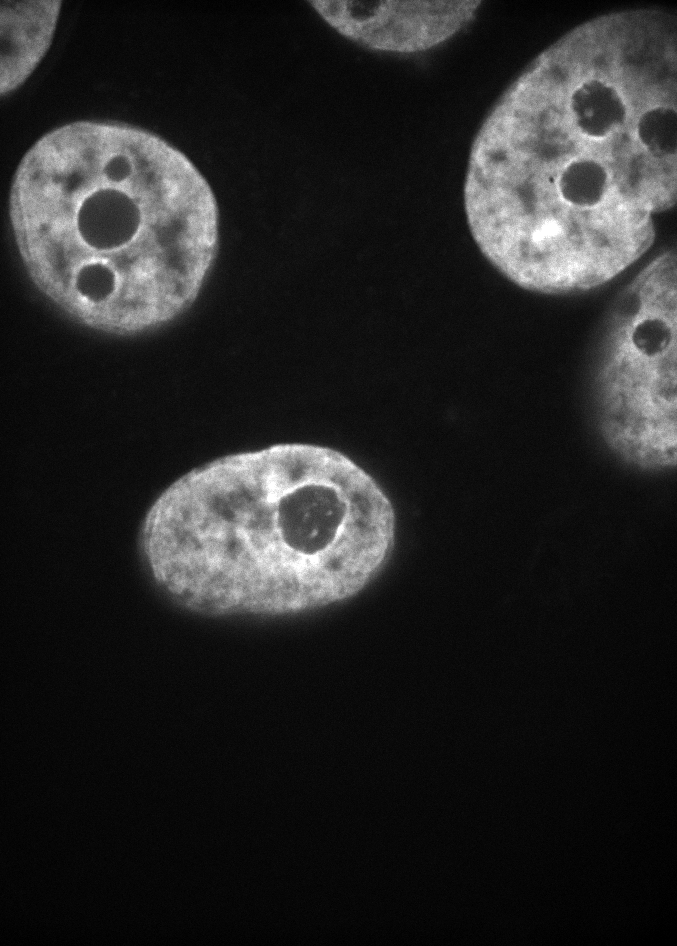

Supplement: Supplementary file 12 — Source data Fig. 5-1 [file 44318_2026_705_MOESM12_ESM.zip › Figure 5-1/B-1/MCF7STARD3/20240528_MCF7STARD3_4_w3SPI 405 DAPI.TIF]

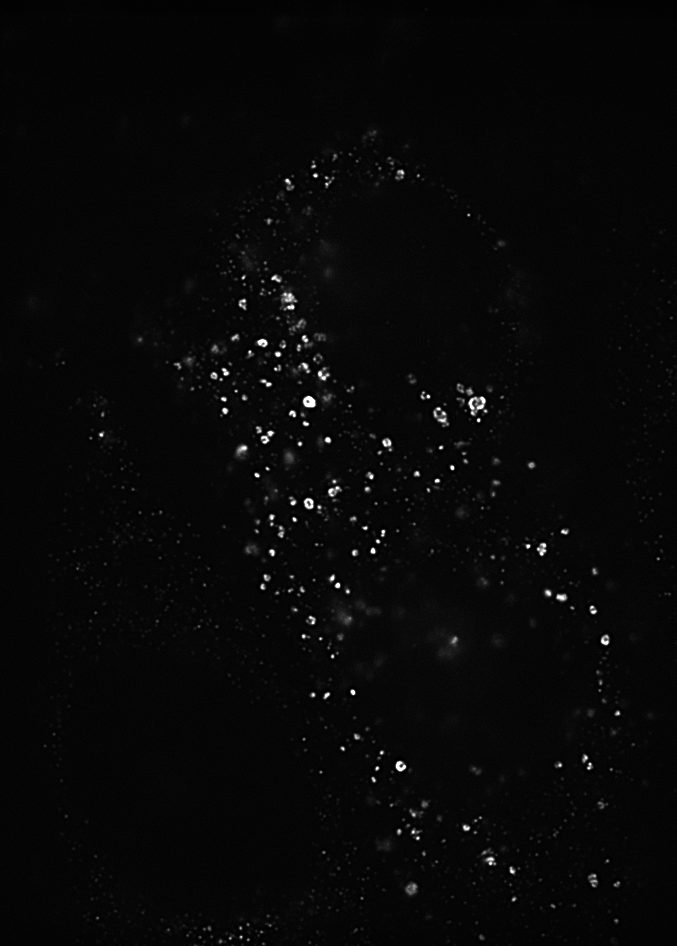

Supplement: Supplementary file 12 — Source data Fig. 5-1 [file 44318_2026_705_MOESM12_ESM.zip › Figure 5-1/B-1/MCF7STARD3_sictrl/20240528_MCF7STARD3_siCtrl_6_SR_w1SPI 491 GFP.TIF]

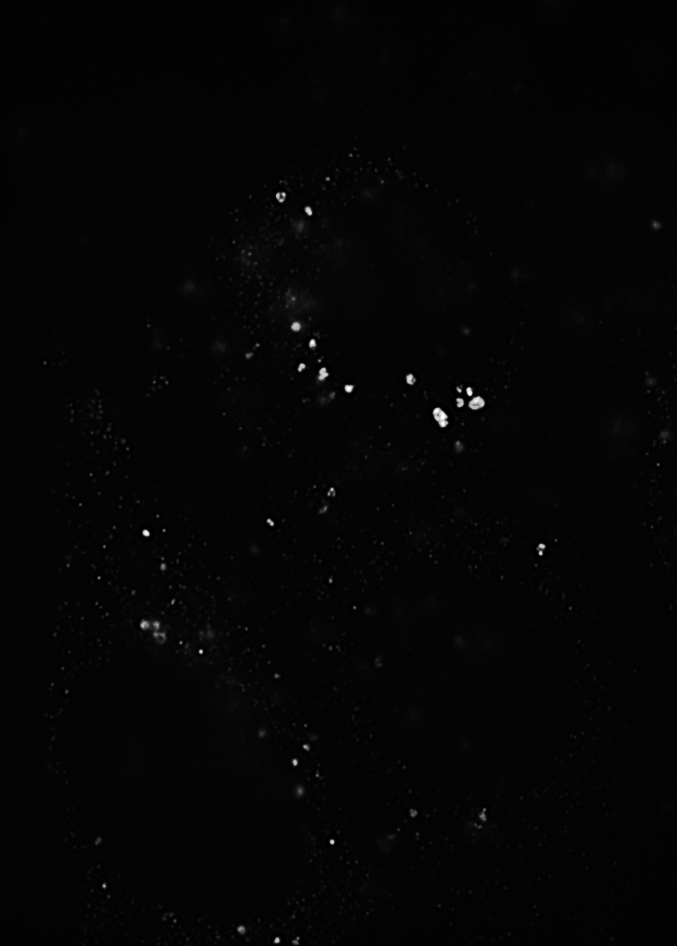

Supplement: Supplementary file 12 — Source data Fig. 5-1 [file 44318_2026_705_MOESM12_ESM.zip › Figure 5-1/B-1/MCF7STARD3_sictrl/20240528_MCF7STARD3_siCtrl_6_SR_w2SPI 561 mCherry.TIF]

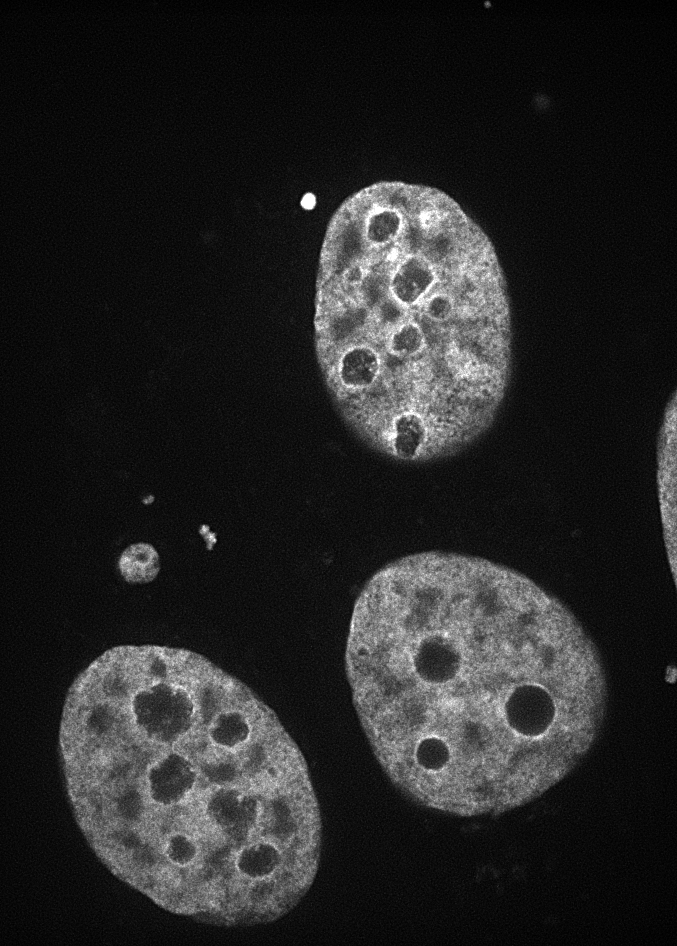

Supplement: Supplementary file 12 — Source data Fig. 5-1 [file 44318_2026_705_MOESM12_ESM.zip › Figure 5-1/B-1/MCF7STARD3_sictrl/20240528_MCF7STARD3_siCtrl_6_SR_w3SPI 405 DAPI.TIF]

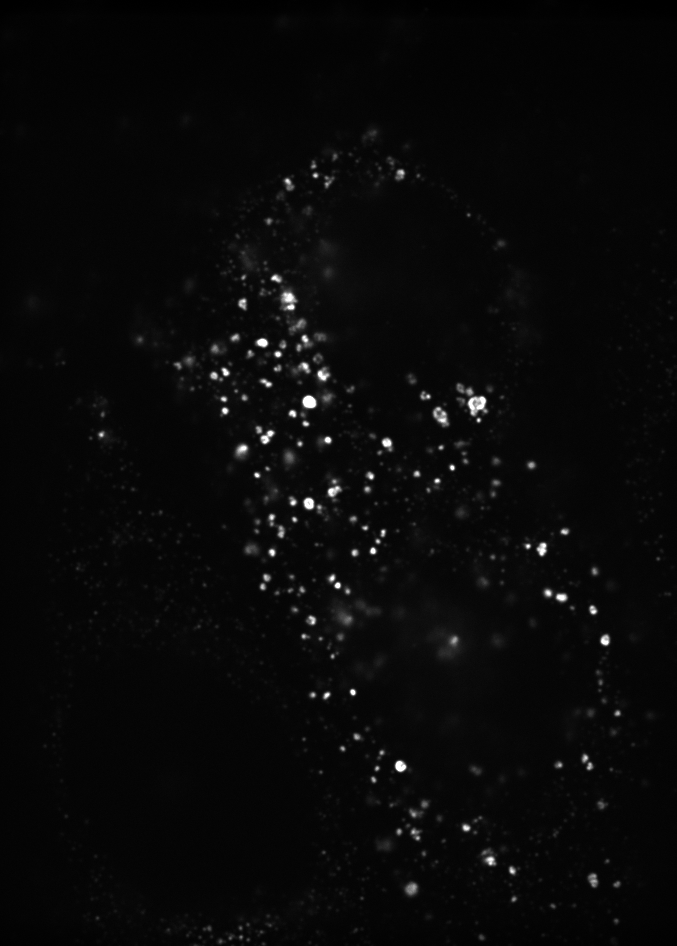

Supplement: Supplementary file 12 — Source data Fig. 5-1 [file 44318_2026_705_MOESM12_ESM.zip › Figure 5-1/B-1/MCF7STARD3_sictrl/20240528_MCF7STARD3_siCtrl_6_w1SPI 491 GFP.TIF]

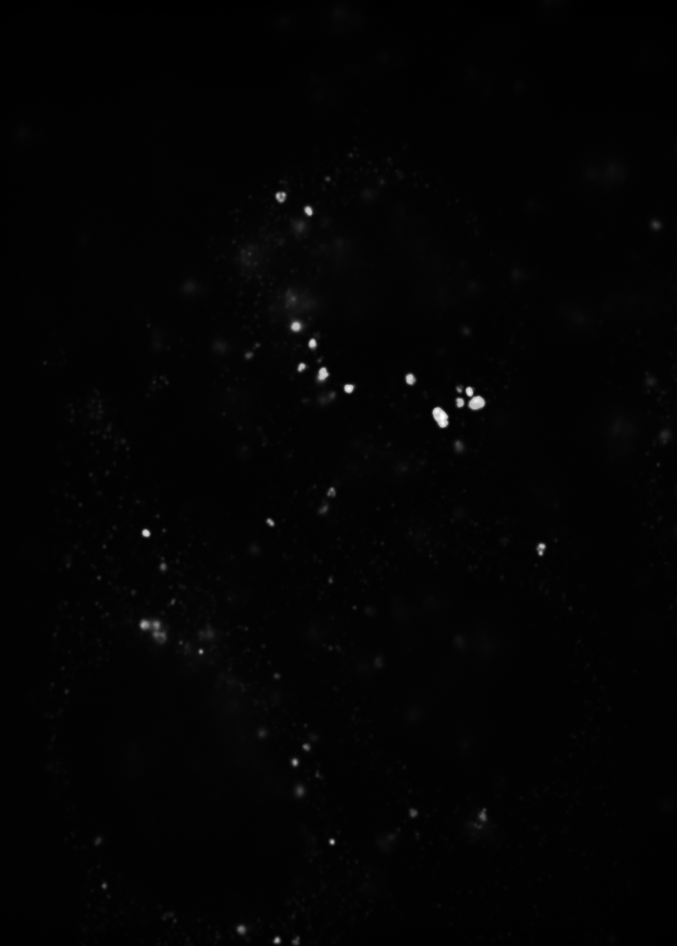

Supplement: Supplementary file 12 — Source data Fig. 5-1 [file 44318_2026_705_MOESM12_ESM.zip › Figure 5-1/B-1/MCF7STARD3_sictrl/20240528_MCF7STARD3_siCtrl_6_w2SPI 561 mCherry.TIF]

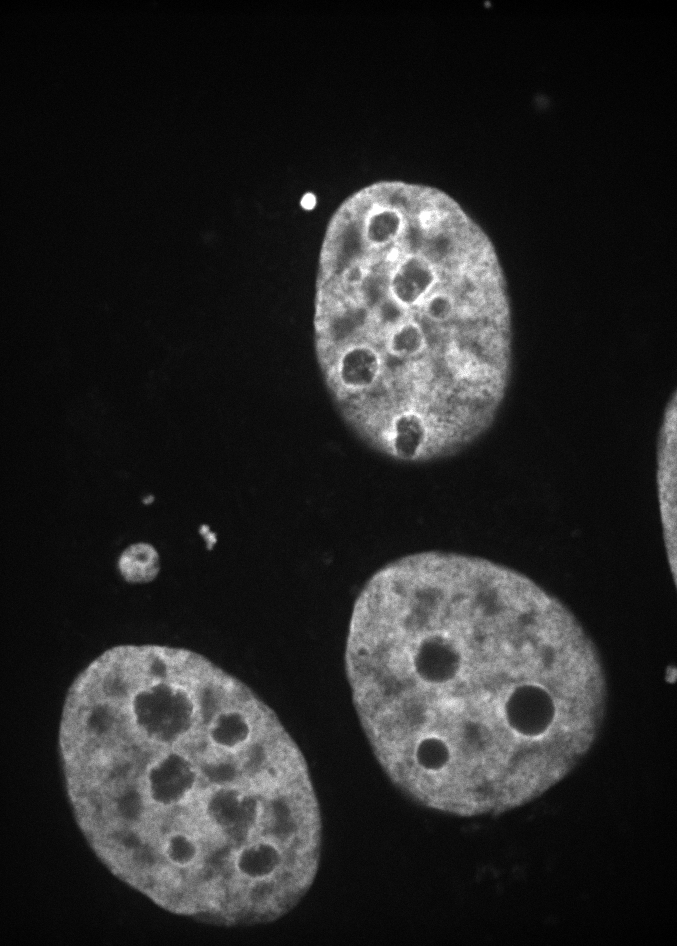

Supplement: Supplementary file 12 — Source data Fig. 5-1 [file 44318_2026_705_MOESM12_ESM.zip › Figure 5-1/B-1/MCF7STARD3_sictrl/20240528_MCF7STARD3_siCtrl_6_w3SPI 405 DAPI.TIF]

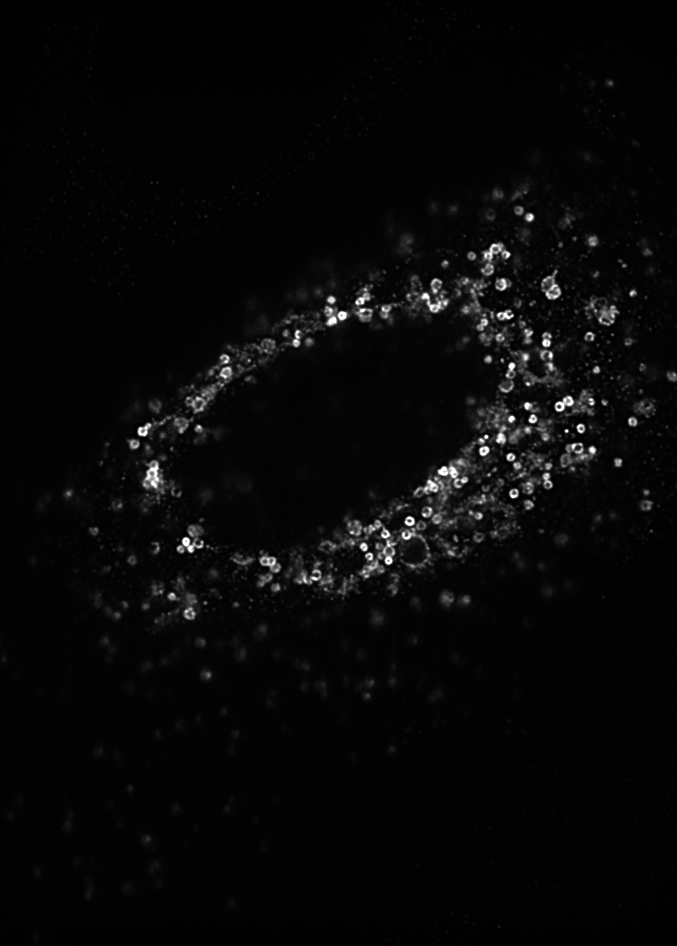

Supplement: Supplementary file 12 — Source data Fig. 5-1 [file 44318_2026_705_MOESM12_ESM.zip › Figure 5-1/B-1/MCF7STARD3_siMOSPD2/20240527_MCF7STARD3_siM2_6_SR_w1SPI 491 GFP.TIF]

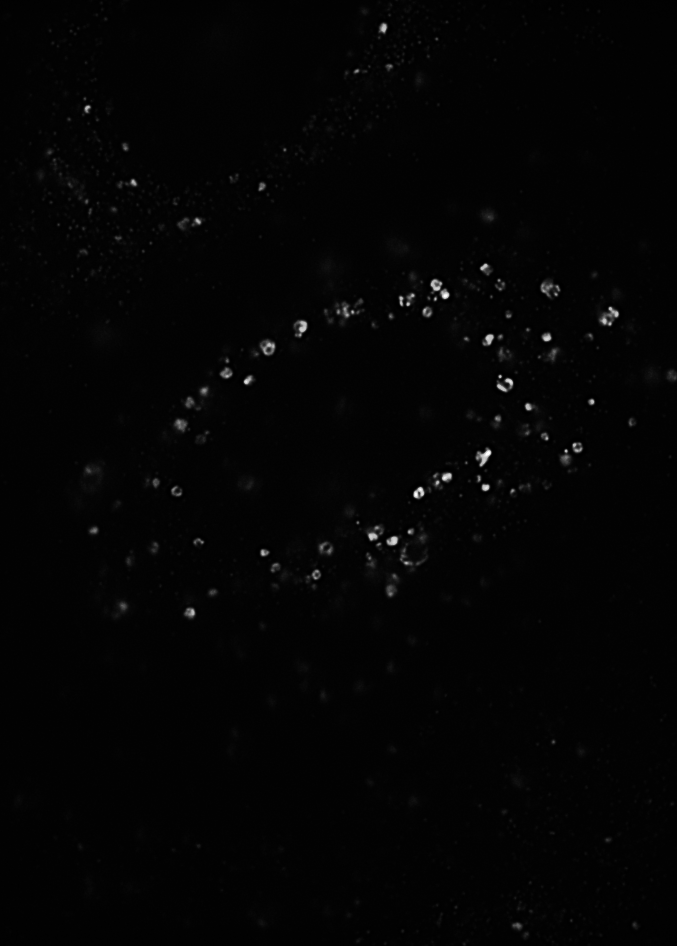

Supplement: Supplementary file 12 — Source data Fig. 5-1 [file 44318_2026_705_MOESM12_ESM.zip › Figure 5-1/B-1/MCF7STARD3_siMOSPD2/20240527_MCF7STARD3_siM2_6_SR_w2SPI 561 mCherry.TIF]

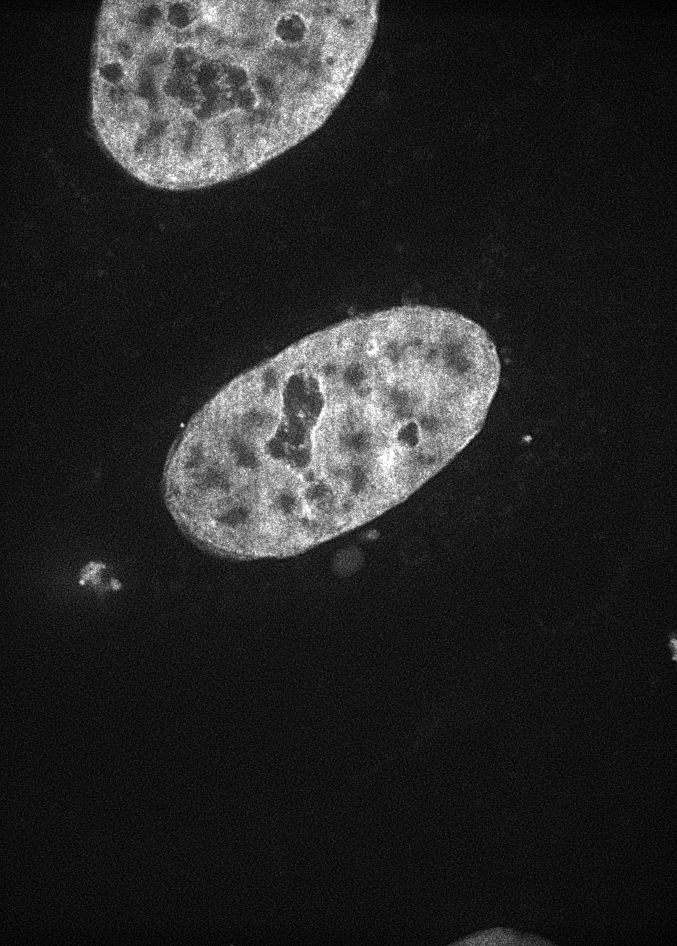

Supplement: Supplementary file 12 — Source data Fig. 5-1 [file 44318_2026_705_MOESM12_ESM.zip › Figure 5-1/B-1/MCF7STARD3_siMOSPD2/20240527_MCF7STARD3_siM2_6_SR_w3SPI 405 DAPI.TIF]

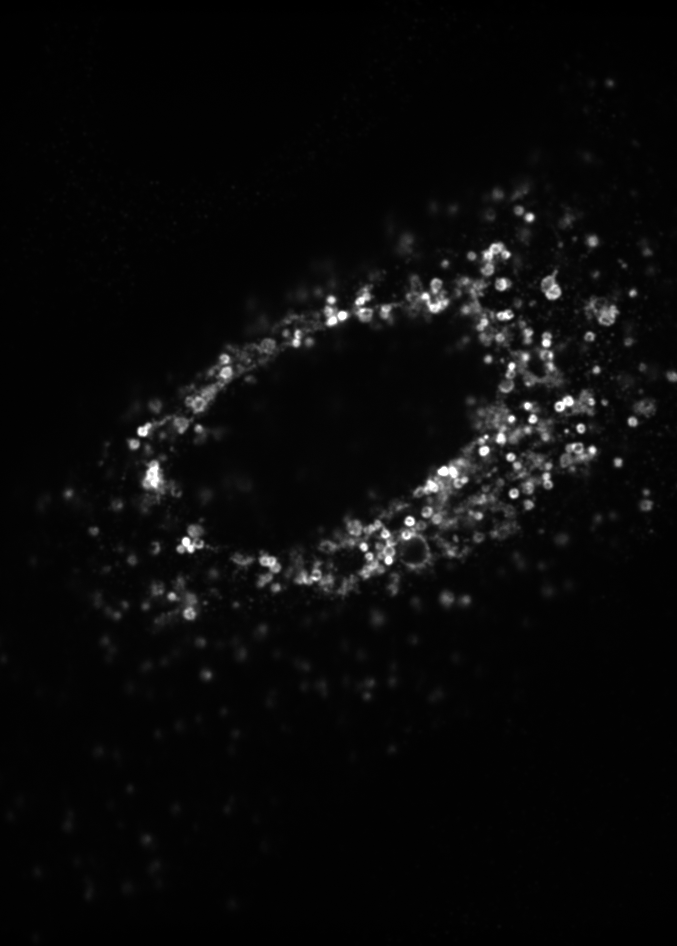

Supplement: Supplementary file 12 — Source data Fig. 5-1 [file 44318_2026_705_MOESM12_ESM.zip › Figure 5-1/B-1/MCF7STARD3_siMOSPD2/20240527_MCF7STARD3_siM2_6_w1SPI 491 GFP.TIF]

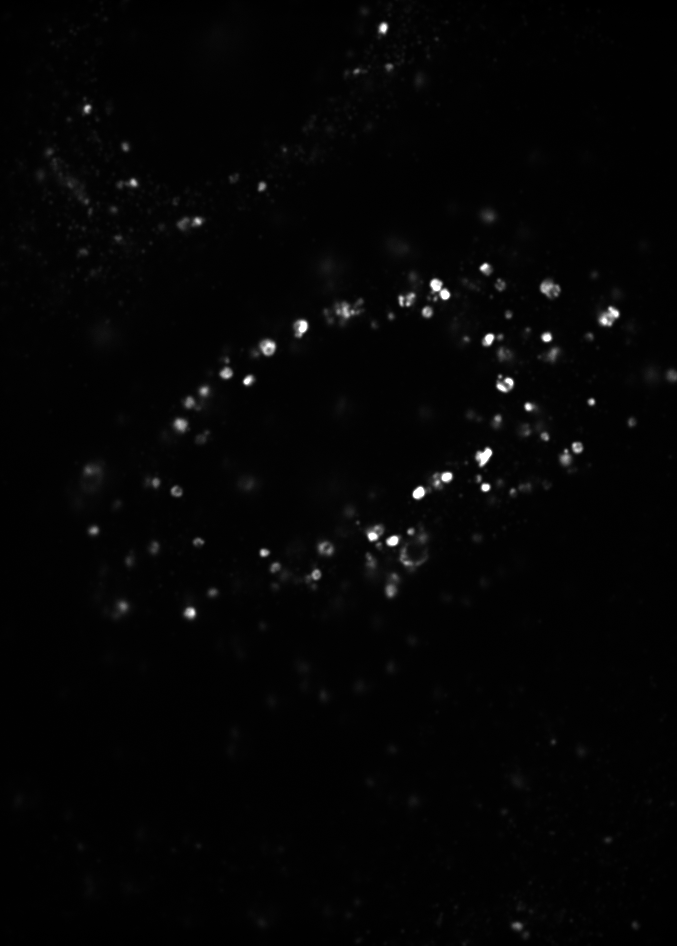

Supplement: Supplementary file 12 — Source data Fig. 5-1 [file 44318_2026_705_MOESM12_ESM.zip › Figure 5-1/B-1/MCF7STARD3_siMOSPD2/20240527_MCF7STARD3_siM2_6_w2SPI 561 mCherry.TIF]

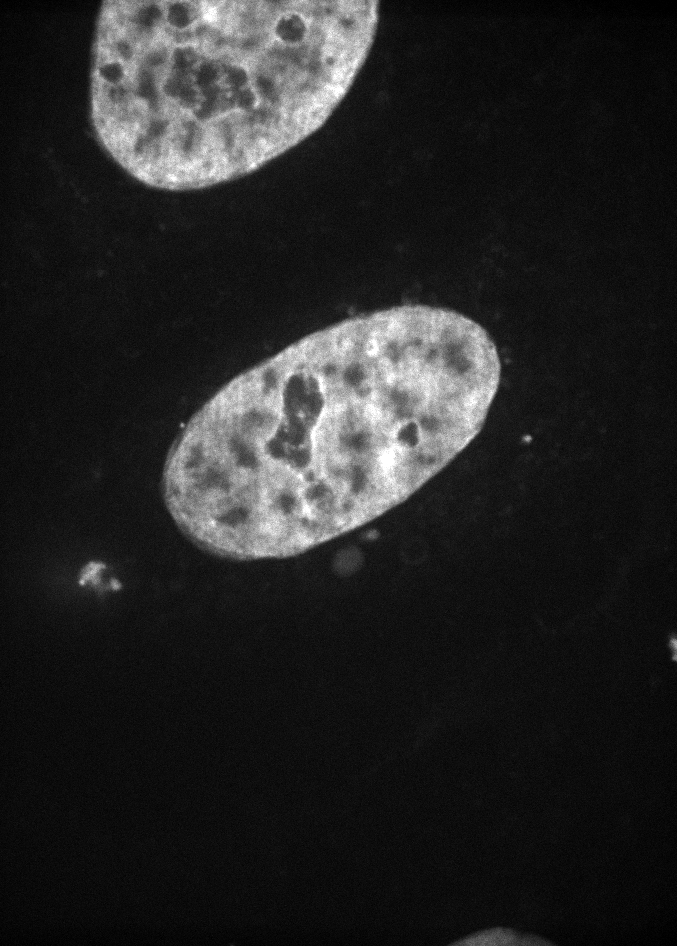

Supplement: Supplementary file 12 — Source data Fig. 5-1 [file 44318_2026_705_MOESM12_ESM.zip › Figure 5-1/B-1/MCF7STARD3_siMOSPD2/20240527_MCF7STARD3_siM2_6_w3SPI 405 DAPI.TIF]

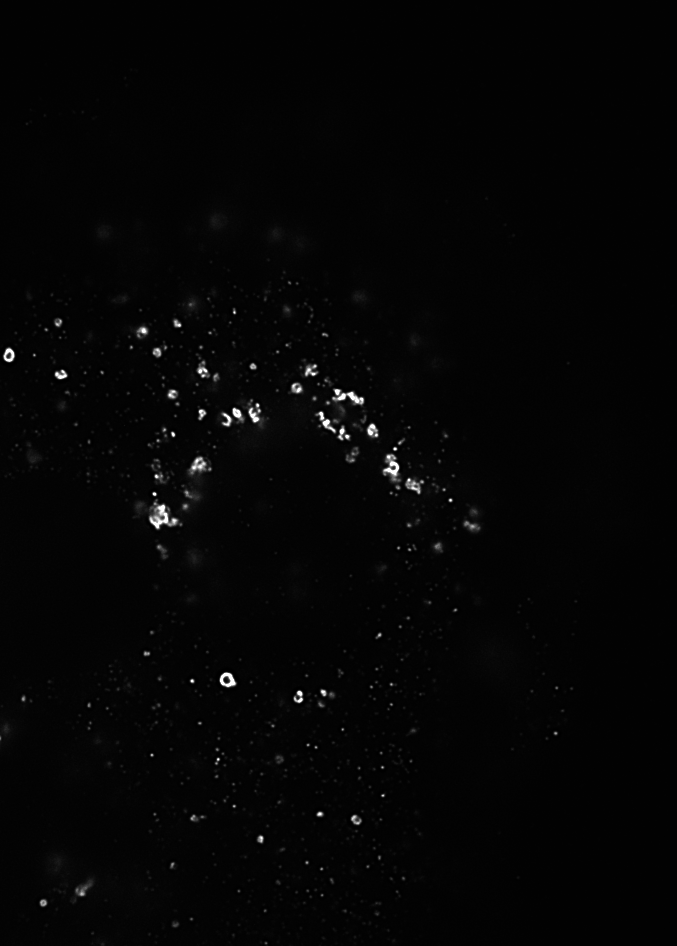

Supplement: Supplementary file 13 — Source data Fig. 5-2 [file 44318_2026_705_MOESM13_ESM.zip › Figure 5-2/B-2/MCF7STARD3_siVAPA/20240528_MCF7STARD3_siVAPA_8_SR_w1SPI 491 GFP.TIF]

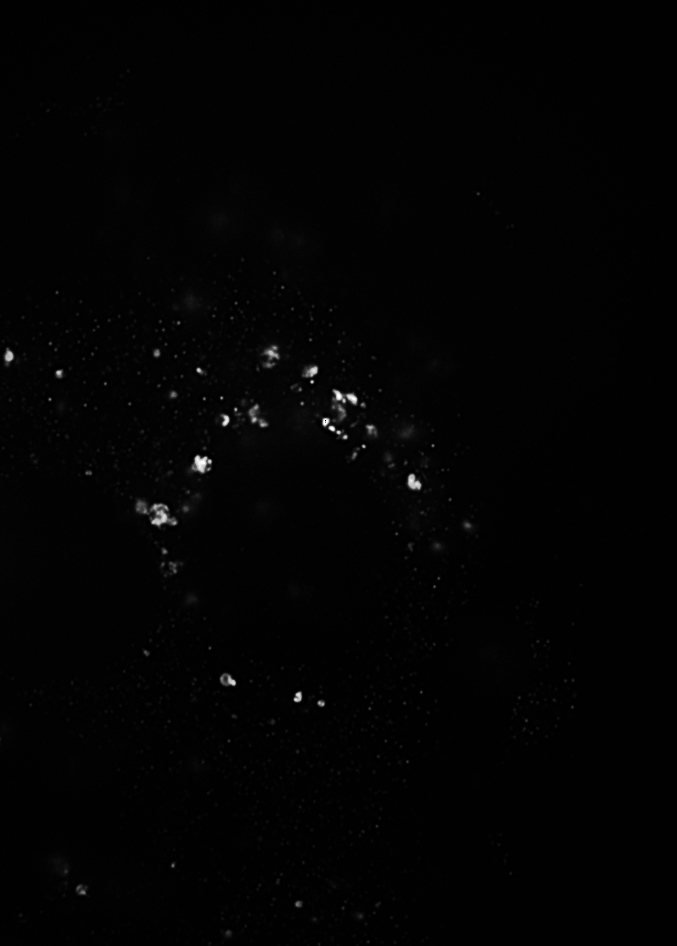

Supplement: Supplementary file 13 — Source data Fig. 5-2 [file 44318_2026_705_MOESM13_ESM.zip › Figure 5-2/B-2/MCF7STARD3_siVAPA/20240528_MCF7STARD3_siVAPA_8_SR_w2SPI 561 mCherry.TIF]

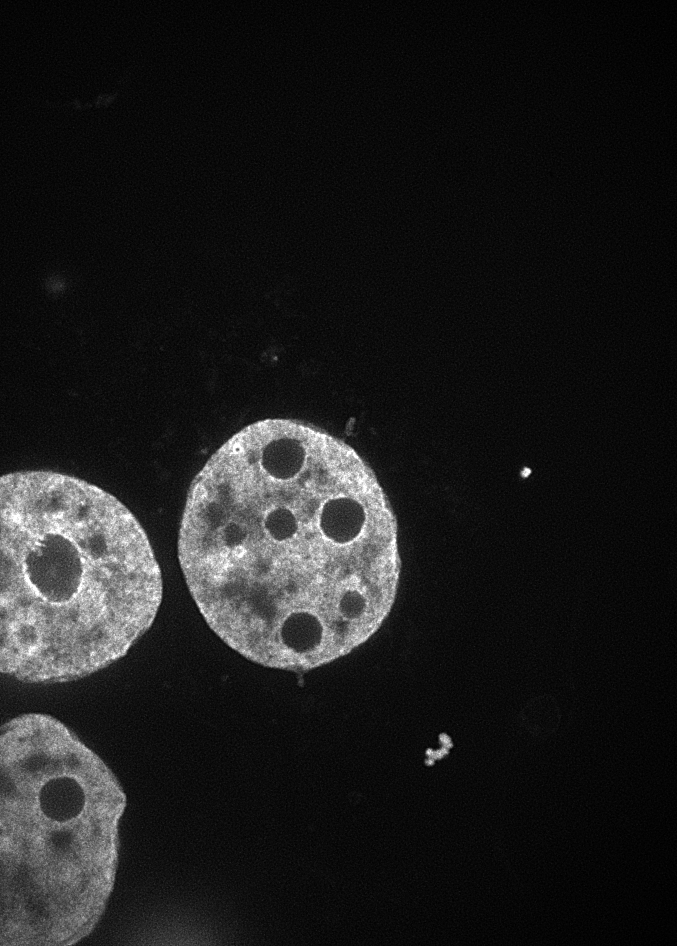

Supplement: Supplementary file 13 — Source data Fig. 5-2 [file 44318_2026_705_MOESM13_ESM.zip › Figure 5-2/B-2/MCF7STARD3_siVAPA/20240528_MCF7STARD3_siVAPA_8_SR_w3SPI 405 DAPI.TIF]

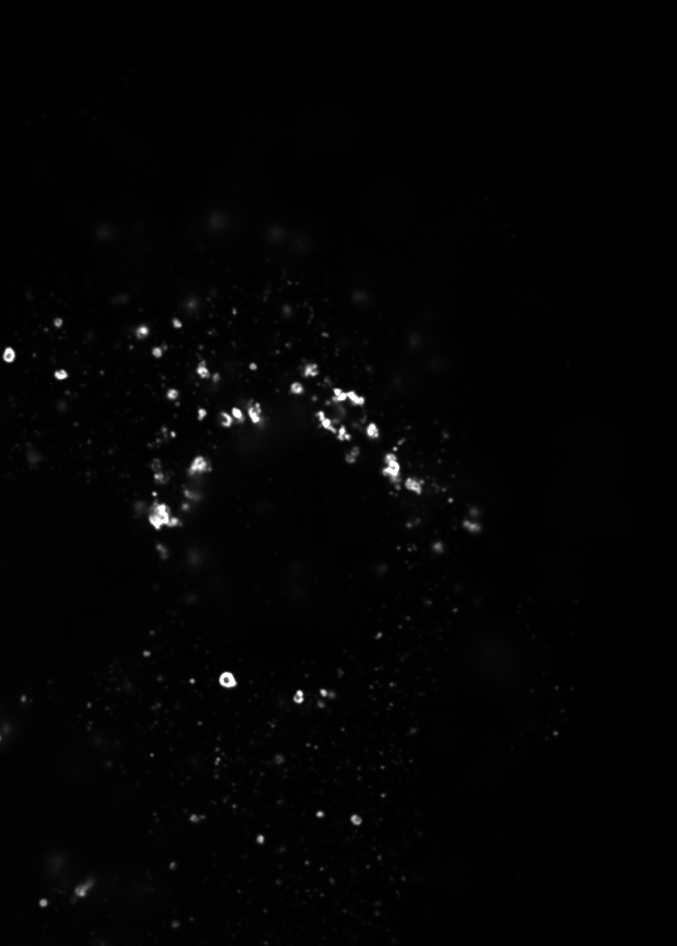

Supplement: Supplementary file 13 — Source data Fig. 5-2 [file 44318_2026_705_MOESM13_ESM.zip › Figure 5-2/B-2/MCF7STARD3_siVAPA/20240528_MCF7STARD3_siVAPA_8_w1SPI 491 GFP.TIF]

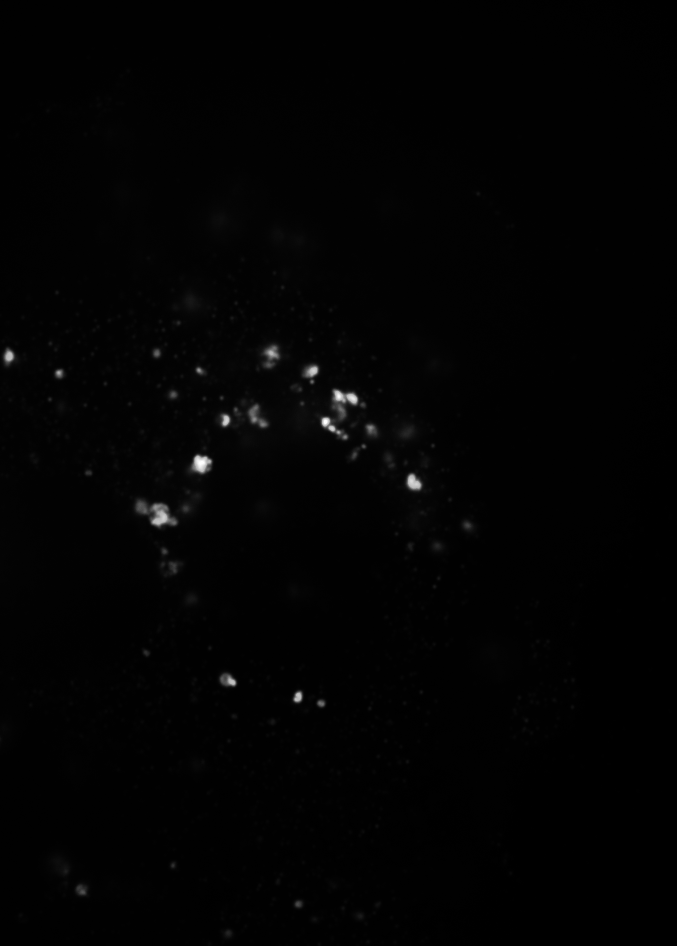

Supplement: Supplementary file 13 — Source data Fig. 5-2 [file 44318_2026_705_MOESM13_ESM.zip › Figure 5-2/B-2/MCF7STARD3_siVAPA/20240528_MCF7STARD3_siVAPA_8_w2SPI 561 mCherry.TIF]

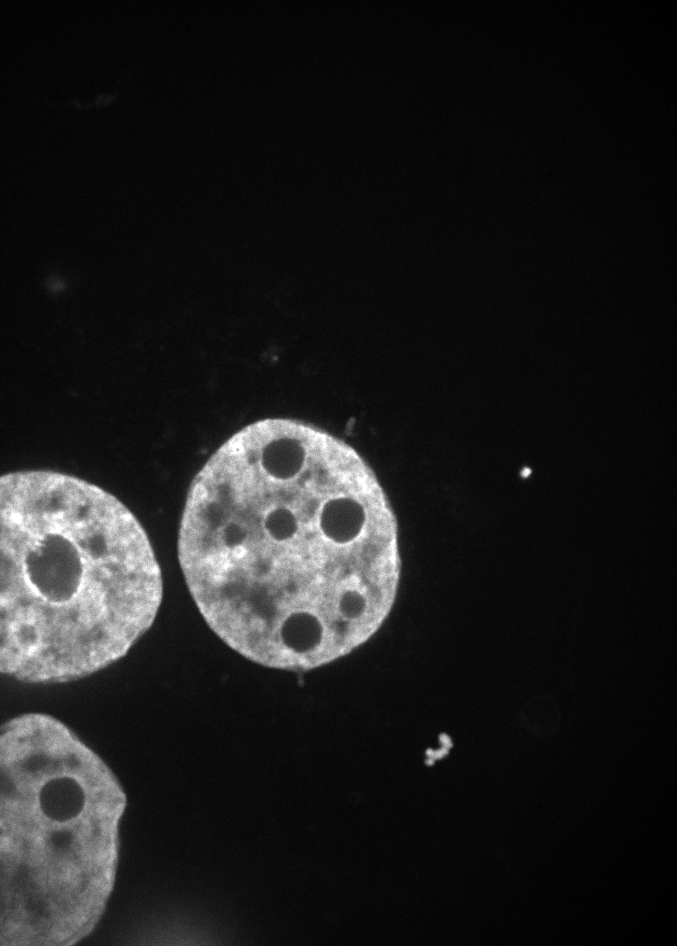

Supplement: Supplementary file 13 — Source data Fig. 5-2 [file 44318_2026_705_MOESM13_ESM.zip › Figure 5-2/B-2/MCF7STARD3_siVAPA/20240528_MCF7STARD3_siVAPA_8_w3SPI 405 DAPI.TIF]

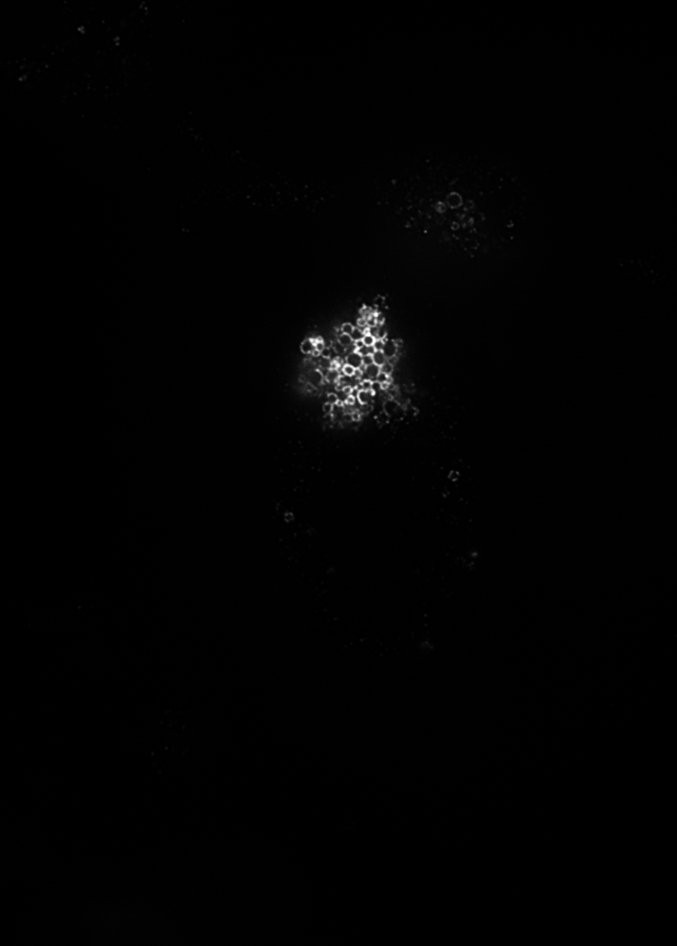

Supplement: Supplementary file 13 — Source data Fig. 5-2 [file 44318_2026_705_MOESM13_ESM.zip › Figure 5-2/B-2/MCF7STARD3_siVAPAVAPBMOSPD2/20240527_MCF7STARD3_si3VAPs_8_SR_w1SPI 491 GFP.TIF]

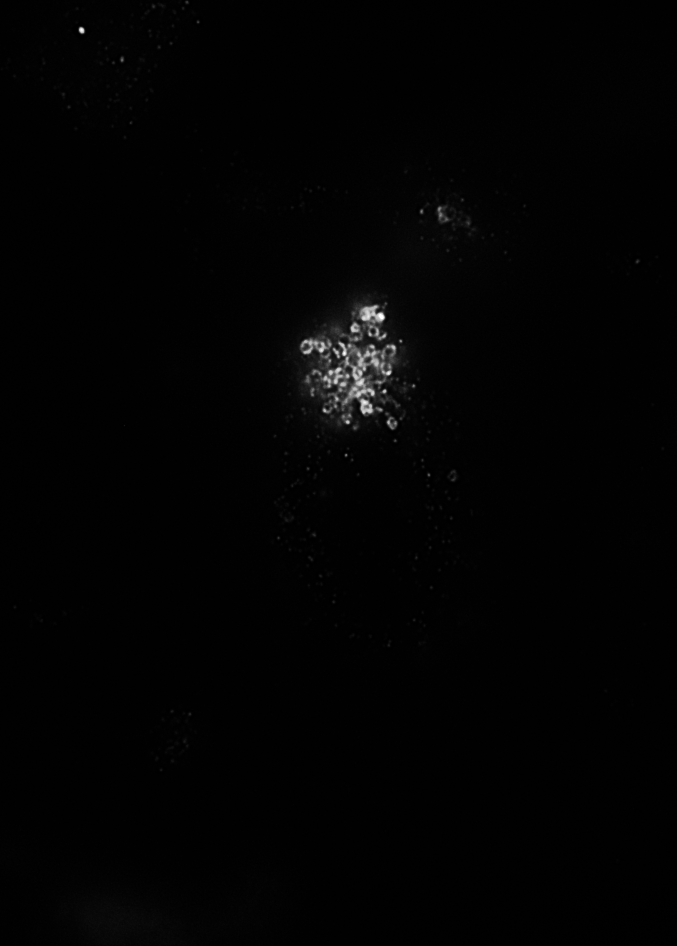

Supplement: Supplementary file 13 — Source data Fig. 5-2 [file 44318_2026_705_MOESM13_ESM.zip › Figure 5-2/B-2/MCF7STARD3_siVAPAVAPBMOSPD2/20240527_MCF7STARD3_si3VAPs_8_SR_w2SPI 561 mCherry.TIF]

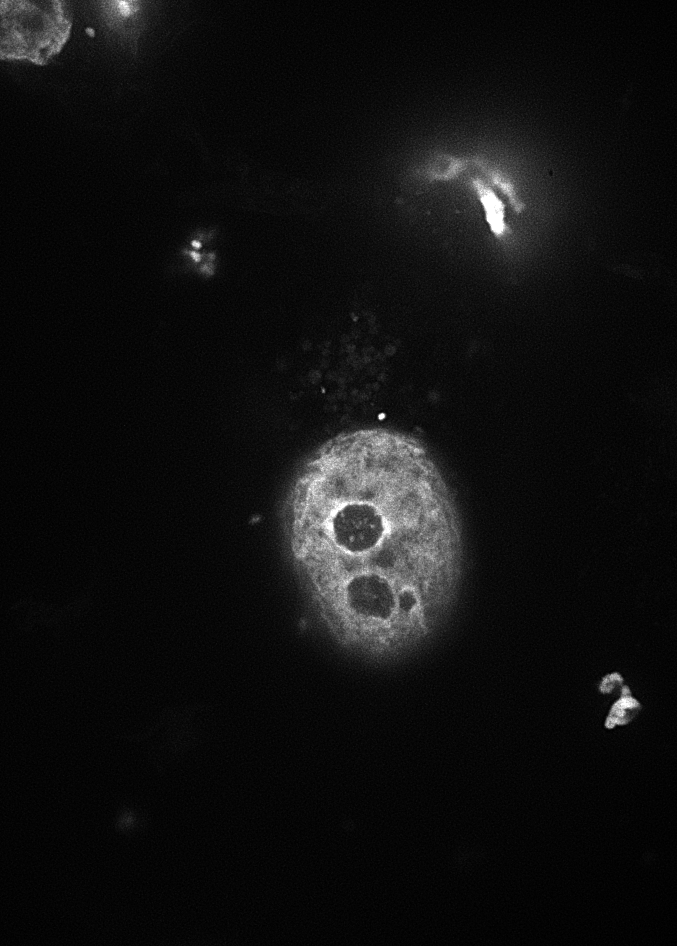

Supplement: Supplementary file 13 — Source data Fig. 5-2 [file 44318_2026_705_MOESM13_ESM.zip › Figure 5-2/B-2/MCF7STARD3_siVAPAVAPBMOSPD2/20240527_MCF7STARD3_si3VAPs_8_SR_w3SPI 405 DAPI.TIF]

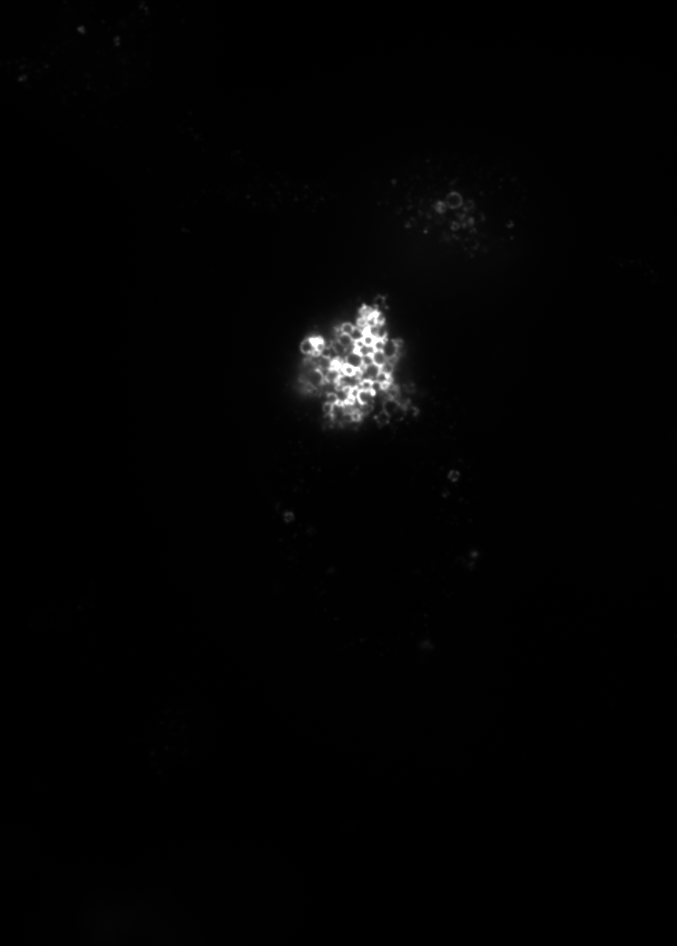

Supplement: Supplementary file 13 — Source data Fig. 5-2 [file 44318_2026_705_MOESM13_ESM.zip › Figure 5-2/B-2/MCF7STARD3_siVAPAVAPBMOSPD2/20240527_MCF7STARD3_si3VAPs_8_w1SPI 491 GFP.TIF]

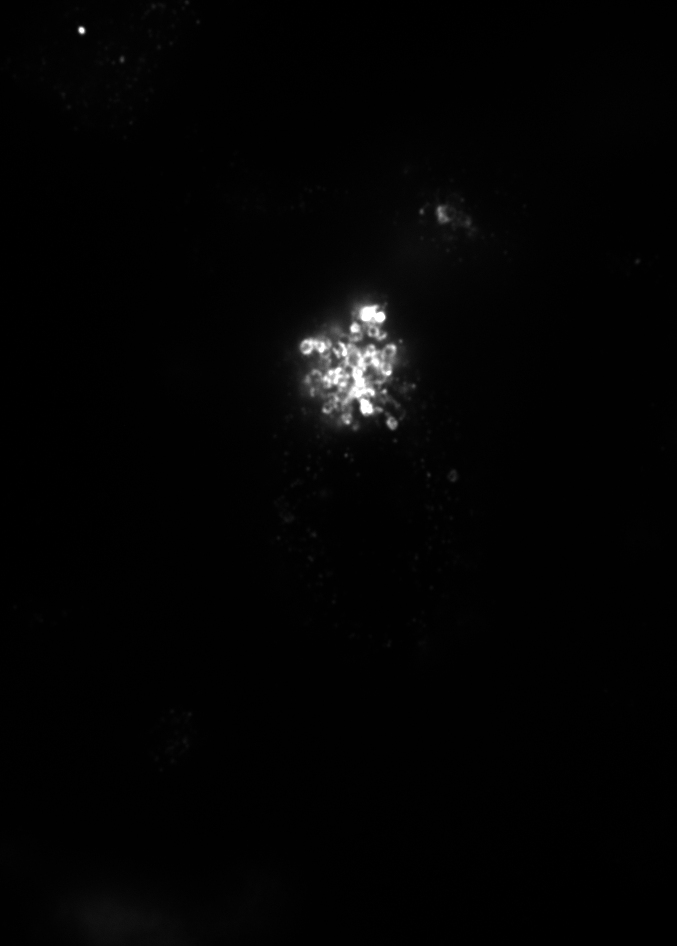

Supplement: Supplementary file 13 — Source data Fig. 5-2 [file 44318_2026_705_MOESM13_ESM.zip › Figure 5-2/B-2/MCF7STARD3_siVAPAVAPBMOSPD2/20240527_MCF7STARD3_si3VAPs_8_w2SPI 561 mCherry.TIF]

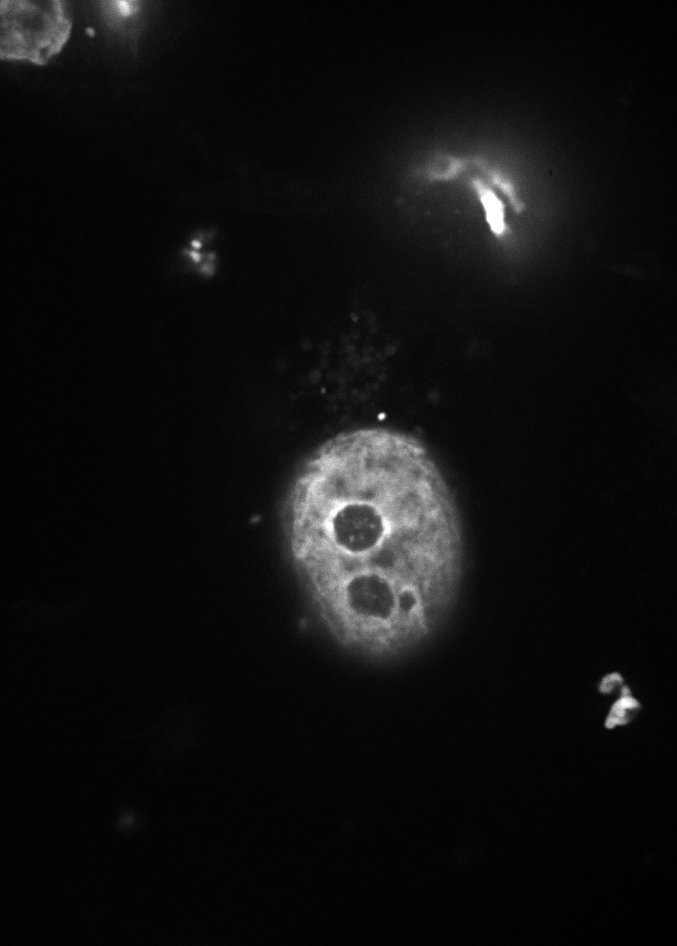

Supplement: Supplementary file 13 — Source data Fig. 5-2 [file 44318_2026_705_MOESM13_ESM.zip › Figure 5-2/B-2/MCF7STARD3_siVAPAVAPBMOSPD2/20240527_MCF7STARD3_si3VAPs_8_w3SPI 405 DAPI.TIF]

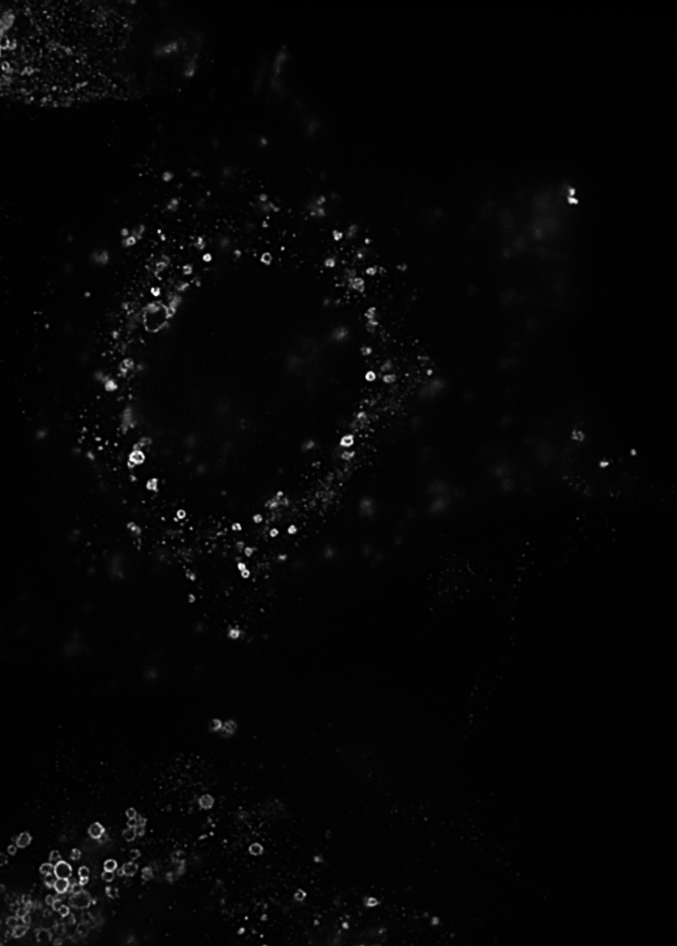

Supplement: Supplementary file 13 — Source data Fig. 5-2 [file 44318_2026_705_MOESM13_ESM.zip › Figure 5-2/B-2/MCF7STARD3_siVAPB/20240527_MCF7STARD3_siVAPB_18_SR_w1SPI 491 GFP.TIF]

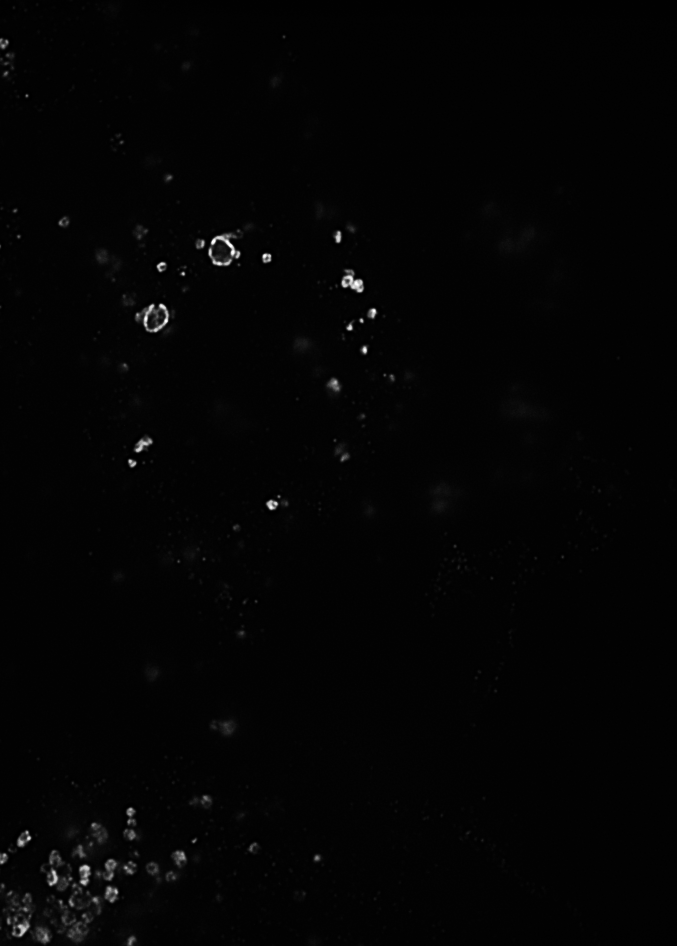

Supplement: Supplementary file 13 — Source data Fig. 5-2 [file 44318_2026_705_MOESM13_ESM.zip › Figure 5-2/B-2/MCF7STARD3_siVAPB/20240527_MCF7STARD3_siVAPB_18_SR_w2SPI 561 mCherry.TIF]

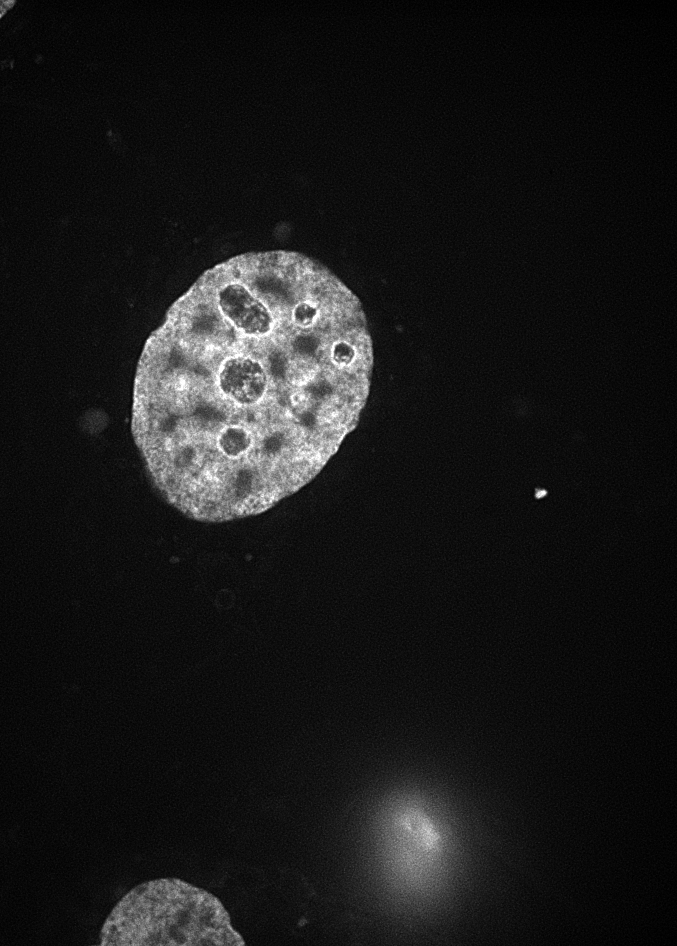

Supplement: Supplementary file 13 — Source data Fig. 5-2 [file 44318_2026_705_MOESM13_ESM.zip › Figure 5-2/B-2/MCF7STARD3_siVAPB/20240527_MCF7STARD3_siVAPB_18_SR_w3SPI 405 DAPI.TIF]

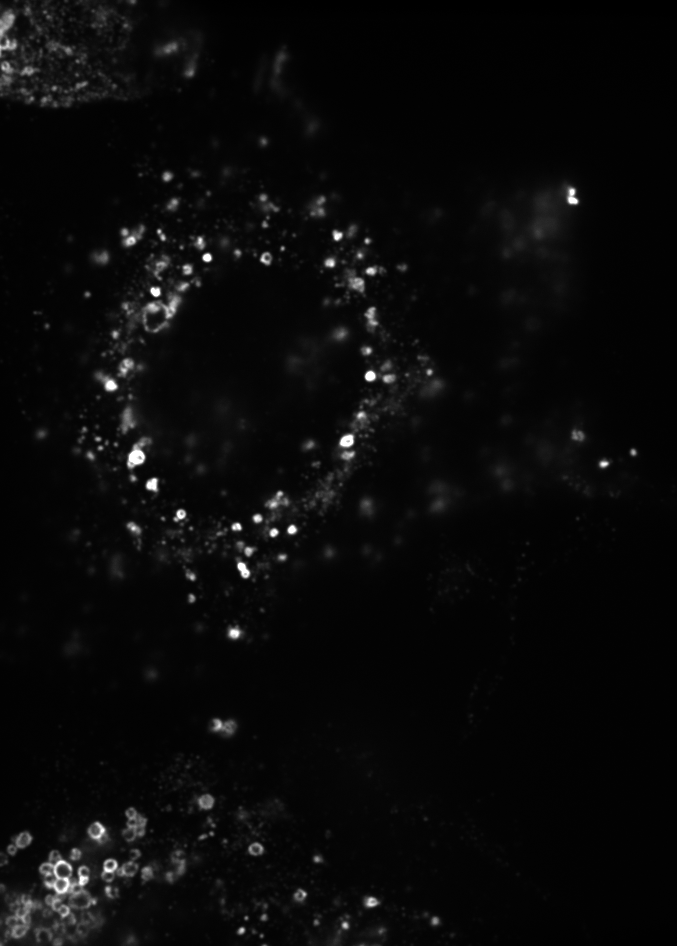

Supplement: Supplementary file 13 — Source data Fig. 5-2 [file 44318_2026_705_MOESM13_ESM.zip › Figure 5-2/B-2/MCF7STARD3_siVAPB/20240527_MCF7STARD3_siVAPB_18_w1SPI 491 GFP.TIF]

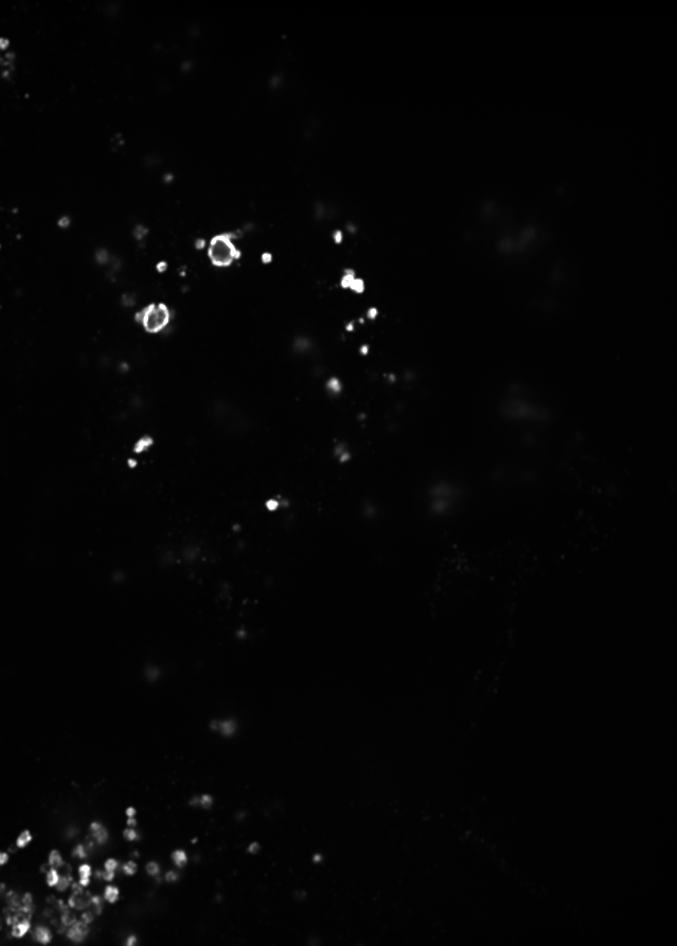

Supplement: Supplementary file 13 — Source data Fig. 5-2 [file 44318_2026_705_MOESM13_ESM.zip › Figure 5-2/B-2/MCF7STARD3_siVAPB/20240527_MCF7STARD3_siVAPB_18_w2SPI 561 mCherry.TIF]

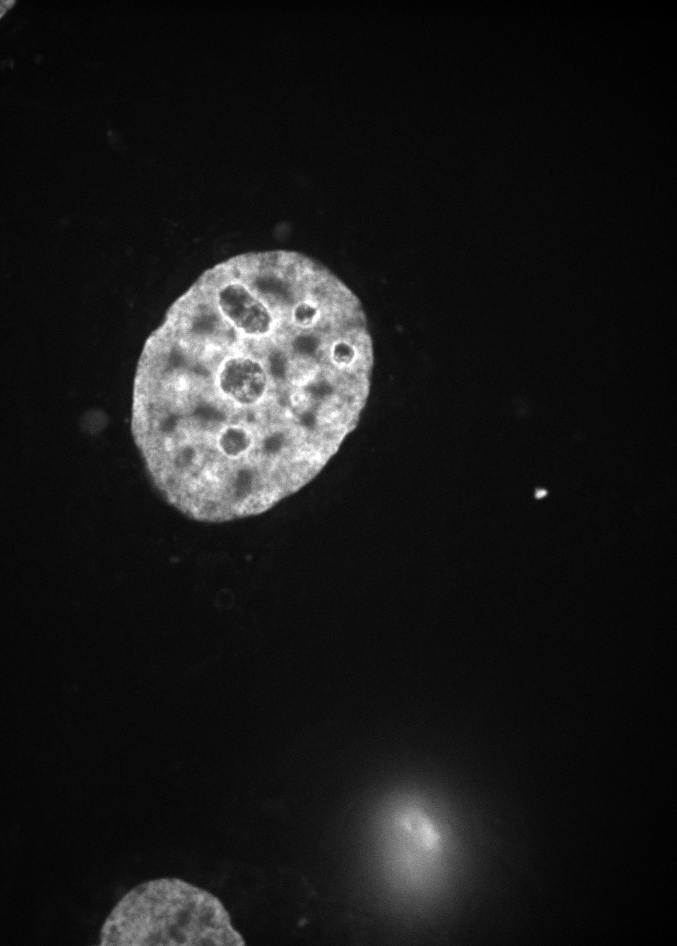

Supplement: Supplementary file 13 — Source data Fig. 5-2 [file 44318_2026_705_MOESM13_ESM.zip › Figure 5-2/B-2/MCF7STARD3_siVAPB/20240527_MCF7STARD3_siVAPB_18_w3SPI 405 DAPI.TIF]

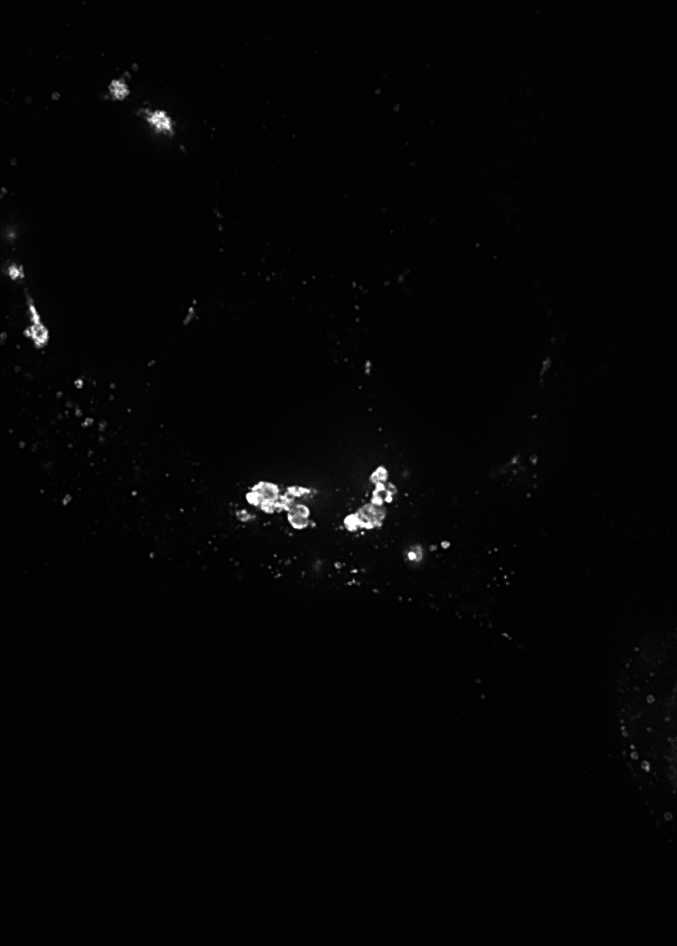

Supplement: Supplementary file 14 — Source data Fig. 6-1 [file 44318_2026_705_MOESM14_ESM.zip › Figure 6-1/B/STARD3WT_CHIR99021/20220308_MCF7_STARD3-CD63_GSK3i-2_SR_w1SPI 491 GFP.TIF]

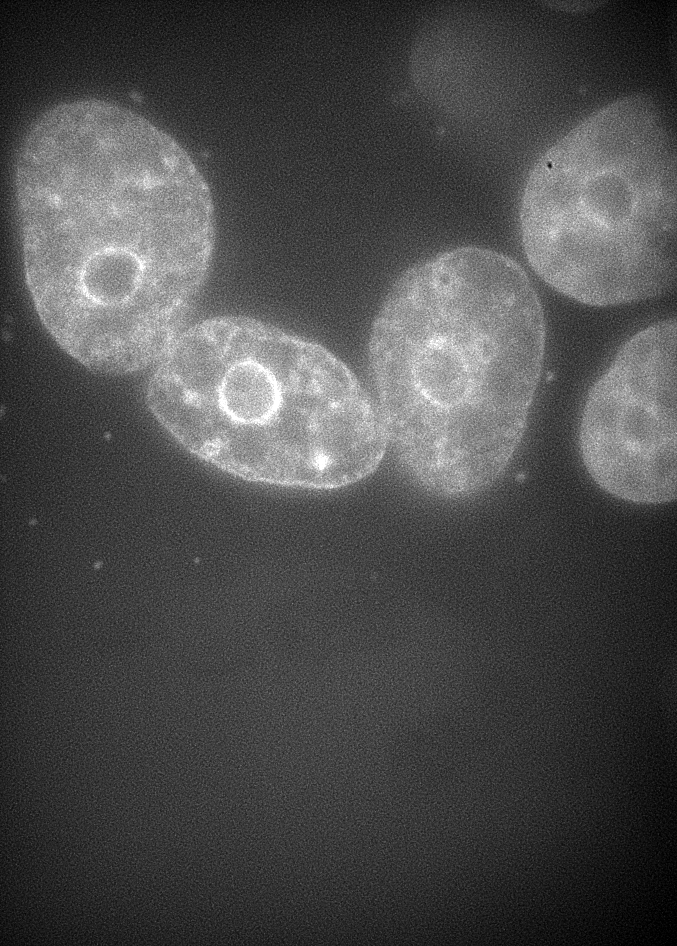

Supplement: Supplementary file 14 — Source data Fig. 6-1 [file 44318_2026_705_MOESM14_ESM.zip › Figure 6-1/B/STARD3WT_CHIR99021/20220308_MCF7_STARD3-CD63_GSK3i-2_SR_w2SPI 405 DAPI.TIF]

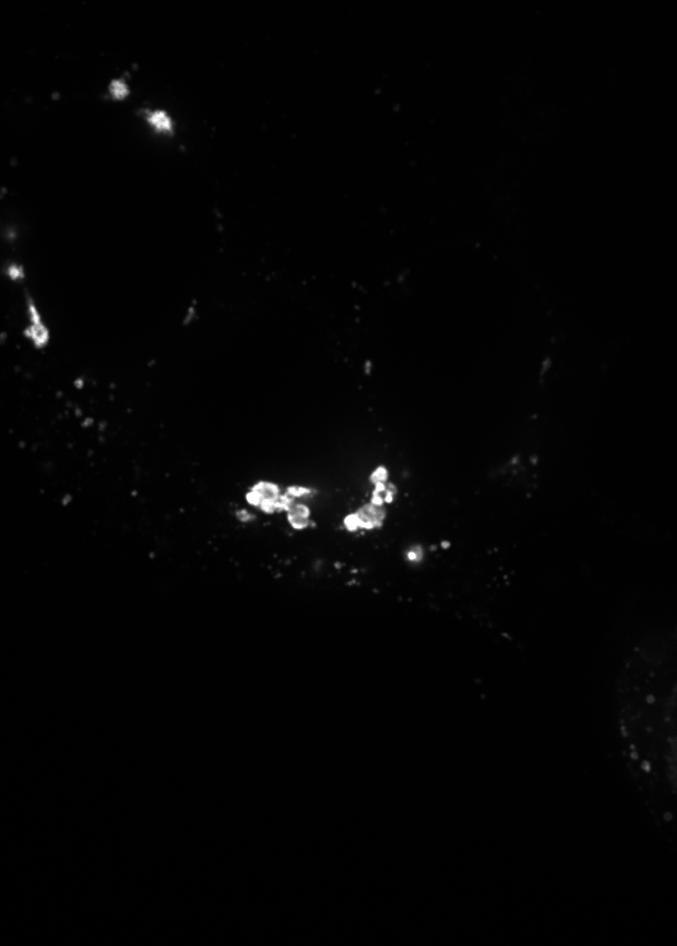

Supplement: Supplementary file 14 — Source data Fig. 6-1 [file 44318_2026_705_MOESM14_ESM.zip › Figure 6-1/B/STARD3WT_CHIR99021/20220308_MCF7_STARD3-CD63_GSK3i-2_w1SPI 491 GFP.TIF]

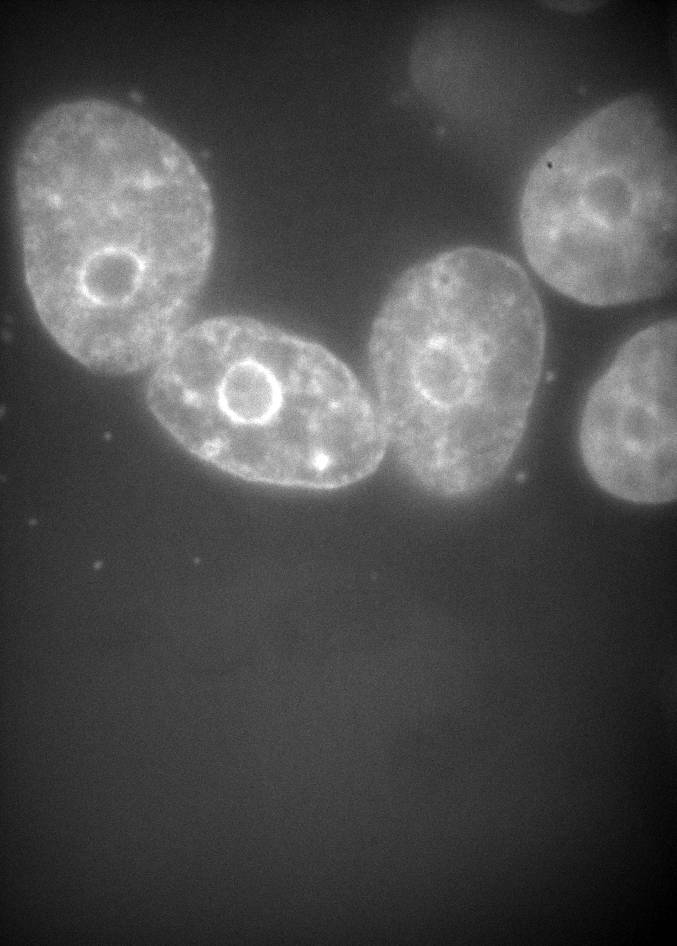

Supplement: Supplementary file 14 — Source data Fig. 6-1 [file 44318_2026_705_MOESM14_ESM.zip › Figure 6-1/B/STARD3WT_CHIR99021/20220308_MCF7_STARD3-CD63_GSK3i-2_w2SPI 405 DAPI.TIF]

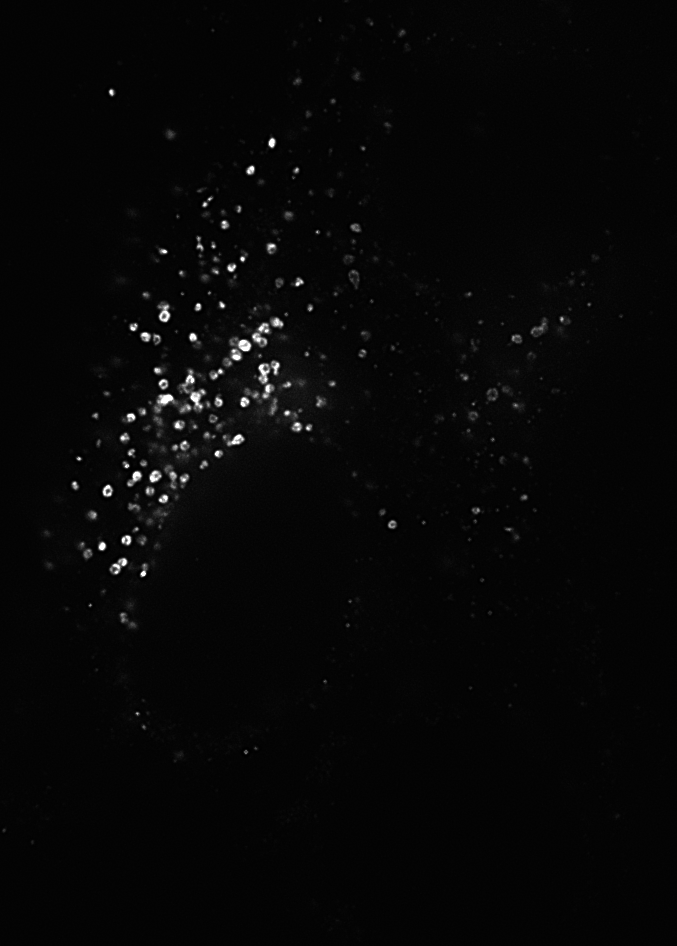

Supplement: Supplementary file 14 — Source data Fig. 6-1 [file 44318_2026_705_MOESM14_ESM.zip › Figure 6-1/B/STARD3WT_NT/20220308_MCF7_STARD3-CD63_WT-3_SR_w1SPI 491 GFP.TIF]

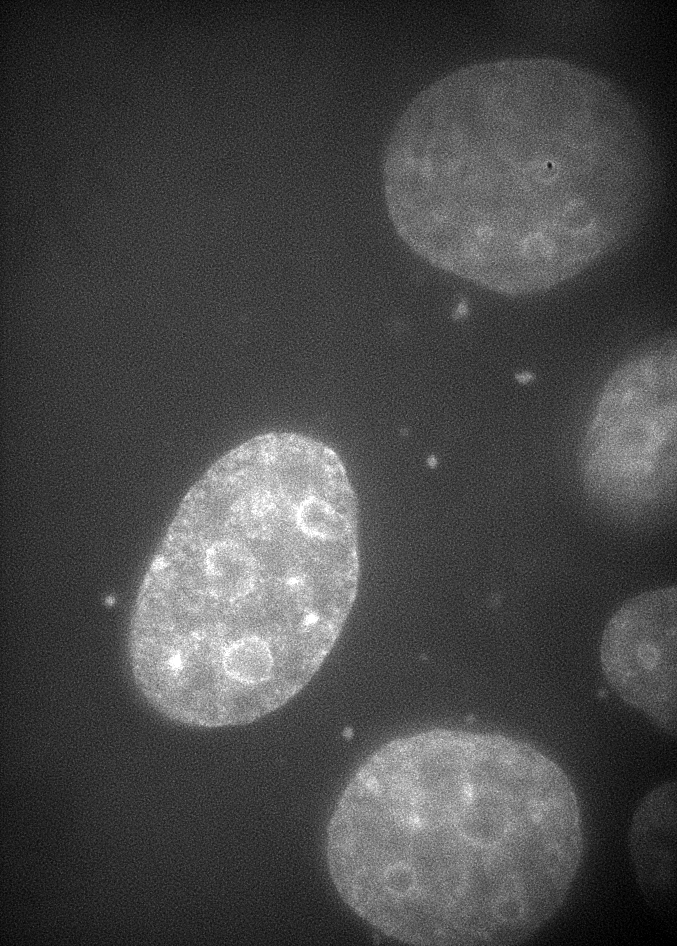

Supplement: Supplementary file 14 — Source data Fig. 6-1 [file 44318_2026_705_MOESM14_ESM.zip › Figure 6-1/B/STARD3WT_NT/20220308_MCF7_STARD3-CD63_WT-3_SR_w2SPI 405 DAPI.TIF]

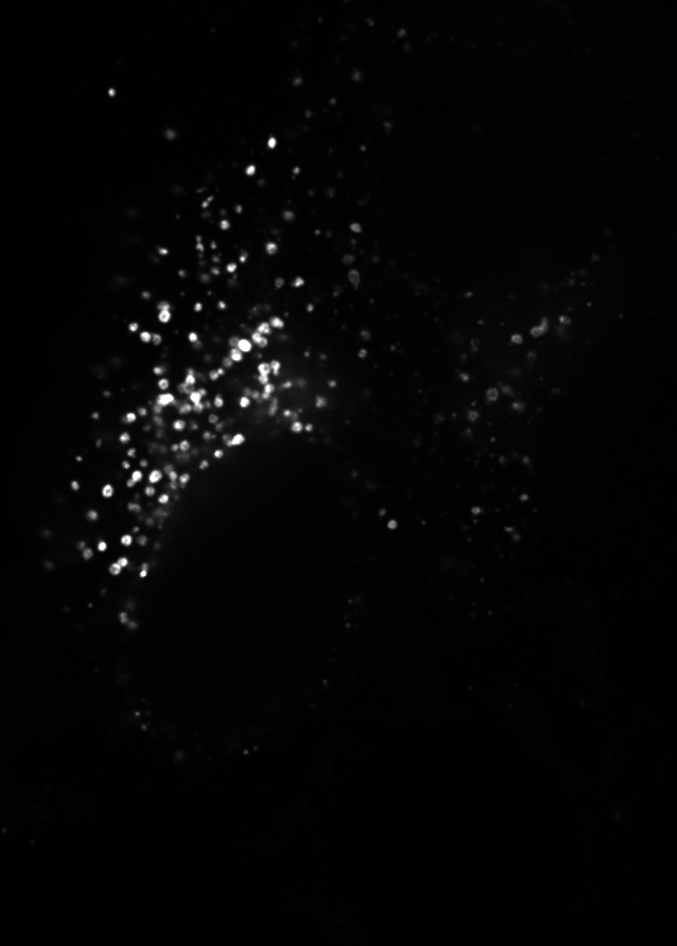

Supplement: Supplementary file 14 — Source data Fig. 6-1 [file 44318_2026_705_MOESM14_ESM.zip › Figure 6-1/B/STARD3WT_NT/20220308_MCF7_STARD3-CD63_WT-3_w1SPI 491 GFP.TIF]

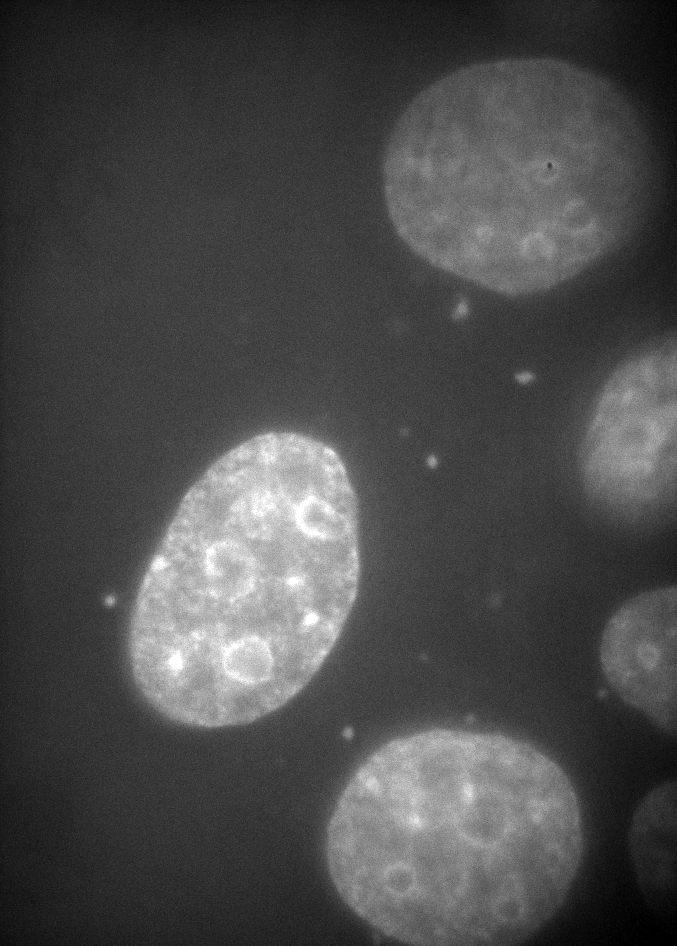

Supplement: Supplementary file 14 — Source data Fig. 6-1 [file 44318_2026_705_MOESM14_ESM.zip › Figure 6-1/B/STARD3WT_NT/20220308_MCF7_STARD3-CD63_WT-3_w2SPI 405 DAPI.TIF]

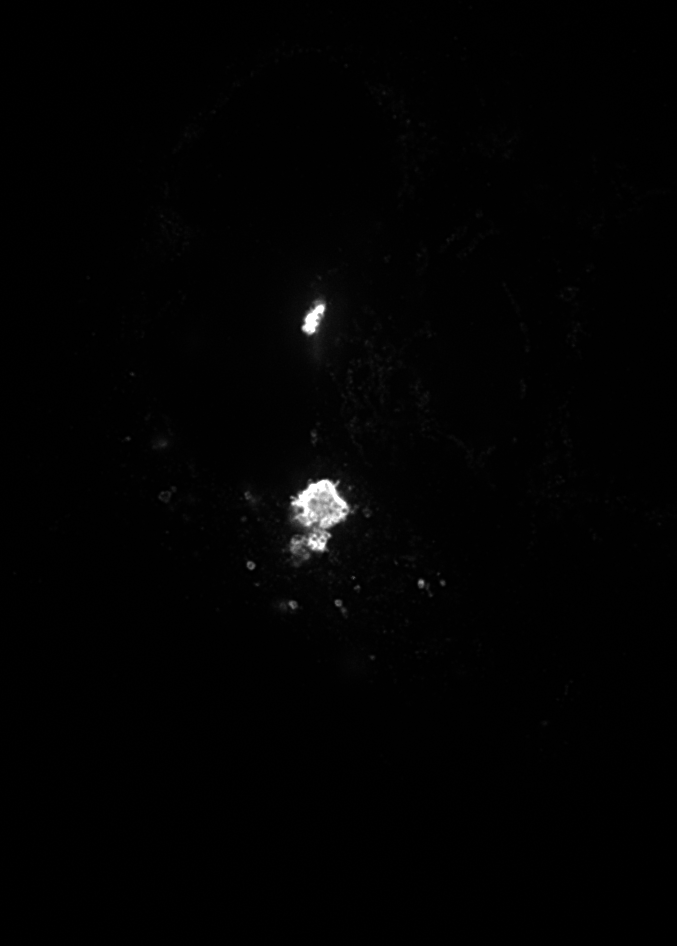

Supplement: Supplementary file 14 — Source data Fig. 6-1 [file 44318_2026_705_MOESM14_ESM.zip › Figure 6-1/C/STARD3S209A_CHIR99021/20220610_STARD3S209A_GSK3i_5_SR_w1SPI 491 GFP.TIF]

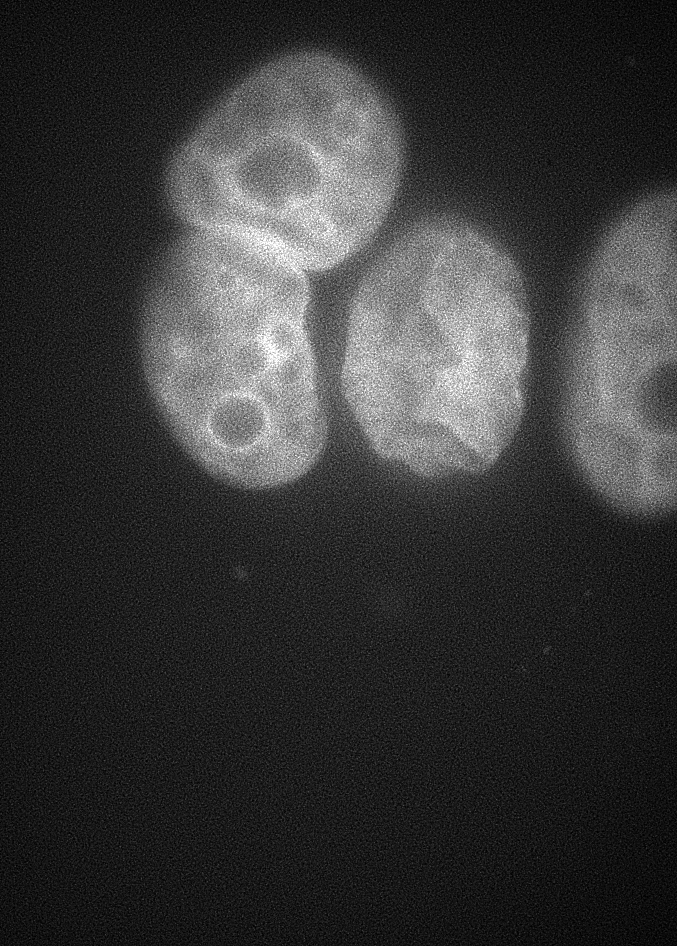

Supplement: Supplementary file 14 — Source data Fig. 6-1 [file 44318_2026_705_MOESM14_ESM.zip › Figure 6-1/C/STARD3S209A_CHIR99021/20220610_STARD3S209A_GSK3i_5_SR_w2SPI 405 DAPI.TIF]

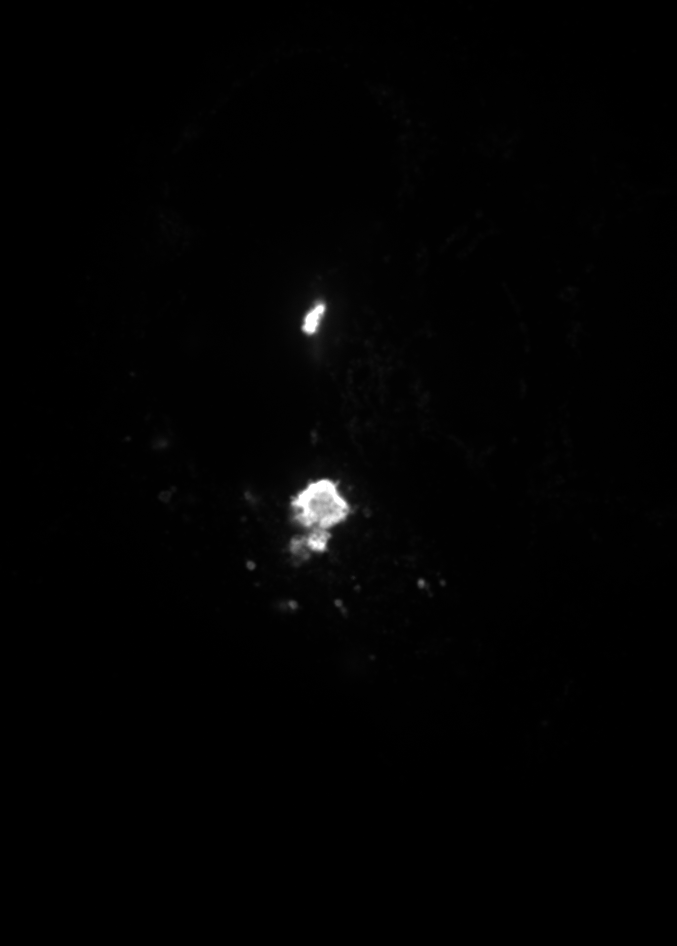

Supplement: Supplementary file 14 — Source data Fig. 6-1 [file 44318_2026_705_MOESM14_ESM.zip › Figure 6-1/C/STARD3S209A_CHIR99021/20220610_STARD3S209A_GSK3i_5_w1SPI 491 GFP.TIF]

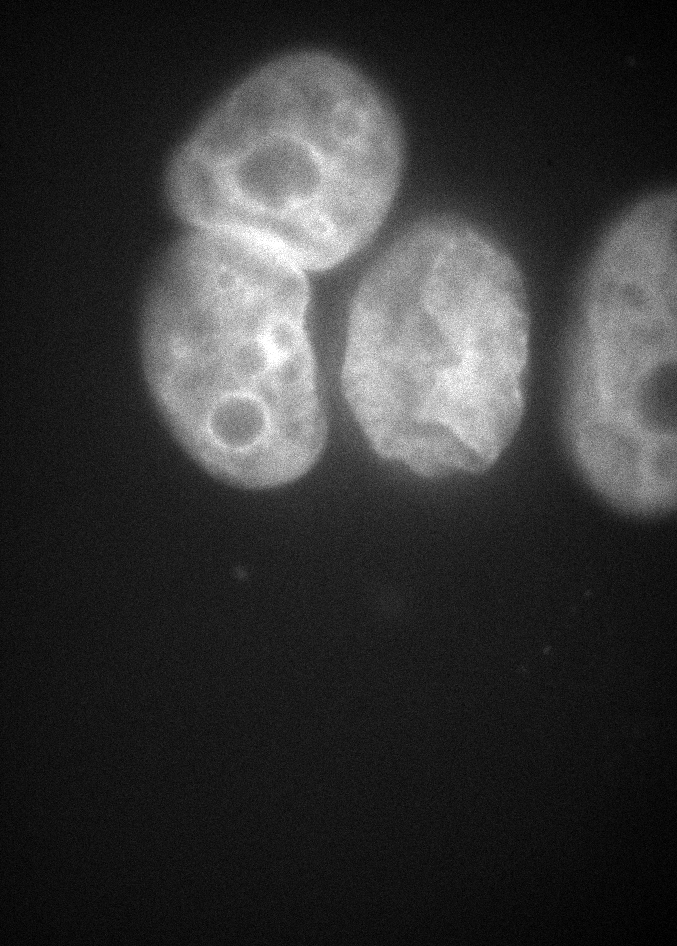

Supplement: Supplementary file 14 — Source data Fig. 6-1 [file 44318_2026_705_MOESM14_ESM.zip › Figure 6-1/C/STARD3S209A_CHIR99021/20220610_STARD3S209A_GSK3i_5_w2SPI 405 DAPI.TIF]

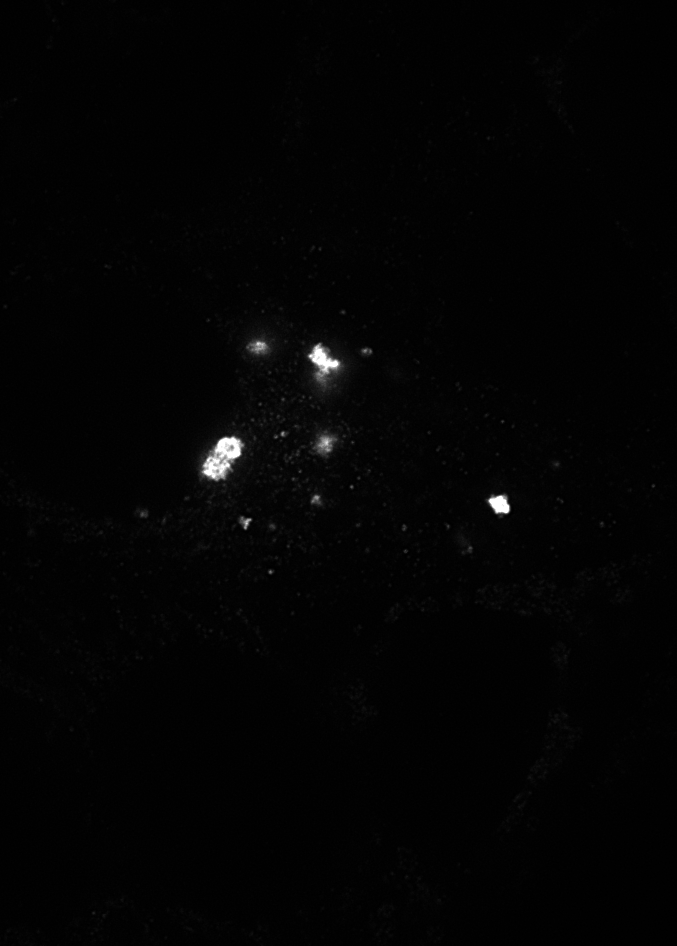

Supplement: Supplementary file 14 — Source data Fig. 6-1 [file 44318_2026_705_MOESM14_ESM.zip › Figure 6-1/C/STARD3S209A_NT/20220610_STARD3S209A_NT_2_SR_w1SPI 491 GFP.TIF]

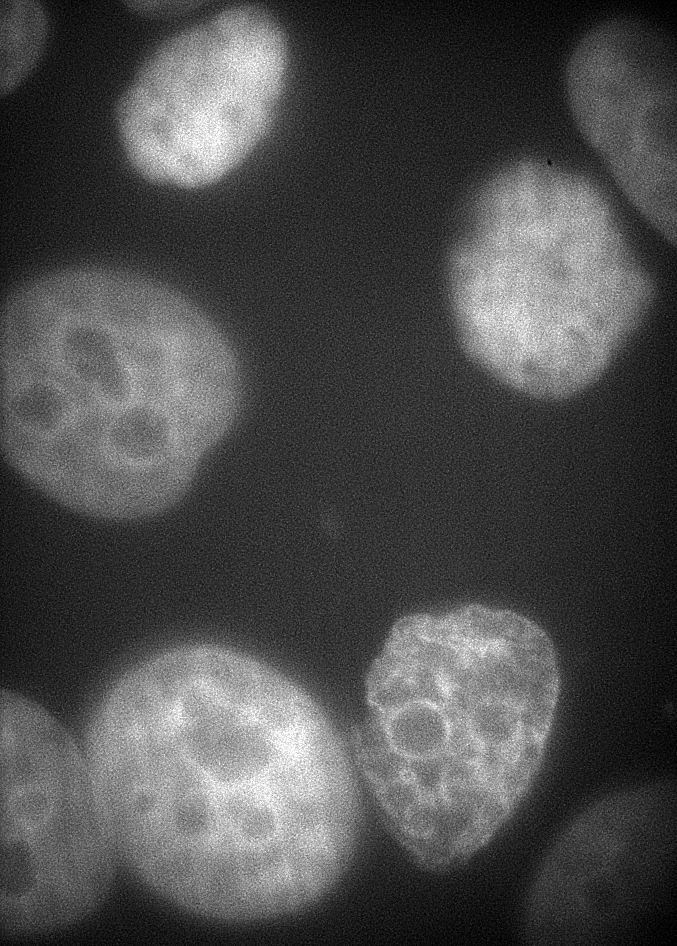

Supplement: Supplementary file 14 — Source data Fig. 6-1 [file 44318_2026_705_MOESM14_ESM.zip › Figure 6-1/C/STARD3S209A_NT/20220610_STARD3S209A_NT_2_SR_w2SPI 405 DAPI.TIF]

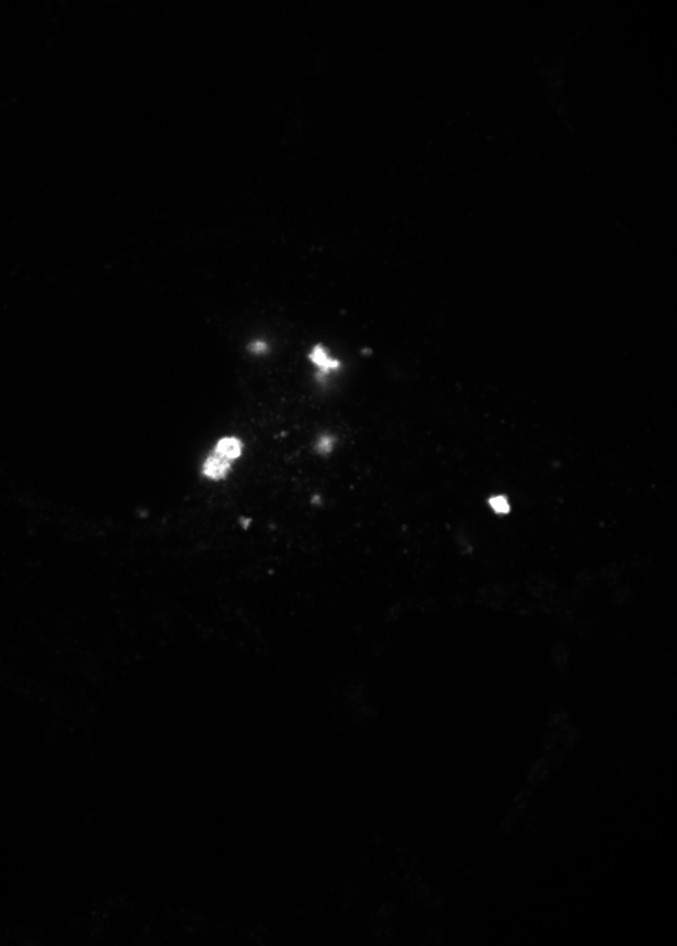

Supplement: Supplementary file 14 — Source data Fig. 6-1 [file 44318_2026_705_MOESM14_ESM.zip › Figure 6-1/C/STARD3S209A_NT/20220610_STARD3S209A_NT_2_w1SPI 491 GFP.TIF]

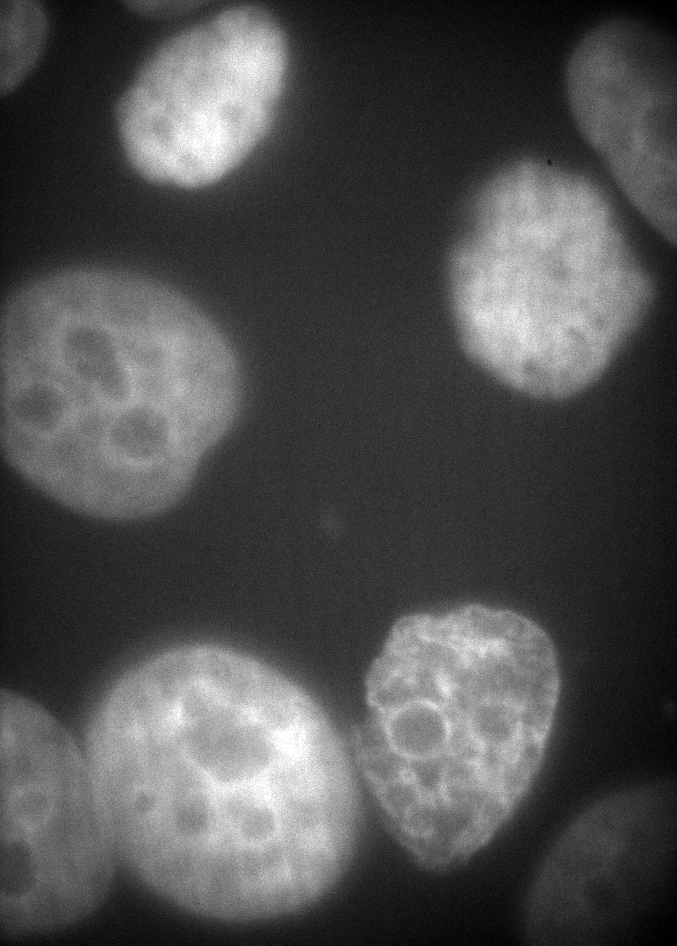

Supplement: Supplementary file 14 — Source data Fig. 6-1 [file 44318_2026_705_MOESM14_ESM.zip › Figure 6-1/C/STARD3S209A_NT/20220610_STARD3S209A_NT_2_w2SPI 405 DAPI.TIF]

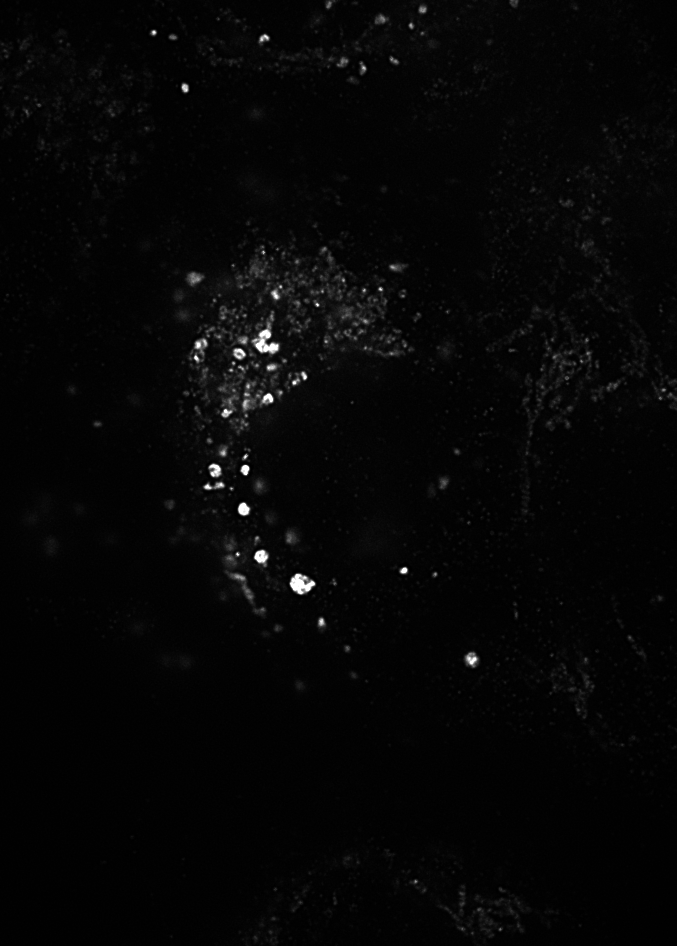

Supplement: Supplementary file 14 — Source data Fig. 6-1 [file 44318_2026_705_MOESM14_ESM.zip › Figure 6-1/D/STARD3_SDPA_CHIR99021/20220509_MCF7_STARD3DPDA_LAMPmch_GSK3i_2_SR_w1SPI 491 GFP.TIF]

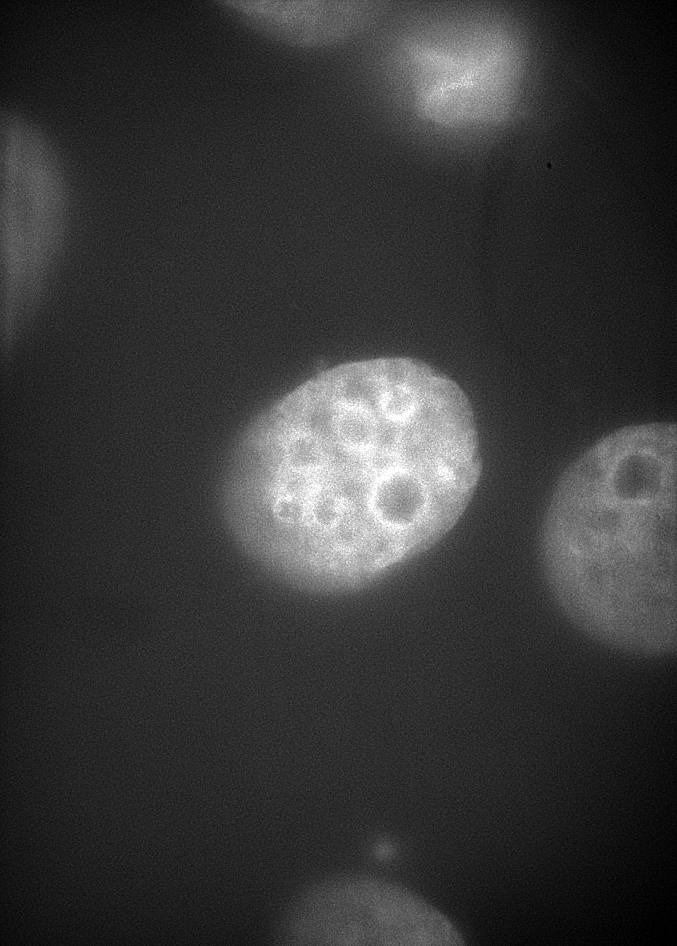

Supplement: Supplementary file 14 — Source data Fig. 6-1 [file 44318_2026_705_MOESM14_ESM.zip › Figure 6-1/D/STARD3_SDPA_CHIR99021/20220509_MCF7_STARD3DPDA_LAMPmch_GSK3i_2_SR_w2SPI 405 DAPI.TIF]

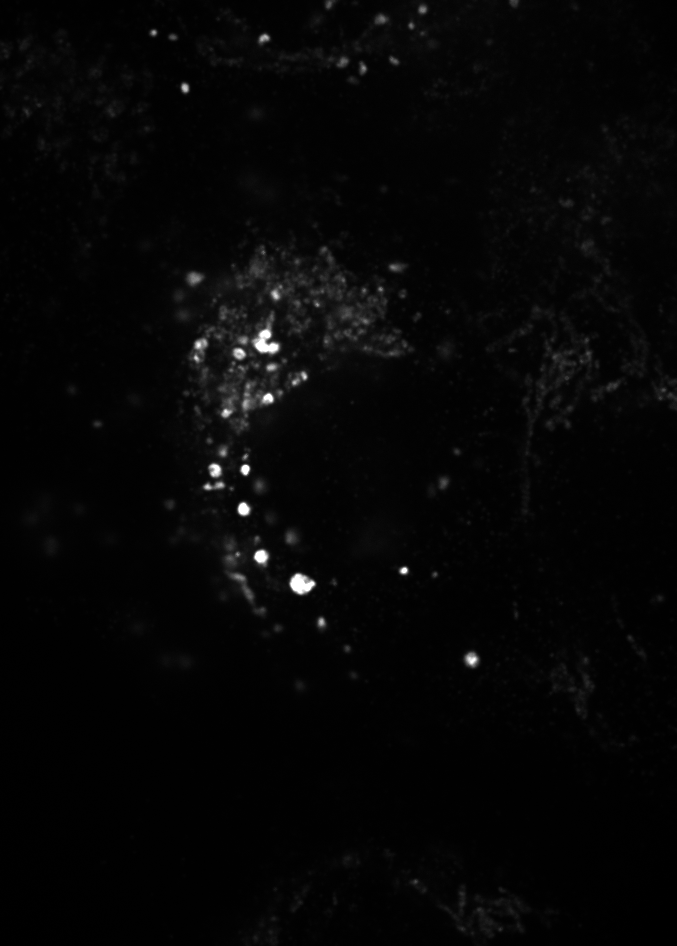

Supplement: Supplementary file 14 — Source data Fig. 6-1 [file 44318_2026_705_MOESM14_ESM.zip › Figure 6-1/D/STARD3_SDPA_CHIR99021/20220509_MCF7_STARD3DPDA_LAMPmch_GSK3i_2_w1SPI 491 GFP.TIF]

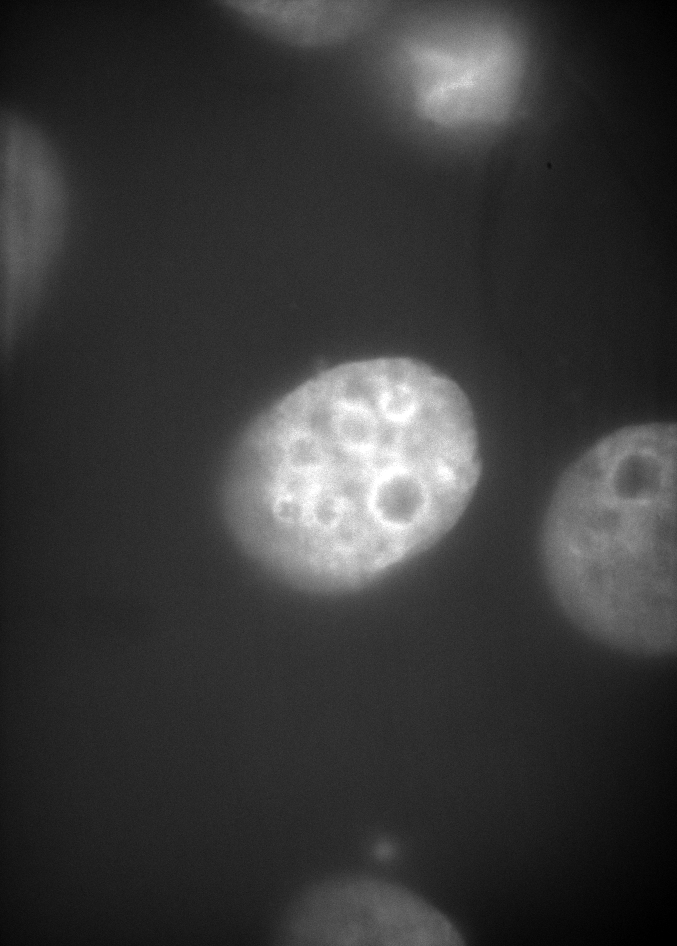

Supplement: Supplementary file 14 — Source data Fig. 6-1 [file 44318_2026_705_MOESM14_ESM.zip › Figure 6-1/D/STARD3_SDPA_CHIR99021/20220509_MCF7_STARD3DPDA_LAMPmch_GSK3i_2_w2SPI 405 DAPI.TIF]

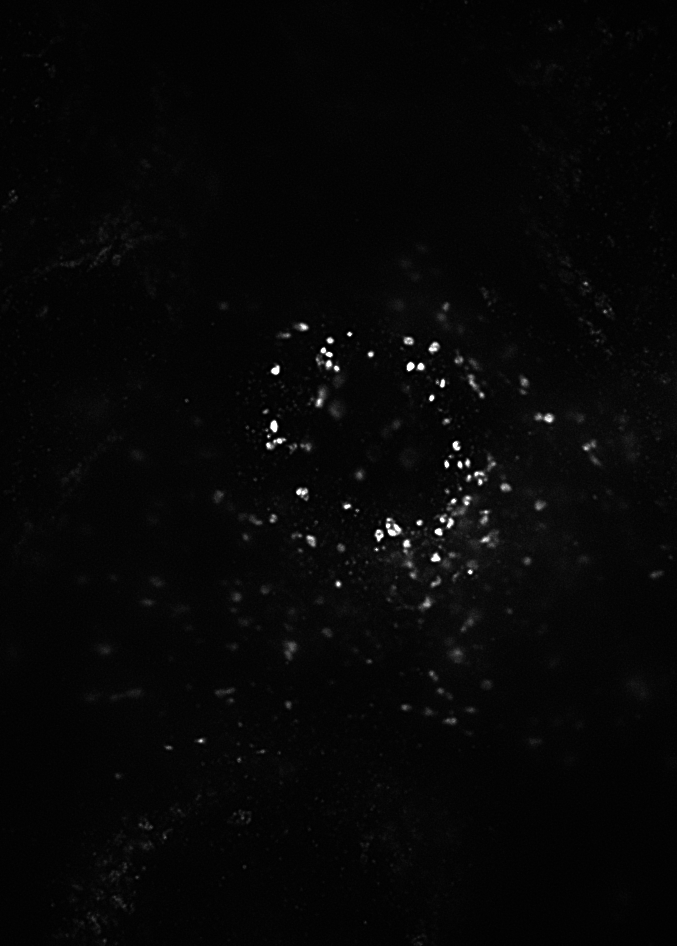

Supplement: Supplementary file 14 — Source data Fig. 6-1 [file 44318_2026_705_MOESM14_ESM.zip › Figure 6-1/D/STARD3_SDPA_NT/20220509_MCF7_STARD3DPDA_LAMPmch_NT_1_SR_w1SPI 491 GFP.TIF]

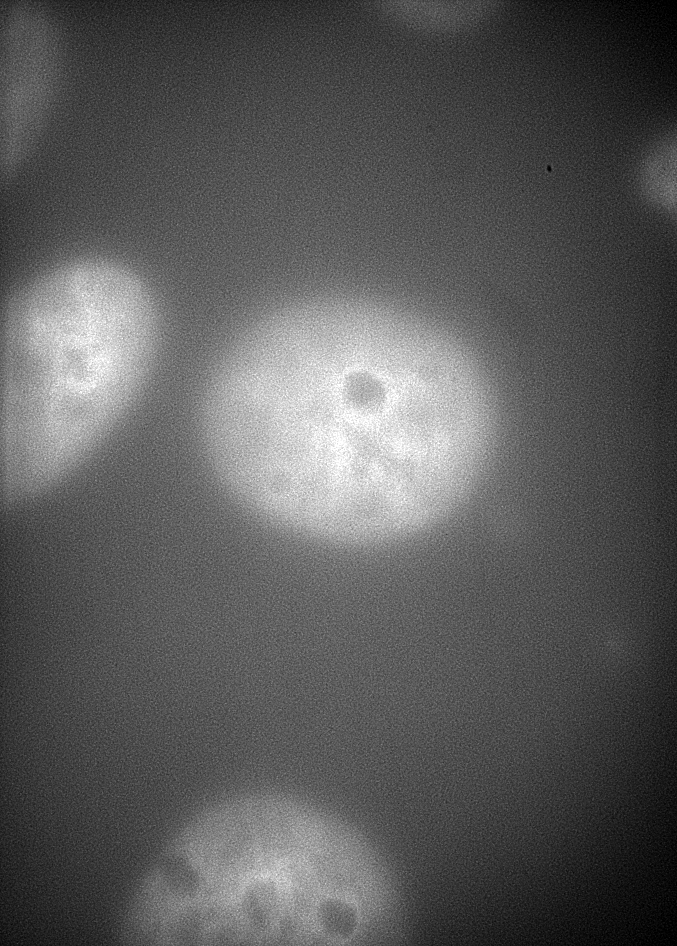

Supplement: Supplementary file 14 — Source data Fig. 6-1 [file 44318_2026_705_MOESM14_ESM.zip › Figure 6-1/D/STARD3_SDPA_NT/20220509_MCF7_STARD3DPDA_LAMPmch_NT_1_SR_w2SPI 405 DAPI.TIF]

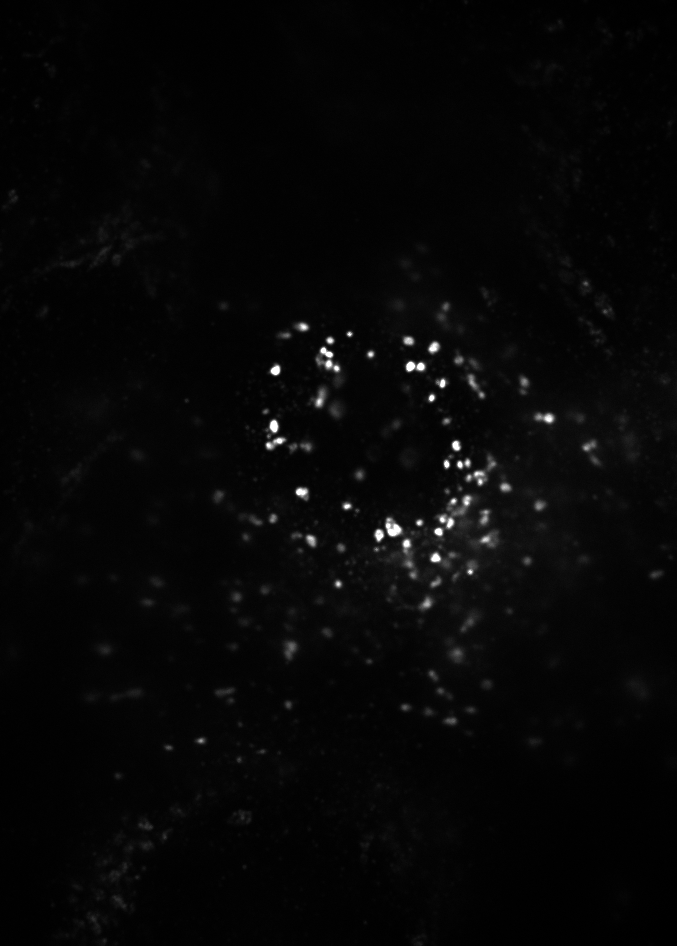

Supplement: Supplementary file 14 — Source data Fig. 6-1 [file 44318_2026_705_MOESM14_ESM.zip › Figure 6-1/D/STARD3_SDPA_NT/20220509_MCF7_STARD3DPDA_LAMPmch_NT_1_w1SPI 491 GFP.TIF]

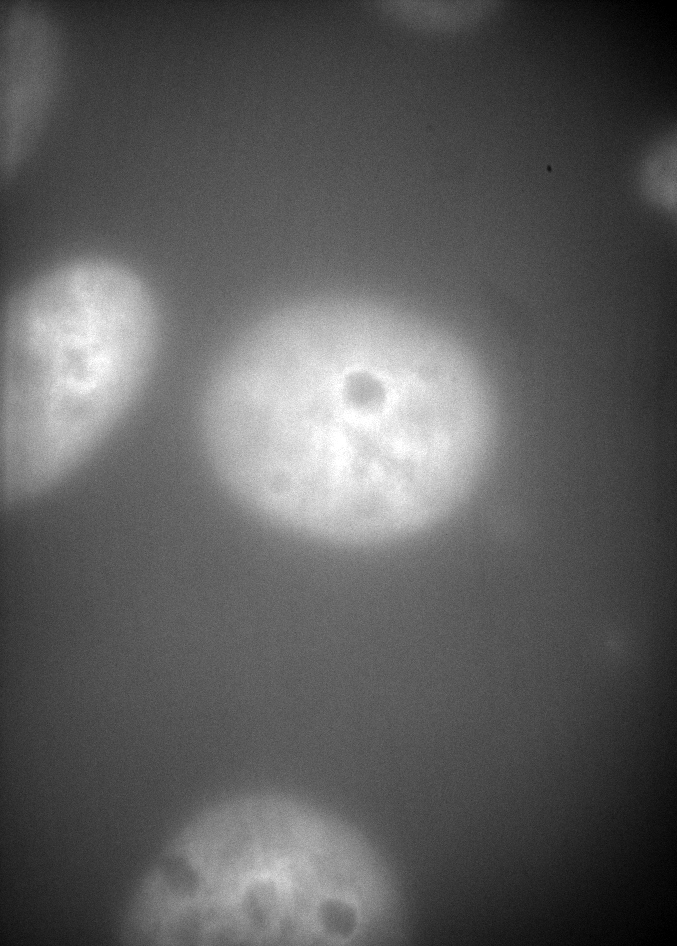

Supplement: Supplementary file 14 — Source data Fig. 6-1 [file 44318_2026_705_MOESM14_ESM.zip › Figure 6-1/D/STARD3_SDPA_NT/20220509_MCF7_STARD3DPDA_LAMPmch_NT_1_w2SPI 405 DAPI.TIF]

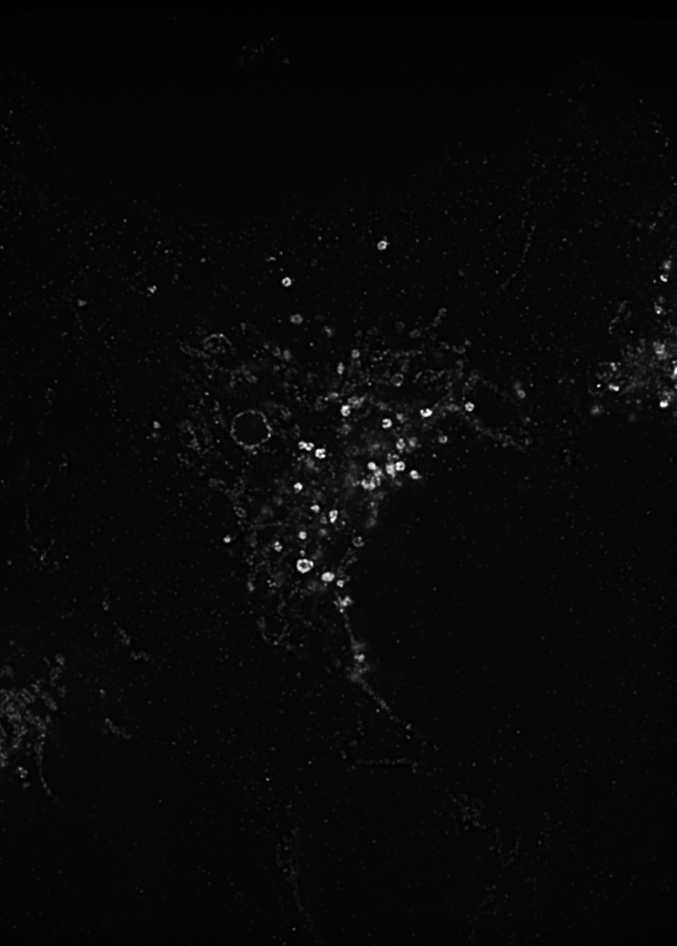

Supplement: Supplementary file 14 — Source data Fig. 6-1 [file 44318_2026_705_MOESM14_ESM.zip › Figure 6-1/E/STARD3deltaSTART_CHIR99021/20220805_MCF7STARD3deltaSTART_GSK3i_2_SR_w1SPI 491 GFP.TIF]

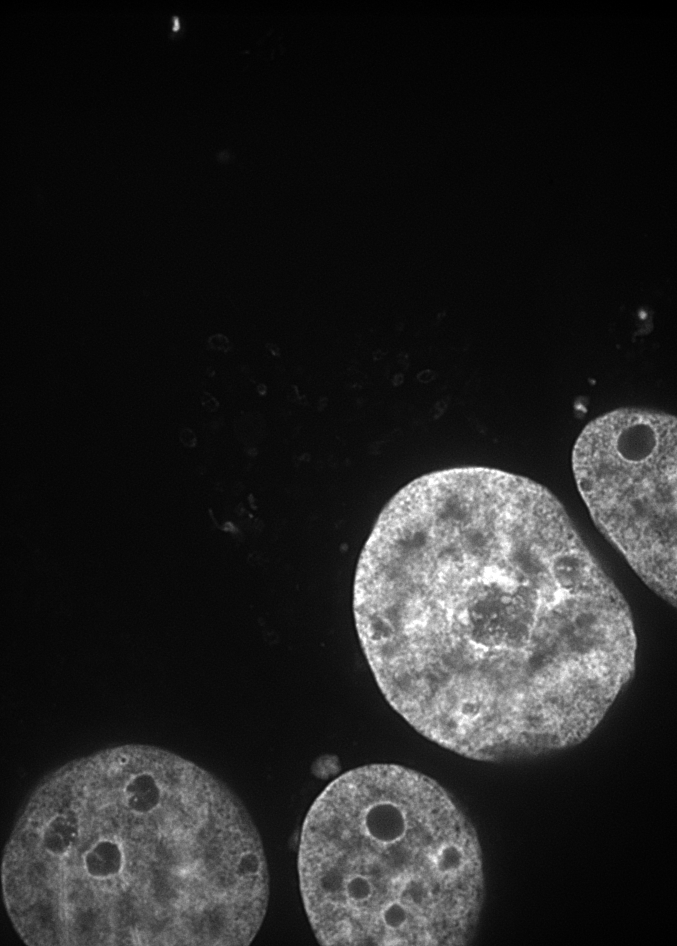

Supplement: Supplementary file 14 — Source data Fig. 6-1 [file 44318_2026_705_MOESM14_ESM.zip › Figure 6-1/E/STARD3deltaSTART_CHIR99021/20220805_MCF7STARD3deltaSTART_GSK3i_2_SR_w2SPI 405 DAPI.TIF]

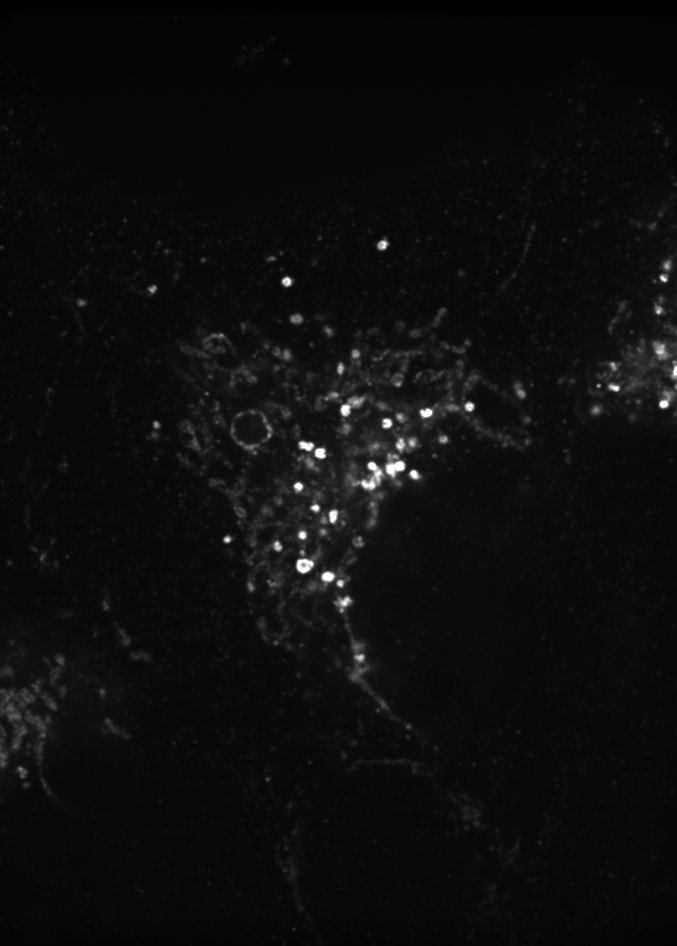

Supplement: Supplementary file 14 — Source data Fig. 6-1 [file 44318_2026_705_MOESM14_ESM.zip › Figure 6-1/E/STARD3deltaSTART_CHIR99021/20220805_MCF7STARD3deltaSTART_GSK3i_2_w1SPI 491 GFP.TIF]

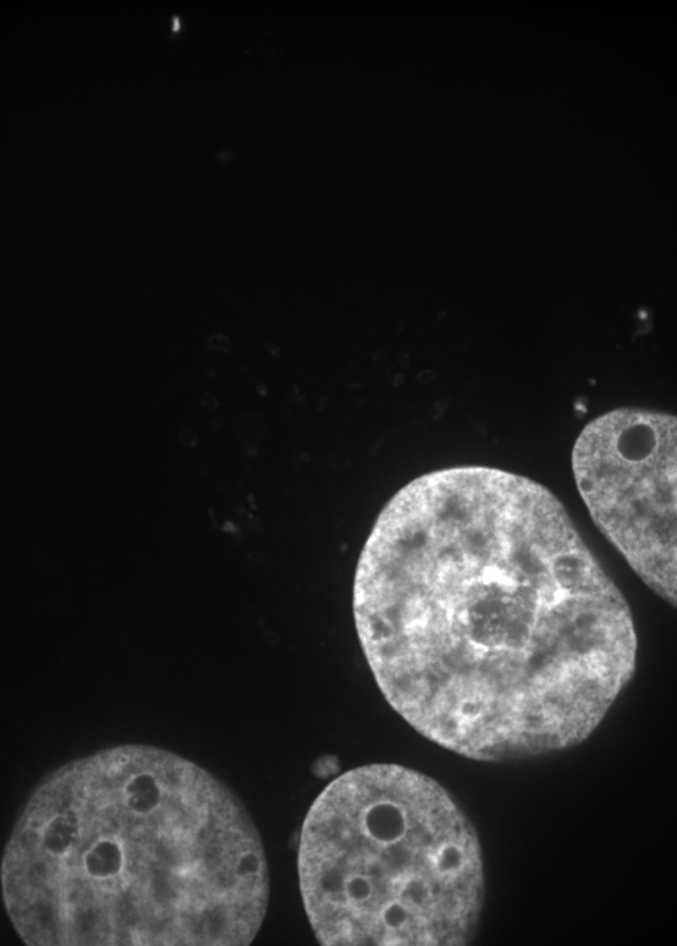

Supplement: Supplementary file 14 — Source data Fig. 6-1 [file 44318_2026_705_MOESM14_ESM.zip › Figure 6-1/E/STARD3deltaSTART_CHIR99021/20220805_MCF7STARD3deltaSTART_GSK3i_2_w2SPI 405 DAPI.TIF]

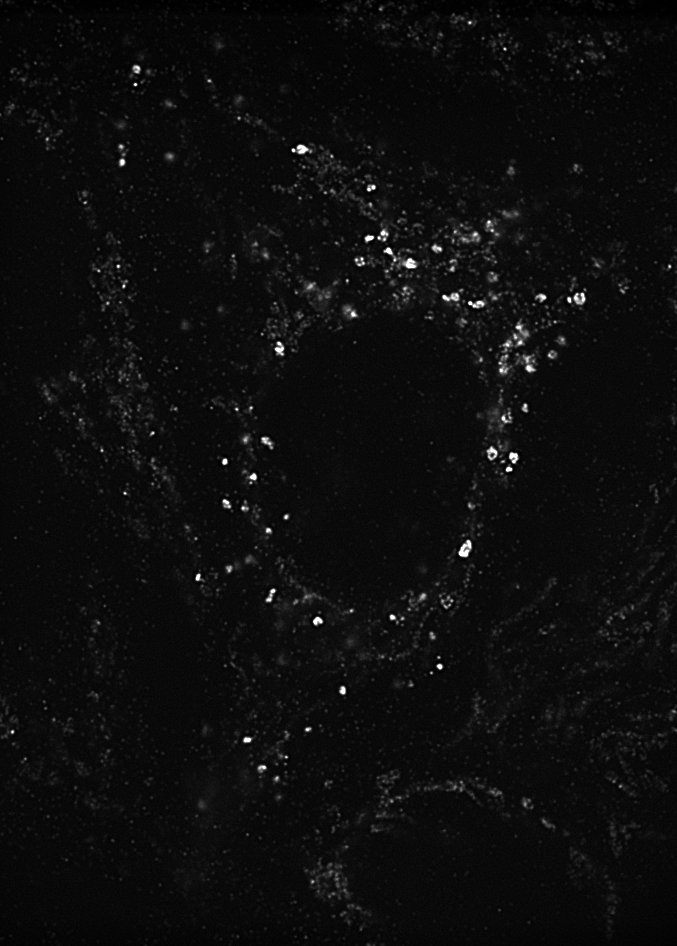

Supplement: Supplementary file 14 — Source data Fig. 6-1 [file 44318_2026_705_MOESM14_ESM.zip › Figure 6-1/E/STARD3deltaSTART_NT/20220805_MCF7STARD3deltaSTART_NT_7_SR_w1SPI 491 GFP.TIF]

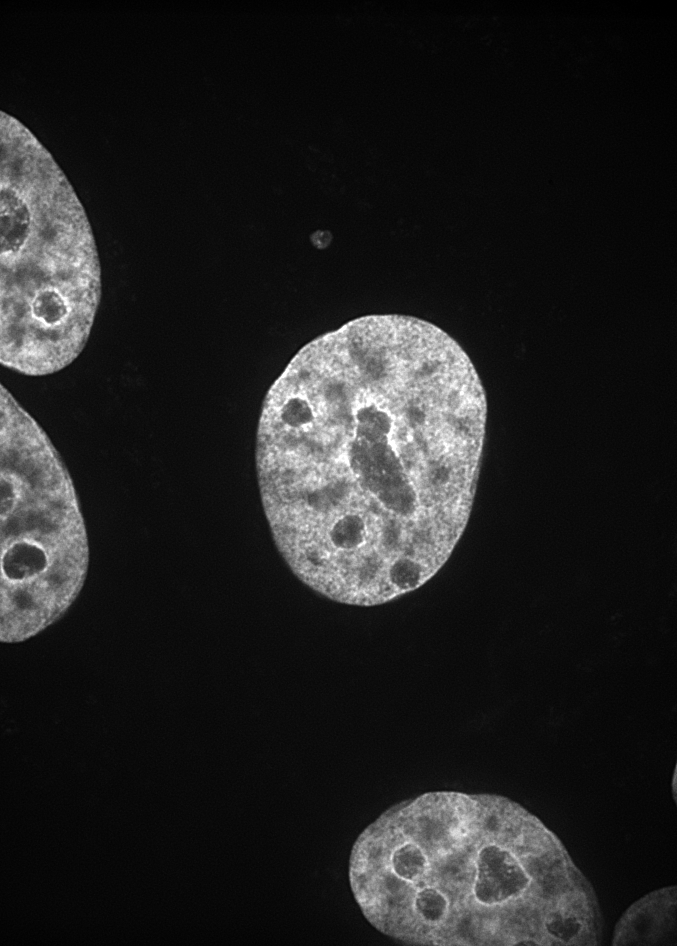

Supplement: Supplementary file 14 — Source data Fig. 6-1 [file 44318_2026_705_MOESM14_ESM.zip › Figure 6-1/E/STARD3deltaSTART_NT/20220805_MCF7STARD3deltaSTART_NT_7_SR_w2SPI 405 DAPI.TIF]

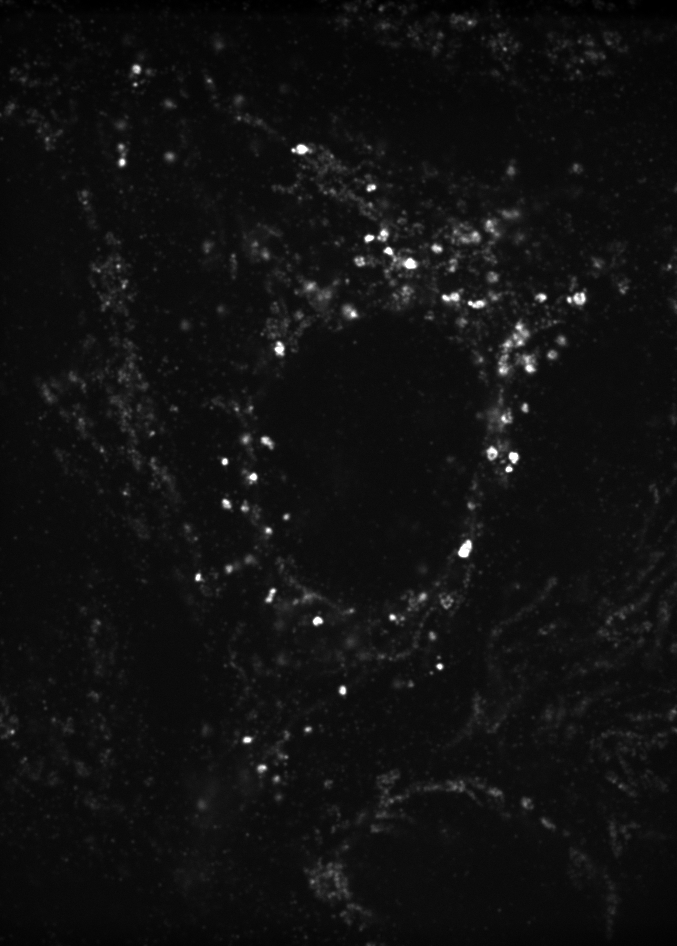

Supplement: Supplementary file 14 — Source data Fig. 6-1 [file 44318_2026_705_MOESM14_ESM.zip › Figure 6-1/E/STARD3deltaSTART_NT/20220805_MCF7STARD3deltaSTART_NT_7_w1SPI 491 GFP.TIF]

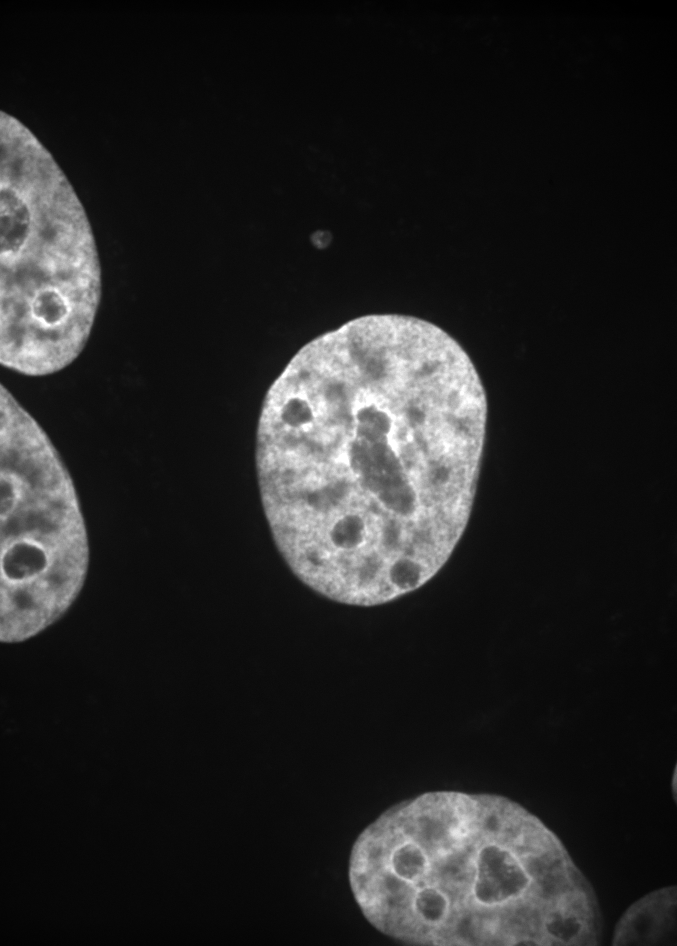

Supplement: Supplementary file 14 — Source data Fig. 6-1 [file 44318_2026_705_MOESM14_ESM.zip › Figure 6-1/E/STARD3deltaSTART_NT/20220805_MCF7STARD3deltaSTART_NT_7_w2SPI 405 DAPI.TIF]

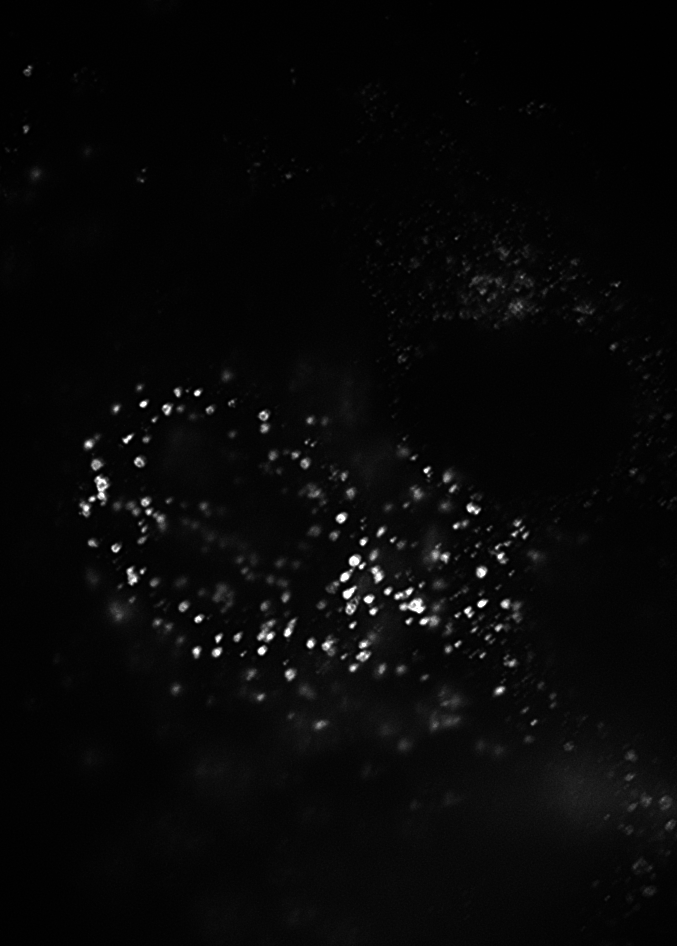

Supplement: Supplementary file 15 — Source data Fig. 6-2 [file 44318_2026_705_MOESM15_ESM.zip › Figure 6-2/H/STARD3NL_CHIR99021/20220728_MCF7STDAR3NL_GSK3i_2_SR_w1SPI 491 GFP.TIF]

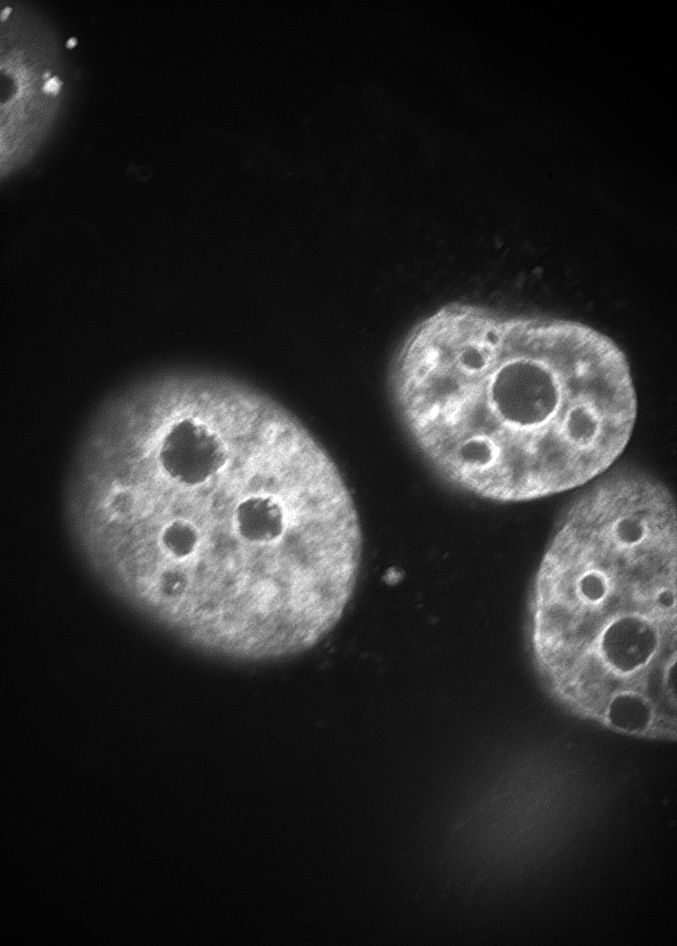

Supplement: Supplementary file 15 — Source data Fig. 6-2 [file 44318_2026_705_MOESM15_ESM.zip › Figure 6-2/H/STARD3NL_CHIR99021/20220728_MCF7STDAR3NL_GSK3i_2_SR_w2SPI 405 DAPI.TIF]

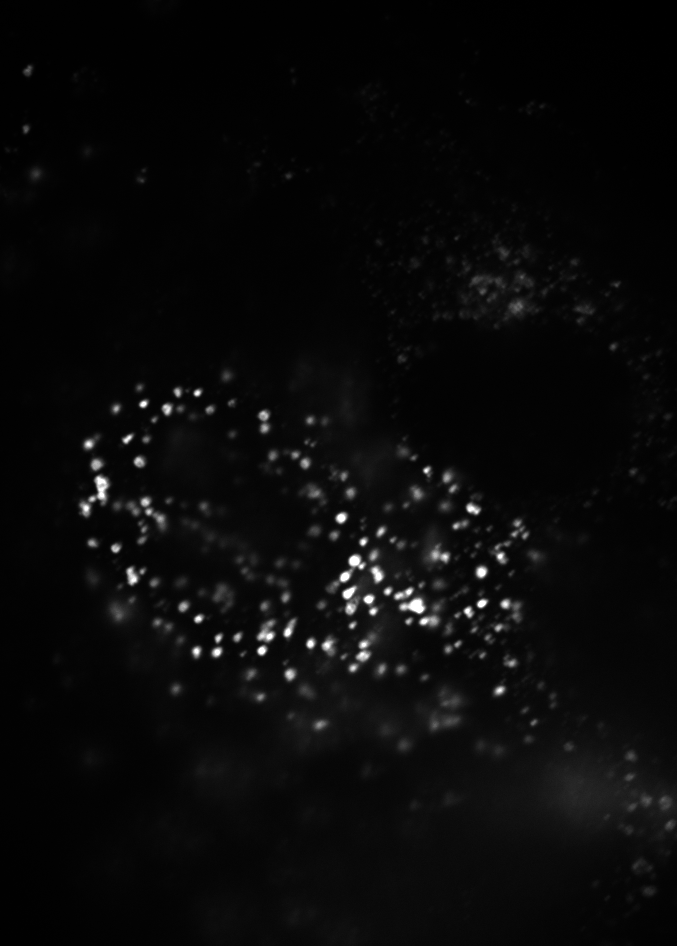

Supplement: Supplementary file 15 — Source data Fig. 6-2 [file 44318_2026_705_MOESM15_ESM.zip › Figure 6-2/H/STARD3NL_CHIR99021/20220728_MCF7STDAR3NL_GSK3i_2_w1SPI 491 GFP.TIF]

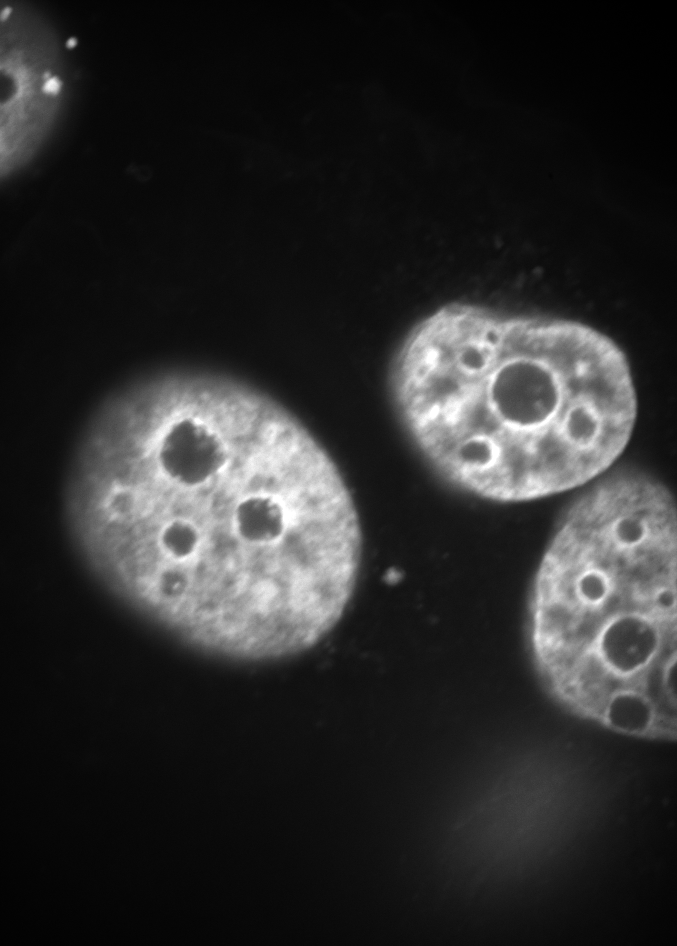

Supplement: Supplementary file 15 — Source data Fig. 6-2 [file 44318_2026_705_MOESM15_ESM.zip › Figure 6-2/H/STARD3NL_CHIR99021/20220728_MCF7STDAR3NL_GSK3i_2_w2SPI 405 DAPI.TIF]

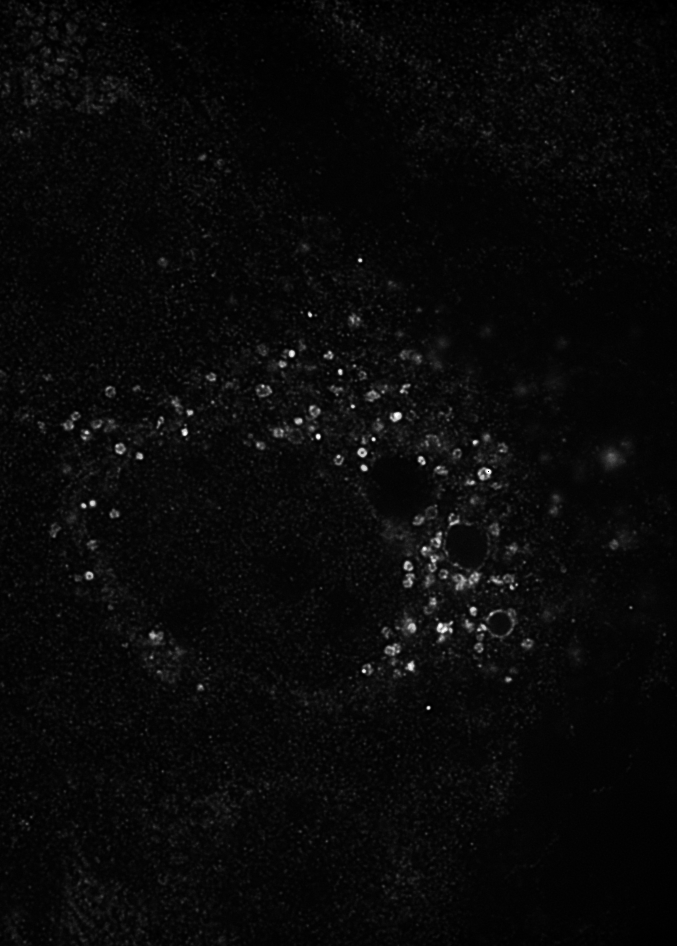

Supplement: Supplementary file 15 — Source data Fig. 6-2 [file 44318_2026_705_MOESM15_ESM.zip › Figure 6-2/H/STARD3NL_NT/20220805_MCF7STARD3NL_NT_9_SR_w1SPI 491 GFP.TIF]

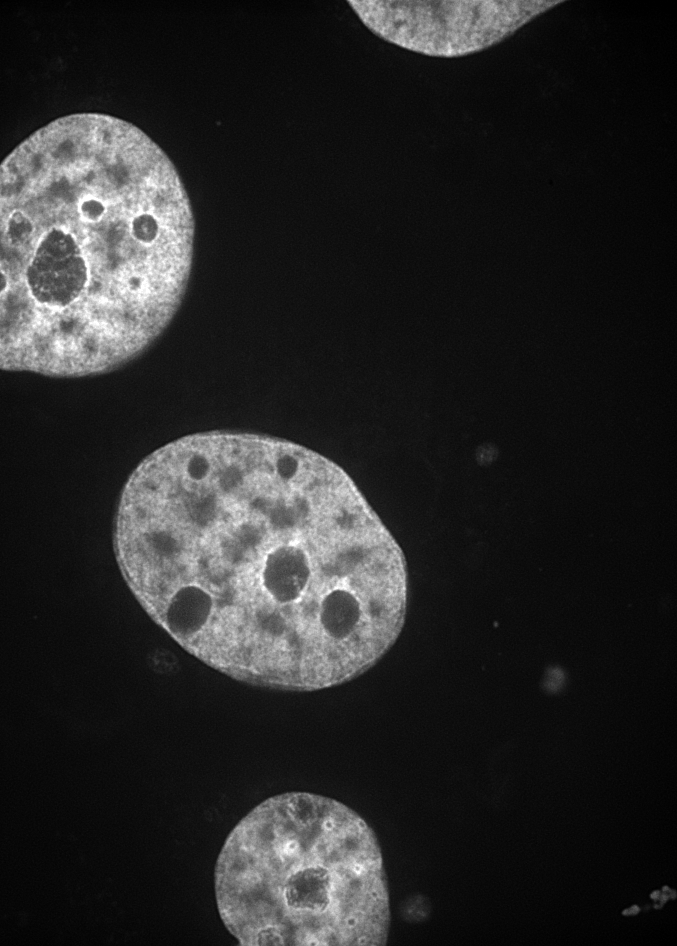

Supplement: Supplementary file 15 — Source data Fig. 6-2 [file 44318_2026_705_MOESM15_ESM.zip › Figure 6-2/H/STARD3NL_NT/20220805_MCF7STARD3NL_NT_9_SR_w2SPI 405 DAPI.TIF]

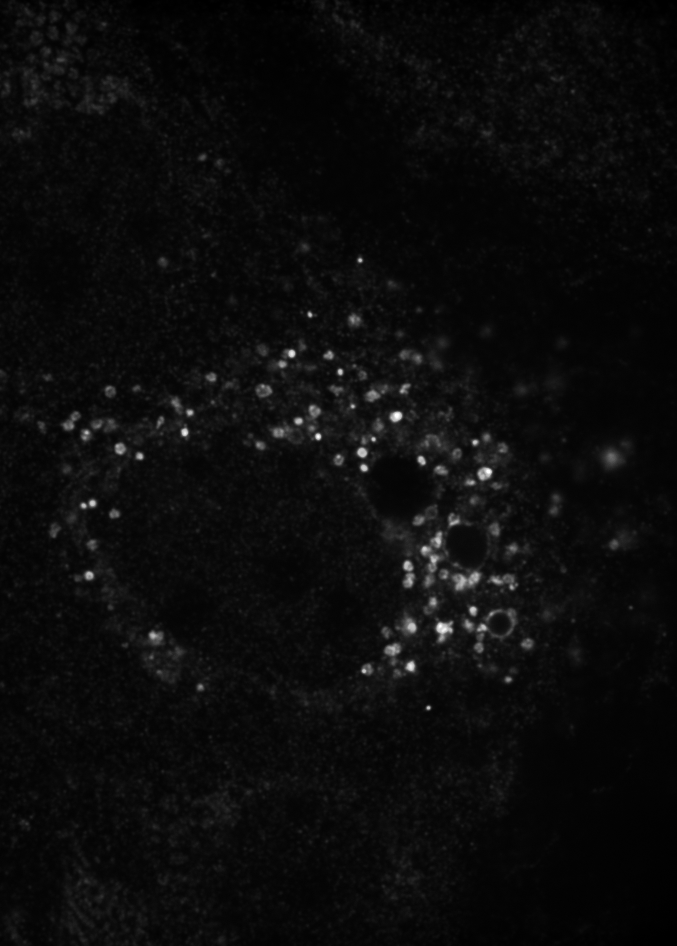

Supplement: Supplementary file 15 — Source data Fig. 6-2 [file 44318_2026_705_MOESM15_ESM.zip › Figure 6-2/H/STARD3NL_NT/20220805_MCF7STARD3NL_NT_9_w1SPI 491 GFP.TIF]

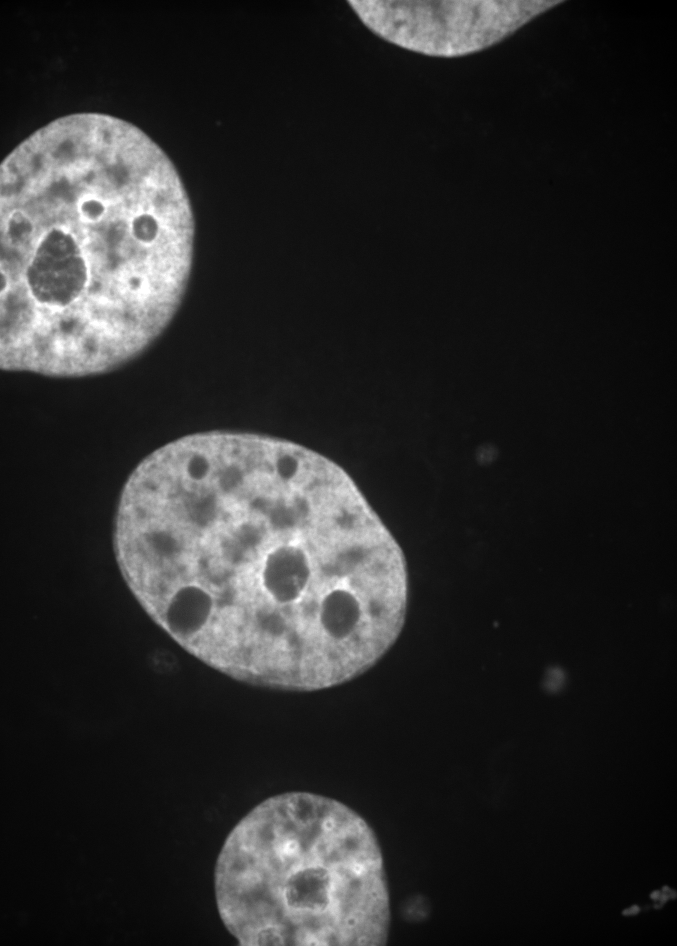

Supplement: Supplementary file 15 — Source data Fig. 6-2 [file 44318_2026_705_MOESM15_ESM.zip › Figure 6-2/H/STARD3NL_NT/20220805_MCF7STARD3NL_NT_9_w2SPI 405 DAPI.TIF]

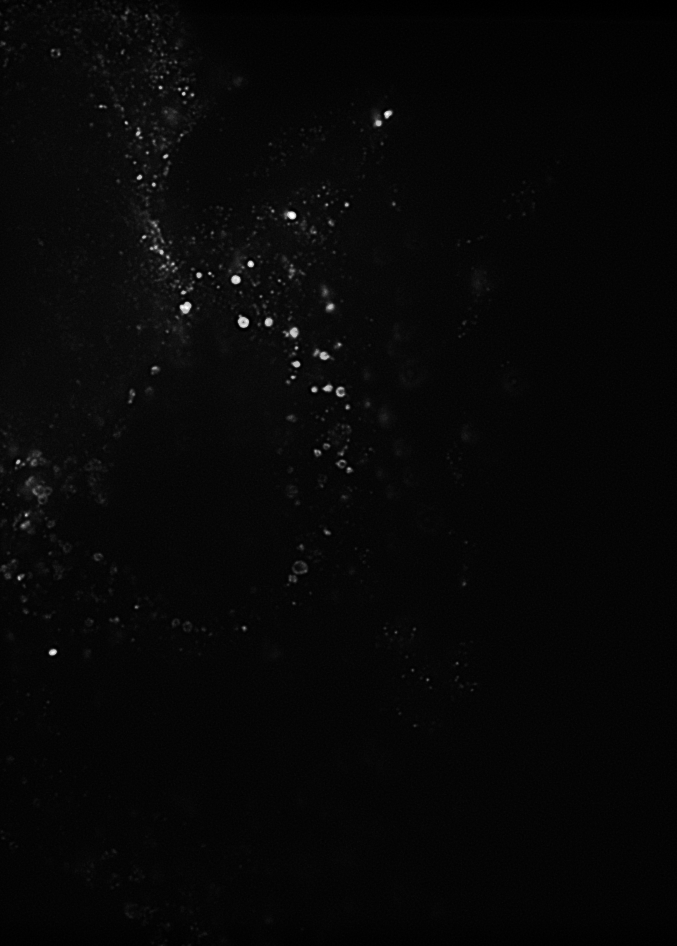

Supplement: Supplementary file 15 — Source data Fig. 6-2 [file 44318_2026_705_MOESM15_ESM.zip › Figure 6-2/I/STARD3NL_deltaFFAT_CHIR99021/20220823_STARD3NLdeltaFFAT_GSK3i_2_SR_w1SPI 491 GFP.TIF]

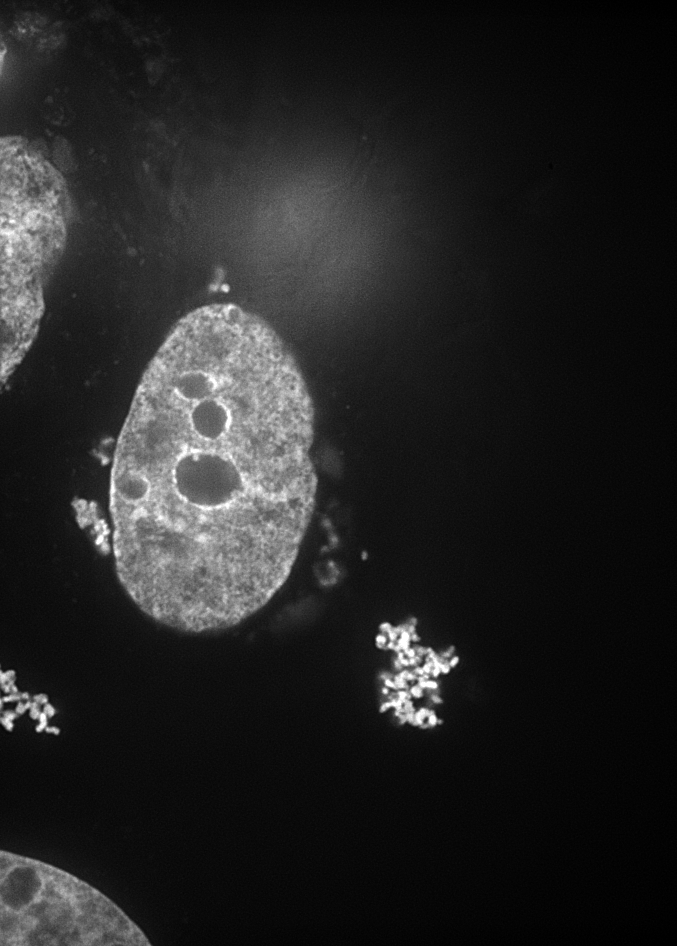

Supplement: Supplementary file 15 — Source data Fig. 6-2 [file 44318_2026_705_MOESM15_ESM.zip › Figure 6-2/I/STARD3NL_deltaFFAT_CHIR99021/20220823_STARD3NLdeltaFFAT_GSK3i_2_SR_w2SPI 405 DAPI.TIF]

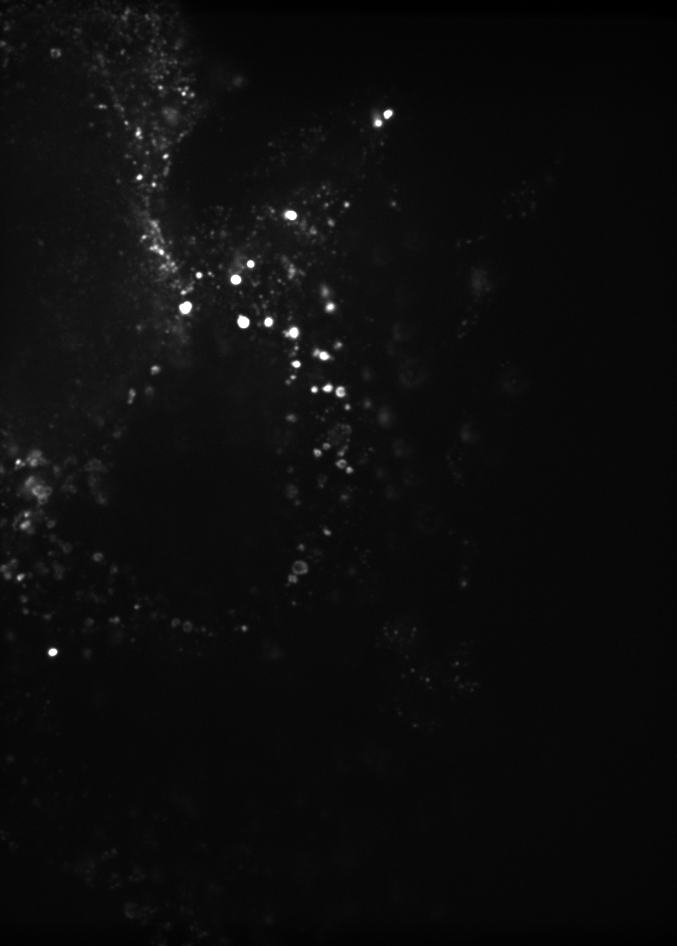

Supplement: Supplementary file 15 — Source data Fig. 6-2 [file 44318_2026_705_MOESM15_ESM.zip › Figure 6-2/I/STARD3NL_deltaFFAT_CHIR99021/20220823_STARD3NLdeltaFFAT_GSK3i_2_w1SPI 491 GFP.TIF]

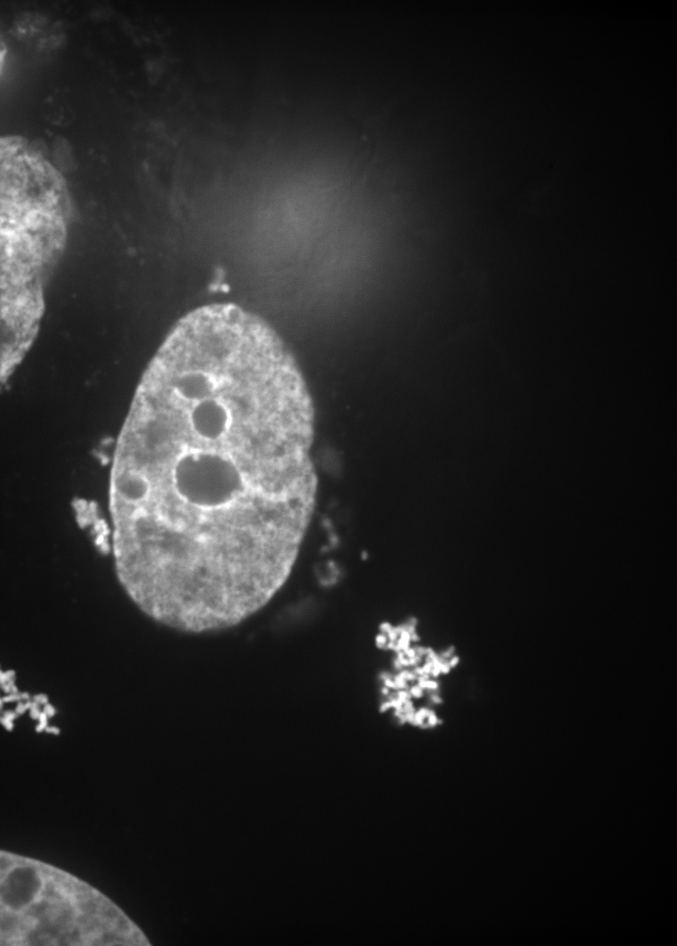

Supplement: Supplementary file 15 — Source data Fig. 6-2 [file 44318_2026_705_MOESM15_ESM.zip › Figure 6-2/I/STARD3NL_deltaFFAT_CHIR99021/20220823_STARD3NLdeltaFFAT_GSK3i_2_w2SPI 405 DAPI.TIF]

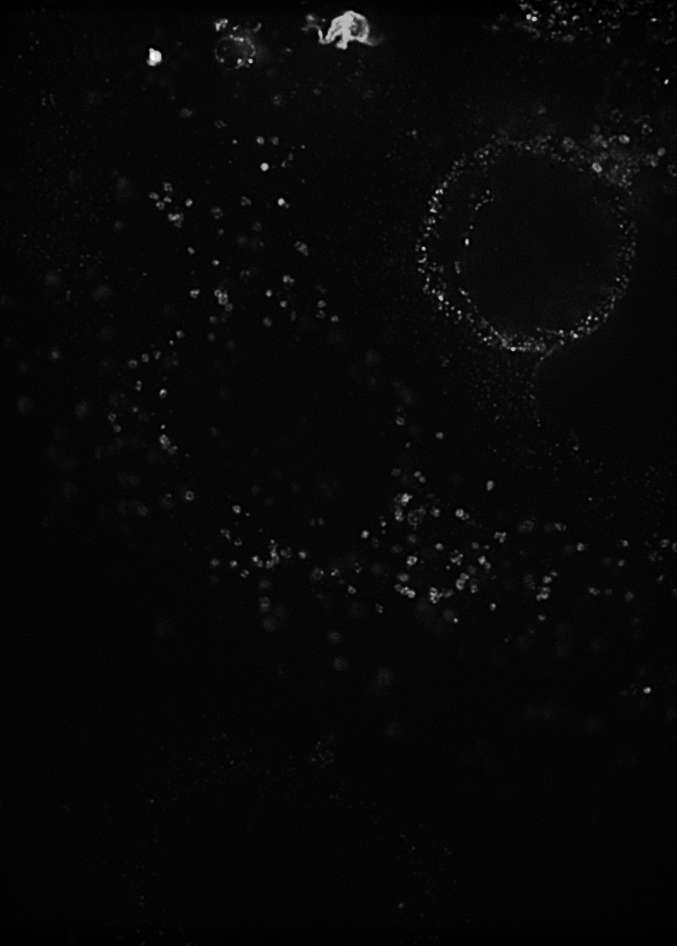

Supplement: Supplementary file 15 — Source data Fig. 6-2 [file 44318_2026_705_MOESM15_ESM.zip › Figure 6-2/I/STARD3NL_deltaFFAT_NT/20220823_STARD3NLdeltaFFAT_NT_5_SR_w1SPI 491 GFP.TIF]

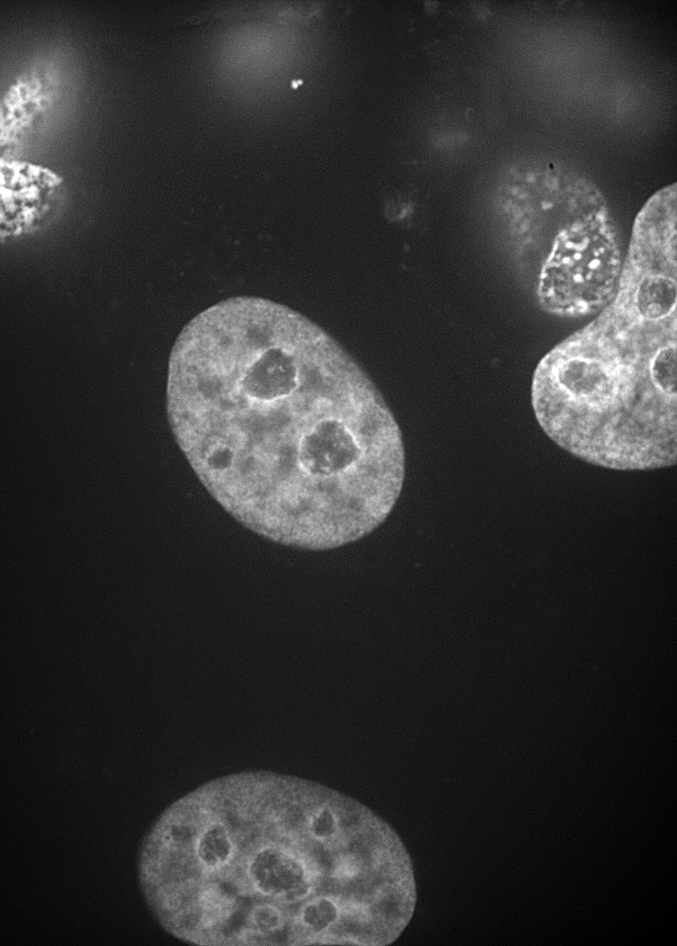

Supplement: Supplementary file 15 — Source data Fig. 6-2 [file 44318_2026_705_MOESM15_ESM.zip › Figure 6-2/I/STARD3NL_deltaFFAT_NT/20220823_STARD3NLdeltaFFAT_NT_5_SR_w2SPI 405 DAPI.TIF]
