# Supplementary figures and images for: STARD3 regulates lysosome positioning and contacts via a GSK3-controlled phosphorylation switch (part 3 of 7)
Source: EMBO J. 2026 Feb 25;45(7):2239–77. doi: 10.1038/s44318-026-00705-3 (PMC13044316; doi:10.1038/s44318-026-00705-3)

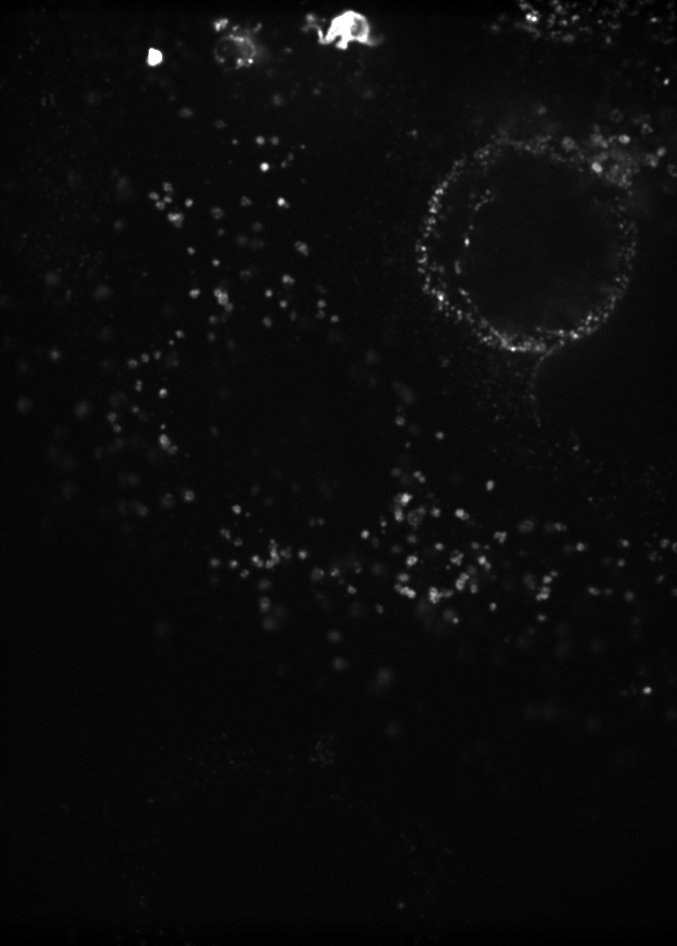

Supplement: Supplementary file 15 — Source data Fig. 6-2 [file 44318_2026_705_MOESM15_ESM.zip › Figure 6-2/I/STARD3NL_deltaFFAT_NT/20220823_STARD3NLdeltaFFAT_NT_5_w1SPI 491 GFP.TIF]

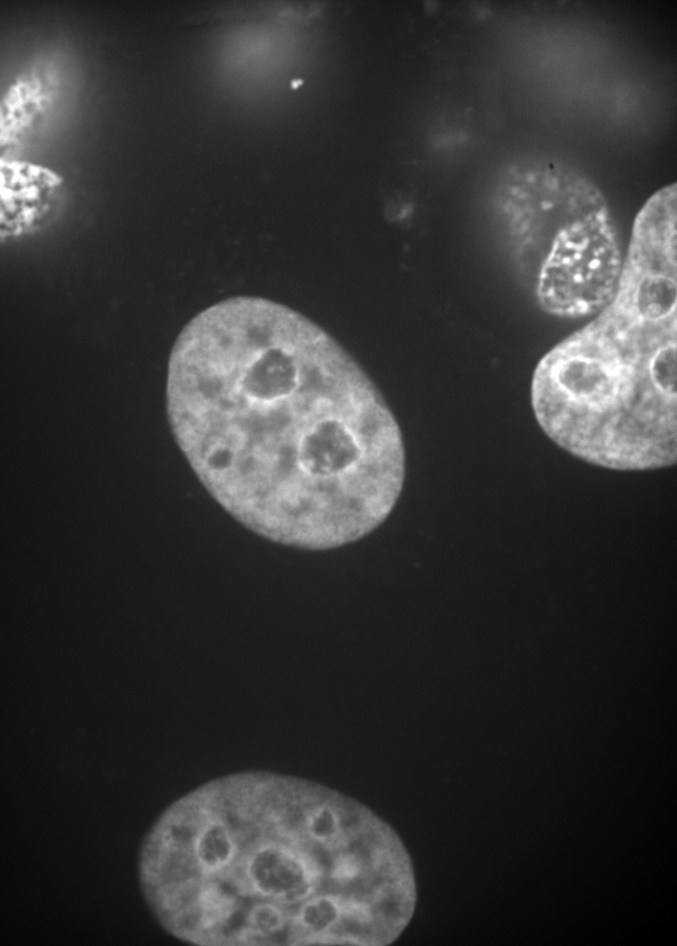

Supplement: Supplementary file 15 — Source data Fig. 6-2 [file 44318_2026_705_MOESM15_ESM.zip › Figure 6-2/I/STARD3NL_deltaFFAT_NT/20220823_STARD3NLdeltaFFAT_NT_5_w2SPI 405 DAPI.TIF]

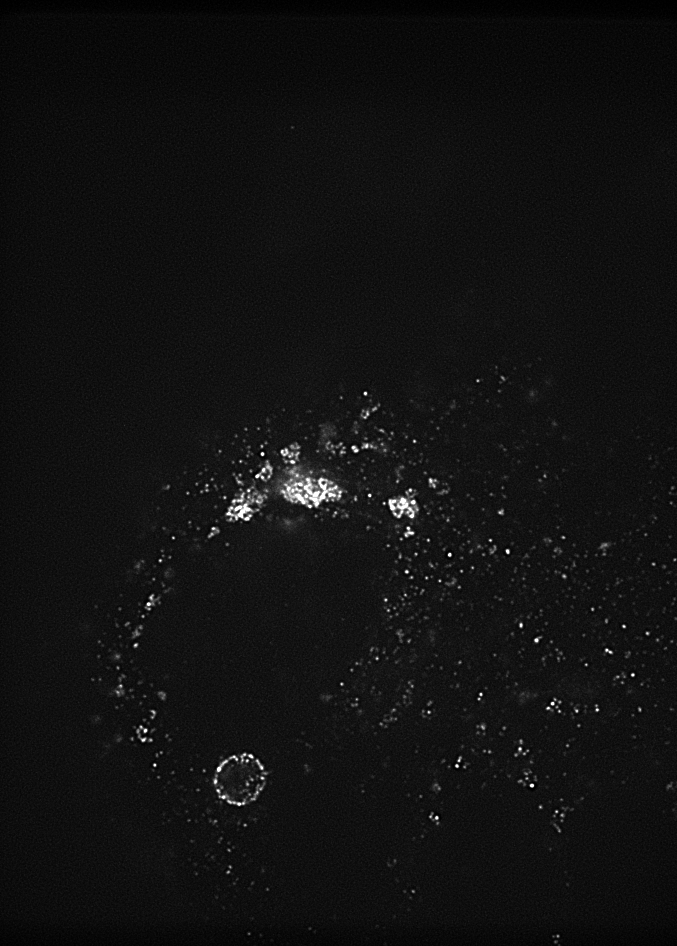

Supplement: Supplementary file 15 — Source data Fig. 6-2 [file 44318_2026_705_MOESM15_ESM.zip › Figure 6-2/J/STARD3NL-START_CHIR99021/20220825_STARD3NLSTART_GSK3i_3_SR_w1SPI 491 GFP.TIF]

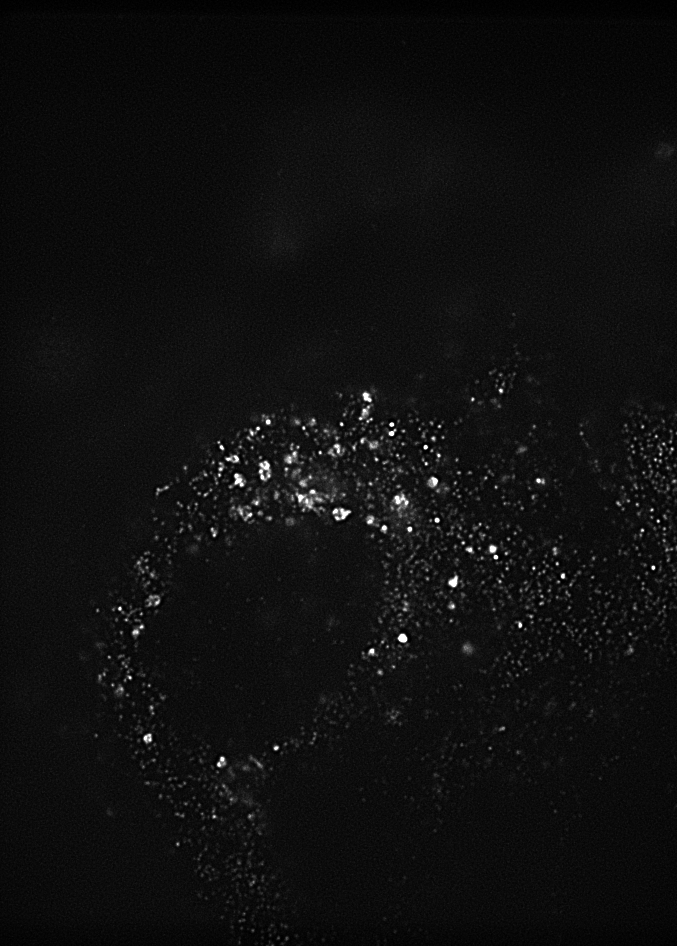

Supplement: Supplementary file 15 — Source data Fig. 6-2 [file 44318_2026_705_MOESM15_ESM.zip › Figure 6-2/J/STARD3NL-START_CHIR99021/20220825_STARD3NLSTART_GSK3i_3_SR_w2SPI 561 mCherry.TIF]

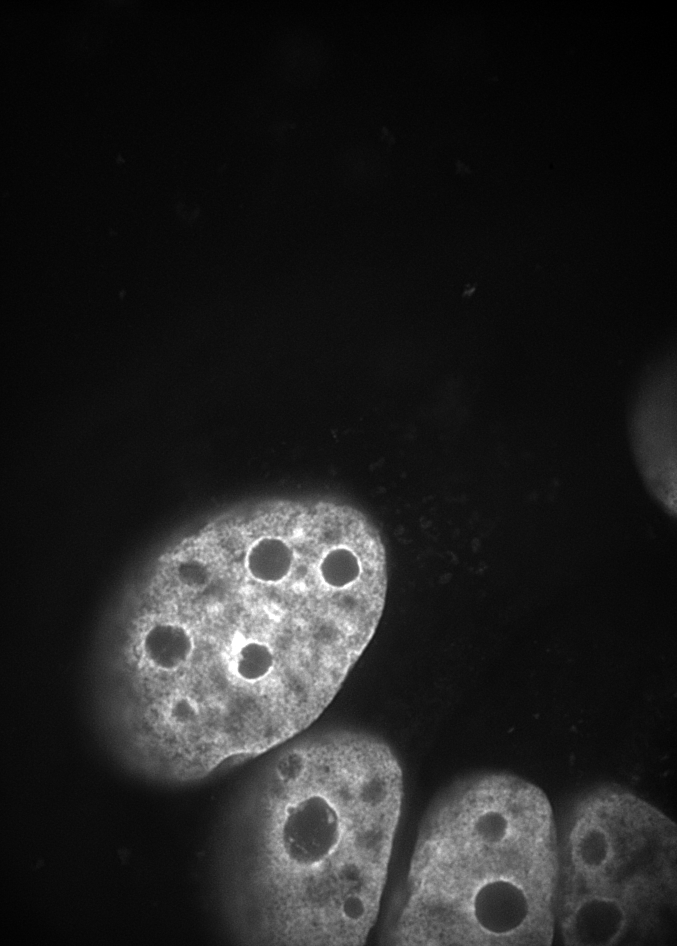

Supplement: Supplementary file 15 — Source data Fig. 6-2 [file 44318_2026_705_MOESM15_ESM.zip › Figure 6-2/J/STARD3NL-START_CHIR99021/20220825_STARD3NLSTART_GSK3i_3_SR_w3SPI 405 DAPI.TIF]

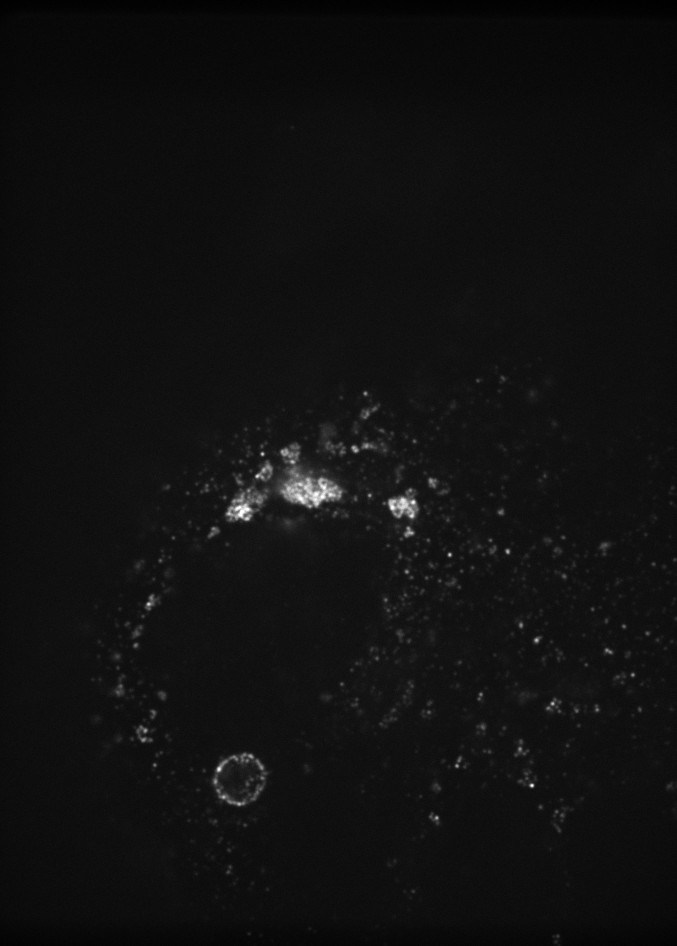

Supplement: Supplementary file 15 — Source data Fig. 6-2 [file 44318_2026_705_MOESM15_ESM.zip › Figure 6-2/J/STARD3NL-START_CHIR99021/20220825_STARD3NLSTART_GSK3i_3_w1SPI 491 GFP.TIF]

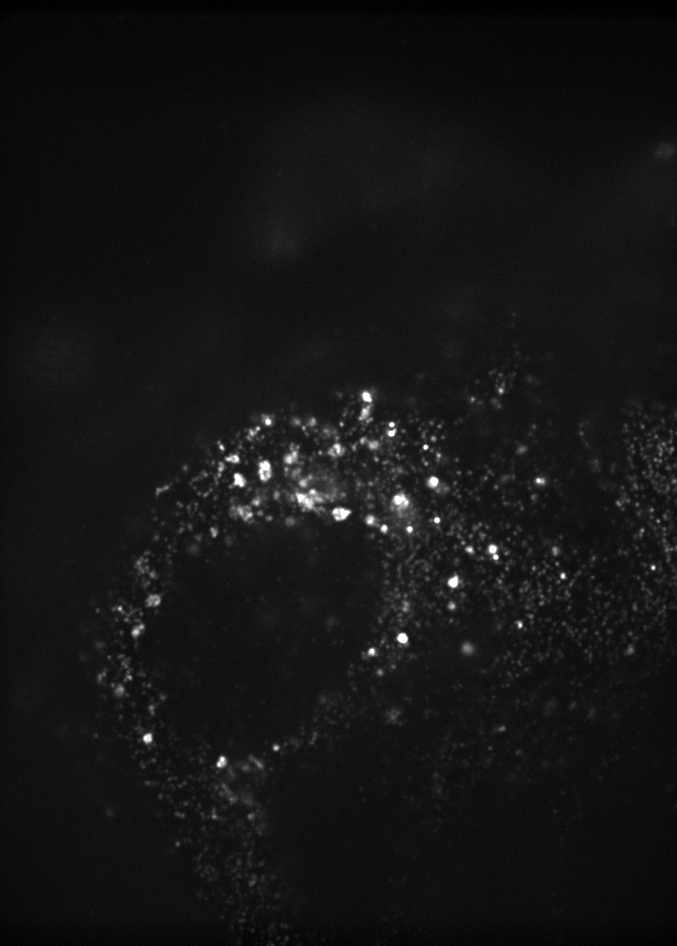

Supplement: Supplementary file 15 — Source data Fig. 6-2 [file 44318_2026_705_MOESM15_ESM.zip › Figure 6-2/J/STARD3NL-START_CHIR99021/20220825_STARD3NLSTART_GSK3i_3_w2SPI 561 mCherry.TIF]

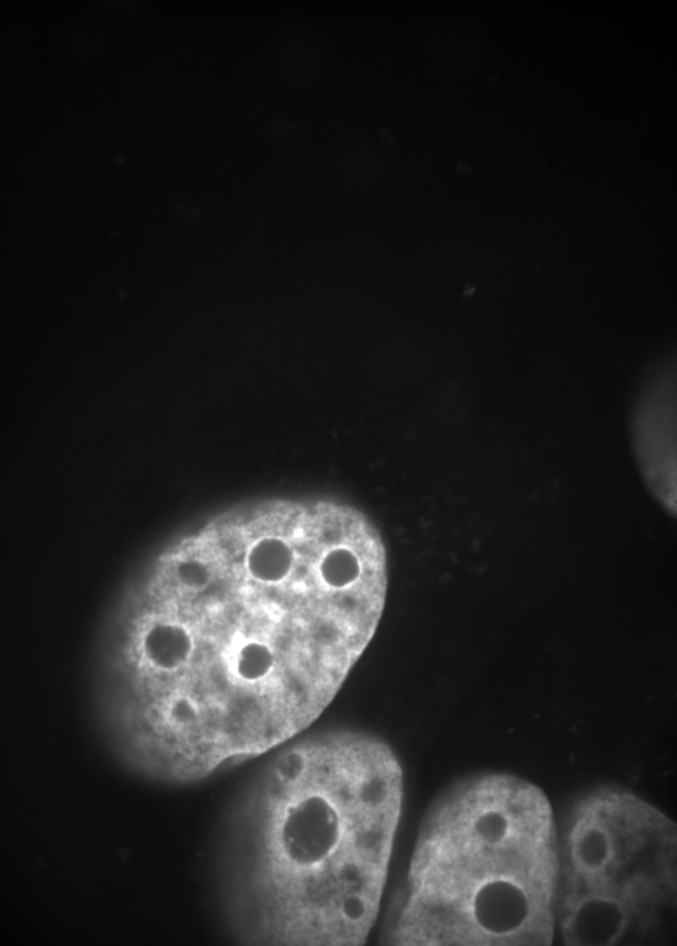

Supplement: Supplementary file 15 — Source data Fig. 6-2 [file 44318_2026_705_MOESM15_ESM.zip › Figure 6-2/J/STARD3NL-START_CHIR99021/20220825_STARD3NLSTART_GSK3i_3_w3SPI 405 DAPI.TIF]

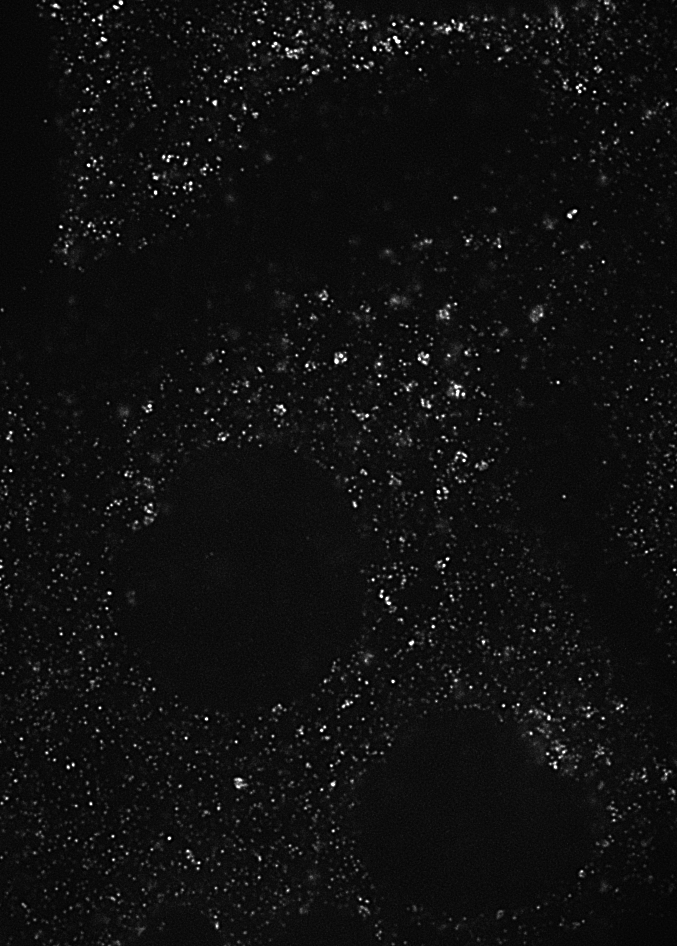

Supplement: Supplementary file 15 — Source data Fig. 6-2 [file 44318_2026_705_MOESM15_ESM.zip › Figure 6-2/J/STARD3NL-START_NT/20220825_STARD3NLSTART_NT_2_SR_w1SPI 491 GFP.TIF]

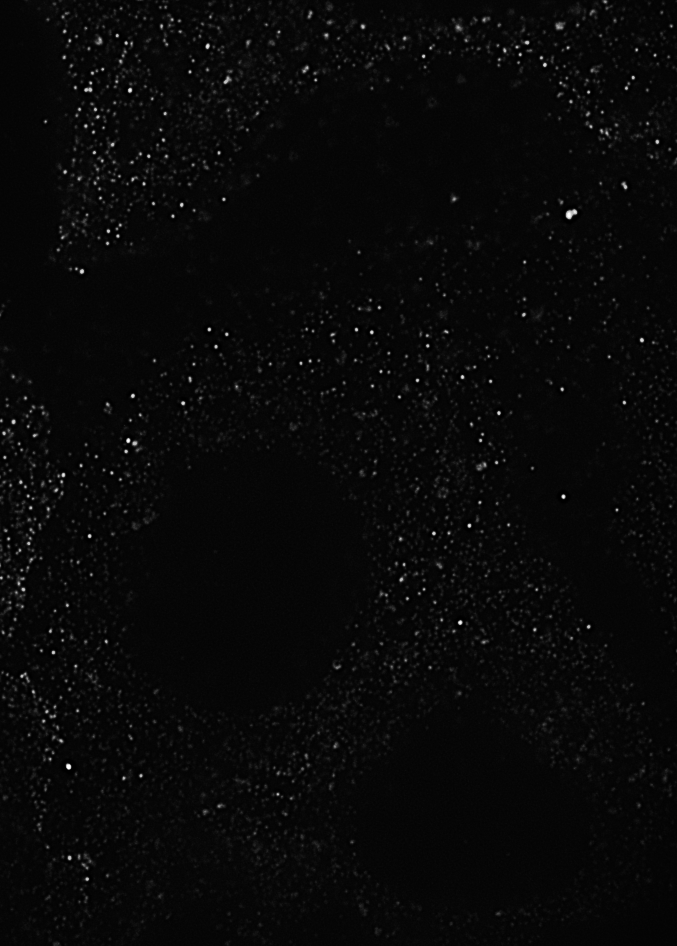

Supplement: Supplementary file 15 — Source data Fig. 6-2 [file 44318_2026_705_MOESM15_ESM.zip › Figure 6-2/J/STARD3NL-START_NT/20220825_STARD3NLSTART_NT_2_SR_w2SPI 561 mCherry.TIF]

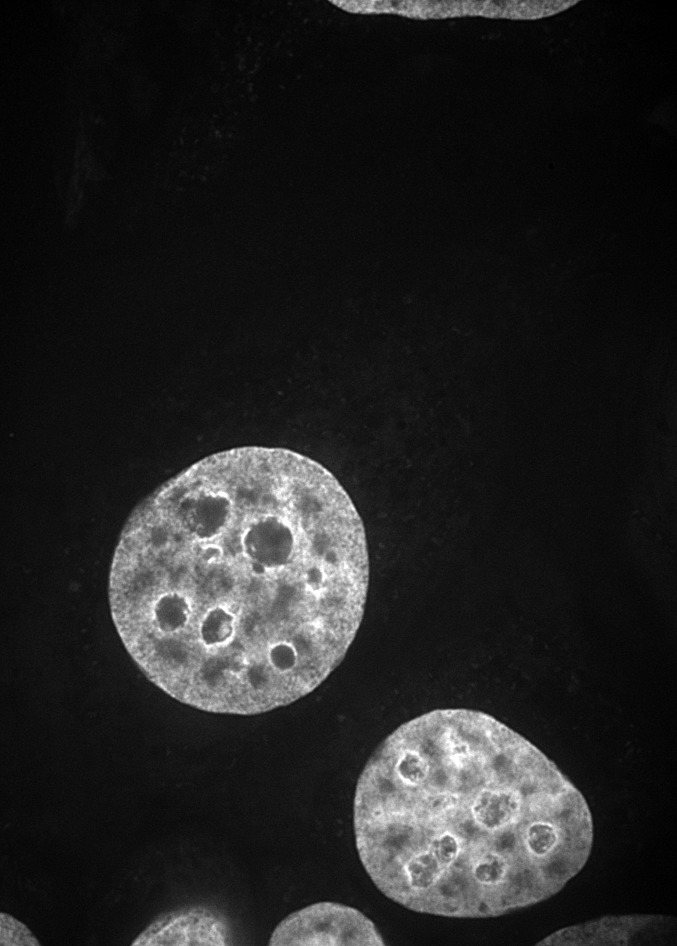

Supplement: Supplementary file 15 — Source data Fig. 6-2 [file 44318_2026_705_MOESM15_ESM.zip › Figure 6-2/J/STARD3NL-START_NT/20220825_STARD3NLSTART_NT_2_SR_w3SPI 405 DAPI.TIF]

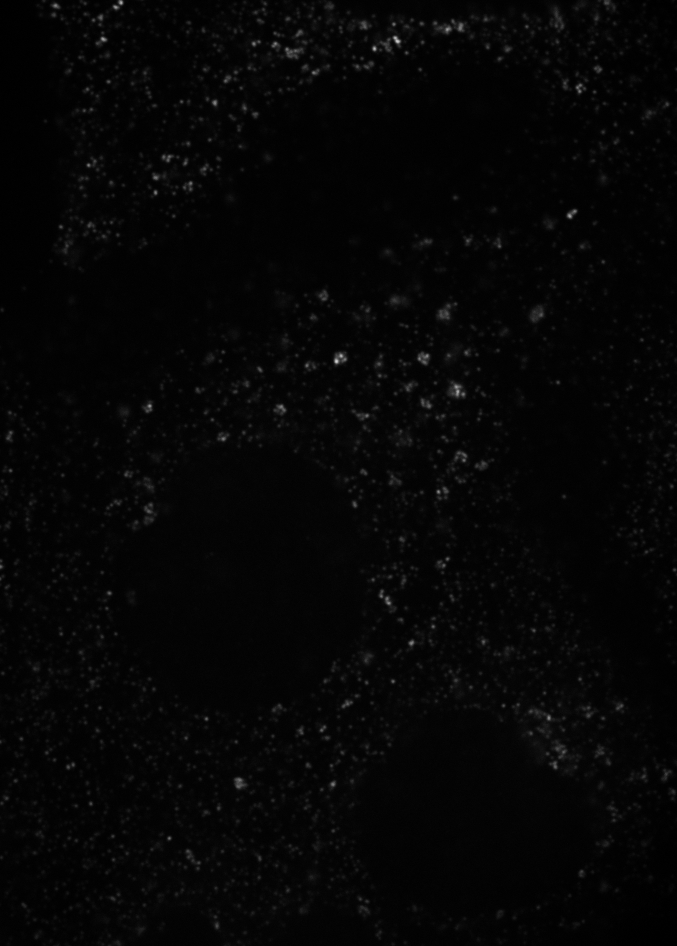

Supplement: Supplementary file 15 — Source data Fig. 6-2 [file 44318_2026_705_MOESM15_ESM.zip › Figure 6-2/J/STARD3NL-START_NT/20220825_STARD3NLSTART_NT_2_w1SPI 491 GFP.TIF]

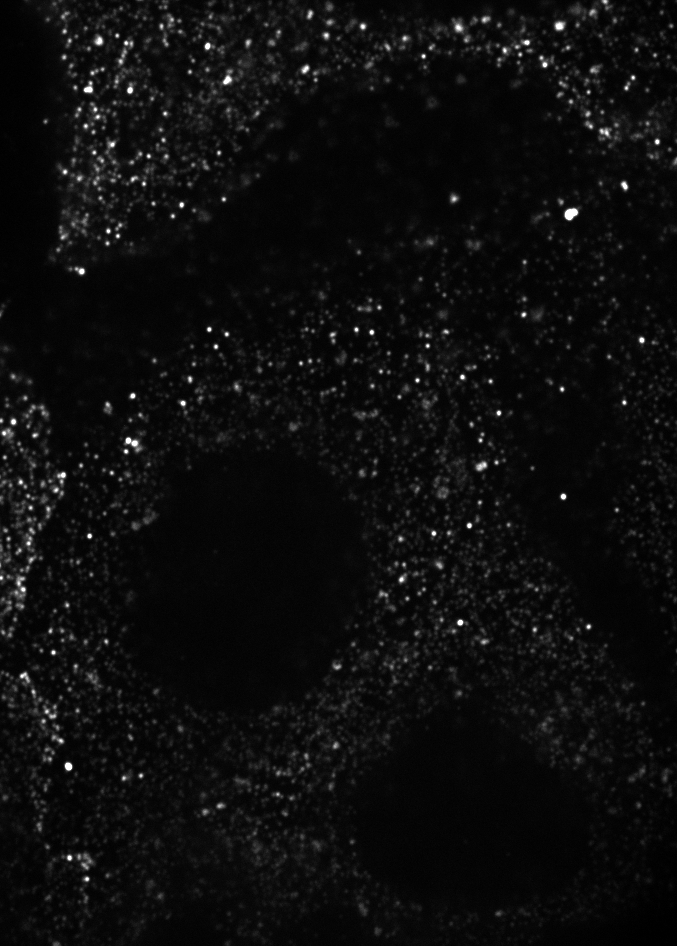

Supplement: Supplementary file 15 — Source data Fig. 6-2 [file 44318_2026_705_MOESM15_ESM.zip › Figure 6-2/J/STARD3NL-START_NT/20220825_STARD3NLSTART_NT_2_w2SPI 561 mCherry.TIF]

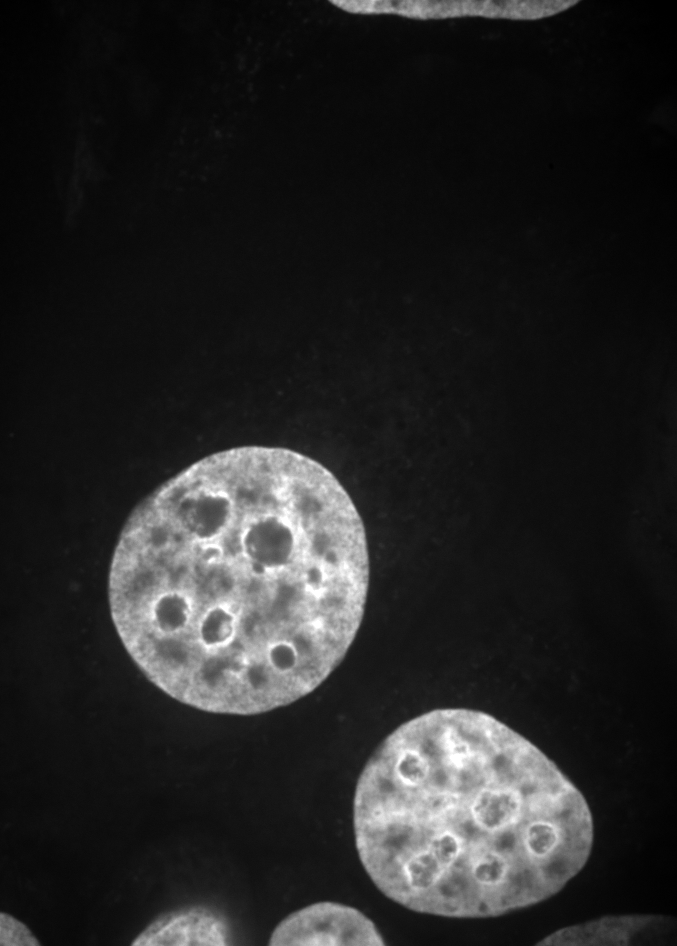

Supplement: Supplementary file 15 — Source data Fig. 6-2 [file 44318_2026_705_MOESM15_ESM.zip › Figure 6-2/J/STARD3NL-START_NT/20220825_STARD3NLSTART_NT_2_w3SPI 405 DAPI.TIF]

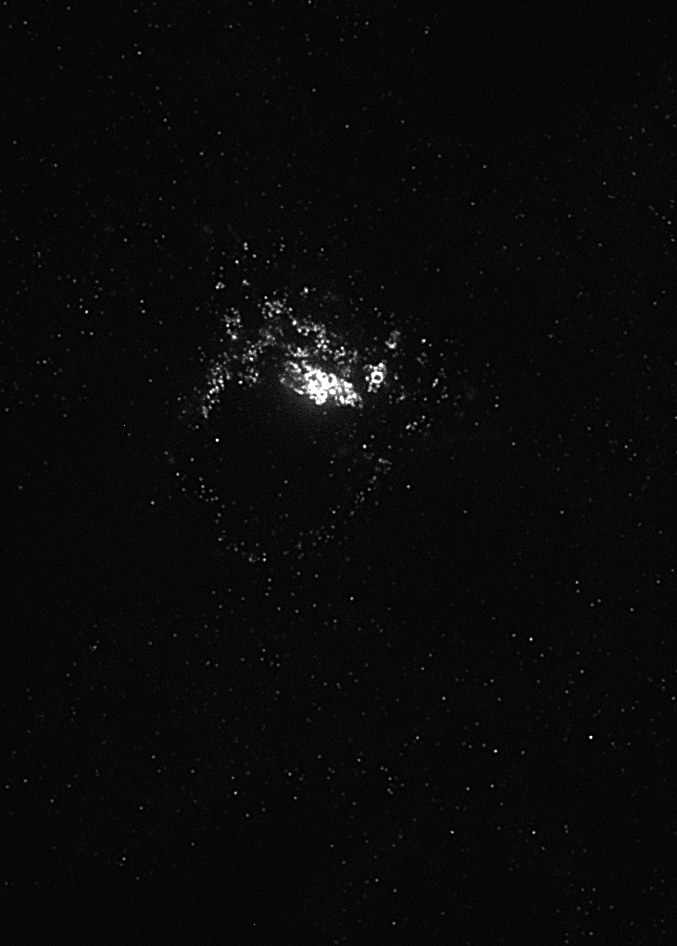

Supplement: Supplementary file 16 — Source data Fig. 6-3 [file 44318_2026_705_MOESM16_ESM.zip › Figure 6-3/M/TMEM192-START_CHIR99021/20250228_MCF7STARD3TMEMS209ASTART_CHIR_1_SR_w1SPI 491 GFP.TIF]

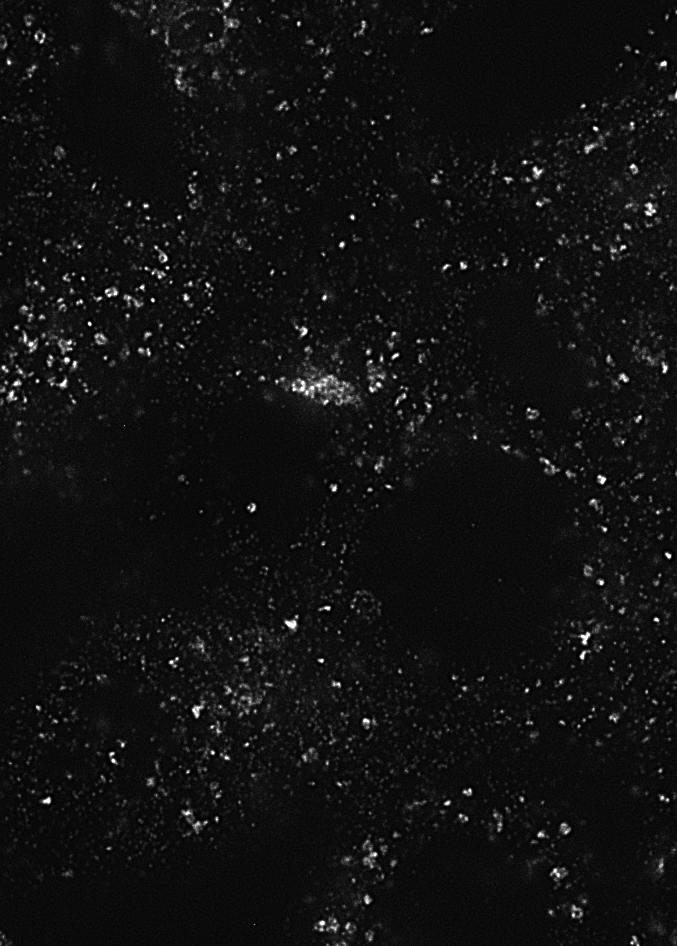

Supplement: Supplementary file 16 — Source data Fig. 6-3 [file 44318_2026_705_MOESM16_ESM.zip › Figure 6-3/M/TMEM192-START_CHIR99021/20250228_MCF7STARD3TMEMS209ASTART_CHIR_1_SR_w2SPI 561 mCherry.TIF]

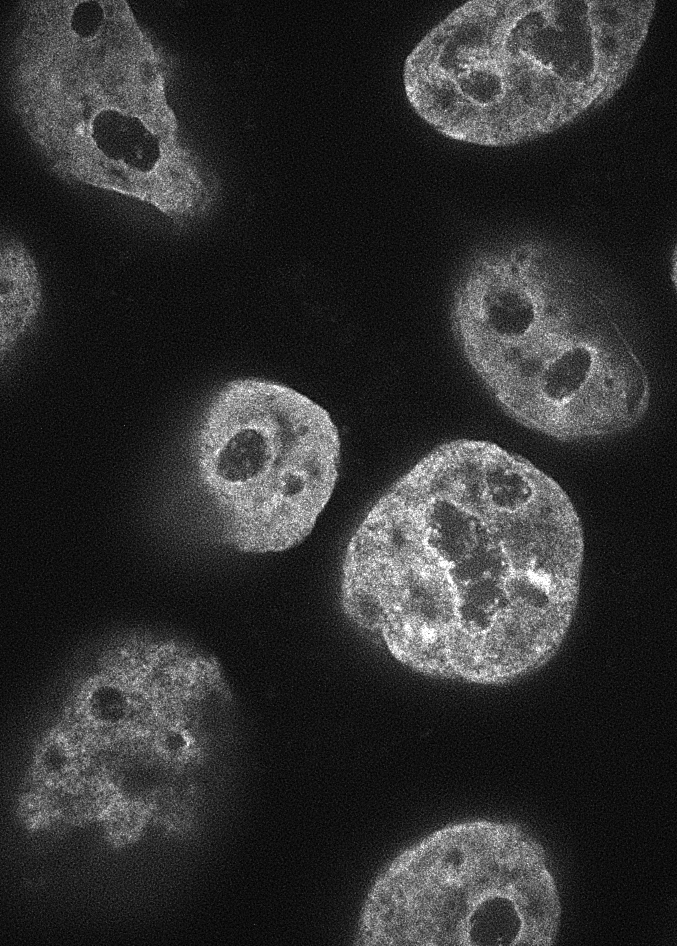

Supplement: Supplementary file 16 — Source data Fig. 6-3 [file 44318_2026_705_MOESM16_ESM.zip › Figure 6-3/M/TMEM192-START_CHIR99021/20250228_MCF7STARD3TMEMS209ASTART_CHIR_1_SR_w3SPI 405 DAPI.TIF]

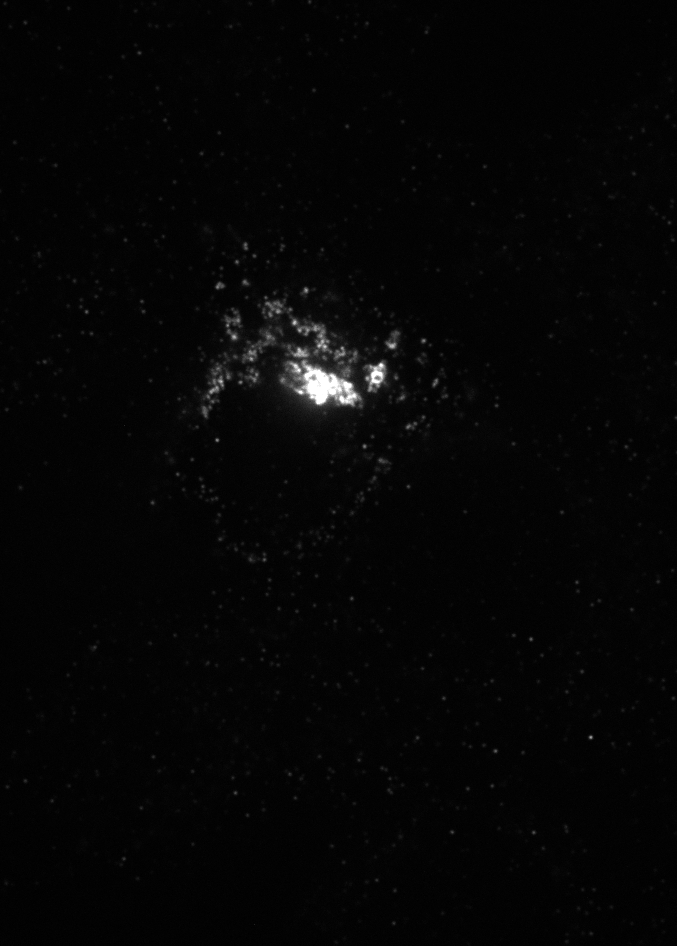

Supplement: Supplementary file 16 — Source data Fig. 6-3 [file 44318_2026_705_MOESM16_ESM.zip › Figure 6-3/M/TMEM192-START_CHIR99021/20250228_MCF7STARD3TMEMS209ASTART_CHIR_1_w1SPI 491 GFP.TIF]

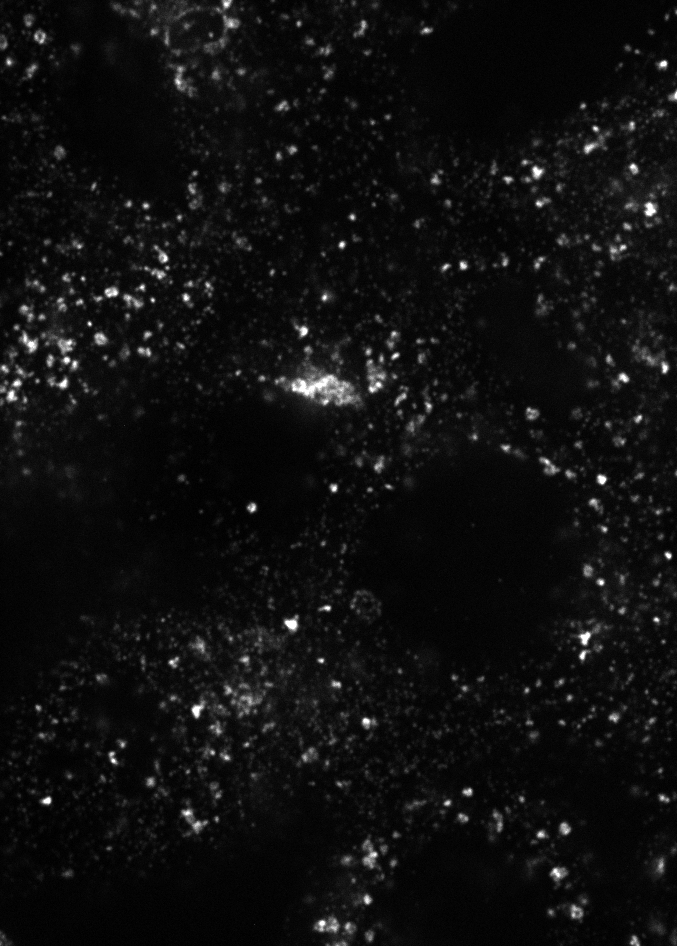

Supplement: Supplementary file 16 — Source data Fig. 6-3 [file 44318_2026_705_MOESM16_ESM.zip › Figure 6-3/M/TMEM192-START_CHIR99021/20250228_MCF7STARD3TMEMS209ASTART_CHIR_1_w2SPI 561 mCherry.TIF]

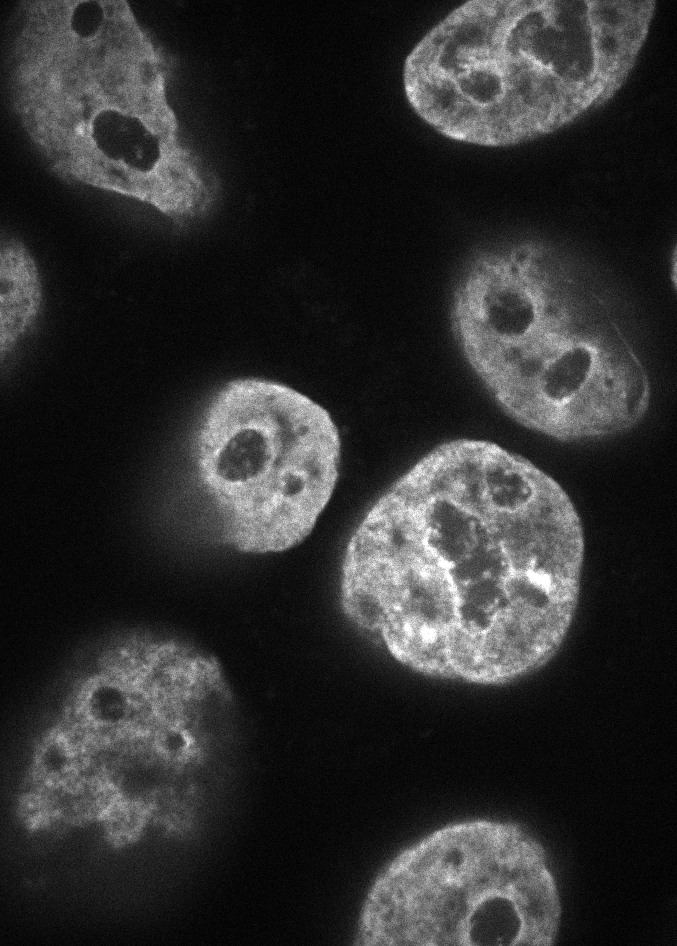

Supplement: Supplementary file 16 — Source data Fig. 6-3 [file 44318_2026_705_MOESM16_ESM.zip › Figure 6-3/M/TMEM192-START_CHIR99021/20250228_MCF7STARD3TMEMS209ASTART_CHIR_1_w3SPI 405 DAPI.TIF]

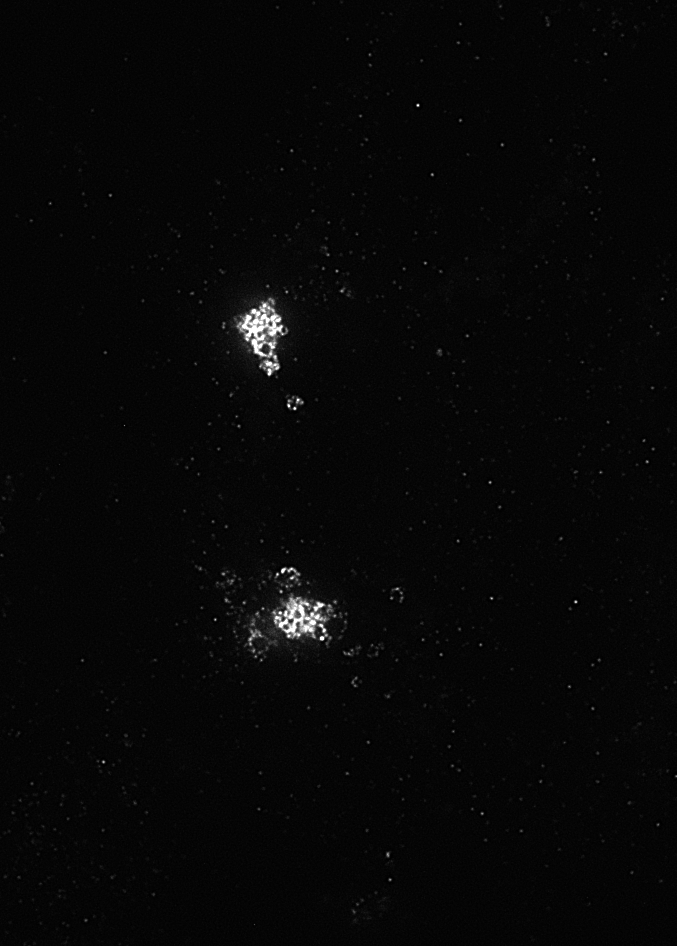

Supplement: Supplementary file 16 — Source data Fig. 6-3 [file 44318_2026_705_MOESM16_ESM.zip › Figure 6-3/M/TMEM192-START_NT/20250210_STARD3TMEM_NT_3_SR_w1SPI 491 GFP.TIF]

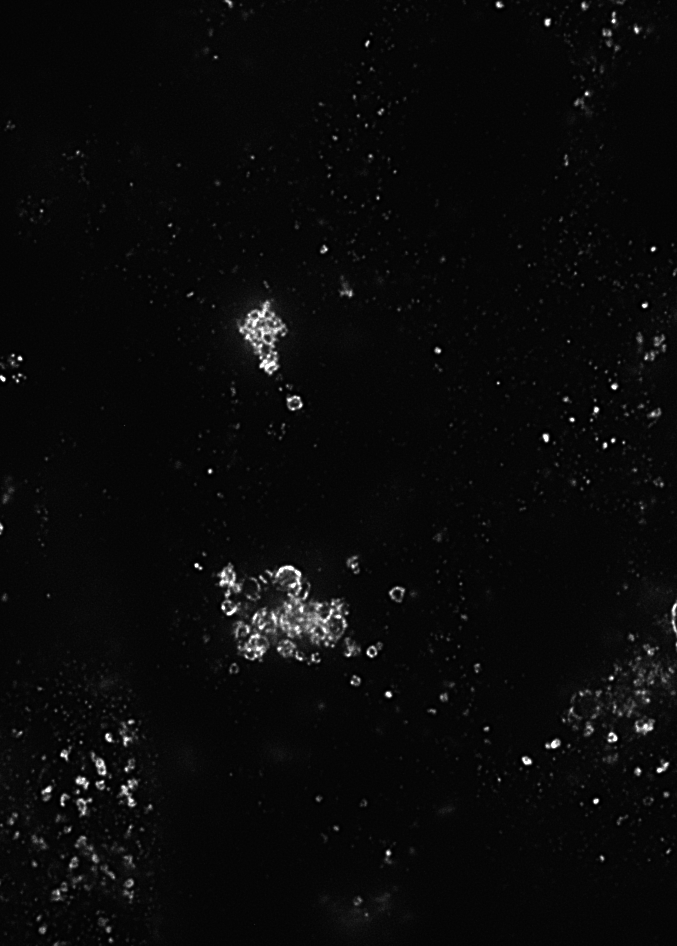

Supplement: Supplementary file 16 — Source data Fig. 6-3 [file 44318_2026_705_MOESM16_ESM.zip › Figure 6-3/M/TMEM192-START_NT/20250210_STARD3TMEM_NT_3_SR_w2SPI 561 mCherry.TIF]

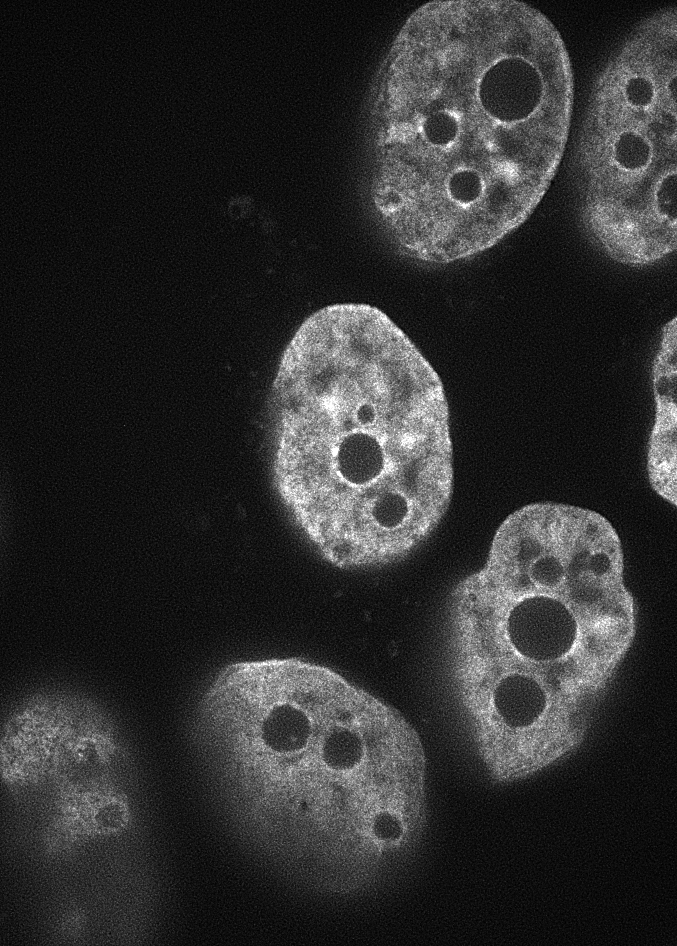

Supplement: Supplementary file 16 — Source data Fig. 6-3 [file 44318_2026_705_MOESM16_ESM.zip › Figure 6-3/M/TMEM192-START_NT/20250210_STARD3TMEM_NT_3_SR_w3SPI 405 DAPI.TIF]

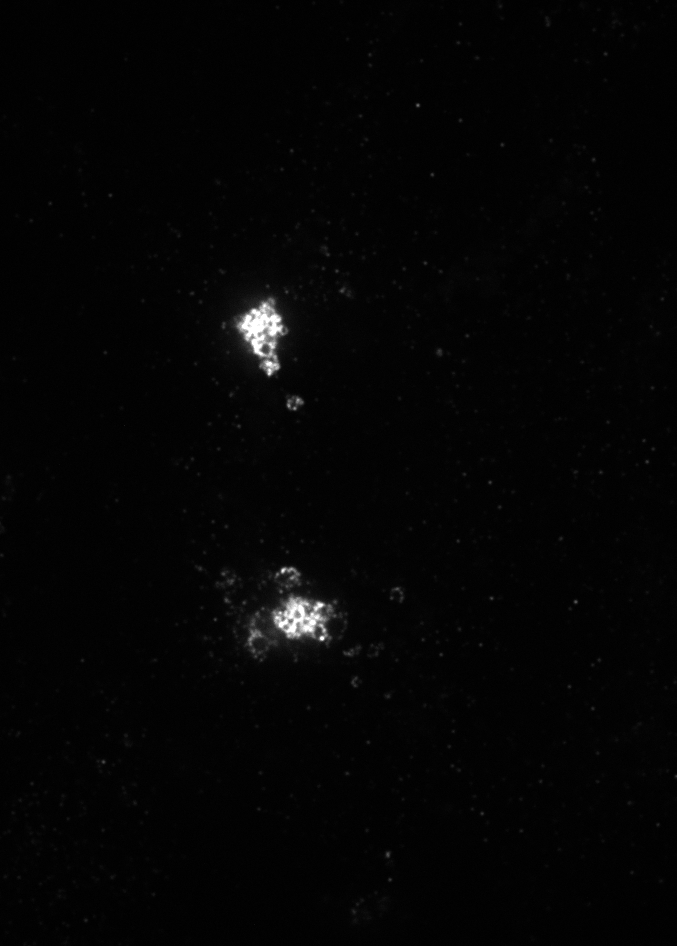

Supplement: Supplementary file 16 — Source data Fig. 6-3 [file 44318_2026_705_MOESM16_ESM.zip › Figure 6-3/M/TMEM192-START_NT/20250210_STARD3TMEM_NT_3_w1SPI 491 GFP.TIF]

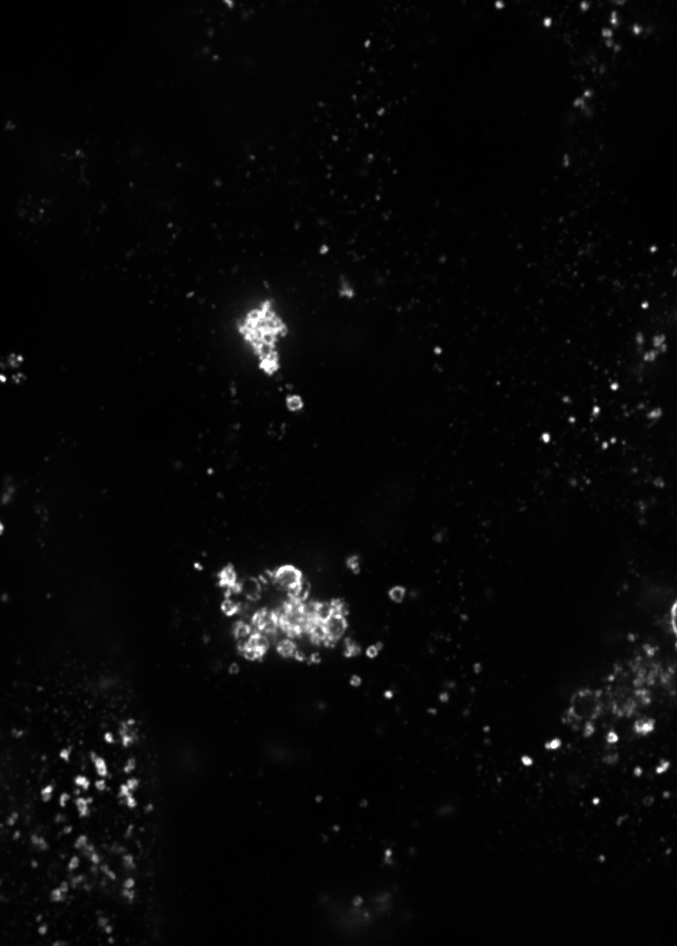

Supplement: Supplementary file 16 — Source data Fig. 6-3 [file 44318_2026_705_MOESM16_ESM.zip › Figure 6-3/M/TMEM192-START_NT/20250210_STARD3TMEM_NT_3_w2SPI 561 mCherry.TIF]

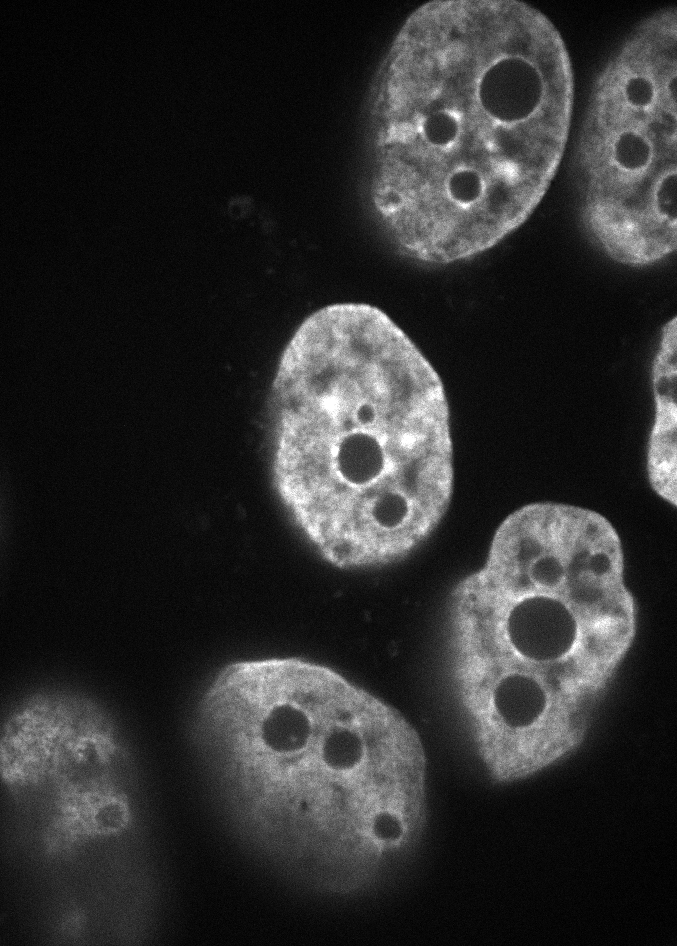

Supplement: Supplementary file 16 — Source data Fig. 6-3 [file 44318_2026_705_MOESM16_ESM.zip › Figure 6-3/M/TMEM192-START_NT/20250210_STARD3TMEM_NT_3_w3SPI 405 DAPI.TIF]

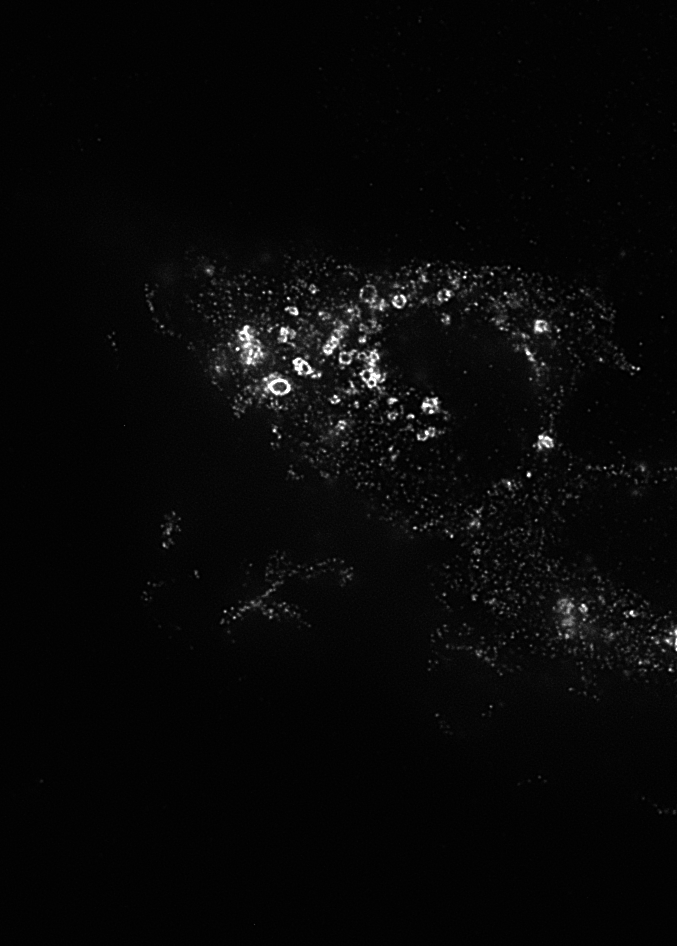

Supplement: Supplementary file 16 — Source data Fig. 6-3 [file 44318_2026_705_MOESM16_ESM.zip › Figure 6-3/N/Lyso-START_CHIR99021/20250228_MCF7STARD3LAMTOR_CHIR_5_SR_w1SPI 491 GFP.tif]

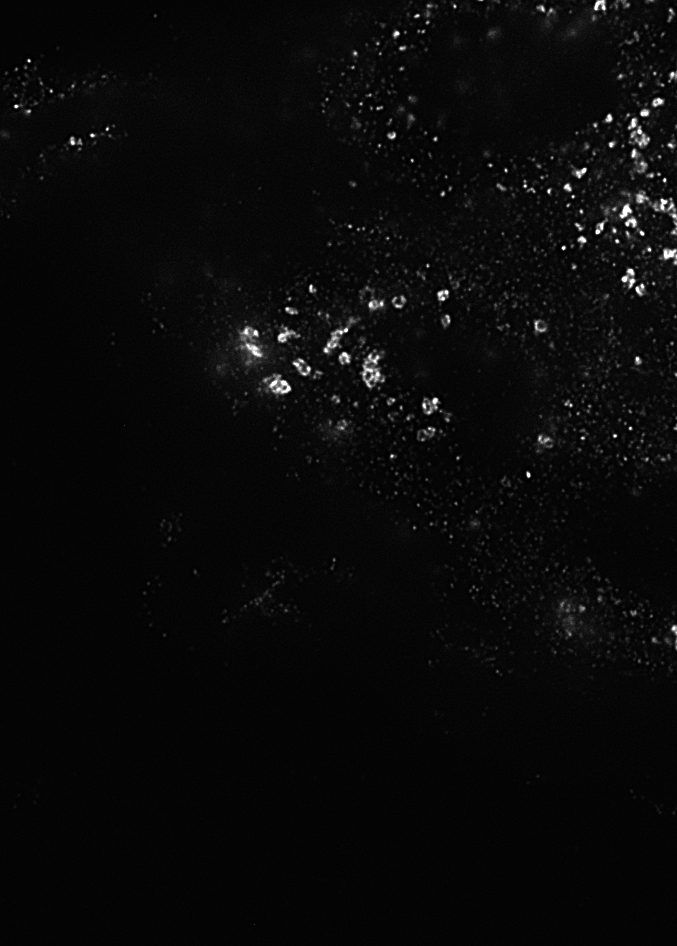

Supplement: Supplementary file 16 — Source data Fig. 6-3 [file 44318_2026_705_MOESM16_ESM.zip › Figure 6-3/N/Lyso-START_CHIR99021/20250228_MCF7STARD3LAMTOR_CHIR_5_SR_w2SPI 561 mCherry.tif]

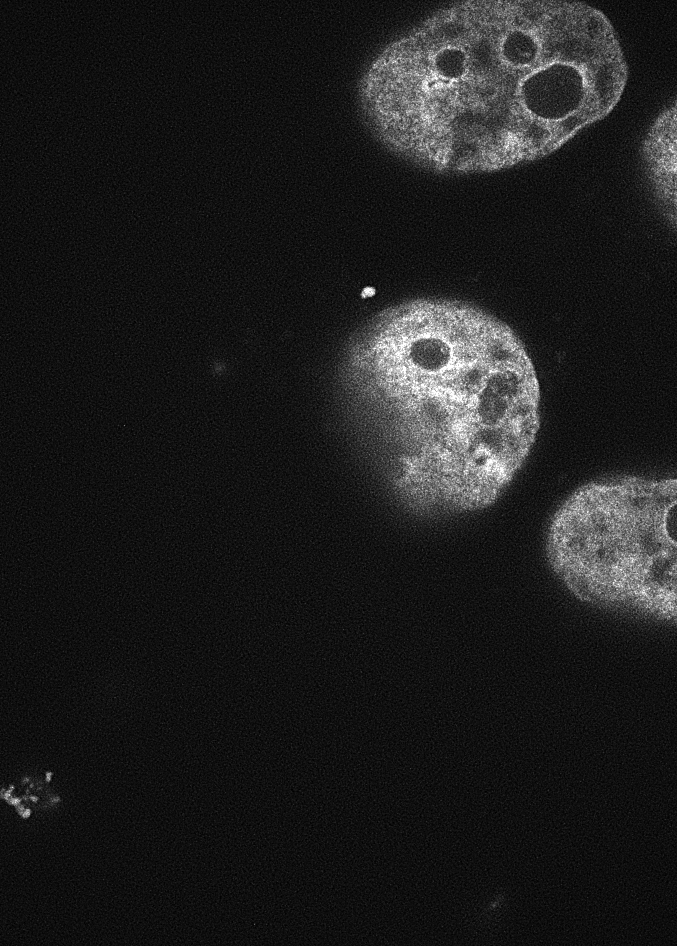

Supplement: Supplementary file 16 — Source data Fig. 6-3 [file 44318_2026_705_MOESM16_ESM.zip › Figure 6-3/N/Lyso-START_CHIR99021/20250228_MCF7STARD3LAMTOR_CHIR_5_SR_w3SPI 405 DAPI.TIF]

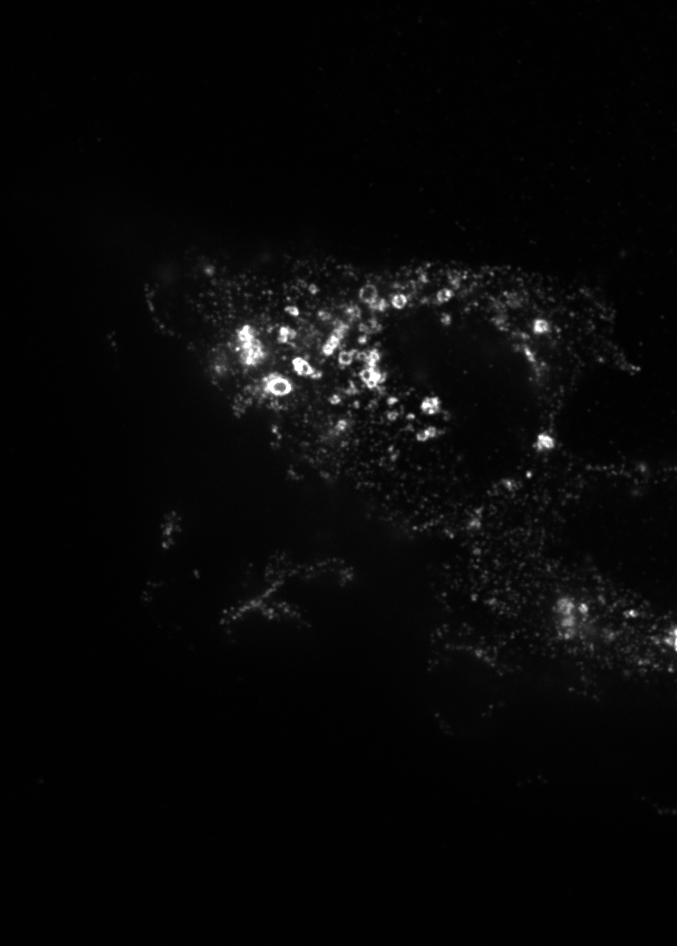

Supplement: Supplementary file 16 — Source data Fig. 6-3 [file 44318_2026_705_MOESM16_ESM.zip › Figure 6-3/N/Lyso-START_CHIR99021/20250228_MCF7STARD3LAMTOR_CHIR_5_w1SPI 491 GFP.TIF]

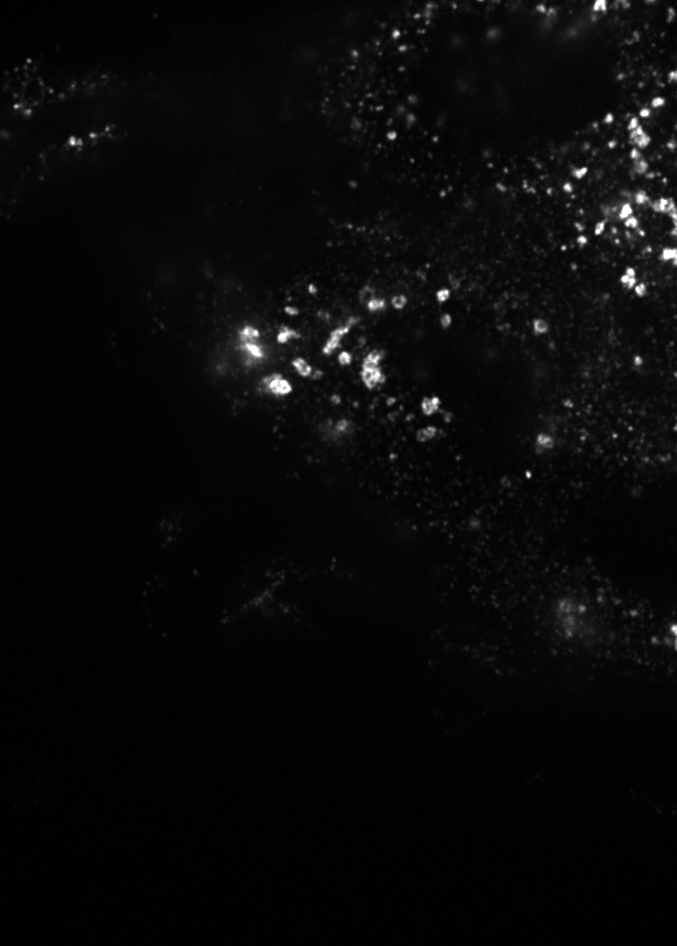

Supplement: Supplementary file 16 — Source data Fig. 6-3 [file 44318_2026_705_MOESM16_ESM.zip › Figure 6-3/N/Lyso-START_CHIR99021/20250228_MCF7STARD3LAMTOR_CHIR_5_w2SPI 561 mCherry.TIF]

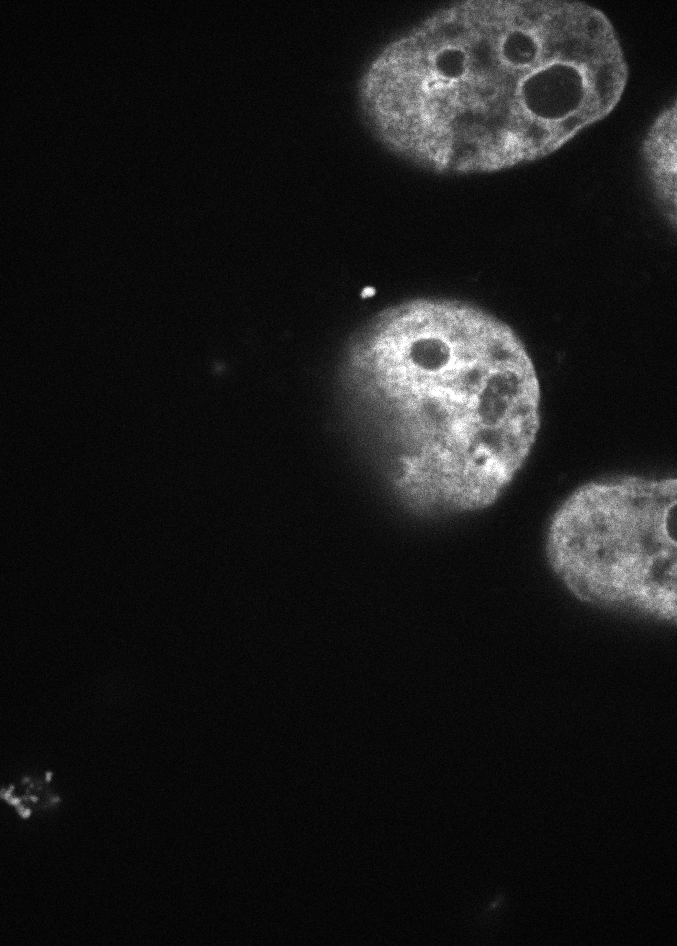

Supplement: Supplementary file 16 — Source data Fig. 6-3 [file 44318_2026_705_MOESM16_ESM.zip › Figure 6-3/N/Lyso-START_CHIR99021/20250228_MCF7STARD3LAMTOR_CHIR_5_w3SPI 405 DAPI.TIF]

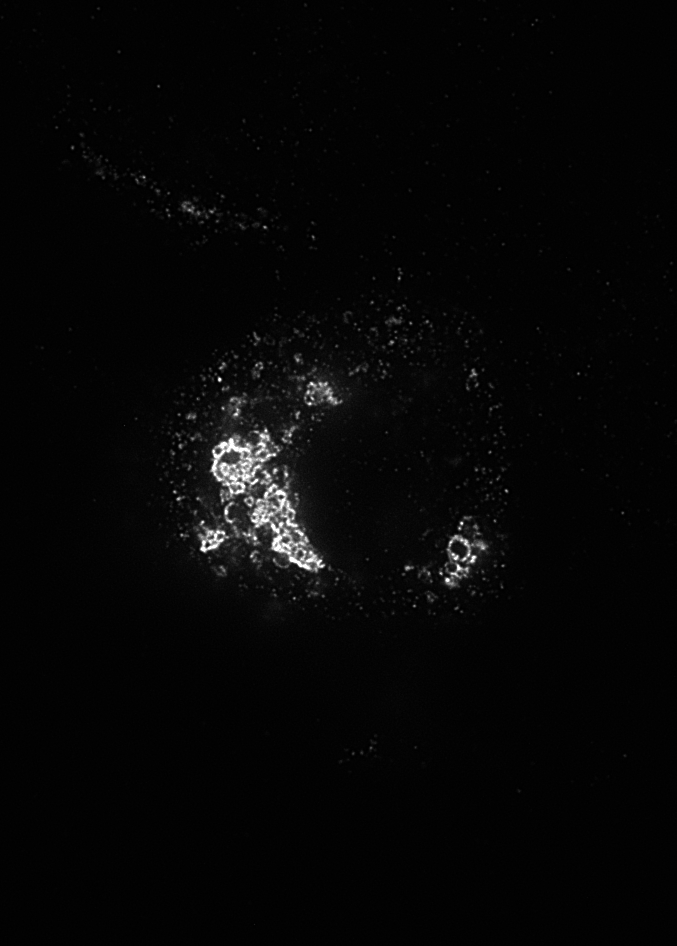

Supplement: Supplementary file 16 — Source data Fig. 6-3 [file 44318_2026_705_MOESM16_ESM.zip › Figure 6-3/N/Lyso-START_NT/20250210_LAMTORSTART605_NT_4_SR_w1SPI 491 GFP.TIF]

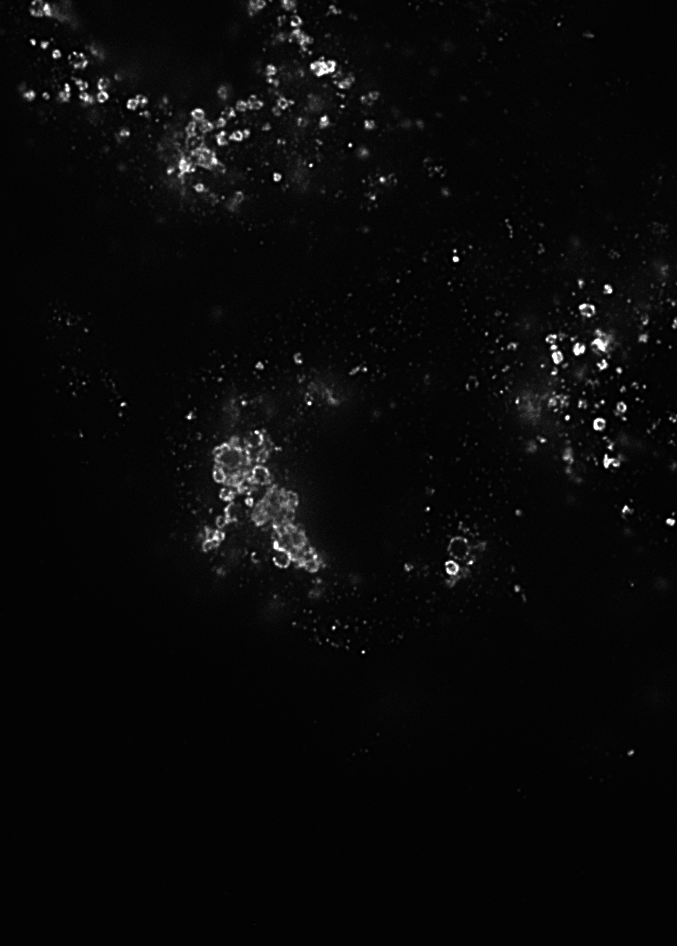

Supplement: Supplementary file 16 — Source data Fig. 6-3 [file 44318_2026_705_MOESM16_ESM.zip › Figure 6-3/N/Lyso-START_NT/20250210_LAMTORSTART605_NT_4_SR_w2SPI 561 mCherry.TIF]

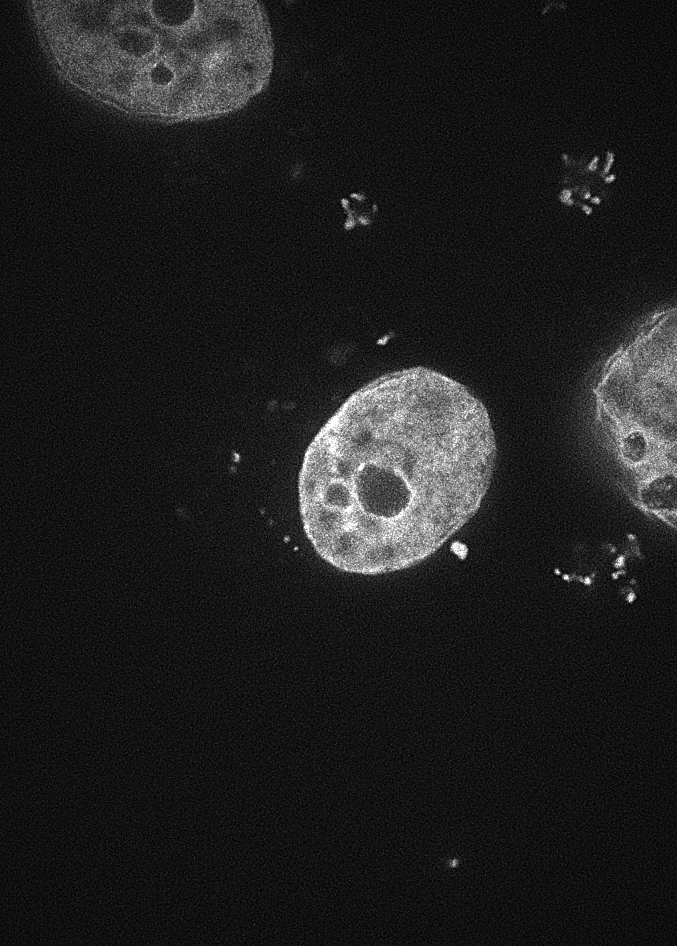

Supplement: Supplementary file 16 — Source data Fig. 6-3 [file 44318_2026_705_MOESM16_ESM.zip › Figure 6-3/N/Lyso-START_NT/20250210_LAMTORSTART605_NT_4_SR_w3SPI 405 DAPI.TIF]

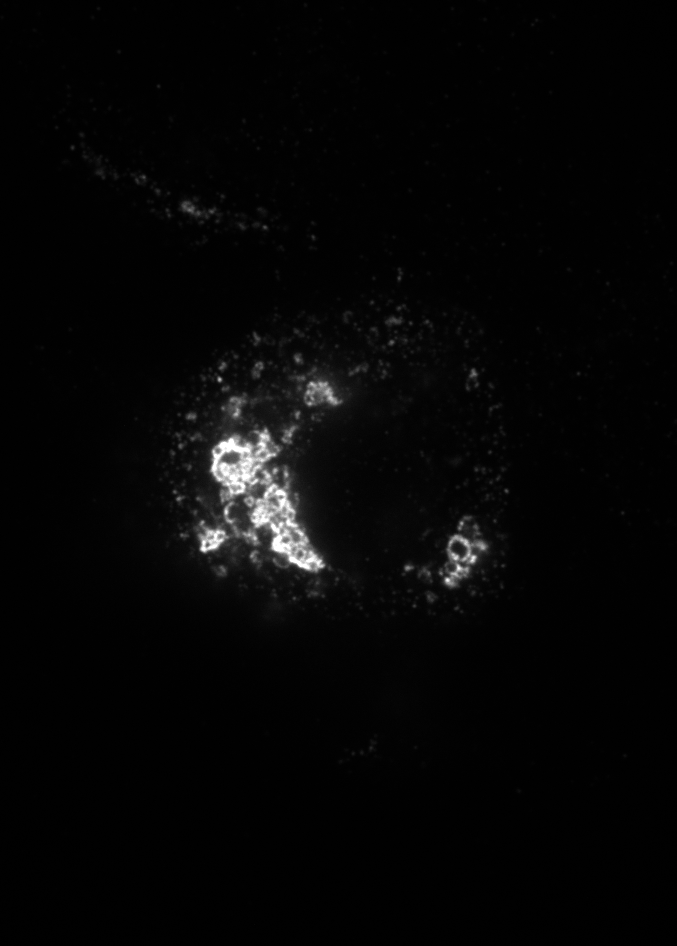

Supplement: Supplementary file 16 — Source data Fig. 6-3 [file 44318_2026_705_MOESM16_ESM.zip › Figure 6-3/N/Lyso-START_NT/20250210_LAMTORSTART605_NT_4_w1SPI 491 GFP.TIF]

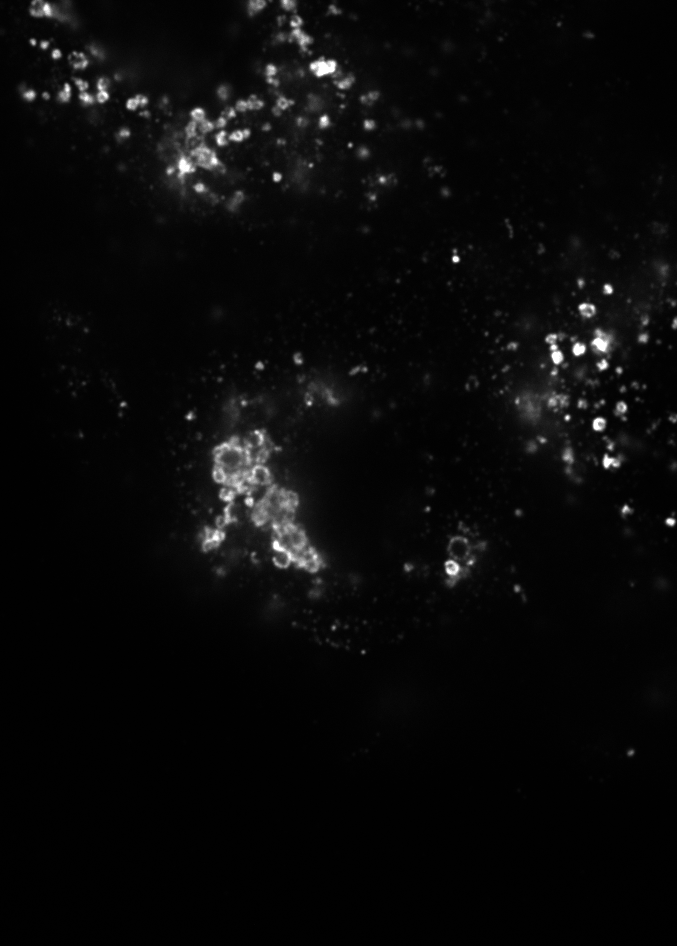

Supplement: Supplementary file 16 — Source data Fig. 6-3 [file 44318_2026_705_MOESM16_ESM.zip › Figure 6-3/N/Lyso-START_NT/20250210_LAMTORSTART605_NT_4_w2SPI 561 mCherry.TIF]

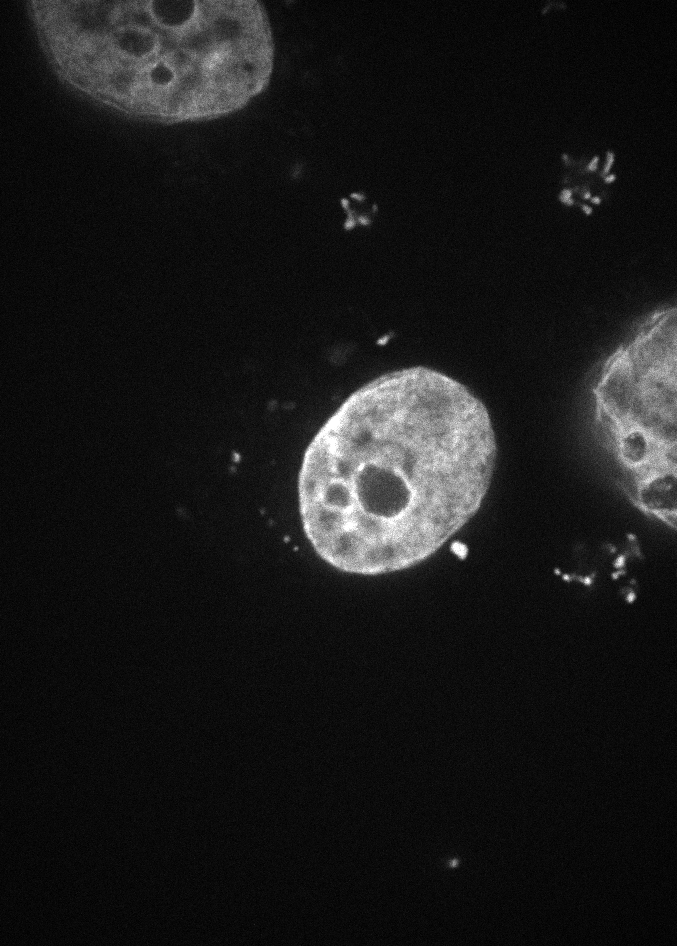

Supplement: Supplementary file 16 — Source data Fig. 6-3 [file 44318_2026_705_MOESM16_ESM.zip › Figure 6-3/N/Lyso-START_NT/20250210_LAMTORSTART605_NT_4_w3SPI 405 DAPI.TIF]

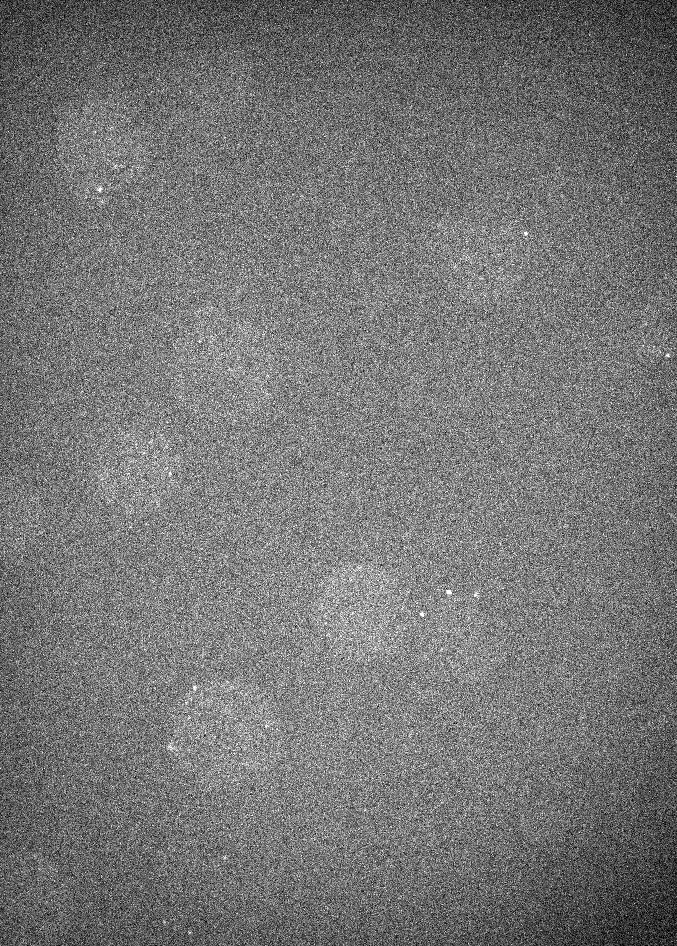

Supplement: Supplementary file 17 — Source data Fig. 7 [file 44318_2026_705_MOESM17_ESM.zip › Figure 7/D/PC_MSP/20230208_LipoPC_MSP_1_w1SPI 491 GFP.TIF]

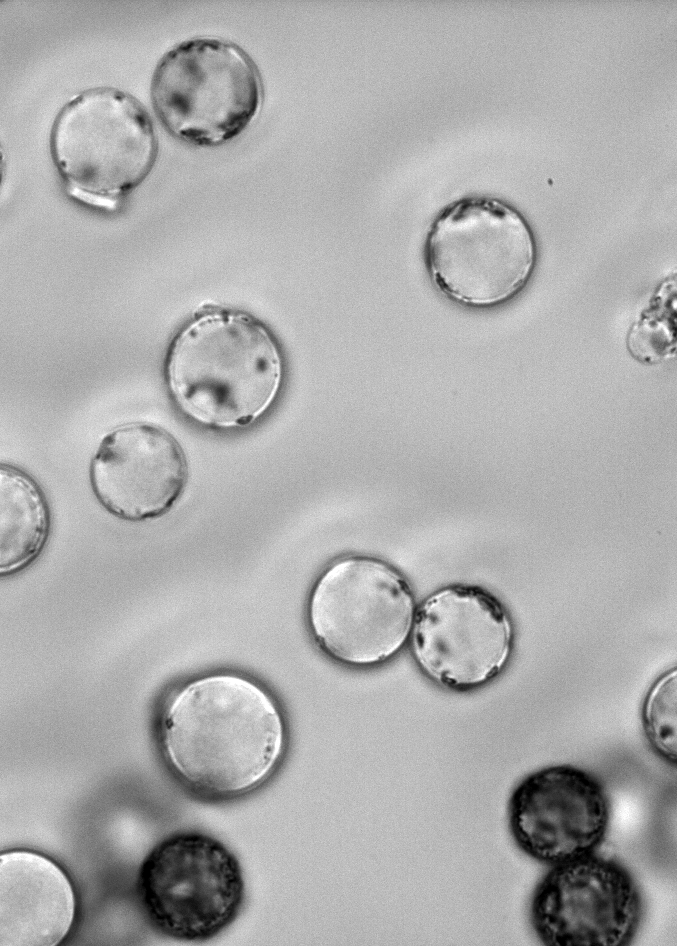

Supplement: Supplementary file 17 — Source data Fig. 7 [file 44318_2026_705_MOESM17_ESM.zip › Figure 7/D/PC_MSP/20230208_LipoPC_MSP_1_w2TRANS.TIF]

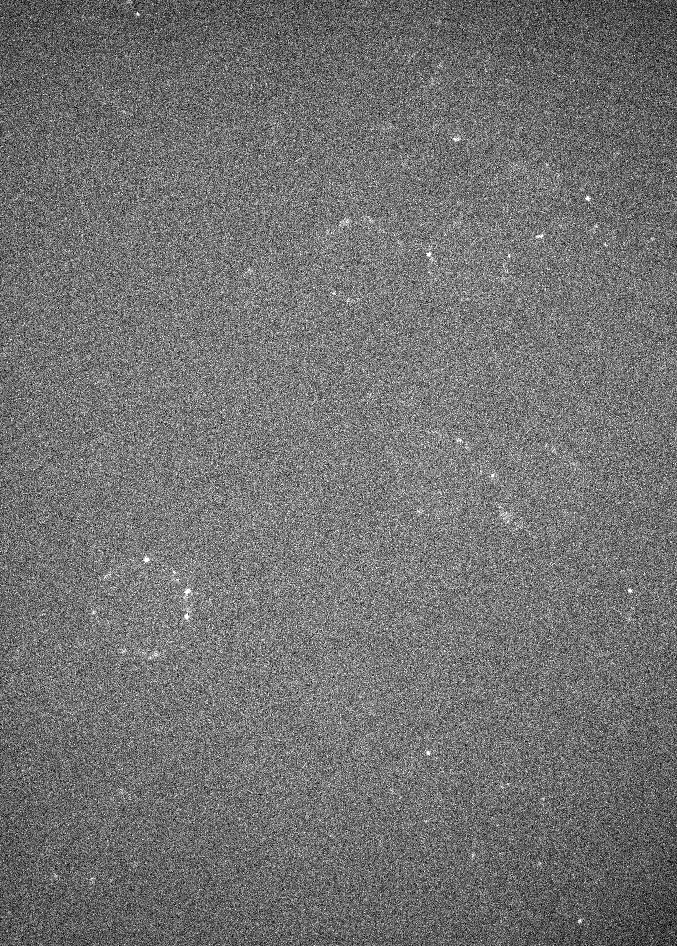

Supplement: Supplementary file 17 — Source data Fig. 7 [file 44318_2026_705_MOESM17_ESM.zip › Figure 7/D/PC_noprot/20230208_LipoPC_noProt_1_w1SPI 491 GFP.TIF]

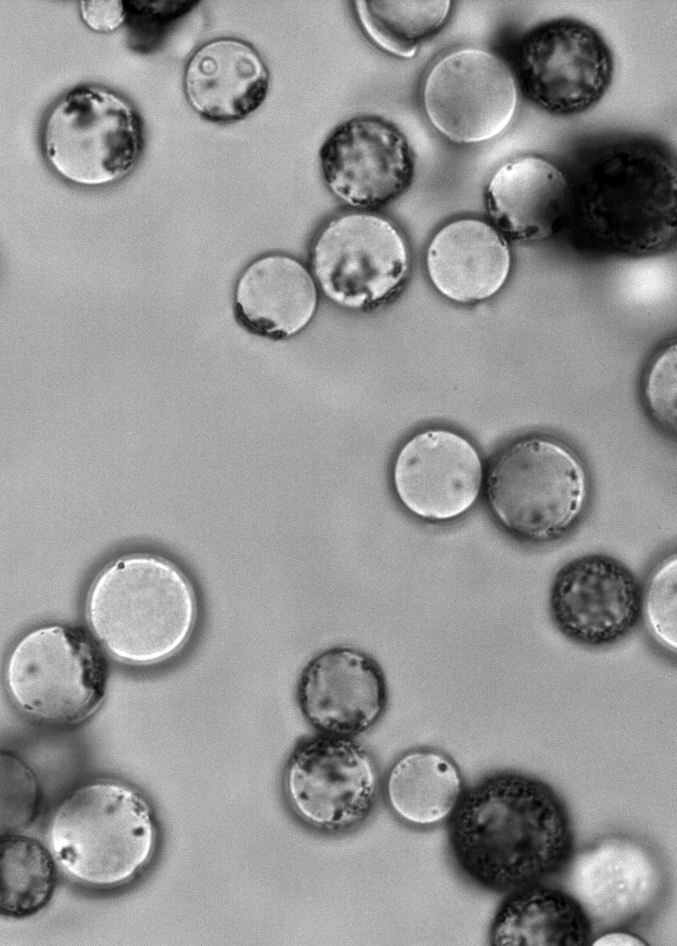

Supplement: Supplementary file 17 — Source data Fig. 7 [file 44318_2026_705_MOESM17_ESM.zip › Figure 7/D/PC_noprot/20230208_LipoPC_noProt_1_w2TRANS.TIF]

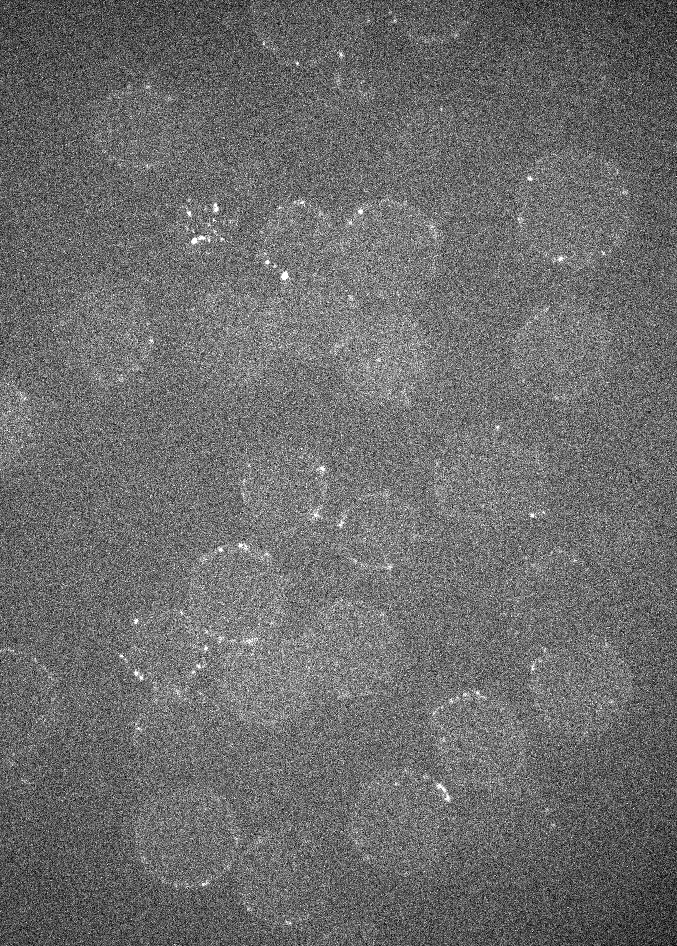

Supplement: Supplementary file 17 — Source data Fig. 7 [file 44318_2026_705_MOESM17_ESM.zip › Figure 7/D/PC_STARTWT/20230208_LipoPC_STARD3WT_1_w1SPI 491 GFP.TIF]

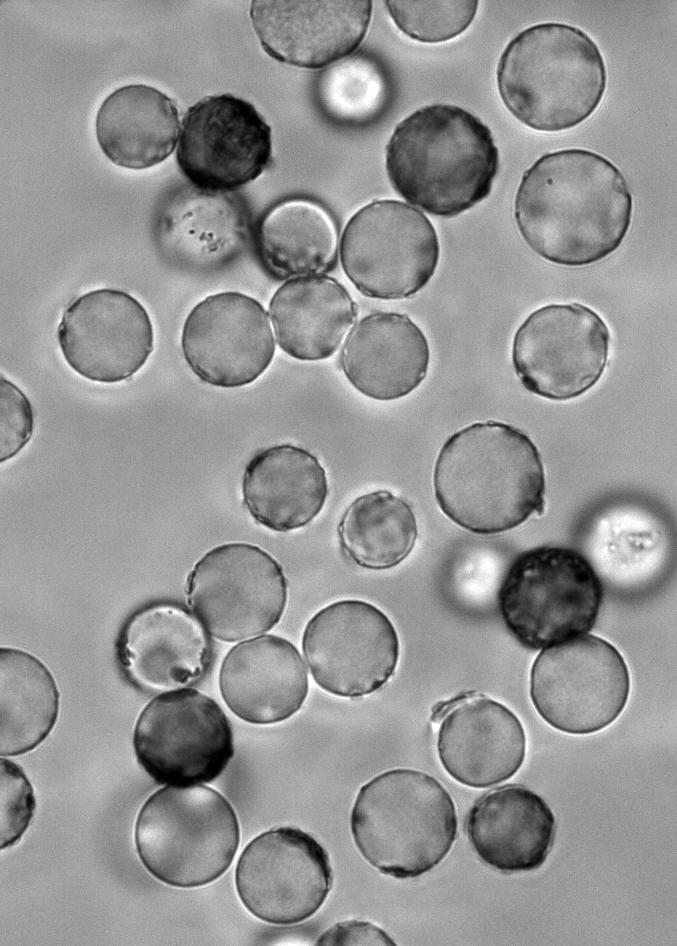

Supplement: Supplementary file 17 — Source data Fig. 7 [file 44318_2026_705_MOESM17_ESM.zip › Figure 7/D/PC_STARTWT/20230208_LipoPC_STARD3WT_1_w2TRANS.TIF]

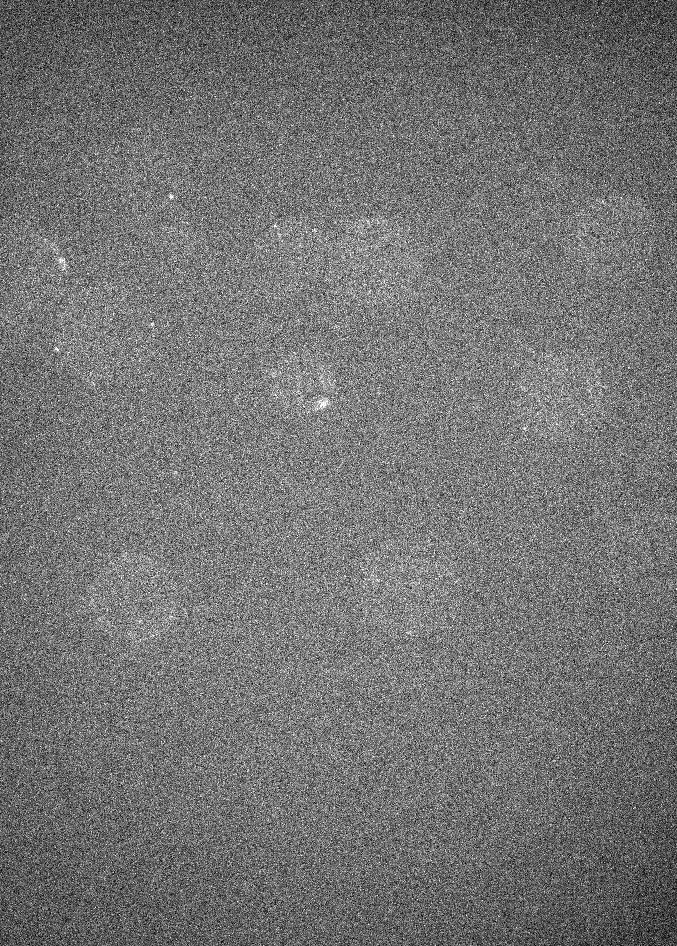

Supplement: Supplementary file 17 — Source data Fig. 7 [file 44318_2026_705_MOESM17_ESM.zip › Figure 7/E/PCPS_MSP/20230208_LipoPCPS_MSP_3_w1SPI 491 GFP.TIF]

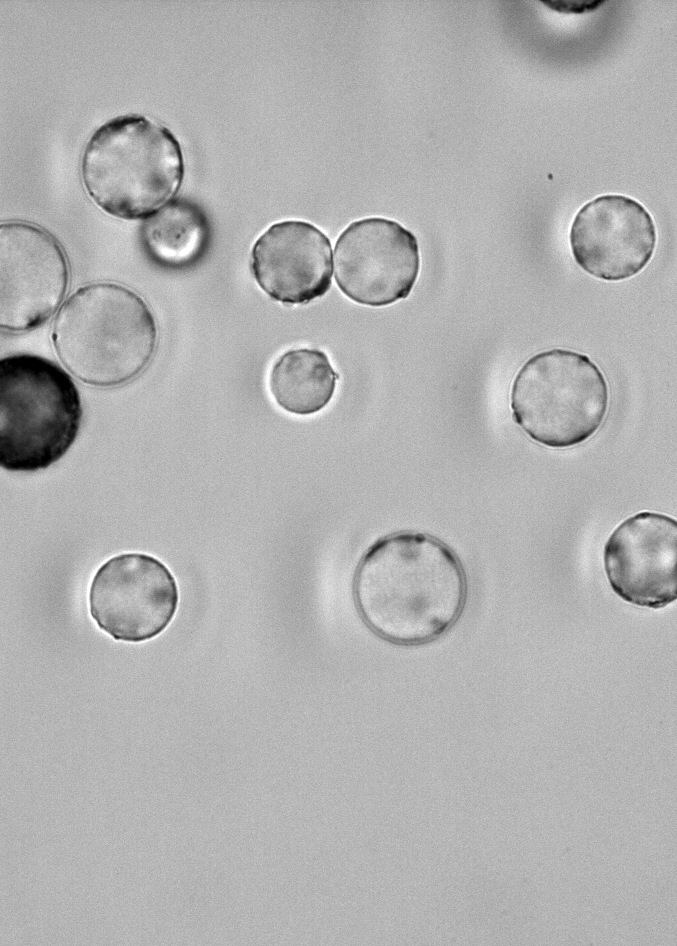

Supplement: Supplementary file 17 — Source data Fig. 7 [file 44318_2026_705_MOESM17_ESM.zip › Figure 7/E/PCPS_MSP/20230208_LipoPCPS_MSP_3_w2TRANS.TIF]

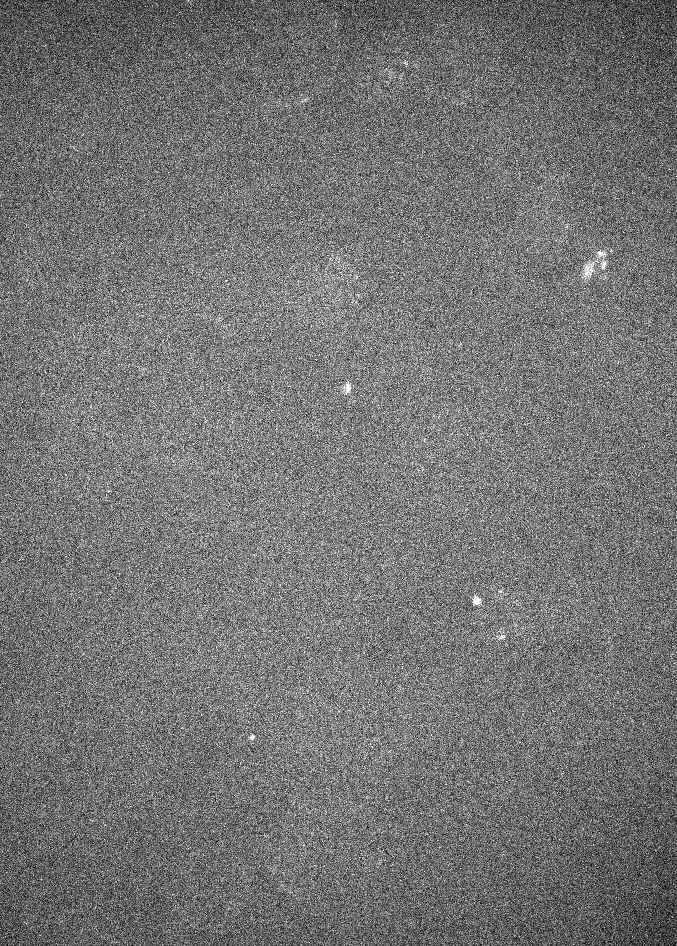

Supplement: Supplementary file 17 — Source data Fig. 7 [file 44318_2026_705_MOESM17_ESM.zip › Figure 7/E/PCPS_noprot/20230208_LipoPCPS_NoProt_3_w1SPI 491 GFP.TIF]

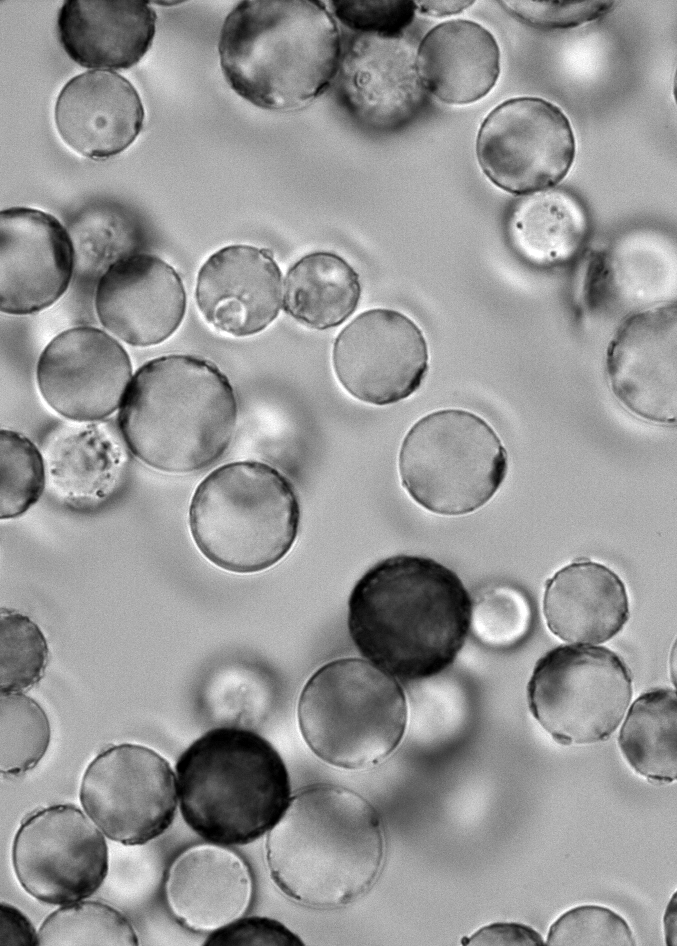

Supplement: Supplementary file 17 — Source data Fig. 7 [file 44318_2026_705_MOESM17_ESM.zip › Figure 7/E/PCPS_noprot/20230208_LipoPCPS_NoProt_3_w2TRANS.TIF]

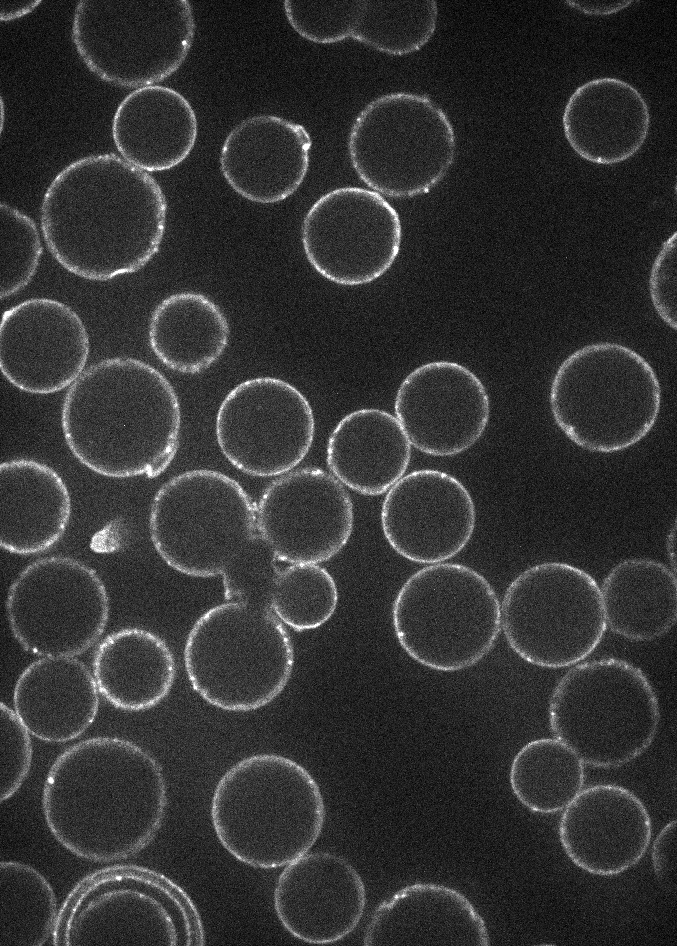

Supplement: Supplementary file 17 — Source data Fig. 7 [file 44318_2026_705_MOESM17_ESM.zip › Figure 7/E/PCPS_STARTWT/20230208_LipoPCPS_STARD3WT_1_w1SPI 491 GFP.TIF]

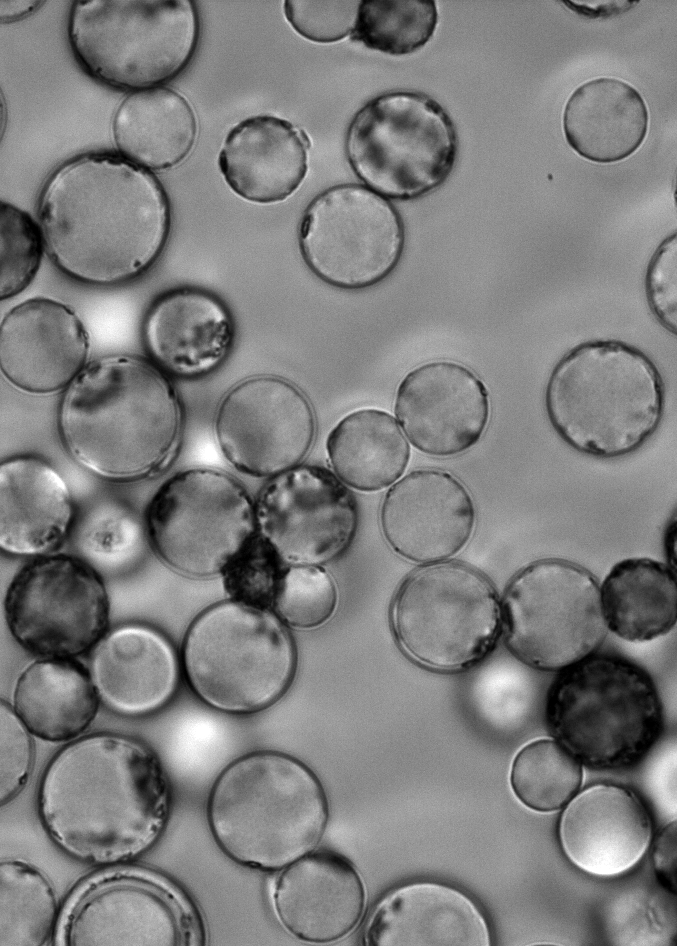

Supplement: Supplementary file 17 — Source data Fig. 7 [file 44318_2026_705_MOESM17_ESM.zip › Figure 7/E/PCPS_STARTWT/20230208_LipoPCPS_STARD3WT_1_w2TRANS.TIF]

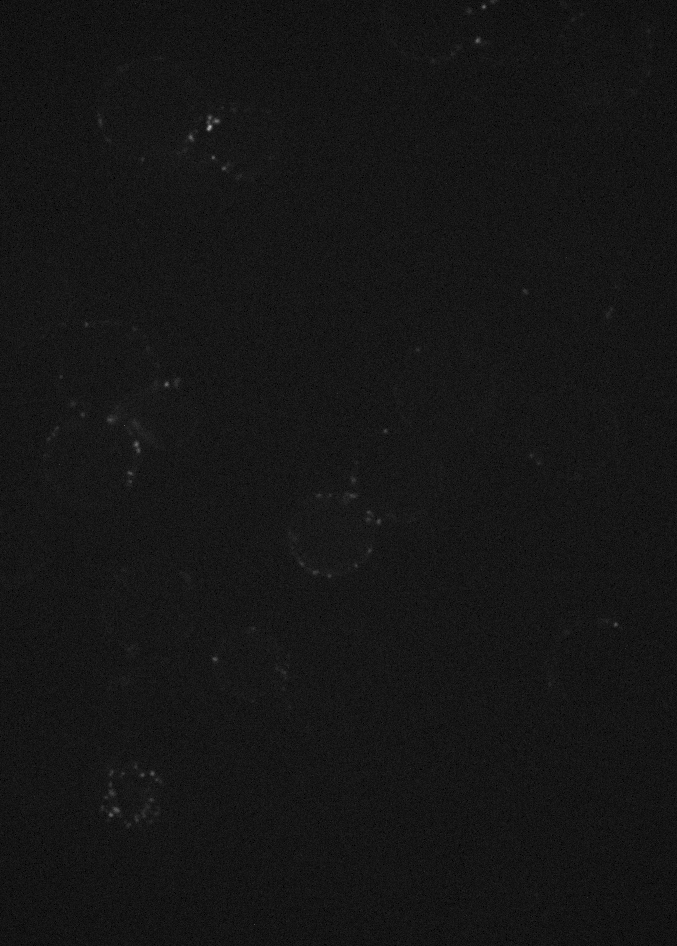

Supplement: Supplementary file 17 — Source data Fig. 7 [file 44318_2026_705_MOESM17_ESM.zip › Figure 7/G/PCPS_noProt/20250205_STARTnoProt_2_w1SPI 491 GFP.TIF]

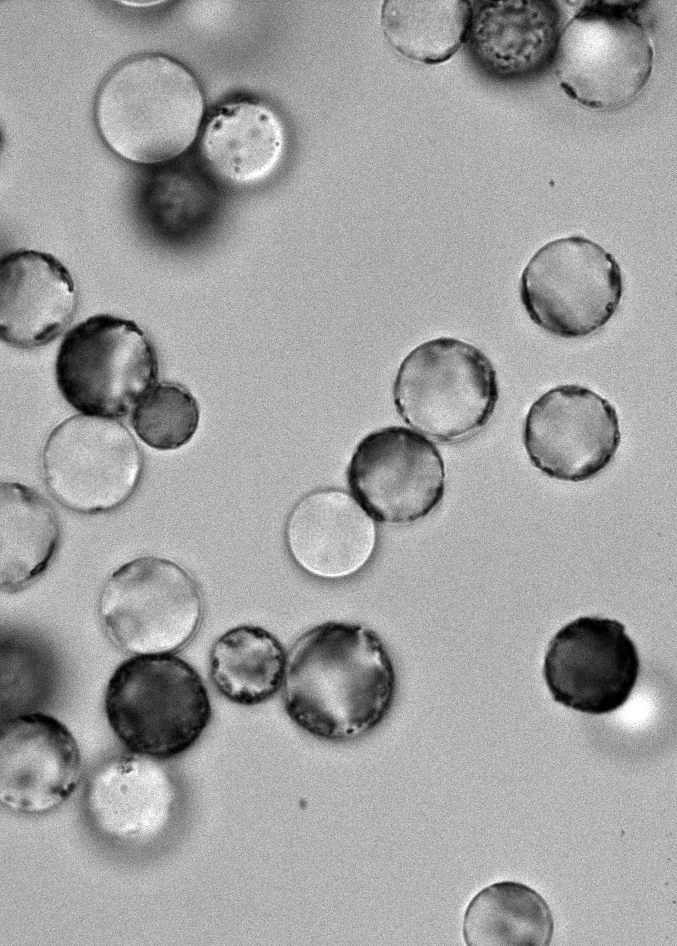

Supplement: Supplementary file 17 — Source data Fig. 7 [file 44318_2026_705_MOESM17_ESM.zip › Figure 7/G/PCPS_noProt/20250205_STARTnoProt_2_w2TRANS.TIF]

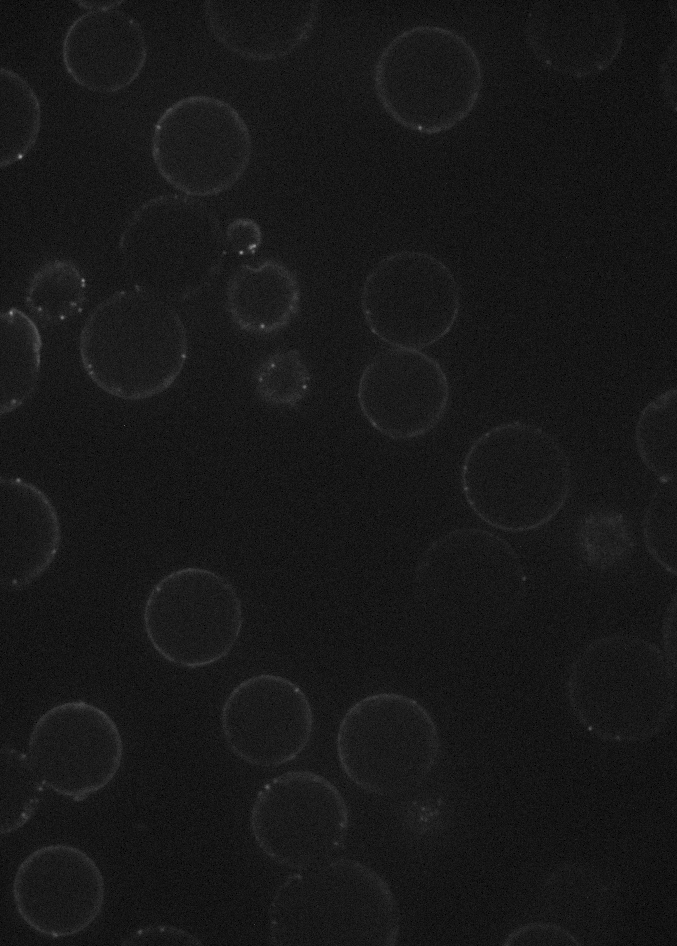

Supplement: Supplementary file 17 — Source data Fig. 7 [file 44318_2026_705_MOESM17_ESM.zip › Figure 7/G/PCPS_STARTKDKD/20250205_STARTK2D2t_5_w1SPI 491 GFP.TIF]

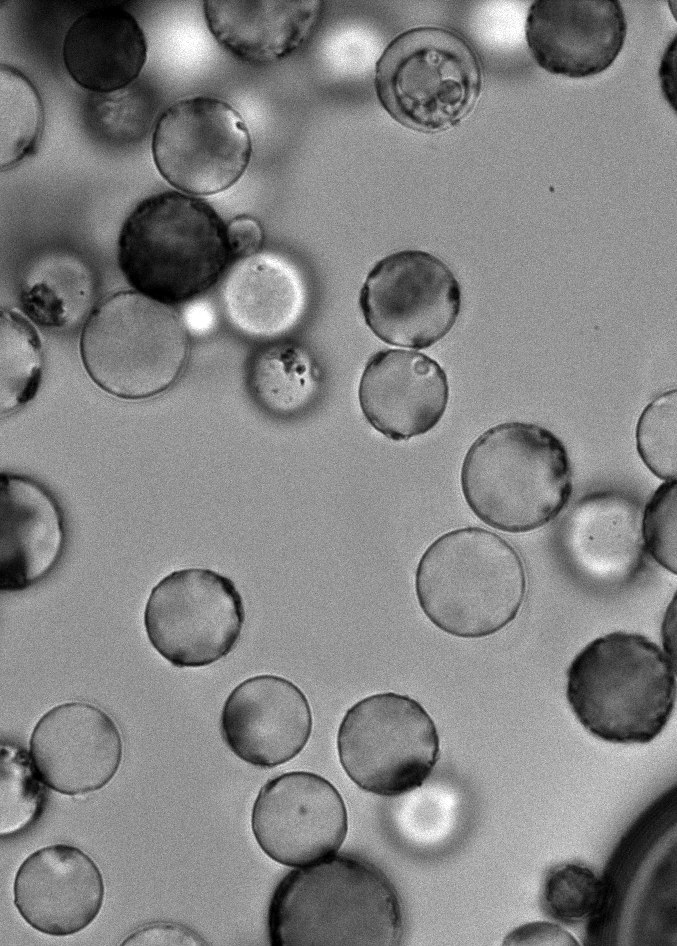

Supplement: Supplementary file 17 — Source data Fig. 7 [file 44318_2026_705_MOESM17_ESM.zip › Figure 7/G/PCPS_STARTKDKD/20250205_STARTK2D2t_5_w2TRANS.TIF]

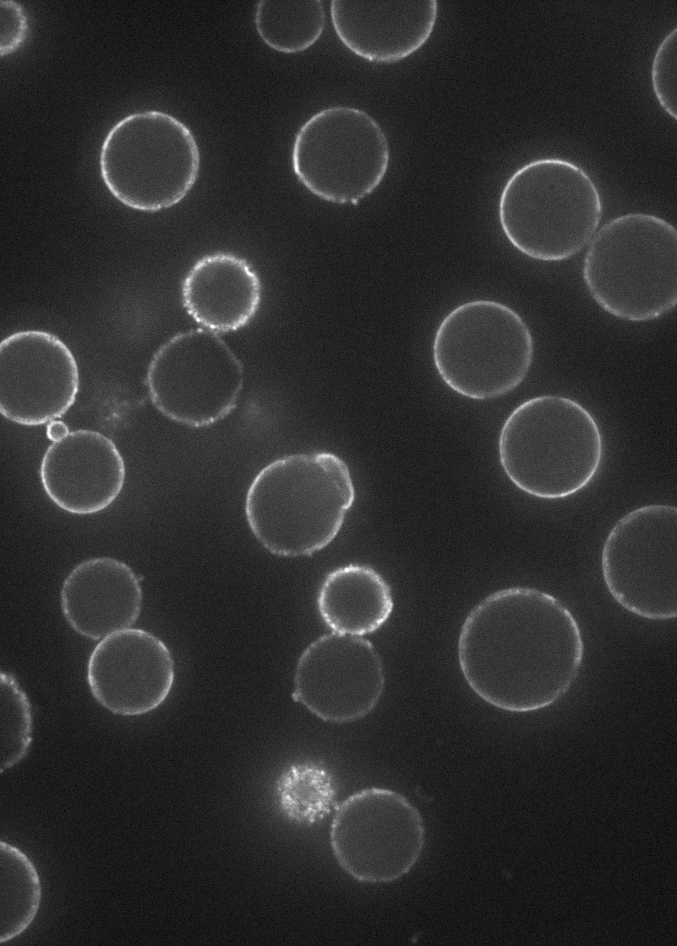

Supplement: Supplementary file 17 — Source data Fig. 7 [file 44318_2026_705_MOESM17_ESM.zip › Figure 7/G/PCPS_STARTWT/20250205_STARTWT_6_w1SPI 491 GFP.TIF]

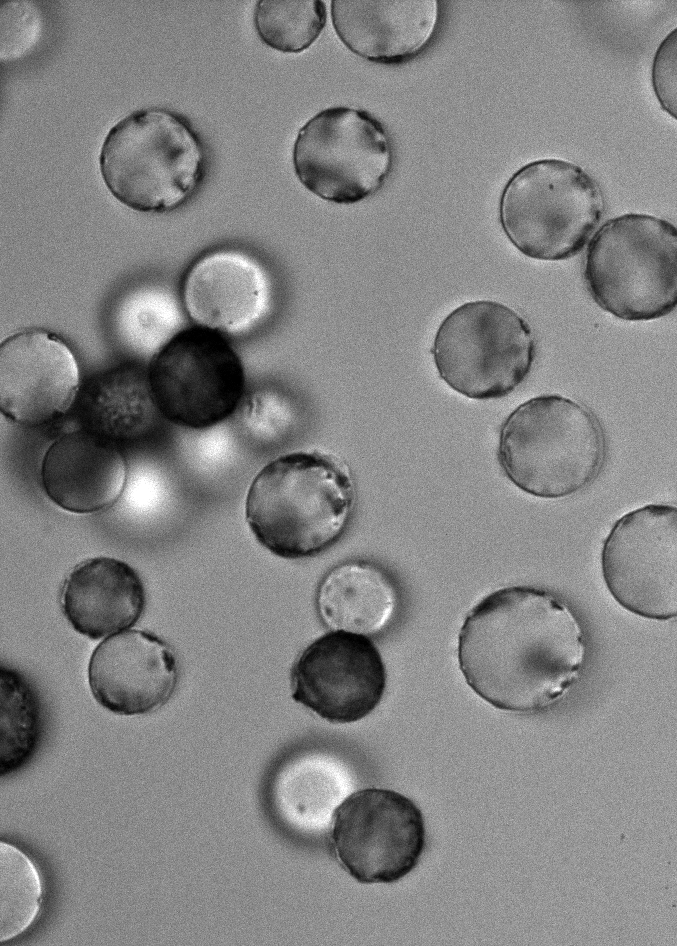

Supplement: Supplementary file 17 — Source data Fig. 7 [file 44318_2026_705_MOESM17_ESM.zip › Figure 7/G/PCPS_STARTWT/20250205_STARTWT_6_w2TRANS.TIF]

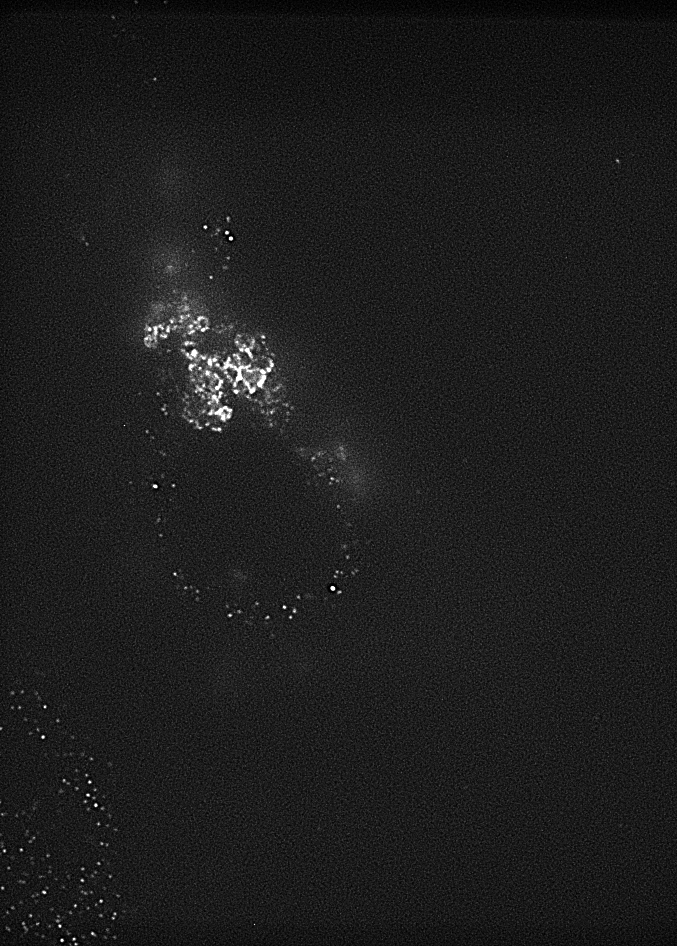

Supplement: Supplementary file 18 — Source data Fig. 8 [file 44318_2026_705_MOESM18_ESM.zip › Figure 8/B/STARD3WT_CHIR99021/20250113_MCF7STARD3WT_CHIR_2_SR_w1SPI 491 GFP.TIF]

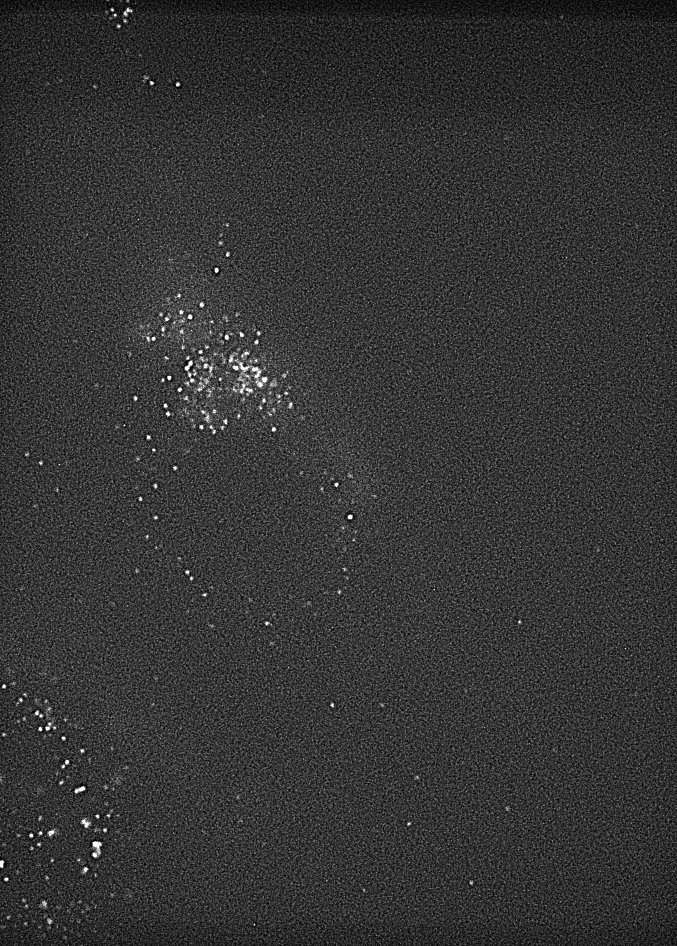

Supplement: Supplementary file 18 — Source data Fig. 8 [file 44318_2026_705_MOESM18_ESM.zip › Figure 8/B/STARD3WT_CHIR99021/20250113_MCF7STARD3WT_CHIR_2_SR_w2SPI 561 mCherry.TIF]

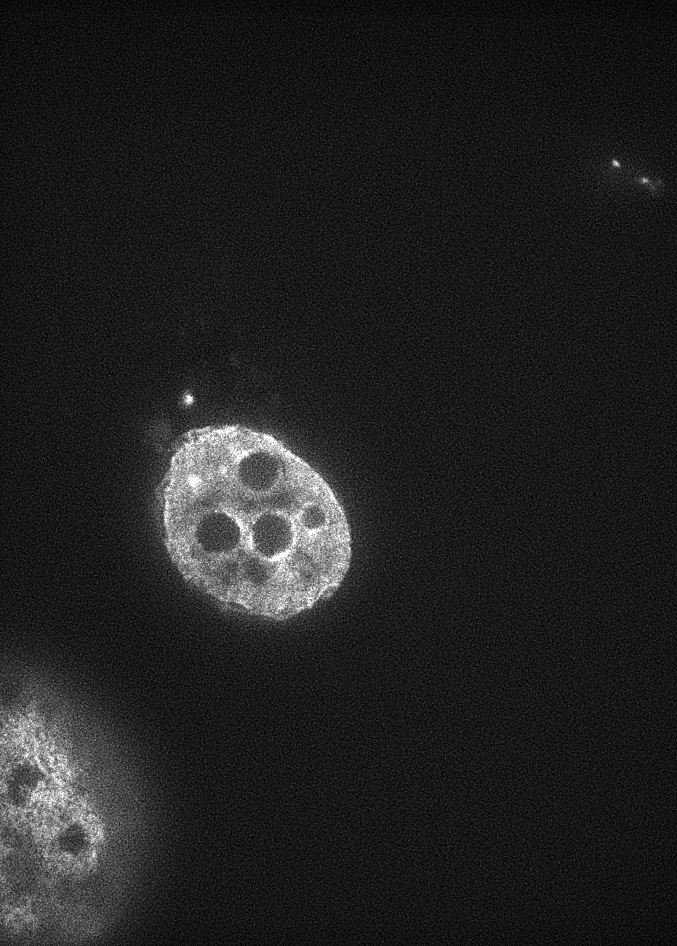

Supplement: Supplementary file 18 — Source data Fig. 8 [file 44318_2026_705_MOESM18_ESM.zip › Figure 8/B/STARD3WT_CHIR99021/20250113_MCF7STARD3WT_CHIR_2_SR_w3SPI 405 DAPI.TIF]

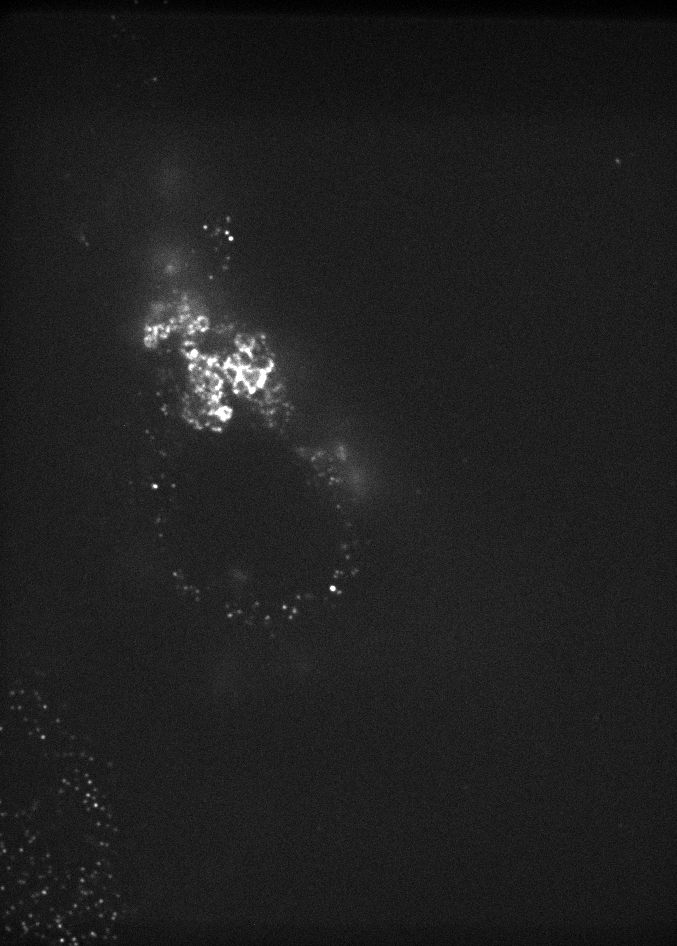

Supplement: Supplementary file 18 — Source data Fig. 8 [file 44318_2026_705_MOESM18_ESM.zip › Figure 8/B/STARD3WT_CHIR99021/20250113_MCF7STARD3WT_CHIR_2_w1SPI 491 GFP.TIF]

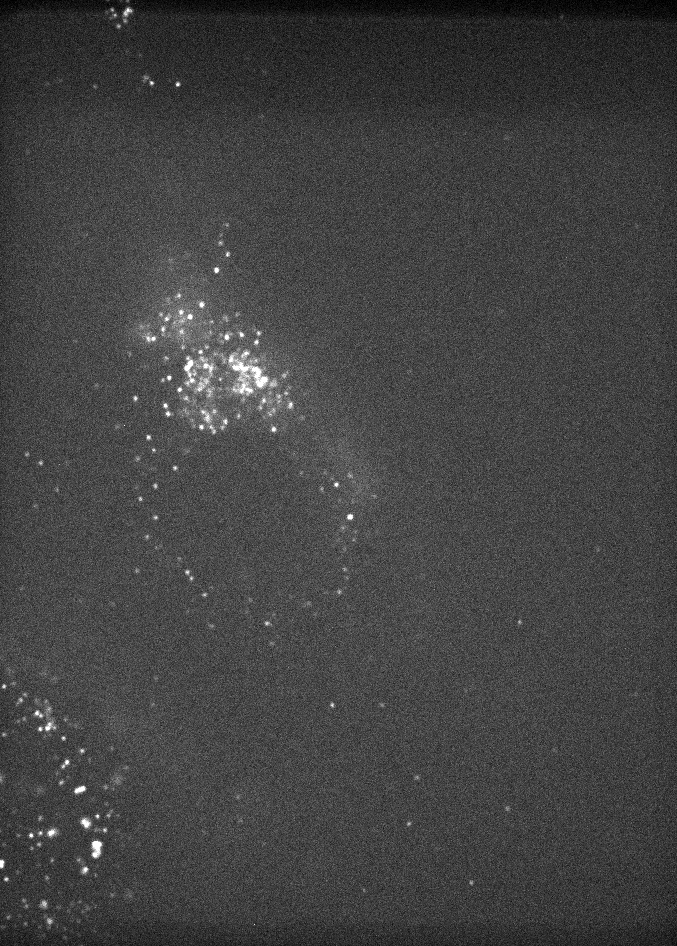

Supplement: Supplementary file 18 — Source data Fig. 8 [file 44318_2026_705_MOESM18_ESM.zip › Figure 8/B/STARD3WT_CHIR99021/20250113_MCF7STARD3WT_CHIR_2_w2SPI 561 mCherry.TIF]

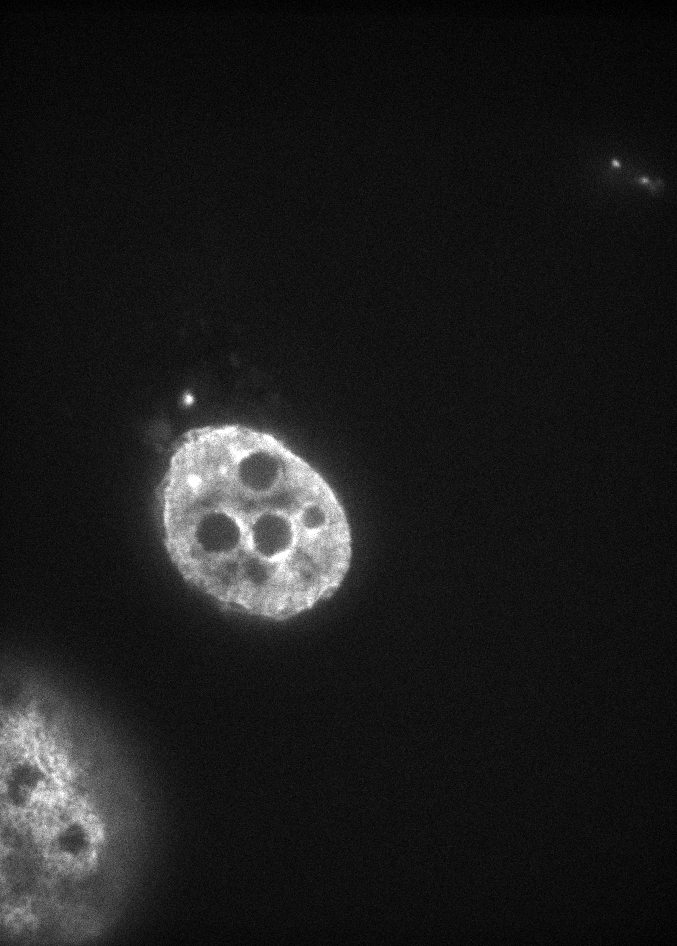

Supplement: Supplementary file 18 — Source data Fig. 8 [file 44318_2026_705_MOESM18_ESM.zip › Figure 8/B/STARD3WT_CHIR99021/20250113_MCF7STARD3WT_CHIR_2_w3SPI 405 DAPI.TIF]

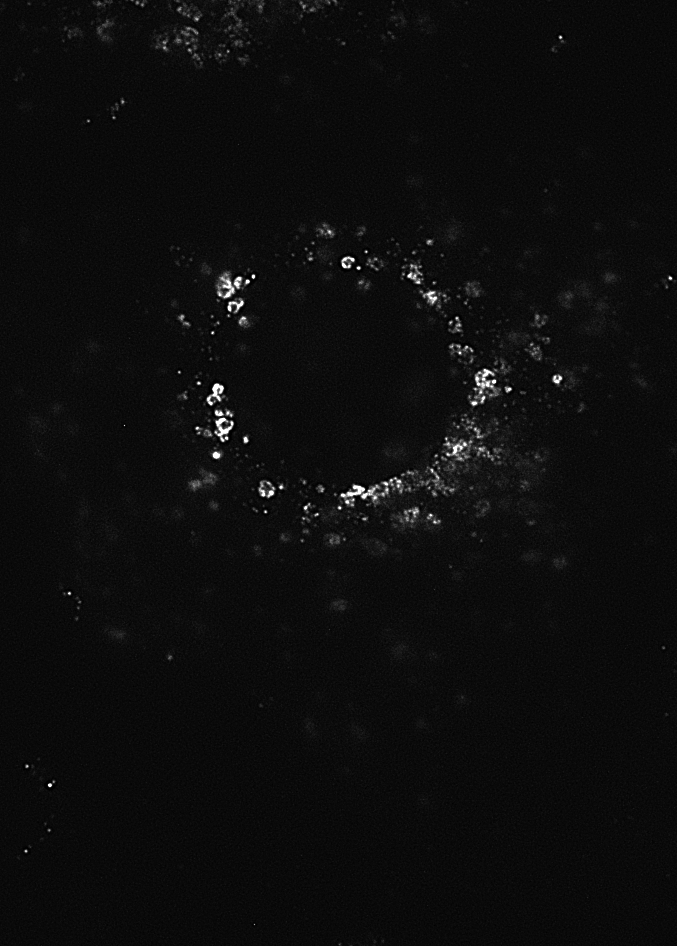

Supplement: Supplementary file 18 — Source data Fig. 8 [file 44318_2026_705_MOESM18_ESM.zip › Figure 8/B/STARD3WT_NT/20250113_MCF7STARD3WT_NT_1_SR_w1SPI 491 GFP.TIF]

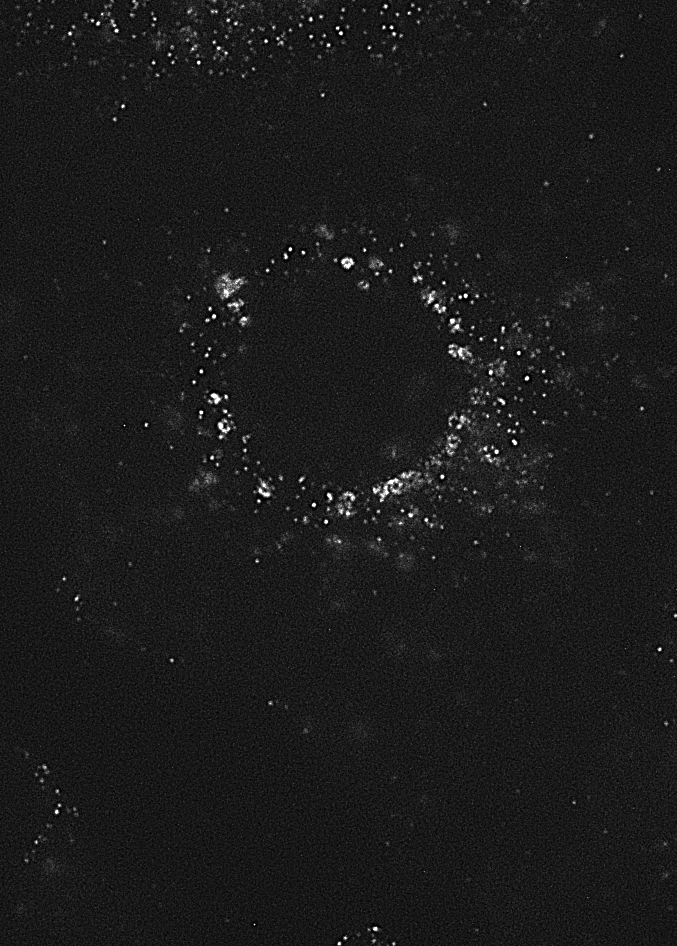

Supplement: Supplementary file 18 — Source data Fig. 8 [file 44318_2026_705_MOESM18_ESM.zip › Figure 8/B/STARD3WT_NT/20250113_MCF7STARD3WT_NT_1_SR_w2SPI 561 mCherry.TIF]

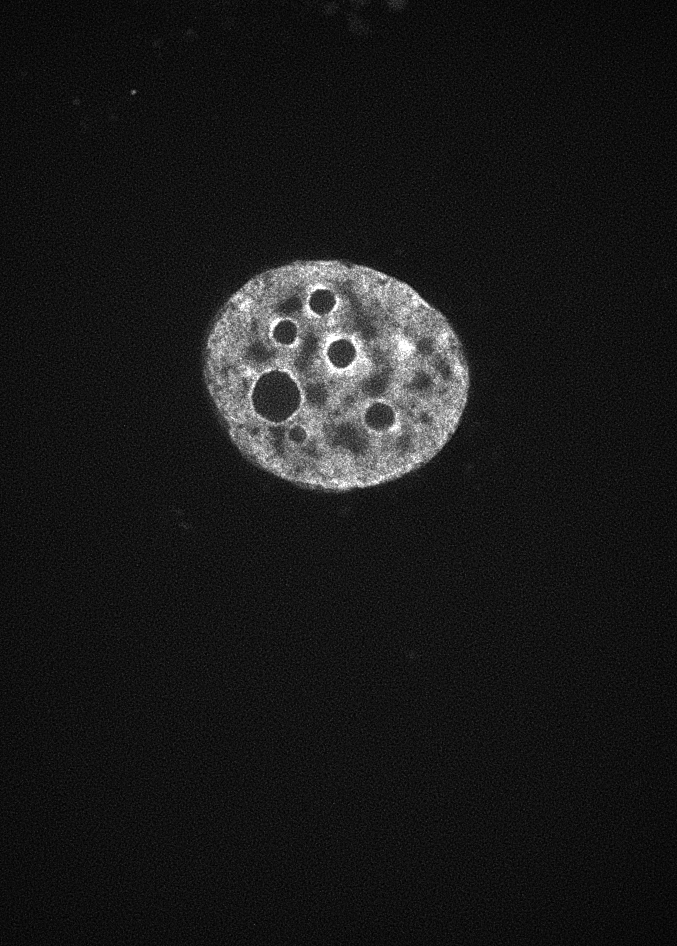

Supplement: Supplementary file 18 — Source data Fig. 8 [file 44318_2026_705_MOESM18_ESM.zip › Figure 8/B/STARD3WT_NT/20250113_MCF7STARD3WT_NT_1_SR_w3SPI 405 DAPI.TIF]

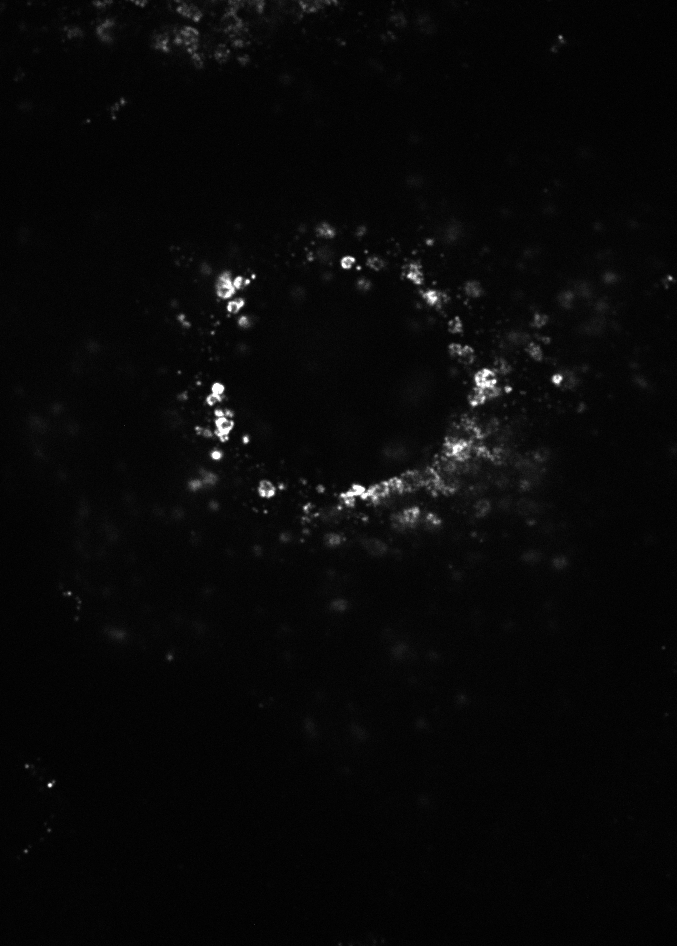

Supplement: Supplementary file 18 — Source data Fig. 8 [file 44318_2026_705_MOESM18_ESM.zip › Figure 8/B/STARD3WT_NT/20250113_MCF7STARD3WT_NT_1_w1SPI 491 GFP.TIF]

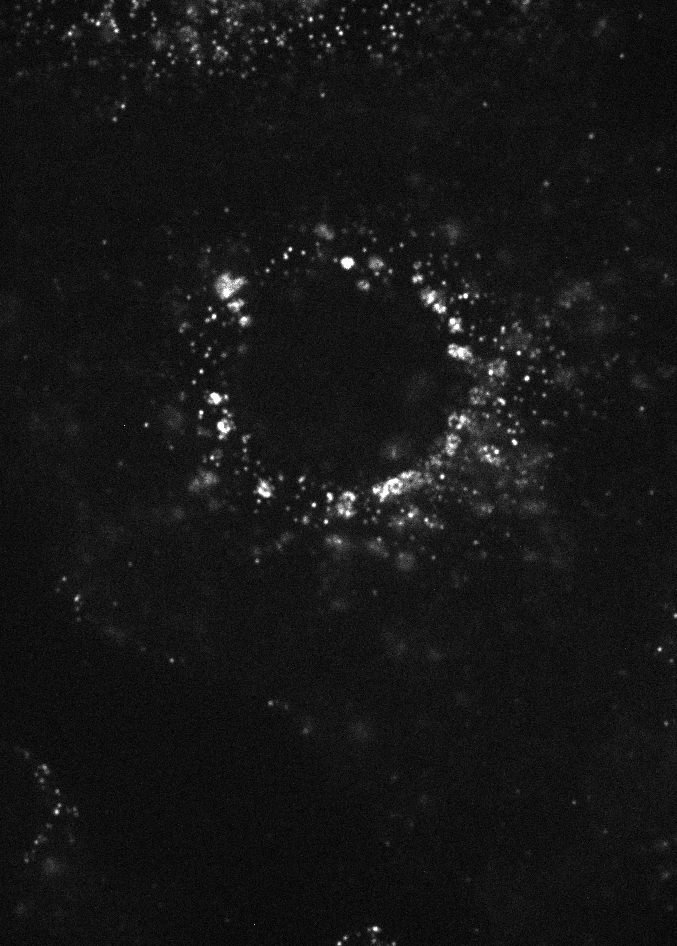

Supplement: Supplementary file 18 — Source data Fig. 8 [file 44318_2026_705_MOESM18_ESM.zip › Figure 8/B/STARD3WT_NT/20250113_MCF7STARD3WT_NT_1_w2SPI 561 mCherry.TIF]

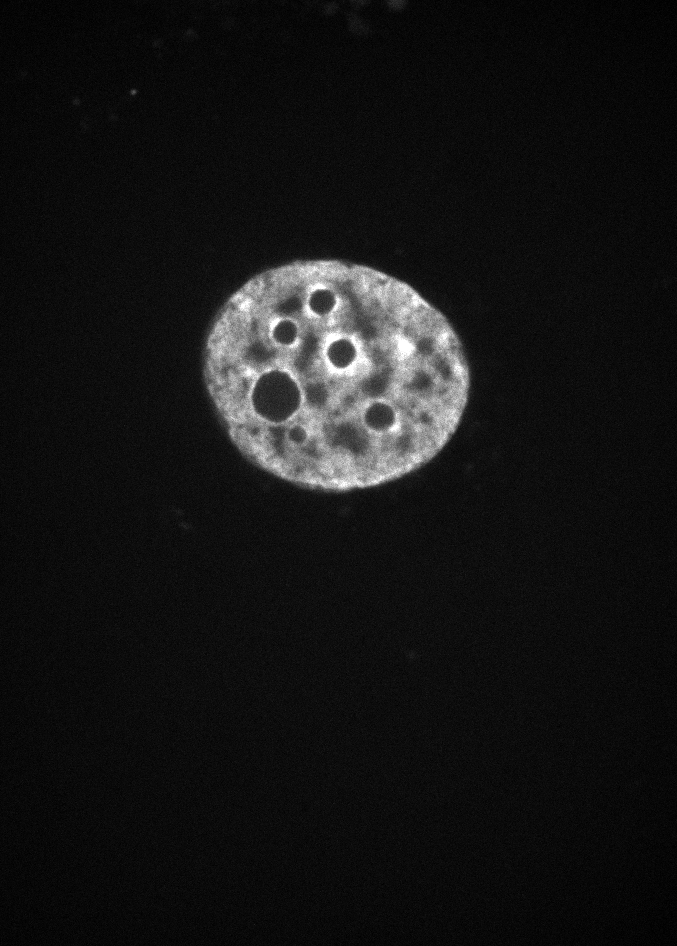

Supplement: Supplementary file 18 — Source data Fig. 8 [file 44318_2026_705_MOESM18_ESM.zip › Figure 8/B/STARD3WT_NT/20250113_MCF7STARD3WT_NT_1_w3SPI 405 DAPI.TIF]

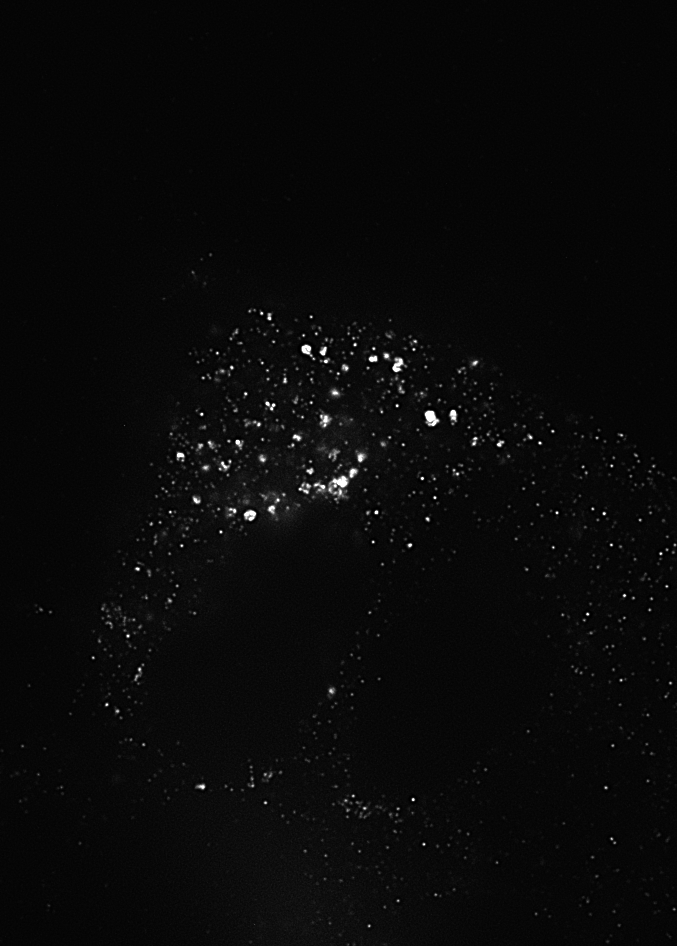

Supplement: Supplementary file 18 — Source data Fig. 8 [file 44318_2026_705_MOESM18_ESM.zip › Figure 8/C/STARD3_KD_KD_CHIR99021/20250129_MCF7STARD3K2D2_CHIR_7_SR_w1SPI 491 GFP.TIF]

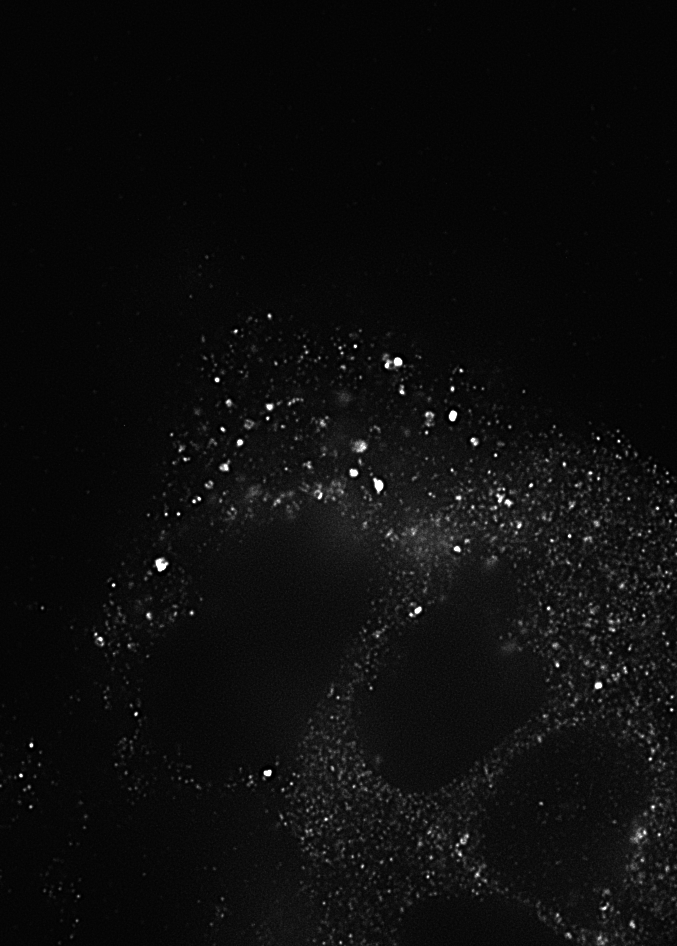

Supplement: Supplementary file 18 — Source data Fig. 8 [file 44318_2026_705_MOESM18_ESM.zip › Figure 8/C/STARD3_KD_KD_CHIR99021/20250129_MCF7STARD3K2D2_CHIR_7_SR_w2SPI 561 mCherry.TIF]

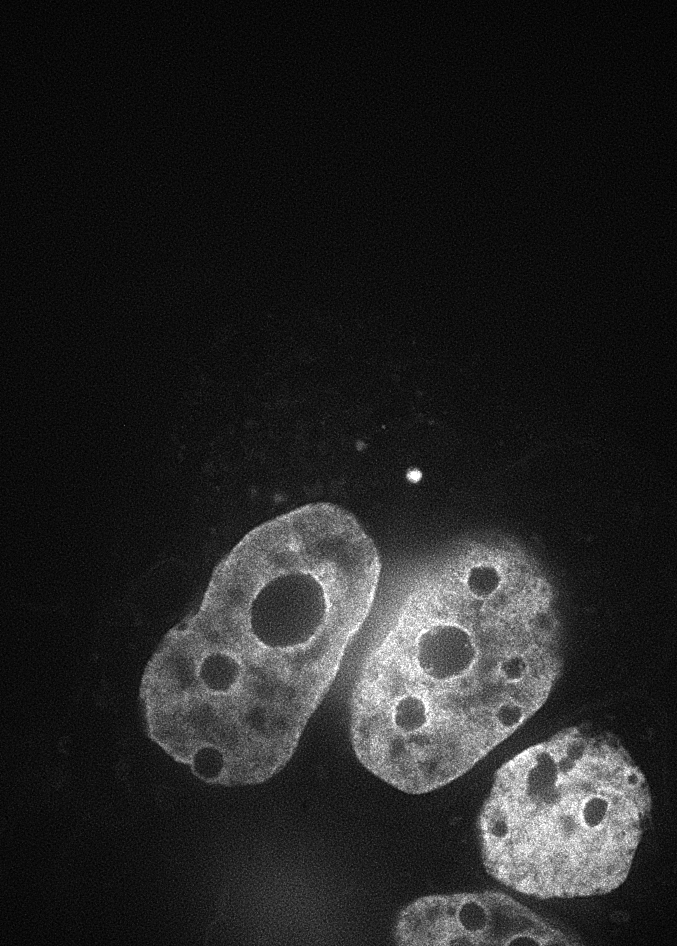

Supplement: Supplementary file 18 — Source data Fig. 8 [file 44318_2026_705_MOESM18_ESM.zip › Figure 8/C/STARD3_KD_KD_CHIR99021/20250129_MCF7STARD3K2D2_CHIR_7_SR_w3SPI 405 DAPI.TIF]

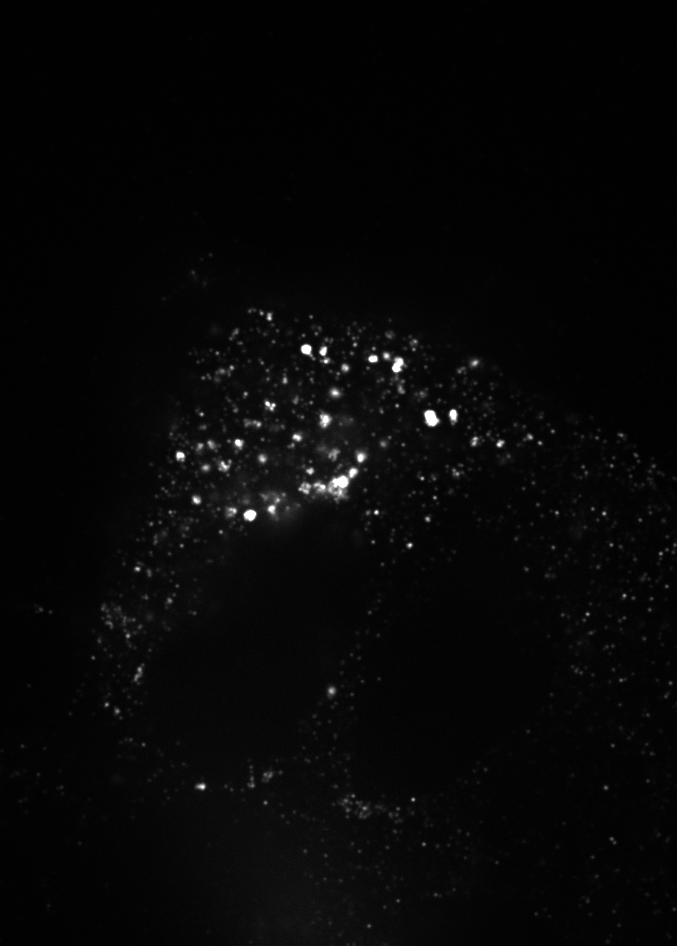

Supplement: Supplementary file 18 — Source data Fig. 8 [file 44318_2026_705_MOESM18_ESM.zip › Figure 8/C/STARD3_KD_KD_CHIR99021/20250129_MCF7STARD3K2D2_CHIR_7_w1SPI 491 GFP.TIF]

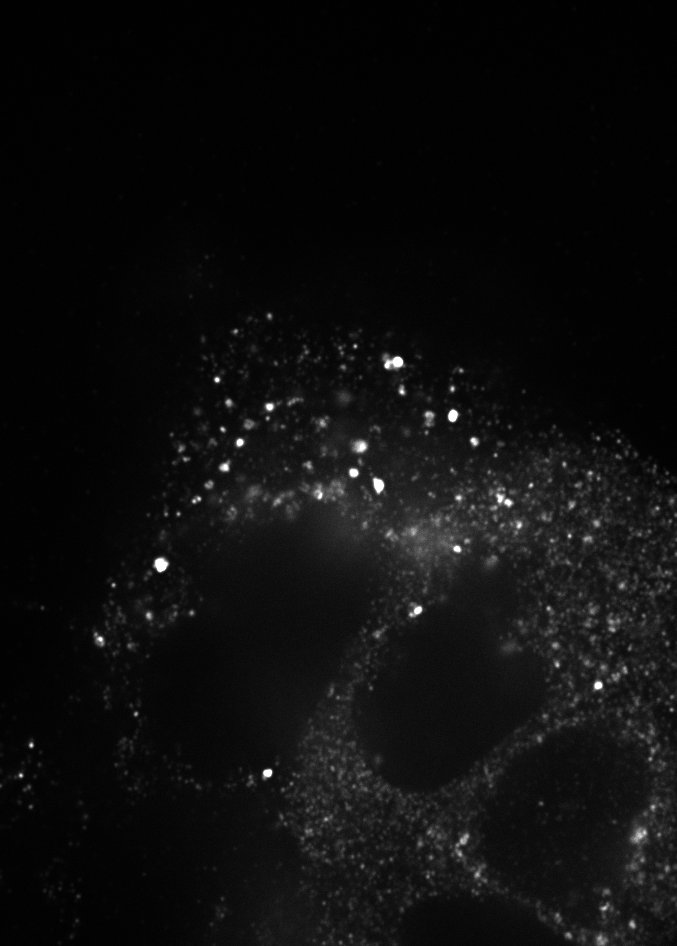

Supplement: Supplementary file 18 — Source data Fig. 8 [file 44318_2026_705_MOESM18_ESM.zip › Figure 8/C/STARD3_KD_KD_CHIR99021/20250129_MCF7STARD3K2D2_CHIR_7_w2SPI 561 mCherry.TIF]

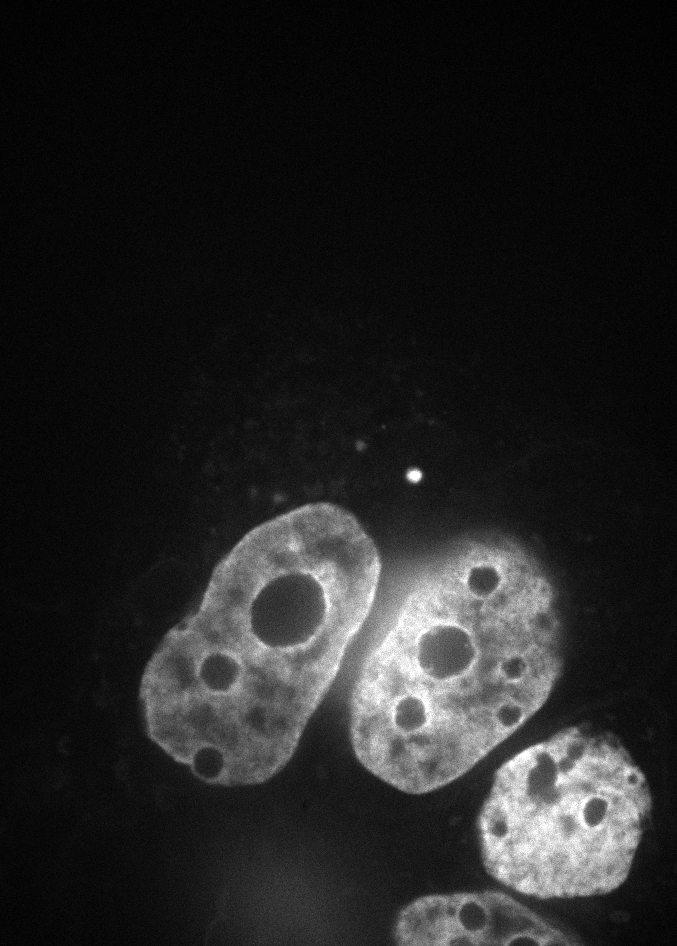

Supplement: Supplementary file 18 — Source data Fig. 8 [file 44318_2026_705_MOESM18_ESM.zip › Figure 8/C/STARD3_KD_KD_CHIR99021/20250129_MCF7STARD3K2D2_CHIR_7_w3SPI 405 DAPI.TIF]

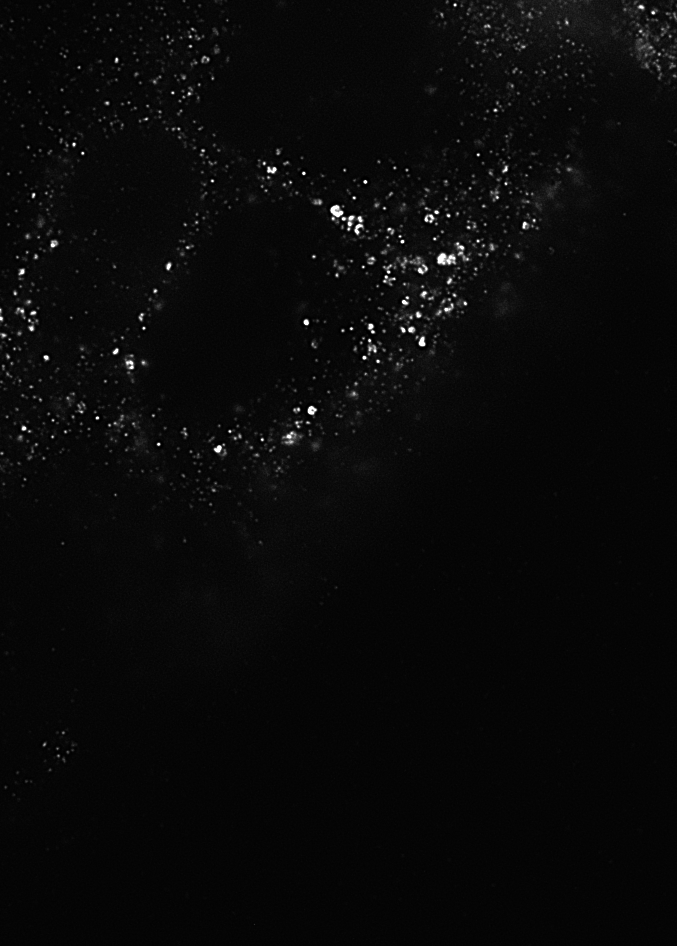

Supplement: Supplementary file 18 — Source data Fig. 8 [file 44318_2026_705_MOESM18_ESM.zip › Figure 8/C/STARD3_KD_KD_NT/20250129_MCF7STARD3K2D2_NT_3_SR_w1SPI 491 GFP.TIF]

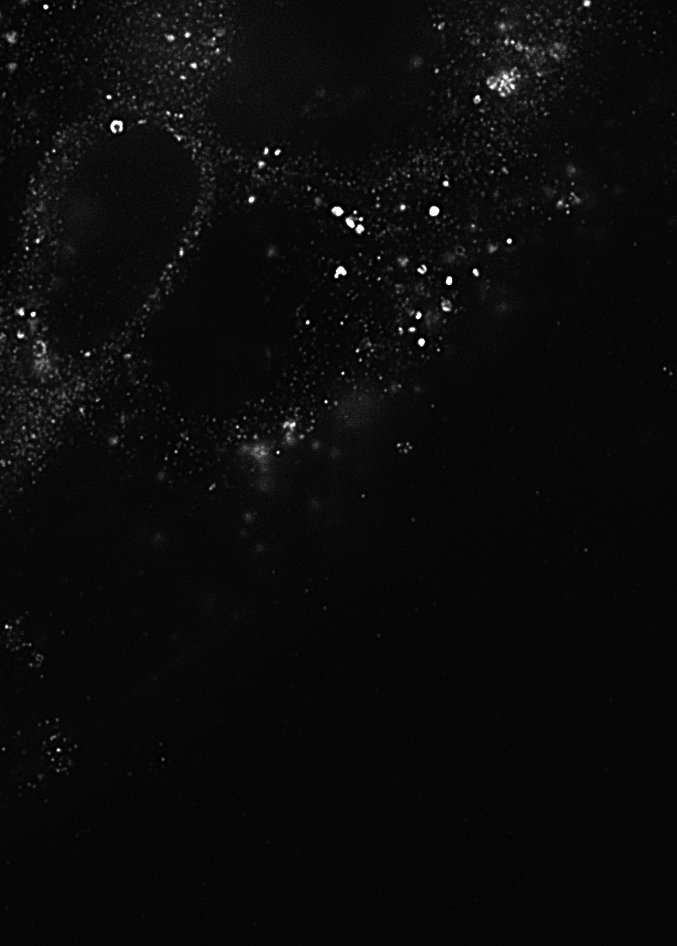

Supplement: Supplementary file 18 — Source data Fig. 8 [file 44318_2026_705_MOESM18_ESM.zip › Figure 8/C/STARD3_KD_KD_NT/20250129_MCF7STARD3K2D2_NT_3_SR_w2SPI 561 mCherry.TIF]

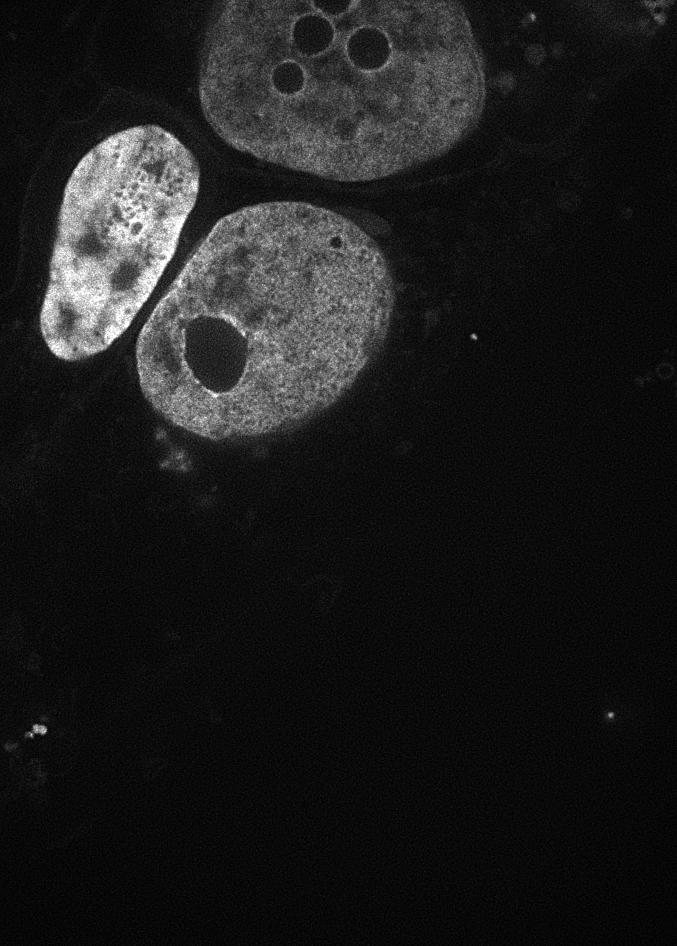

Supplement: Supplementary file 18 — Source data Fig. 8 [file 44318_2026_705_MOESM18_ESM.zip › Figure 8/C/STARD3_KD_KD_NT/20250129_MCF7STARD3K2D2_NT_3_SR_w3SPI 405 DAPI.TIF]

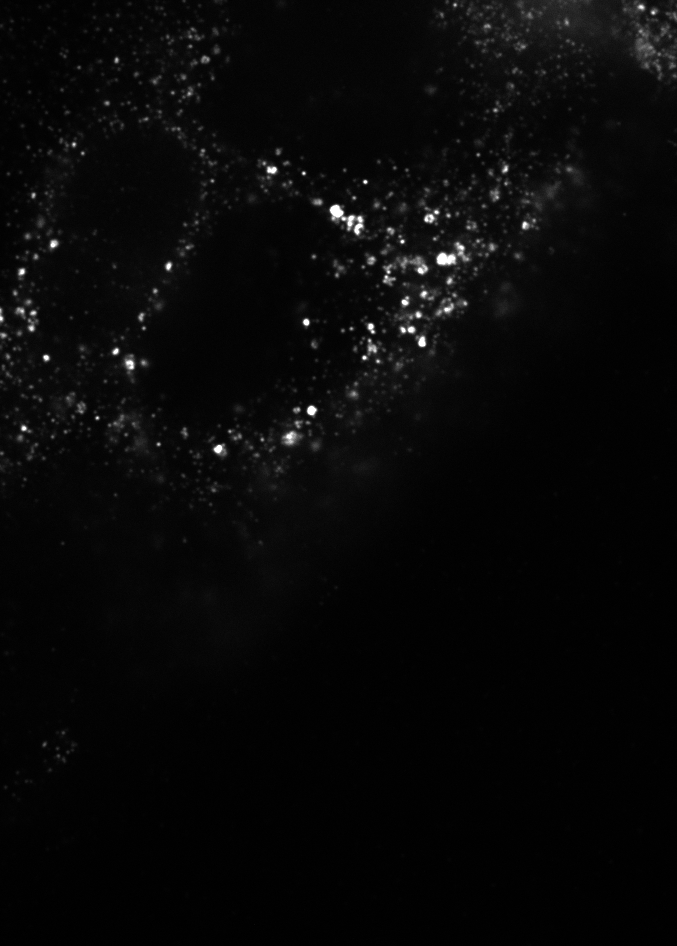

Supplement: Supplementary file 18 — Source data Fig. 8 [file 44318_2026_705_MOESM18_ESM.zip › Figure 8/C/STARD3_KD_KD_NT/20250129_MCF7STARD3K2D2_NT_3_w1SPI 491 GFP.TIF]

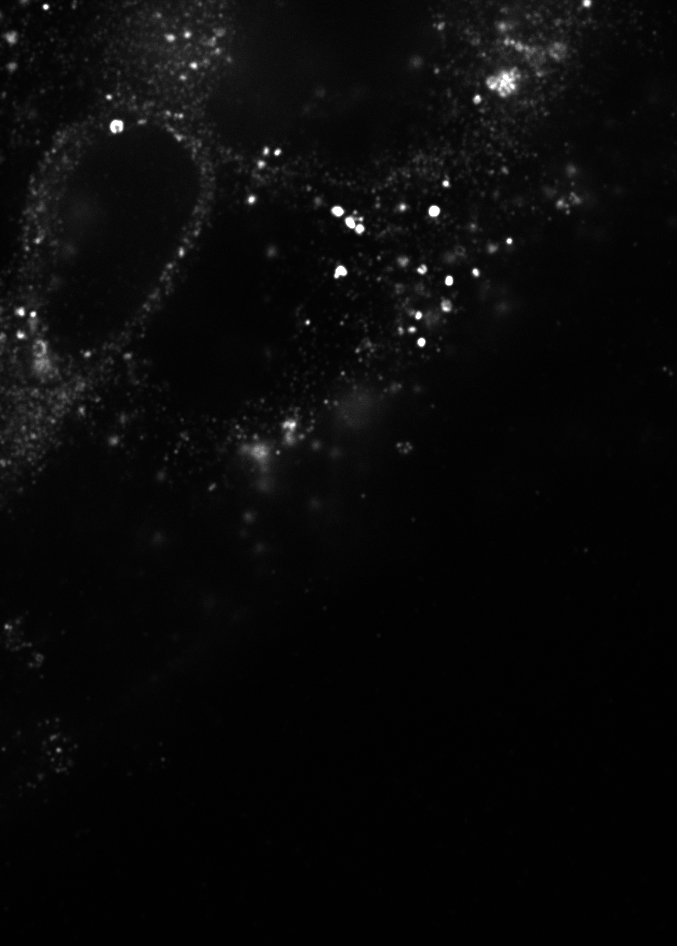

Supplement: Supplementary file 18 — Source data Fig. 8 [file 44318_2026_705_MOESM18_ESM.zip › Figure 8/C/STARD3_KD_KD_NT/20250129_MCF7STARD3K2D2_NT_3_w2SPI 561 mCherry.TIF]

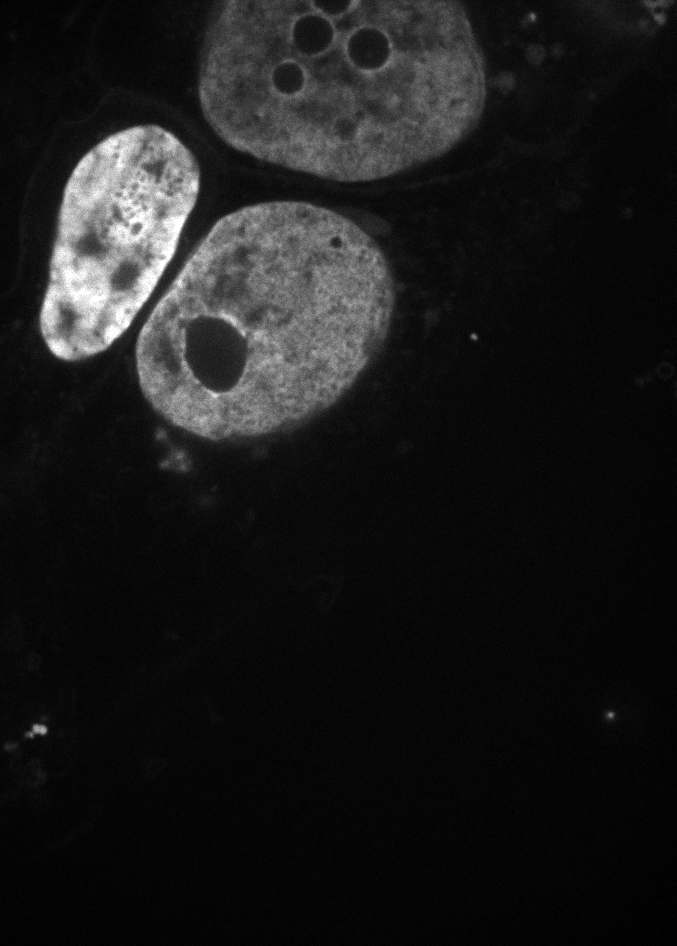

Supplement: Supplementary file 18 — Source data Fig. 8 [file 44318_2026_705_MOESM18_ESM.zip › Figure 8/C/STARD3_KD_KD_NT/20250129_MCF7STARD3K2D2_NT_3_w3SPI 405 DAPI.TIF]

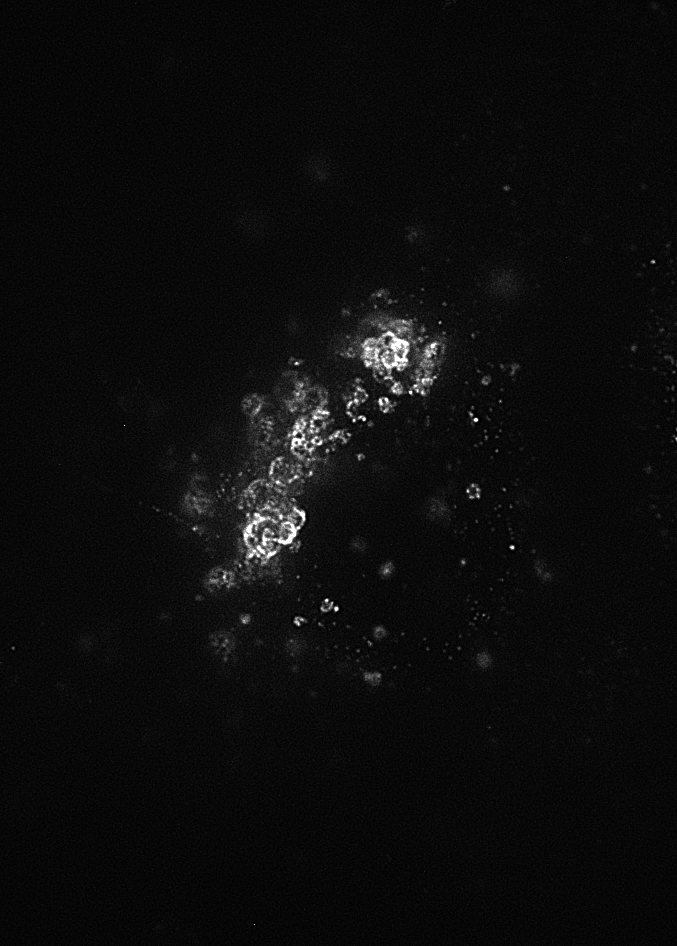

Supplement: Supplementary file 18 — Source data Fig. 8 [file 44318_2026_705_MOESM18_ESM.zip › Figure 8/D/20250113_MCF7STARD3S209A_NT_5_SR_w1SPI 491 GFP.TIF]

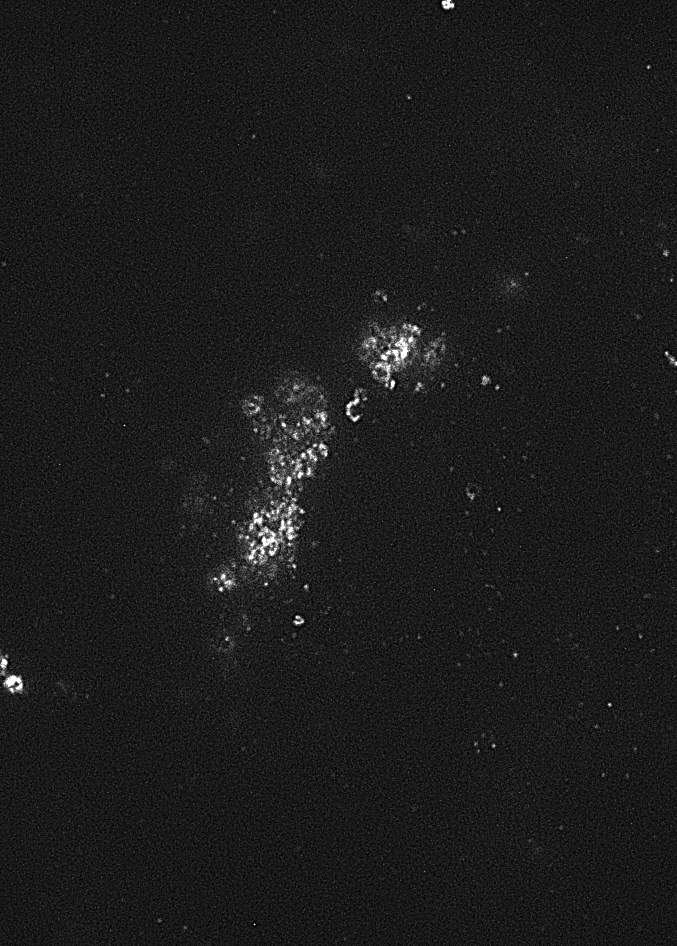

Supplement: Supplementary file 18 — Source data Fig. 8 [file 44318_2026_705_MOESM18_ESM.zip › Figure 8/D/20250113_MCF7STARD3S209A_NT_5_SR_w2SPI 561 mCherry.TIF]

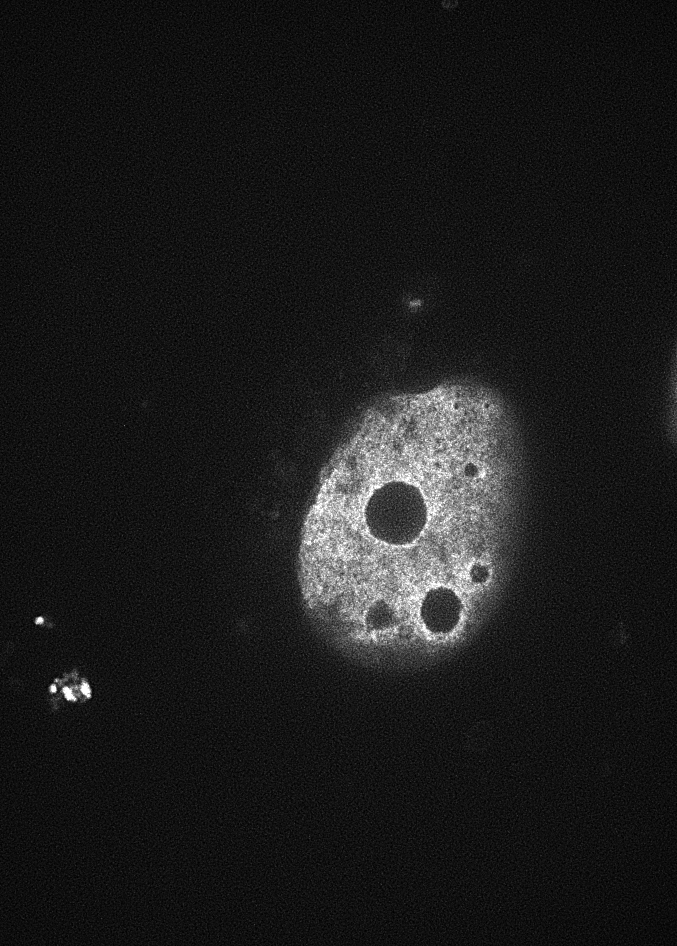

Supplement: Supplementary file 18 — Source data Fig. 8 [file 44318_2026_705_MOESM18_ESM.zip › Figure 8/D/20250113_MCF7STARD3S209A_NT_5_SR_w3SPI 405 DAPI.TIF]

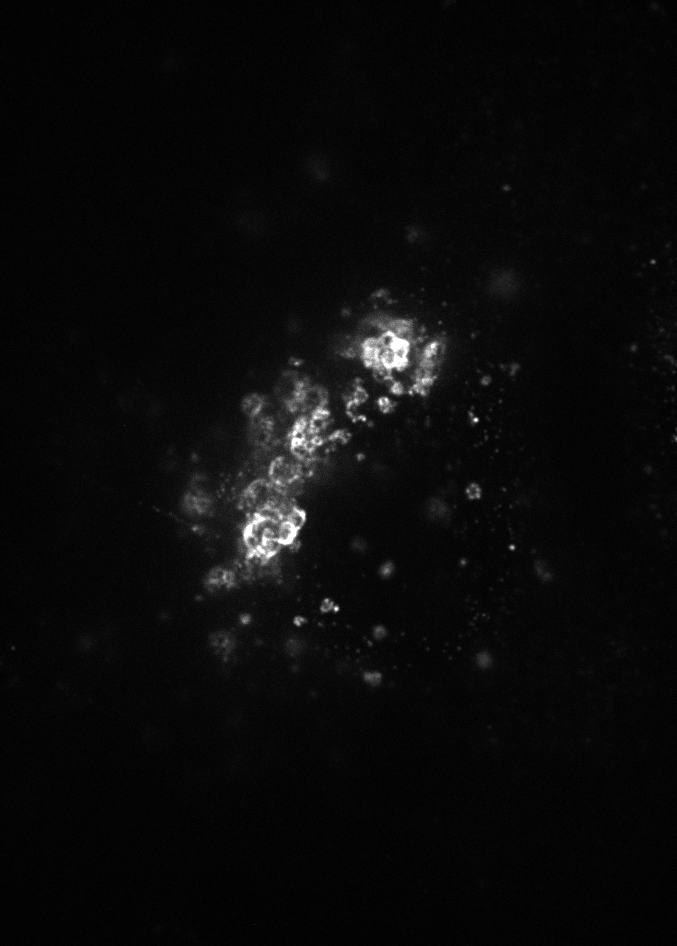

Supplement: Supplementary file 18 — Source data Fig. 8 [file 44318_2026_705_MOESM18_ESM.zip › Figure 8/D/20250113_MCF7STARD3S209A_NT_5_w1SPI 491 GFP.TIF]

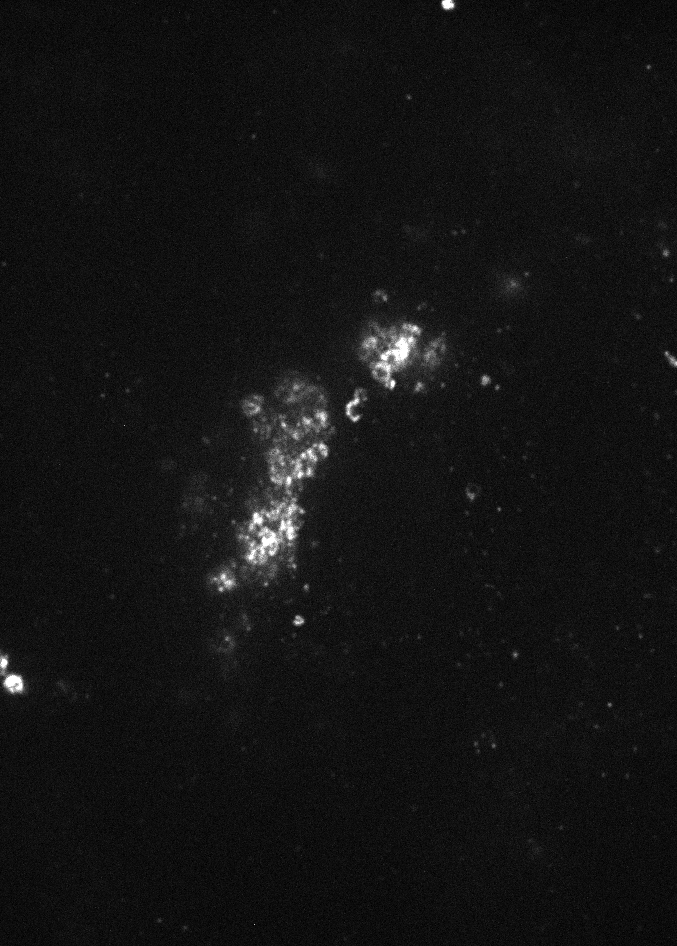

Supplement: Supplementary file 18 — Source data Fig. 8 [file 44318_2026_705_MOESM18_ESM.zip › Figure 8/D/20250113_MCF7STARD3S209A_NT_5_w2SPI 561 mCherry.TIF]

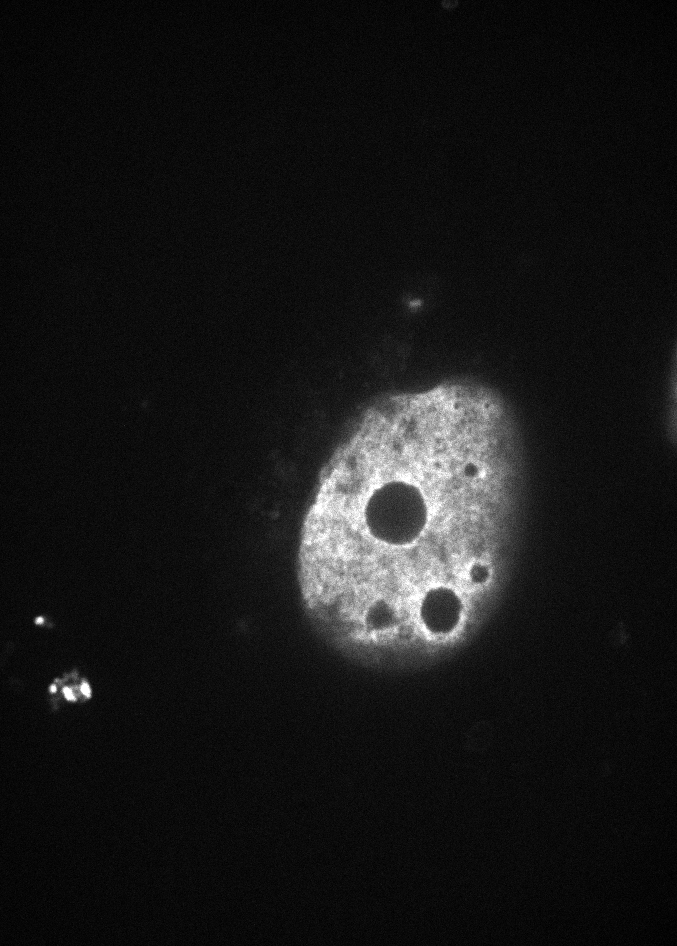

Supplement: Supplementary file 18 — Source data Fig. 8 [file 44318_2026_705_MOESM18_ESM.zip › Figure 8/D/20250113_MCF7STARD3S209A_NT_5_w3SPI 405 DAPI.TIF]

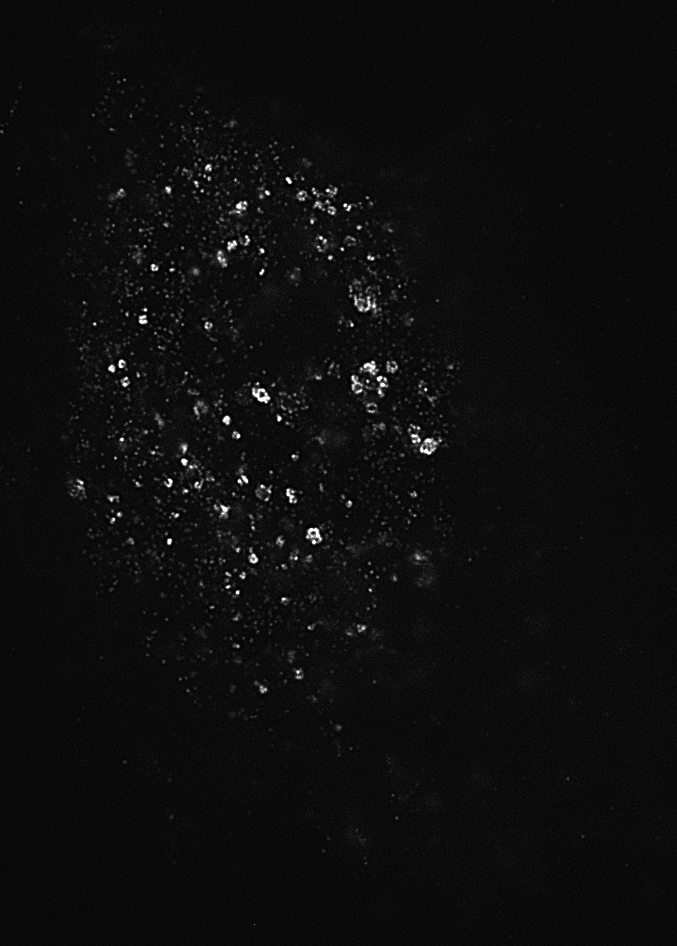

Supplement: Supplementary file 18 — Source data Fig. 8 [file 44318_2026_705_MOESM18_ESM.zip › Figure 8/E/20250114_MCF7STARD3S209AK2D2_NT_19_SR_w1SPI 491 GFP.TIF]

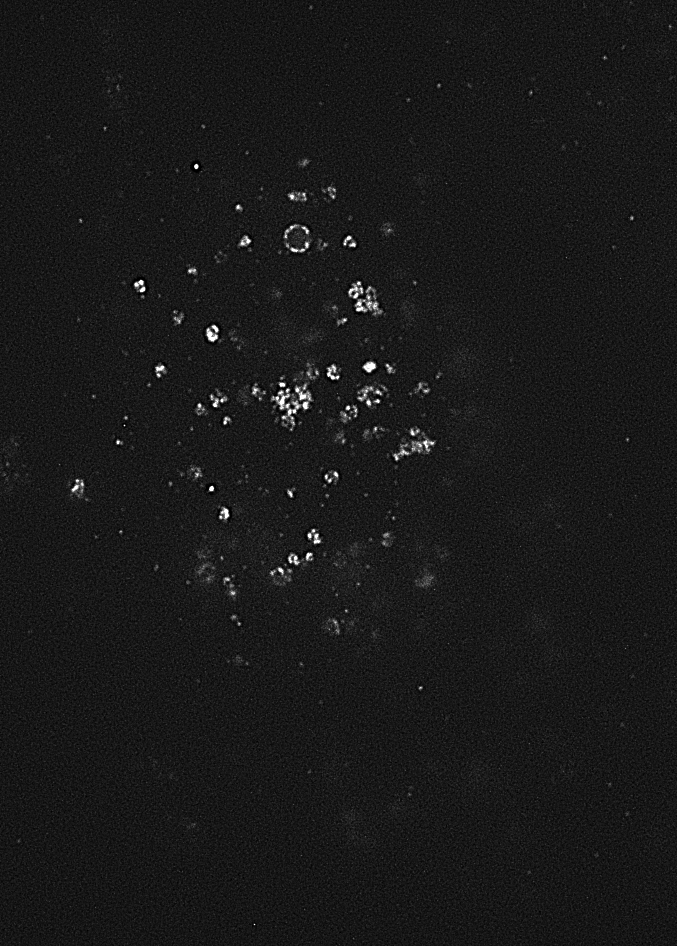

Supplement: Supplementary file 18 — Source data Fig. 8 [file 44318_2026_705_MOESM18_ESM.zip › Figure 8/E/20250114_MCF7STARD3S209AK2D2_NT_19_SR_w2SPI 561 mCherry.TIF]

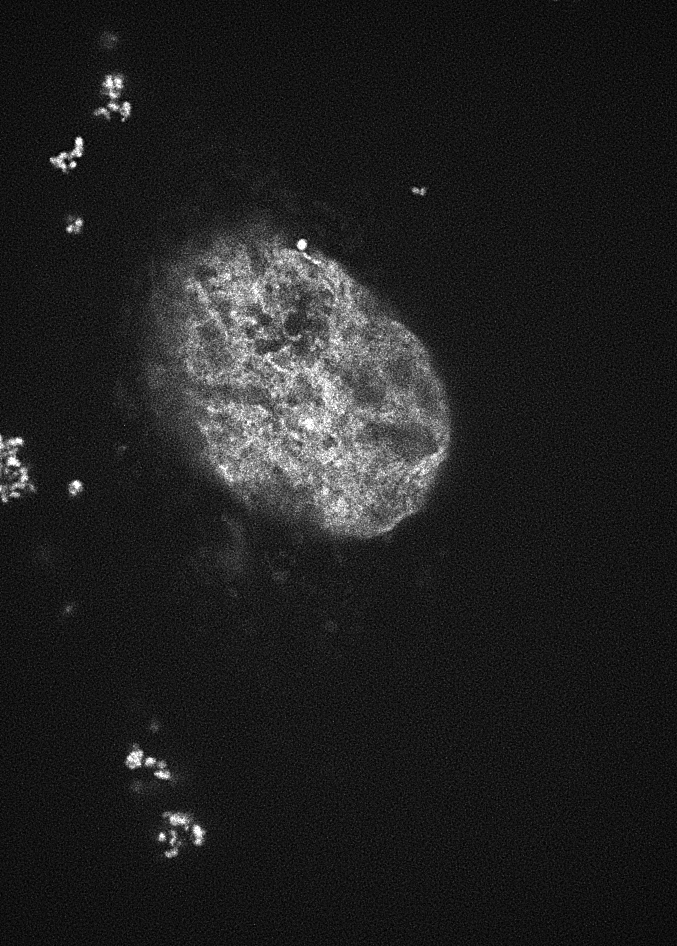

Supplement: Supplementary file 18 — Source data Fig. 8 [file 44318_2026_705_MOESM18_ESM.zip › Figure 8/E/20250114_MCF7STARD3S209AK2D2_NT_19_SR_w3SPI 405 DAPI.TIF]

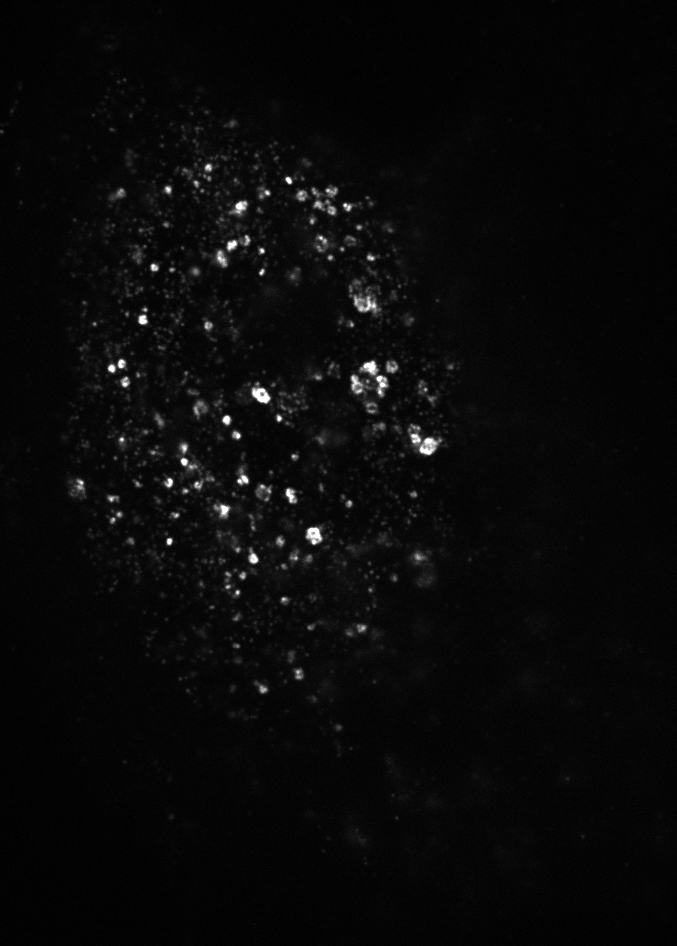

Supplement: Supplementary file 18 — Source data Fig. 8 [file 44318_2026_705_MOESM18_ESM.zip › Figure 8/E/20250114_MCF7STARD3S209AK2D2_NT_19_w1SPI 491 GFP.TIF]

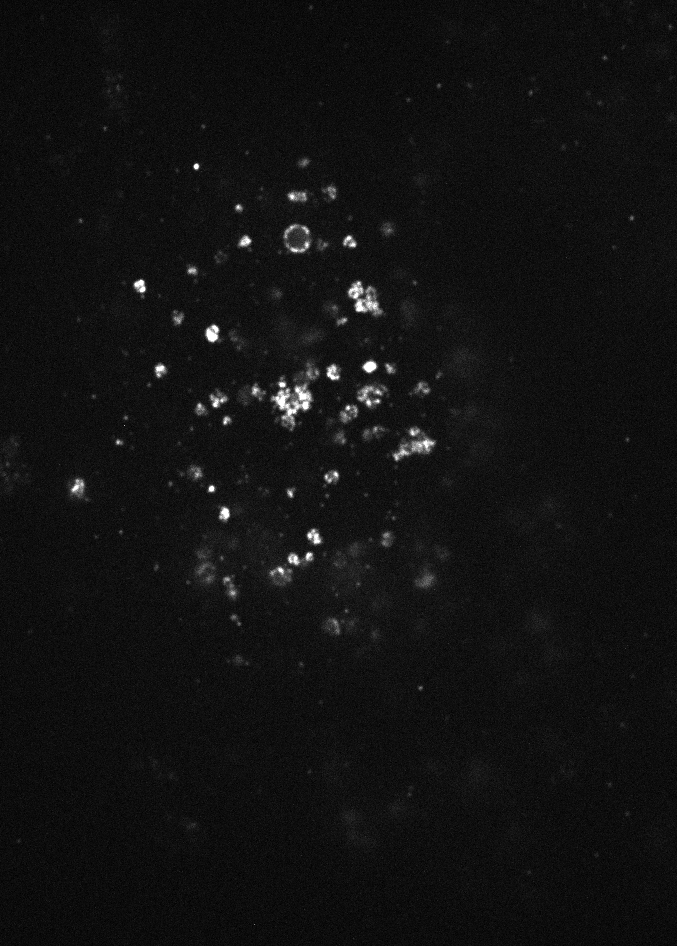

Supplement: Supplementary file 18 — Source data Fig. 8 [file 44318_2026_705_MOESM18_ESM.zip › Figure 8/E/20250114_MCF7STARD3S209AK2D2_NT_19_w2SPI 561 mCherry.TIF]

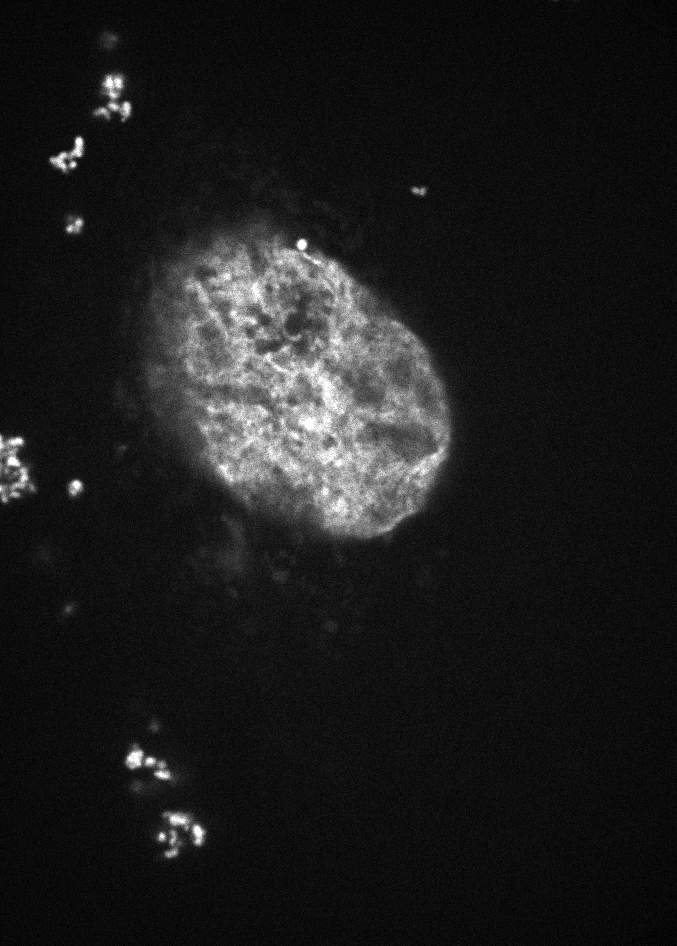

Supplement: Supplementary file 18 — Source data Fig. 8 [file 44318_2026_705_MOESM18_ESM.zip › Figure 8/E/20250114_MCF7STARD3S209AK2D2_NT_19_w3SPI 405 DAPI.TIF]

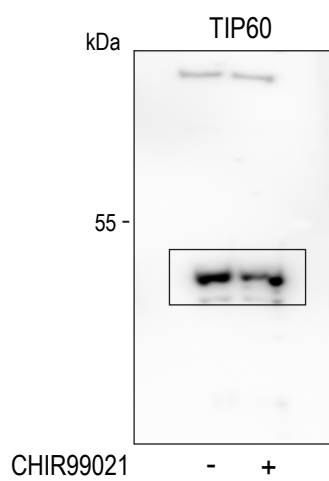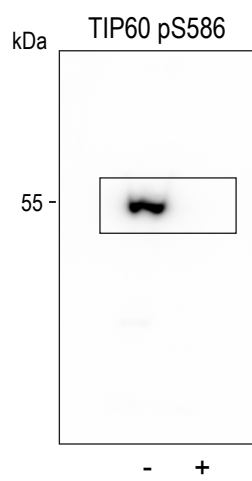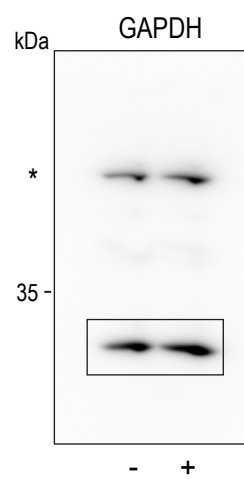

Supplement: Supplementary file 19 — Figure EV1 Source Data [file 44318_2026_705_MOESM19_ESM.zip › Figure EV1/A/Tip60_WB.pdf]

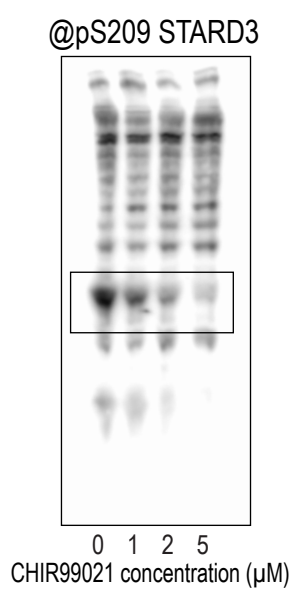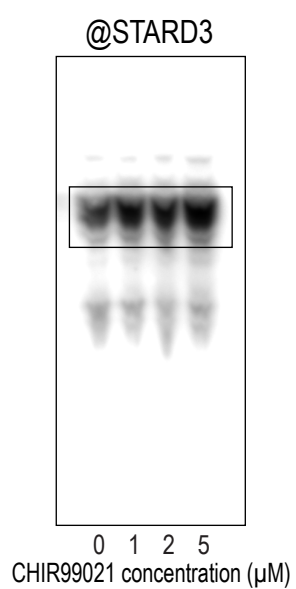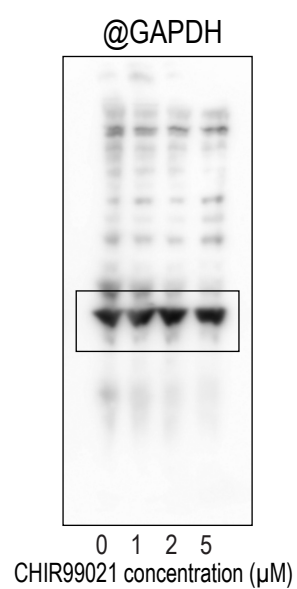

Supplement: Supplementary file 19 — Figure EV1 Source Data [file 44318_2026_705_MOESM19_ESM.zip › Figure EV1/B/Dose-response_WB.pdf]

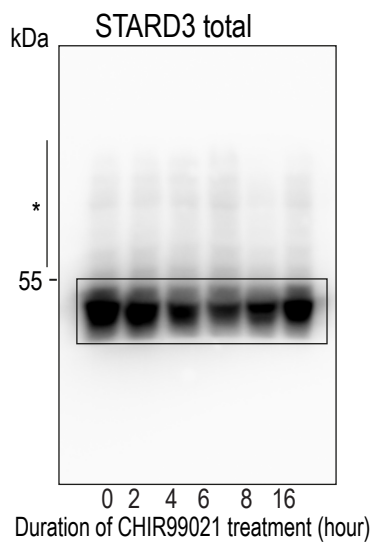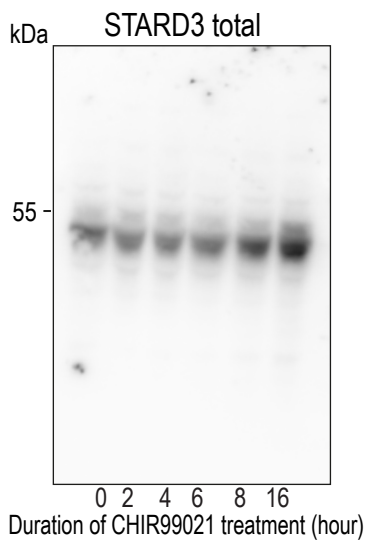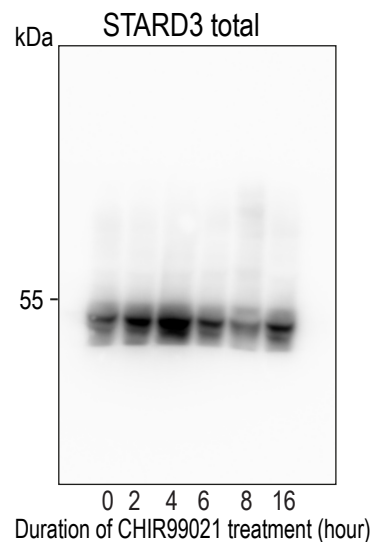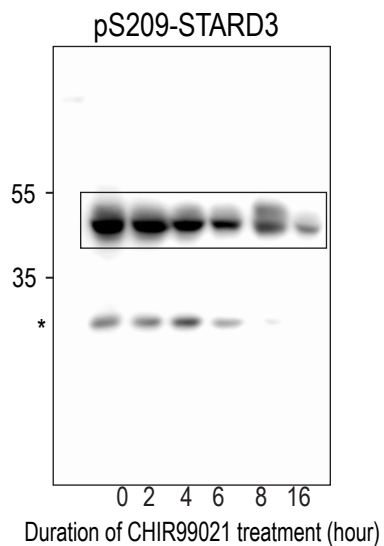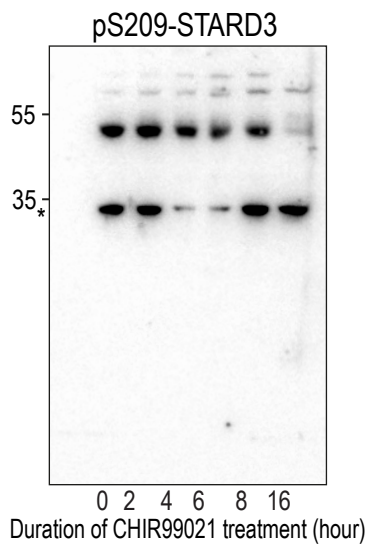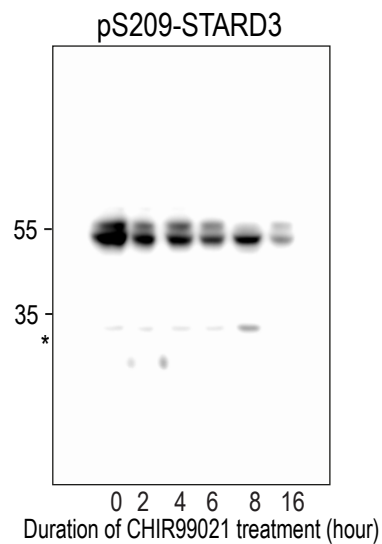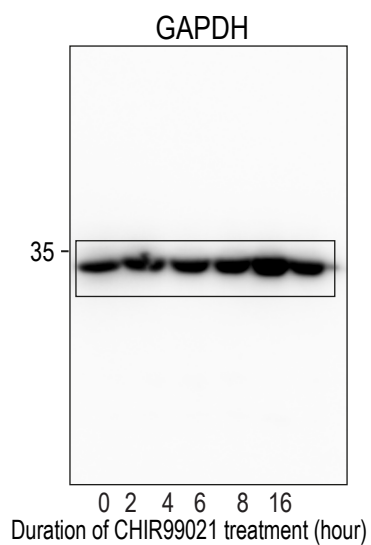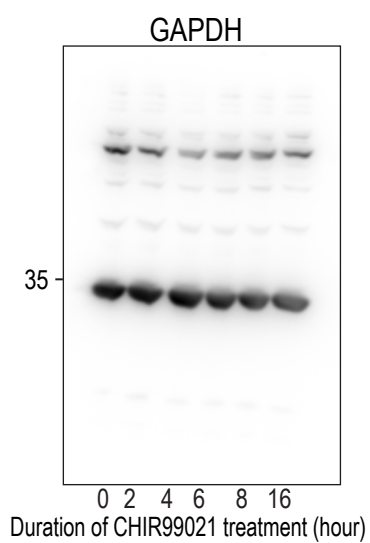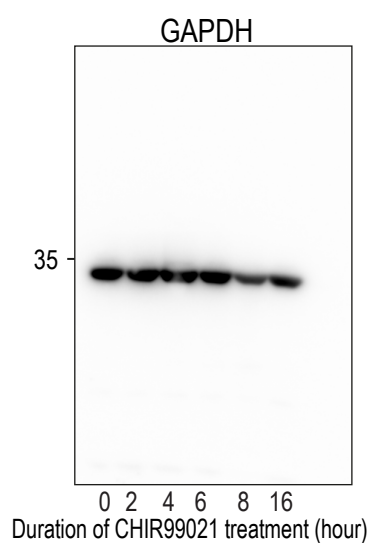

Supplement: Supplementary file 19 — Figure EV1 Source Data [file 44318_2026_705_MOESM19_ESM.zip › Figure EV1/C/a/HCC1954_dose_response_WB.pdf]

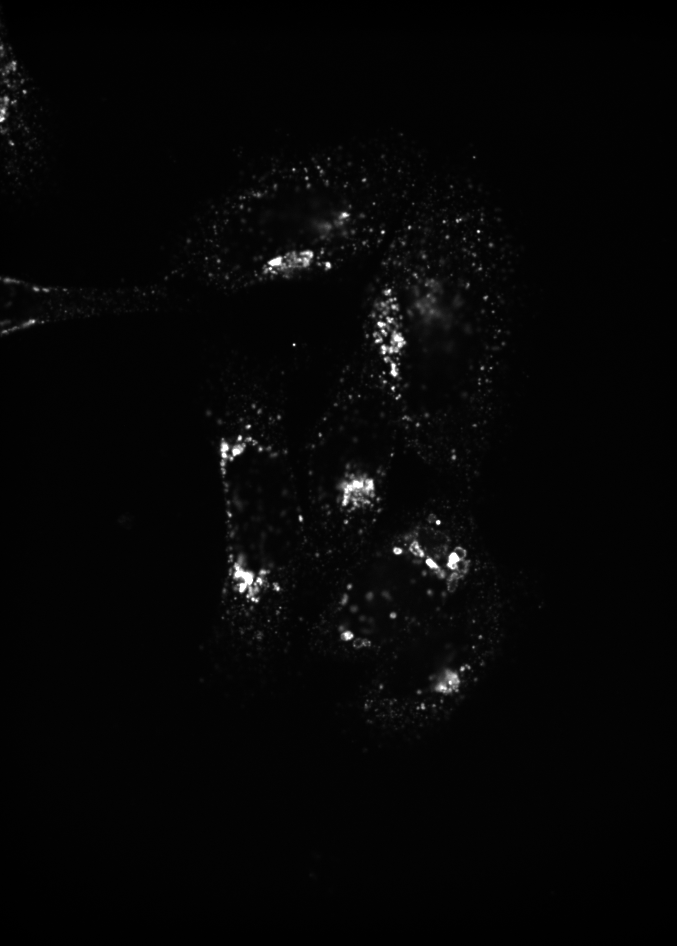

Supplement: Supplementary file 19 — Figure EV1 Source Data [file 44318_2026_705_MOESM19_ESM.zip › Figure EV1/C/c/16h/20230126_HCC_STARD3LAMP_ON_PL_1_w1SPI 491 GFP.TIF]

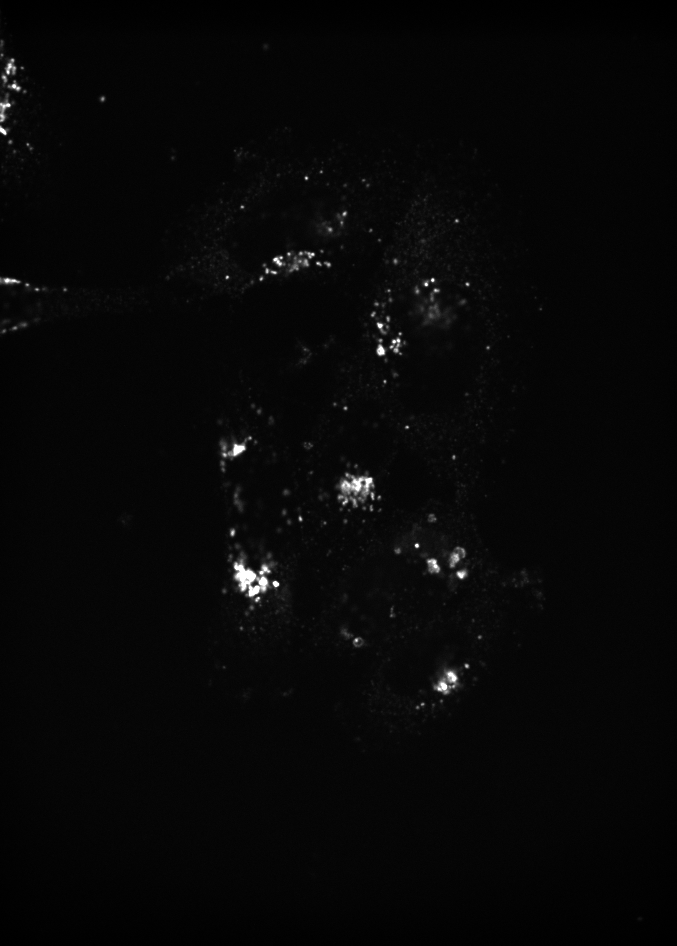

Supplement: Supplementary file 19 — Figure EV1 Source Data [file 44318_2026_705_MOESM19_ESM.zip › Figure EV1/C/c/16h/20230126_HCC_STARD3LAMP_ON_PL_1_w2SPI 561 mCherry.TIF]

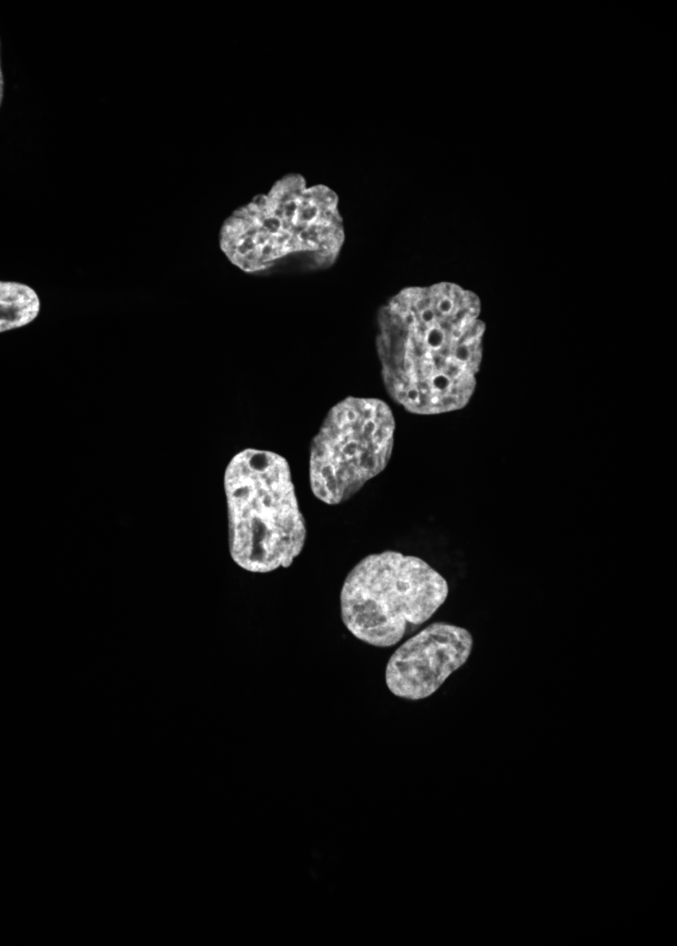

Supplement: Supplementary file 19 — Figure EV1 Source Data [file 44318_2026_705_MOESM19_ESM.zip › Figure EV1/C/c/16h/20230126_HCC_STARD3LAMP_ON_PL_1_w3SPI 405 DAPI.TIF]

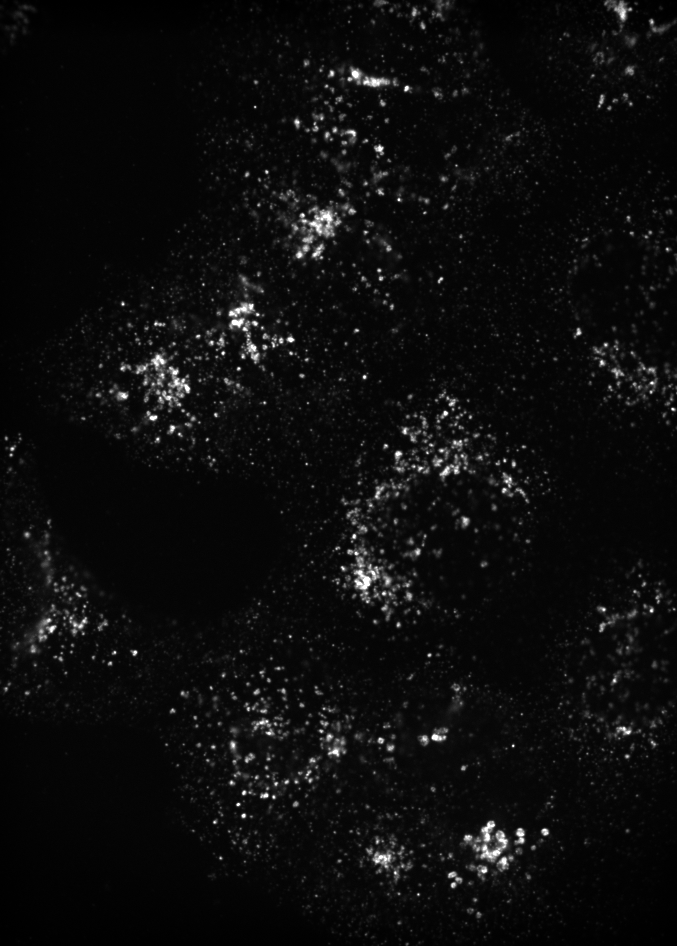

Supplement: Supplementary file 19 — Figure EV1 Source Data [file 44318_2026_705_MOESM19_ESM.zip › Figure EV1/C/c/2h/20240126_HCC_LAMPSTARD3_2h_3_w1SPI 491 GFP.TIF]

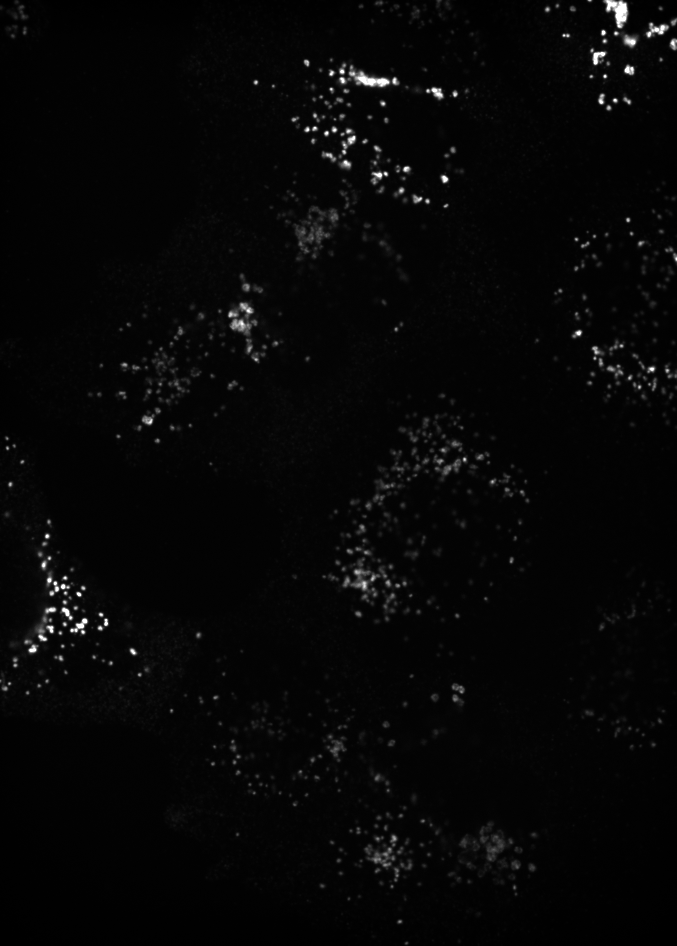

Supplement: Supplementary file 19 — Figure EV1 Source Data [file 44318_2026_705_MOESM19_ESM.zip › Figure EV1/C/c/2h/20240126_HCC_LAMPSTARD3_2h_3_w2SPI 561 mCherry.TIF]
