# Supplementary figures and images for: STARD3 regulates lysosome positioning and contacts via a GSK3-controlled phosphorylation switch (part 4 of 7)
Source: EMBO J. 2026 Feb 25;45(7):2239–77. doi: 10.1038/s44318-026-00705-3 (PMC13044316; doi:10.1038/s44318-026-00705-3)

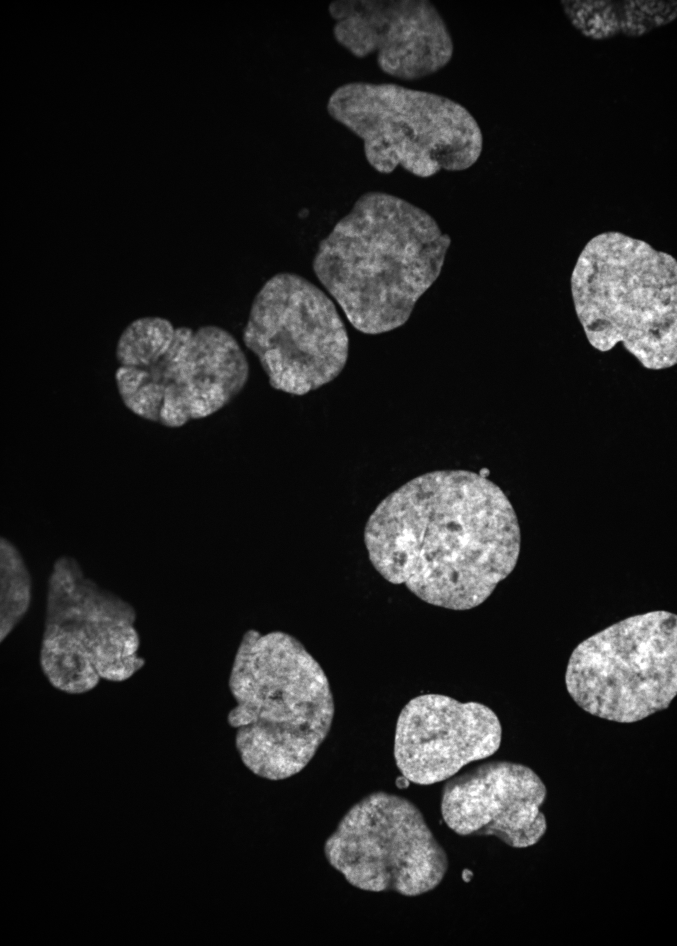

Supplement: Supplementary file 19 — Figure EV1 Source Data [file 44318_2026_705_MOESM19_ESM.zip › Figure EV1/C/c/2h/20240126_HCC_LAMPSTARD3_2h_3_w3SPI 405 DAPI.TIF]

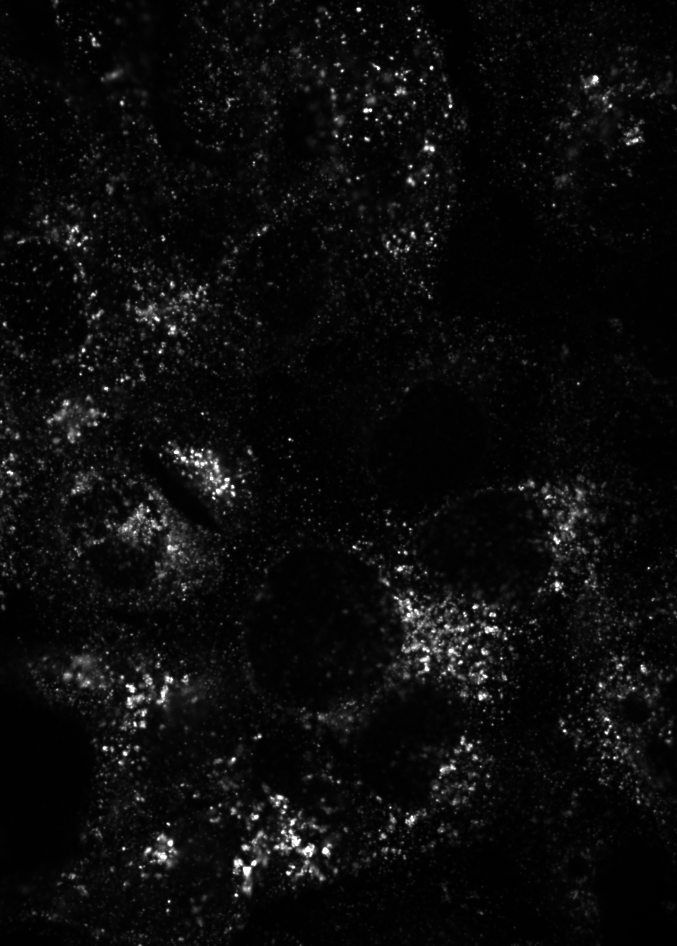

Supplement: Supplementary file 19 — Figure EV1 Source Data [file 44318_2026_705_MOESM19_ESM.zip › Figure EV1/C/c/4h/20240129_HCC_LAMPSTARD3_4h_PL_1_w1SPI 491 GFP.TIF]

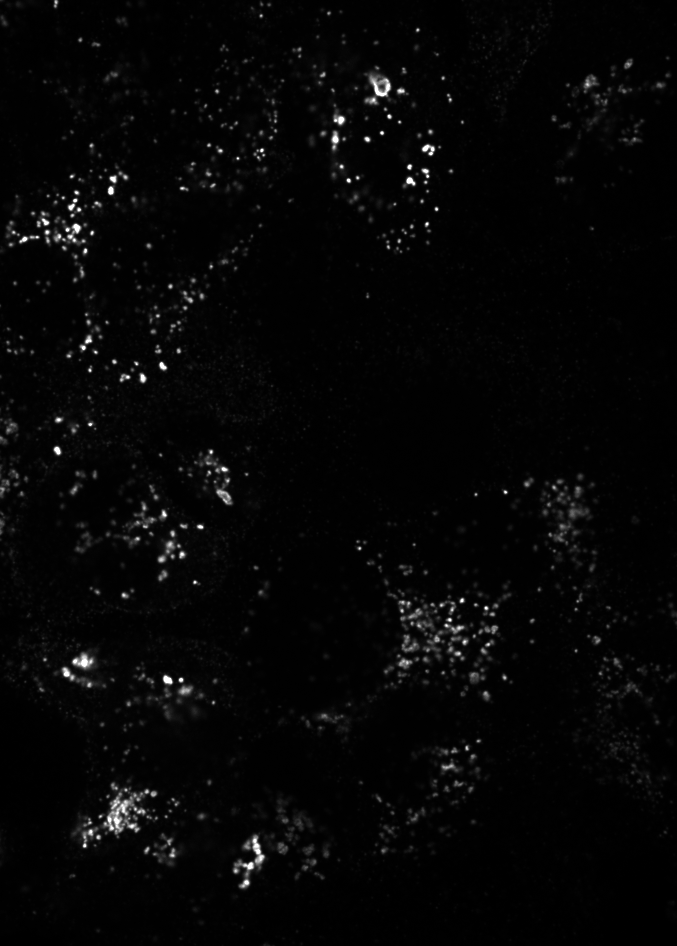

Supplement: Supplementary file 19 — Figure EV1 Source Data [file 44318_2026_705_MOESM19_ESM.zip › Figure EV1/C/c/4h/20240129_HCC_LAMPSTARD3_4h_PL_1_w2SPI 561 mCherry.TIF]

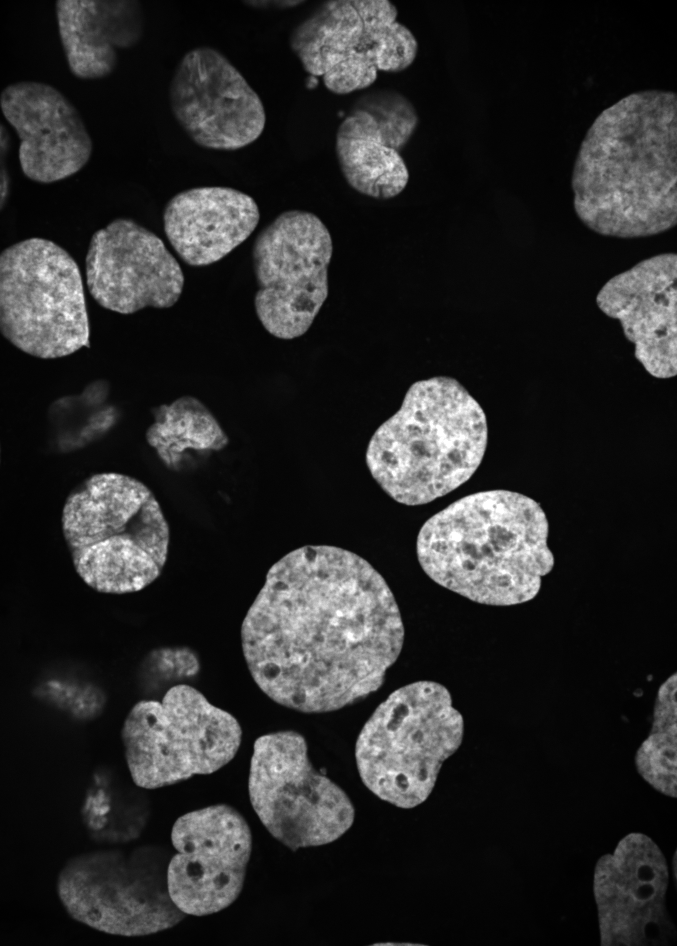

Supplement: Supplementary file 19 — Figure EV1 Source Data [file 44318_2026_705_MOESM19_ESM.zip › Figure EV1/C/c/4h/20240129_HCC_LAMPSTARD3_4h_PL_1_w3SPI 405 DAPI.TIF]

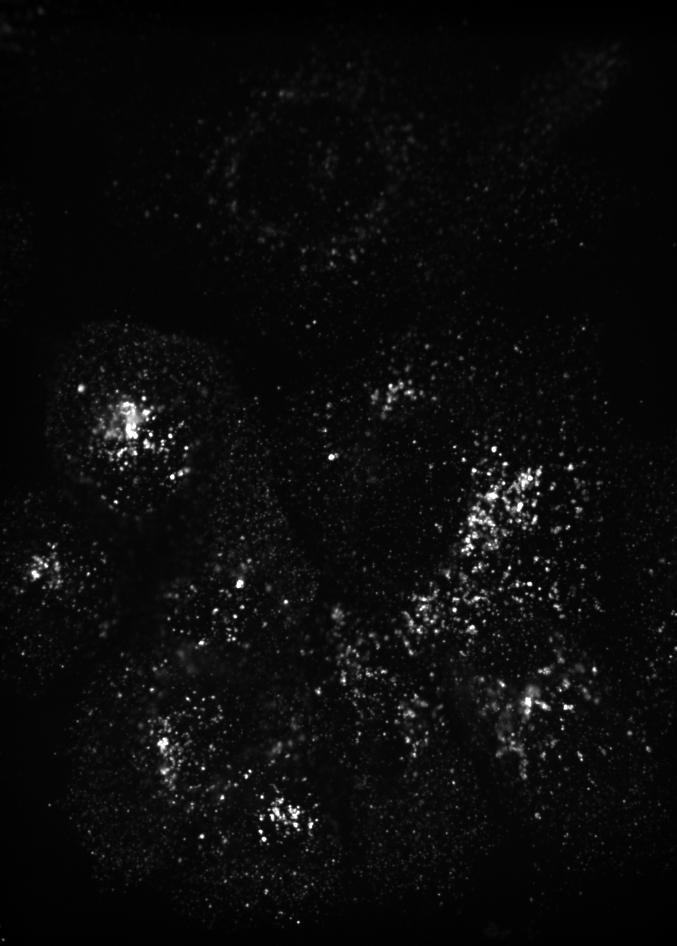

Supplement: Supplementary file 19 — Figure EV1 Source Data [file 44318_2026_705_MOESM19_ESM.zip › Figure EV1/C/c/6h/20240126_HCC_LAMPSTARD3_6h_PL_1_w1SPI 491 GFP.TIF]

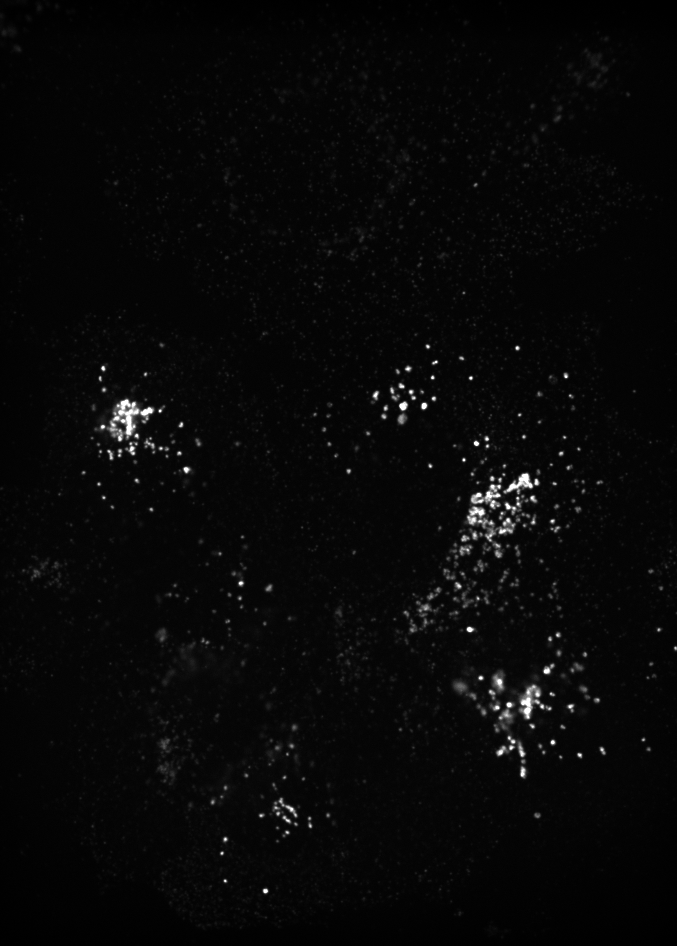

Supplement: Supplementary file 19 — Figure EV1 Source Data [file 44318_2026_705_MOESM19_ESM.zip › Figure EV1/C/c/6h/20240126_HCC_LAMPSTARD3_6h_PL_1_w2SPI 561 mCherry.TIF]

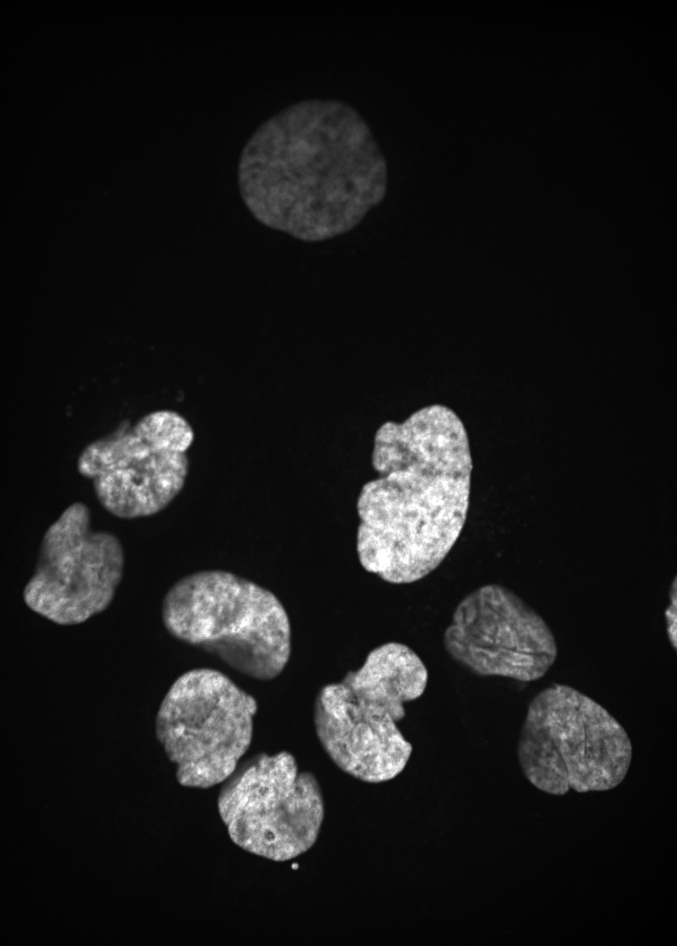

Supplement: Supplementary file 19 — Figure EV1 Source Data [file 44318_2026_705_MOESM19_ESM.zip › Figure EV1/C/c/6h/20240126_HCC_LAMPSTARD3_6h_PL_1_w3SPI 405 DAPI.TIF]

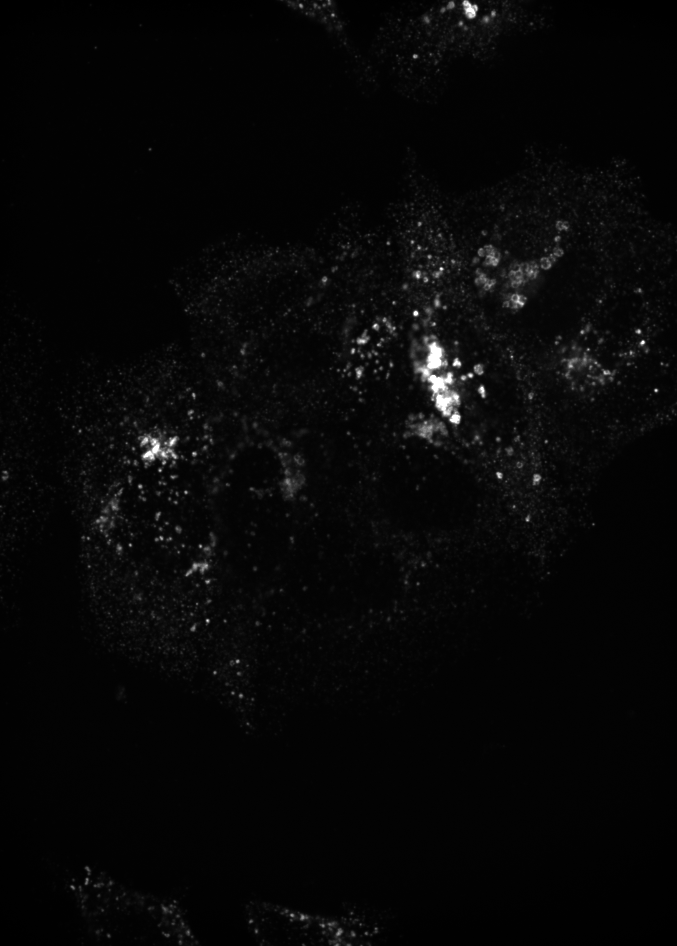

Supplement: Supplementary file 19 — Figure EV1 Source Data [file 44318_2026_705_MOESM19_ESM.zip › Figure EV1/C/c/8h/20240126_HCC_LAMPSTARD3_8h_PL_2_w1SPI 491 GFP.TIF]

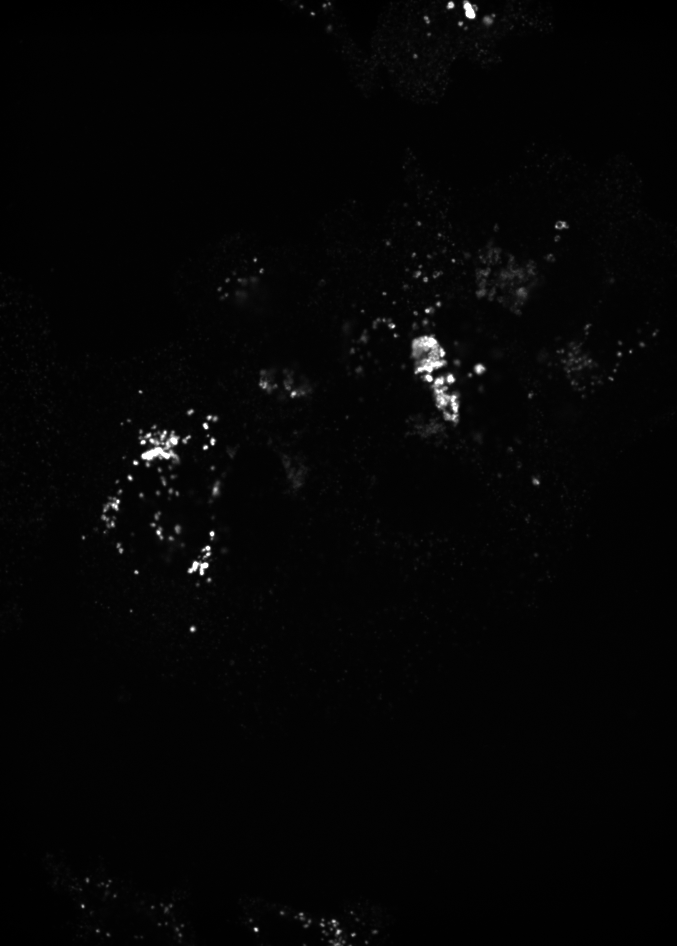

Supplement: Supplementary file 19 — Figure EV1 Source Data [file 44318_2026_705_MOESM19_ESM.zip › Figure EV1/C/c/8h/20240126_HCC_LAMPSTARD3_8h_PL_2_w2SPI 561 mCherry.TIF]

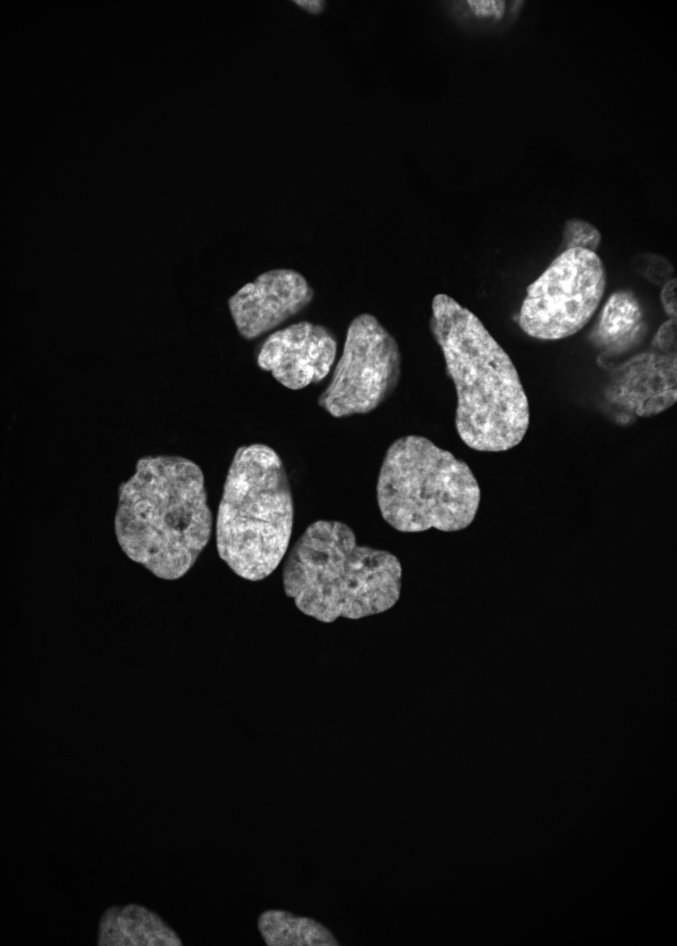

Supplement: Supplementary file 19 — Figure EV1 Source Data [file 44318_2026_705_MOESM19_ESM.zip › Figure EV1/C/c/8h/20240126_HCC_LAMPSTARD3_8h_PL_2_w3SPI 405 DAPI.TIF]

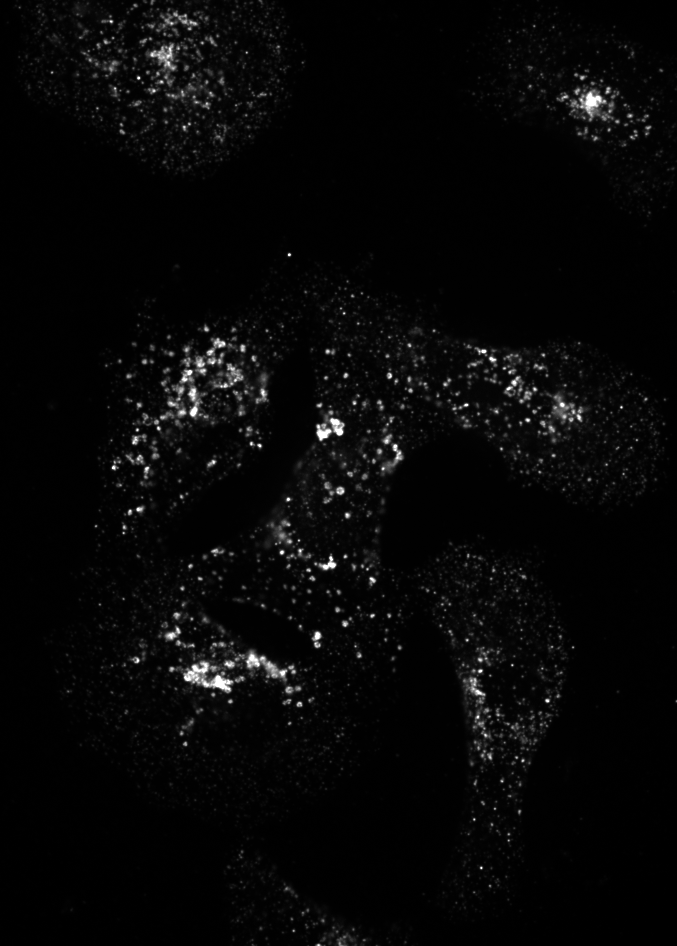

Supplement: Supplementary file 19 — Figure EV1 Source Data [file 44318_2026_705_MOESM19_ESM.zip › Figure EV1/C/c/NT/20230126_HCC_STARD3LAMP_NT_PL_1_w1SPI 491 GFP.TIF]

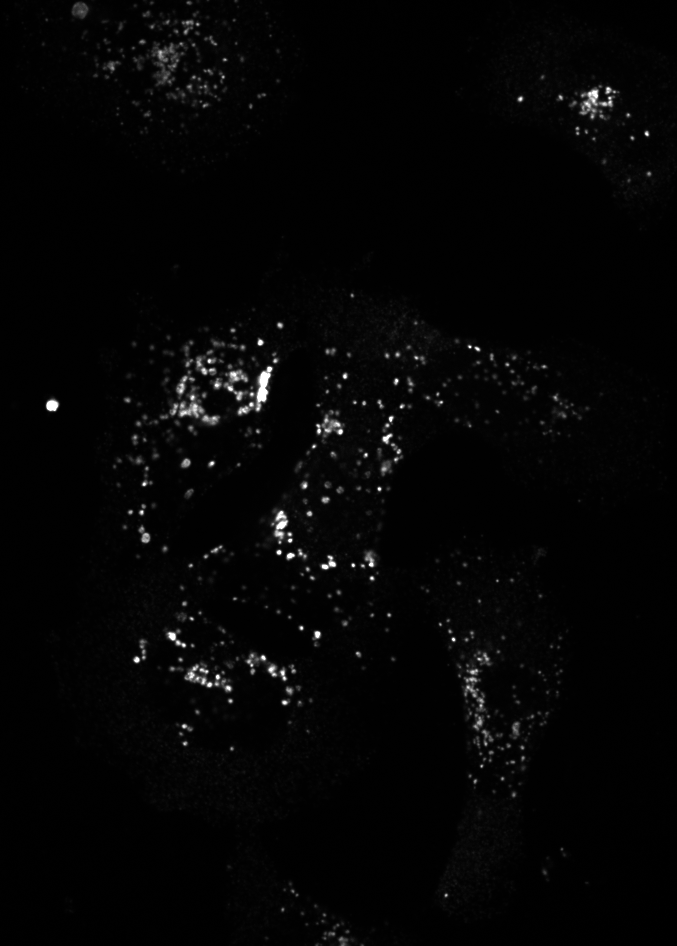

Supplement: Supplementary file 19 — Figure EV1 Source Data [file 44318_2026_705_MOESM19_ESM.zip › Figure EV1/C/c/NT/20230126_HCC_STARD3LAMP_NT_PL_1_w2SPI 561 mCherry.TIF]

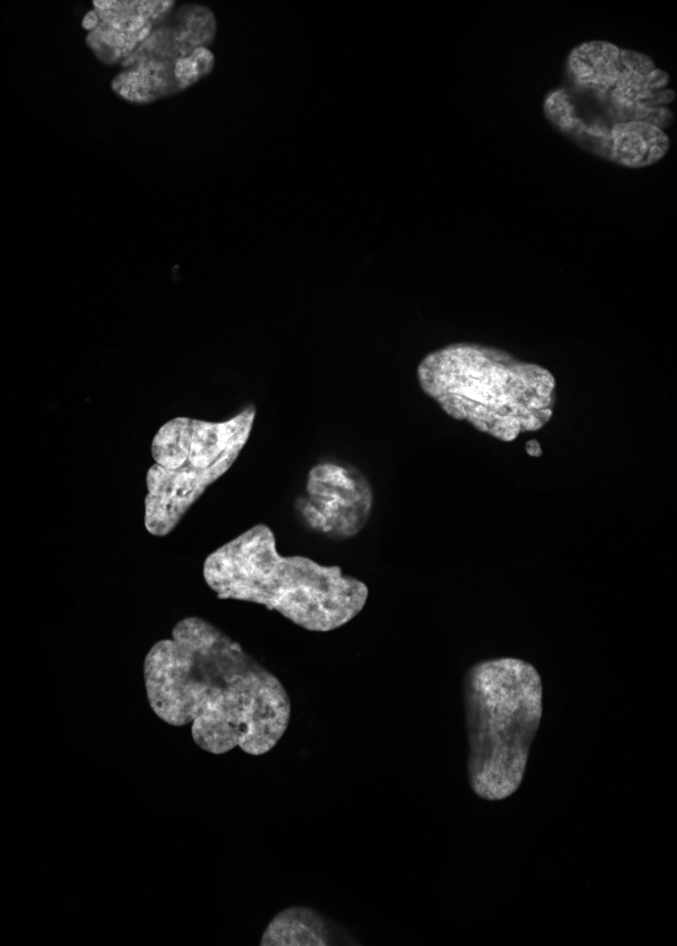

Supplement: Supplementary file 19 — Figure EV1 Source Data [file 44318_2026_705_MOESM19_ESM.zip › Figure EV1/C/c/NT/20230126_HCC_STARD3LAMP_NT_PL_1_w3SPI 405 DAPI.TIF]

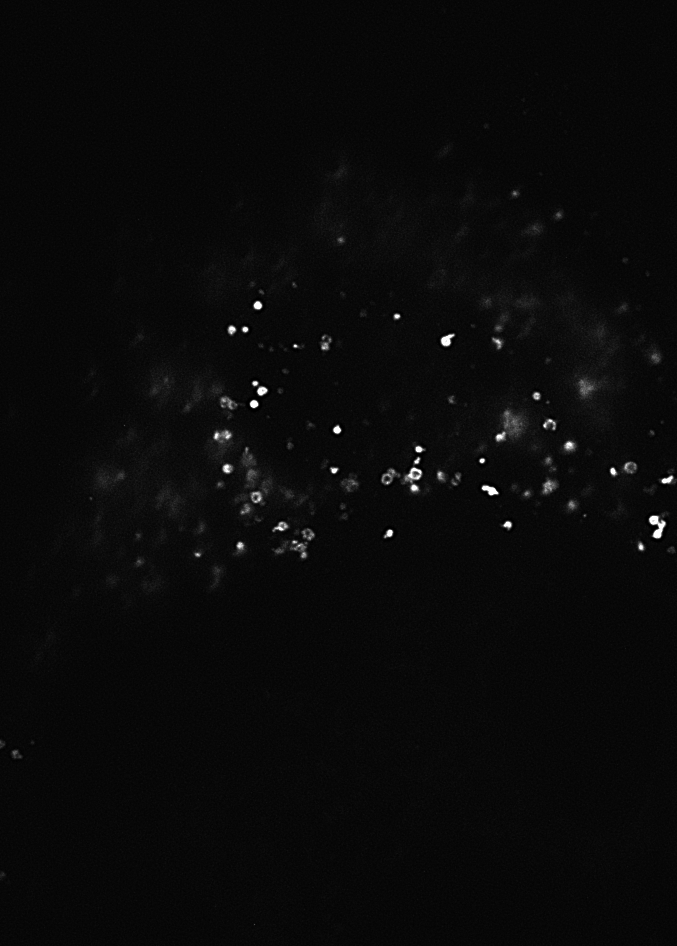

Supplement: Supplementary file 20 — Figure EV2 Source Data [file 44318_2026_705_MOESM20_ESM.zip › Figure EV2/A/20250828_MCF7msER_TMEMGFP_NT_2_SR_w1SPI 491 GFP.TIF]

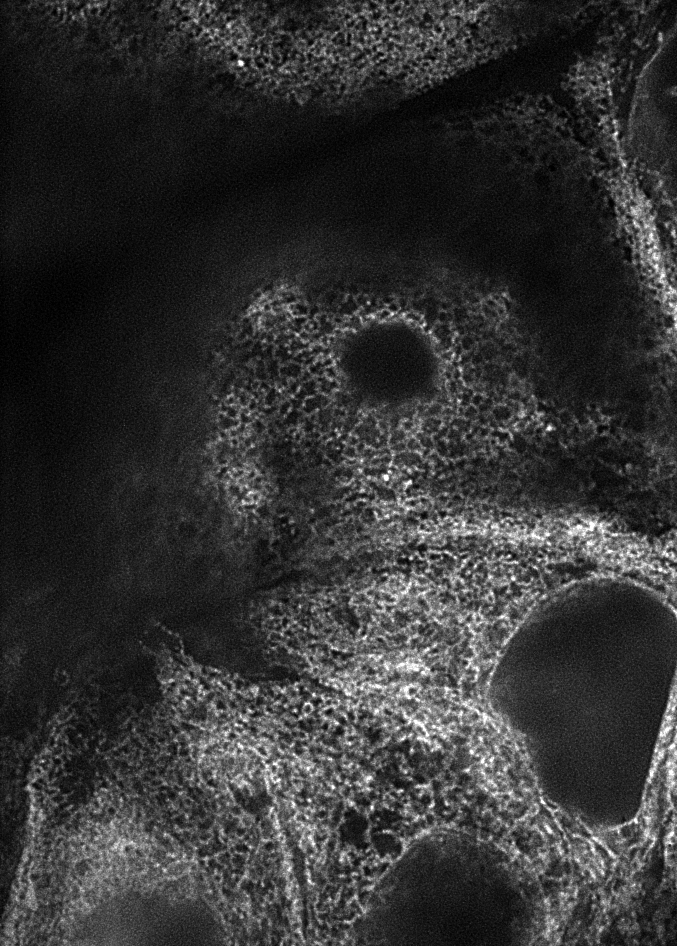

Supplement: Supplementary file 20 — Figure EV2 Source Data [file 44318_2026_705_MOESM20_ESM.zip › Figure EV2/A/20250828_MCF7msER_TMEMGFP_NT_2_SR_w2SPI 561 mCherry.TIF]

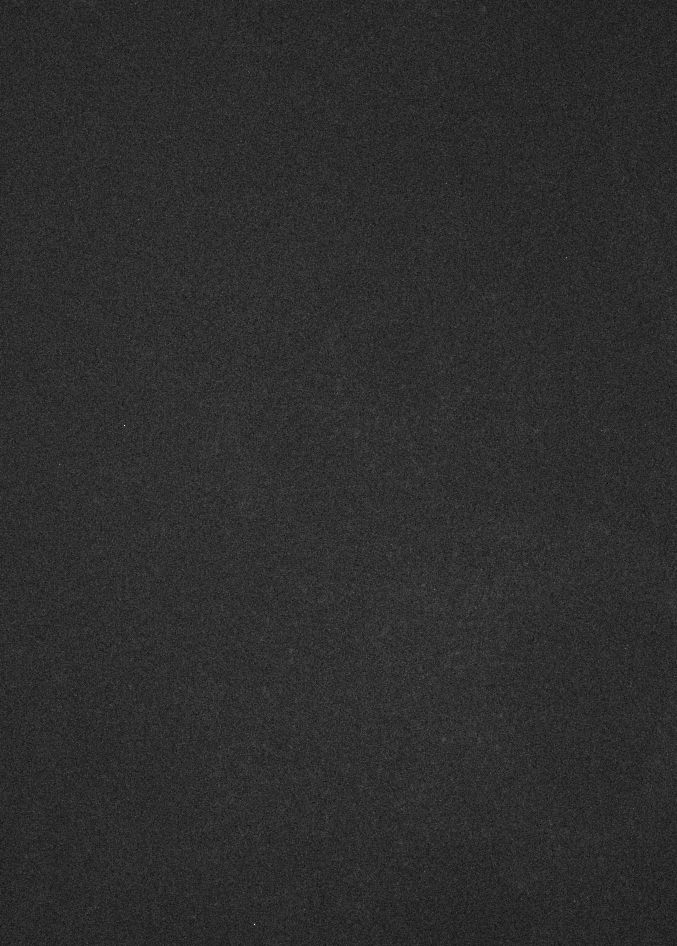

Supplement: Supplementary file 20 — Figure EV2 Source Data [file 44318_2026_705_MOESM20_ESM.zip › Figure EV2/A/20250828_MCF7msER_TMEMGFP_NT_2_SR_w3SPI 635 CY5 .TIF]

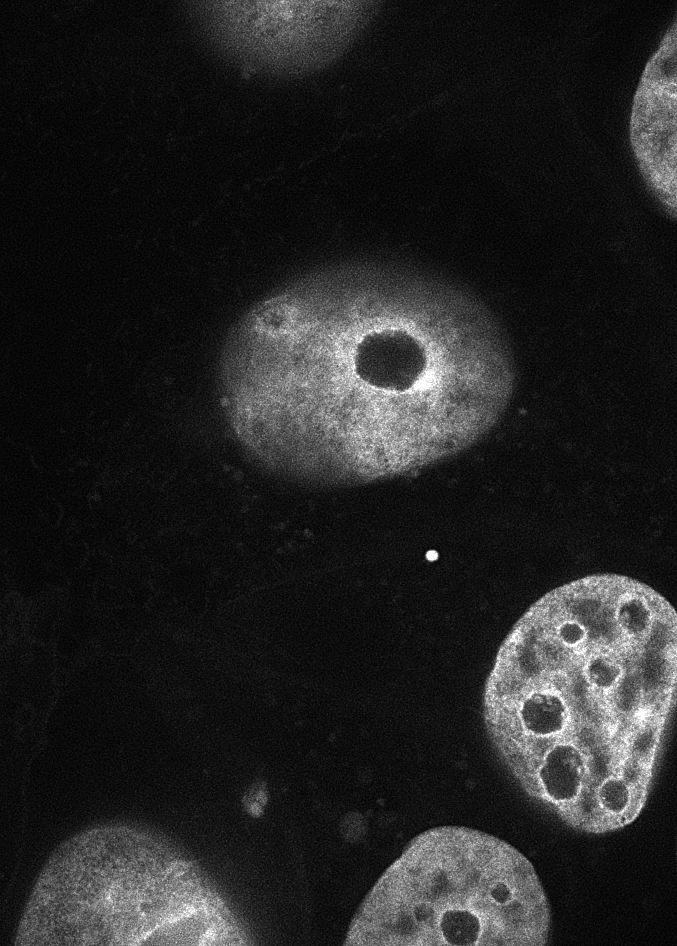

Supplement: Supplementary file 20 — Figure EV2 Source Data [file 44318_2026_705_MOESM20_ESM.zip › Figure EV2/A/20250828_MCF7msER_TMEMGFP_NT_2_SR_w4SPI 405 DAPI.TIF]

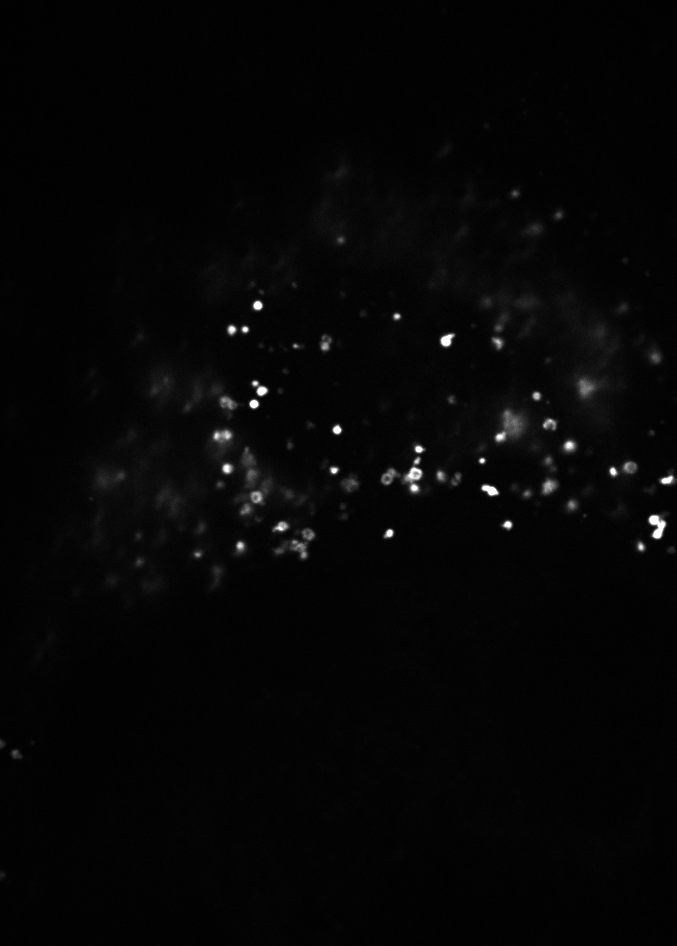

Supplement: Supplementary file 20 — Figure EV2 Source Data [file 44318_2026_705_MOESM20_ESM.zip › Figure EV2/A/20250828_MCF7msER_TMEMGFP_NT_2_w1SPI 491 GFP.TIF]

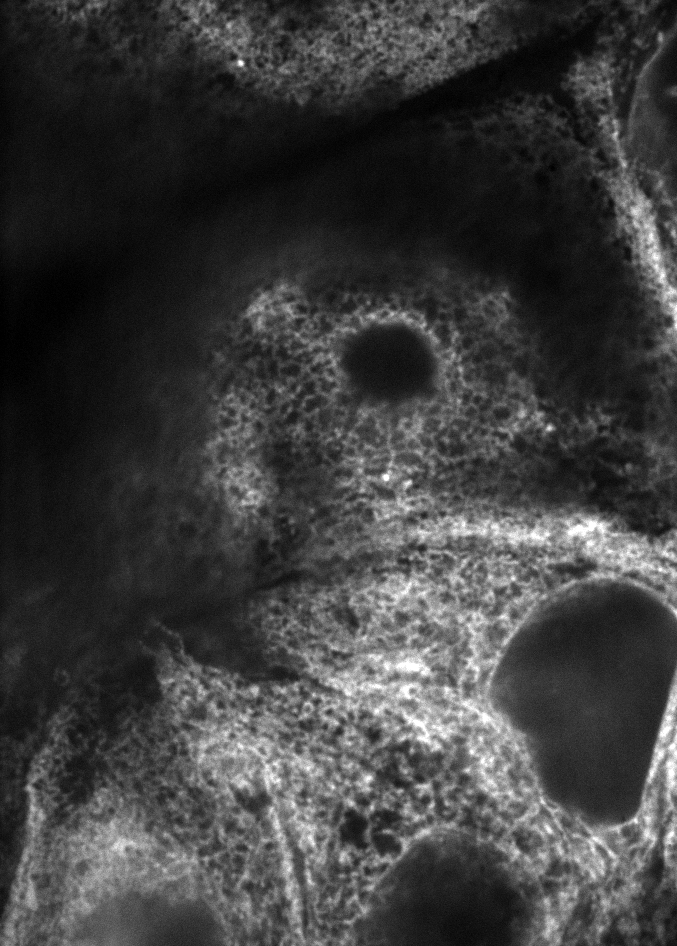

Supplement: Supplementary file 20 — Figure EV2 Source Data [file 44318_2026_705_MOESM20_ESM.zip › Figure EV2/A/20250828_MCF7msER_TMEMGFP_NT_2_w2SPI 561 mCherry.TIF]

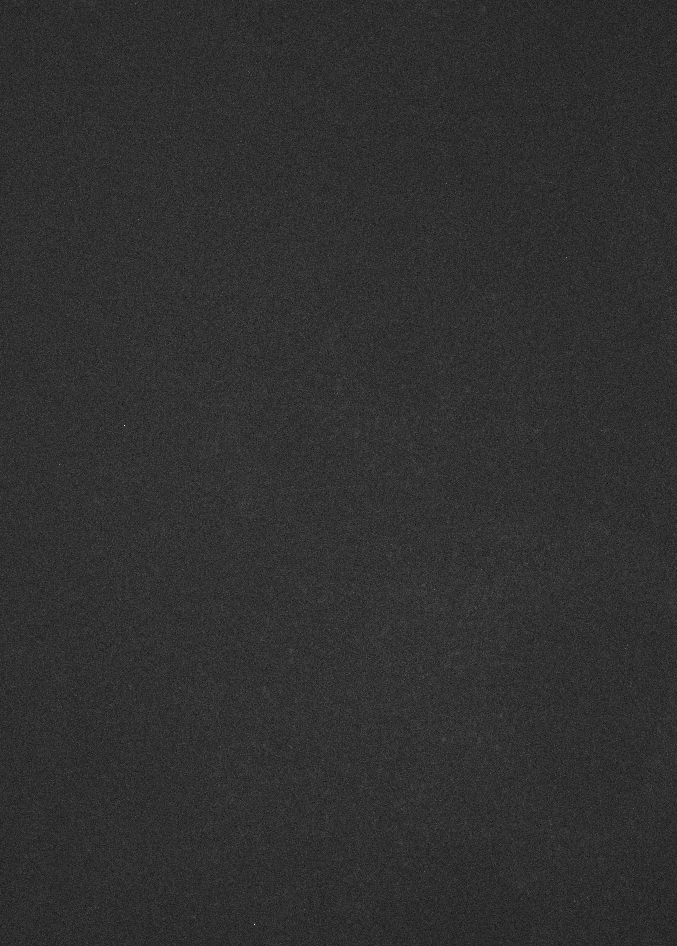

Supplement: Supplementary file 20 — Figure EV2 Source Data [file 44318_2026_705_MOESM20_ESM.zip › Figure EV2/A/20250828_MCF7msER_TMEMGFP_NT_2_w3SPI 635 CY5 .TIF]

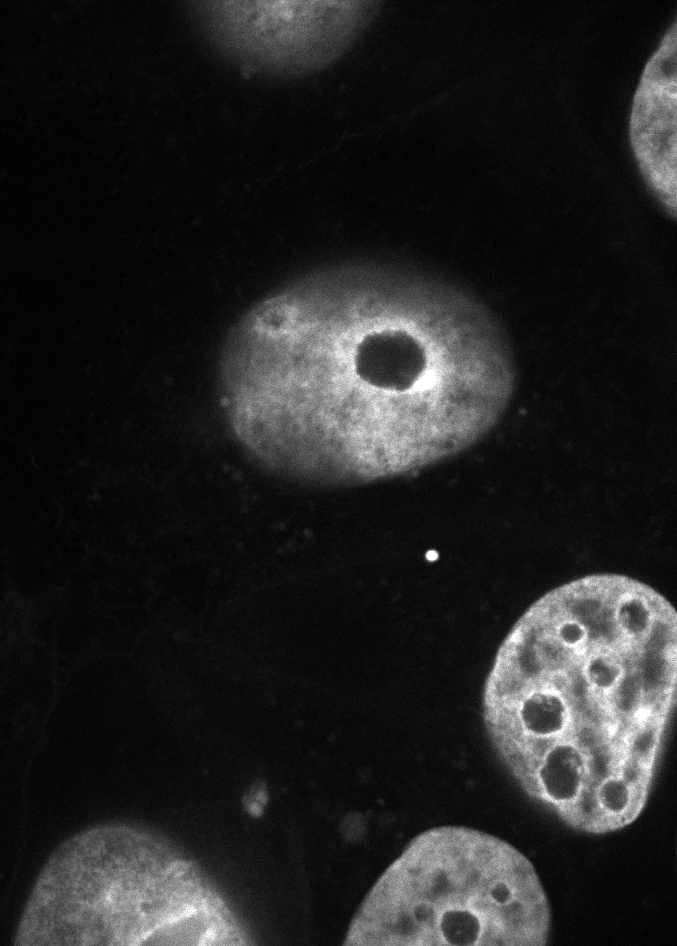

Supplement: Supplementary file 20 — Figure EV2 Source Data [file 44318_2026_705_MOESM20_ESM.zip › Figure EV2/A/20250828_MCF7msER_TMEMGFP_NT_2_w4SPI 405 DAPI.TIF]

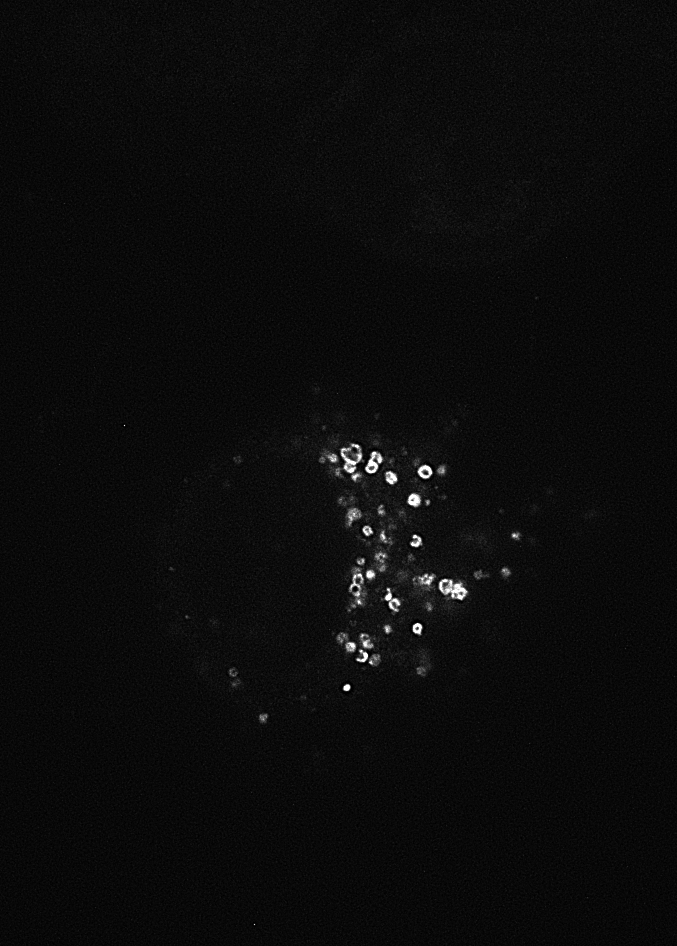

Supplement: Supplementary file 20 — Figure EV2 Source Data [file 44318_2026_705_MOESM20_ESM.zip › Figure EV2/B/20250830_MCF7msER_TMEMGFP_CHIR_5_SR_w1SPI 491 GFP.TIF]

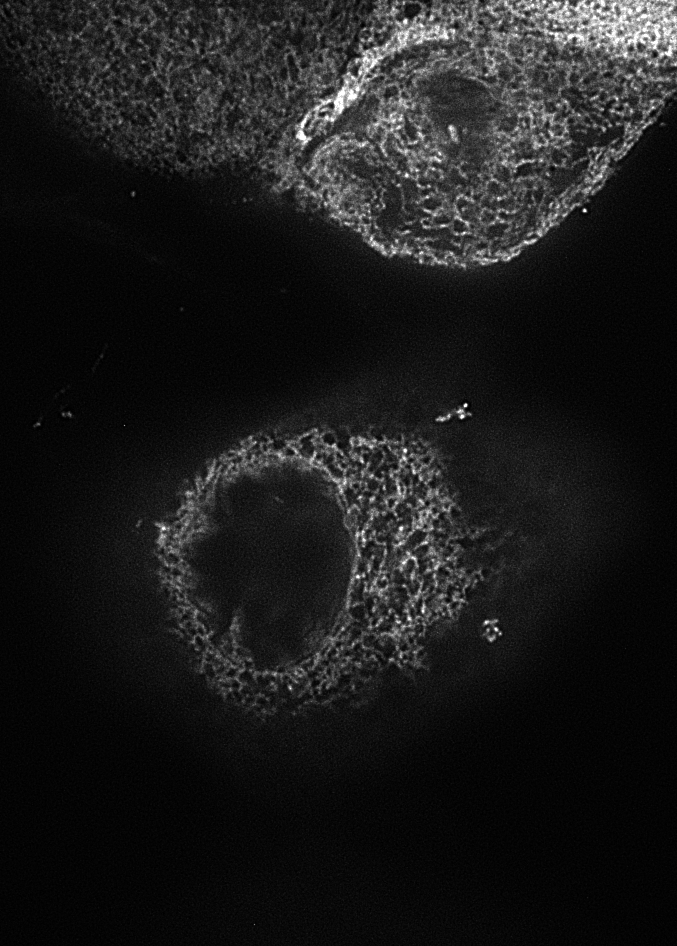

Supplement: Supplementary file 20 — Figure EV2 Source Data [file 44318_2026_705_MOESM20_ESM.zip › Figure EV2/B/20250830_MCF7msER_TMEMGFP_CHIR_5_SR_w2SPI 561 mCherry.TIF]

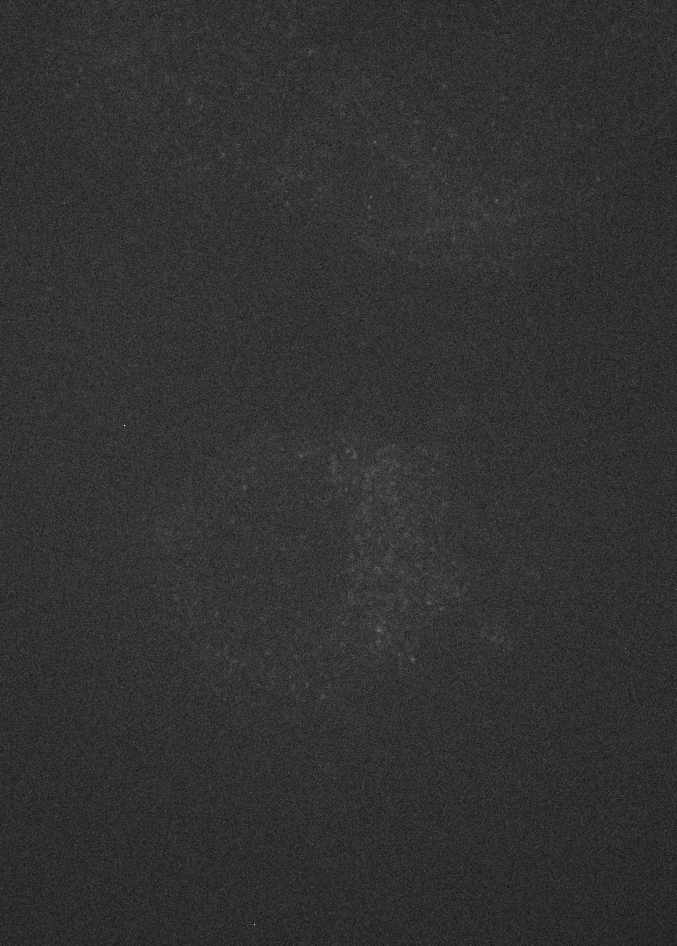

Supplement: Supplementary file 20 — Figure EV2 Source Data [file 44318_2026_705_MOESM20_ESM.zip › Figure EV2/B/20250830_MCF7msER_TMEMGFP_CHIR_5_SR_w3SPI 635 CY5 .TIF]

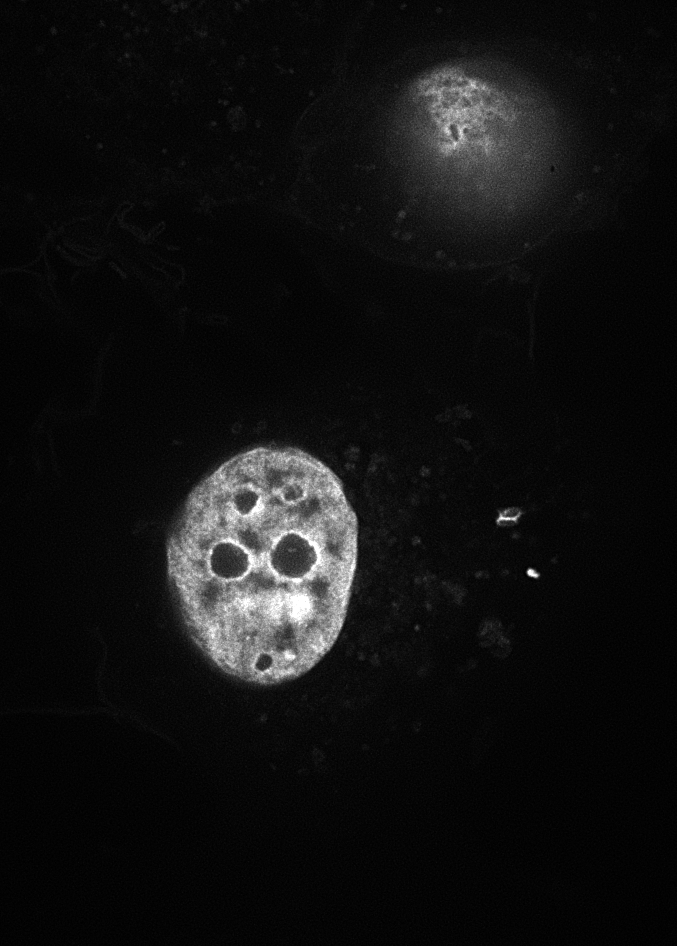

Supplement: Supplementary file 20 — Figure EV2 Source Data [file 44318_2026_705_MOESM20_ESM.zip › Figure EV2/B/20250830_MCF7msER_TMEMGFP_CHIR_5_SR_w4SPI 405 DAPI.TIF]

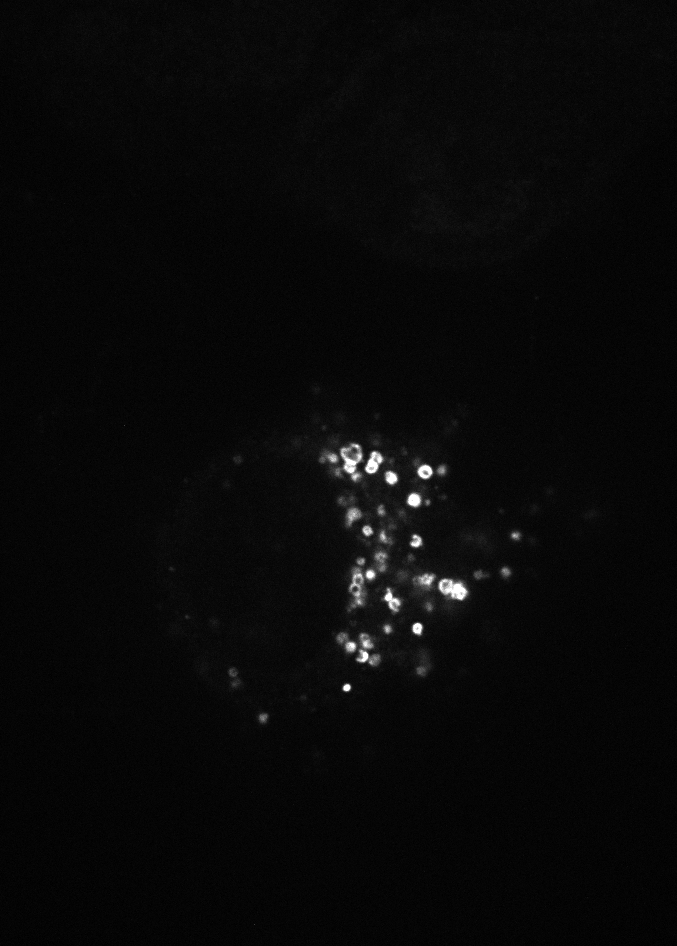

Supplement: Supplementary file 20 — Figure EV2 Source Data [file 44318_2026_705_MOESM20_ESM.zip › Figure EV2/B/20250830_MCF7msER_TMEMGFP_CHIR_5_w1SPI 491 GFP.TIF]

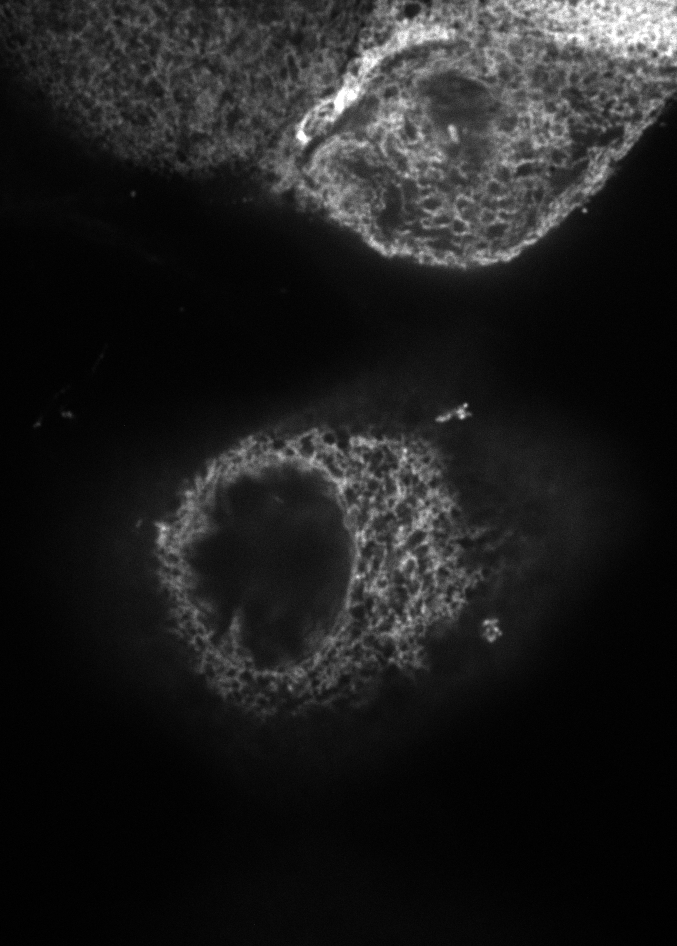

Supplement: Supplementary file 20 — Figure EV2 Source Data [file 44318_2026_705_MOESM20_ESM.zip › Figure EV2/B/20250830_MCF7msER_TMEMGFP_CHIR_5_w2SPI 561 mCherry.TIF]

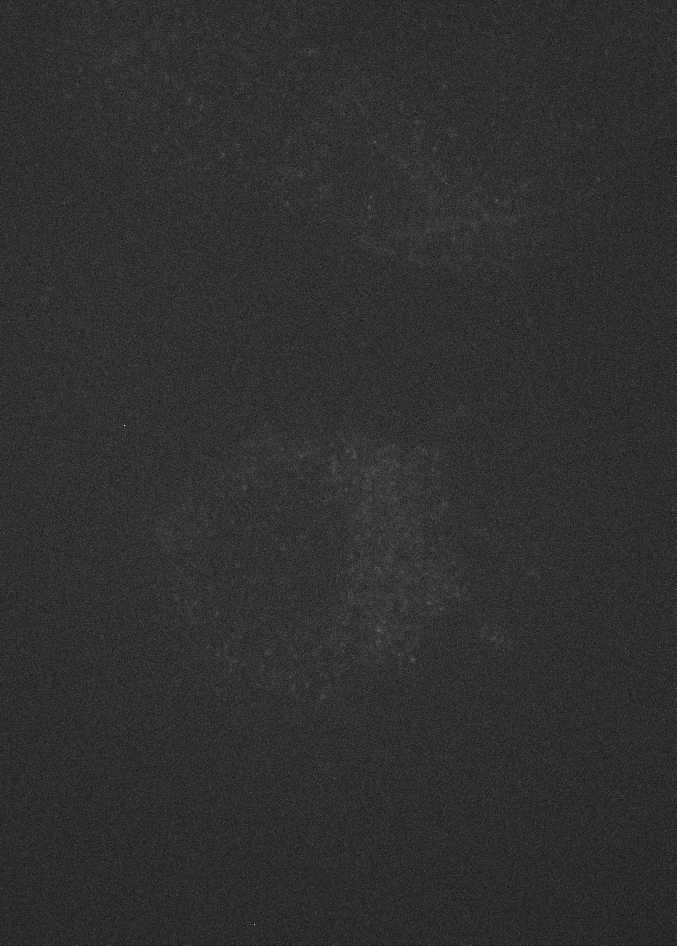

Supplement: Supplementary file 20 — Figure EV2 Source Data [file 44318_2026_705_MOESM20_ESM.zip › Figure EV2/B/20250830_MCF7msER_TMEMGFP_CHIR_5_w3SPI 635 CY5 .TIF]

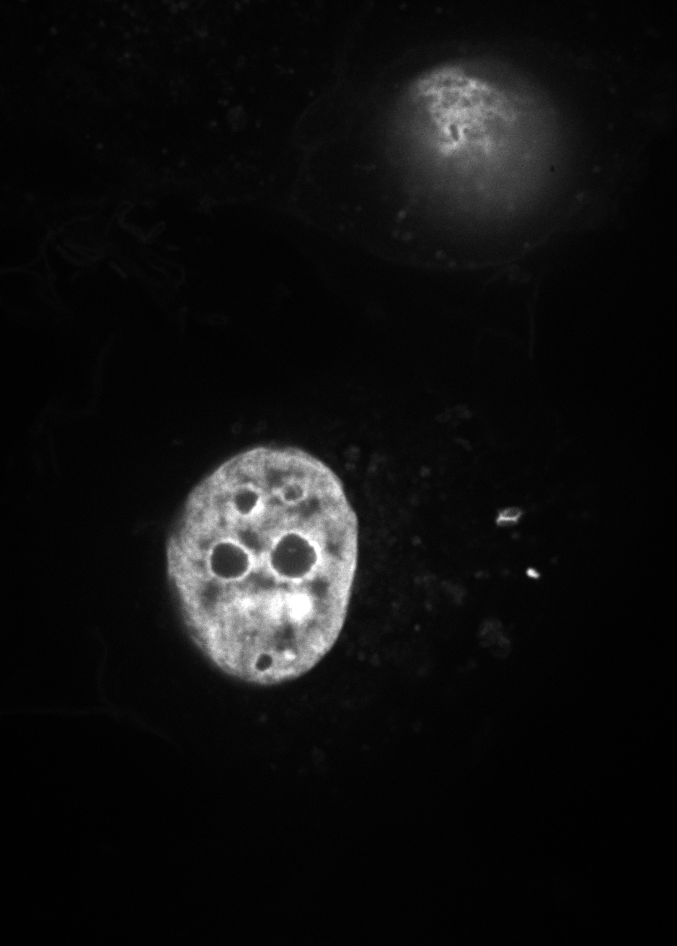

Supplement: Supplementary file 20 — Figure EV2 Source Data [file 44318_2026_705_MOESM20_ESM.zip › Figure EV2/B/20250830_MCF7msER_TMEMGFP_CHIR_5_w4SPI 405 DAPI.TIF]

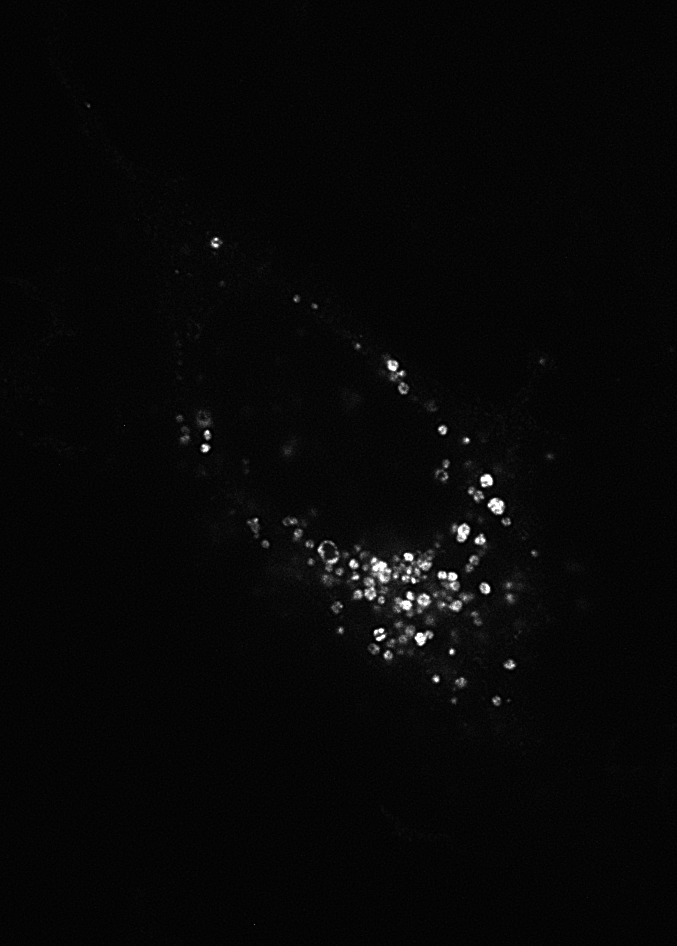

Supplement: Supplementary file 20 — Figure EV2 Source Data [file 44318_2026_705_MOESM20_ESM.zip › Figure EV2/C/20250829_MCF7msER_TMEMGFP_STARD3dr_NT_4_SR_w1SPI 491 GFP.TIF]

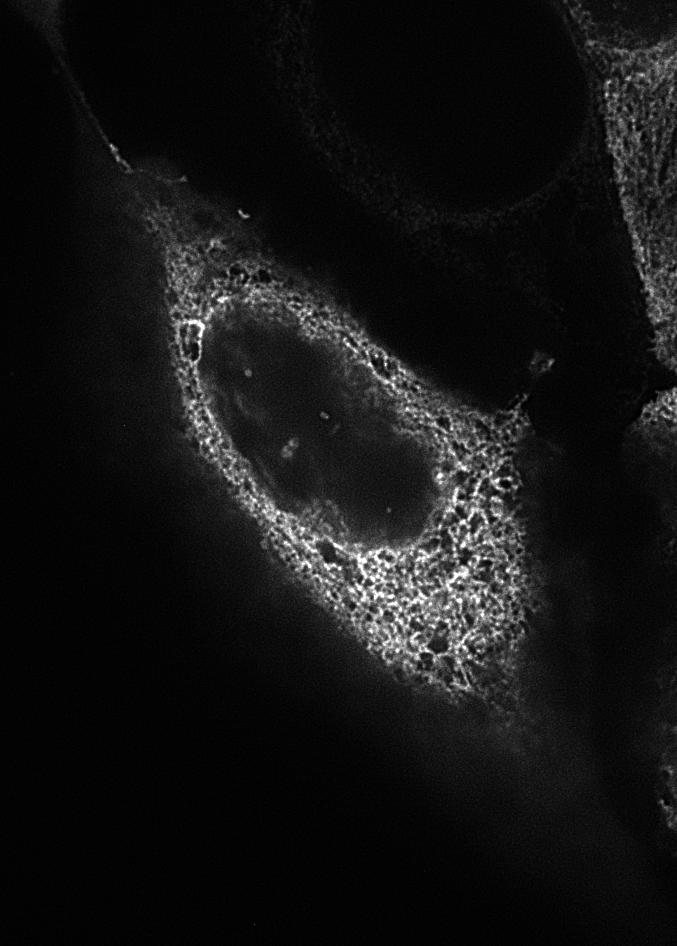

Supplement: Supplementary file 20 — Figure EV2 Source Data [file 44318_2026_705_MOESM20_ESM.zip › Figure EV2/C/20250829_MCF7msER_TMEMGFP_STARD3dr_NT_4_SR_w2SPI 561 mCherry.TIF]

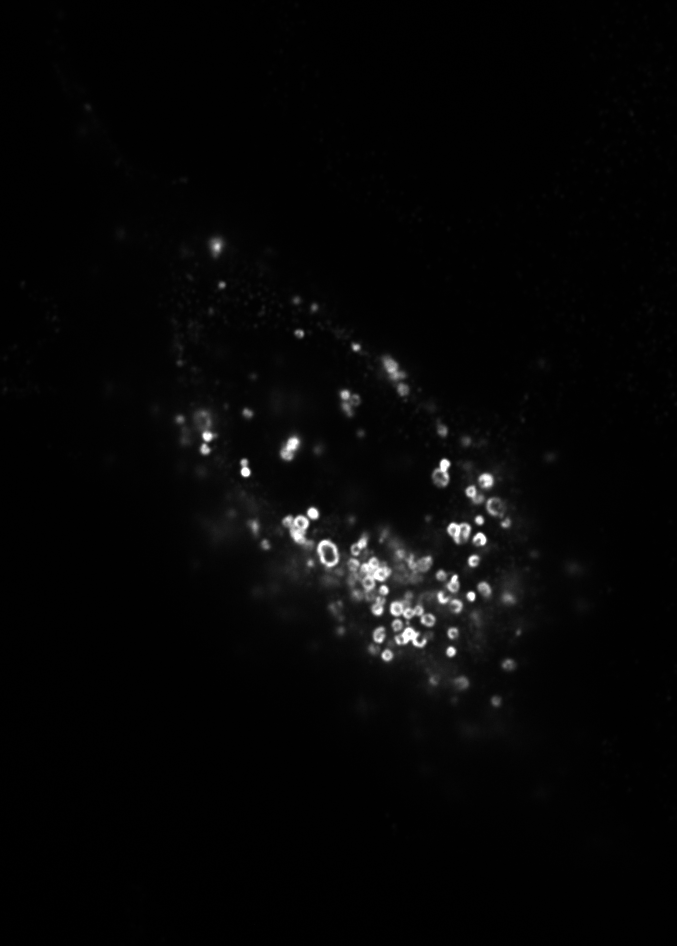

Supplement: Supplementary file 20 — Figure EV2 Source Data [file 44318_2026_705_MOESM20_ESM.zip › Figure EV2/C/20250829_MCF7msER_TMEMGFP_STARD3dr_NT_4_SR_w3SPI 635 CY5 .TIF]

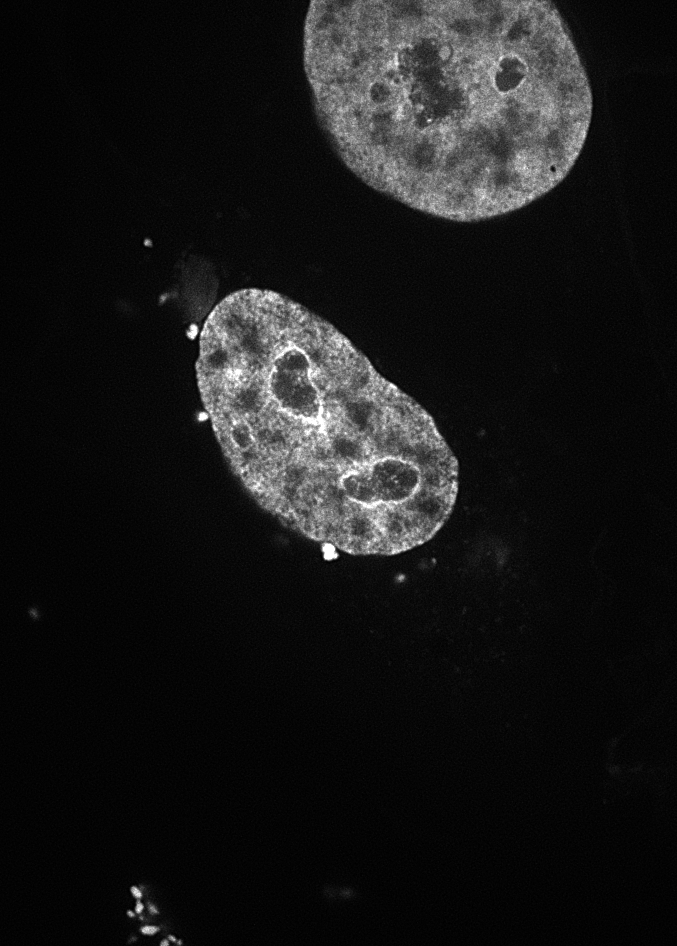

Supplement: Supplementary file 20 — Figure EV2 Source Data [file 44318_2026_705_MOESM20_ESM.zip › Figure EV2/C/20250829_MCF7msER_TMEMGFP_STARD3dr_NT_4_SR_w4SPI 405 DAPI.TIF]

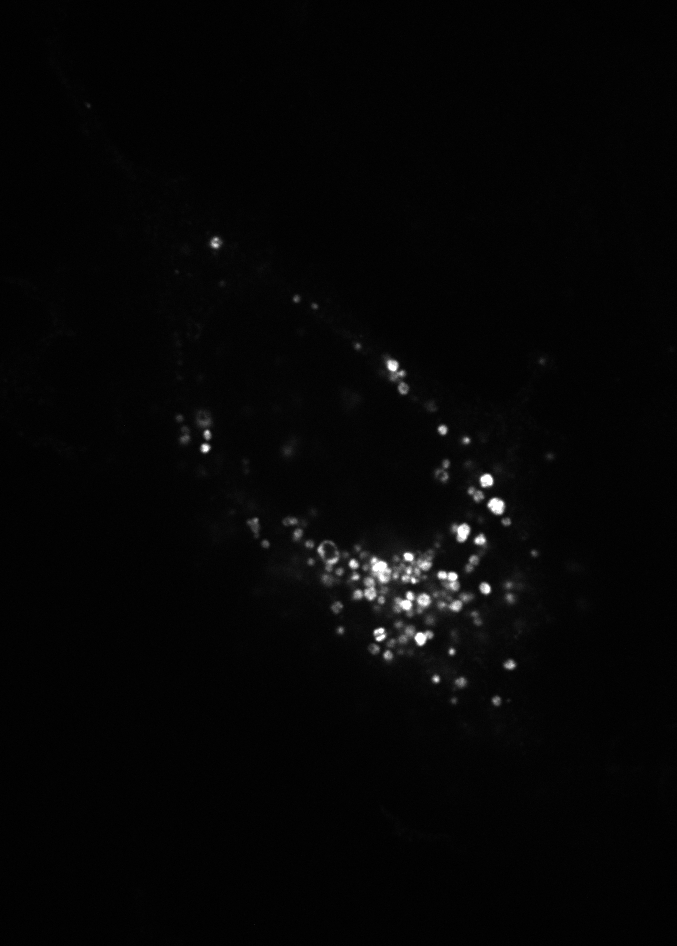

Supplement: Supplementary file 20 — Figure EV2 Source Data [file 44318_2026_705_MOESM20_ESM.zip › Figure EV2/C/20250829_MCF7msER_TMEMGFP_STARD3dr_NT_4_w1SPI 491 GFP.TIF]

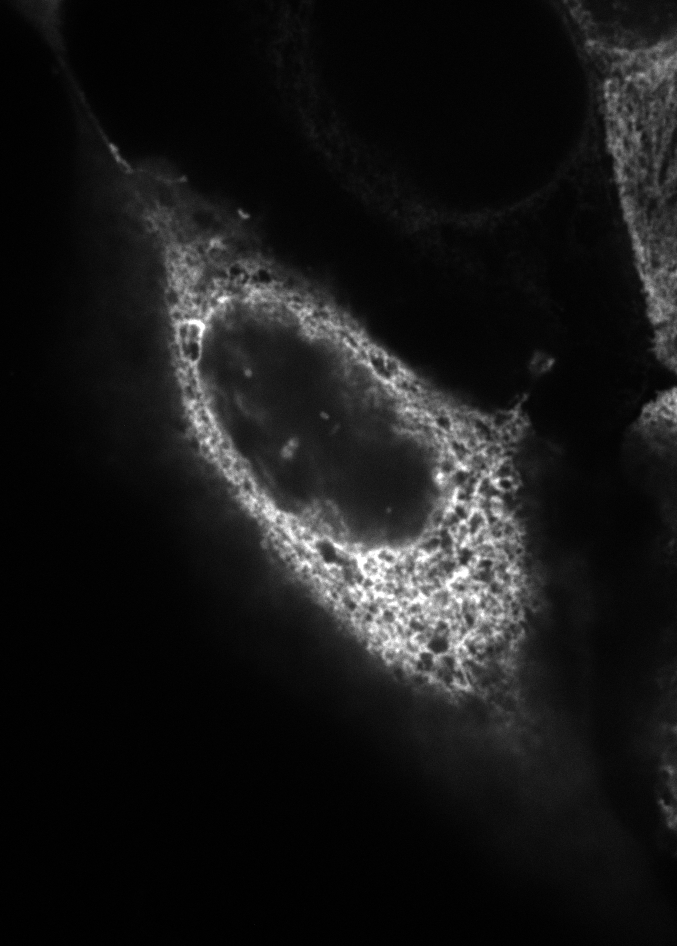

Supplement: Supplementary file 20 — Figure EV2 Source Data [file 44318_2026_705_MOESM20_ESM.zip › Figure EV2/C/20250829_MCF7msER_TMEMGFP_STARD3dr_NT_4_w2SPI 561 mCherry.TIF]

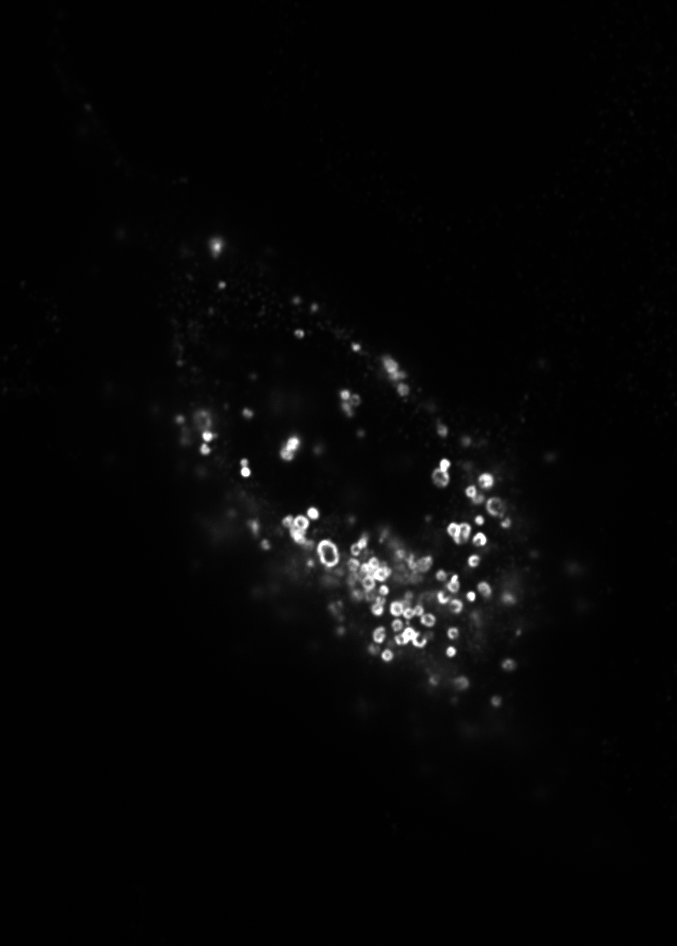

Supplement: Supplementary file 20 — Figure EV2 Source Data [file 44318_2026_705_MOESM20_ESM.zip › Figure EV2/C/20250829_MCF7msER_TMEMGFP_STARD3dr_NT_4_w3SPI 635 CY5 .TIF]

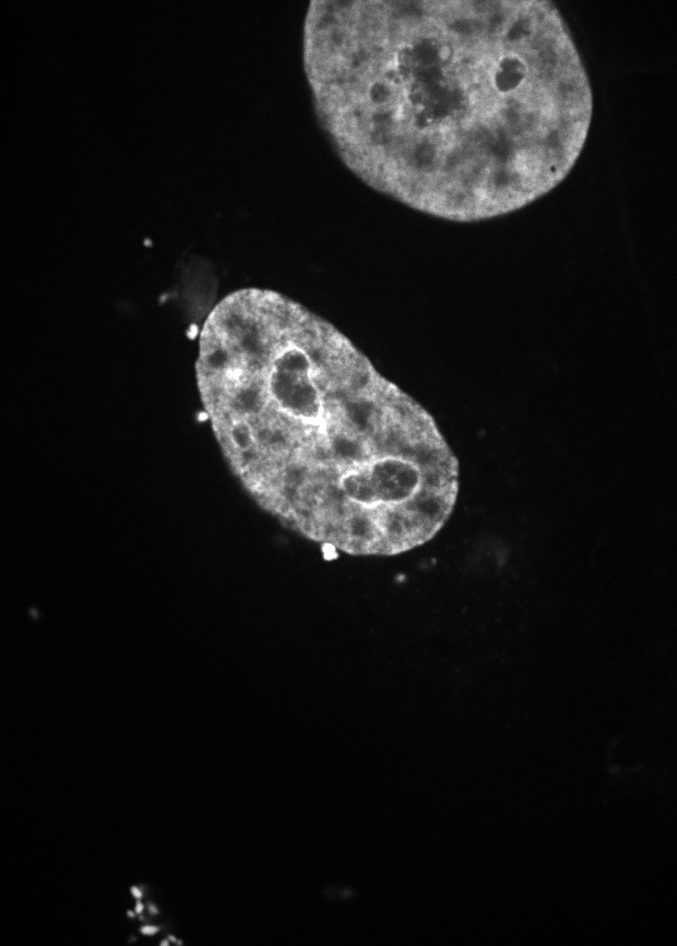

Supplement: Supplementary file 20 — Figure EV2 Source Data [file 44318_2026_705_MOESM20_ESM.zip › Figure EV2/C/20250829_MCF7msER_TMEMGFP_STARD3dr_NT_4_w4SPI 405 DAPI.TIF]

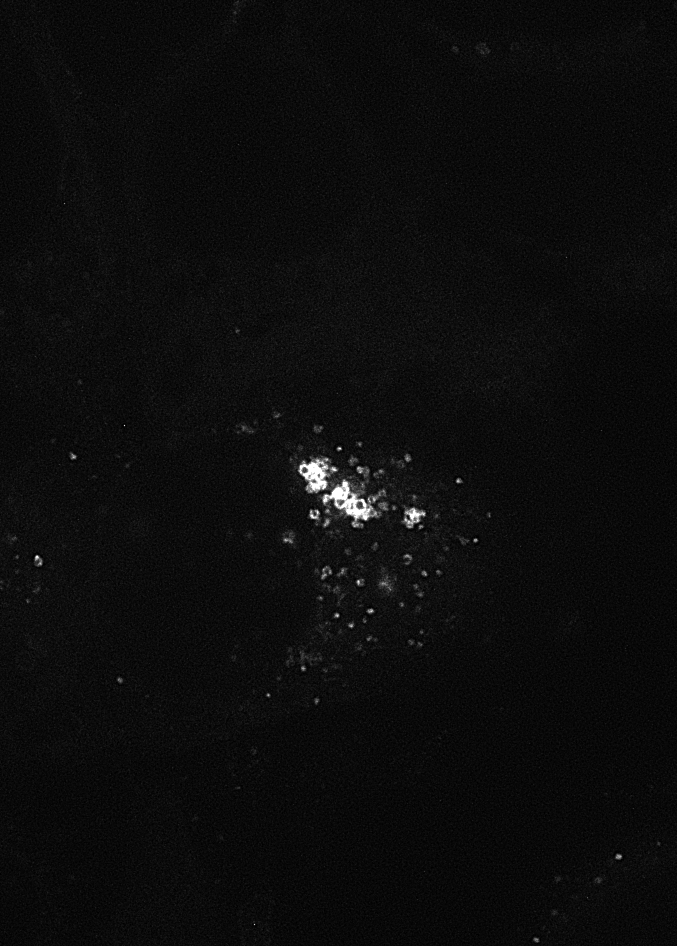

Supplement: Supplementary file 20 — Figure EV2 Source Data [file 44318_2026_705_MOESM20_ESM.zip › Figure EV2/D/20250828_MCF7msER_TMEMGFP_STARD3dr_CHIR_6_SR_w1SPI 491 GFP.TIF]

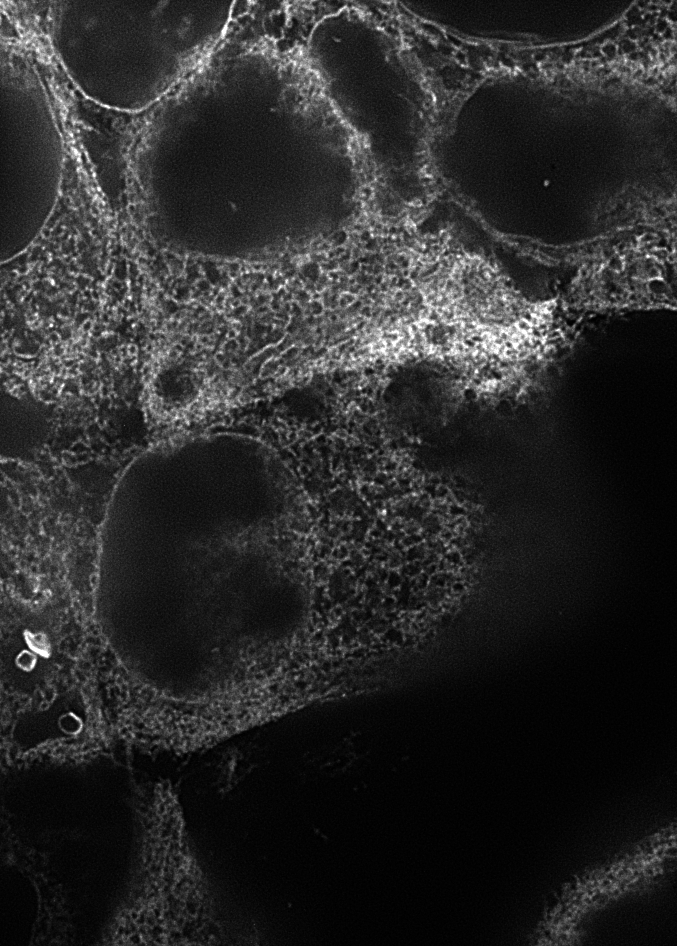

Supplement: Supplementary file 20 — Figure EV2 Source Data [file 44318_2026_705_MOESM20_ESM.zip › Figure EV2/D/20250828_MCF7msER_TMEMGFP_STARD3dr_CHIR_6_SR_w2SPI 561 mCherry.TIF]

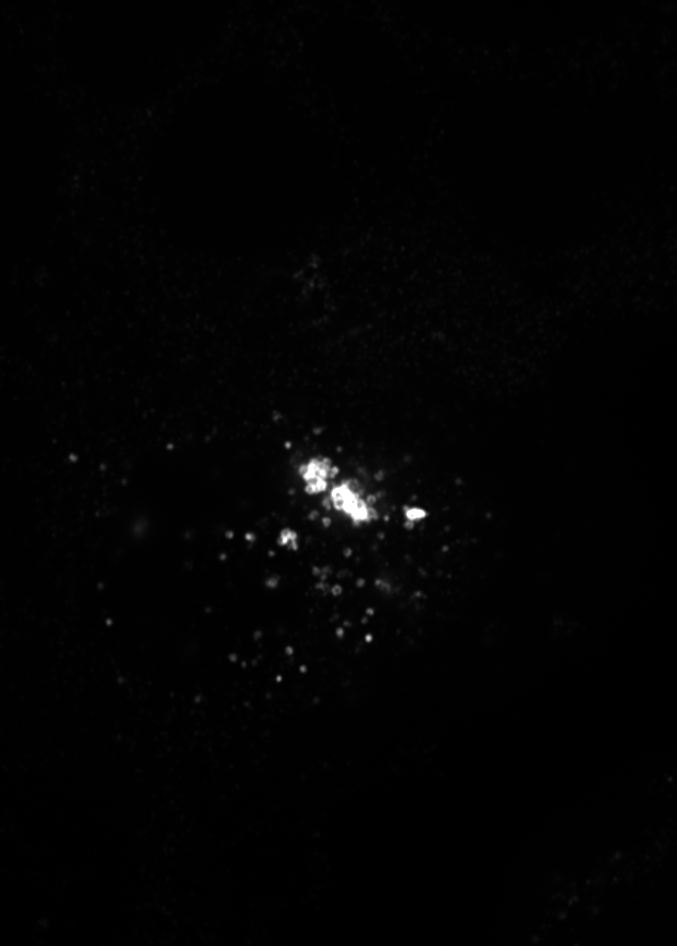

Supplement: Supplementary file 20 — Figure EV2 Source Data [file 44318_2026_705_MOESM20_ESM.zip › Figure EV2/D/20250828_MCF7msER_TMEMGFP_STARD3dr_CHIR_6_SR_w3SPI 635 CY5 .TIF]

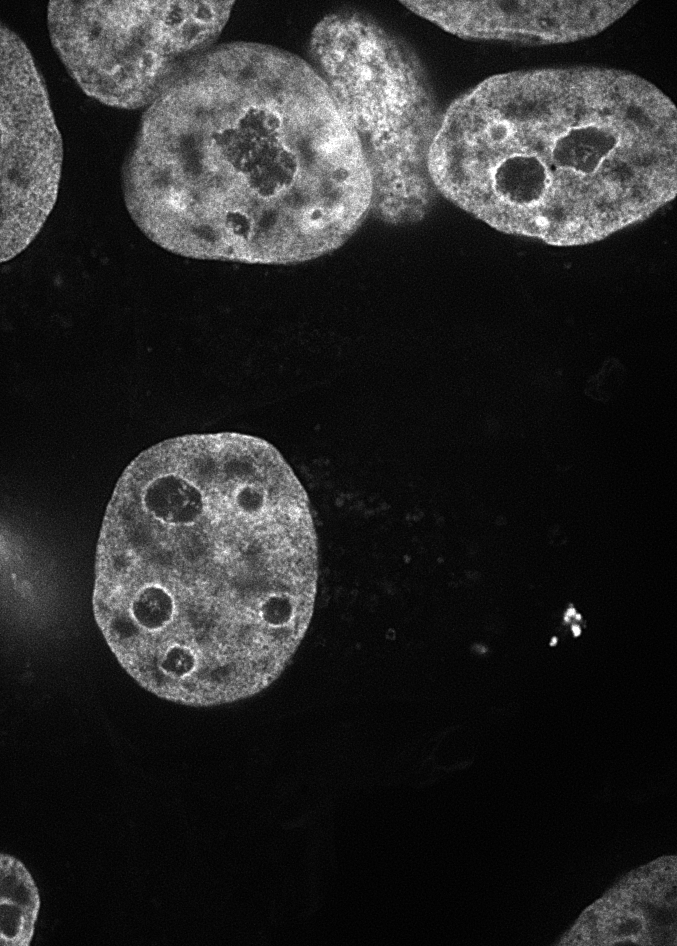

Supplement: Supplementary file 20 — Figure EV2 Source Data [file 44318_2026_705_MOESM20_ESM.zip › Figure EV2/D/20250828_MCF7msER_TMEMGFP_STARD3dr_CHIR_6_SR_w4SPI 405 DAPI.TIF]

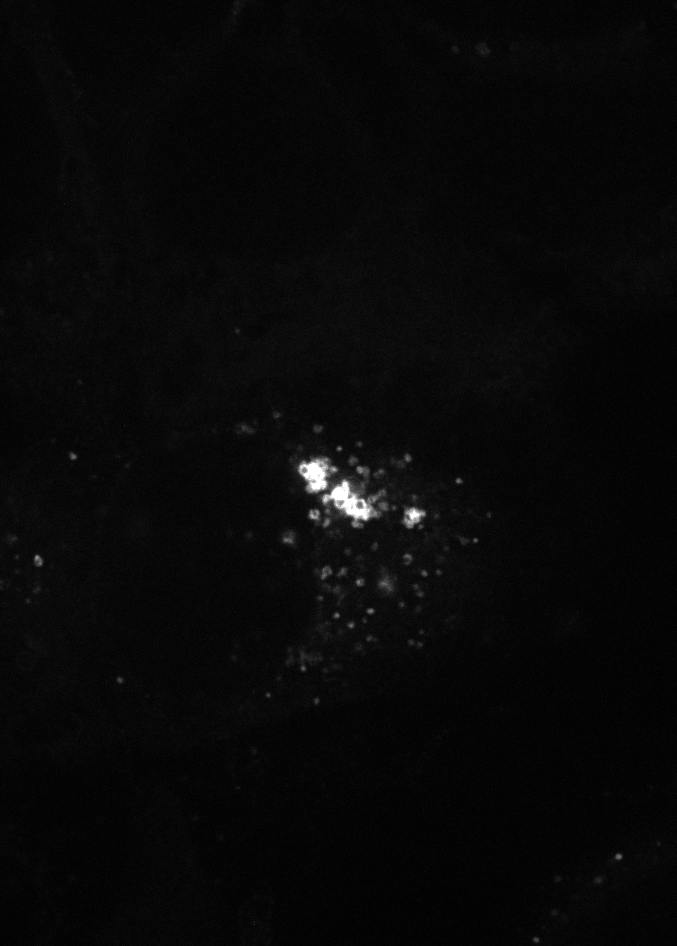

Supplement: Supplementary file 20 — Figure EV2 Source Data [file 44318_2026_705_MOESM20_ESM.zip › Figure EV2/D/20250828_MCF7msER_TMEMGFP_STARD3dr_CHIR_6_w1SPI 491 GFP.TIF]

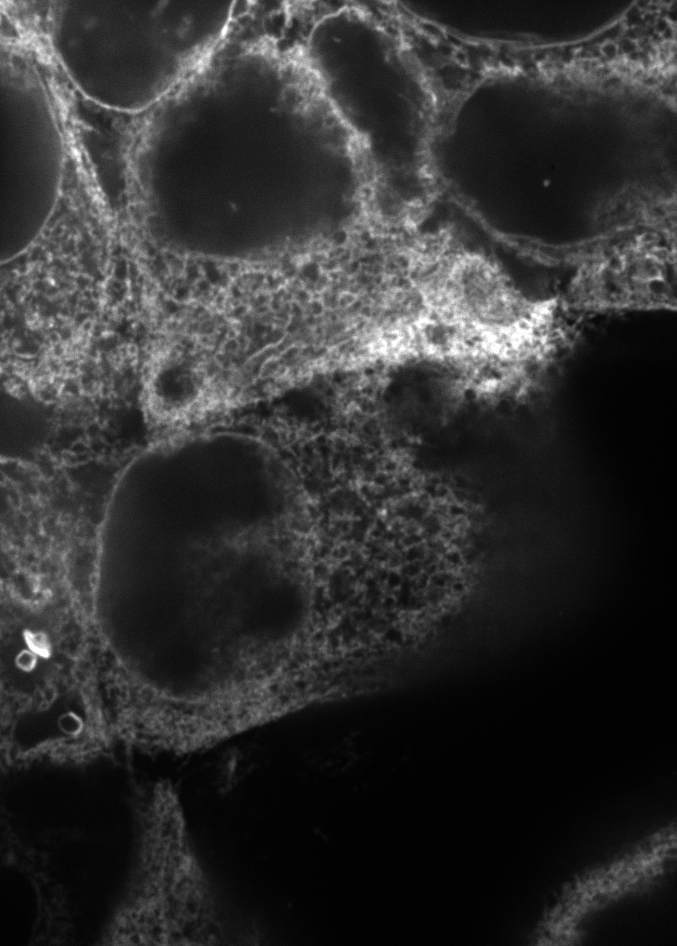

Supplement: Supplementary file 20 — Figure EV2 Source Data [file 44318_2026_705_MOESM20_ESM.zip › Figure EV2/D/20250828_MCF7msER_TMEMGFP_STARD3dr_CHIR_6_w2SPI 561 mCherry.TIF]

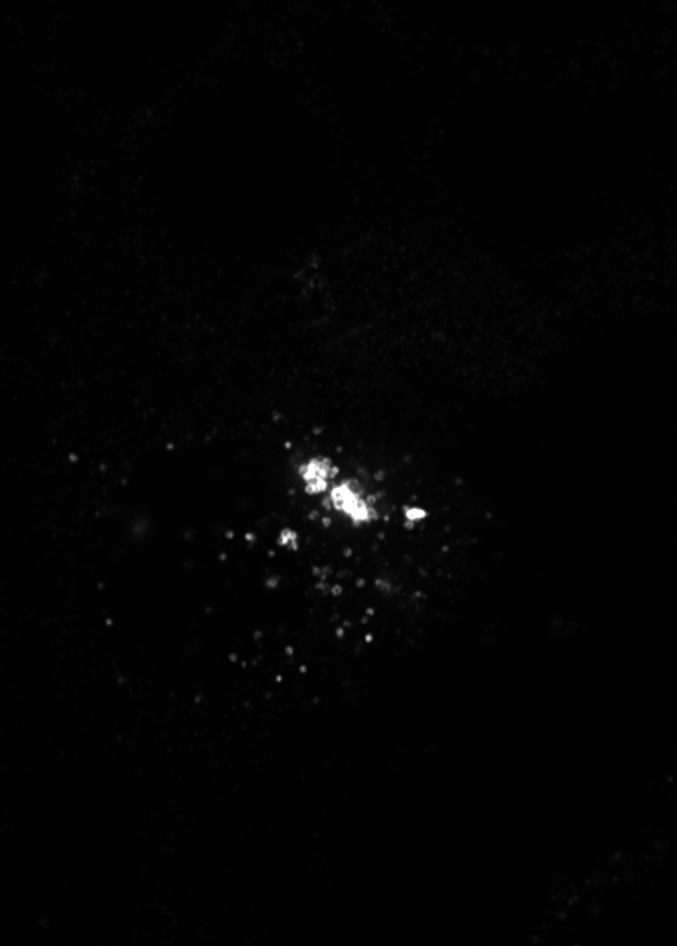

Supplement: Supplementary file 20 — Figure EV2 Source Data [file 44318_2026_705_MOESM20_ESM.zip › Figure EV2/D/20250828_MCF7msER_TMEMGFP_STARD3dr_CHIR_6_w3SPI 635 CY5 .TIF]

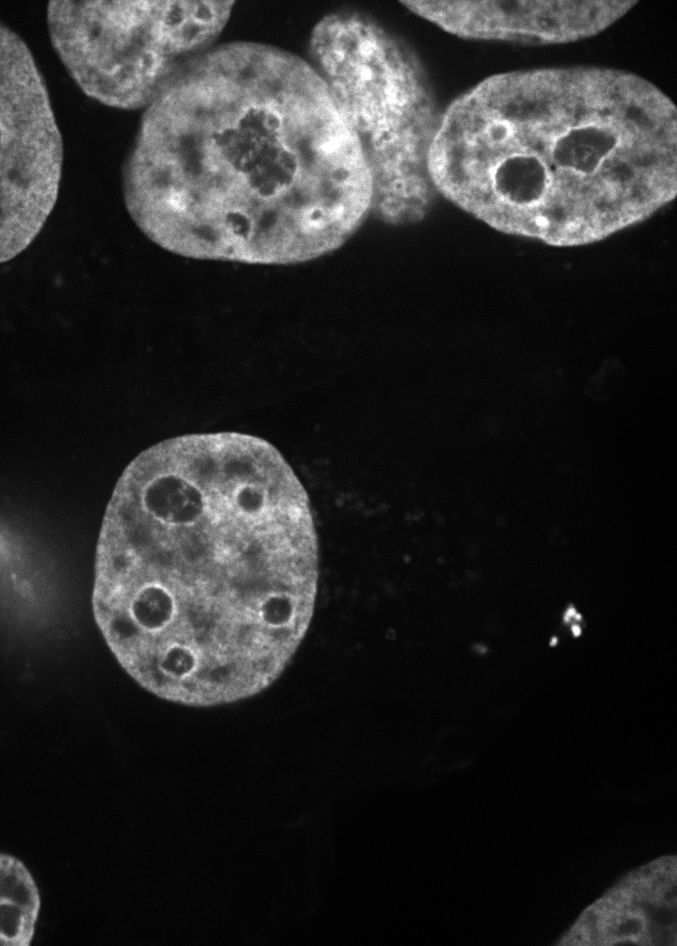

Supplement: Supplementary file 20 — Figure EV2 Source Data [file 44318_2026_705_MOESM20_ESM.zip › Figure EV2/D/20250828_MCF7msER_TMEMGFP_STARD3dr_CHIR_6_w4SPI 405 DAPI.TIF]

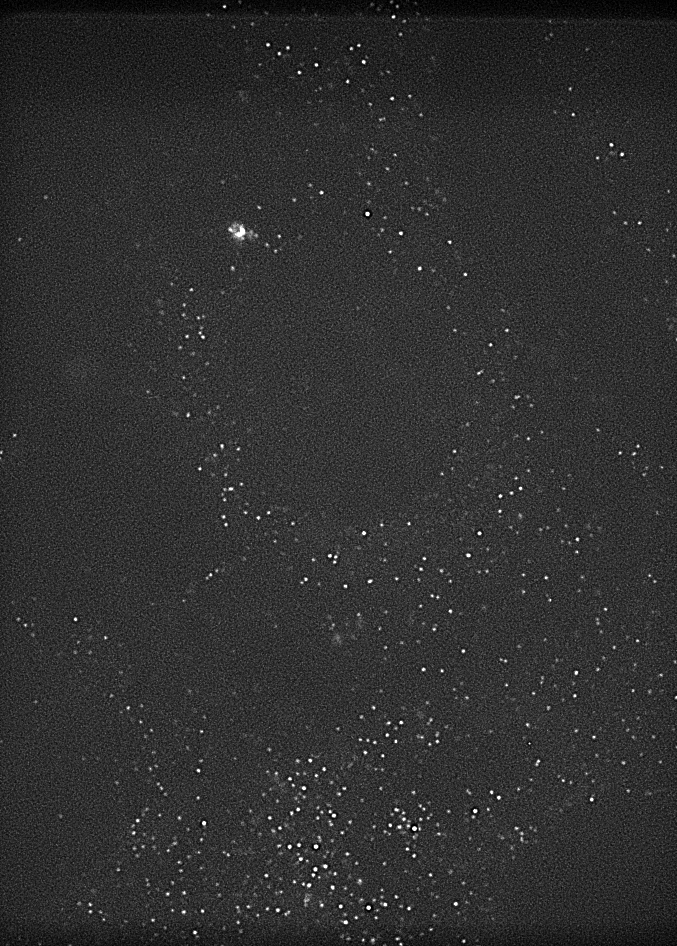

Supplement: Supplementary file 21 — Figure EV3 Source Data [file 44318_2026_705_MOESM21_ESM.zip › Figure EV3-1/A/MCF7WT_LAMP1_CHIR99021/20221014_MCF7ctrl_GSK3i_5_SR_w1SPI 491 GFP.TIF]

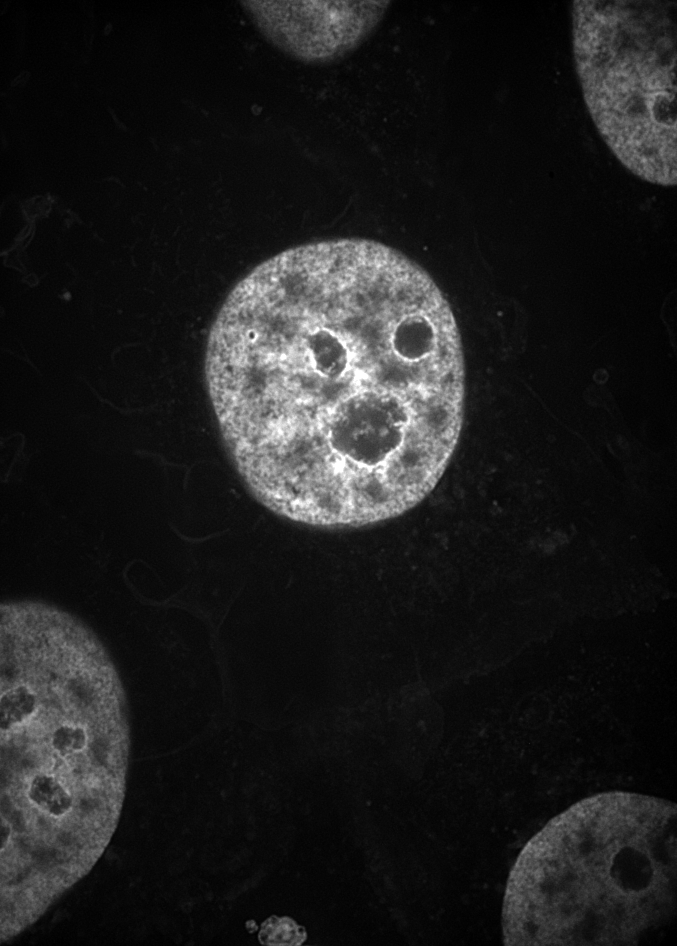

Supplement: Supplementary file 21 — Figure EV3 Source Data [file 44318_2026_705_MOESM21_ESM.zip › Figure EV3-1/A/MCF7WT_LAMP1_CHIR99021/20221014_MCF7ctrl_GSK3i_5_SR_w2SPI 405 DAPI.TIF]

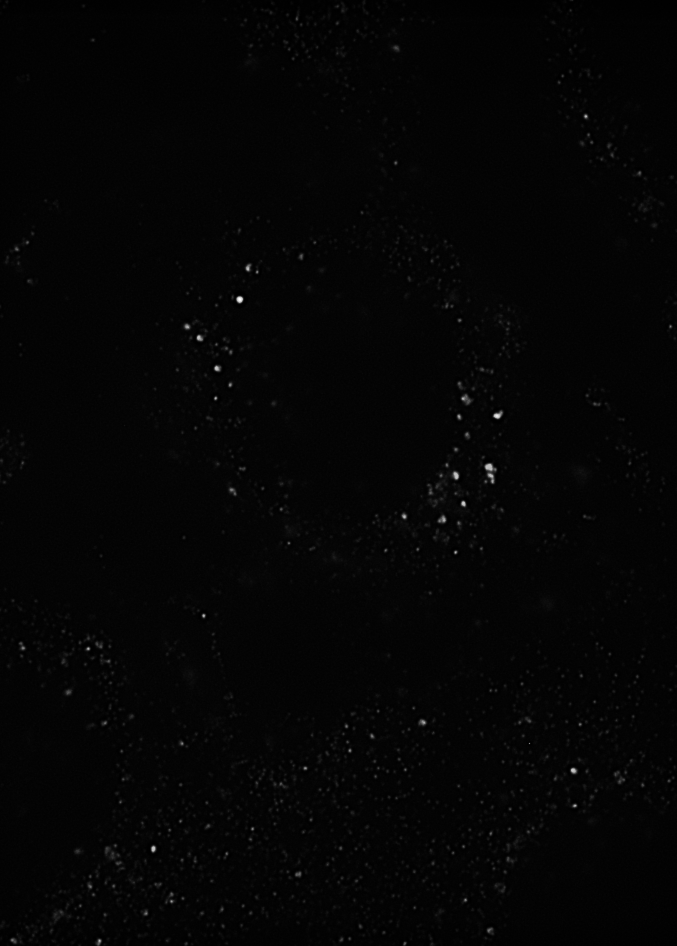

Supplement: Supplementary file 21 — Figure EV3 Source Data [file 44318_2026_705_MOESM21_ESM.zip › Figure EV3-1/A/MCF7WT_LAMP1_CHIR99021/20221014_MCF7ctrl_GSK3i_5_SR_w3SPI 561 mCherry.TIF]

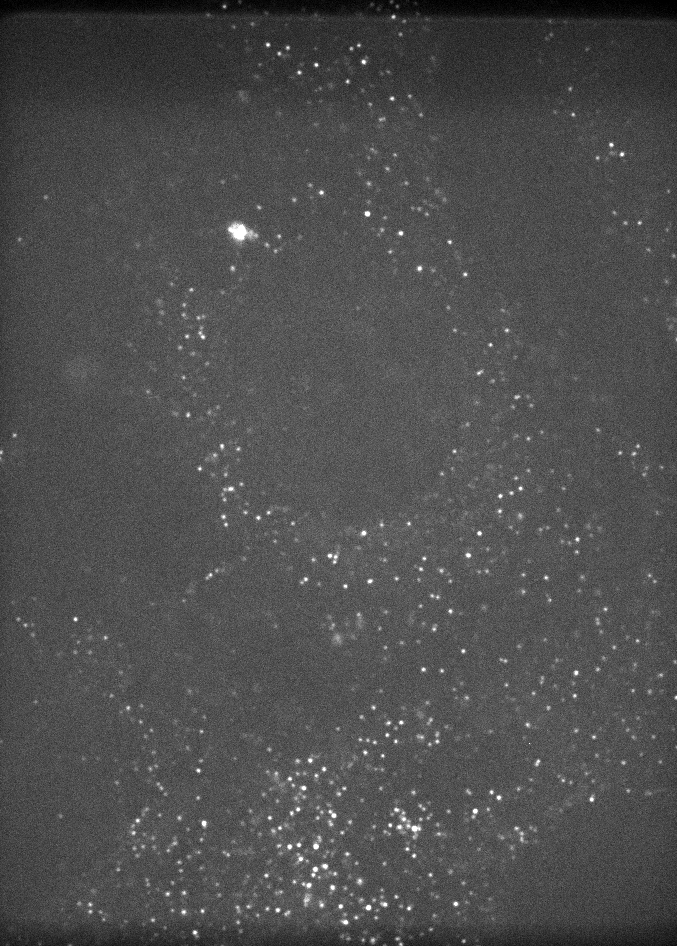

Supplement: Supplementary file 21 — Figure EV3 Source Data [file 44318_2026_705_MOESM21_ESM.zip › Figure EV3-1/A/MCF7WT_LAMP1_CHIR99021/20221014_MCF7ctrl_GSK3i_5_w1SPI 491 GFP.TIF]

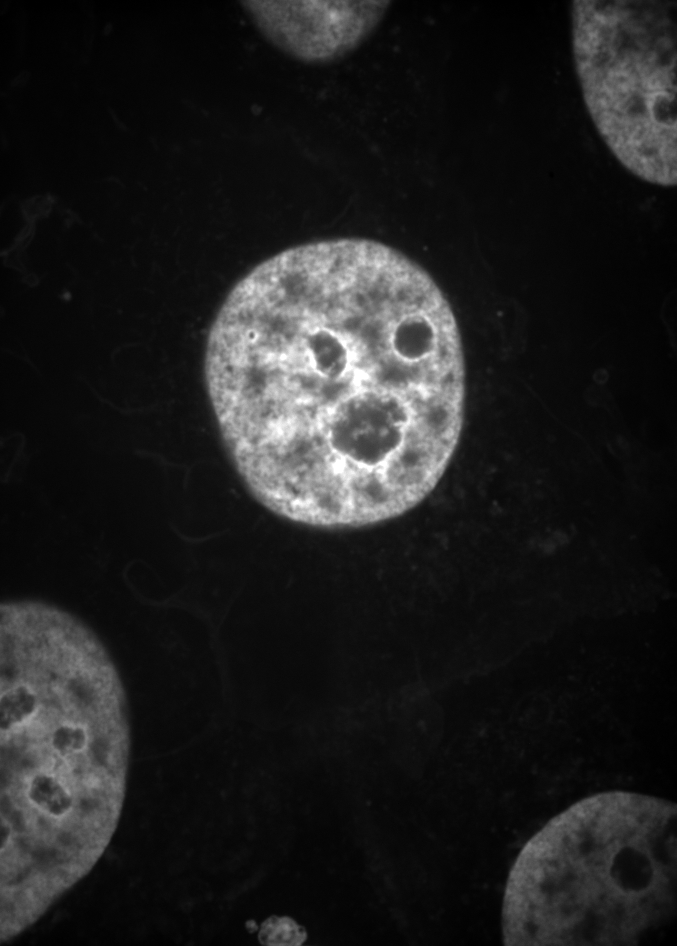

Supplement: Supplementary file 21 — Figure EV3 Source Data [file 44318_2026_705_MOESM21_ESM.zip › Figure EV3-1/A/MCF7WT_LAMP1_CHIR99021/20221014_MCF7ctrl_GSK3i_5_w2SPI 405 DAPI.TIF]

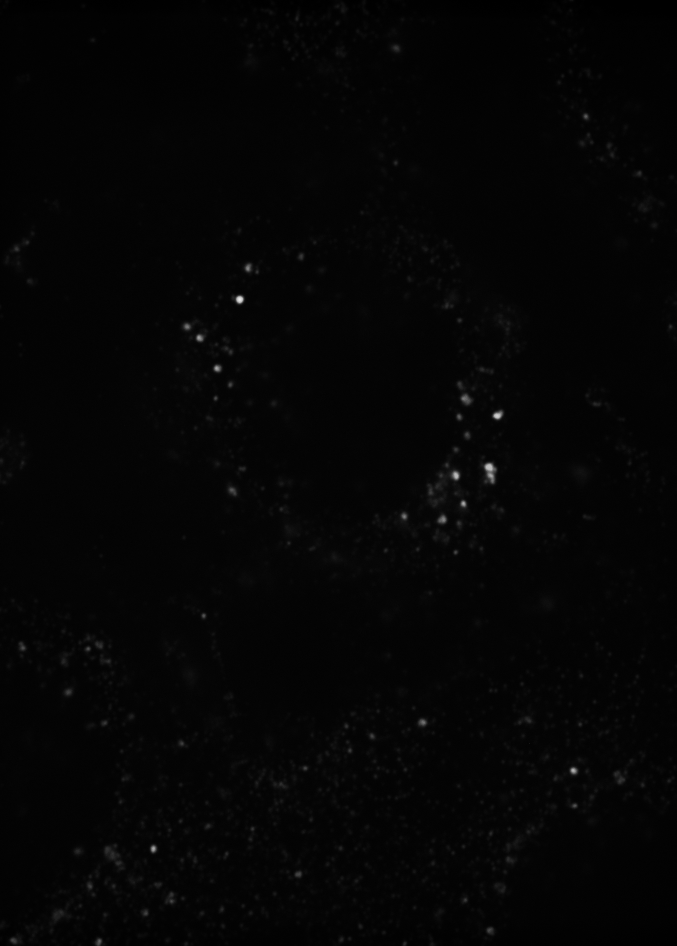

Supplement: Supplementary file 21 — Figure EV3 Source Data [file 44318_2026_705_MOESM21_ESM.zip › Figure EV3-1/A/MCF7WT_LAMP1_CHIR99021/20221014_MCF7ctrl_GSK3i_5_w3SPI 561 mCherry.TIF]

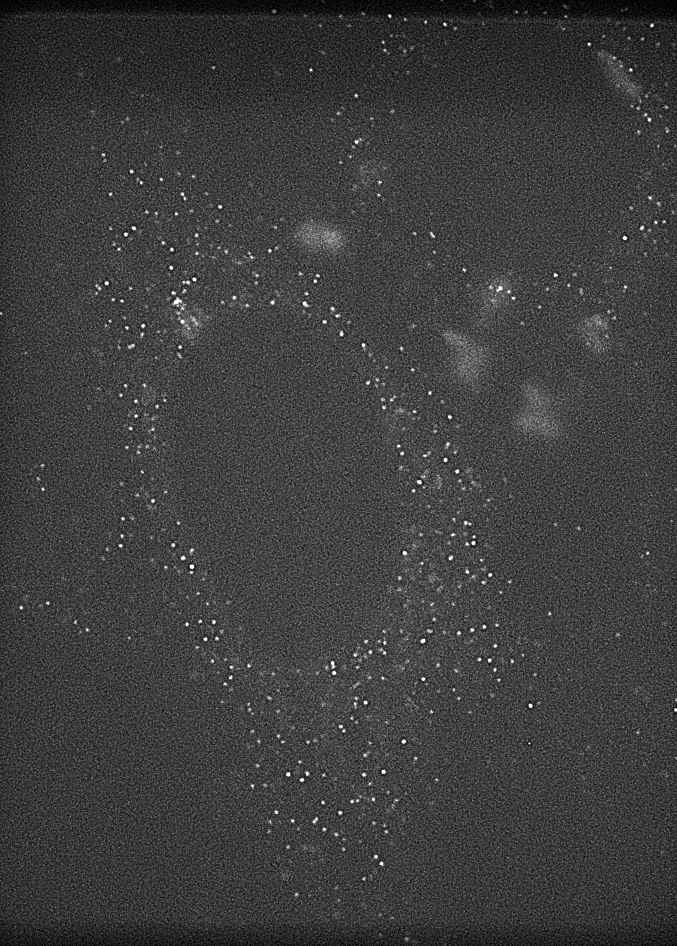

Supplement: Supplementary file 21 — Figure EV3 Source Data [file 44318_2026_705_MOESM21_ESM.zip › Figure EV3-1/A/MCF7WT_LAMP1_NT/20221014_MCF7ctrl_NT_1_SR_w1SPI 491 GFP.TIF]

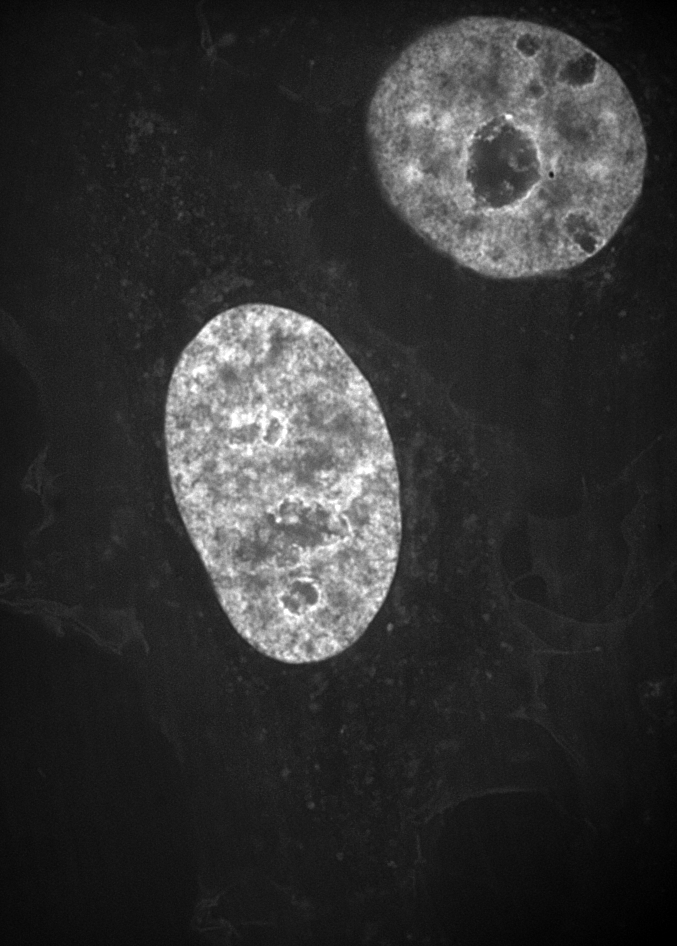

Supplement: Supplementary file 21 — Figure EV3 Source Data [file 44318_2026_705_MOESM21_ESM.zip › Figure EV3-1/A/MCF7WT_LAMP1_NT/20221014_MCF7ctrl_NT_1_SR_w2SPI 405 DAPI.TIF]

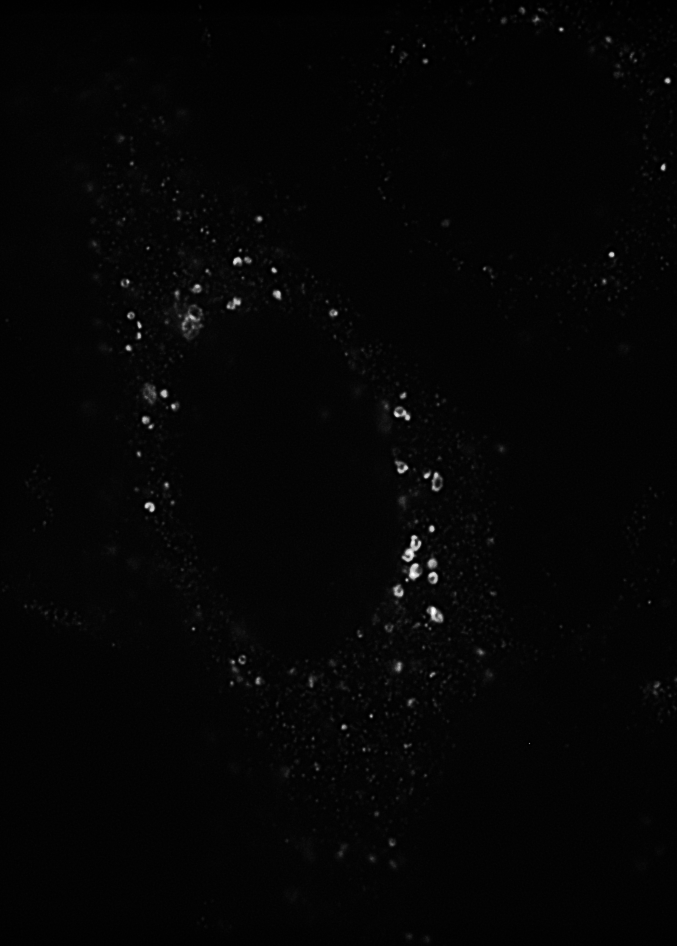

Supplement: Supplementary file 21 — Figure EV3 Source Data [file 44318_2026_705_MOESM21_ESM.zip › Figure EV3-1/A/MCF7WT_LAMP1_NT/20221014_MCF7ctrl_NT_1_SR_w3SPI 561 mCherry.TIF]

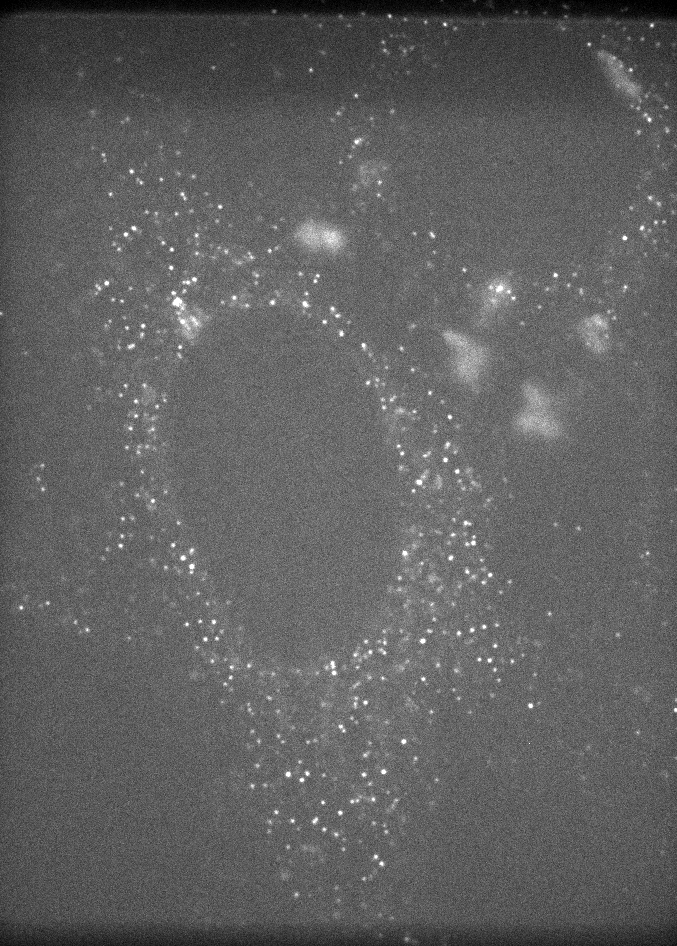

Supplement: Supplementary file 21 — Figure EV3 Source Data [file 44318_2026_705_MOESM21_ESM.zip › Figure EV3-1/A/MCF7WT_LAMP1_NT/20221014_MCF7ctrl_NT_1_w1SPI 491 GFP.TIF]

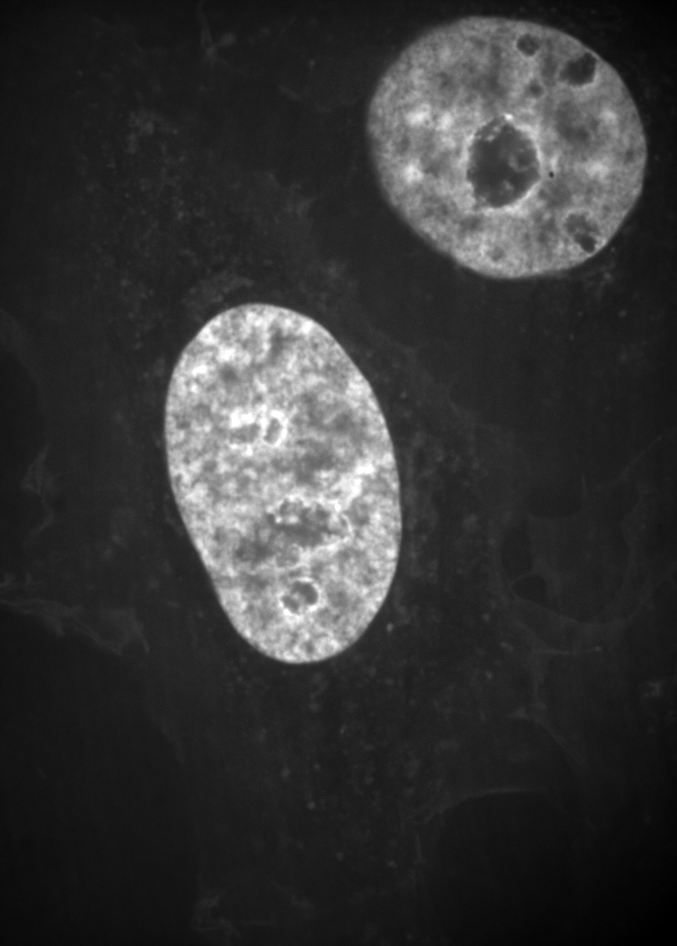

Supplement: Supplementary file 21 — Figure EV3 Source Data [file 44318_2026_705_MOESM21_ESM.zip › Figure EV3-1/A/MCF7WT_LAMP1_NT/20221014_MCF7ctrl_NT_1_w2SPI 405 DAPI.TIF]

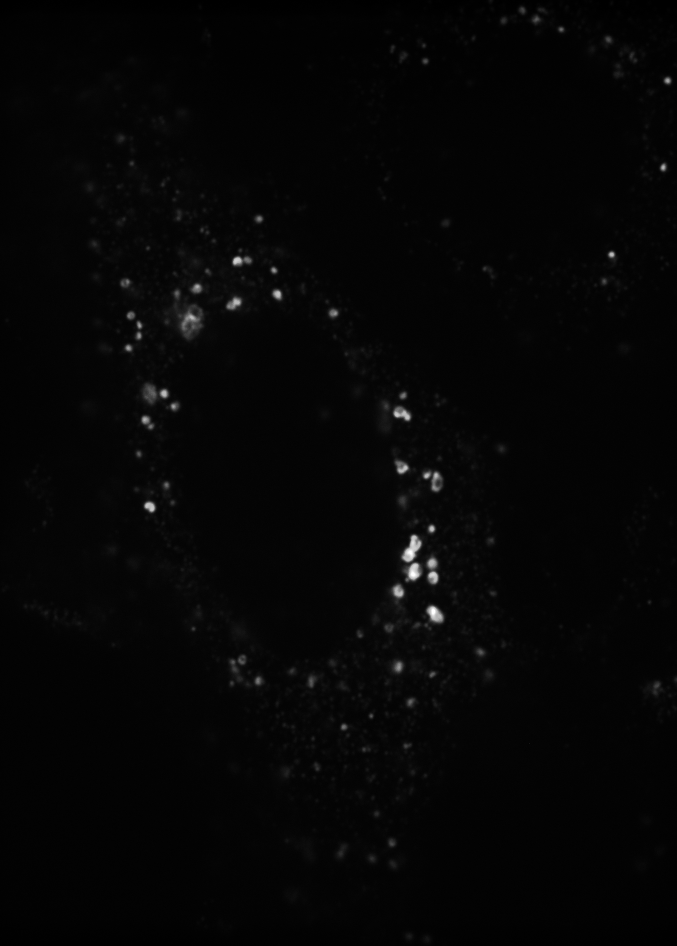

Supplement: Supplementary file 21 — Figure EV3 Source Data [file 44318_2026_705_MOESM21_ESM.zip › Figure EV3-1/A/MCF7WT_LAMP1_NT/20221014_MCF7ctrl_NT_1_w3SPI 561 mCherry.TIF]

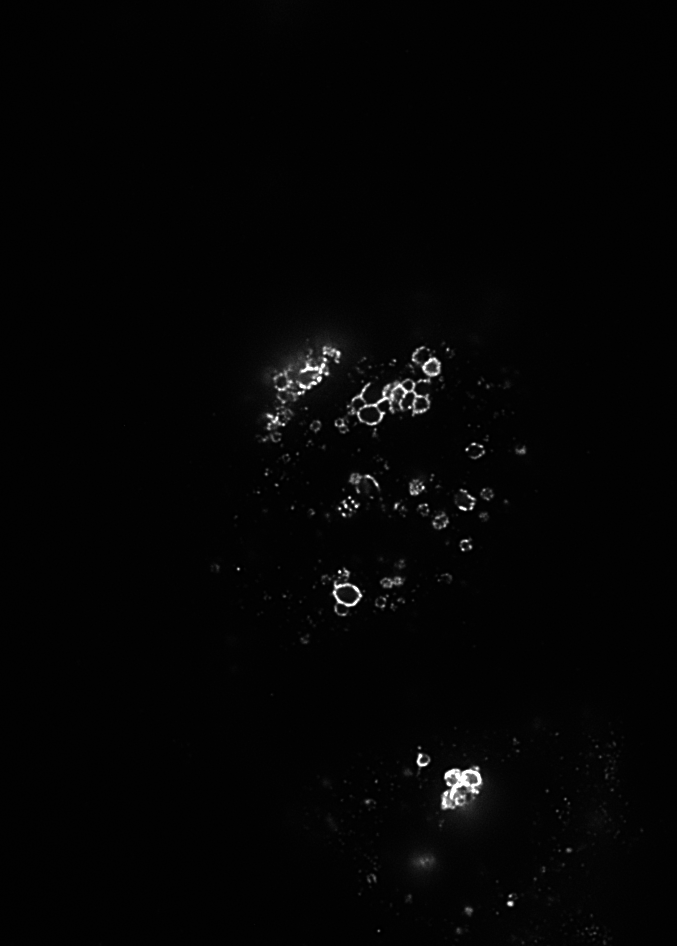

Supplement: Supplementary file 21 — Figure EV3 Source Data [file 44318_2026_705_MOESM21_ESM.zip › Figure EV3-1/B/STARD3_LAMP1_CHIR99021/20250127_MCF7STARD3WT_CHIR_10_SR_w1SPI 491 GFP.TIF]

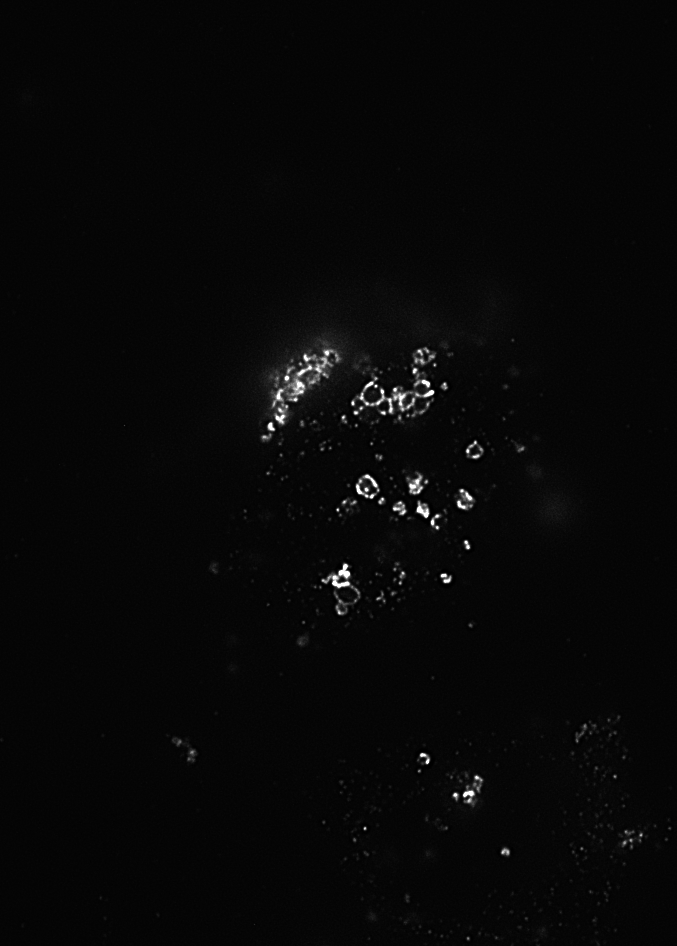

Supplement: Supplementary file 21 — Figure EV3 Source Data [file 44318_2026_705_MOESM21_ESM.zip › Figure EV3-1/B/STARD3_LAMP1_CHIR99021/20250127_MCF7STARD3WT_CHIR_10_SR_w2SPI 561 mCherry.TIF]

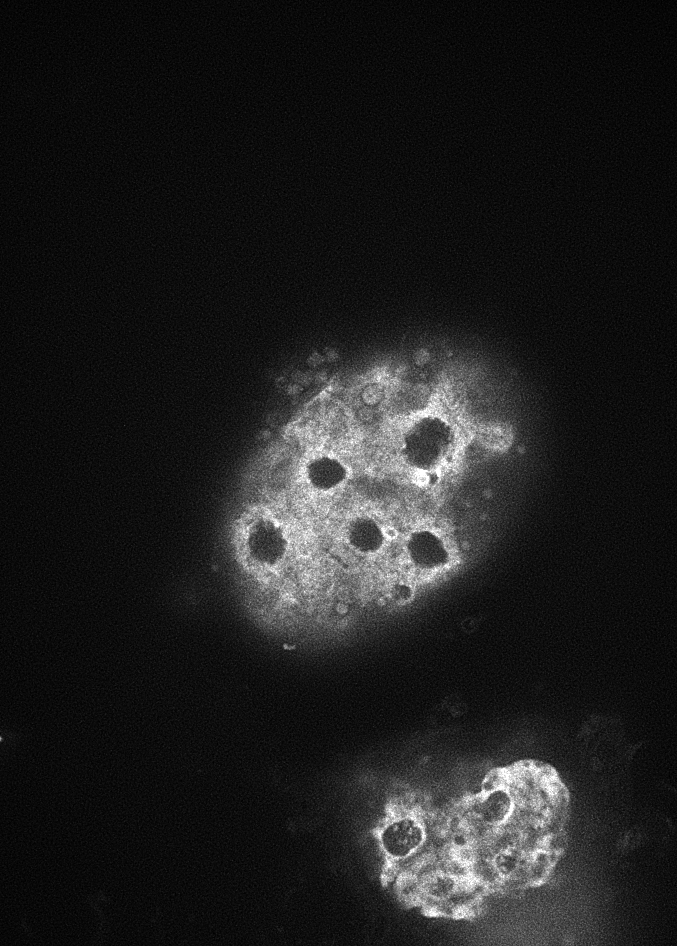

Supplement: Supplementary file 21 — Figure EV3 Source Data [file 44318_2026_705_MOESM21_ESM.zip › Figure EV3-1/B/STARD3_LAMP1_CHIR99021/20250127_MCF7STARD3WT_CHIR_10_SR_w3SPI 405 DAPI.TIF]

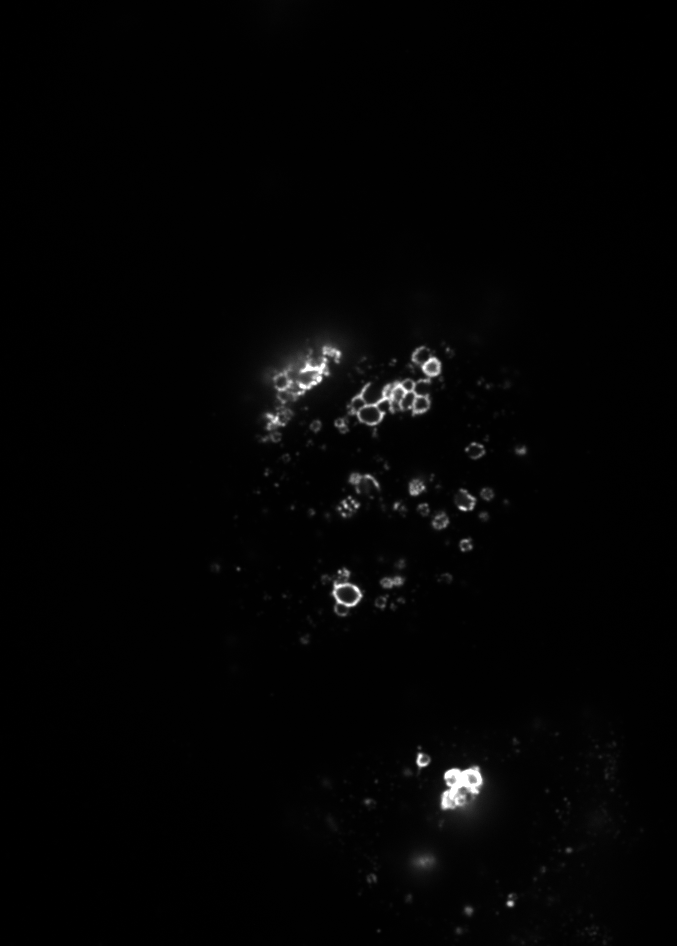

Supplement: Supplementary file 21 — Figure EV3 Source Data [file 44318_2026_705_MOESM21_ESM.zip › Figure EV3-1/B/STARD3_LAMP1_CHIR99021/20250127_MCF7STARD3WT_CHIR_10_w1SPI 491 GFP.TIF]

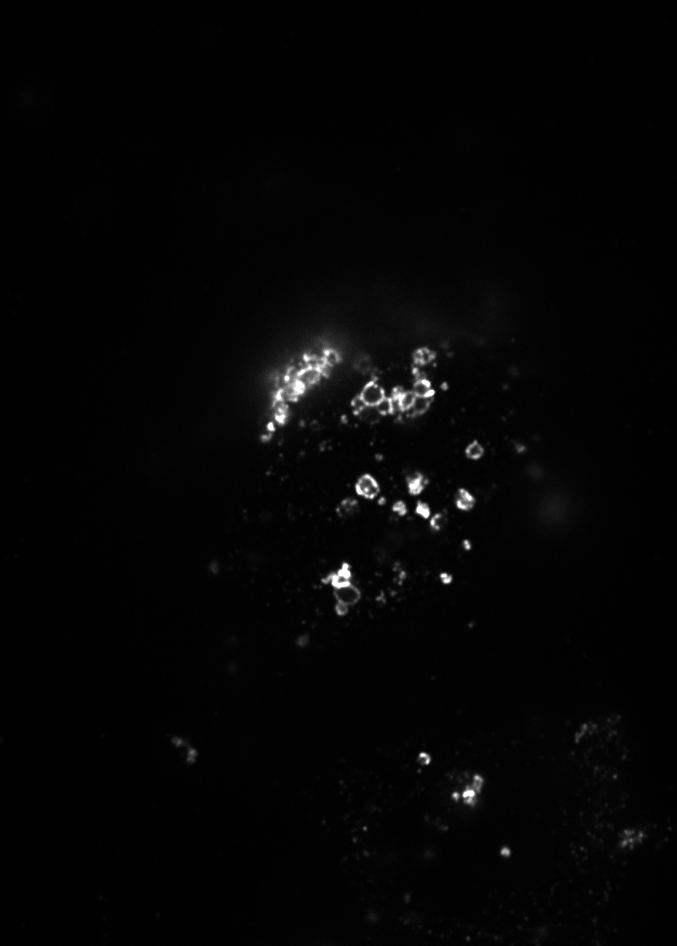

Supplement: Supplementary file 21 — Figure EV3 Source Data [file 44318_2026_705_MOESM21_ESM.zip › Figure EV3-1/B/STARD3_LAMP1_CHIR99021/20250127_MCF7STARD3WT_CHIR_10_w2SPI 561 mCherry.TIF]

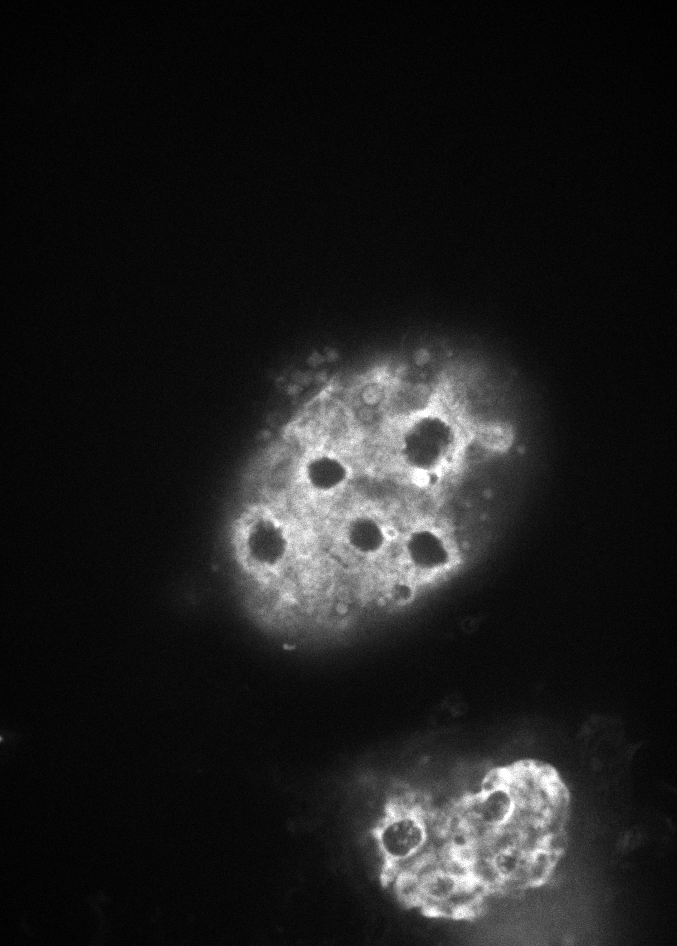

Supplement: Supplementary file 21 — Figure EV3 Source Data [file 44318_2026_705_MOESM21_ESM.zip › Figure EV3-1/B/STARD3_LAMP1_CHIR99021/20250127_MCF7STARD3WT_CHIR_10_w3SPI 405 DAPI.TIF]

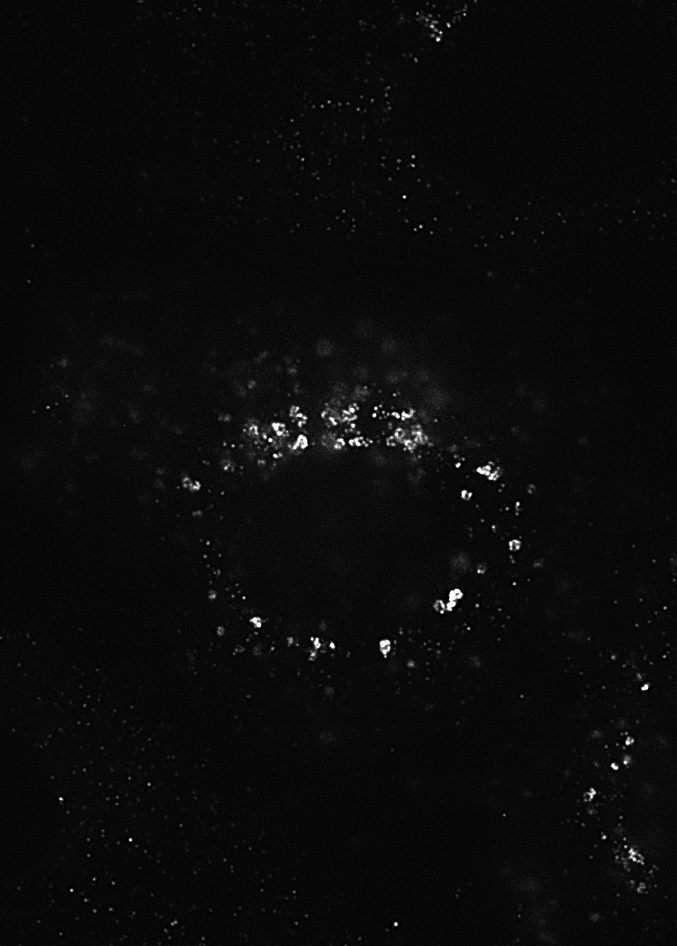

Supplement: Supplementary file 21 — Figure EV3 Source Data [file 44318_2026_705_MOESM21_ESM.zip › Figure EV3-1/B/STARD3_LAMP1_NT/20250120_MCF7STARD3WT_NT2_SR_w1SPI 491 GFP.TIF]

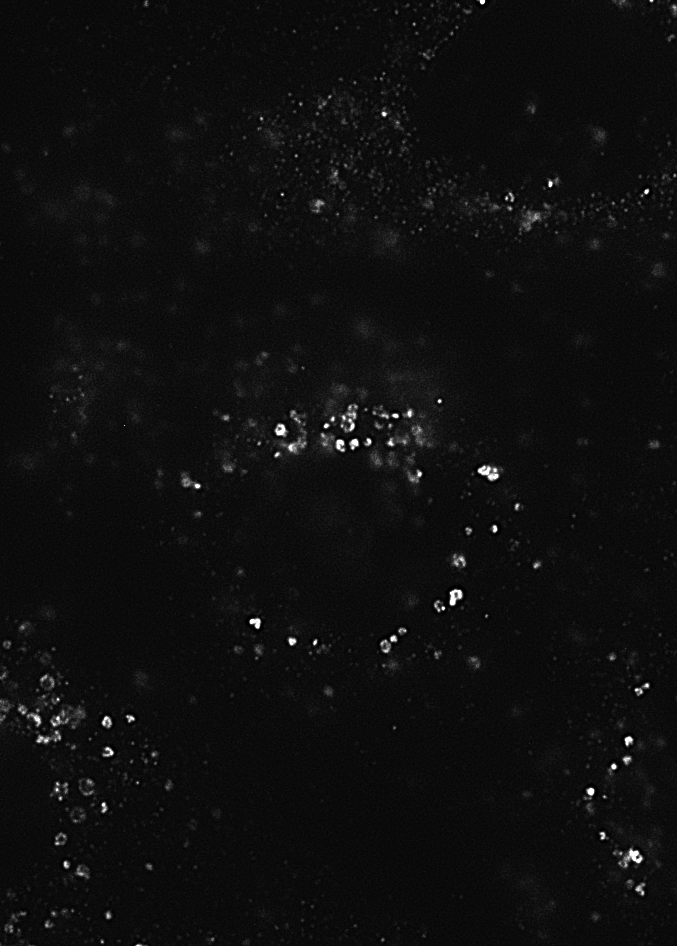

Supplement: Supplementary file 21 — Figure EV3 Source Data [file 44318_2026_705_MOESM21_ESM.zip › Figure EV3-1/B/STARD3_LAMP1_NT/20250120_MCF7STARD3WT_NT2_SR_w2SPI 561 mCherry.TIF]

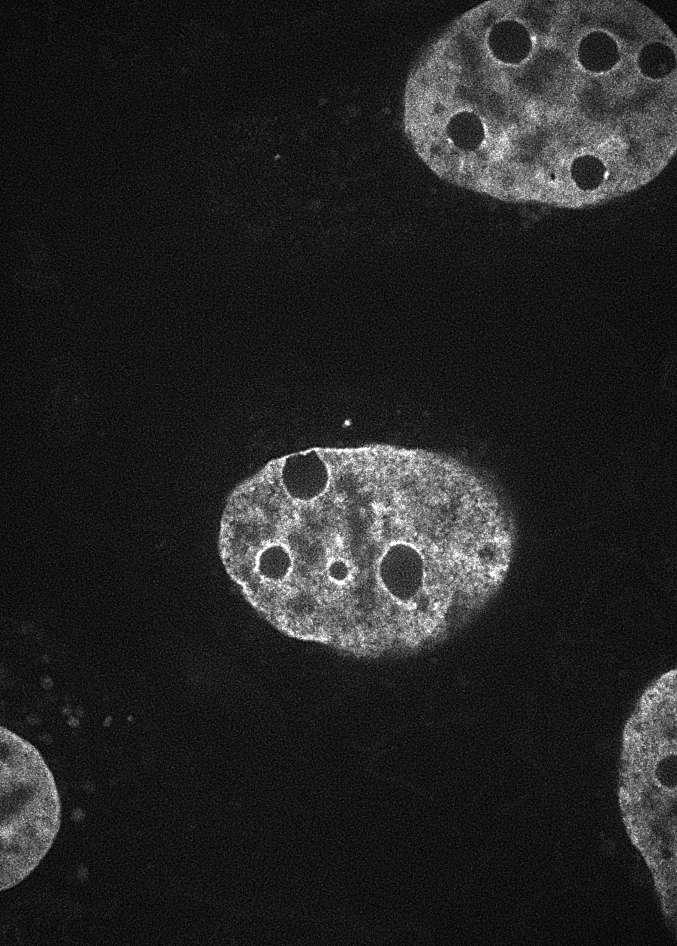

Supplement: Supplementary file 21 — Figure EV3 Source Data [file 44318_2026_705_MOESM21_ESM.zip › Figure EV3-1/B/STARD3_LAMP1_NT/20250120_MCF7STARD3WT_NT2_SR_w3SPI 405 DAPI.TIF]

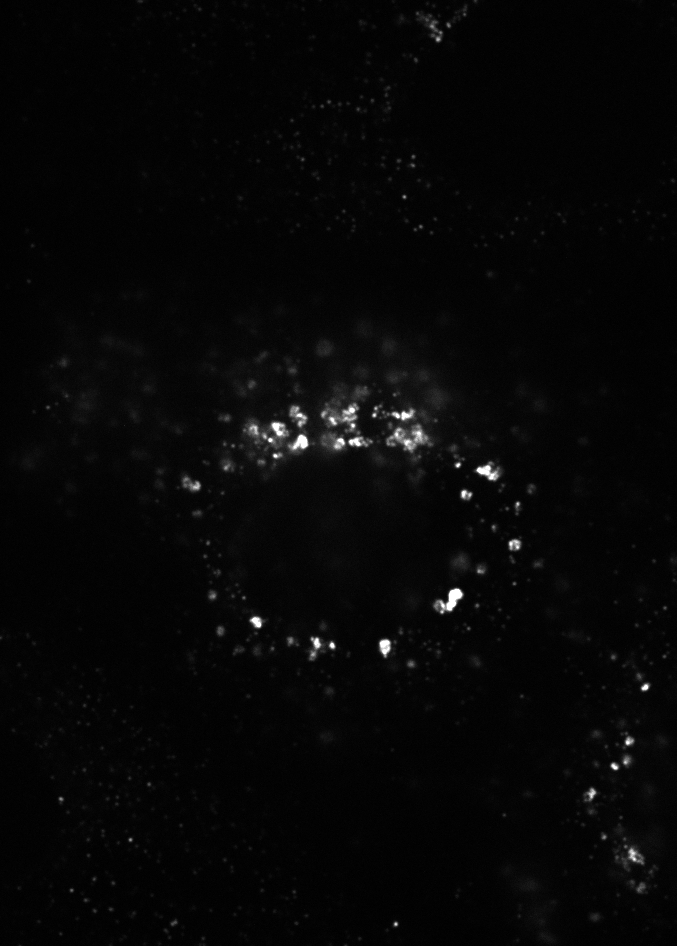

Supplement: Supplementary file 21 — Figure EV3 Source Data [file 44318_2026_705_MOESM21_ESM.zip › Figure EV3-1/B/STARD3_LAMP1_NT/20250120_MCF7STARD3WT_NT2_w1SPI 491 GFP.TIF]

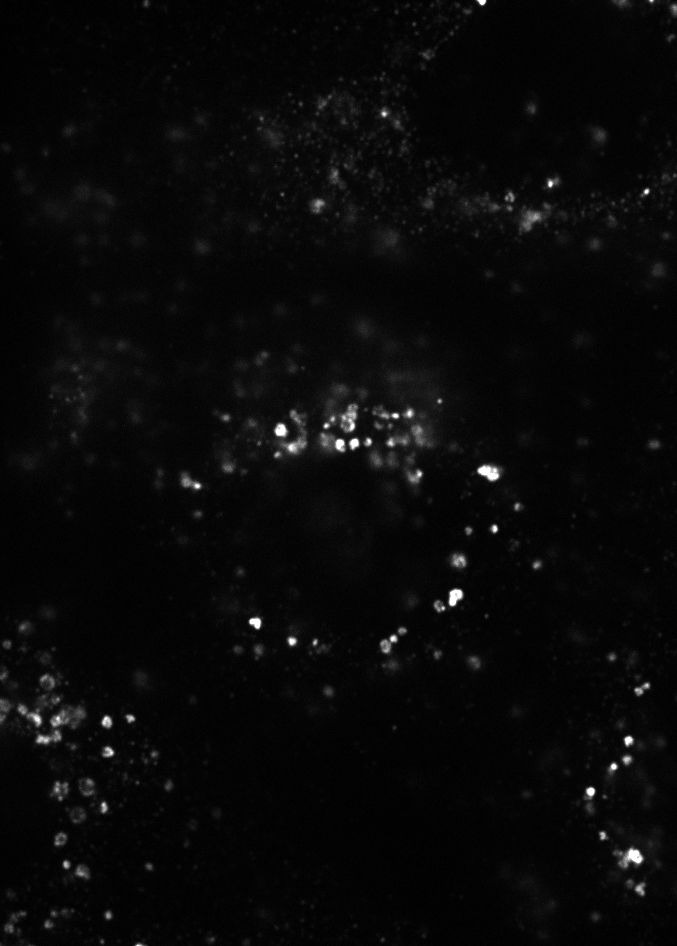

Supplement: Supplementary file 21 — Figure EV3 Source Data [file 44318_2026_705_MOESM21_ESM.zip › Figure EV3-1/B/STARD3_LAMP1_NT/20250120_MCF7STARD3WT_NT2_w2SPI 561 mCherry.TIF]

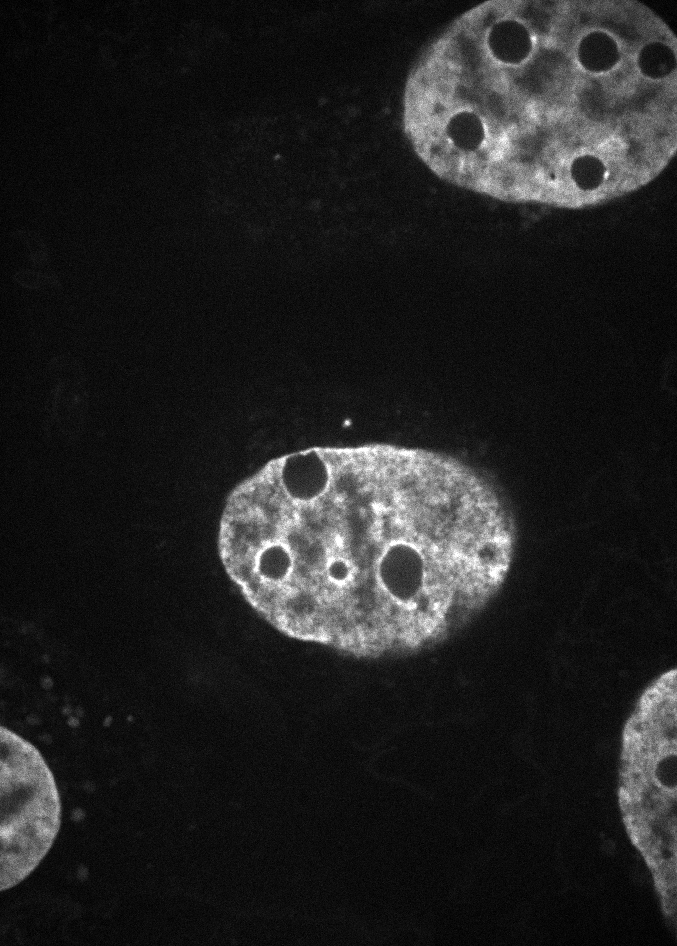

Supplement: Supplementary file 21 — Figure EV3 Source Data [file 44318_2026_705_MOESM21_ESM.zip › Figure EV3-1/B/STARD3_LAMP1_NT/20250120_MCF7STARD3WT_NT2_w3SPI 405 DAPI.TIF]

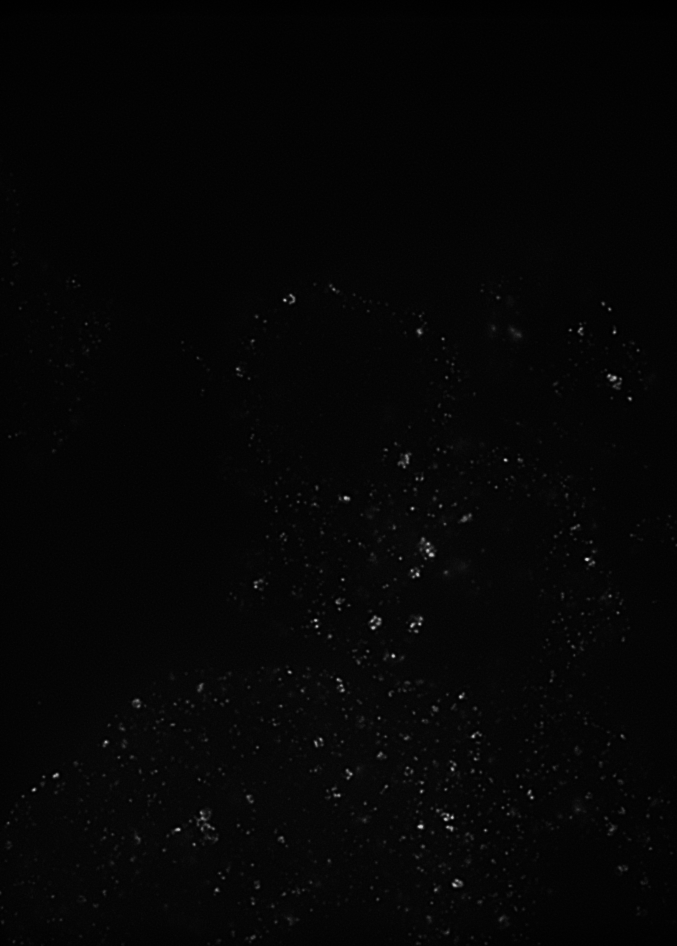

Supplement: Supplementary file 21 — Figure EV3 Source Data [file 44318_2026_705_MOESM21_ESM.zip › Figure EV3-1/C/STARD3_EEA1_CHIR99021/20230224_MCF7STARD3WTredEEA1green_GSK3i_4_SR_w1SPI 491 GFP.TIF]

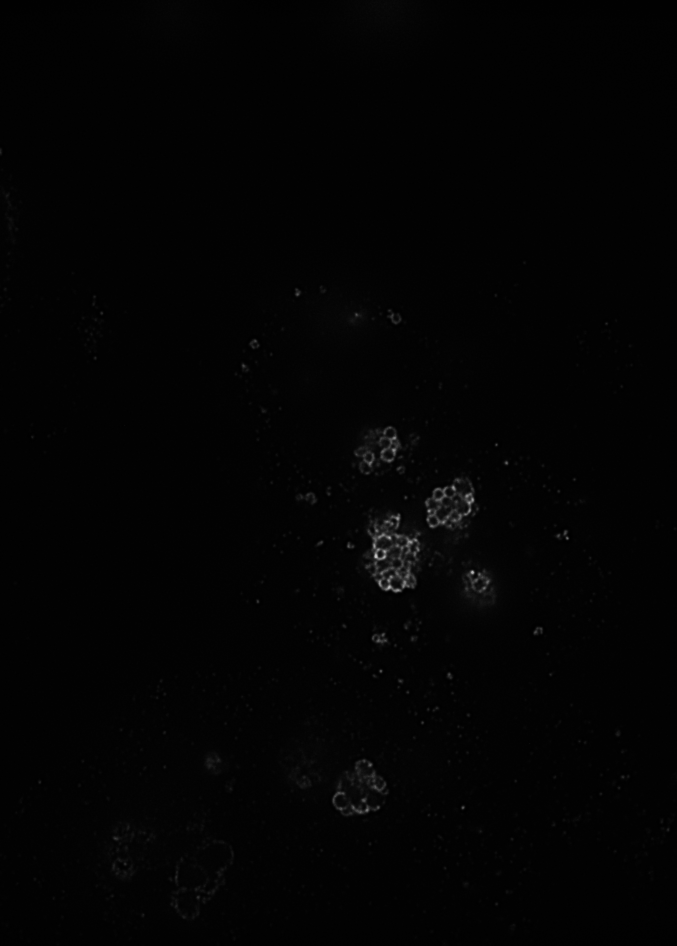

Supplement: Supplementary file 21 — Figure EV3 Source Data [file 44318_2026_705_MOESM21_ESM.zip › Figure EV3-1/C/STARD3_EEA1_CHIR99021/20230224_MCF7STARD3WTredEEA1green_GSK3i_4_SR_w2SPI 561 mCherry.TIF]

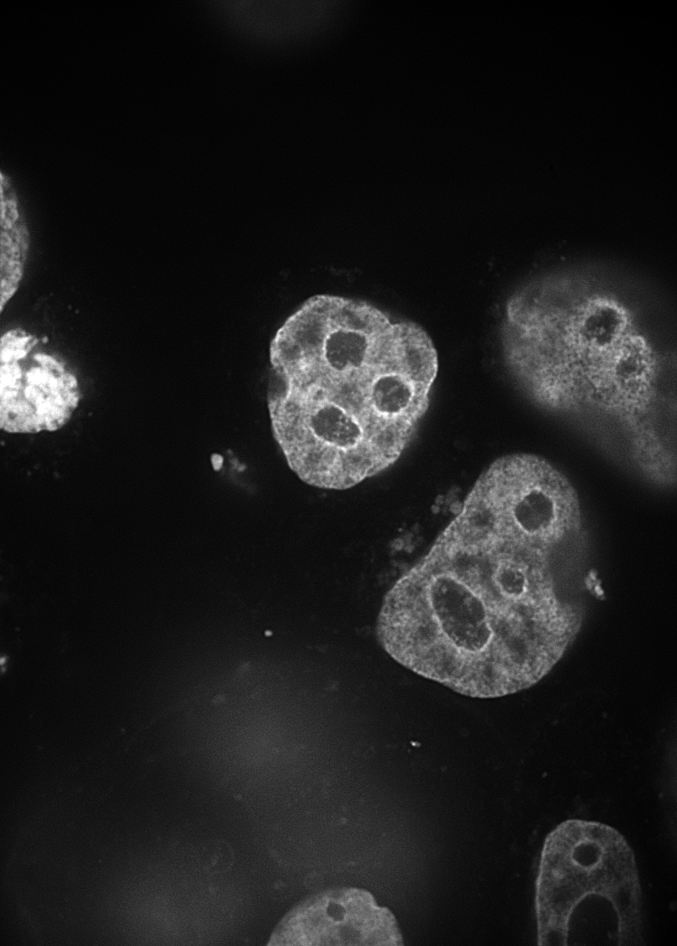

Supplement: Supplementary file 21 — Figure EV3 Source Data [file 44318_2026_705_MOESM21_ESM.zip › Figure EV3-1/C/STARD3_EEA1_CHIR99021/20230224_MCF7STARD3WTredEEA1green_GSK3i_4_SR_w3SPI 405 DAPI.TIF]

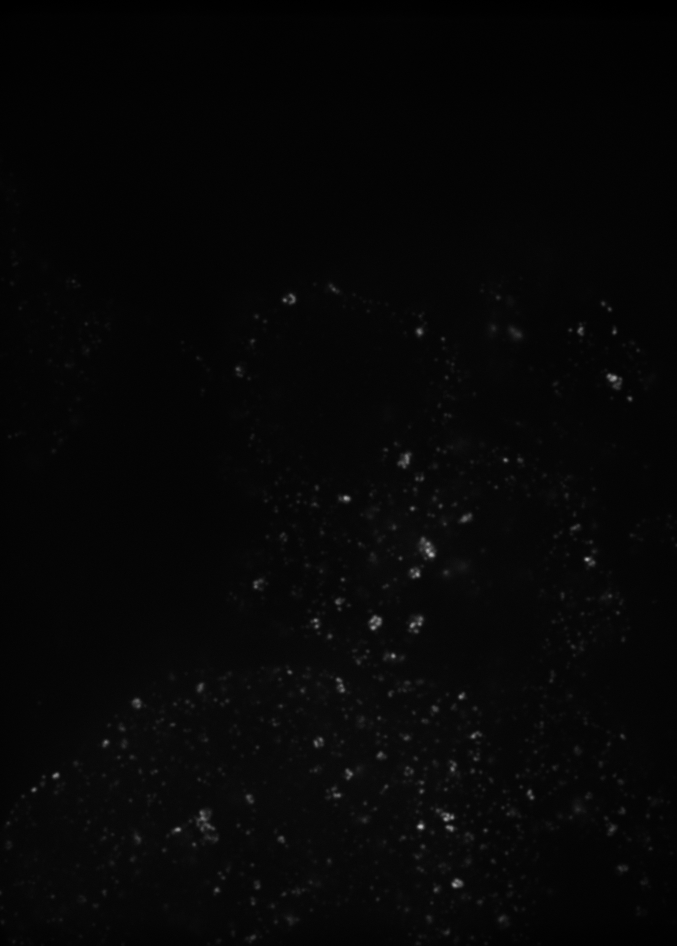

Supplement: Supplementary file 21 — Figure EV3 Source Data [file 44318_2026_705_MOESM21_ESM.zip › Figure EV3-1/C/STARD3_EEA1_CHIR99021/20230224_MCF7STARD3WTredEEA1green_GSK3i_4_w1SPI 491 GFP.TIF]

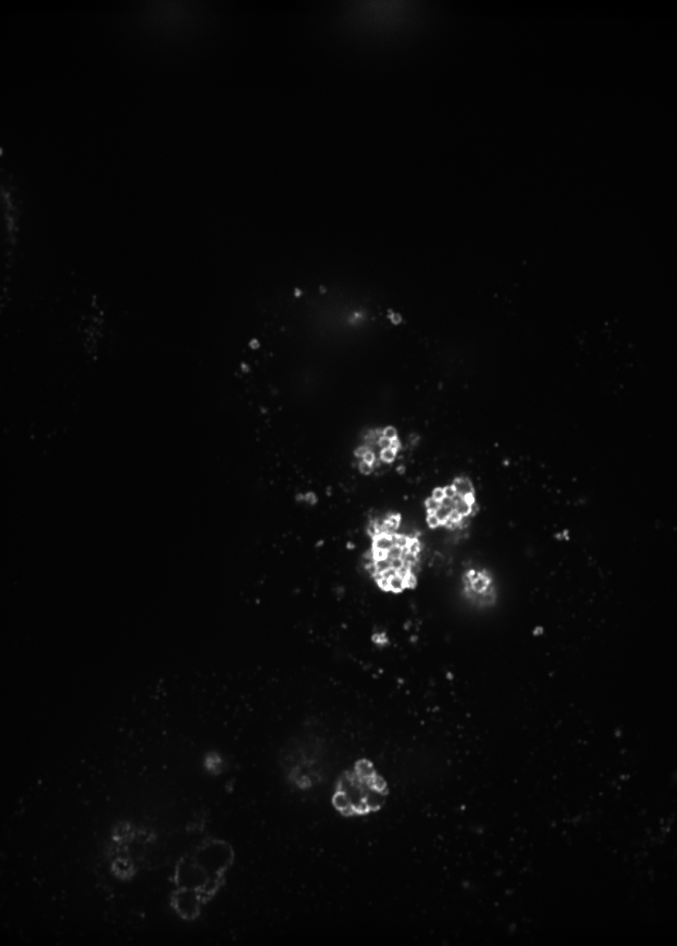

Supplement: Supplementary file 21 — Figure EV3 Source Data [file 44318_2026_705_MOESM21_ESM.zip › Figure EV3-1/C/STARD3_EEA1_CHIR99021/20230224_MCF7STARD3WTredEEA1green_GSK3i_4_w2SPI 561 mCherry.TIF]

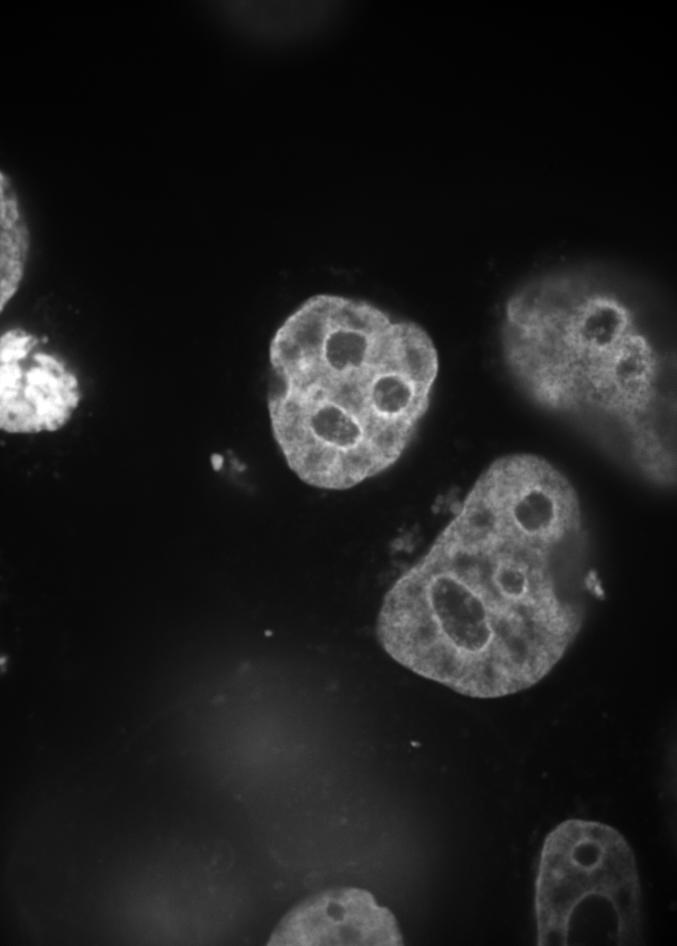

Supplement: Supplementary file 21 — Figure EV3 Source Data [file 44318_2026_705_MOESM21_ESM.zip › Figure EV3-1/C/STARD3_EEA1_CHIR99021/20230224_MCF7STARD3WTredEEA1green_GSK3i_4_w3SPI 405 DAPI.TIF]

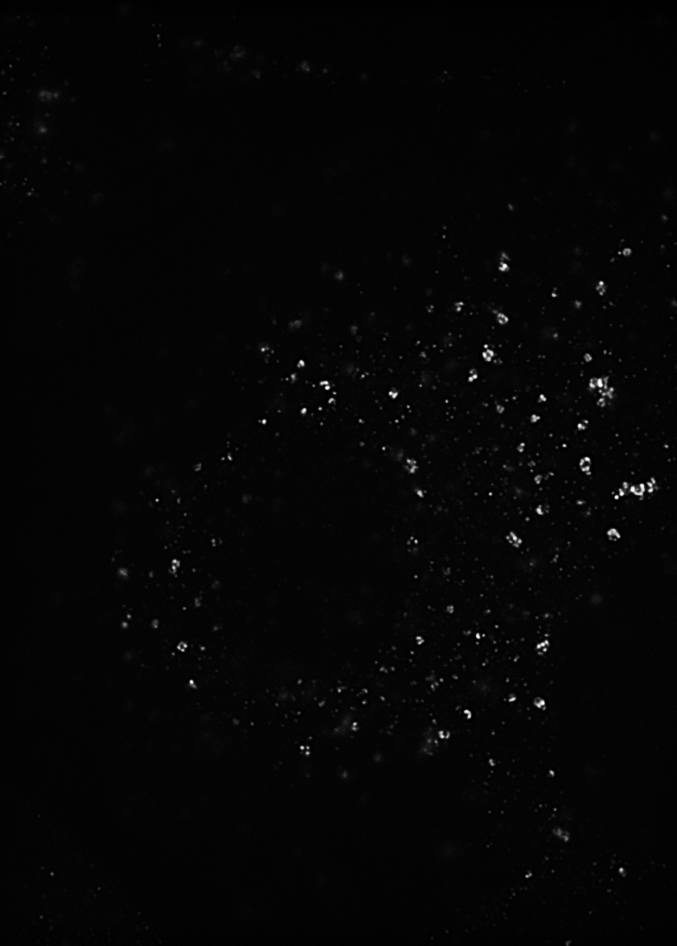

Supplement: Supplementary file 21 — Figure EV3 Source Data [file 44318_2026_705_MOESM21_ESM.zip › Figure EV3-1/C/STARD3_EEA1_NT/20230224_MCF7STARD3WTredEEA1green_NT_1_SR_w1SPI 491 GFP.TIF]

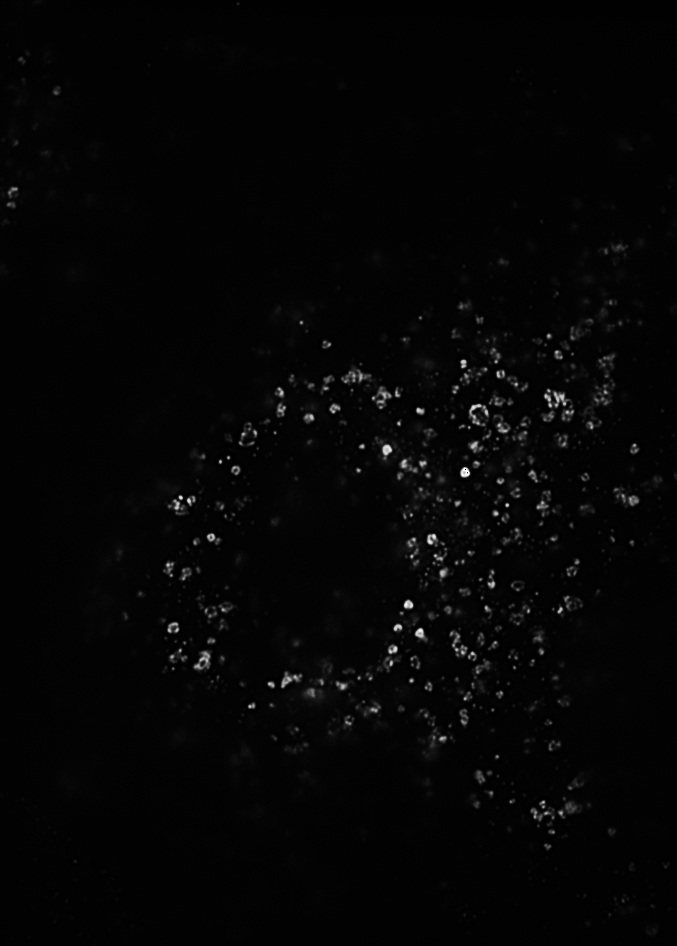

Supplement: Supplementary file 21 — Figure EV3 Source Data [file 44318_2026_705_MOESM21_ESM.zip › Figure EV3-1/C/STARD3_EEA1_NT/20230224_MCF7STARD3WTredEEA1green_NT_1_SR_w2SPI 561 mCherry.TIF]

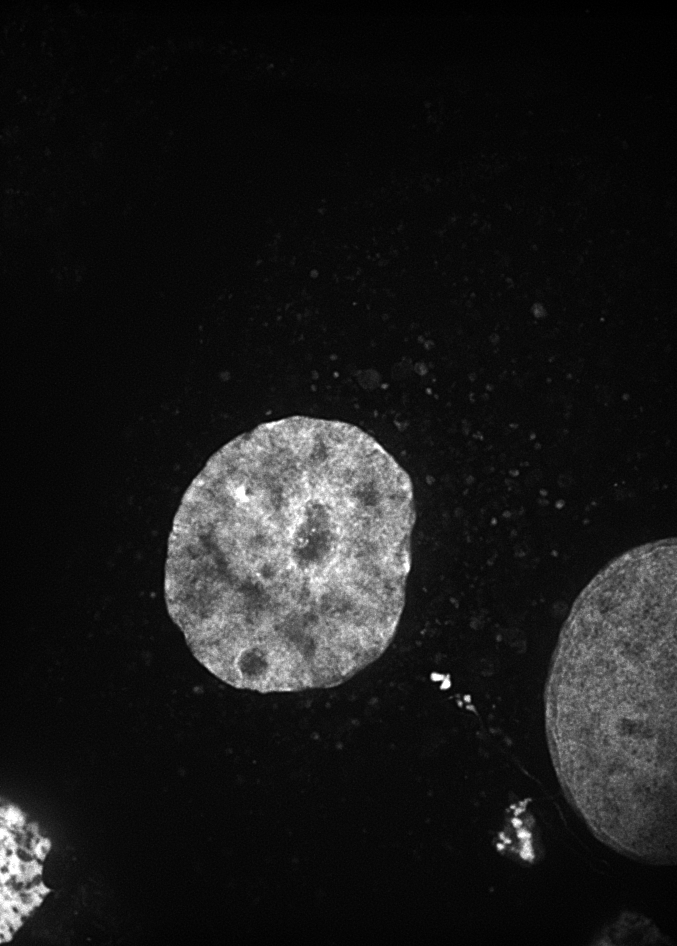

Supplement: Supplementary file 21 — Figure EV3 Source Data [file 44318_2026_705_MOESM21_ESM.zip › Figure EV3-1/C/STARD3_EEA1_NT/20230224_MCF7STARD3WTredEEA1green_NT_1_SR_w3SPI 405 DAPI.TIF]

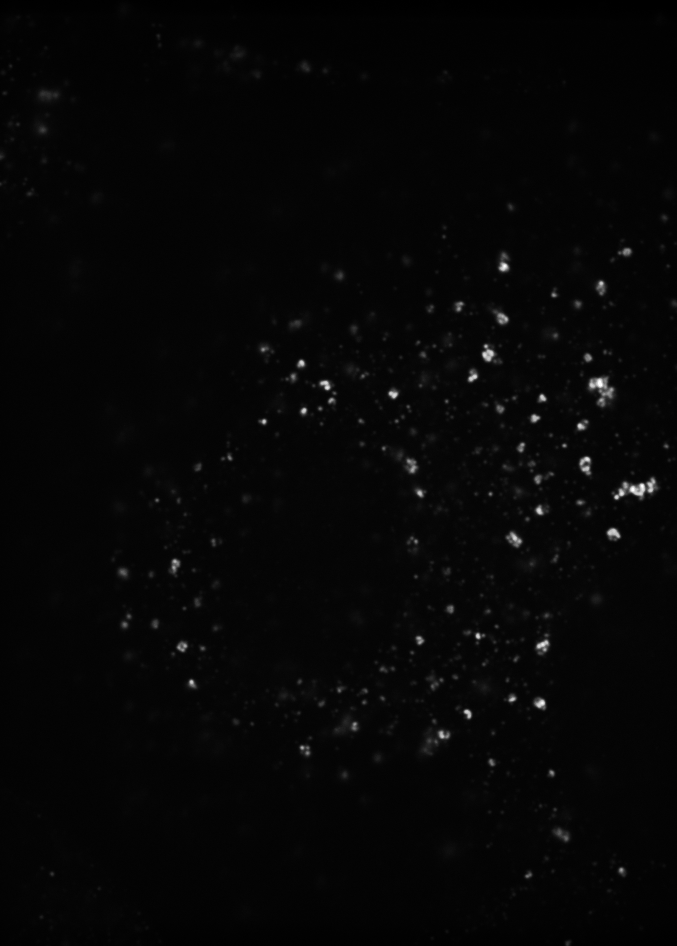

Supplement: Supplementary file 21 — Figure EV3 Source Data [file 44318_2026_705_MOESM21_ESM.zip › Figure EV3-1/C/STARD3_EEA1_NT/20230224_MCF7STARD3WTredEEA1green_NT_1_w1SPI 491 GFP.TIF]

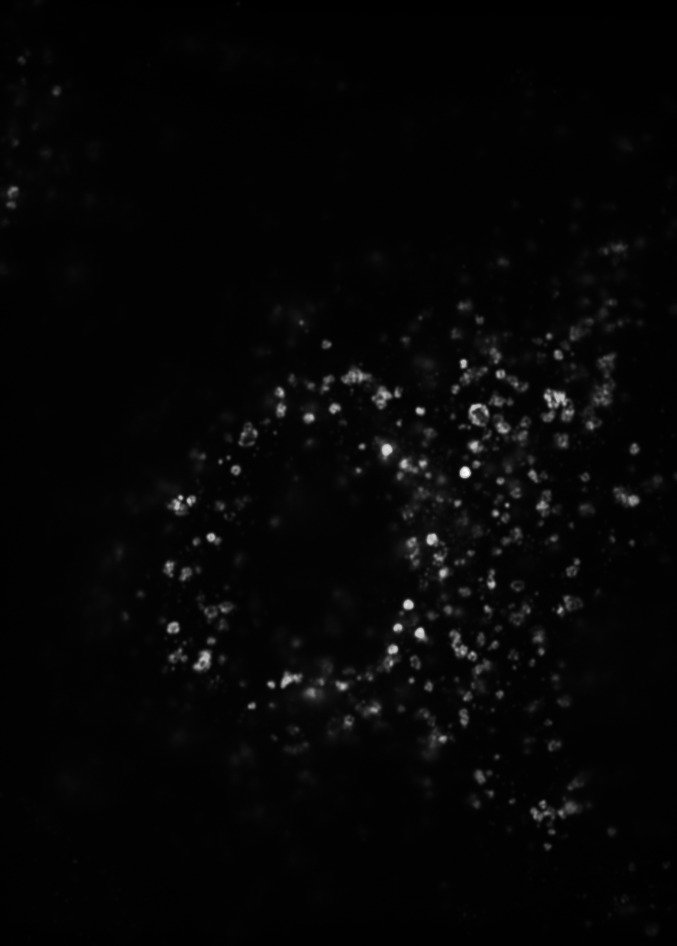

Supplement: Supplementary file 21 — Figure EV3 Source Data [file 44318_2026_705_MOESM21_ESM.zip › Figure EV3-1/C/STARD3_EEA1_NT/20230224_MCF7STARD3WTredEEA1green_NT_1_w2SPI 561 mCherry.TIF]

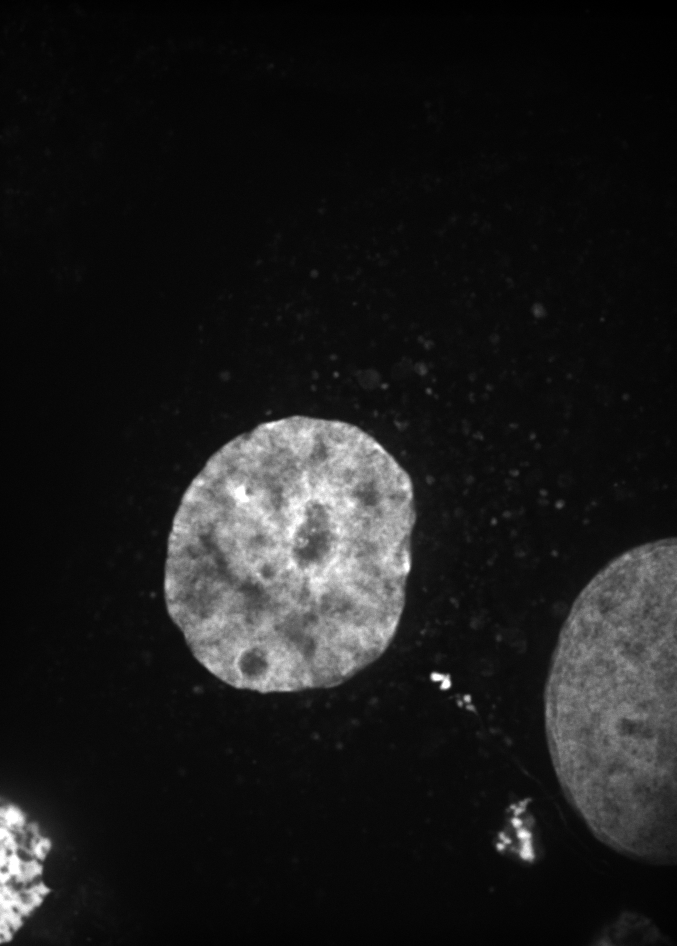

Supplement: Supplementary file 21 — Figure EV3 Source Data [file 44318_2026_705_MOESM21_ESM.zip › Figure EV3-1/C/STARD3_EEA1_NT/20230224_MCF7STARD3WTredEEA1green_NT_1_w3SPI 405 DAPI.TIF]

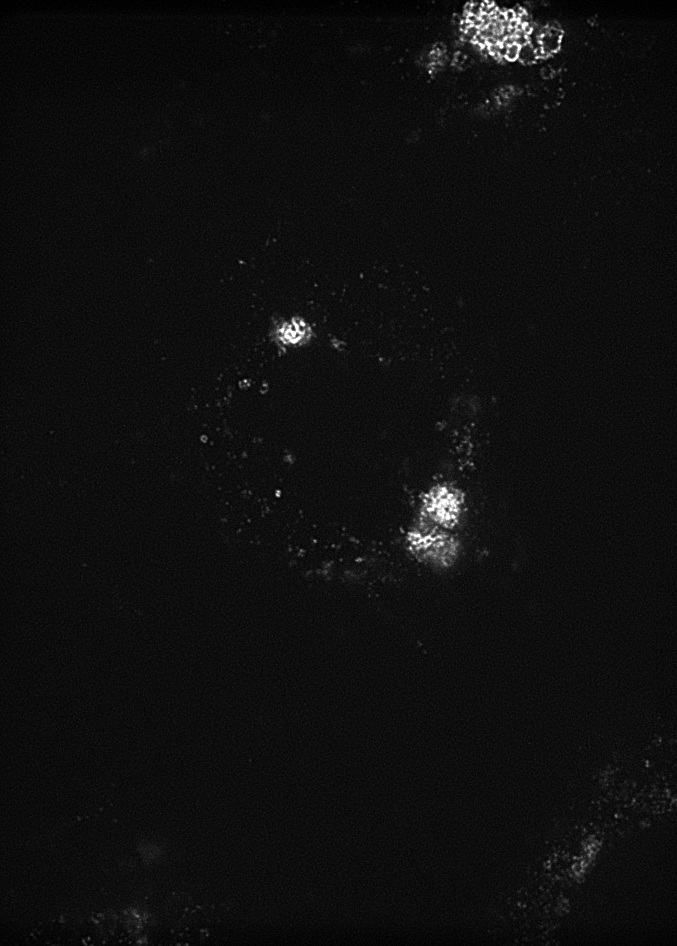

Supplement: Supplementary file 22 — Figure EV4 Source Data [file 44318_2026_705_MOESM22_ESM.zip › Figure EV3-2/D/STARD3_GM130_CHIR99021/20230224_MCF7STARD3SWTgreenGOLGIred_GSK3i_2_SR_w1SPI 491 GFP.TIF]

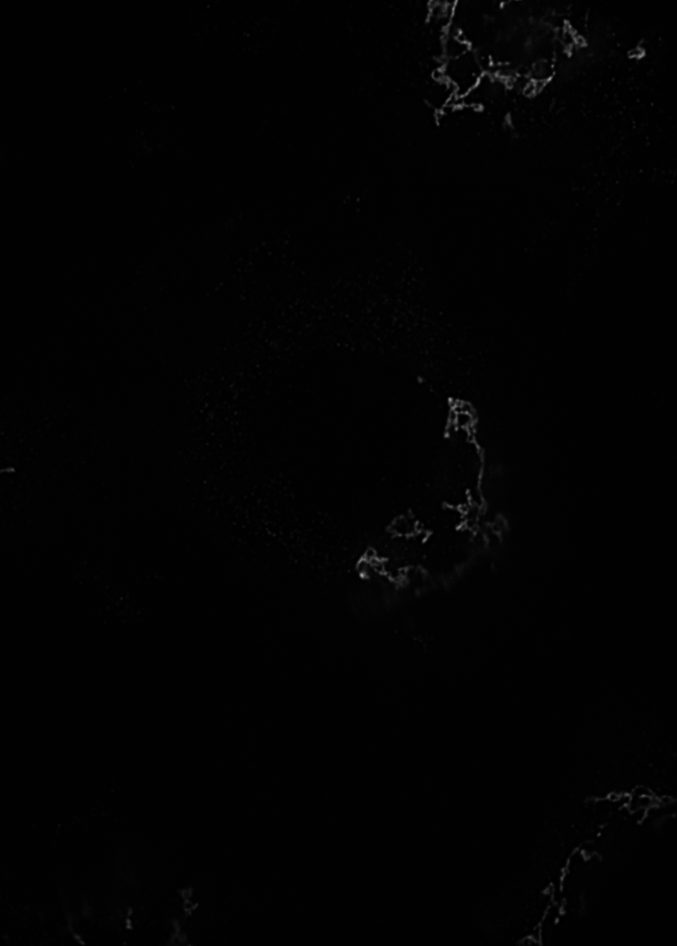

Supplement: Supplementary file 22 — Figure EV4 Source Data [file 44318_2026_705_MOESM22_ESM.zip › Figure EV3-2/D/STARD3_GM130_CHIR99021/20230224_MCF7STARD3SWTgreenGOLGIred_GSK3i_2_SR_w2SPI 561 mCherry.TIF]

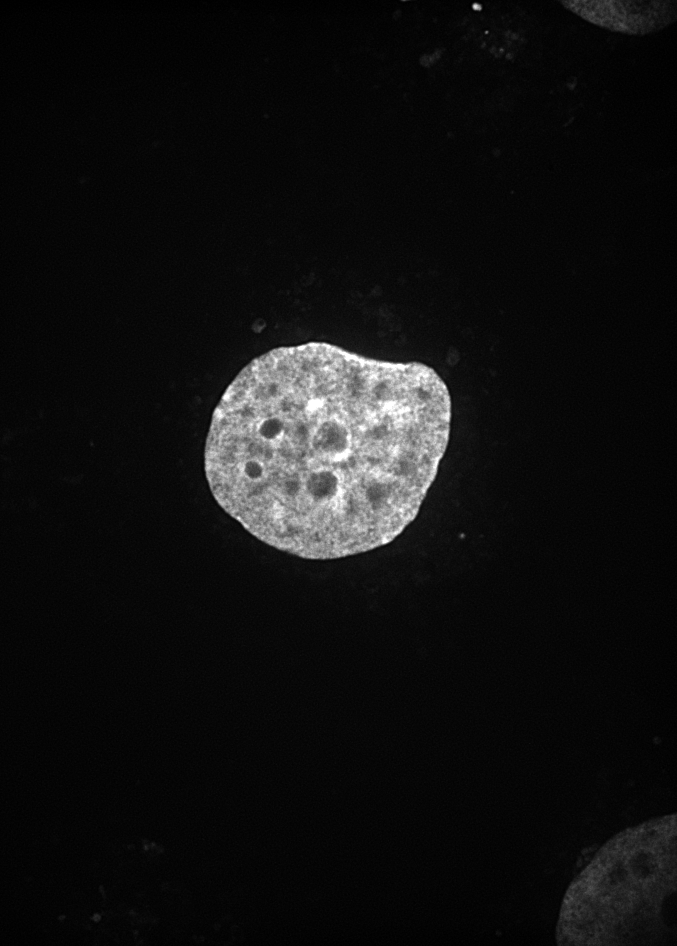

Supplement: Supplementary file 22 — Figure EV4 Source Data [file 44318_2026_705_MOESM22_ESM.zip › Figure EV3-2/D/STARD3_GM130_CHIR99021/20230224_MCF7STARD3SWTgreenGOLGIred_GSK3i_2_SR_w3SPI 405 DAPI.TIF]

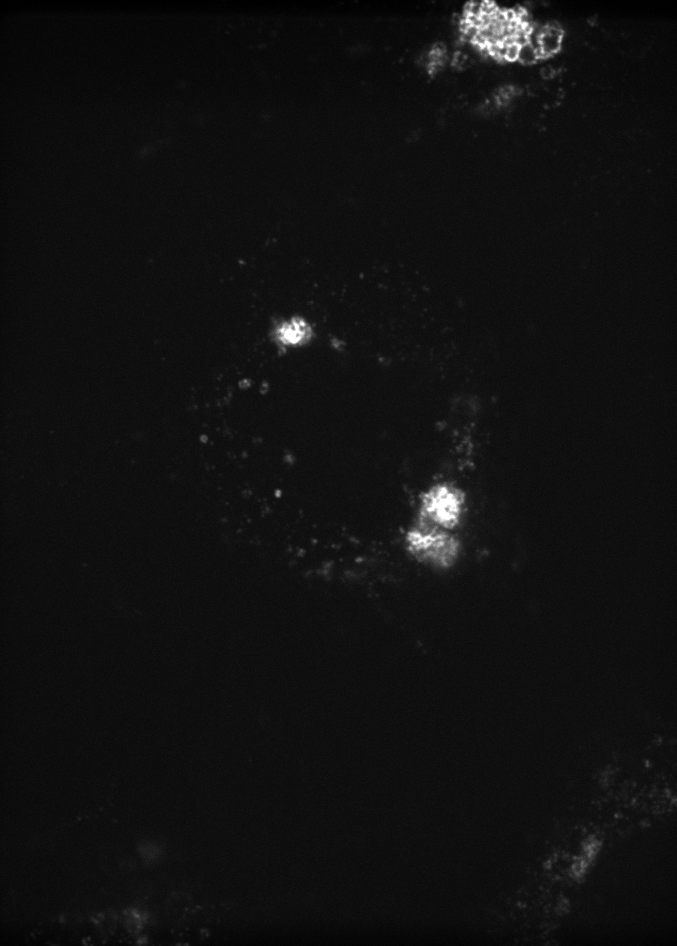

Supplement: Supplementary file 22 — Figure EV4 Source Data [file 44318_2026_705_MOESM22_ESM.zip › Figure EV3-2/D/STARD3_GM130_CHIR99021/20230224_MCF7STARD3SWTgreenGOLGIred_GSK3i_2_w1SPI 491 GFP.TIF]

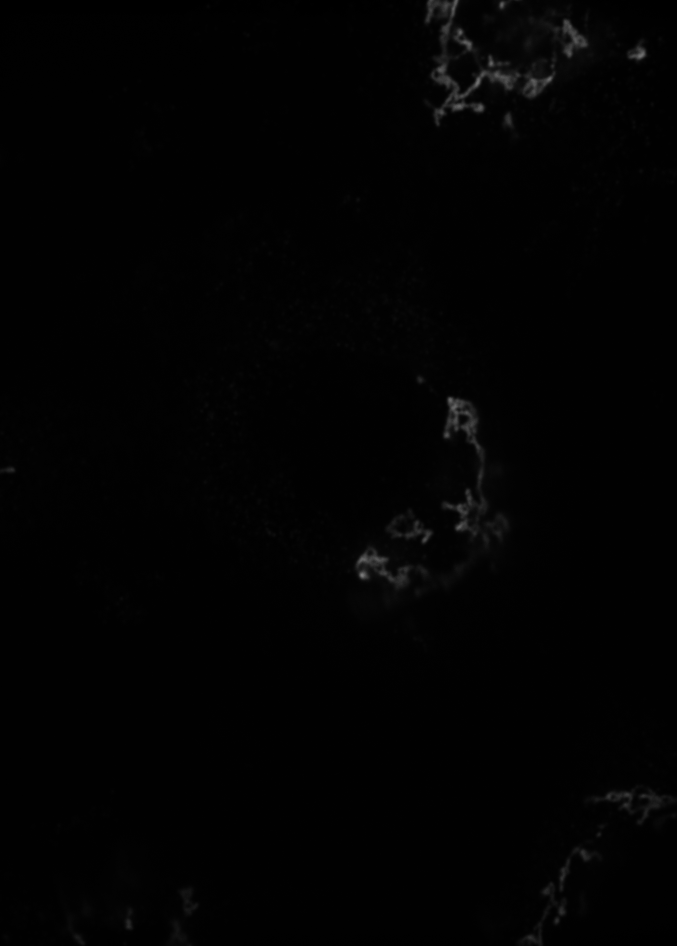

Supplement: Supplementary file 22 — Figure EV4 Source Data [file 44318_2026_705_MOESM22_ESM.zip › Figure EV3-2/D/STARD3_GM130_CHIR99021/20230224_MCF7STARD3SWTgreenGOLGIred_GSK3i_2_w2SPI 561 mCherry.TIF]

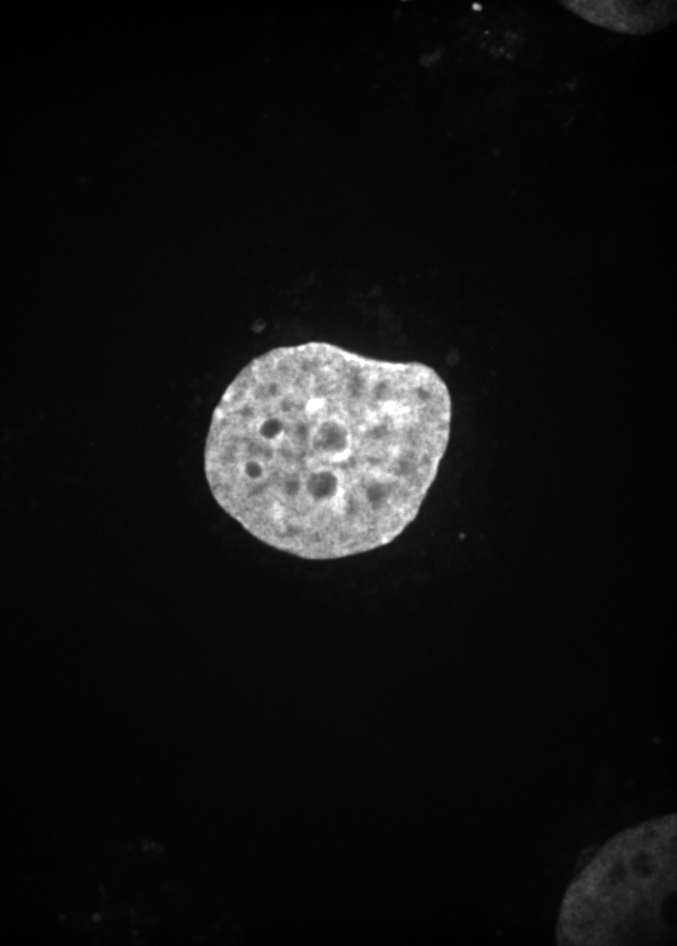

Supplement: Supplementary file 22 — Figure EV4 Source Data [file 44318_2026_705_MOESM22_ESM.zip › Figure EV3-2/D/STARD3_GM130_CHIR99021/20230224_MCF7STARD3SWTgreenGOLGIred_GSK3i_2_w3SPI 405 DAPI.TIF]

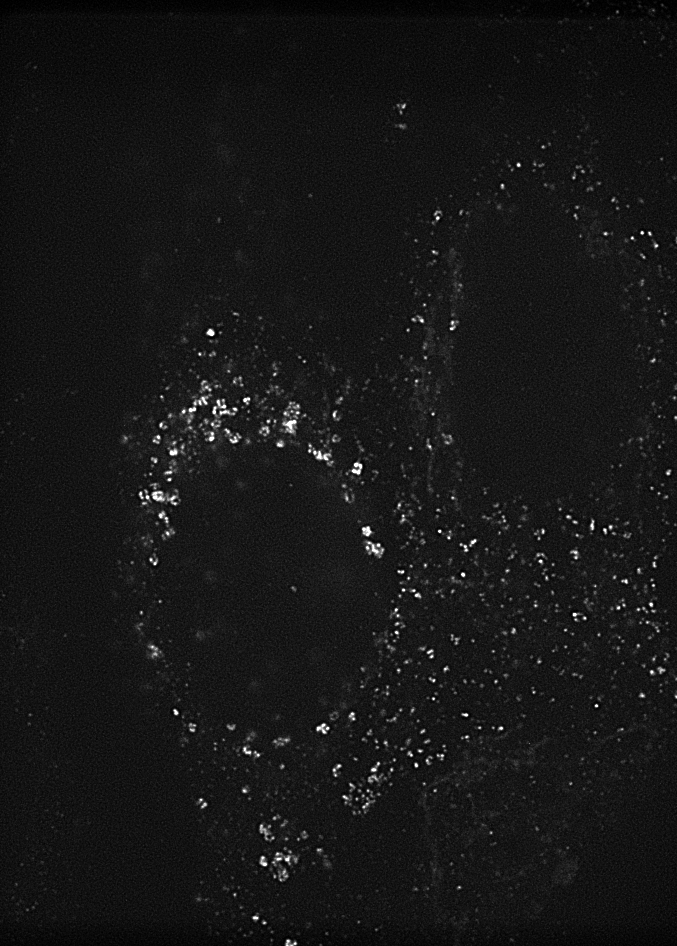

Supplement: Supplementary file 22 — Figure EV4 Source Data [file 44318_2026_705_MOESM22_ESM.zip › Figure EV3-2/D/STARD3_GM130_NT/20230224_MCF7STARD3SWTgreenGOLGIred_NT_4_SR_w1SPI 491 GFP.TIF]

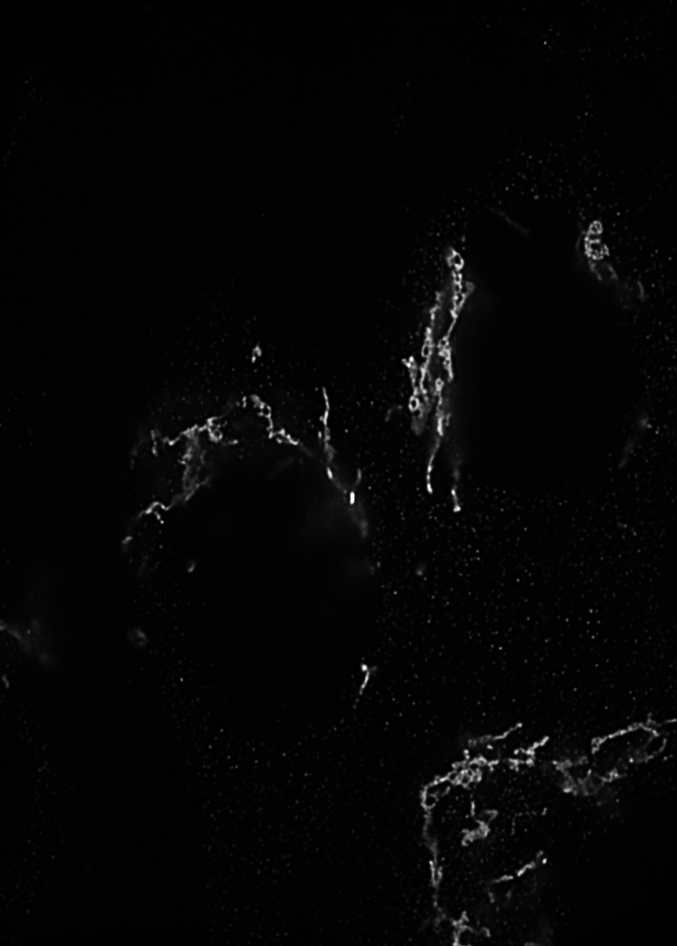

Supplement: Supplementary file 22 — Figure EV4 Source Data [file 44318_2026_705_MOESM22_ESM.zip › Figure EV3-2/D/STARD3_GM130_NT/20230224_MCF7STARD3SWTgreenGOLGIred_NT_4_SR_w2SPI 561 mCherry.TIF]

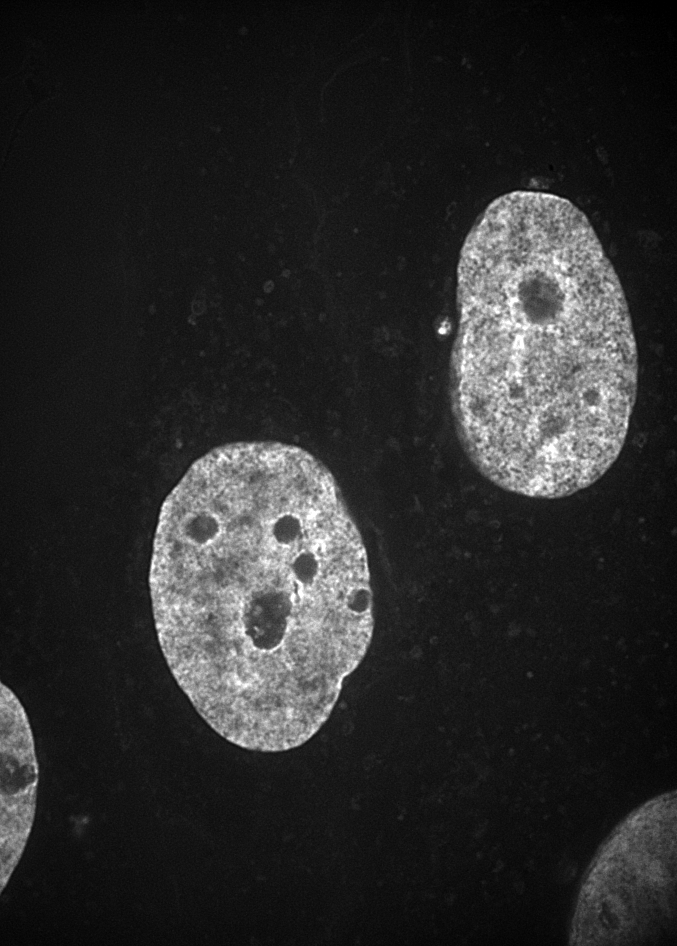

Supplement: Supplementary file 22 — Figure EV4 Source Data [file 44318_2026_705_MOESM22_ESM.zip › Figure EV3-2/D/STARD3_GM130_NT/20230224_MCF7STARD3SWTgreenGOLGIred_NT_4_SR_w3SPI 405 DAPI.TIF]

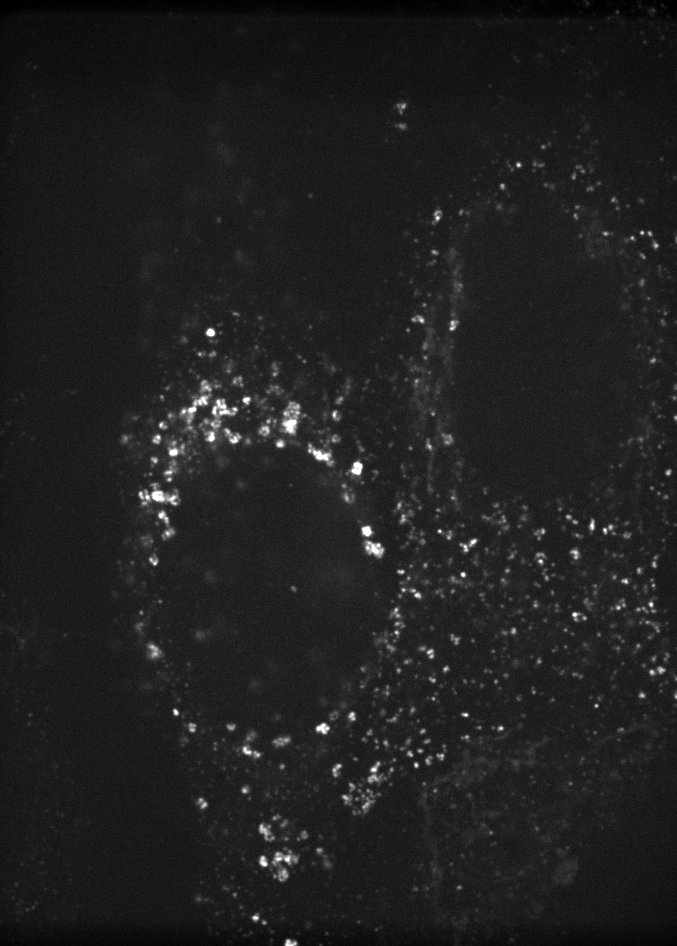

Supplement: Supplementary file 22 — Figure EV4 Source Data [file 44318_2026_705_MOESM22_ESM.zip › Figure EV3-2/D/STARD3_GM130_NT/20230224_MCF7STARD3SWTgreenGOLGIred_NT_4_w1SPI 491 GFP.TIF]

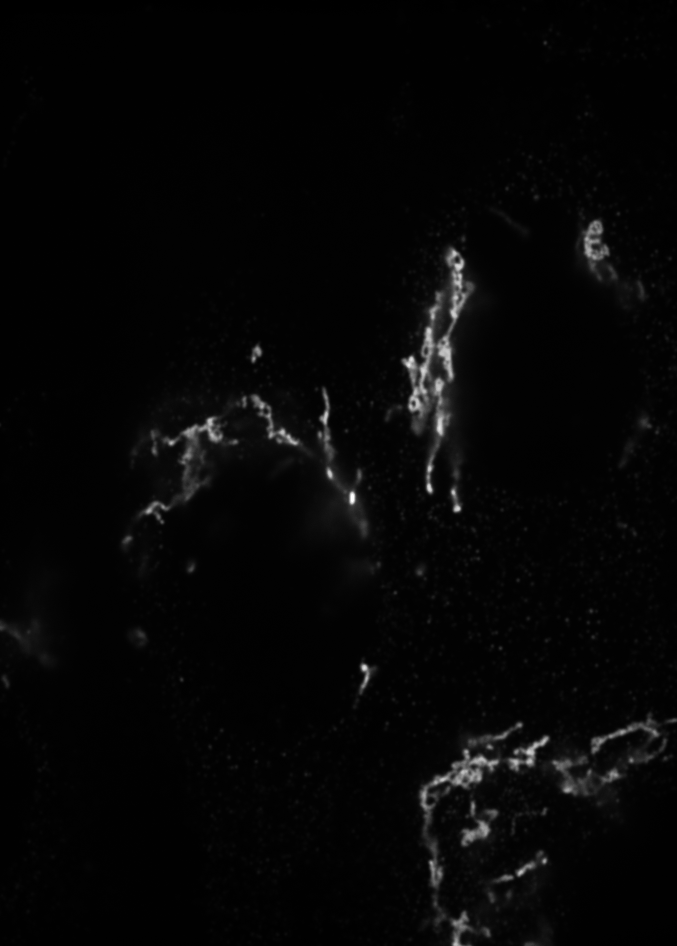

Supplement: Supplementary file 22 — Figure EV4 Source Data [file 44318_2026_705_MOESM22_ESM.zip › Figure EV3-2/D/STARD3_GM130_NT/20230224_MCF7STARD3SWTgreenGOLGIred_NT_4_w2SPI 561 mCherry.TIF]

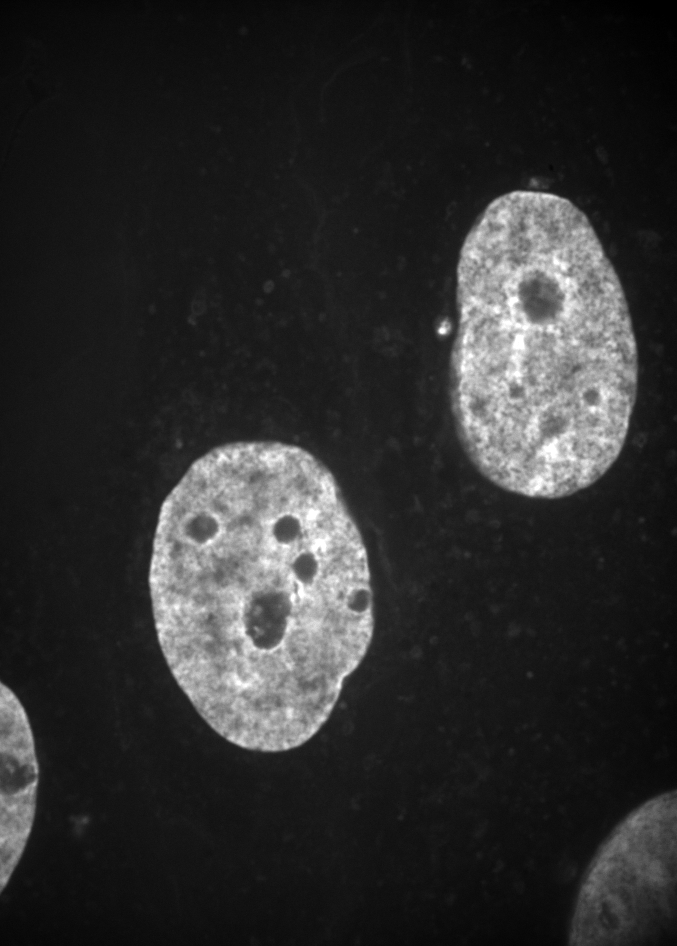

Supplement: Supplementary file 22 — Figure EV4 Source Data [file 44318_2026_705_MOESM22_ESM.zip › Figure EV3-2/D/STARD3_GM130_NT/20230224_MCF7STARD3SWTgreenGOLGIred_NT_4_w3SPI 405 DAPI.TIF]

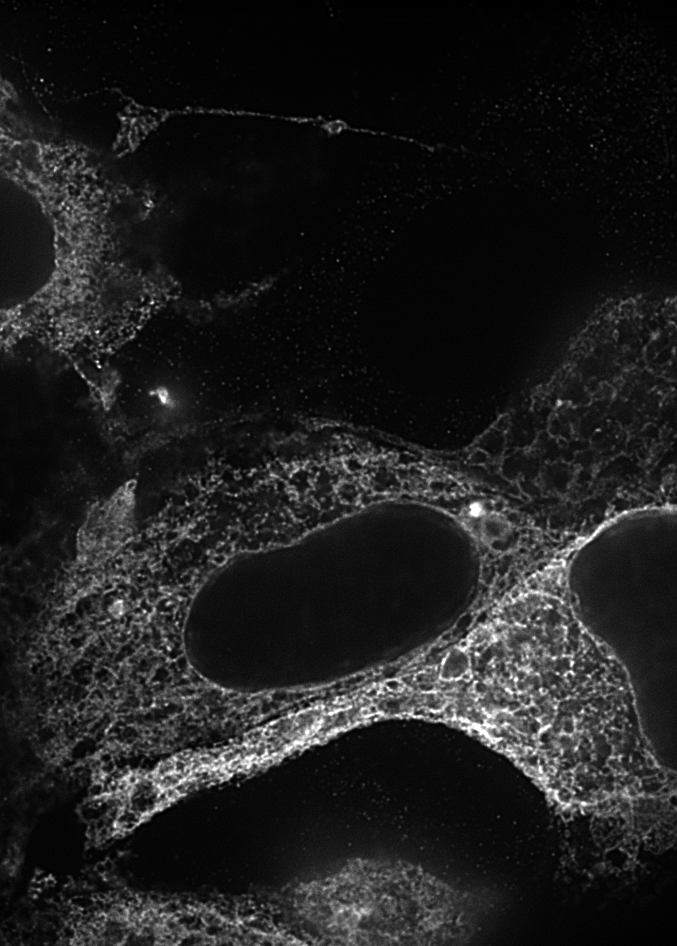

Supplement: Supplementary file 22 — Figure EV4 Source Data [file 44318_2026_705_MOESM22_ESM.zip › Figure EV3-2/E/STARD3_ER_CHIR99021/20230224_MCF7STARD3WTredERgreen_GSK3i_4_SR_w1SPI 491 GFP.TIF]

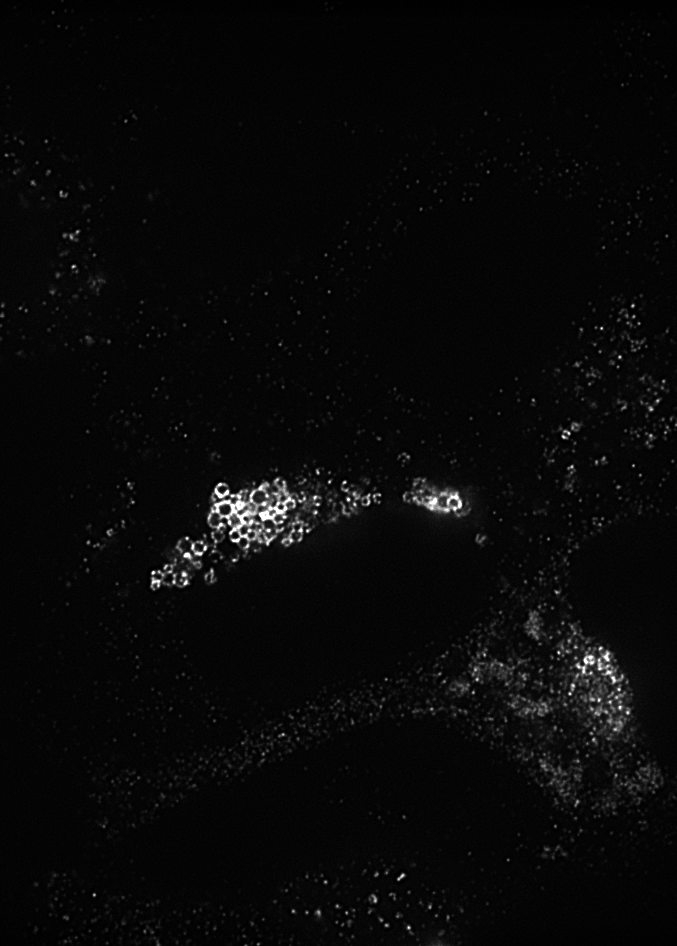

Supplement: Supplementary file 22 — Figure EV4 Source Data [file 44318_2026_705_MOESM22_ESM.zip › Figure EV3-2/E/STARD3_ER_CHIR99021/20230224_MCF7STARD3WTredERgreen_GSK3i_4_SR_w2SPI 561 mCherry.TIF]

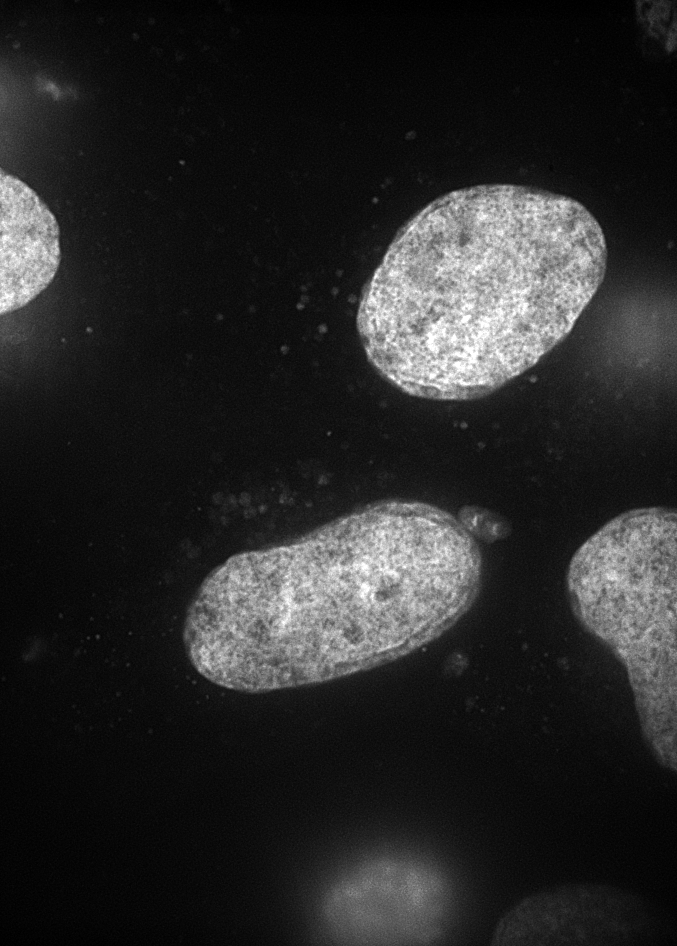

Supplement: Supplementary file 22 — Figure EV4 Source Data [file 44318_2026_705_MOESM22_ESM.zip › Figure EV3-2/E/STARD3_ER_CHIR99021/20230224_MCF7STARD3WTredERgreen_GSK3i_4_SR_w3SPI 405 DAPI.TIF]

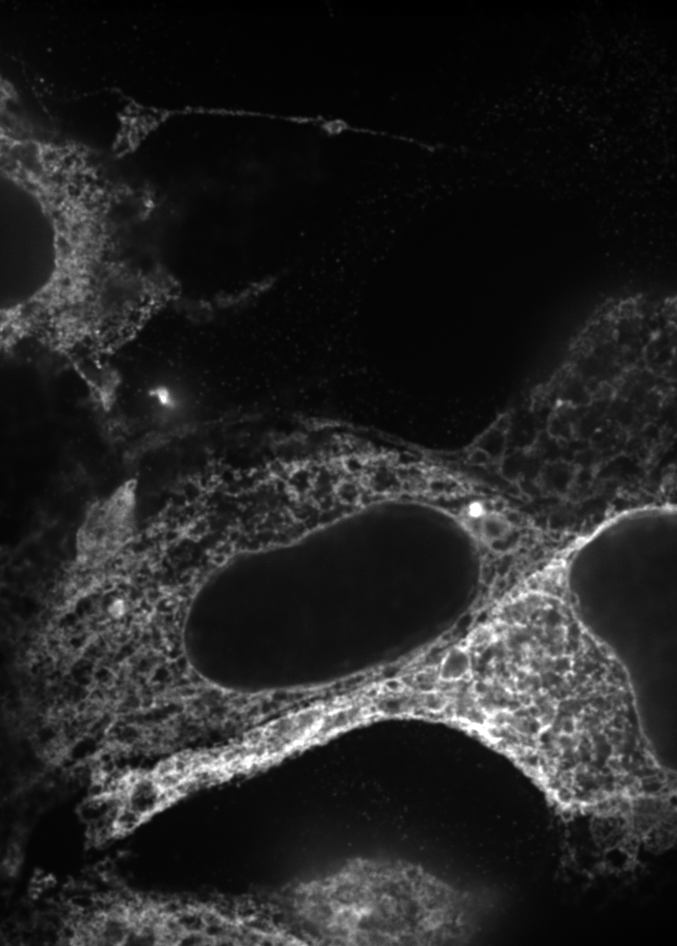

Supplement: Supplementary file 22 — Figure EV4 Source Data [file 44318_2026_705_MOESM22_ESM.zip › Figure EV3-2/E/STARD3_ER_CHIR99021/20230224_MCF7STARD3WTredERgreen_GSK3i_4_w1SPI 491 GFP.TIF]

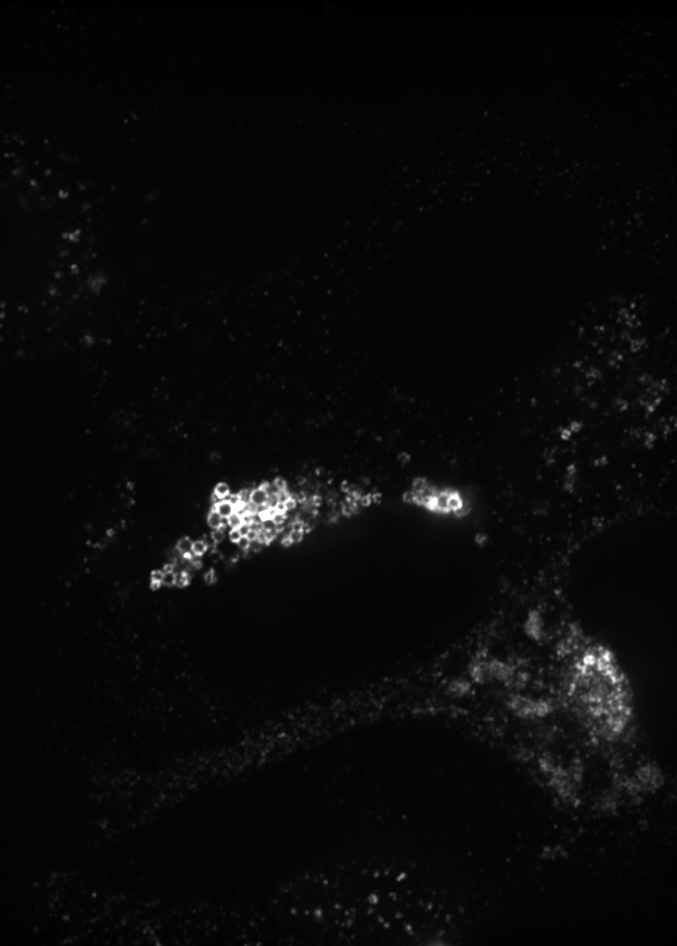

Supplement: Supplementary file 22 — Figure EV4 Source Data [file 44318_2026_705_MOESM22_ESM.zip › Figure EV3-2/E/STARD3_ER_CHIR99021/20230224_MCF7STARD3WTredERgreen_GSK3i_4_w2SPI 561 mCherry.TIF]

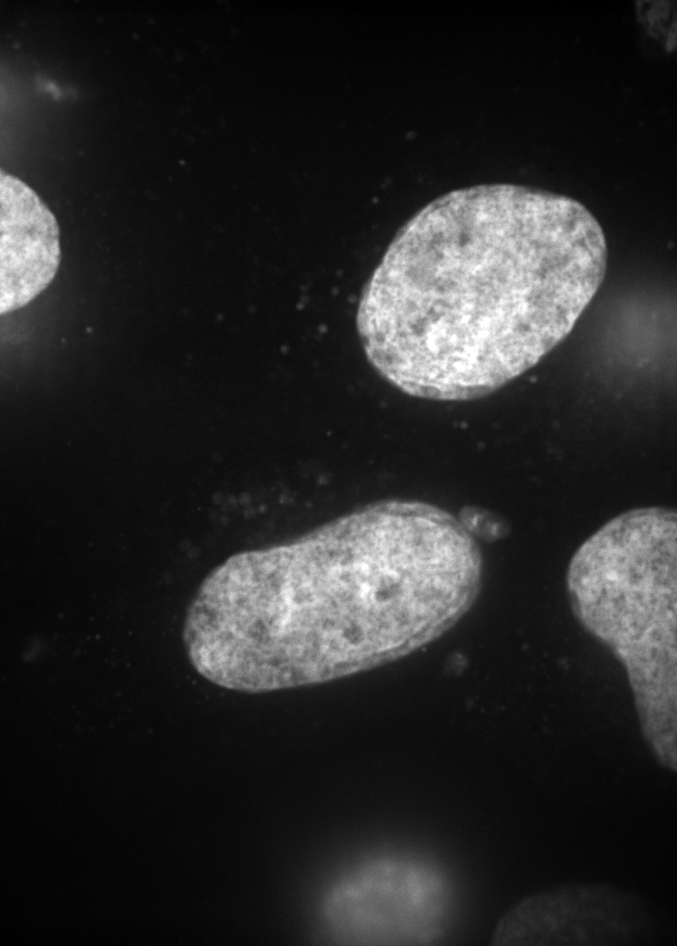

Supplement: Supplementary file 22 — Figure EV4 Source Data [file 44318_2026_705_MOESM22_ESM.zip › Figure EV3-2/E/STARD3_ER_CHIR99021/20230224_MCF7STARD3WTredERgreen_GSK3i_4_w3SPI 405 DAPI.TIF]

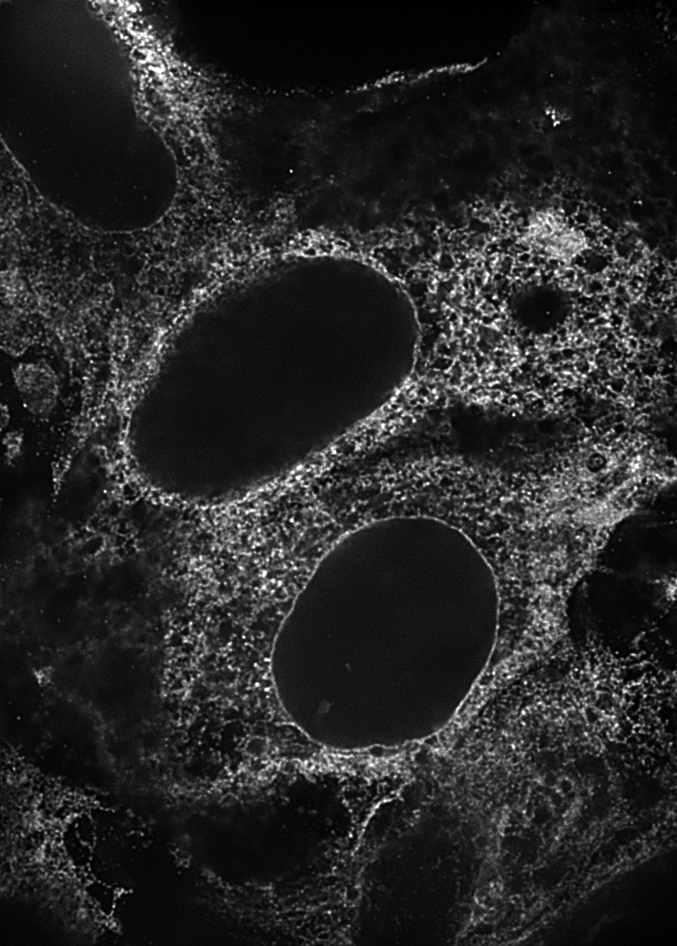

Supplement: Supplementary file 22 — Figure EV4 Source Data [file 44318_2026_705_MOESM22_ESM.zip › Figure EV3-2/E/STARD3_ER_NT/20230224_MCF7STARD3WTredERgreen_NT_5_SR_w1SPI 491 GFP.TIF]
