# Supplementary figures and images for: STARD3 regulates lysosome positioning and contacts via a GSK3-controlled phosphorylation switch (part 5 of 7)
Source: EMBO J. 2026 Feb 25;45(7):2239–77. doi: 10.1038/s44318-026-00705-3 (PMC13044316; doi:10.1038/s44318-026-00705-3)

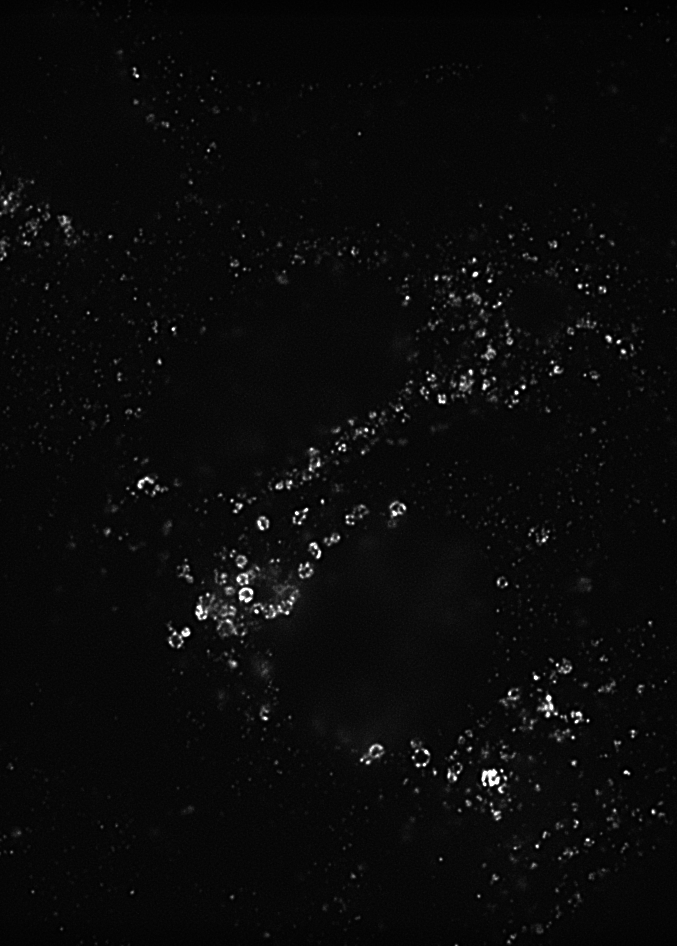

Supplement: Supplementary file 22 — Figure EV4 Source Data [file 44318_2026_705_MOESM22_ESM.zip › Figure EV3-2/E/STARD3_ER_NT/20230224_MCF7STARD3WTredERgreen_NT_5_SR_w2SPI 561 mCherry.TIF]

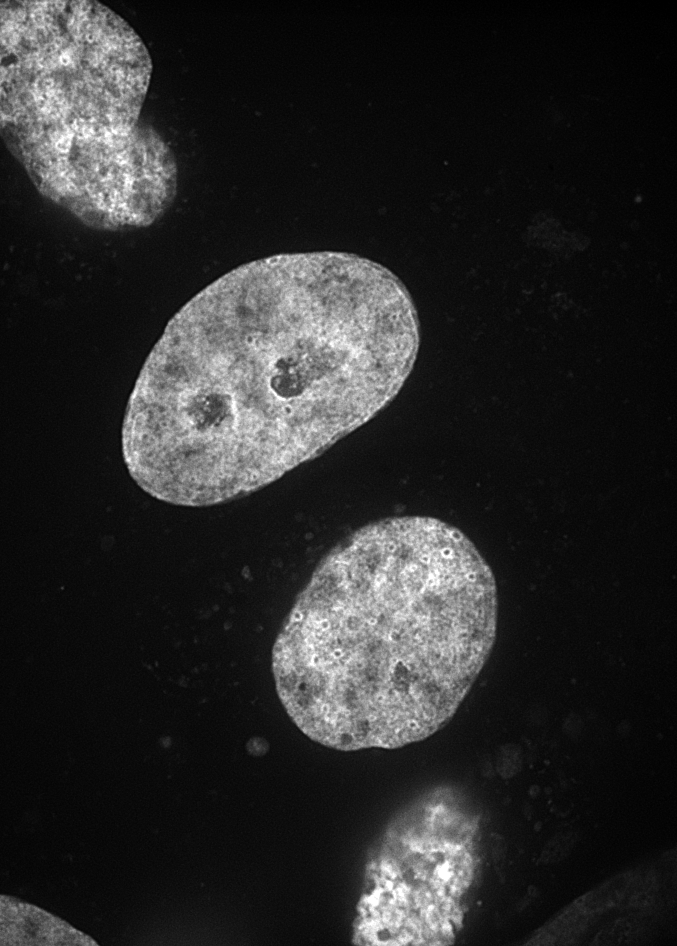

Supplement: Supplementary file 22 — Figure EV4 Source Data [file 44318_2026_705_MOESM22_ESM.zip › Figure EV3-2/E/STARD3_ER_NT/20230224_MCF7STARD3WTredERgreen_NT_5_SR_w3SPI 405 DAPI.TIF]

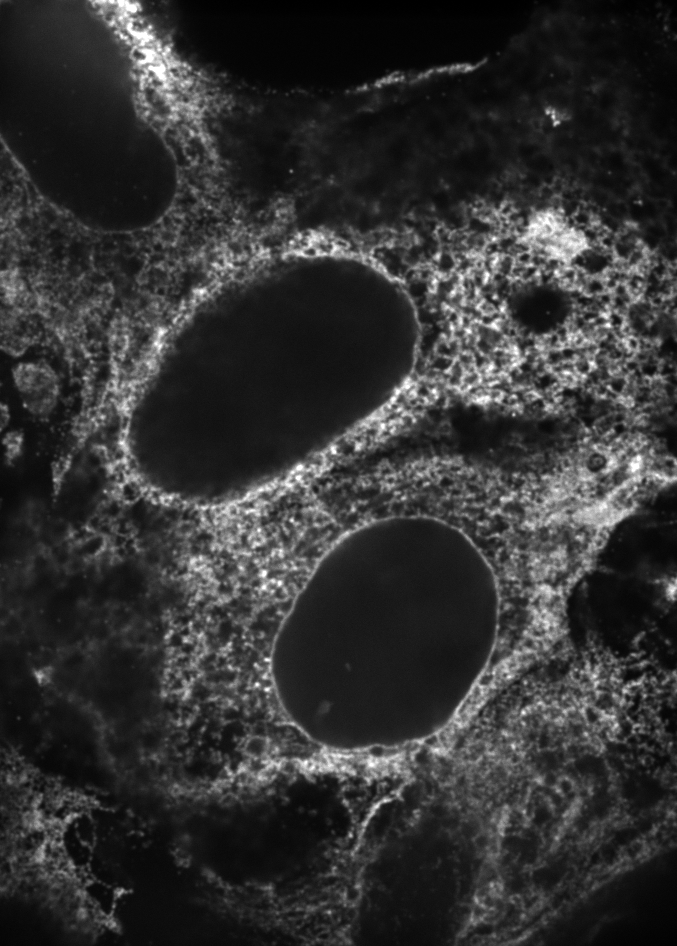

Supplement: Supplementary file 22 — Figure EV4 Source Data [file 44318_2026_705_MOESM22_ESM.zip › Figure EV3-2/E/STARD3_ER_NT/20230224_MCF7STARD3WTredERgreen_NT_5_w1SPI 491 GFP.TIF]

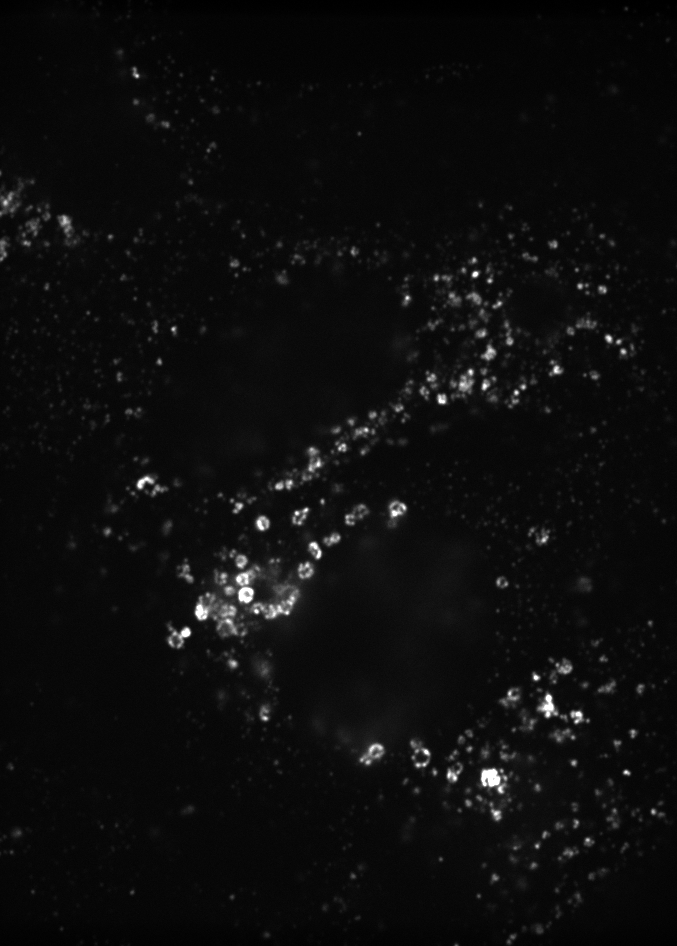

Supplement: Supplementary file 22 — Figure EV4 Source Data [file 44318_2026_705_MOESM22_ESM.zip › Figure EV3-2/E/STARD3_ER_NT/20230224_MCF7STARD3WTredERgreen_NT_5_w2SPI 561 mCherry.TIF]

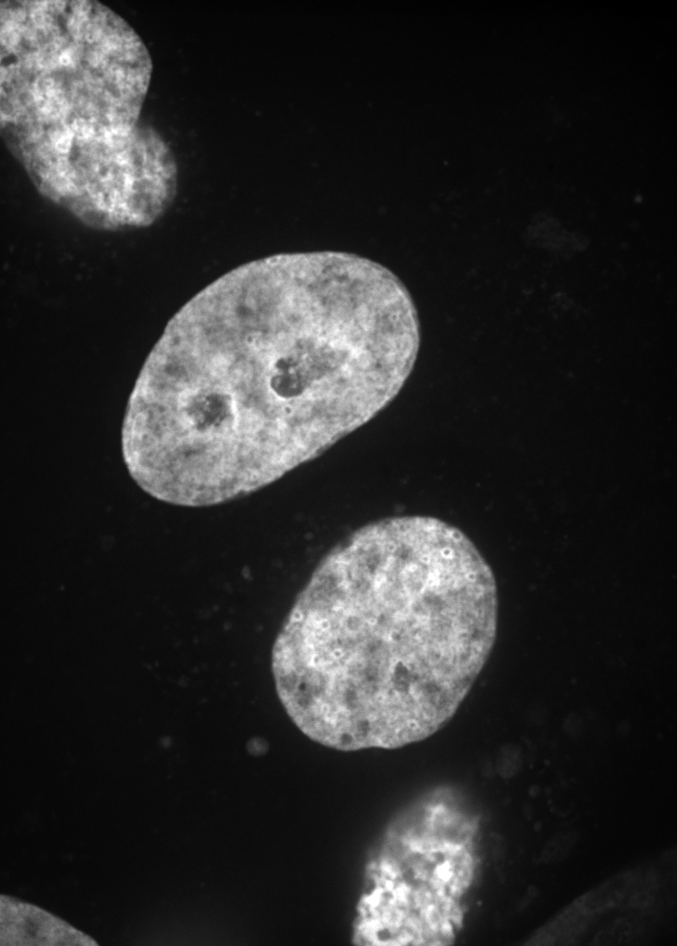

Supplement: Supplementary file 22 — Figure EV4 Source Data [file 44318_2026_705_MOESM22_ESM.zip › Figure EV3-2/E/STARD3_ER_NT/20230224_MCF7STARD3WTredERgreen_NT_5_w3SPI 405 DAPI.TIF]

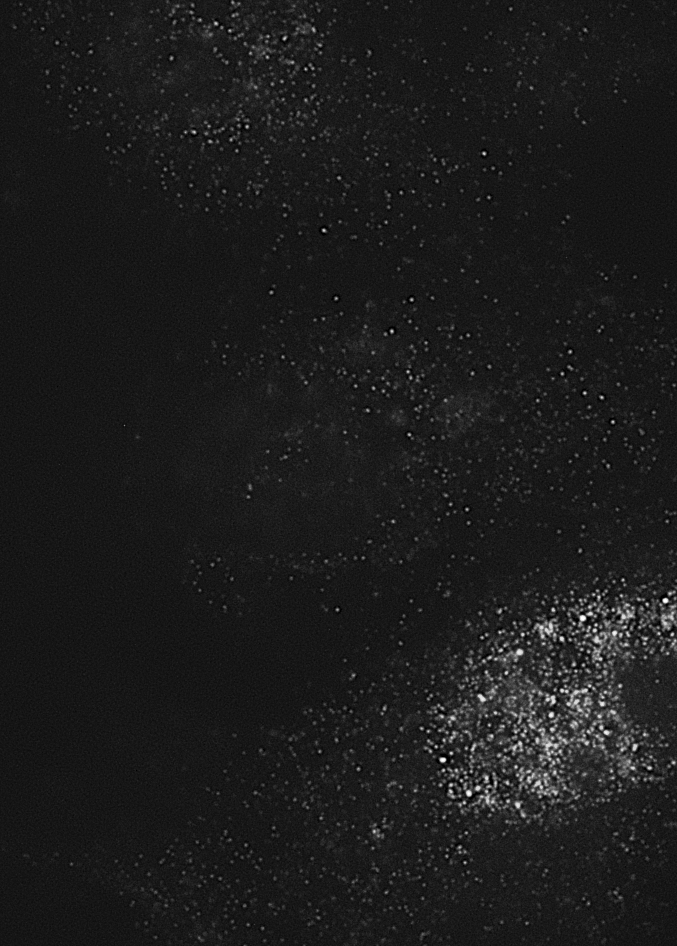

Supplement: Supplementary file 22 — Figure EV4 Source Data [file 44318_2026_705_MOESM22_ESM.zip › Figure EV3-2/F/STARD3FYAA_phospho_CHIR99021/20240523_MCF7STARD3FYAA_2G5r3144g_CHIR_3_SR_w1SPI 491 GFP.TIF]

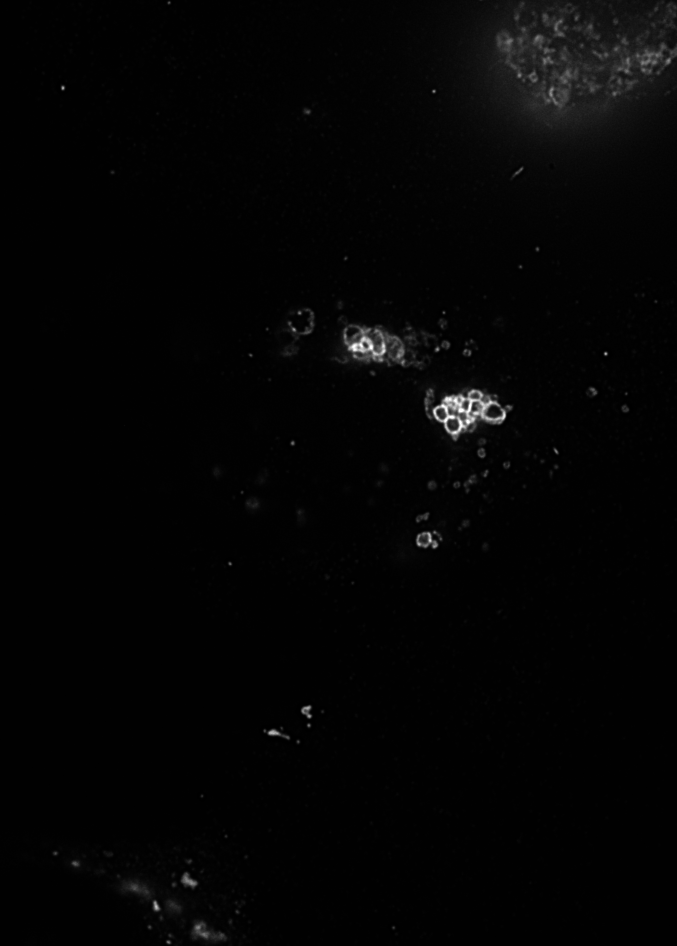

Supplement: Supplementary file 22 — Figure EV4 Source Data [file 44318_2026_705_MOESM22_ESM.zip › Figure EV3-2/F/STARD3FYAA_phospho_CHIR99021/20240523_MCF7STARD3FYAA_2G5r3144g_CHIR_3_SR_w2SPI 561 mCherry.TIF]

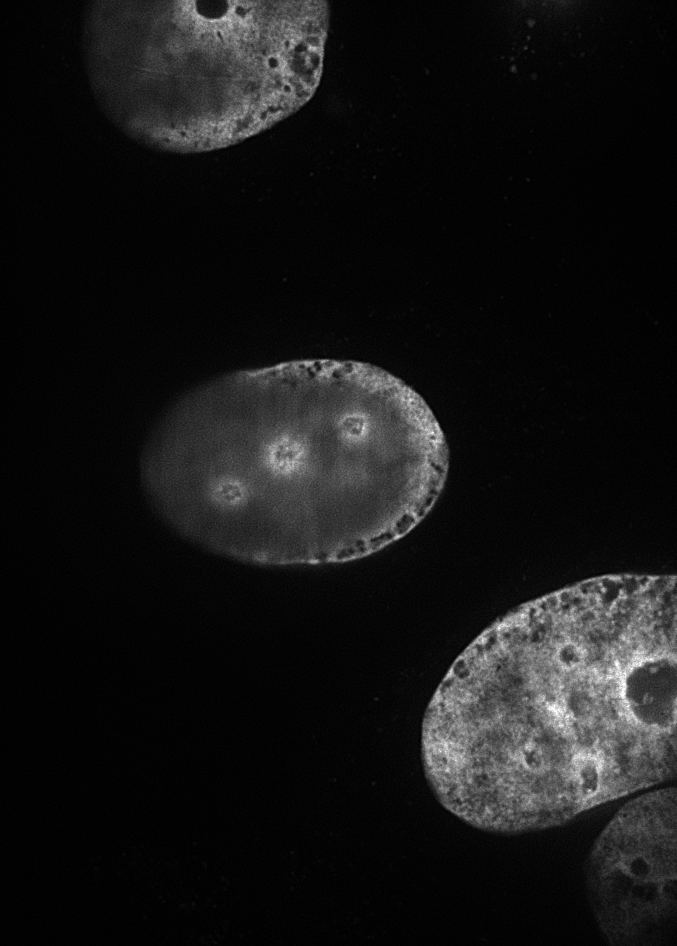

Supplement: Supplementary file 22 — Figure EV4 Source Data [file 44318_2026_705_MOESM22_ESM.zip › Figure EV3-2/F/STARD3FYAA_phospho_CHIR99021/20240523_MCF7STARD3FYAA_2G5r3144g_CHIR_3_SR_w3SPI 405 DAPI.TIF]

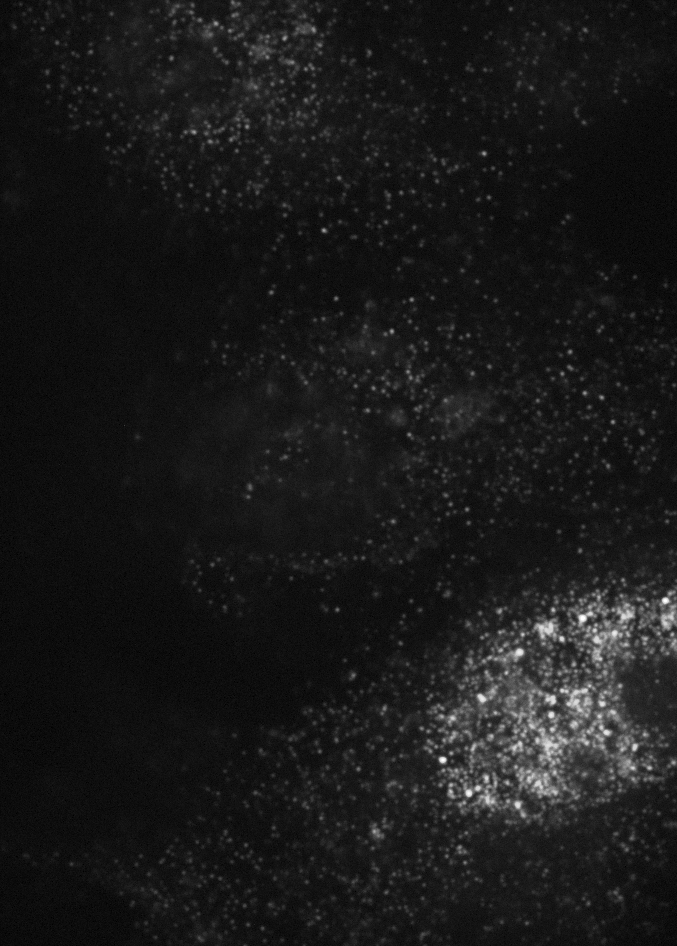

Supplement: Supplementary file 22 — Figure EV4 Source Data [file 44318_2026_705_MOESM22_ESM.zip › Figure EV3-2/F/STARD3FYAA_phospho_CHIR99021/20240523_MCF7STARD3FYAA_2G5r3144g_CHIR_3_w1SPI 491 GFP.TIF]

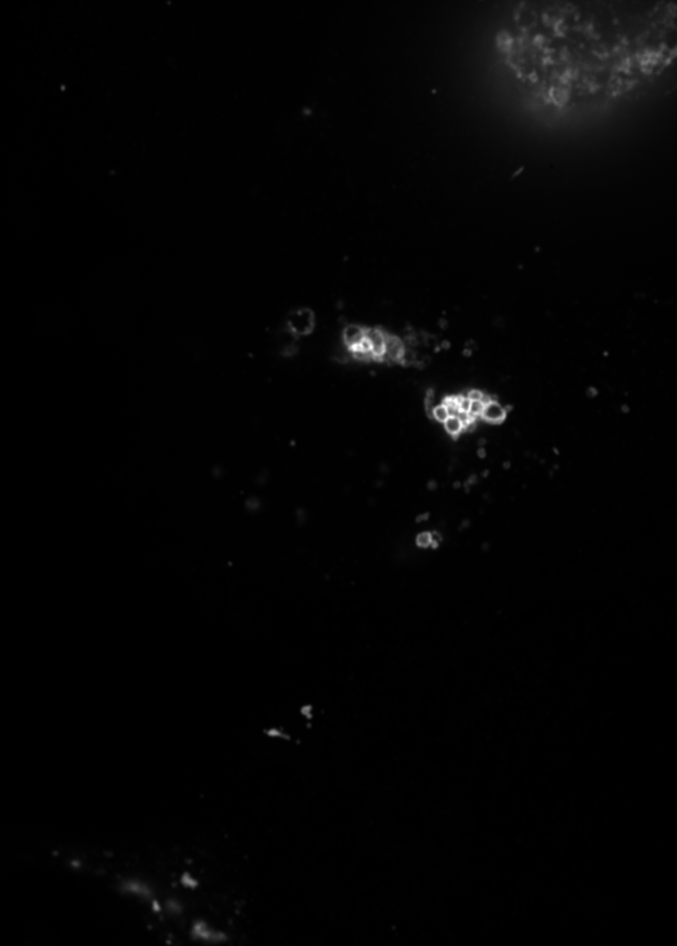

Supplement: Supplementary file 22 — Figure EV4 Source Data [file 44318_2026_705_MOESM22_ESM.zip › Figure EV3-2/F/STARD3FYAA_phospho_CHIR99021/20240523_MCF7STARD3FYAA_2G5r3144g_CHIR_3_w2SPI 561 mCherry.TIF]

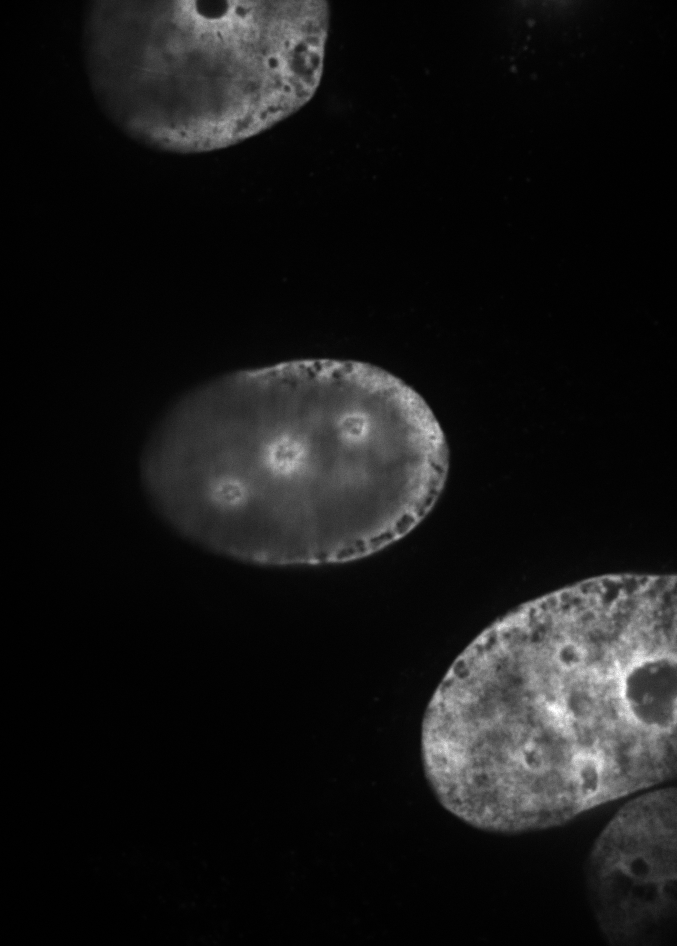

Supplement: Supplementary file 22 — Figure EV4 Source Data [file 44318_2026_705_MOESM22_ESM.zip › Figure EV3-2/F/STARD3FYAA_phospho_CHIR99021/20240523_MCF7STARD3FYAA_2G5r3144g_CHIR_3_w3SPI 405 DAPI.TIF]

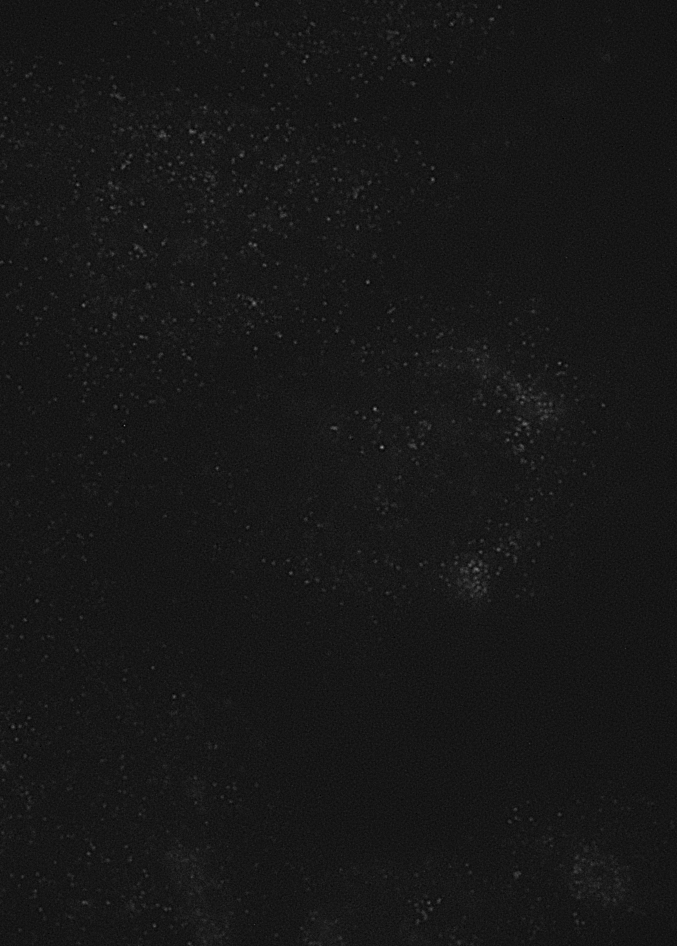

Supplement: Supplementary file 22 — Figure EV4 Source Data [file 44318_2026_705_MOESM22_ESM.zip › Figure EV3-2/F/STARD3FYAA_phospho_NT/20240523_MCF7STARD3FYAA_2G5r3144g_NT_1_SR_w1SPI 491 GFP.TIF]

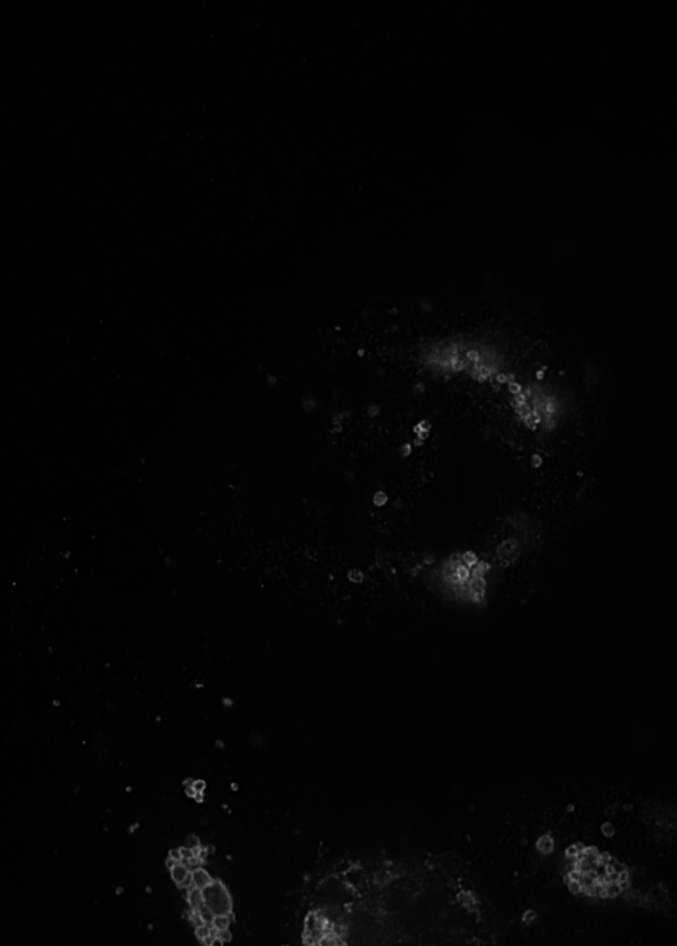

Supplement: Supplementary file 22 — Figure EV4 Source Data [file 44318_2026_705_MOESM22_ESM.zip › Figure EV3-2/F/STARD3FYAA_phospho_NT/20240523_MCF7STARD3FYAA_2G5r3144g_NT_1_SR_w2SPI 561 mCherry.TIF]

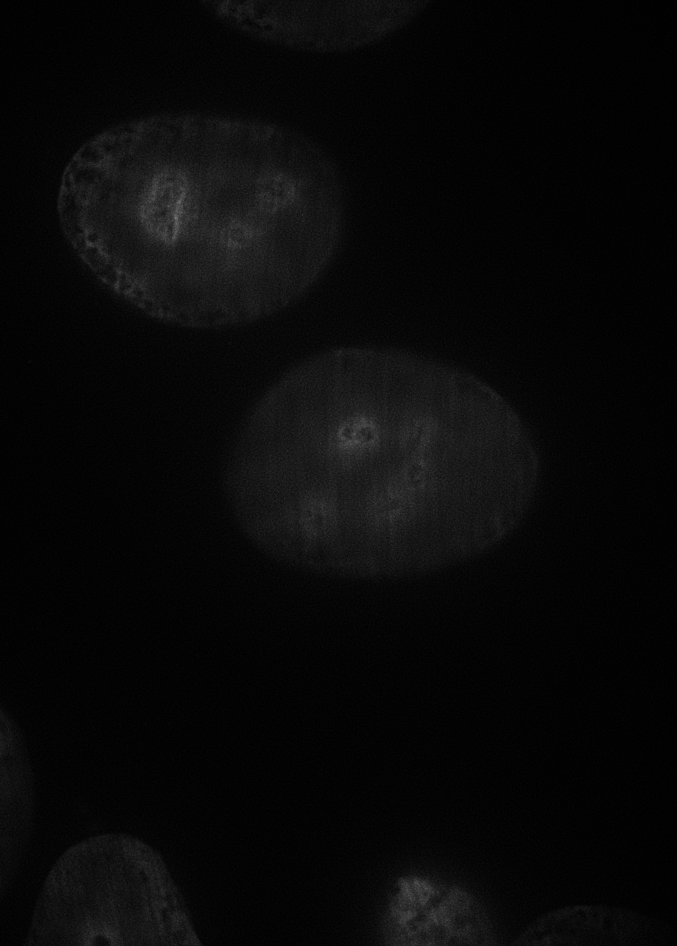

Supplement: Supplementary file 22 — Figure EV4 Source Data [file 44318_2026_705_MOESM22_ESM.zip › Figure EV3-2/F/STARD3FYAA_phospho_NT/20240523_MCF7STARD3FYAA_2G5r3144g_NT_1_SR_w3SPI 405 DAPI.TIF]

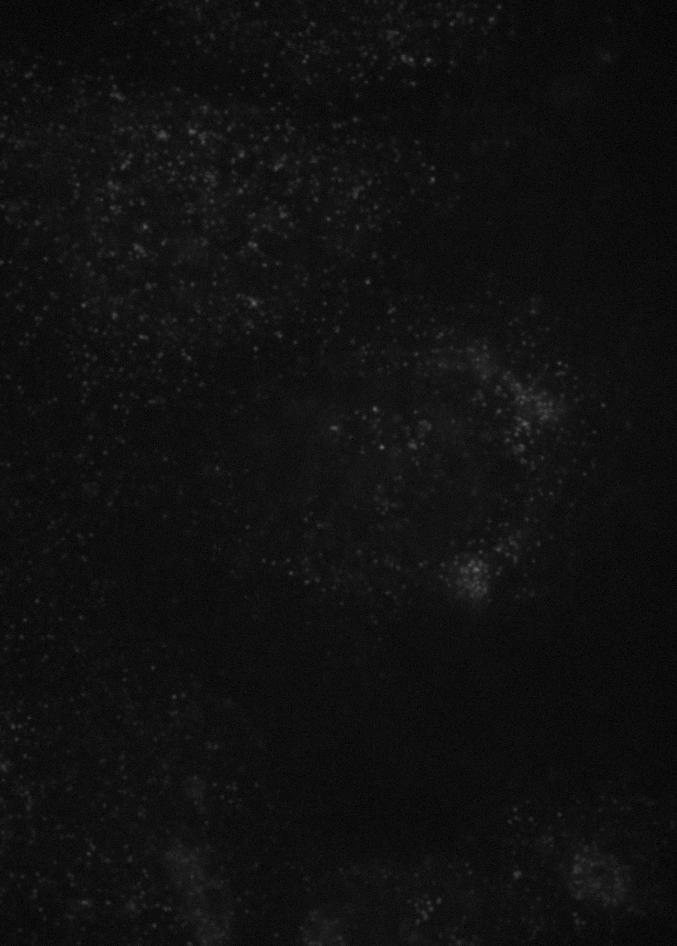

Supplement: Supplementary file 22 — Figure EV4 Source Data [file 44318_2026_705_MOESM22_ESM.zip › Figure EV3-2/F/STARD3FYAA_phospho_NT/20240523_MCF7STARD3FYAA_2G5r3144g_NT_1_w1SPI 491 GFP.TIF]

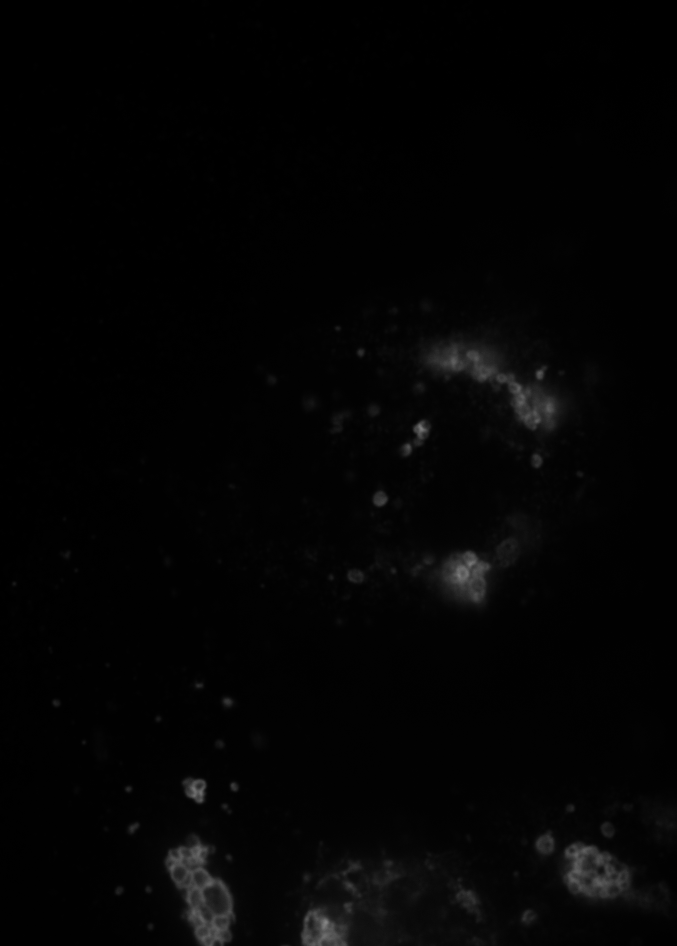

Supplement: Supplementary file 22 — Figure EV4 Source Data [file 44318_2026_705_MOESM22_ESM.zip › Figure EV3-2/F/STARD3FYAA_phospho_NT/20240523_MCF7STARD3FYAA_2G5r3144g_NT_1_w2SPI 561 mCherry.TIF]

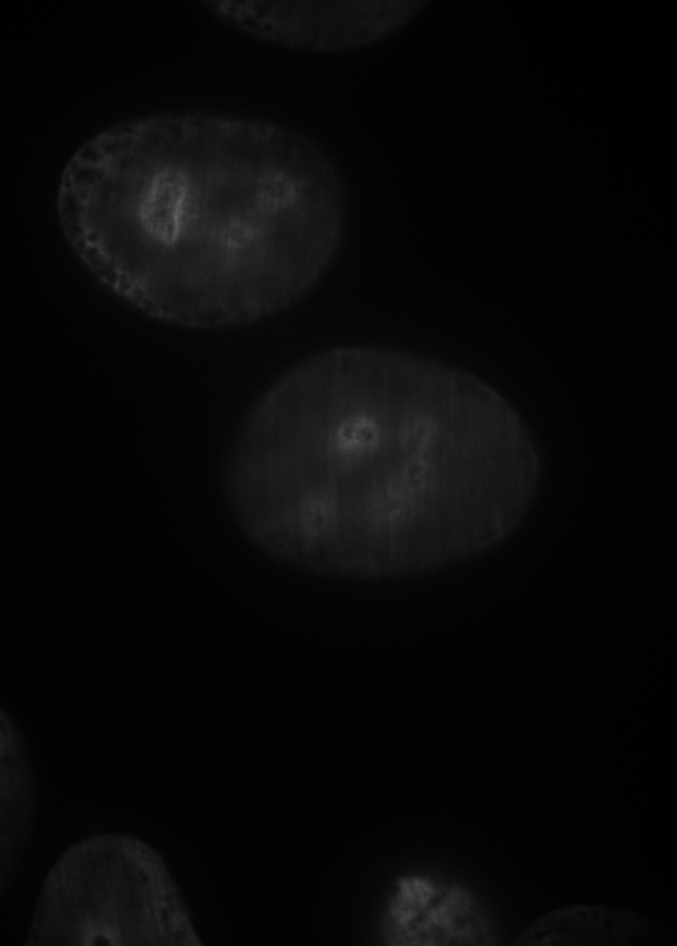

Supplement: Supplementary file 22 — Figure EV4 Source Data [file 44318_2026_705_MOESM22_ESM.zip › Figure EV3-2/F/STARD3FYAA_phospho_NT/20240523_MCF7STARD3FYAA_2G5r3144g_NT_1_w3SPI 405 DAPI.TIF]

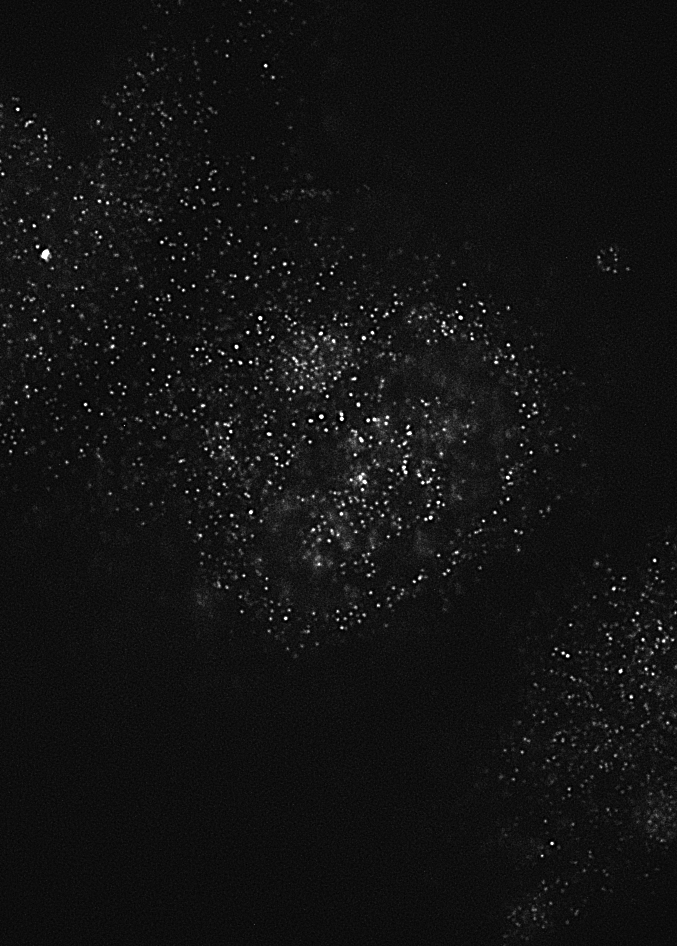

Supplement: Supplementary file 22 — Figure EV4 Source Data [file 44318_2026_705_MOESM22_ESM.zip › Figure EV3-2/G/STARD3deltaFFAT_phospho_CHIR99021/20240524_MCF7STARD3deltaFFAT_2G5r3144g_CHIR_1_SR_w1SPI 491 GFP.TIF]

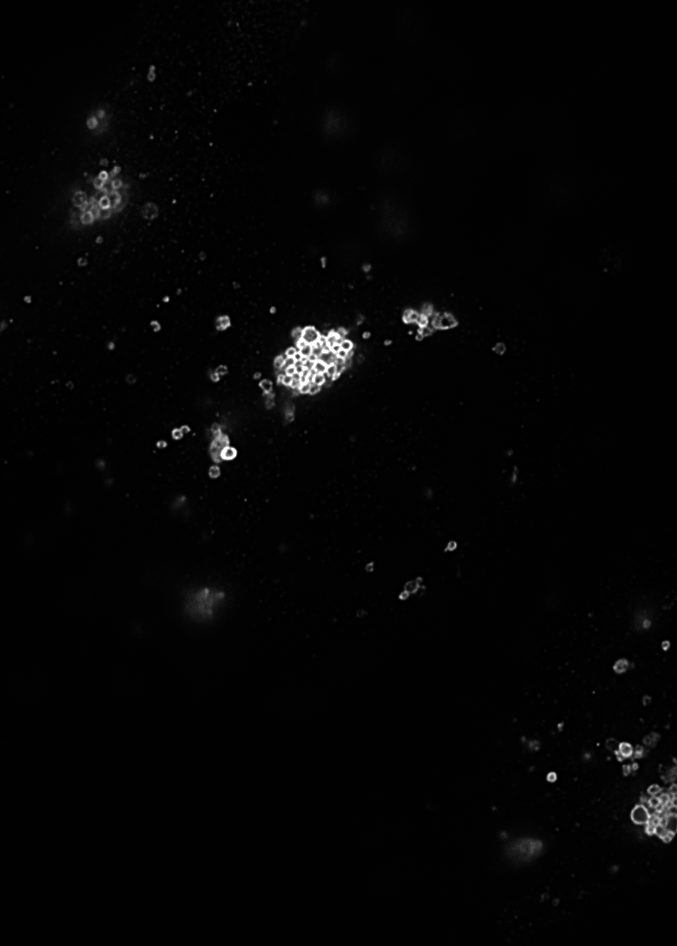

Supplement: Supplementary file 22 — Figure EV4 Source Data [file 44318_2026_705_MOESM22_ESM.zip › Figure EV3-2/G/STARD3deltaFFAT_phospho_CHIR99021/20240524_MCF7STARD3deltaFFAT_2G5r3144g_CHIR_1_SR_w2SPI 561 mCherry.TIF]

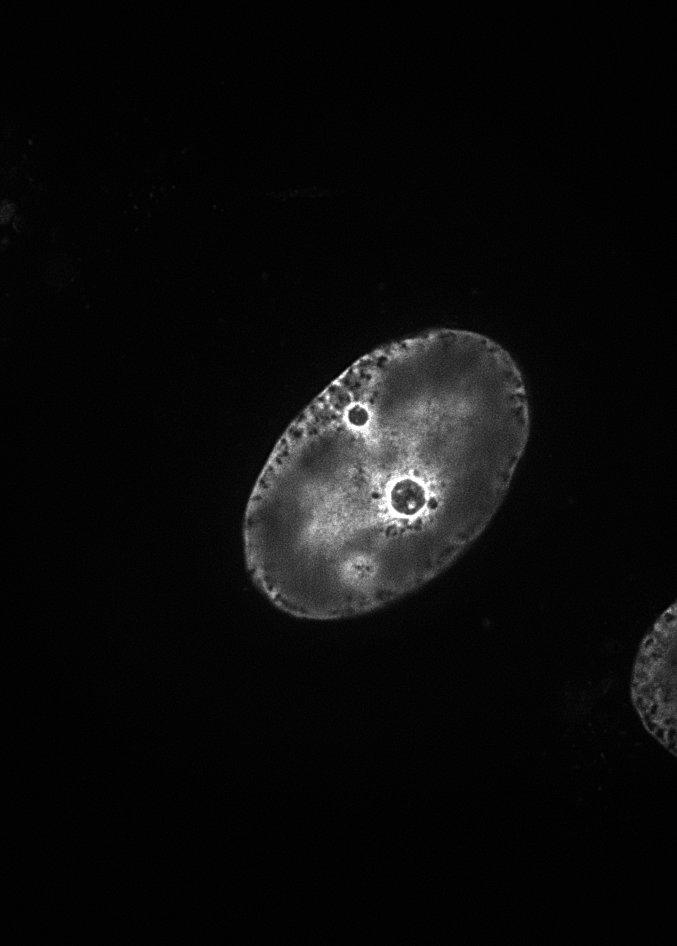

Supplement: Supplementary file 22 — Figure EV4 Source Data [file 44318_2026_705_MOESM22_ESM.zip › Figure EV3-2/G/STARD3deltaFFAT_phospho_CHIR99021/20240524_MCF7STARD3deltaFFAT_2G5r3144g_CHIR_1_SR_w3SPI 405 DAPI.TIF]

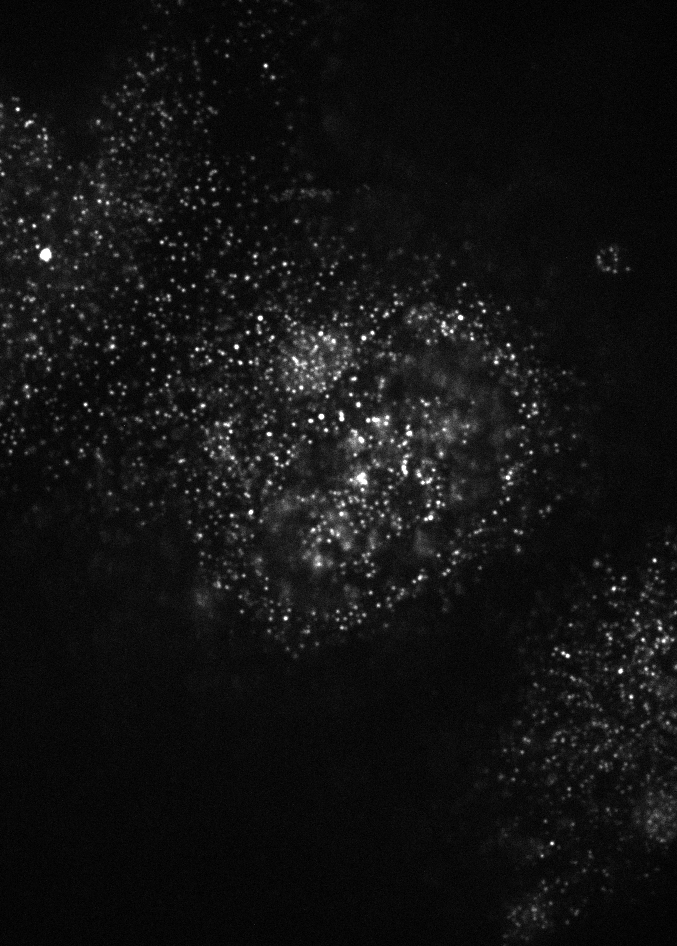

Supplement: Supplementary file 22 — Figure EV4 Source Data [file 44318_2026_705_MOESM22_ESM.zip › Figure EV3-2/G/STARD3deltaFFAT_phospho_CHIR99021/20240524_MCF7STARD3deltaFFAT_2G5r3144g_CHIR_1_w1SPI 491 GFP.TIF]

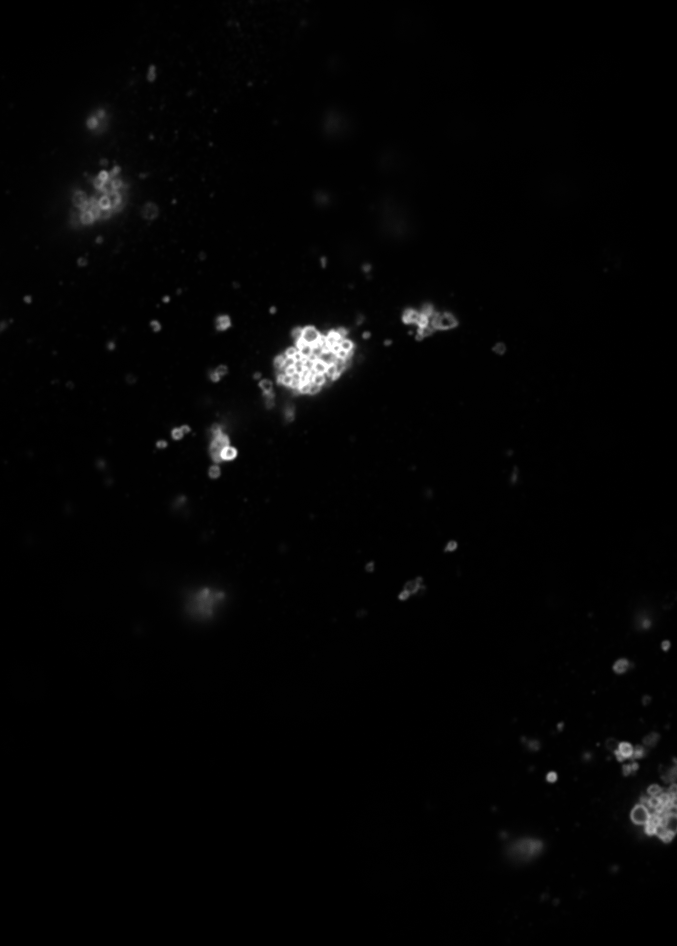

Supplement: Supplementary file 22 — Figure EV4 Source Data [file 44318_2026_705_MOESM22_ESM.zip › Figure EV3-2/G/STARD3deltaFFAT_phospho_CHIR99021/20240524_MCF7STARD3deltaFFAT_2G5r3144g_CHIR_1_w2SPI 561 mCherry.TIF]

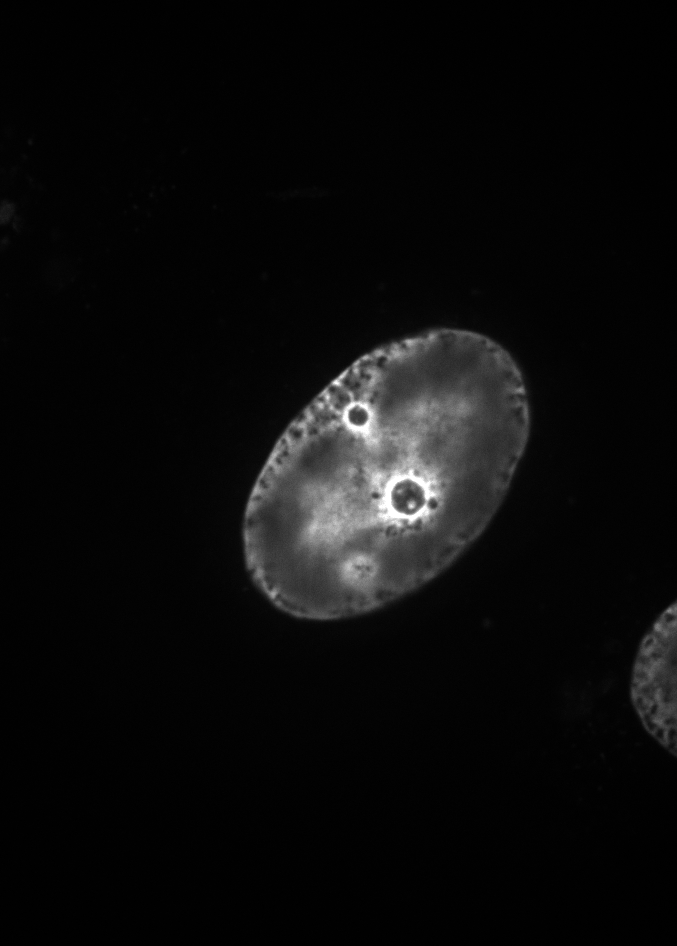

Supplement: Supplementary file 22 — Figure EV4 Source Data [file 44318_2026_705_MOESM22_ESM.zip › Figure EV3-2/G/STARD3deltaFFAT_phospho_CHIR99021/20240524_MCF7STARD3deltaFFAT_2G5r3144g_CHIR_1_w3SPI 405 DAPI.TIF]

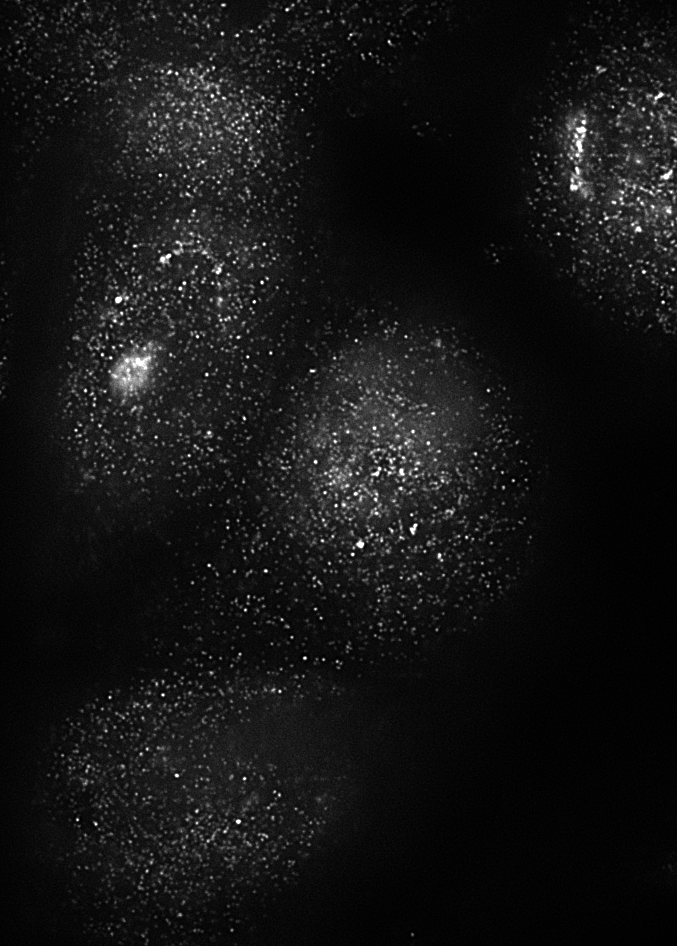

Supplement: Supplementary file 22 — Figure EV4 Source Data [file 44318_2026_705_MOESM22_ESM.zip › Figure EV3-2/G/STARD3deltaFFAT_phospho_NT/20240521_MCF7STARD3deltaFFAT_2G5r3144g_NT_3_SR_w1SPI 491 GFP.TIF]

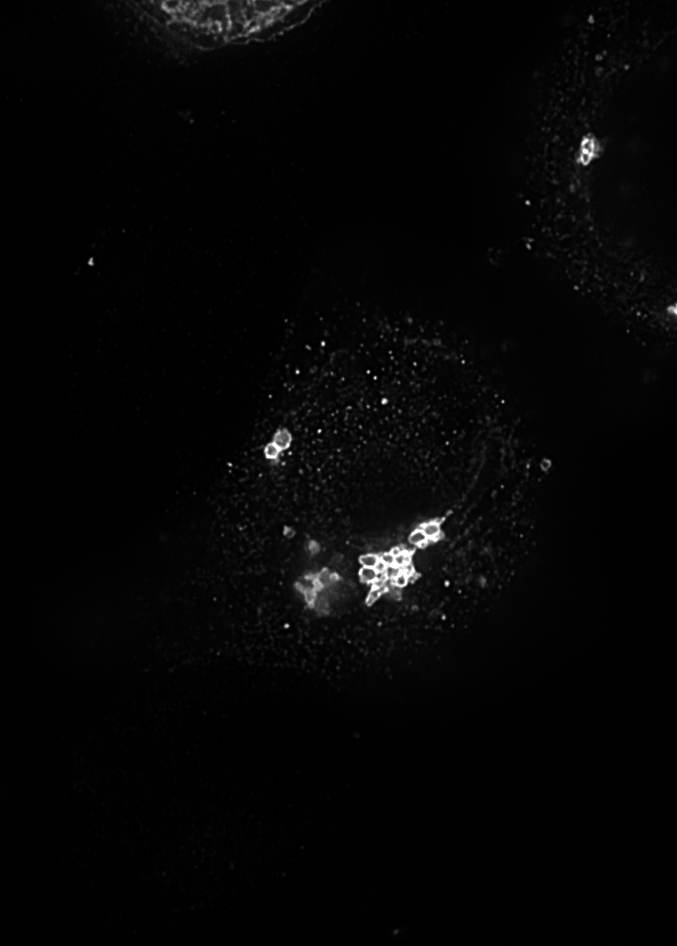

Supplement: Supplementary file 22 — Figure EV4 Source Data [file 44318_2026_705_MOESM22_ESM.zip › Figure EV3-2/G/STARD3deltaFFAT_phospho_NT/20240521_MCF7STARD3deltaFFAT_2G5r3144g_NT_3_SR_w2SPI 561 mCherry.TIF]

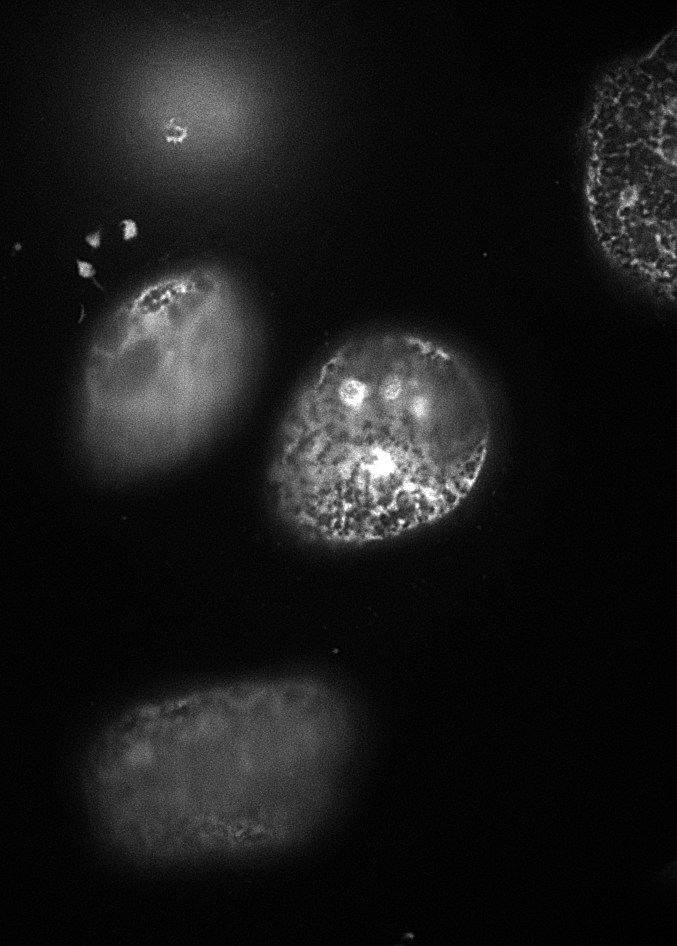

Supplement: Supplementary file 22 — Figure EV4 Source Data [file 44318_2026_705_MOESM22_ESM.zip › Figure EV3-2/G/STARD3deltaFFAT_phospho_NT/20240521_MCF7STARD3deltaFFAT_2G5r3144g_NT_3_SR_w3SPI 405 DAPI.TIF]

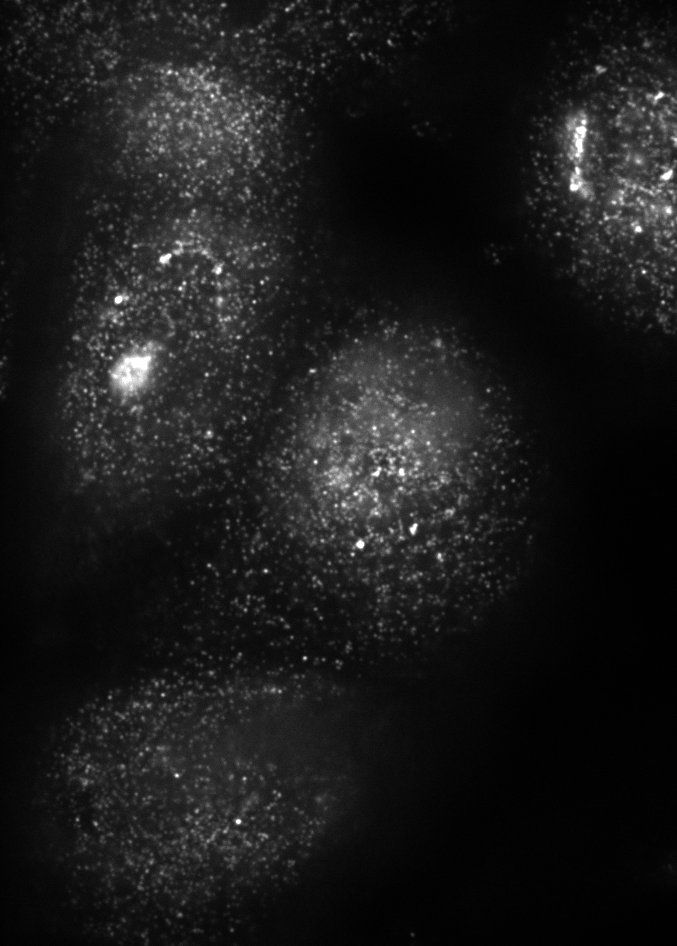

Supplement: Supplementary file 22 — Figure EV4 Source Data [file 44318_2026_705_MOESM22_ESM.zip › Figure EV3-2/G/STARD3deltaFFAT_phospho_NT/20240521_MCF7STARD3deltaFFAT_2G5r3144g_NT_3_w1SPI 491 GFP.TIF]

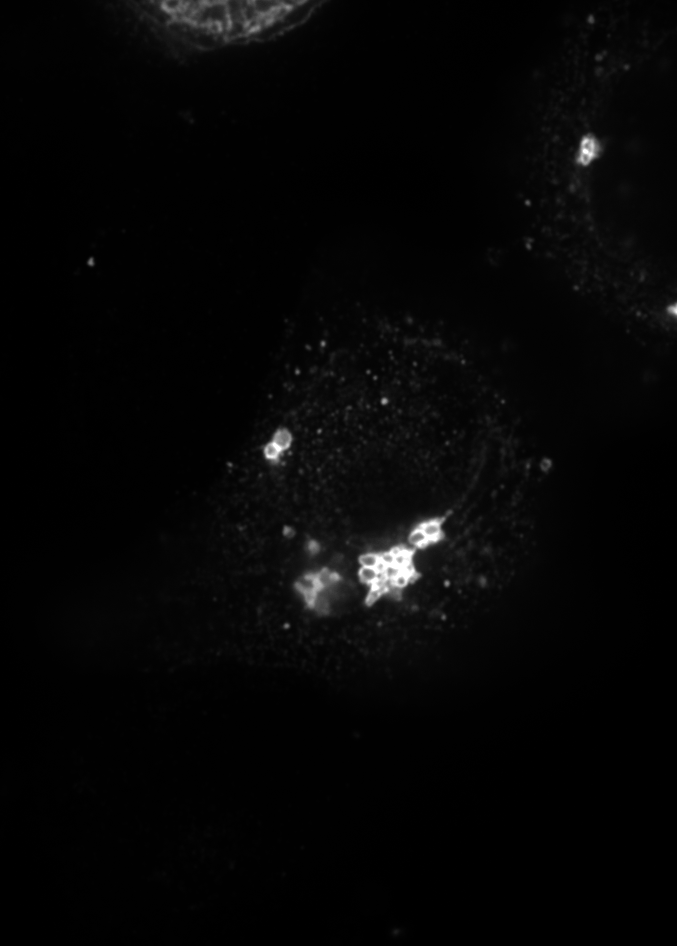

Supplement: Supplementary file 22 — Figure EV4 Source Data [file 44318_2026_705_MOESM22_ESM.zip › Figure EV3-2/G/STARD3deltaFFAT_phospho_NT/20240521_MCF7STARD3deltaFFAT_2G5r3144g_NT_3_w2SPI 561 mCherry.TIF]

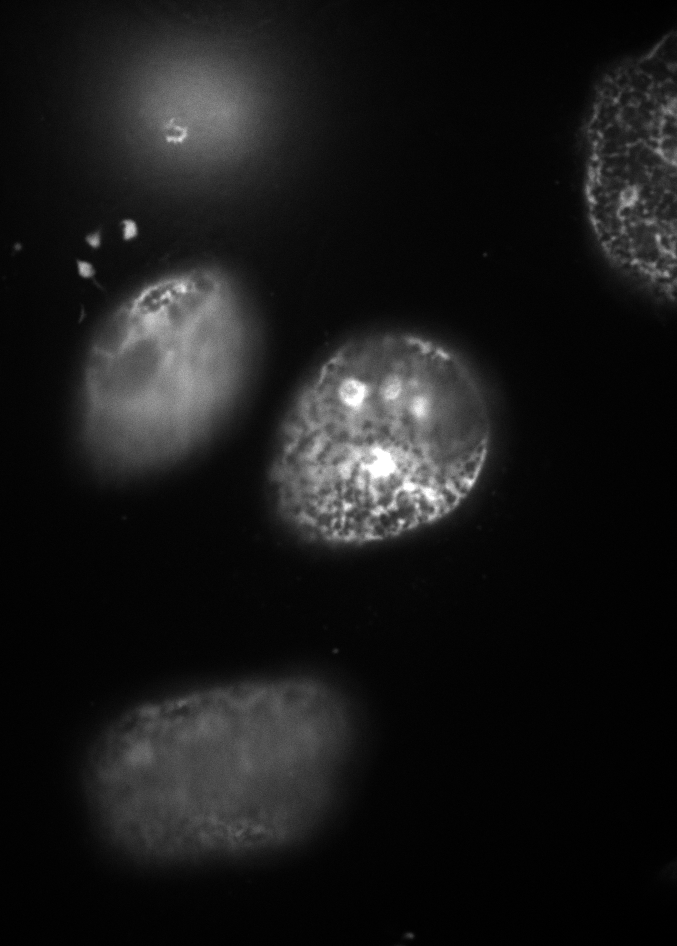

Supplement: Supplementary file 22 — Figure EV4 Source Data [file 44318_2026_705_MOESM22_ESM.zip › Figure EV3-2/G/STARD3deltaFFAT_phospho_NT/20240521_MCF7STARD3deltaFFAT_2G5r3144g_NT_3_w3SPI 405 DAPI.TIF]

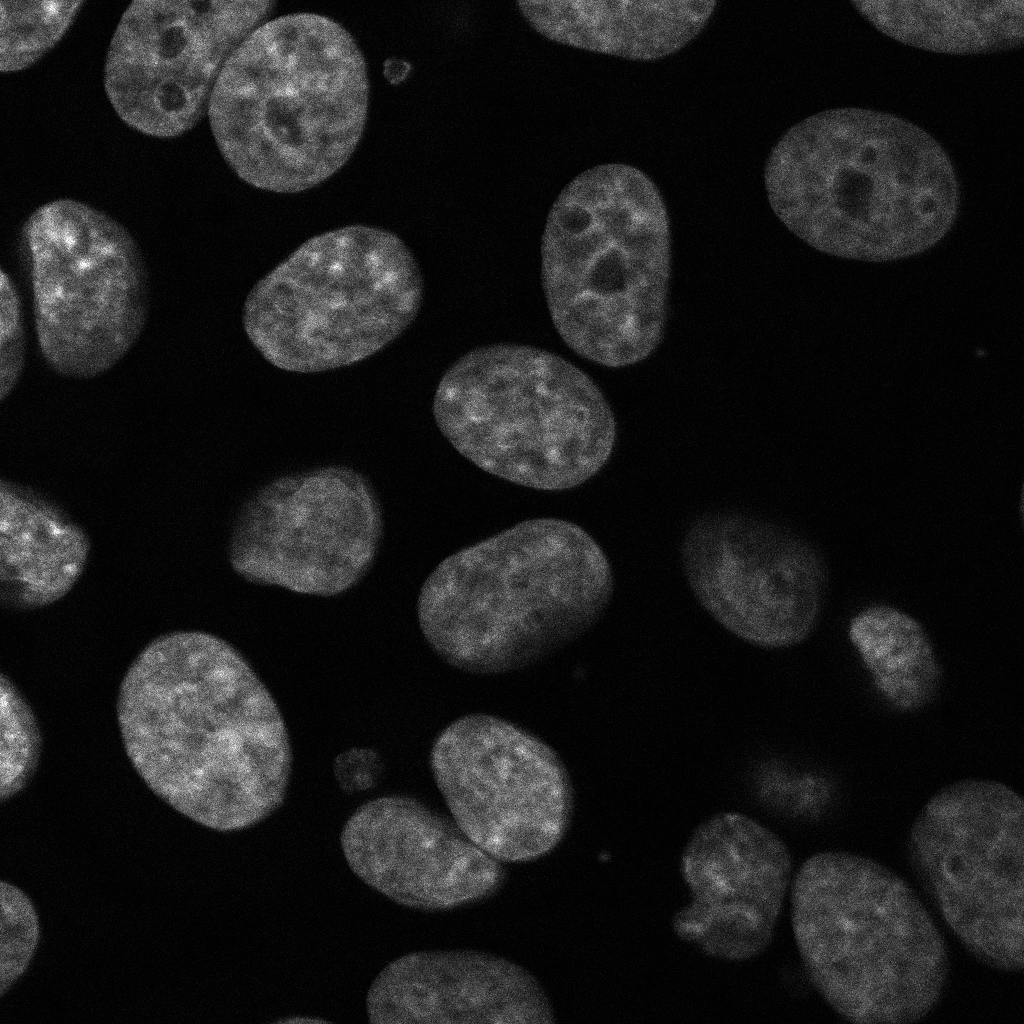

Supplement: Supplementary file 23 — Figure EV4-1 Source Data [file 44318_2026_705_MOESM23_ESM.zip › Figure EV4-1/A/HeLa_CHIR99021/C1-20211022_loc_endo_STARD3_CHIR.tif]

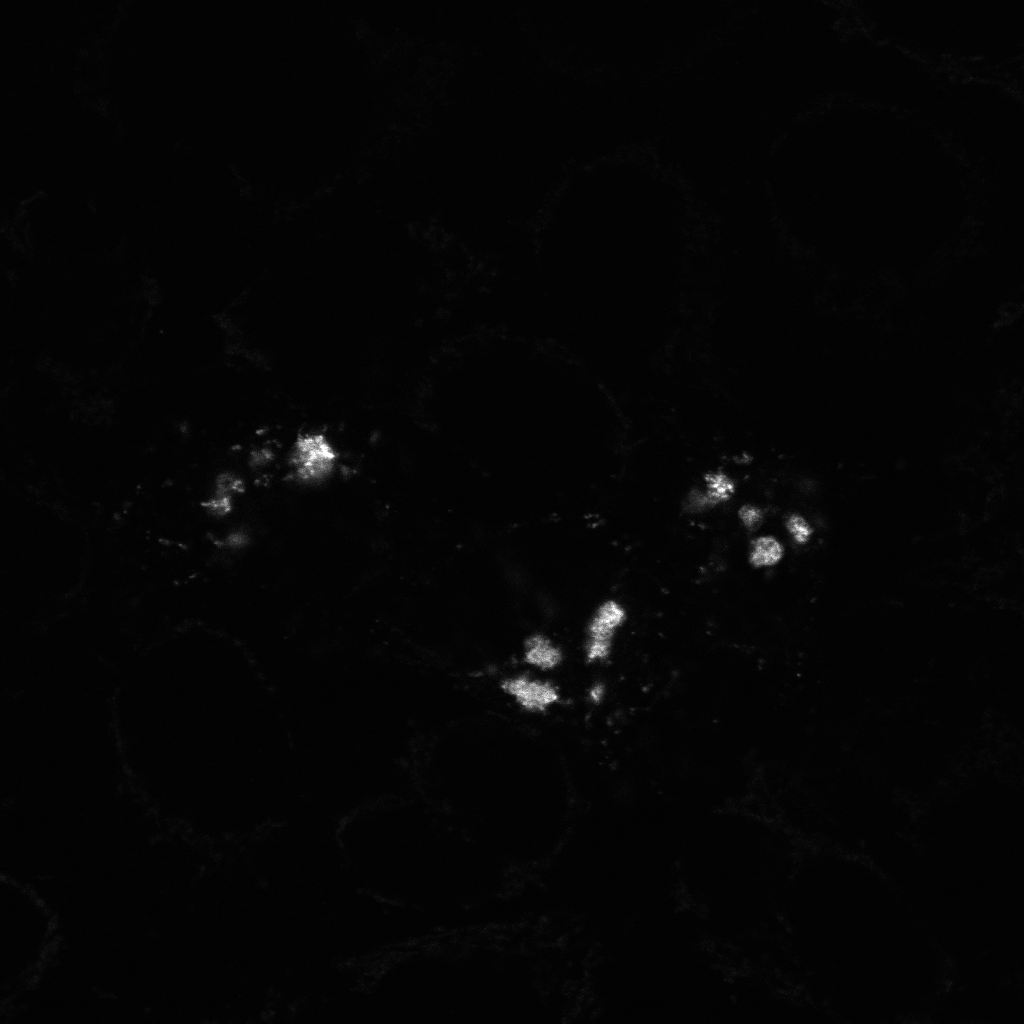

Supplement: Supplementary file 23 — Figure EV4-1 Source Data [file 44318_2026_705_MOESM23_ESM.zip › Figure EV4-1/A/HeLa_CHIR99021/C2-20211022_loc_endo_STARD3_CHIR.tif]

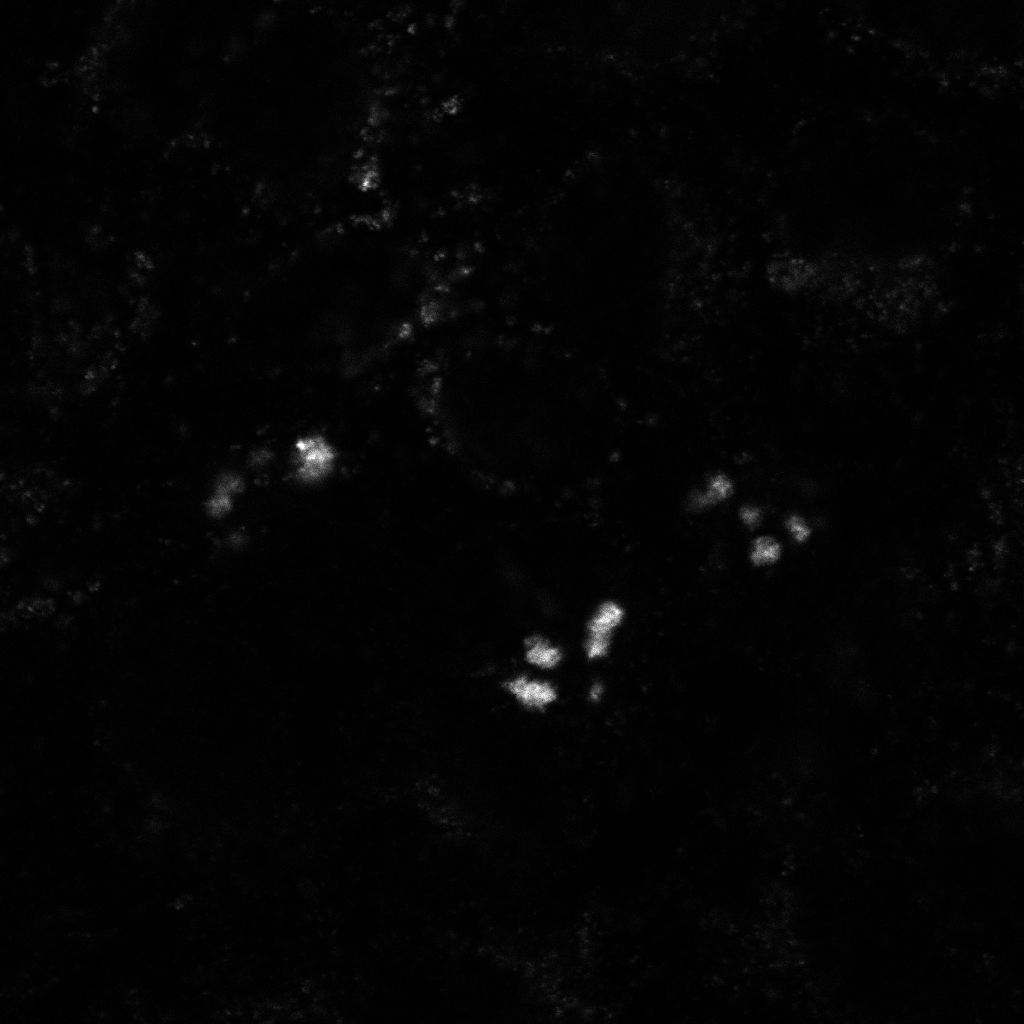

Supplement: Supplementary file 23 — Figure EV4-1 Source Data [file 44318_2026_705_MOESM23_ESM.zip › Figure EV4-1/A/HeLa_CHIR99021/C3-20211022_loc_endo_STARD3_CHIR.tif]

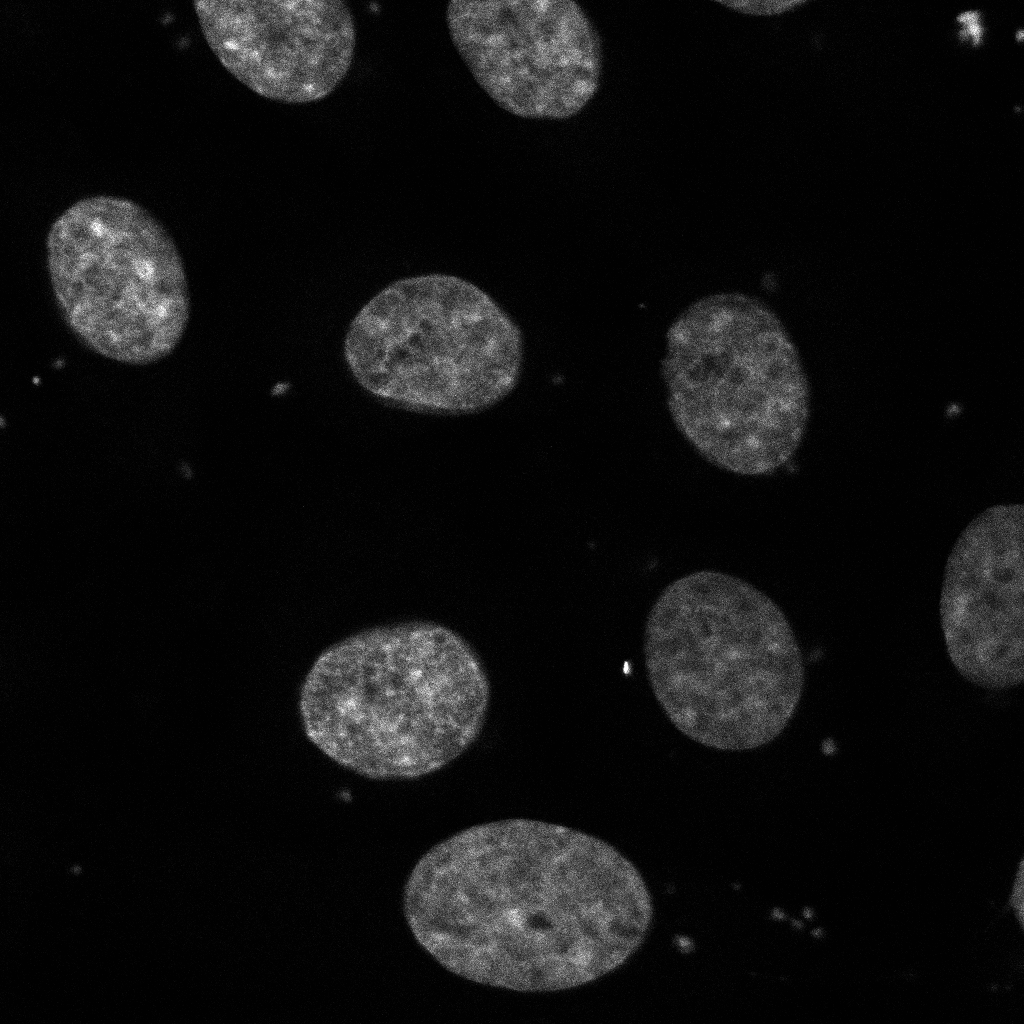

Supplement: Supplementary file 23 — Figure EV4-1 Source Data [file 44318_2026_705_MOESM23_ESM.zip › Figure EV4-1/A/HeLa_NT/C1-20211022_loc_endo_STARD3_WT_NT.tif]

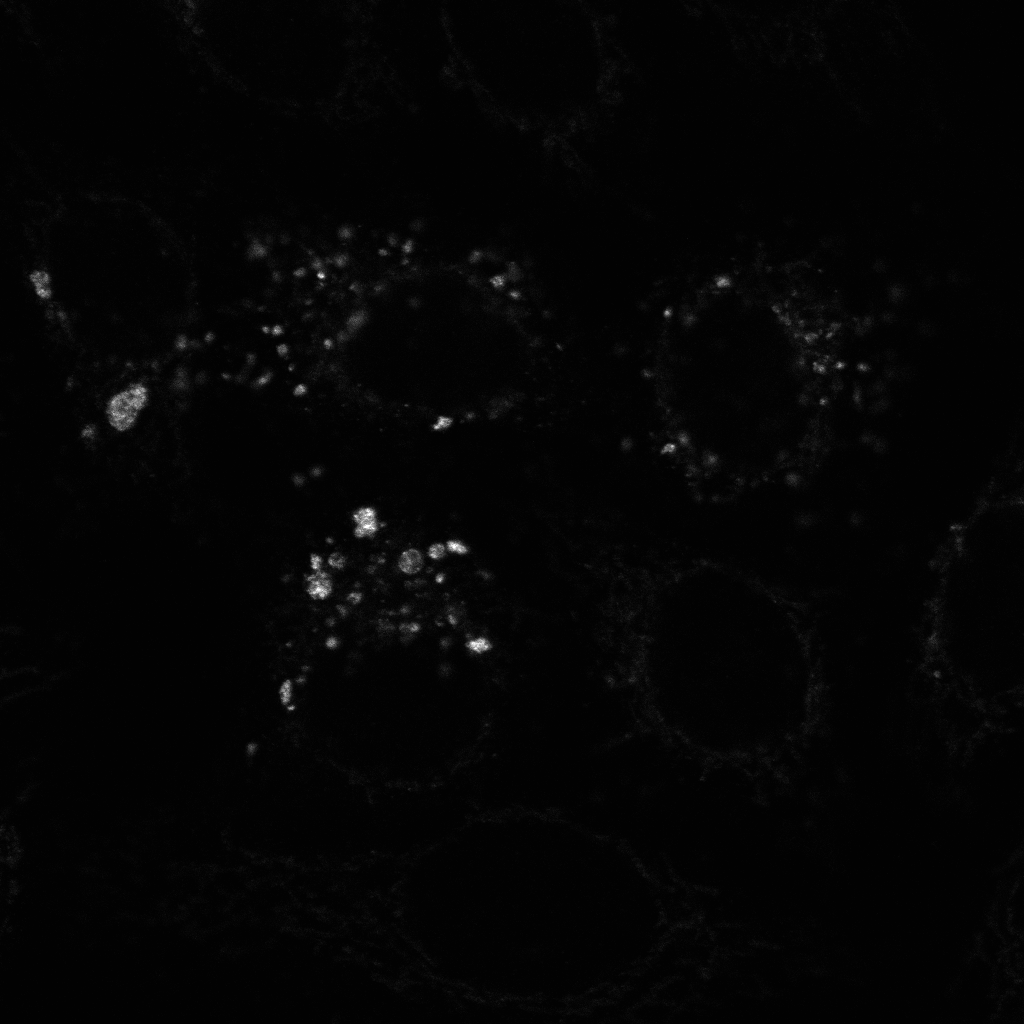

Supplement: Supplementary file 23 — Figure EV4-1 Source Data [file 44318_2026_705_MOESM23_ESM.zip › Figure EV4-1/A/HeLa_NT/C2-20211022_loc_endo_STARD3_WT_NT.tif]

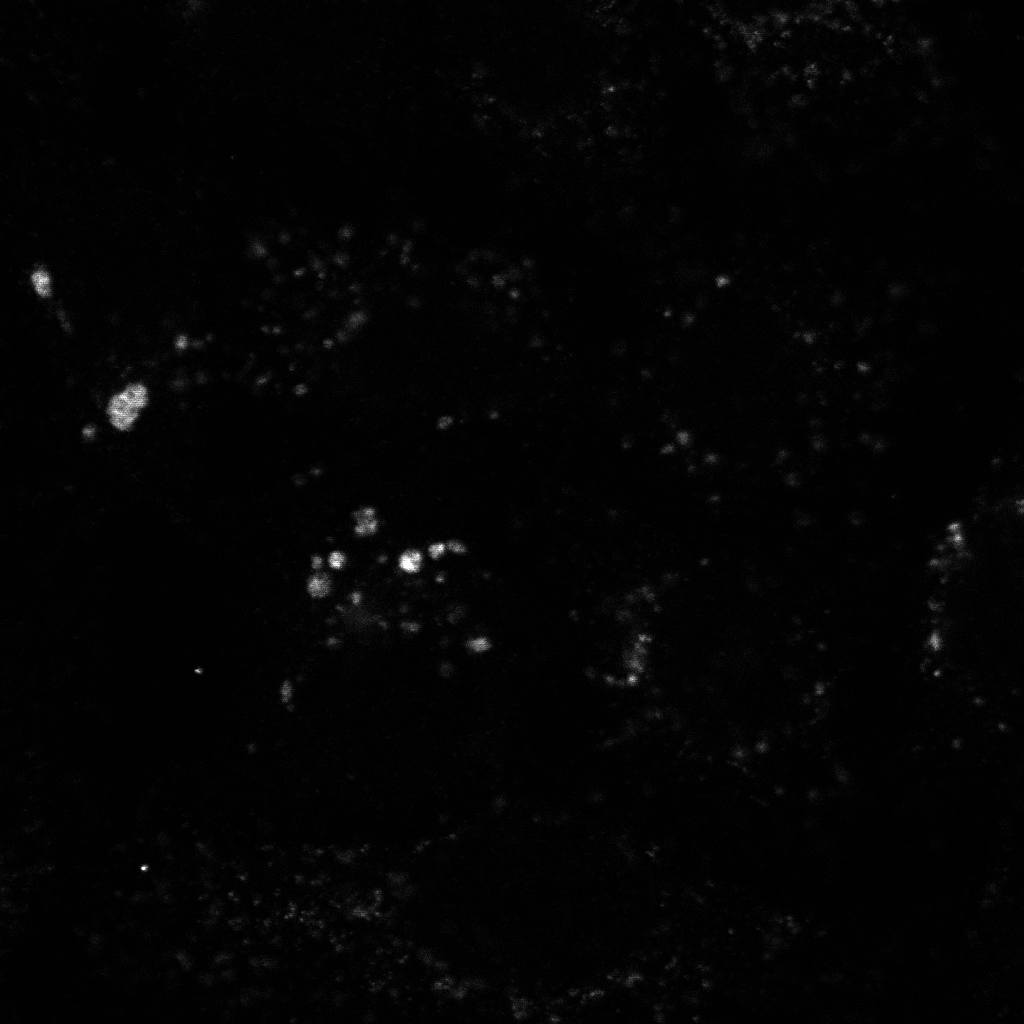

Supplement: Supplementary file 23 — Figure EV4-1 Source Data [file 44318_2026_705_MOESM23_ESM.zip › Figure EV4-1/A/HeLa_NT/C3-20211022_loc_endo_STARD3_WT_NT.tif]

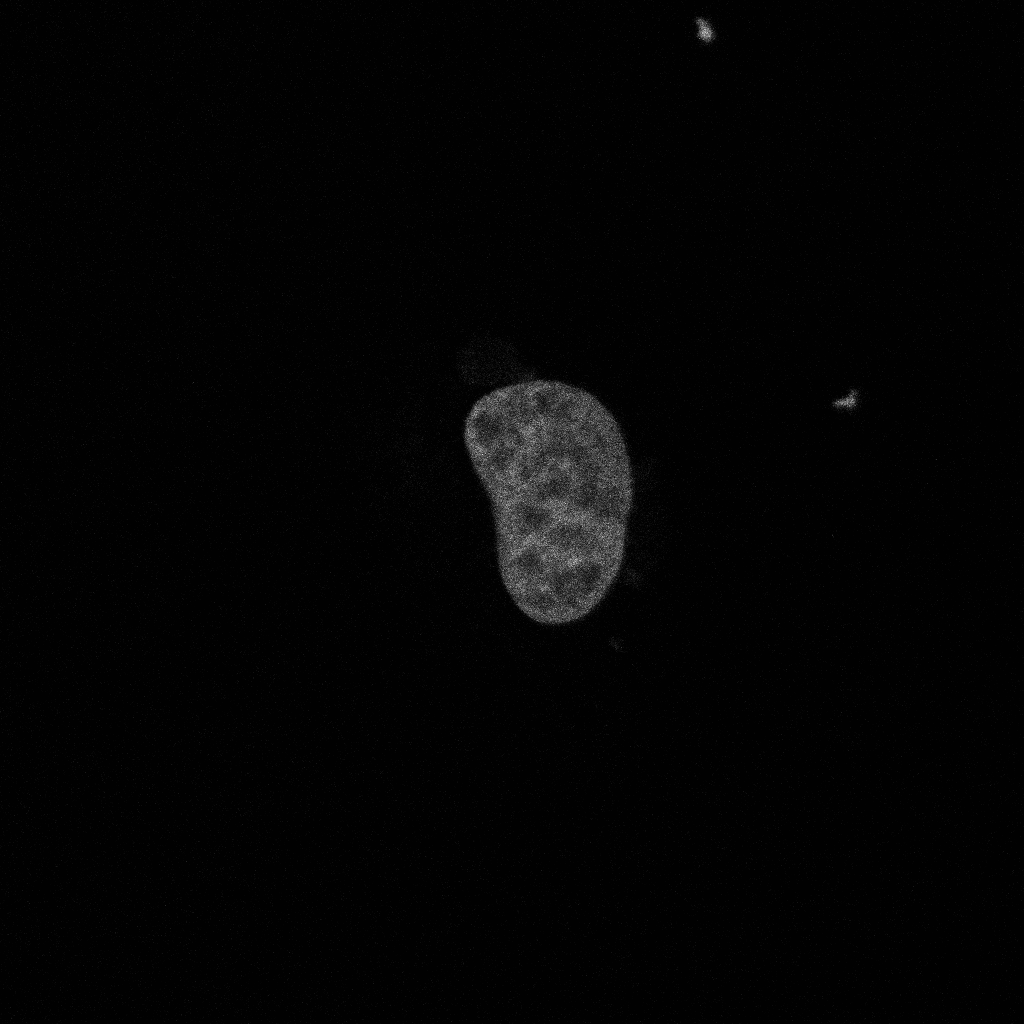

Supplement: Supplementary file 23 — Figure EV4-1 Source Data [file 44318_2026_705_MOESM23_ESM.zip › Figure EV4-1/B/U2OS_CHIR99021/C1-U2OS_STARD3_CHIR.tif]

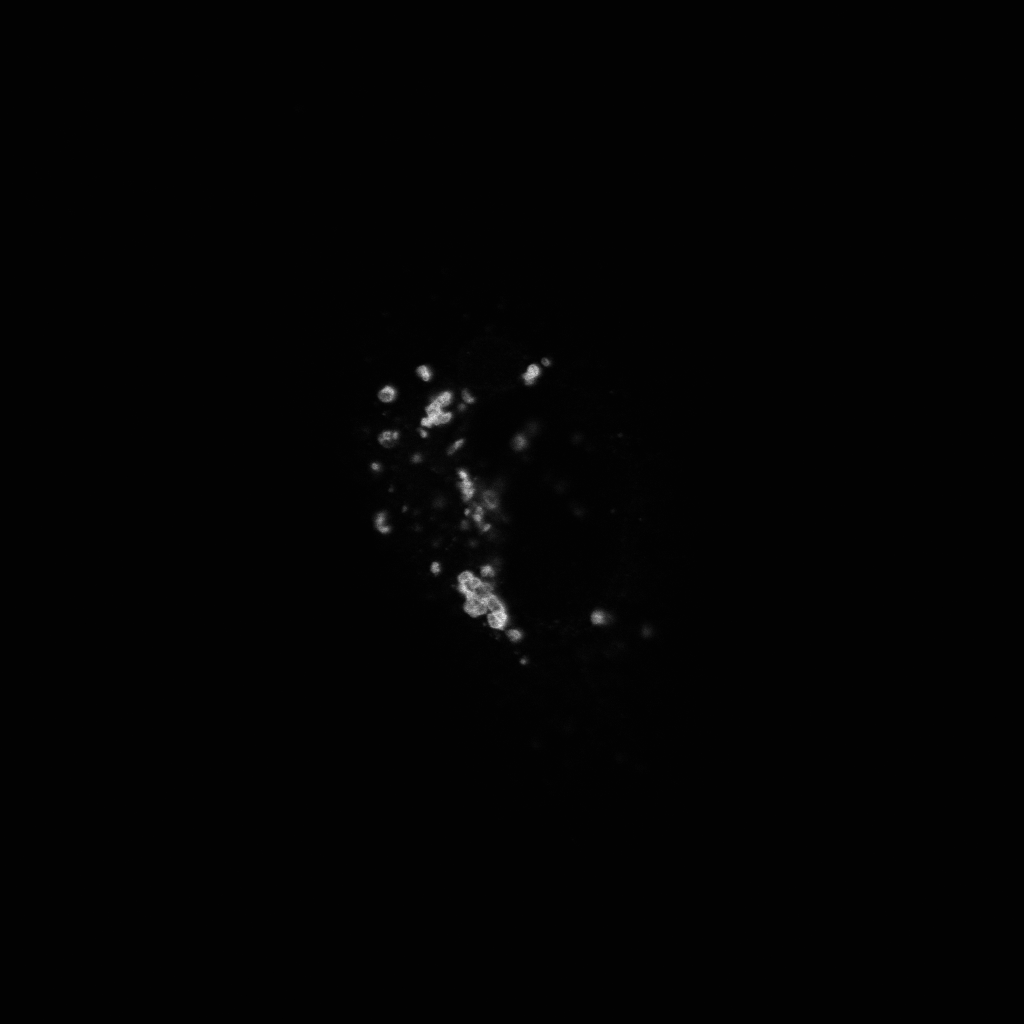

Supplement: Supplementary file 23 — Figure EV4-1 Source Data [file 44318_2026_705_MOESM23_ESM.zip › Figure EV4-1/B/U2OS_CHIR99021/C2-U2OS_STARD3_CHIR.tif]

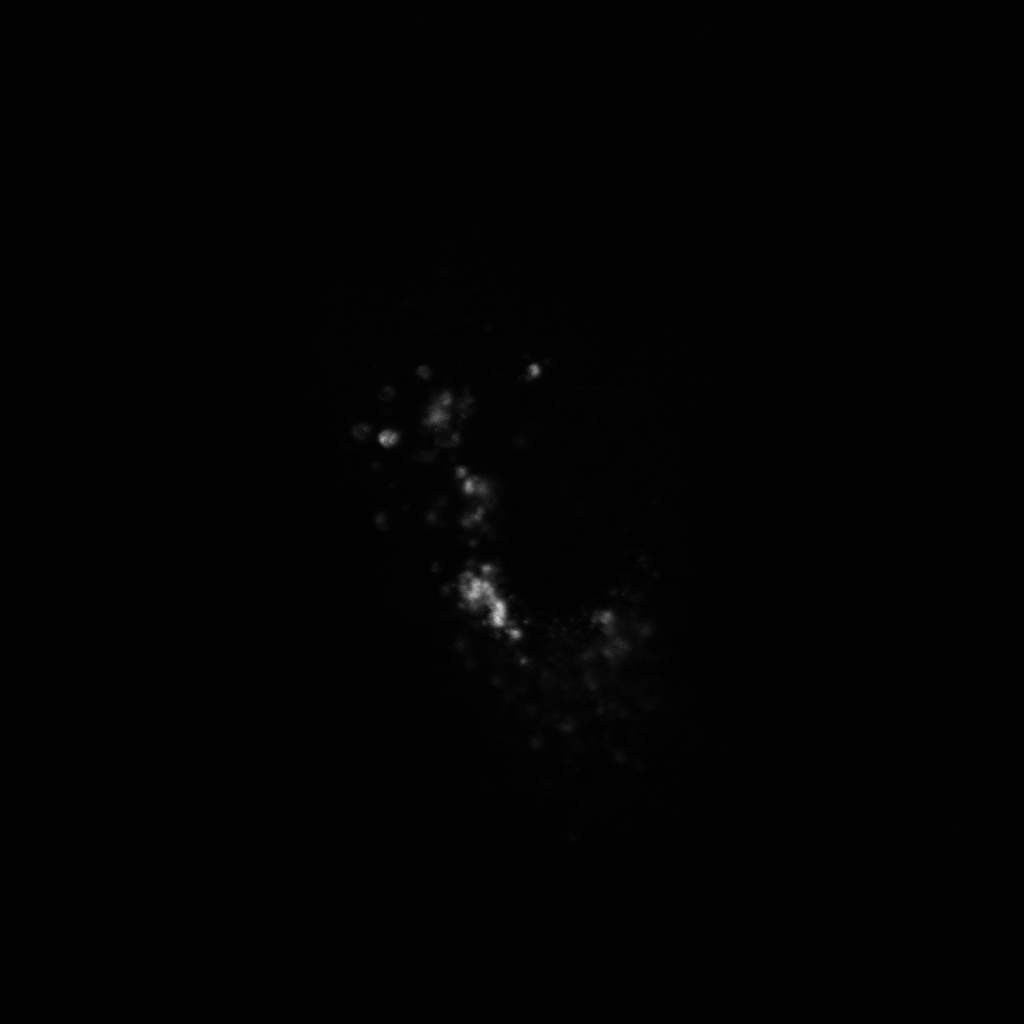

Supplement: Supplementary file 23 — Figure EV4-1 Source Data [file 44318_2026_705_MOESM23_ESM.zip › Figure EV4-1/B/U2OS_CHIR99021/C3-U2OS_STARD3_CHIR.tif]

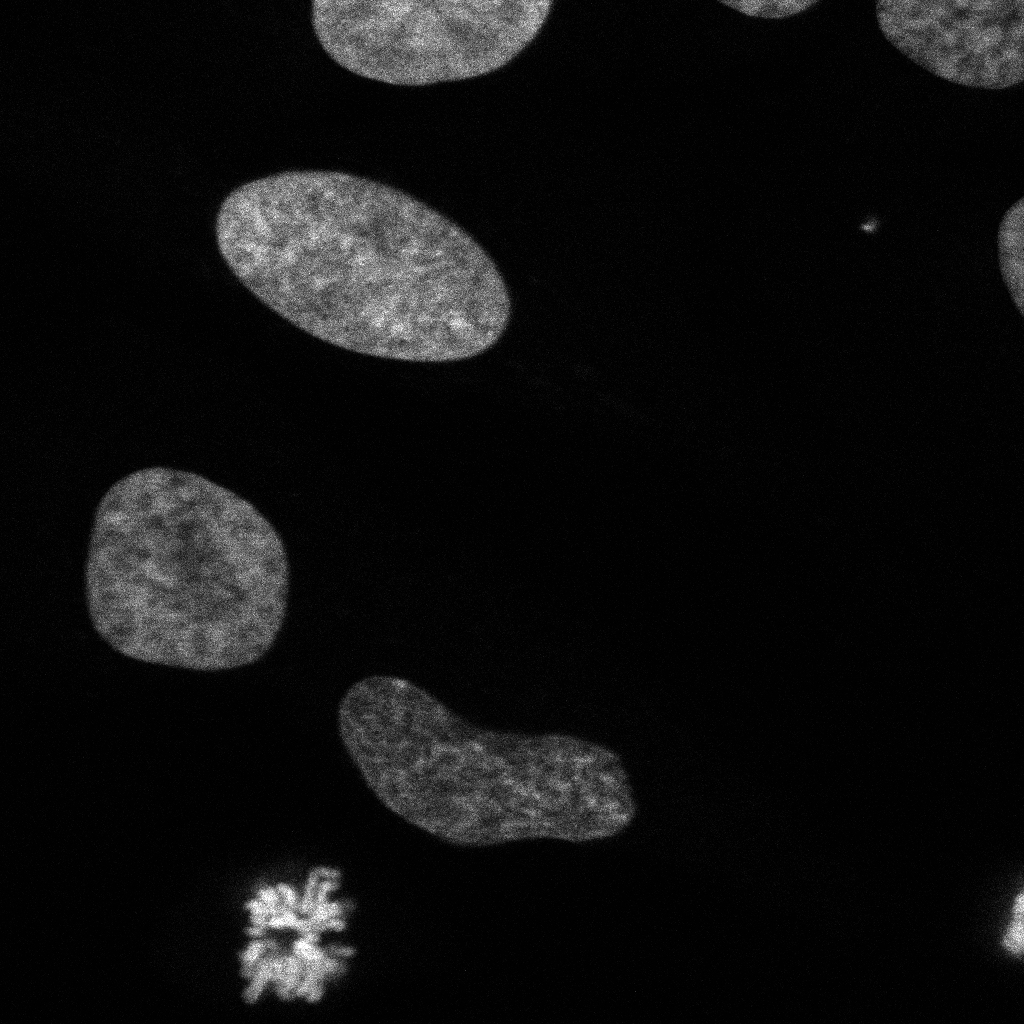

Supplement: Supplementary file 23 — Figure EV4-1 Source Data [file 44318_2026_705_MOESM23_ESM.zip › Figure EV4-1/B/U2OS_NT/C1-20211105_loc_STARD3.lif - U2OSWT_1.tif]

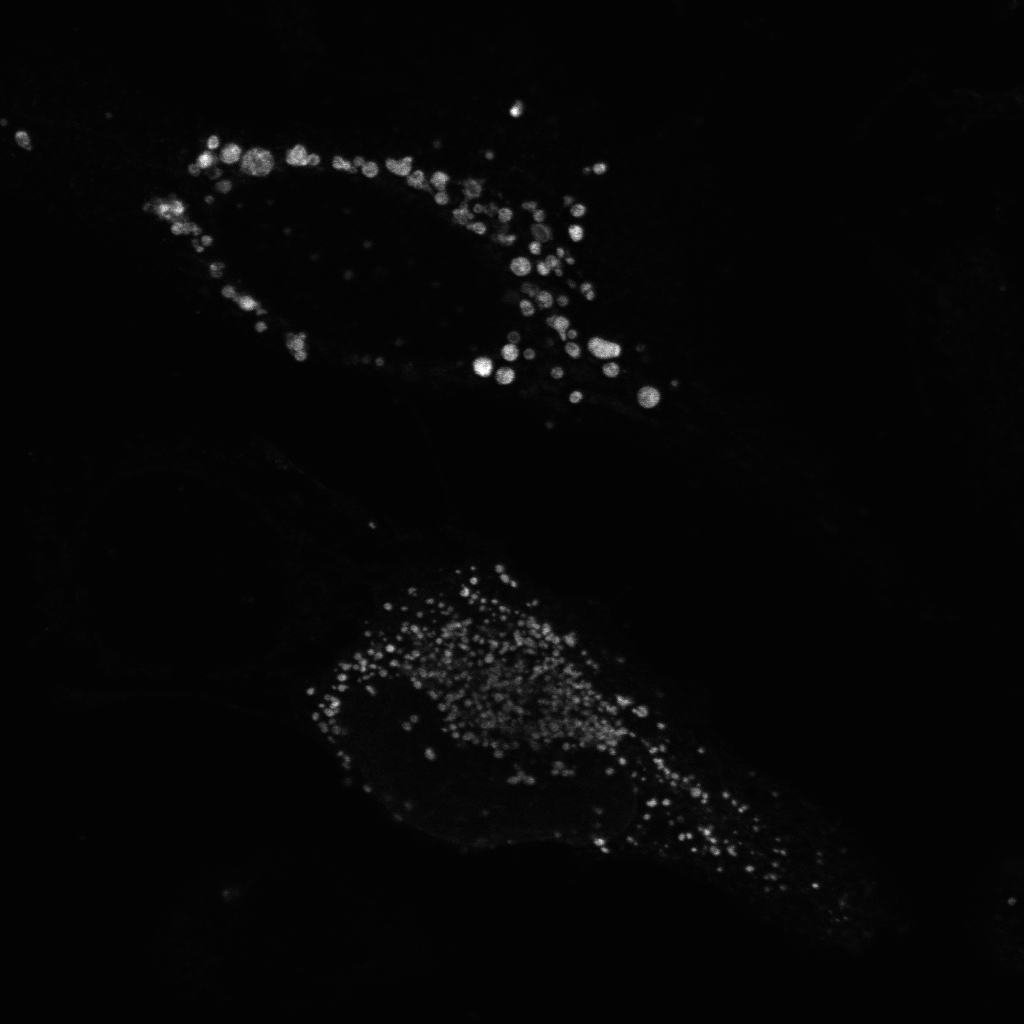

Supplement: Supplementary file 23 — Figure EV4-1 Source Data [file 44318_2026_705_MOESM23_ESM.zip › Figure EV4-1/B/U2OS_NT/C2-20211105_loc_STARD3.lif - U2OSWT_1.tif]

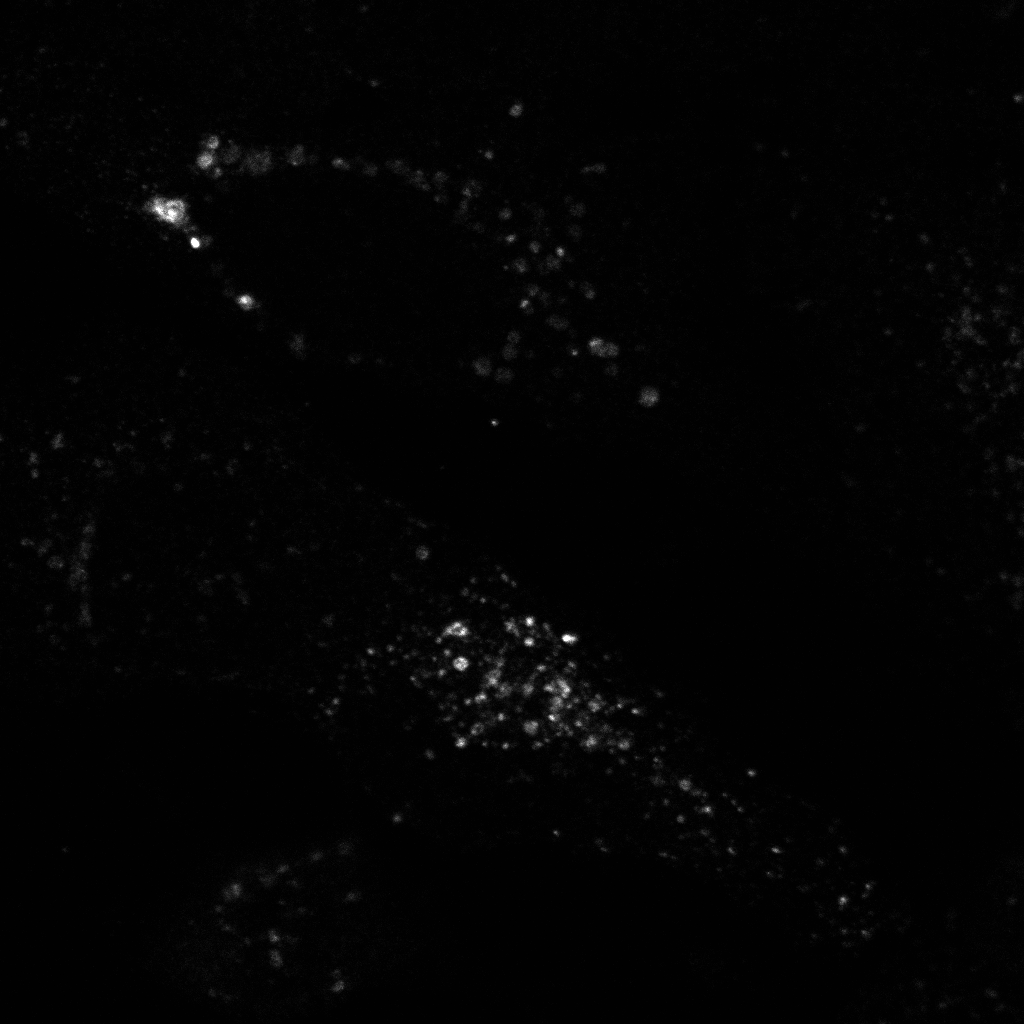

Supplement: Supplementary file 23 — Figure EV4-1 Source Data [file 44318_2026_705_MOESM23_ESM.zip › Figure EV4-1/B/U2OS_NT/C3-20211105_loc_STARD3.lif - U2OSWT_1.tif]

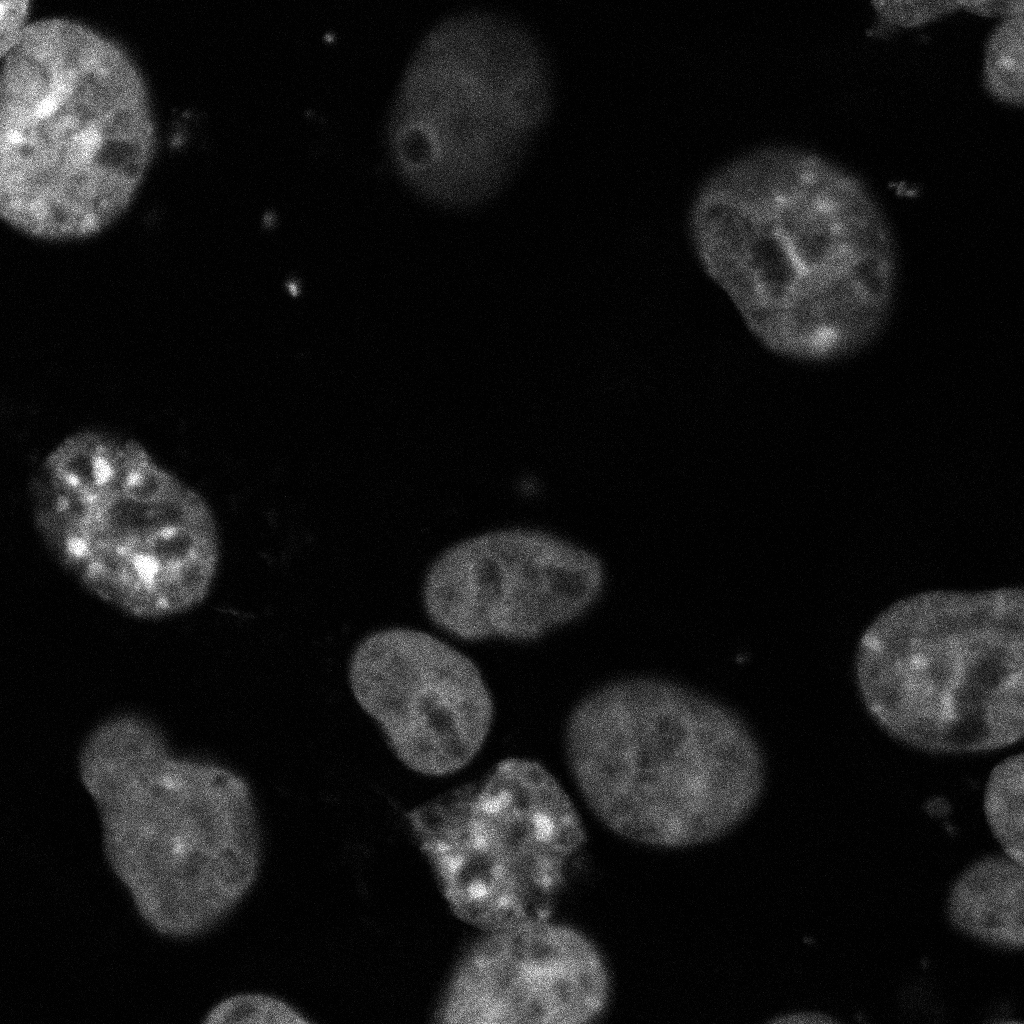

Supplement: Supplementary file 23 — Figure EV4-1 Source Data [file 44318_2026_705_MOESM23_ESM.zip › Figure EV4-1/C/COS7_CHIR99021/C1-20211022_loc_endo_STARD3_Cos_CHIR99021.tif]

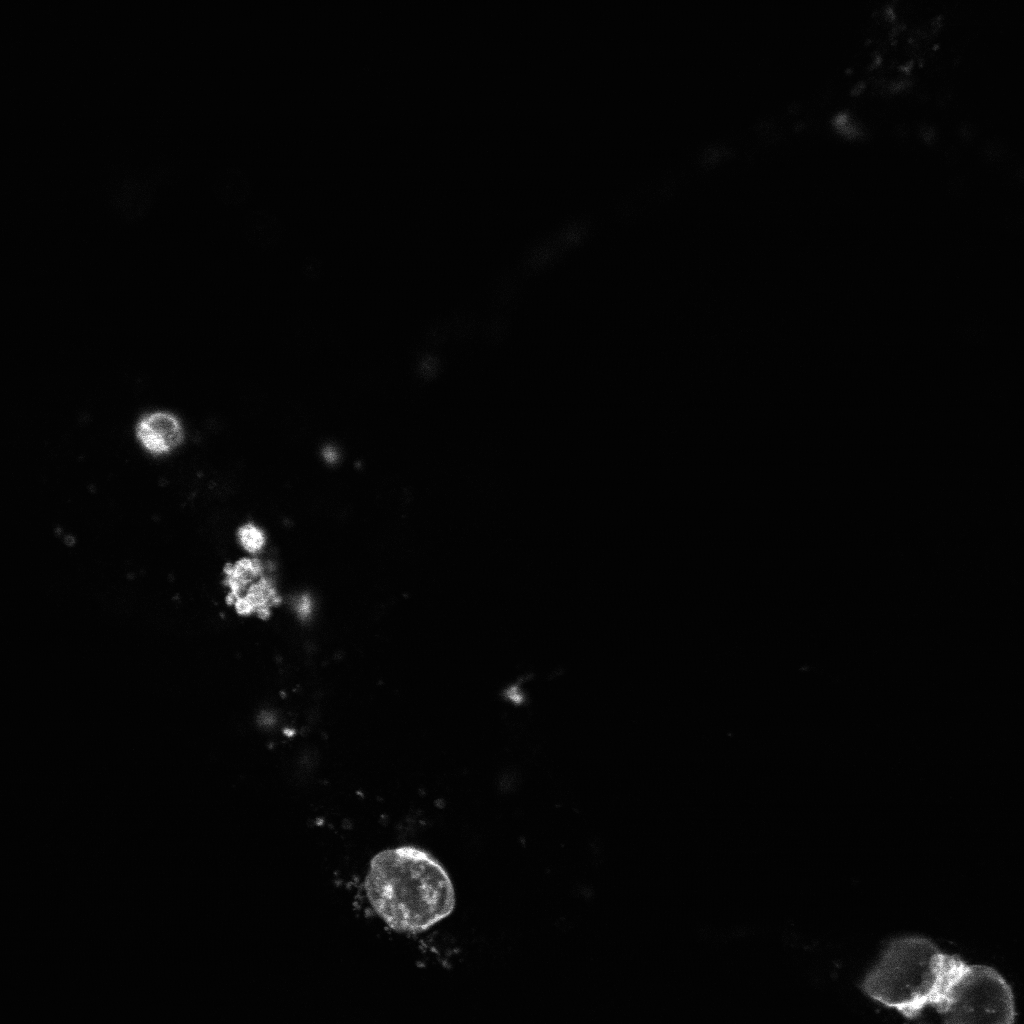

Supplement: Supplementary file 23 — Figure EV4-1 Source Data [file 44318_2026_705_MOESM23_ESM.zip › Figure EV4-1/C/COS7_CHIR99021/C2-20211022_loc_endo_STARD3_Cos_CHIR99021.tif]

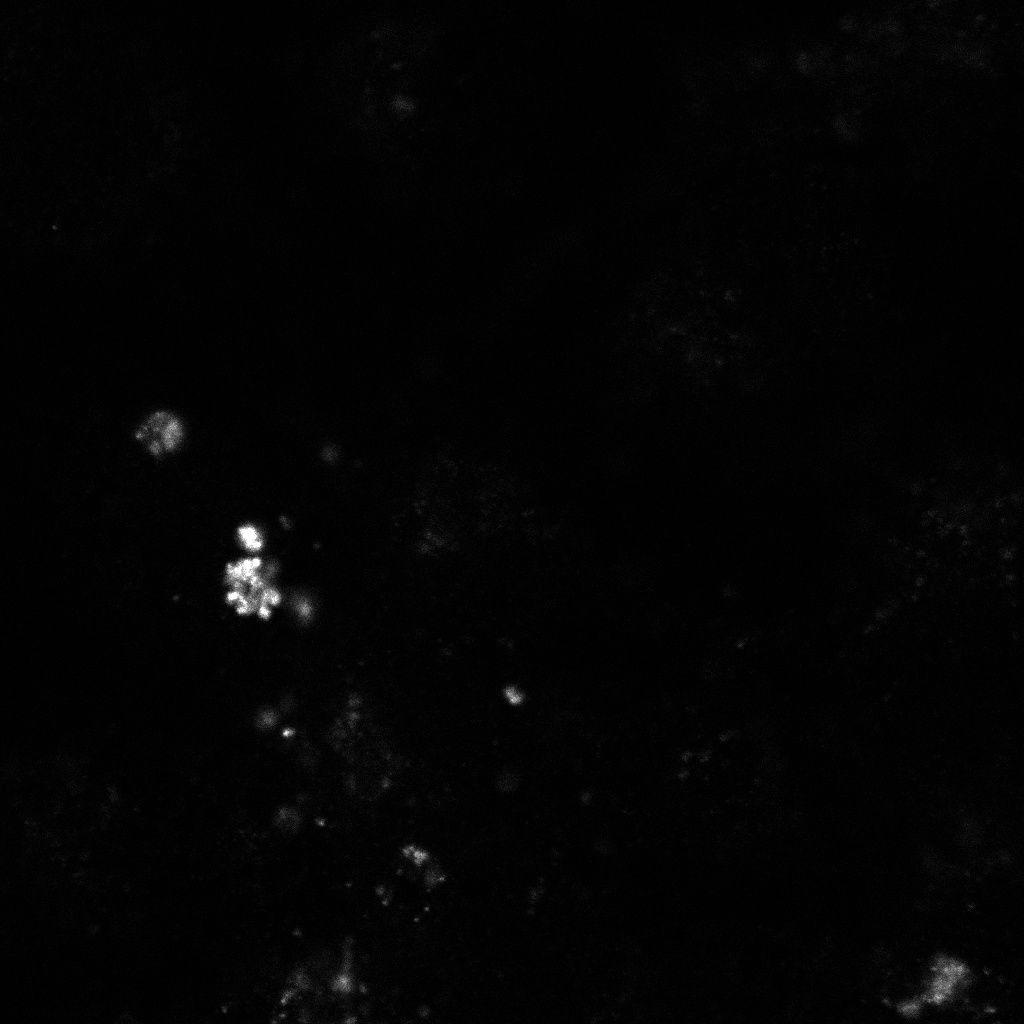

Supplement: Supplementary file 23 — Figure EV4-1 Source Data [file 44318_2026_705_MOESM23_ESM.zip › Figure EV4-1/C/COS7_CHIR99021/C3-20211022_loc_endo_STARD3_Cos_CHIR99021.tif]

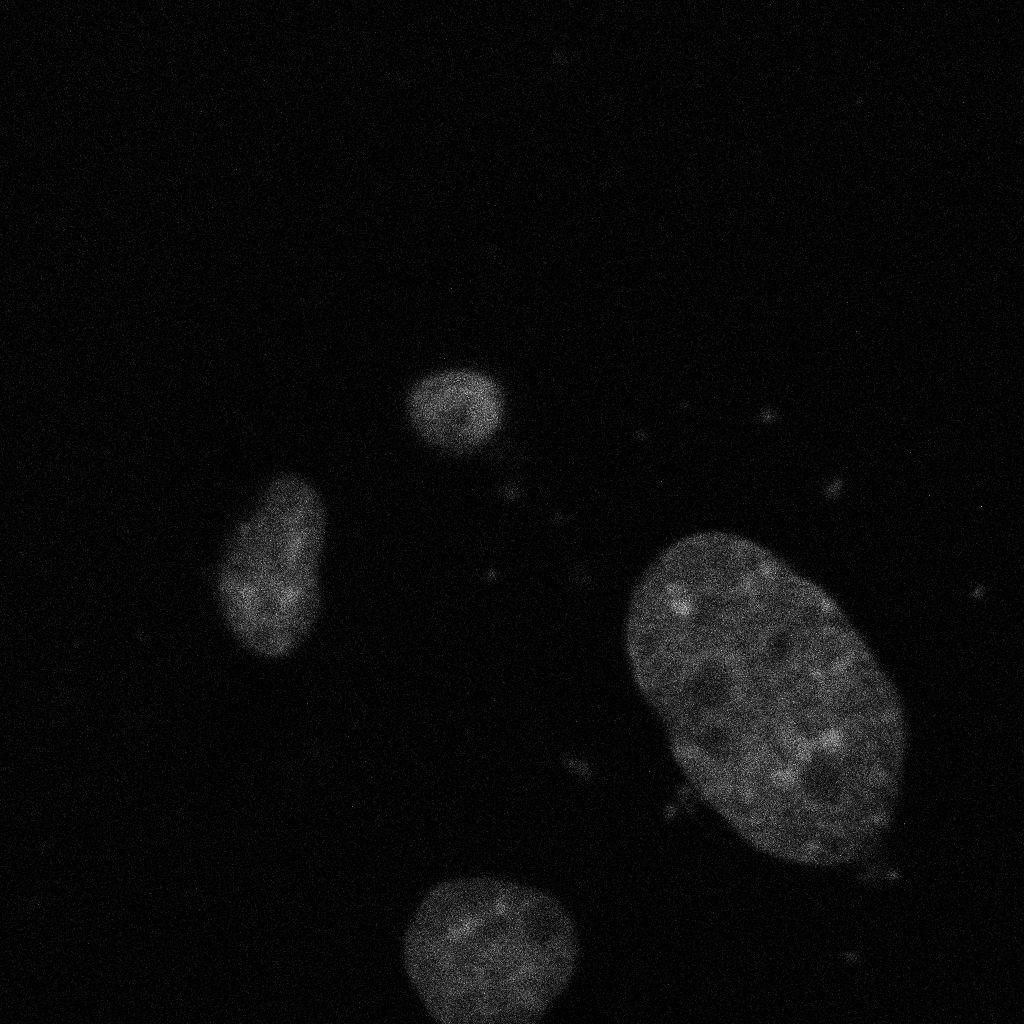

Supplement: Supplementary file 23 — Figure EV4-1 Source Data [file 44318_2026_705_MOESM23_ESM.zip › Figure EV4-1/C/COS7_NT/C1-20211022_loc_endo_STARD3_ Cos_NT.tif]

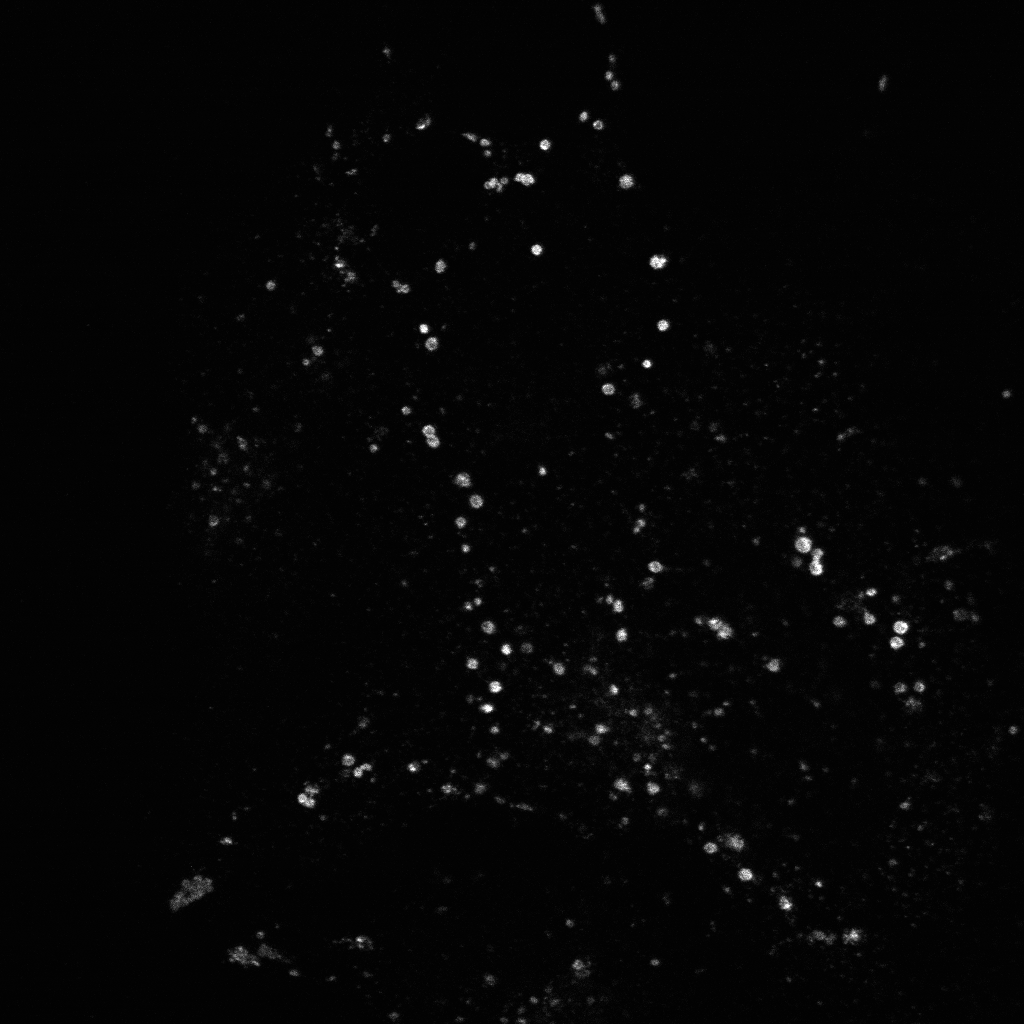

Supplement: Supplementary file 23 — Figure EV4-1 Source Data [file 44318_2026_705_MOESM23_ESM.zip › Figure EV4-1/C/COS7_NT/C2-20211022_loc_endo_STARD3_ Cos_NT.tif]

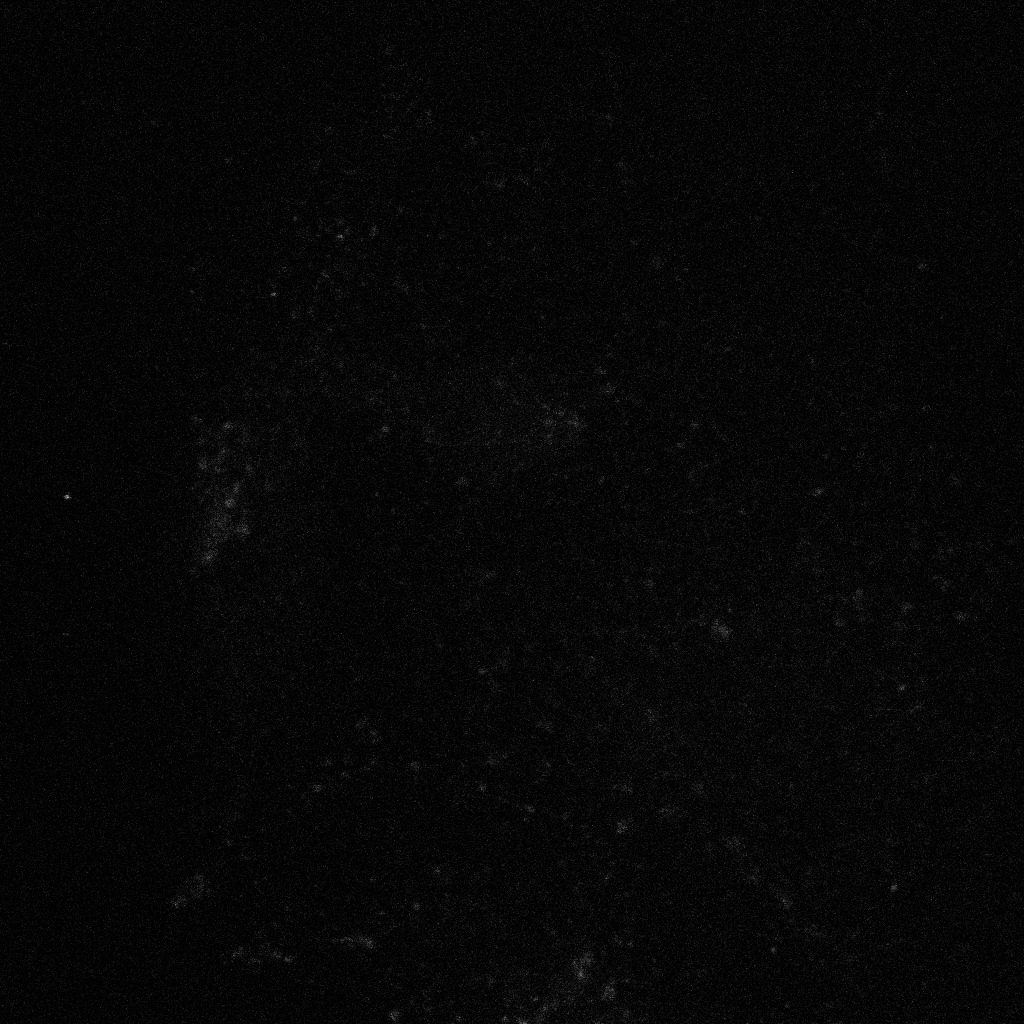

Supplement: Supplementary file 23 — Figure EV4-1 Source Data [file 44318_2026_705_MOESM23_ESM.zip › Figure EV4-1/C/COS7_NT/C3-20211022_loc_endo_STARD3_ Cos_NT.tif]

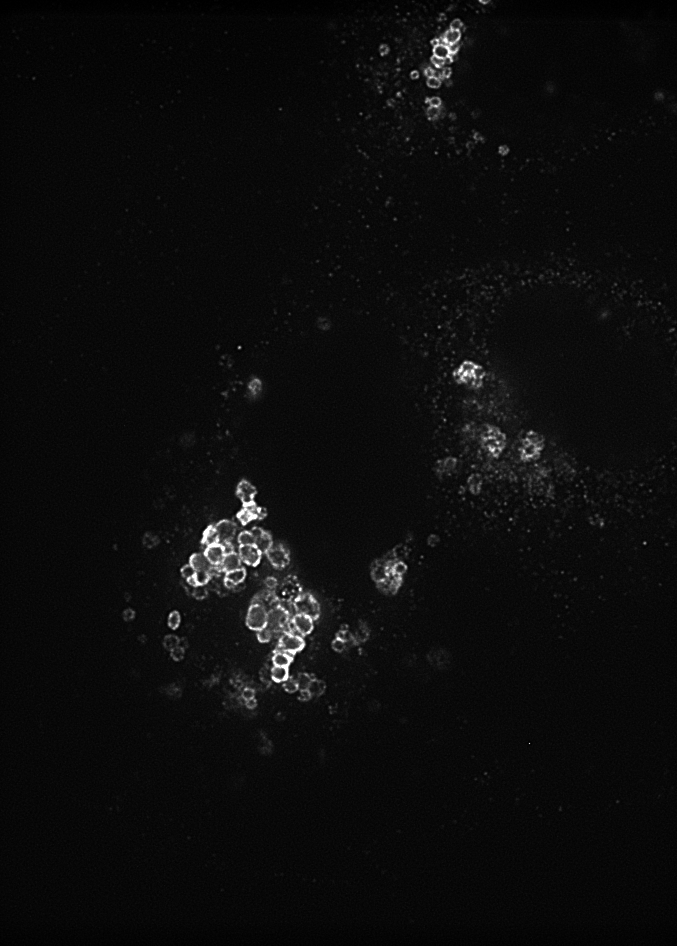

Supplement: Supplementary file 23 — Figure EV4-1 Source Data [file 44318_2026_705_MOESM23_ESM.zip › Figure EV4-1/D/STARD3WT_CHIR99021/20221104_MCF7STARD3WT_GSK3i_10_SR_w1SPI 491 GFP.TIF]

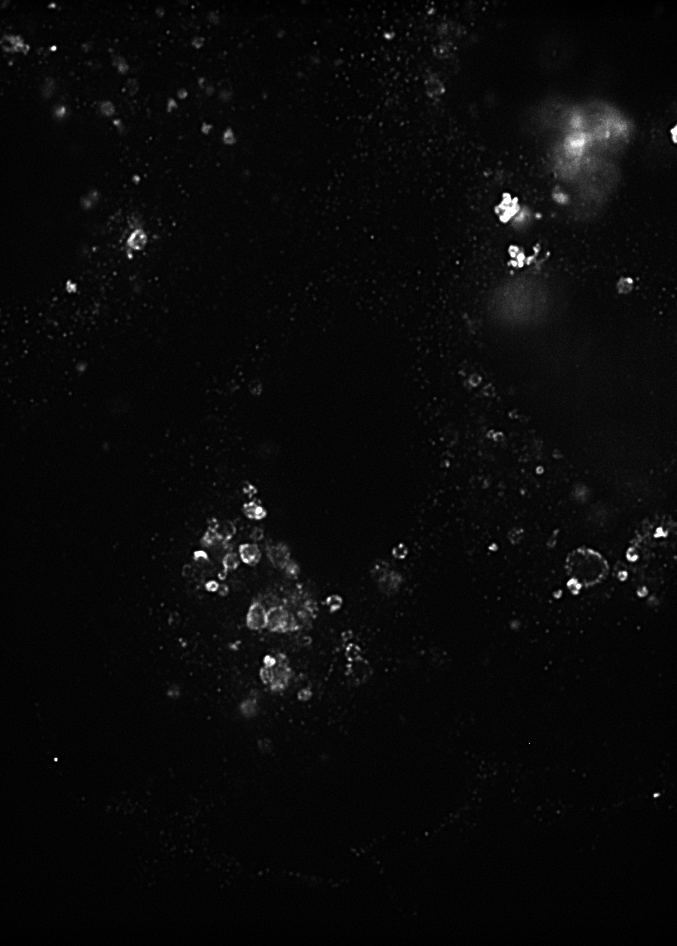

Supplement: Supplementary file 23 — Figure EV4-1 Source Data [file 44318_2026_705_MOESM23_ESM.zip › Figure EV4-1/D/STARD3WT_CHIR99021/20221104_MCF7STARD3WT_GSK3i_10_SR_w2SPI 561 mCherry.TIF]

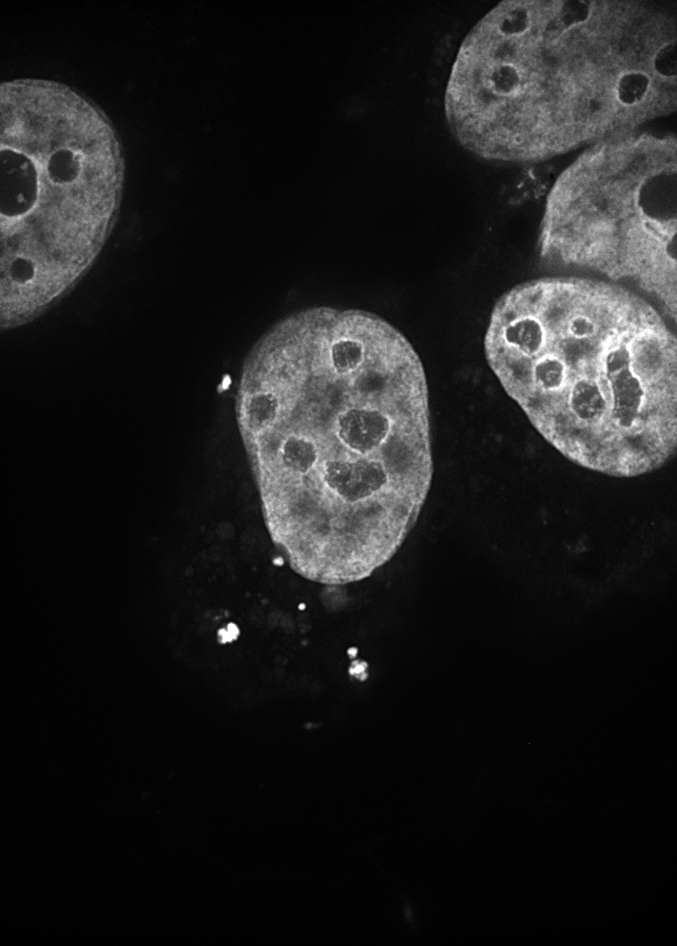

Supplement: Supplementary file 23 — Figure EV4-1 Source Data [file 44318_2026_705_MOESM23_ESM.zip › Figure EV4-1/D/STARD3WT_CHIR99021/20221104_MCF7STARD3WT_GSK3i_10_SR_w3SPI 405 DAPI.TIF]

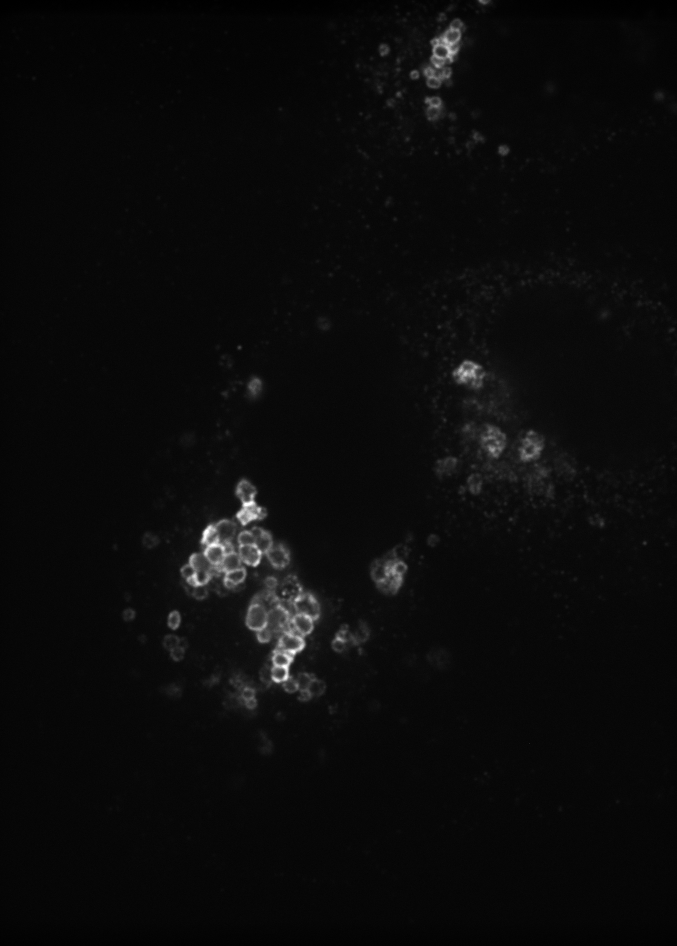

Supplement: Supplementary file 23 — Figure EV4-1 Source Data [file 44318_2026_705_MOESM23_ESM.zip › Figure EV4-1/D/STARD3WT_CHIR99021/20221104_MCF7STARD3WT_GSK3i_10_w1SPI 491 GFP.TIF]

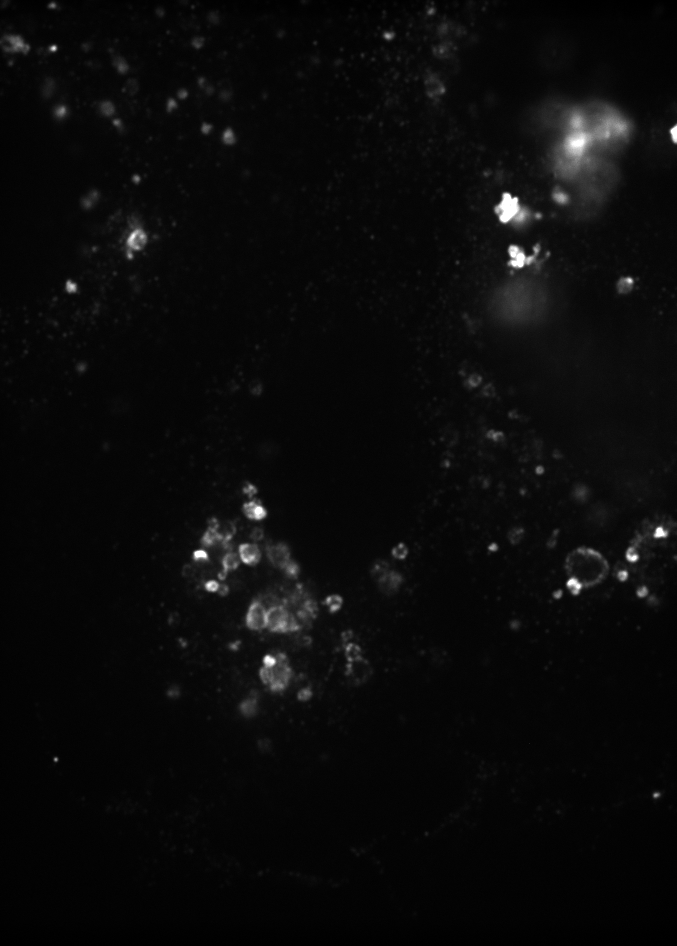

Supplement: Supplementary file 23 — Figure EV4-1 Source Data [file 44318_2026_705_MOESM23_ESM.zip › Figure EV4-1/D/STARD3WT_CHIR99021/20221104_MCF7STARD3WT_GSK3i_10_w2SPI 561 mCherry.TIF]

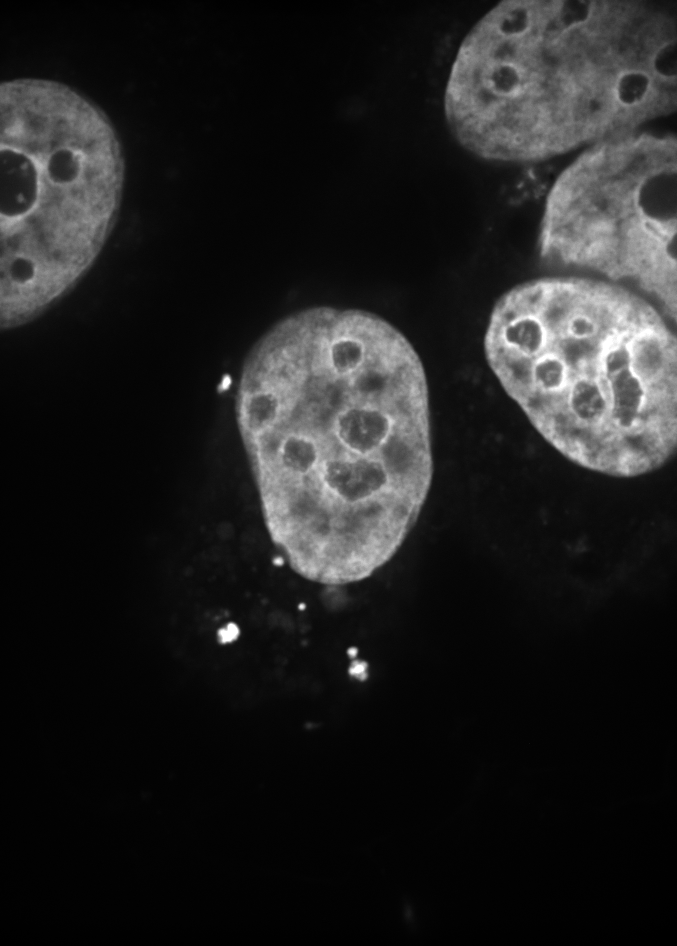

Supplement: Supplementary file 23 — Figure EV4-1 Source Data [file 44318_2026_705_MOESM23_ESM.zip › Figure EV4-1/D/STARD3WT_CHIR99021/20221104_MCF7STARD3WT_GSK3i_10_w3SPI 405 DAPI.TIF]

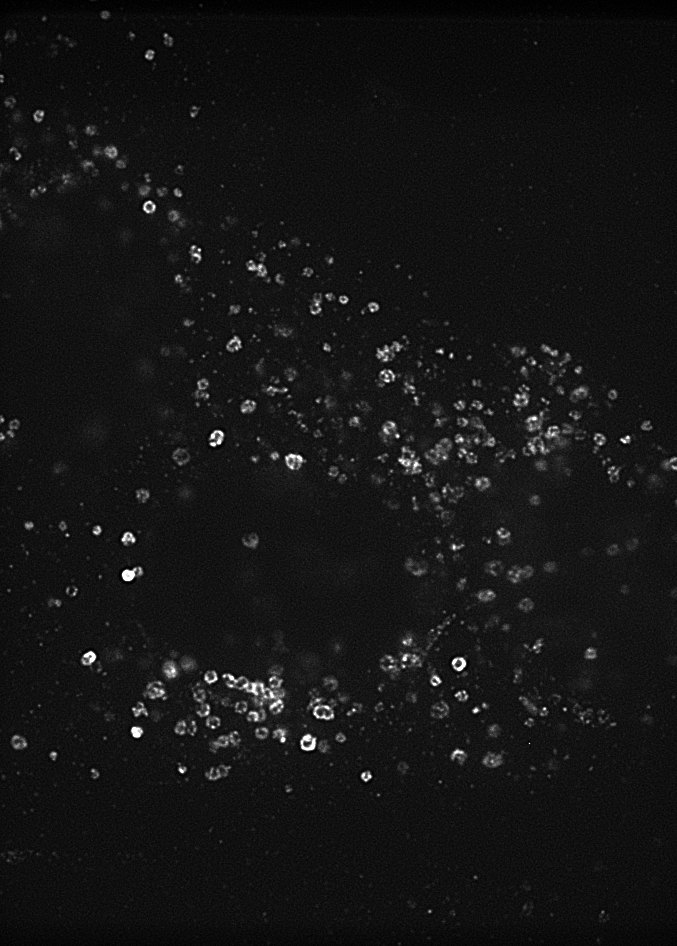

Supplement: Supplementary file 23 — Figure EV4-1 Source Data [file 44318_2026_705_MOESM23_ESM.zip › Figure EV4-1/D/STARD3WT_NT/20221104_MCF7STARD3WT_NT_8_SR_w1SPI 491 GFP.TIF]

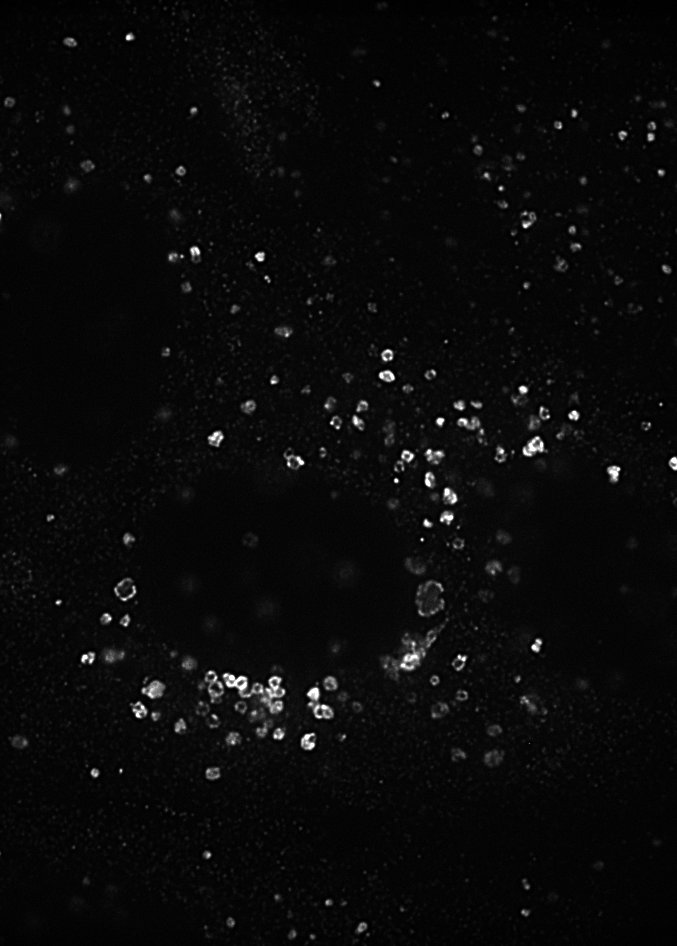

Supplement: Supplementary file 23 — Figure EV4-1 Source Data [file 44318_2026_705_MOESM23_ESM.zip › Figure EV4-1/D/STARD3WT_NT/20221104_MCF7STARD3WT_NT_8_SR_w2SPI 561 mCherry.TIF]

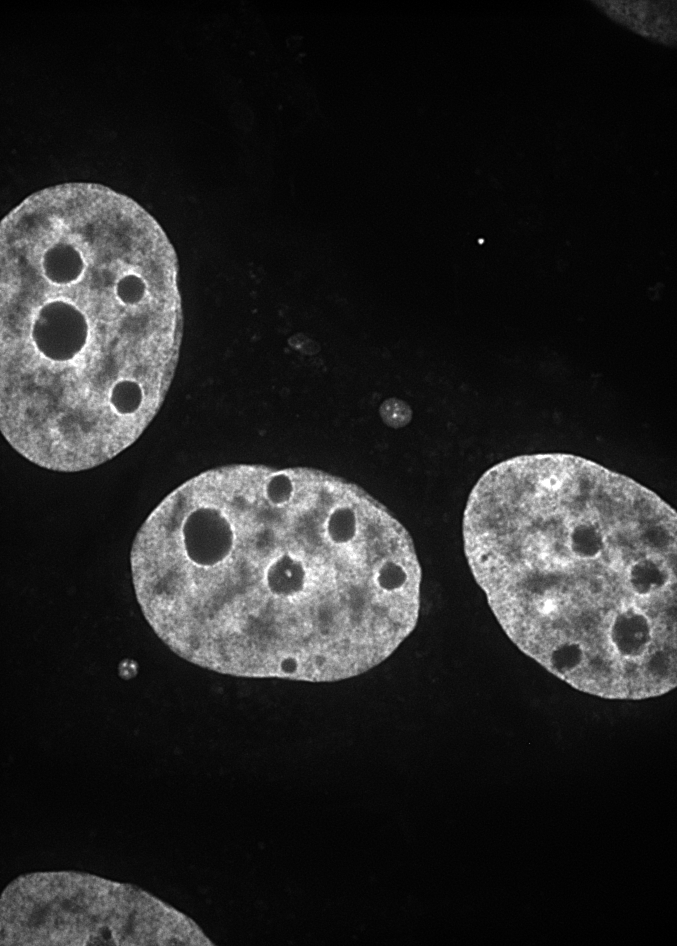

Supplement: Supplementary file 23 — Figure EV4-1 Source Data [file 44318_2026_705_MOESM23_ESM.zip › Figure EV4-1/D/STARD3WT_NT/20221104_MCF7STARD3WT_NT_8_SR_w3SPI 405 DAPI.TIF]

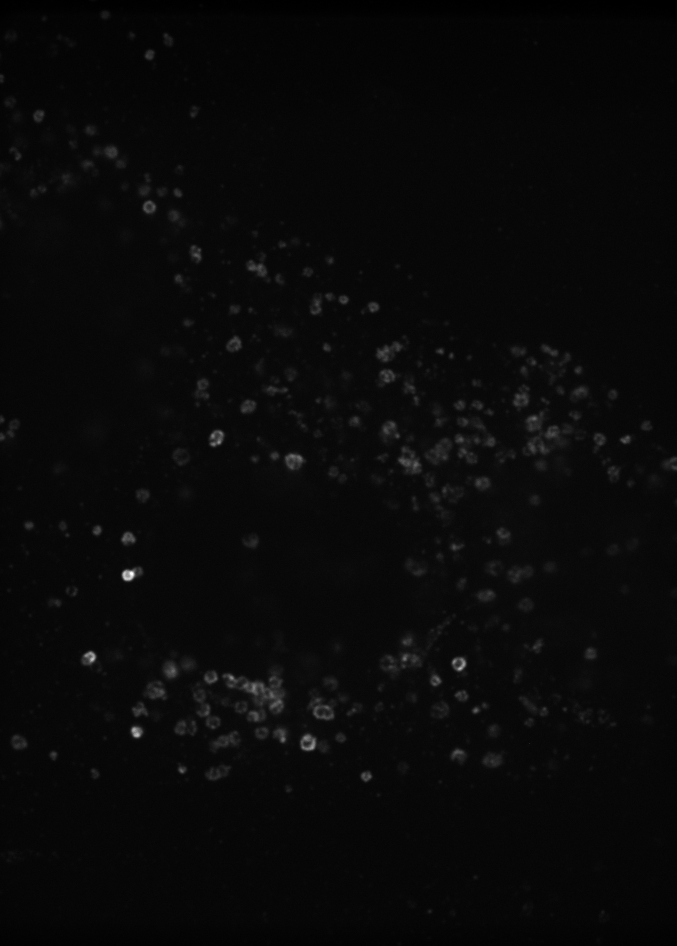

Supplement: Supplementary file 23 — Figure EV4-1 Source Data [file 44318_2026_705_MOESM23_ESM.zip › Figure EV4-1/D/STARD3WT_NT/20221104_MCF7STARD3WT_NT_8_w1SPI 491 GFP.TIF]

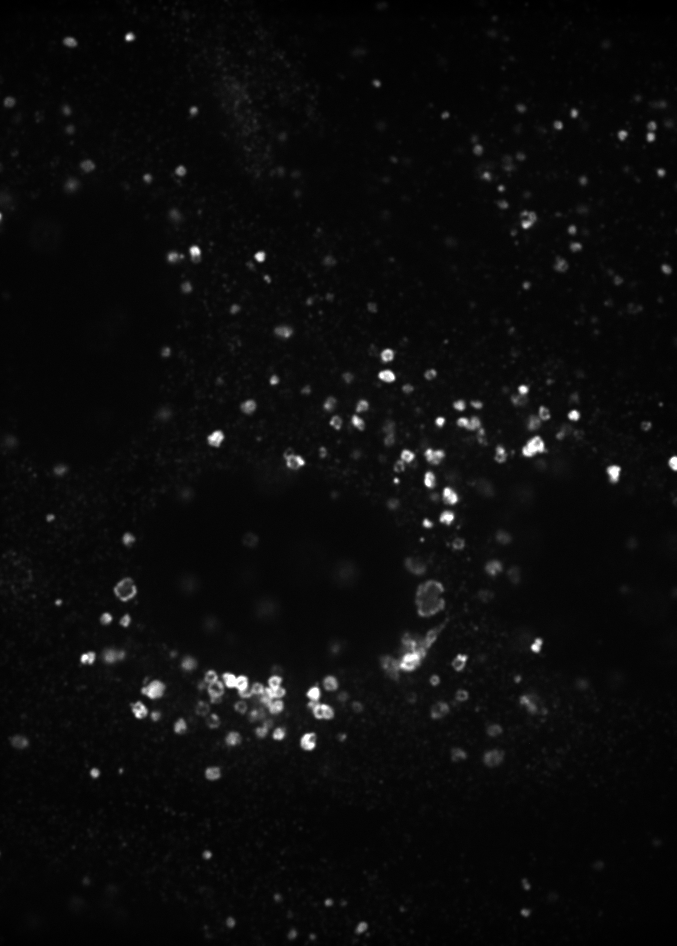

Supplement: Supplementary file 23 — Figure EV4-1 Source Data [file 44318_2026_705_MOESM23_ESM.zip › Figure EV4-1/D/STARD3WT_NT/20221104_MCF7STARD3WT_NT_8_w2SPI 561 mCherry.TIF]

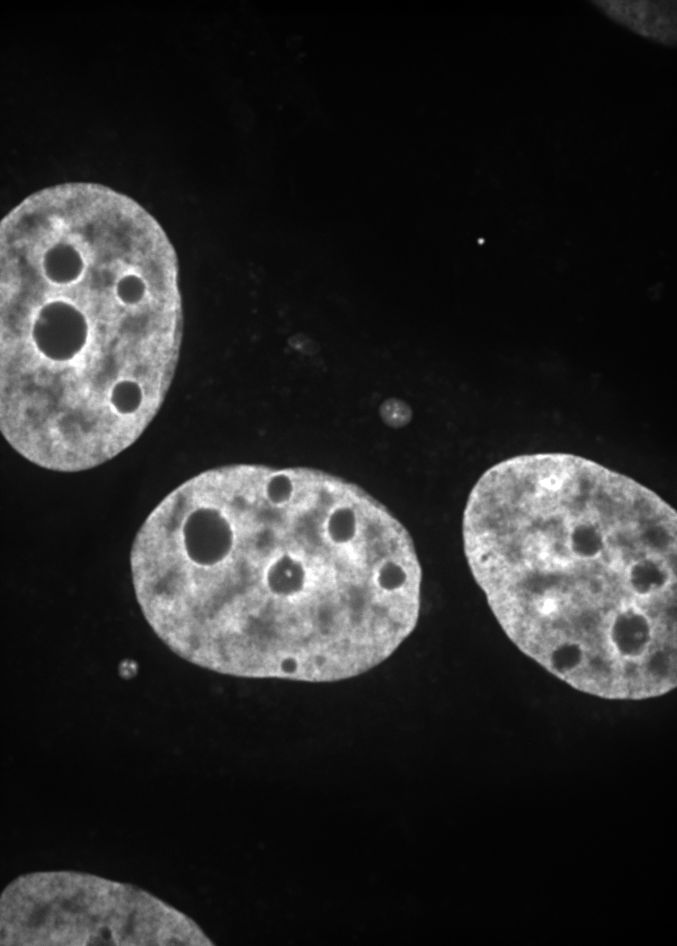

Supplement: Supplementary file 23 — Figure EV4-1 Source Data [file 44318_2026_705_MOESM23_ESM.zip › Figure EV4-1/D/STARD3WT_NT/20221104_MCF7STARD3WT_NT_8_w3SPI 405 DAPI.TIF]

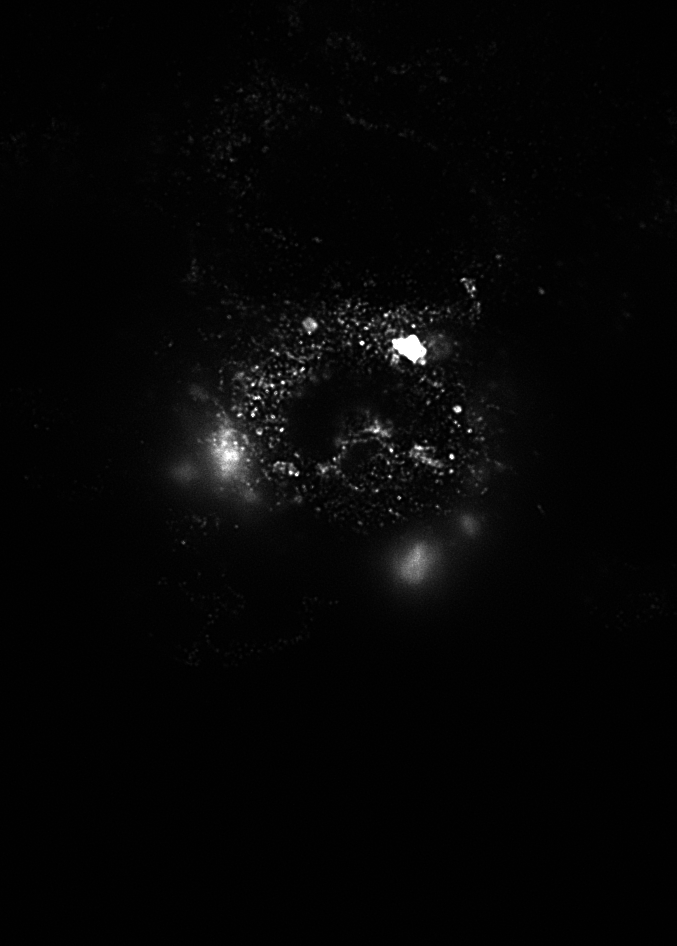

Supplement: Supplementary file 23 — Figure EV4-1 Source Data [file 44318_2026_705_MOESM23_ESM.zip › Figure EV4-1/E/STARD3S209A_CHIR99021/20220509_MCF7_STARD3S209A_LAMPmch_GSK3i_4_SR_w1SPI 491 GFP.TIF]

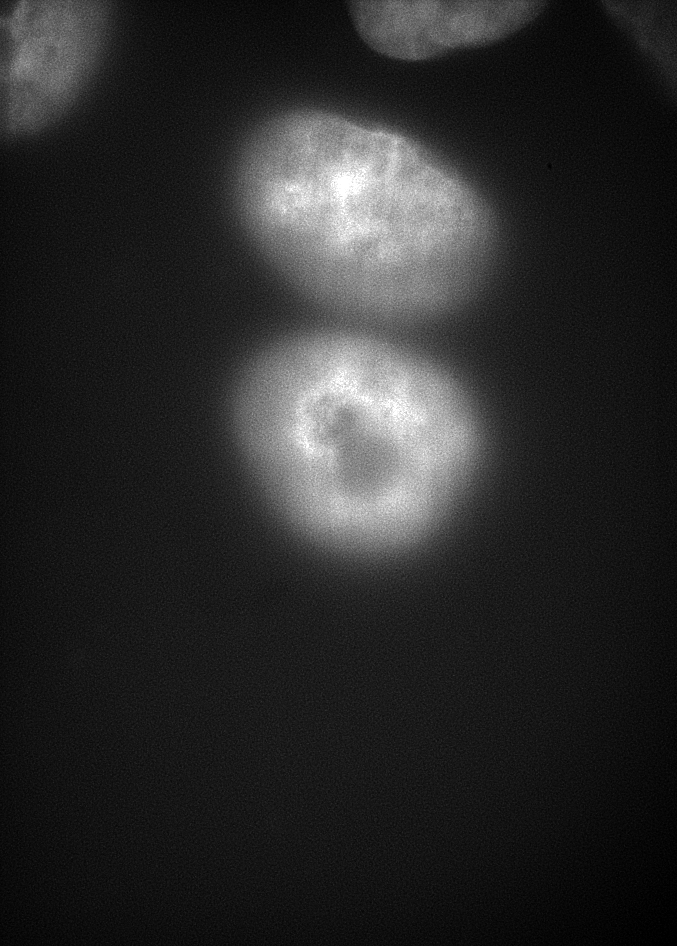

Supplement: Supplementary file 23 — Figure EV4-1 Source Data [file 44318_2026_705_MOESM23_ESM.zip › Figure EV4-1/E/STARD3S209A_CHIR99021/20220509_MCF7_STARD3S209A_LAMPmch_GSK3i_4_SR_w2SPI 405 DAPI.TIF]

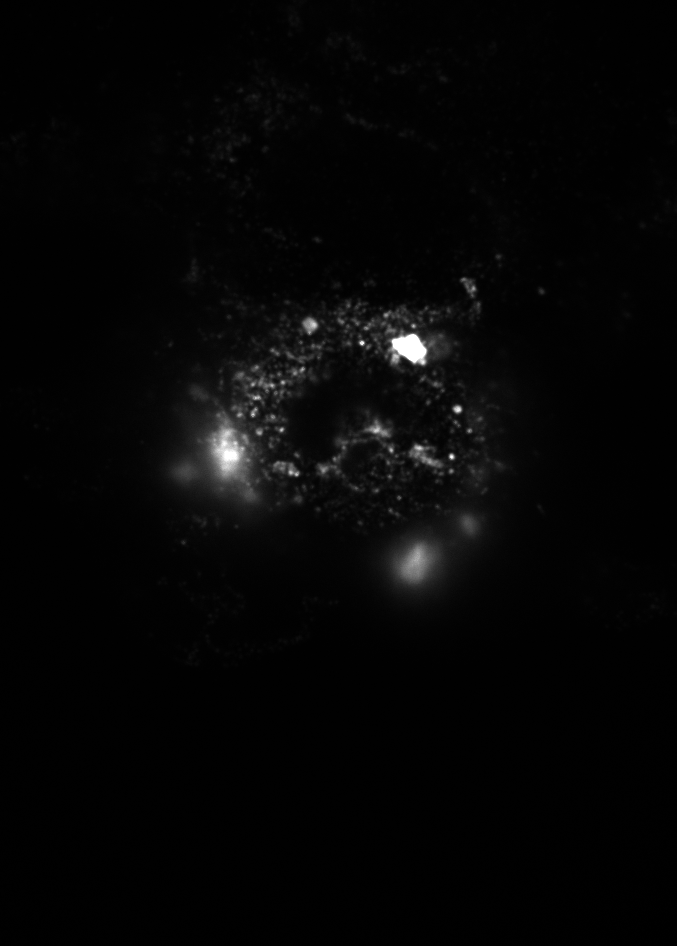

Supplement: Supplementary file 23 — Figure EV4-1 Source Data [file 44318_2026_705_MOESM23_ESM.zip › Figure EV4-1/E/STARD3S209A_CHIR99021/20220509_MCF7_STARD3S209A_LAMPmch_GSK3i_4_w1SPI 491 GFP.TIF]

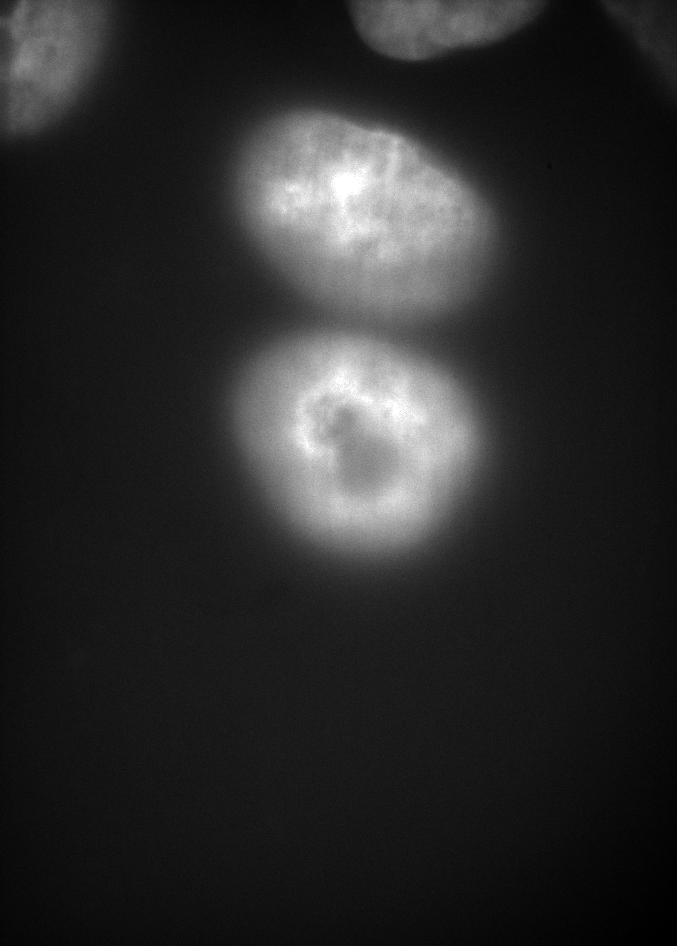

Supplement: Supplementary file 23 — Figure EV4-1 Source Data [file 44318_2026_705_MOESM23_ESM.zip › Figure EV4-1/E/STARD3S209A_CHIR99021/20220509_MCF7_STARD3S209A_LAMPmch_GSK3i_4_w2SPI 405 DAPI.TIF]

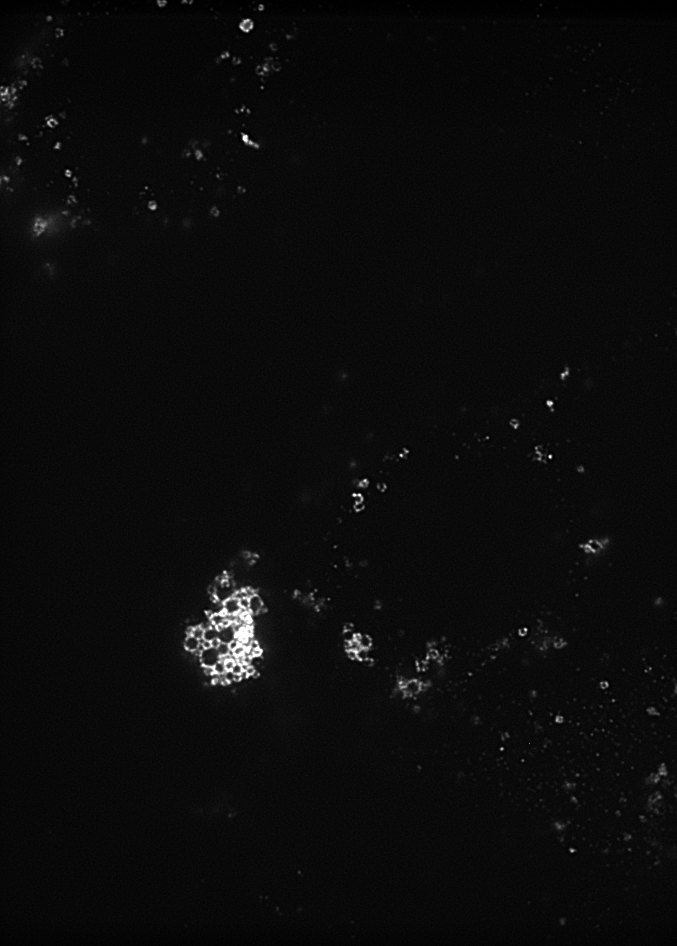

Supplement: Supplementary file 23 — Figure EV4-1 Source Data [file 44318_2026_705_MOESM23_ESM.zip › Figure EV4-1/E/STARD3S209A_NT/20230113_MCF7STARD3S209A_NT_1_SR_w1SPI 491 GFP.TIF]

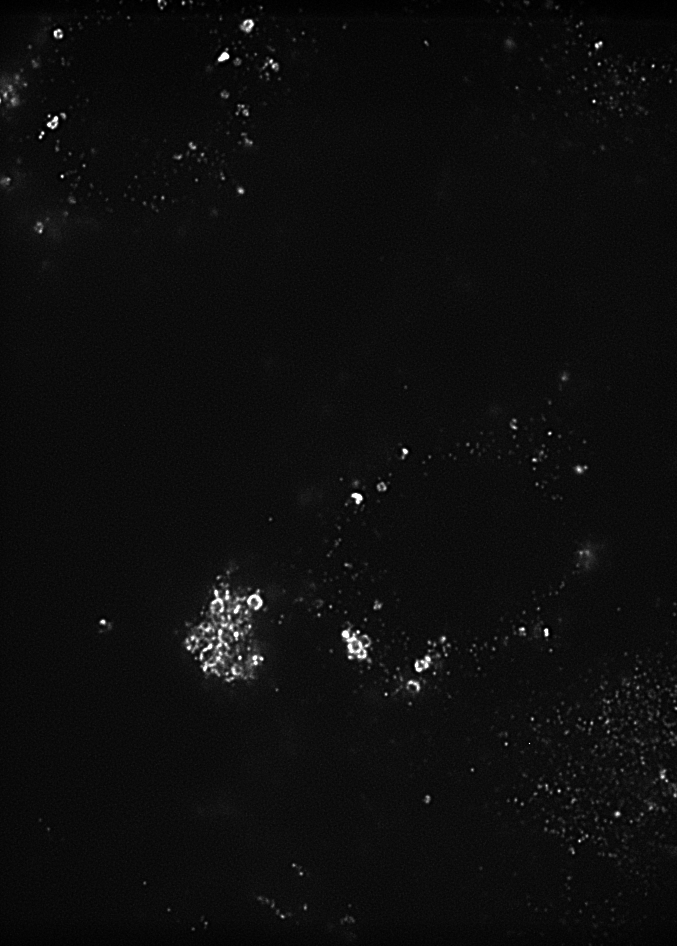

Supplement: Supplementary file 23 — Figure EV4-1 Source Data [file 44318_2026_705_MOESM23_ESM.zip › Figure EV4-1/E/STARD3S209A_NT/20230113_MCF7STARD3S209A_NT_1_SR_w2SPI 561 mCherry.TIF]

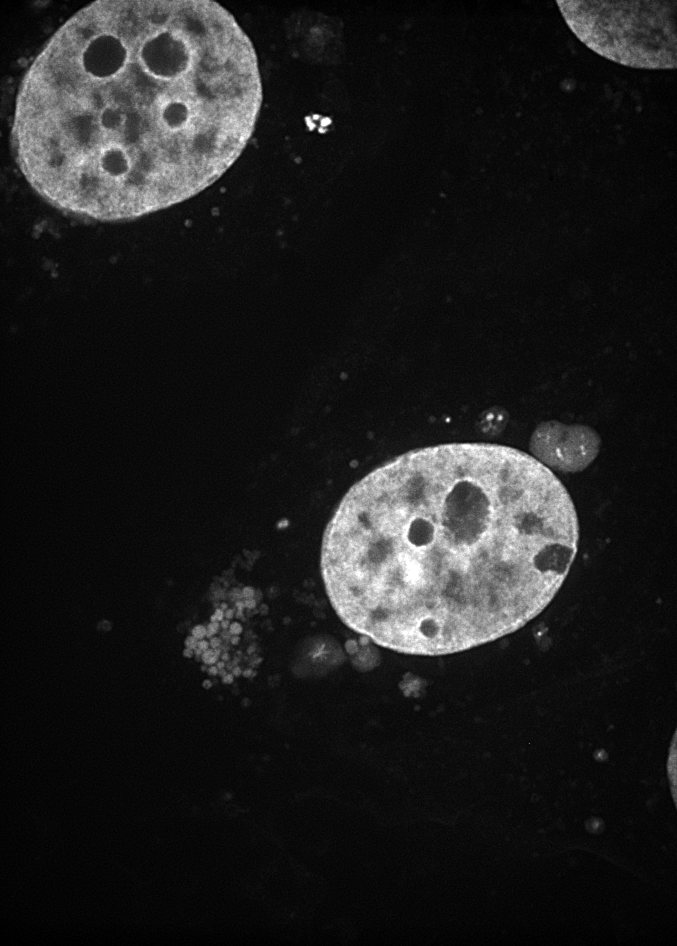

Supplement: Supplementary file 23 — Figure EV4-1 Source Data [file 44318_2026_705_MOESM23_ESM.zip › Figure EV4-1/E/STARD3S209A_NT/20230113_MCF7STARD3S209A_NT_1_SR_w3SPI 405 DAPI.TIF]

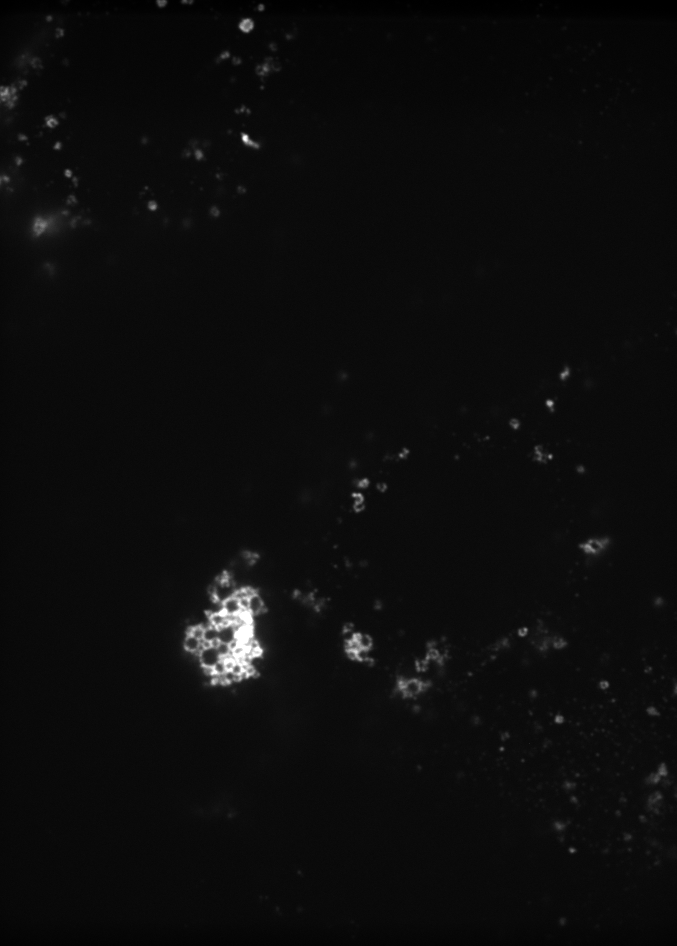

Supplement: Supplementary file 23 — Figure EV4-1 Source Data [file 44318_2026_705_MOESM23_ESM.zip › Figure EV4-1/E/STARD3S209A_NT/20230113_MCF7STARD3S209A_NT_1_w1SPI 491 GFP.TIF]

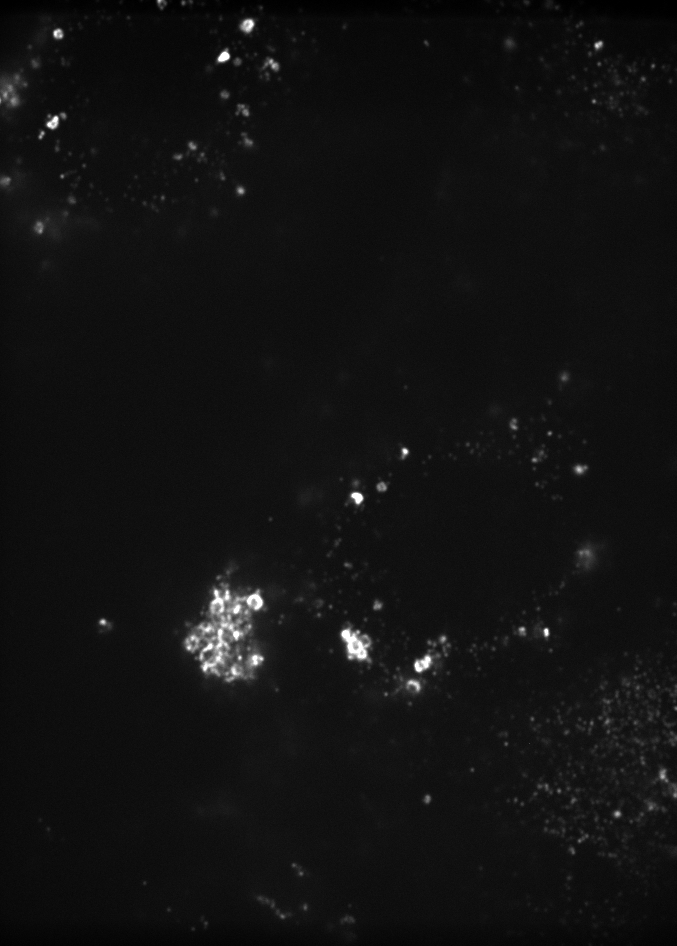

Supplement: Supplementary file 23 — Figure EV4-1 Source Data [file 44318_2026_705_MOESM23_ESM.zip › Figure EV4-1/E/STARD3S209A_NT/20230113_MCF7STARD3S209A_NT_1_w2SPI 561 mCherry.TIF]

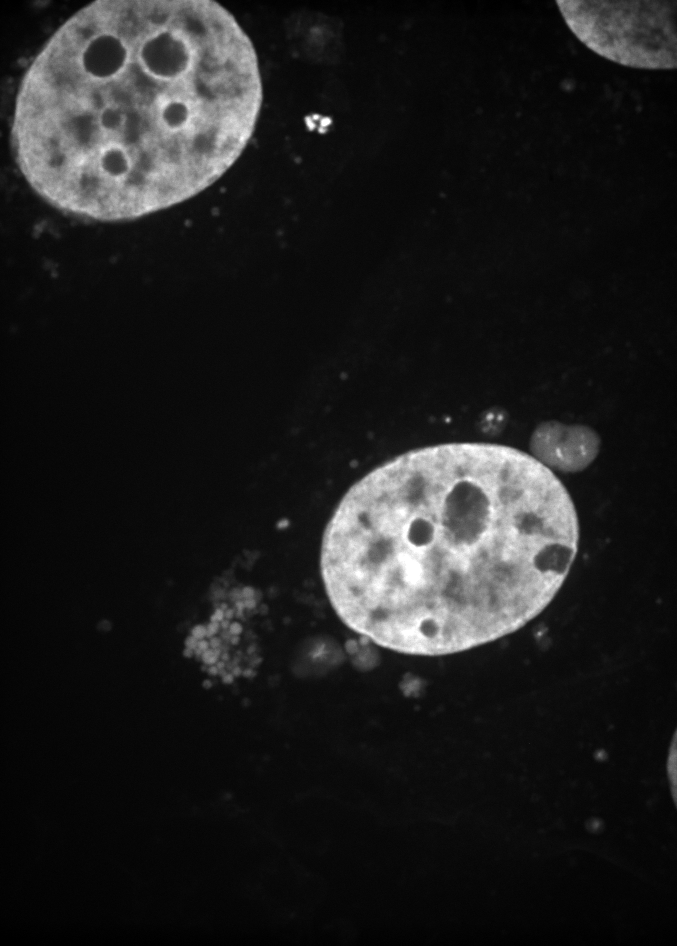

Supplement: Supplementary file 23 — Figure EV4-1 Source Data [file 44318_2026_705_MOESM23_ESM.zip › Figure EV4-1/E/STARD3S209A_NT/20230113_MCF7STARD3S209A_NT_1_w3SPI 405 DAPI.TIF]

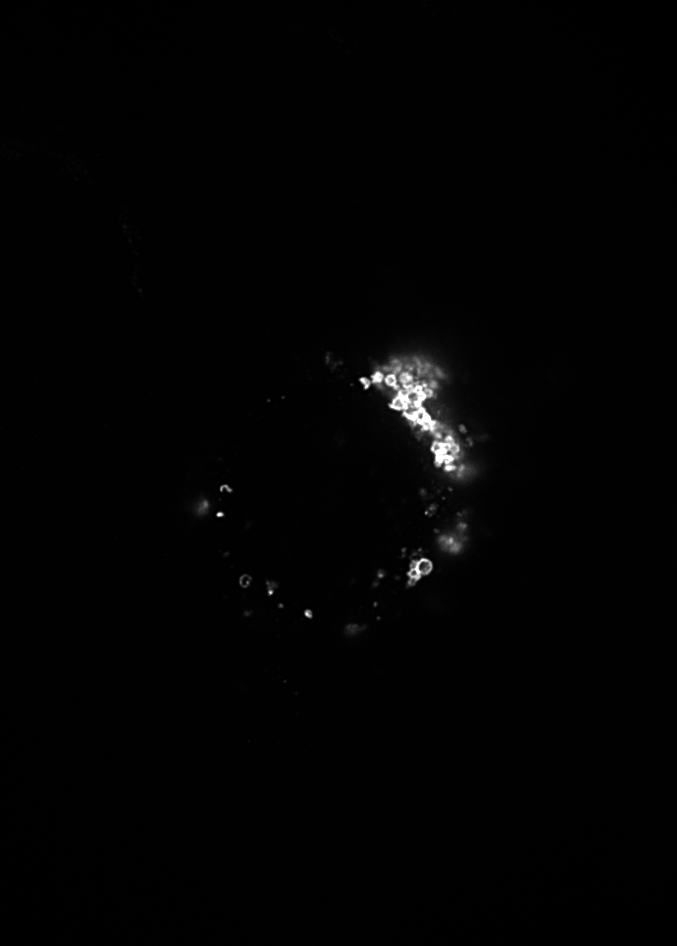

Supplement: Supplementary file 23 — Figure EV4-1 Source Data [file 44318_2026_705_MOESM23_ESM.zip › Figure EV4-1/F/STARD3_deltaFFAT_CHIR99021/20220610_STARD3deltaFFAT_GSK3i_3_SR_w1SPI 491 GFP.TIF]

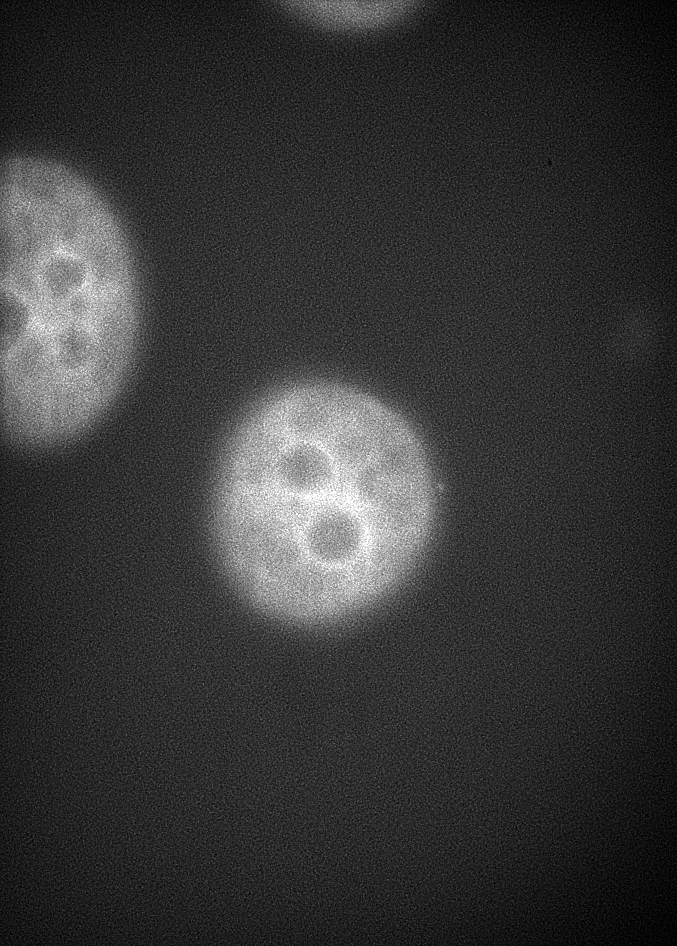

Supplement: Supplementary file 23 — Figure EV4-1 Source Data [file 44318_2026_705_MOESM23_ESM.zip › Figure EV4-1/F/STARD3_deltaFFAT_CHIR99021/20220610_STARD3deltaFFAT_GSK3i_3_SR_w2SPI 405 DAPI.TIF]

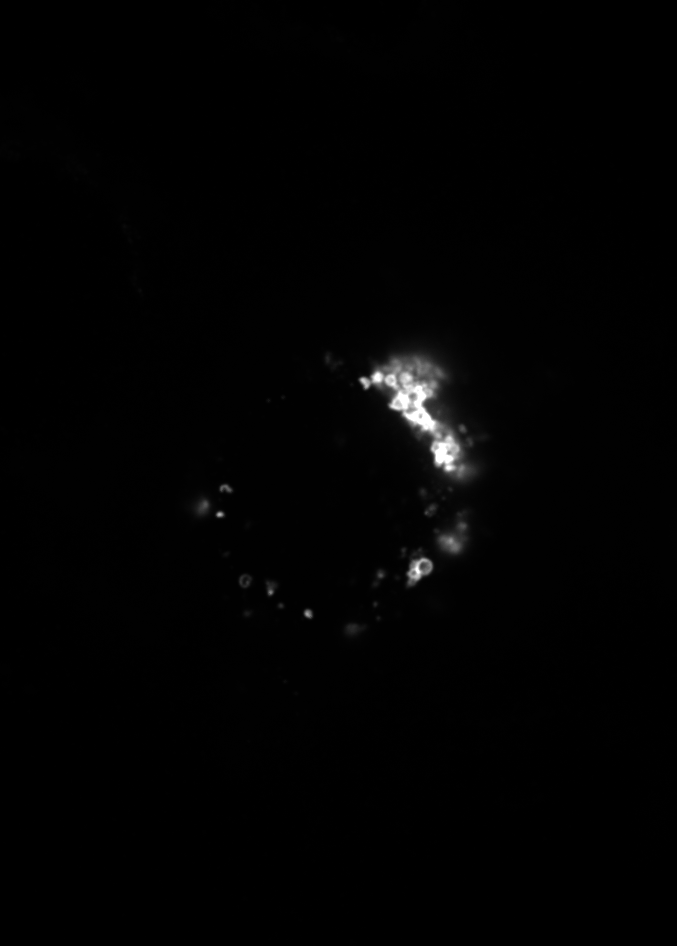

Supplement: Supplementary file 23 — Figure EV4-1 Source Data [file 44318_2026_705_MOESM23_ESM.zip › Figure EV4-1/F/STARD3_deltaFFAT_CHIR99021/20220610_STARD3deltaFFAT_GSK3i_3_w1SPI 491 GFP.TIF]

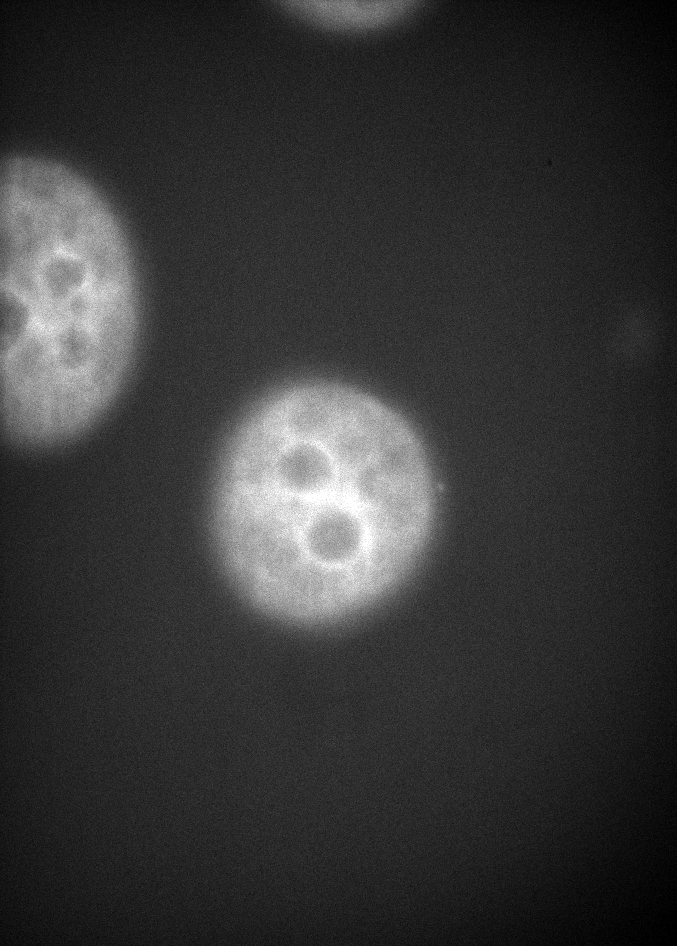

Supplement: Supplementary file 23 — Figure EV4-1 Source Data [file 44318_2026_705_MOESM23_ESM.zip › Figure EV4-1/F/STARD3_deltaFFAT_CHIR99021/20220610_STARD3deltaFFAT_GSK3i_3_w2SPI 405 DAPI.TIF]

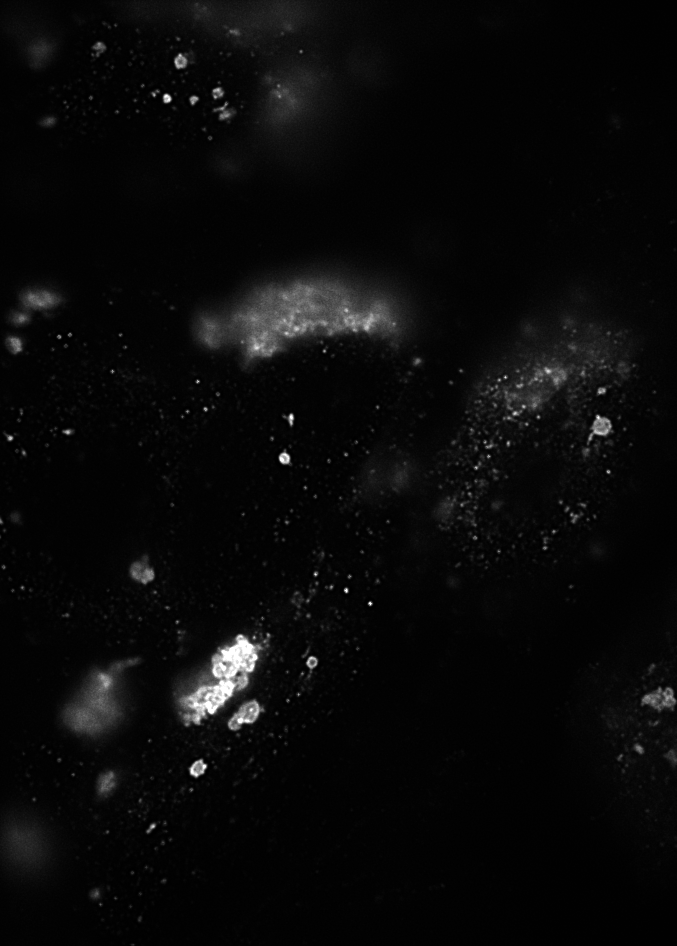

Supplement: Supplementary file 23 — Figure EV4-1 Source Data [file 44318_2026_705_MOESM23_ESM.zip › Figure EV4-1/F/STARD3_deltaFFAT_NT/20220305_MCF7_D3deltaFFAT_NT_SR_w1SPI 491 GFP.TIF]

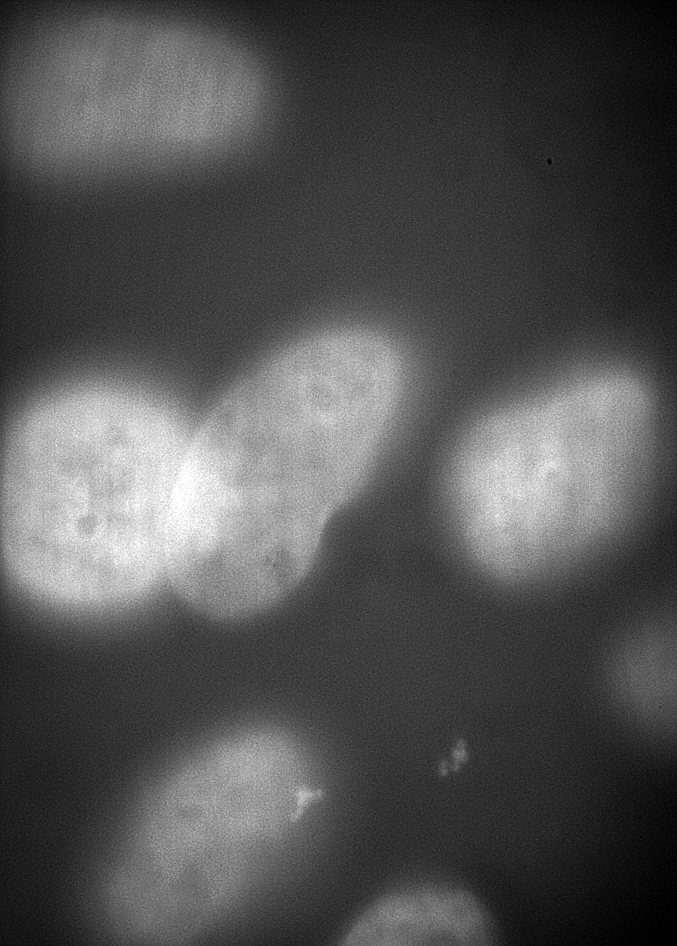

Supplement: Supplementary file 23 — Figure EV4-1 Source Data [file 44318_2026_705_MOESM23_ESM.zip › Figure EV4-1/F/STARD3_deltaFFAT_NT/20220305_MCF7_D3deltaFFAT_NT_SR_w2SPI 405 DAPI.TIF]

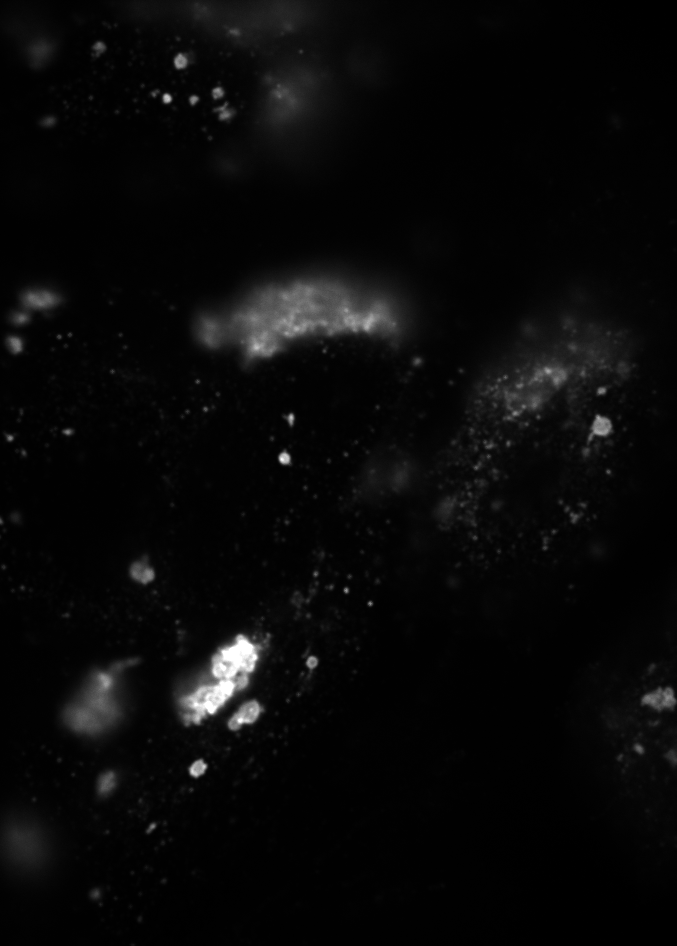

Supplement: Supplementary file 23 — Figure EV4-1 Source Data [file 44318_2026_705_MOESM23_ESM.zip › Figure EV4-1/F/STARD3_deltaFFAT_NT/20220305_MCF7_D3deltaFFAT_NT_w1SPI 491 GFP.TIF]

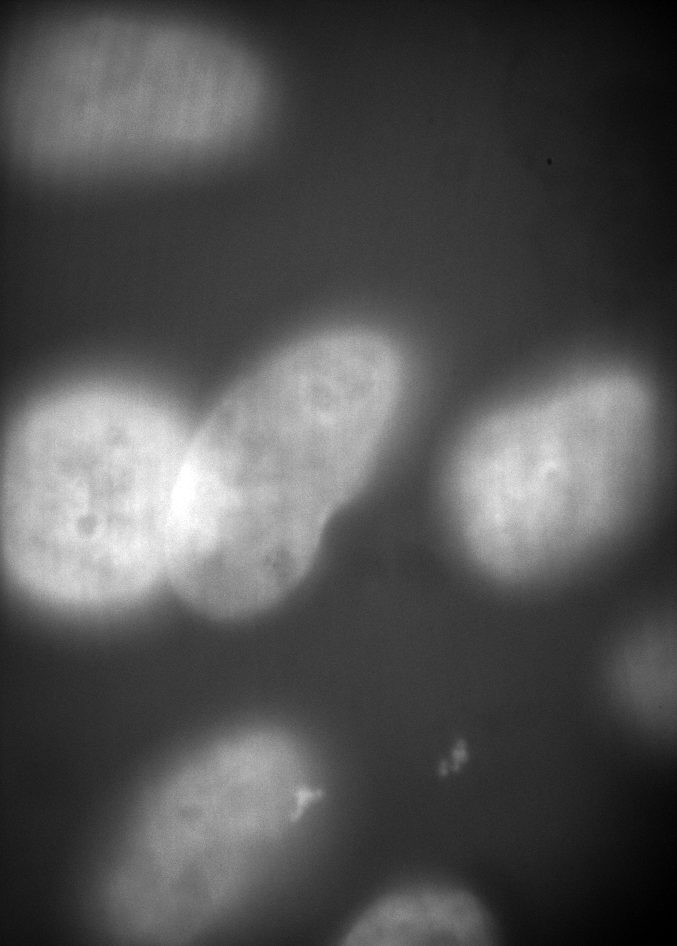

Supplement: Supplementary file 23 — Figure EV4-1 Source Data [file 44318_2026_705_MOESM23_ESM.zip › Figure EV4-1/F/STARD3_deltaFFAT_NT/20220305_MCF7_D3deltaFFAT_NT_w2SPI 405 DAPI.TIF]

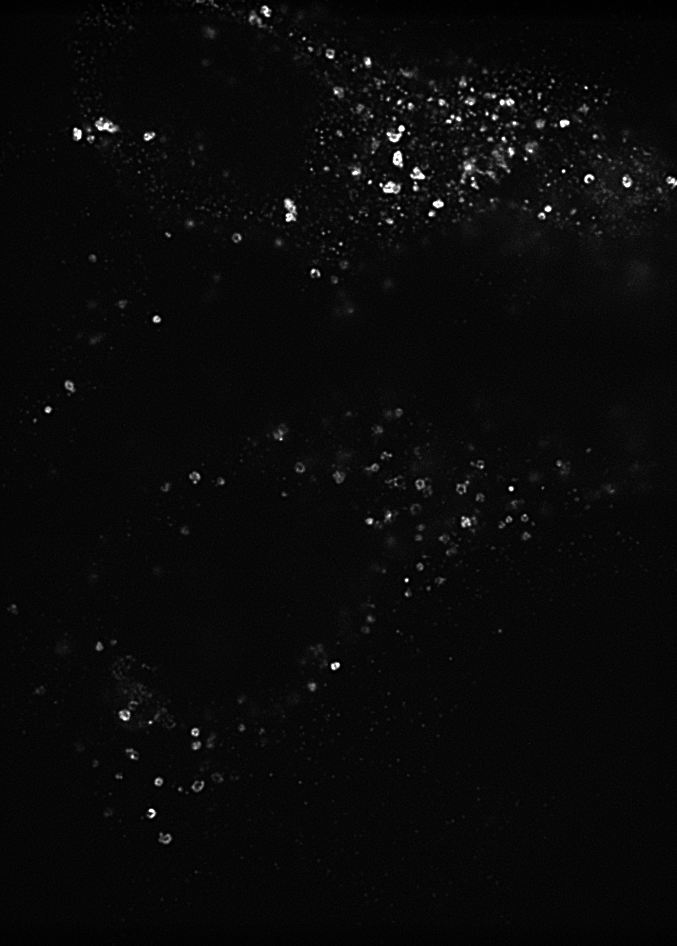

Supplement: Supplementary file 24 — Figure EV4-2 Source Data [file 44318_2026_705_MOESM24_ESM.zip › Figure EV4-2/G/STARD3SPDAS213A_CHIR99021/20230626_MCF7STARD3SPDAS213A_GSK3i_5_SR_w1SPI 491 GFP.TIF]

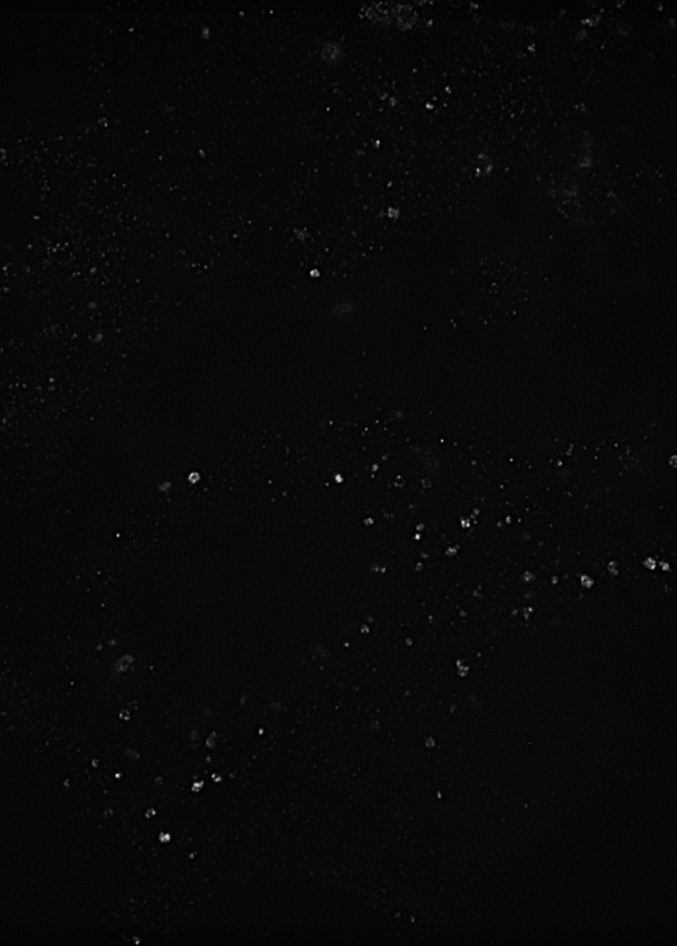

Supplement: Supplementary file 24 — Figure EV4-2 Source Data [file 44318_2026_705_MOESM24_ESM.zip › Figure EV4-2/G/STARD3SPDAS213A_CHIR99021/20230626_MCF7STARD3SPDAS213A_GSK3i_5_SR_w2SPI 561 mCherry.TIF]

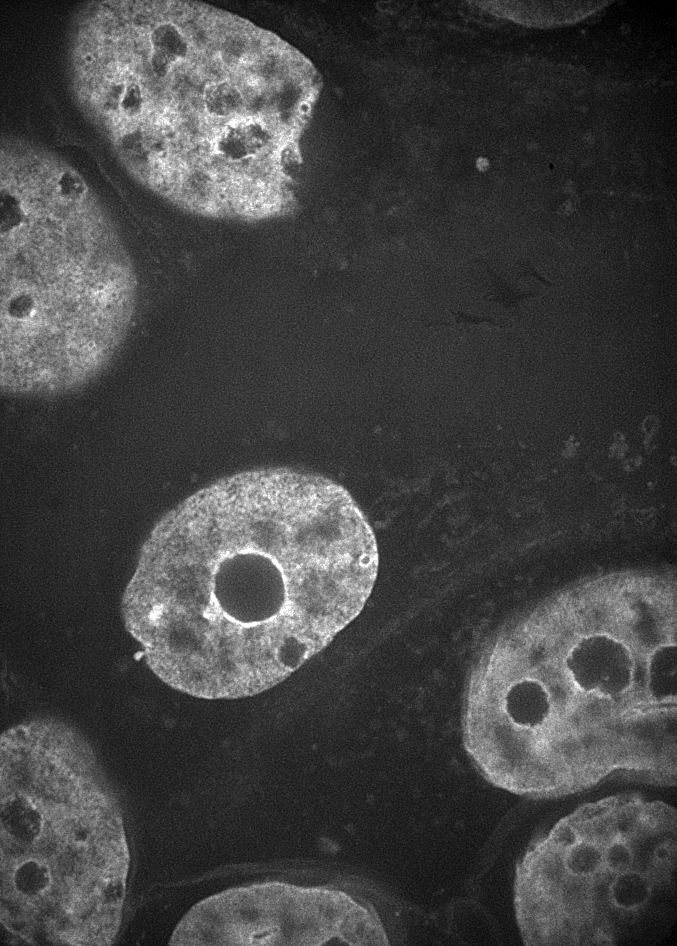

Supplement: Supplementary file 24 — Figure EV4-2 Source Data [file 44318_2026_705_MOESM24_ESM.zip › Figure EV4-2/G/STARD3SPDAS213A_CHIR99021/20230626_MCF7STARD3SPDAS213A_GSK3i_5_SR_w3SPI 405 DAPI.TIF]

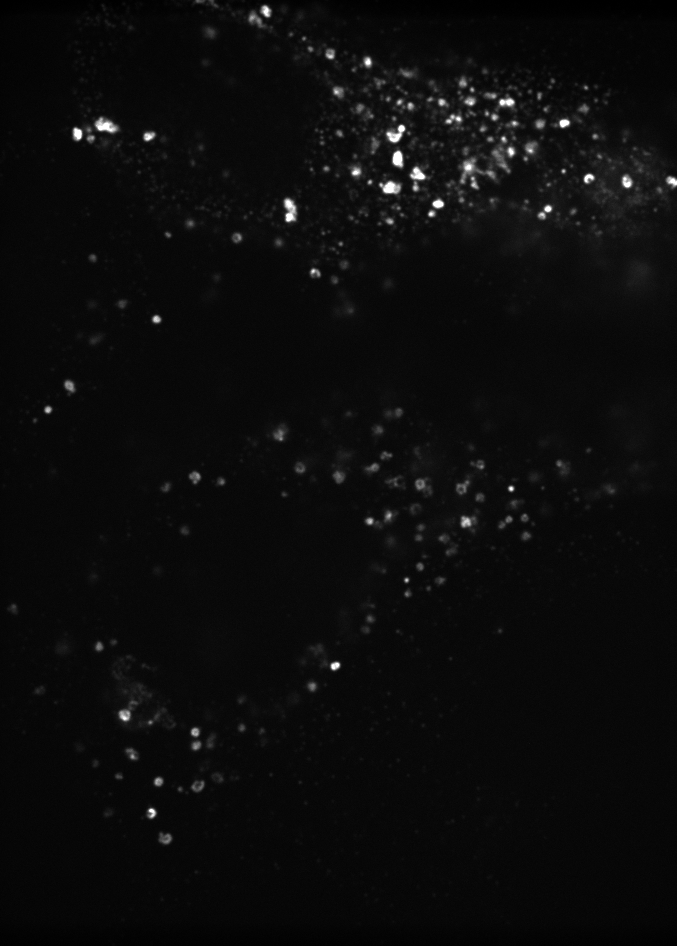

Supplement: Supplementary file 24 — Figure EV4-2 Source Data [file 44318_2026_705_MOESM24_ESM.zip › Figure EV4-2/G/STARD3SPDAS213A_CHIR99021/20230626_MCF7STARD3SPDAS213A_GSK3i_5_w1SPI 491 GFP.TIF]

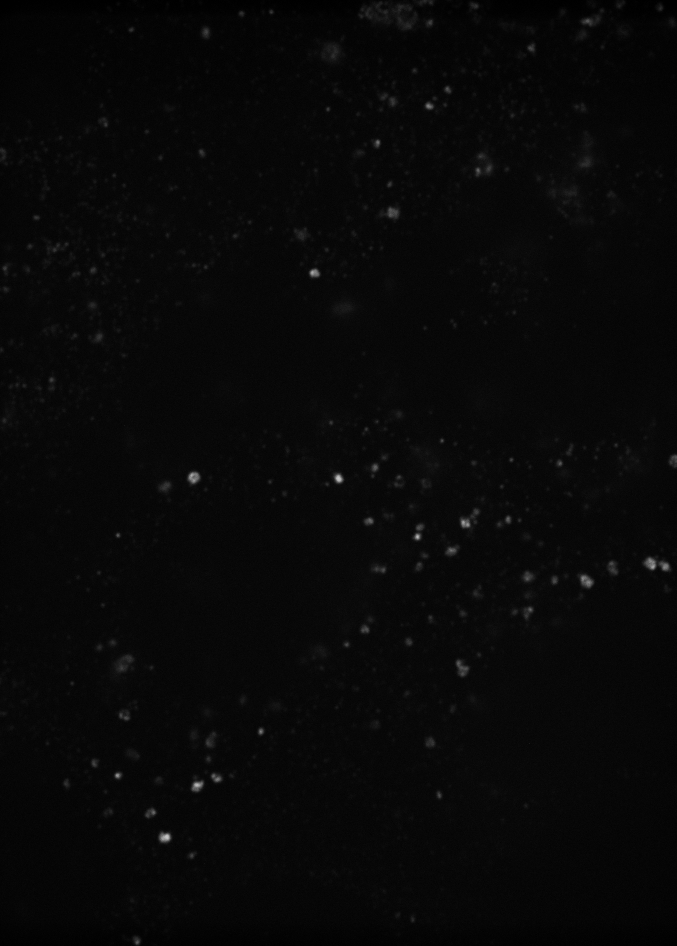

Supplement: Supplementary file 24 — Figure EV4-2 Source Data [file 44318_2026_705_MOESM24_ESM.zip › Figure EV4-2/G/STARD3SPDAS213A_CHIR99021/20230626_MCF7STARD3SPDAS213A_GSK3i_5_w2SPI 561 mCherry.TIF]

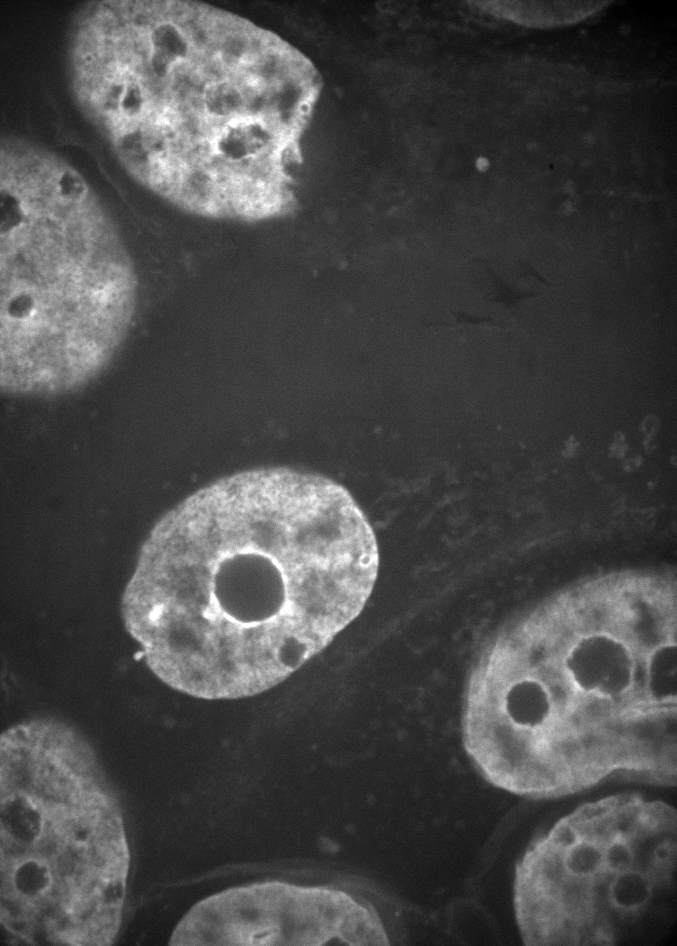

Supplement: Supplementary file 24 — Figure EV4-2 Source Data [file 44318_2026_705_MOESM24_ESM.zip › Figure EV4-2/G/STARD3SPDAS213A_CHIR99021/20230626_MCF7STARD3SPDAS213A_GSK3i_5_w3SPI 405 DAPI.TIF]

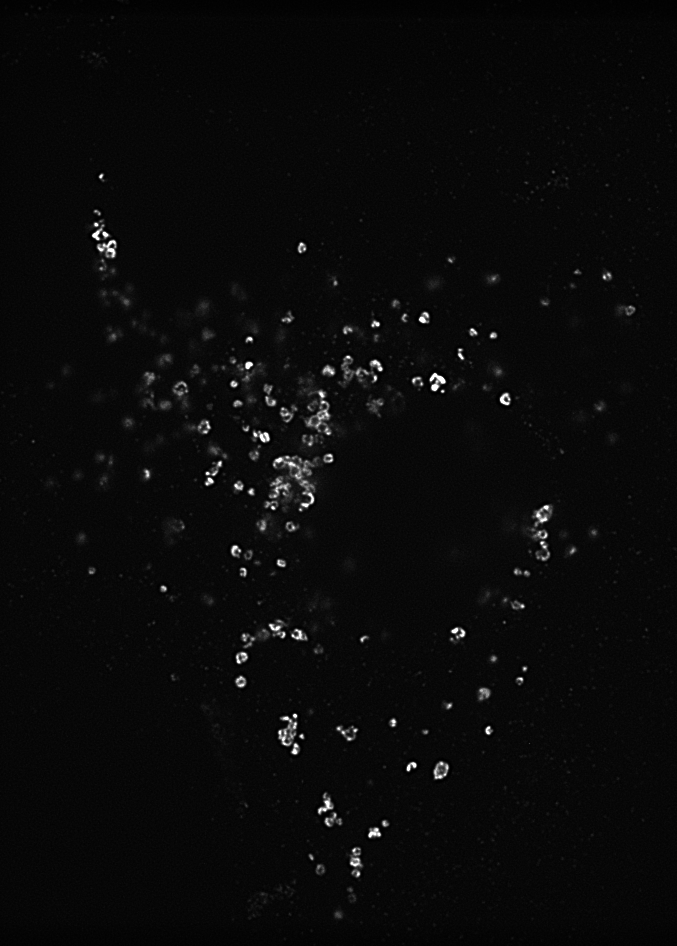

Supplement: Supplementary file 24 — Figure EV4-2 Source Data [file 44318_2026_705_MOESM24_ESM.zip › Figure EV4-2/G/STARD3SPDAS213A_NT/20230626_MCF7STARD3SPDAS213A_2_SR_w1SPI 491 GFP.TIF]

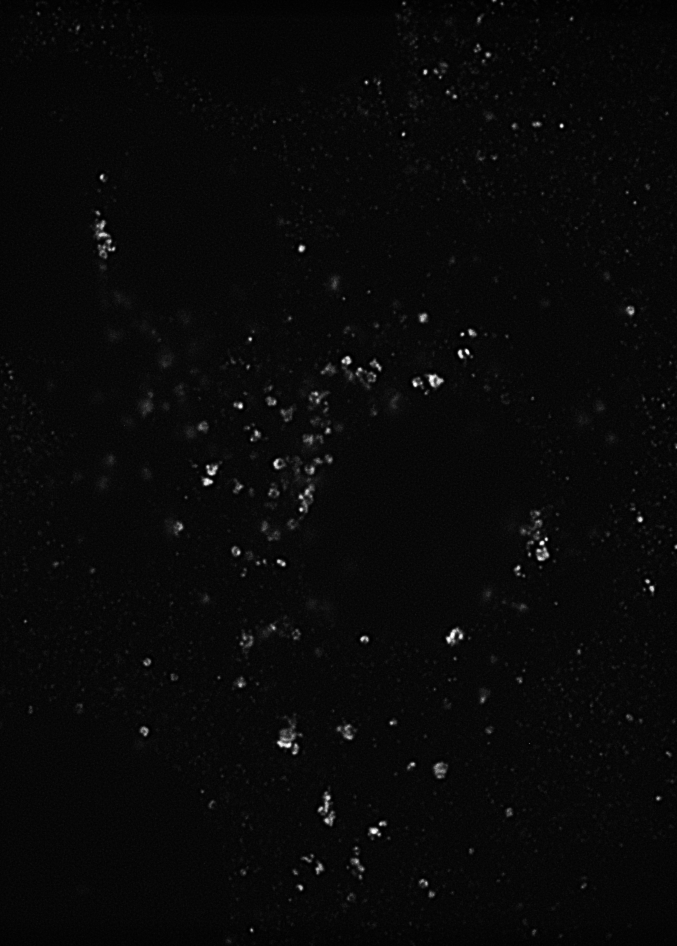

Supplement: Supplementary file 24 — Figure EV4-2 Source Data [file 44318_2026_705_MOESM24_ESM.zip › Figure EV4-2/G/STARD3SPDAS213A_NT/20230626_MCF7STARD3SPDAS213A_2_SR_w2SPI 561 mCherry.TIF]

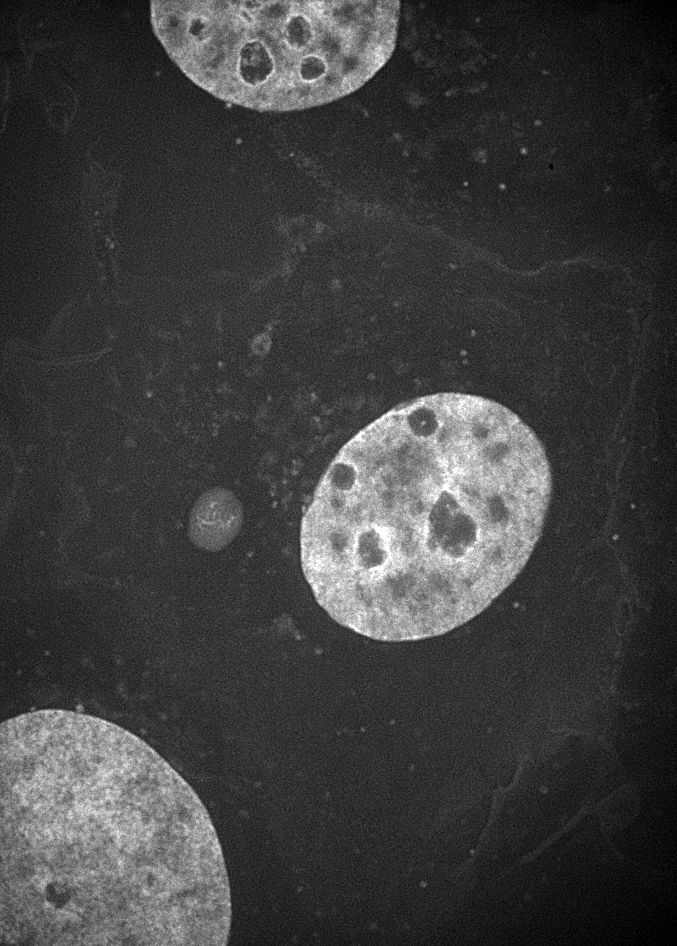

Supplement: Supplementary file 24 — Figure EV4-2 Source Data [file 44318_2026_705_MOESM24_ESM.zip › Figure EV4-2/G/STARD3SPDAS213A_NT/20230626_MCF7STARD3SPDAS213A_2_SR_w3SPI 405 DAPI.TIF]

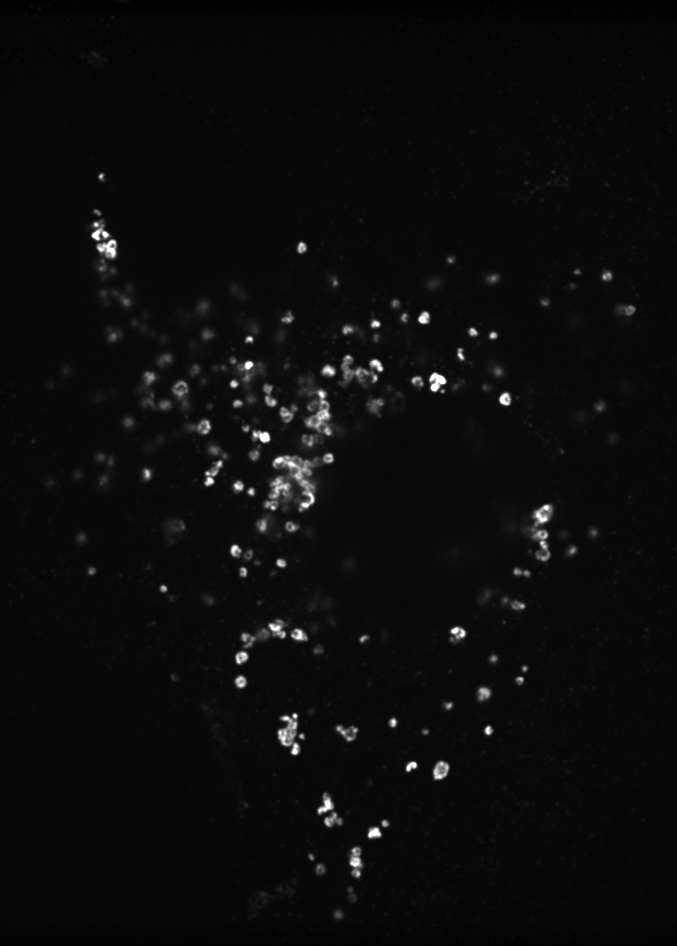

Supplement: Supplementary file 24 — Figure EV4-2 Source Data [file 44318_2026_705_MOESM24_ESM.zip › Figure EV4-2/G/STARD3SPDAS213A_NT/20230626_MCF7STARD3SPDAS213A_2_w1SPI 491 GFP.TIF]

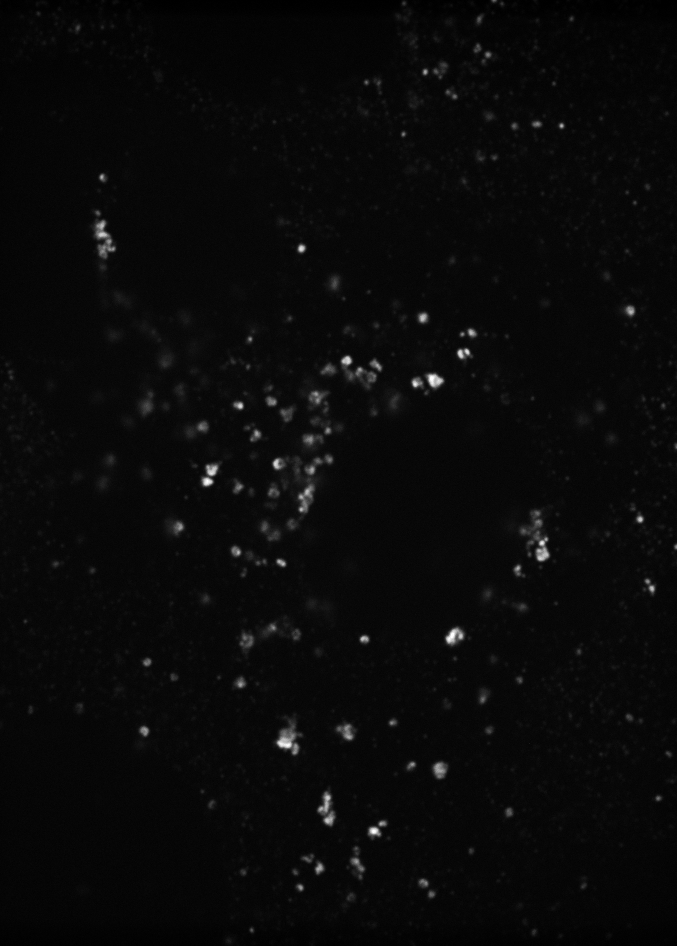

Supplement: Supplementary file 24 — Figure EV4-2 Source Data [file 44318_2026_705_MOESM24_ESM.zip › Figure EV4-2/G/STARD3SPDAS213A_NT/20230626_MCF7STARD3SPDAS213A_2_w2SPI 561 mCherry.TIF]

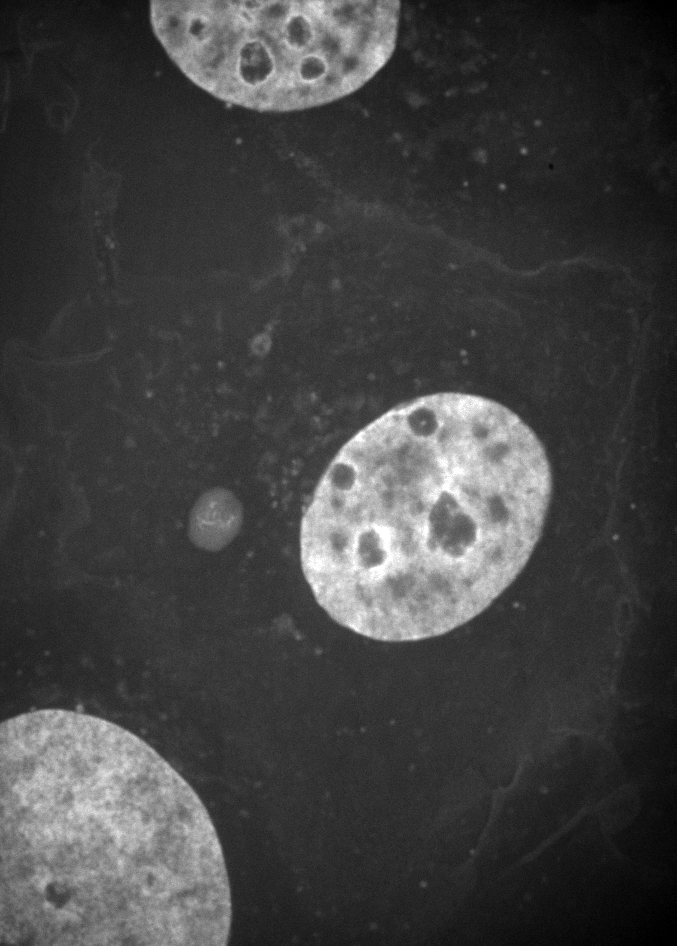

Supplement: Supplementary file 24 — Figure EV4-2 Source Data [file 44318_2026_705_MOESM24_ESM.zip › Figure EV4-2/G/STARD3SPDAS213A_NT/20230626_MCF7STARD3SPDAS213A_2_w3SPI 405 DAPI.TIF]

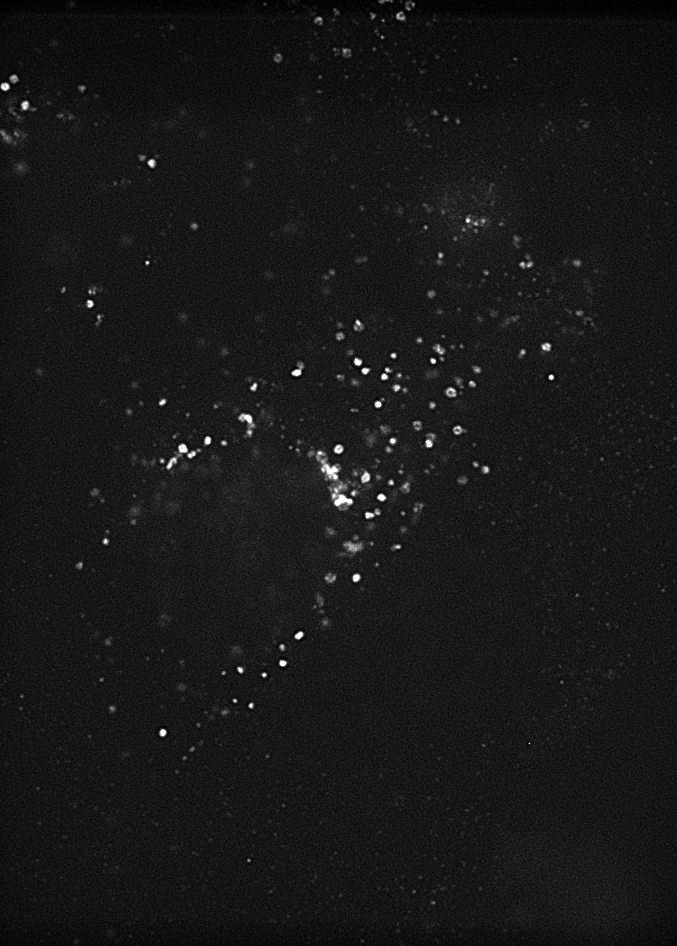

Supplement: Supplementary file 24 — Figure EV4-2 Source Data [file 44318_2026_705_MOESM24_ESM.zip › Figure EV4-2/H/STARD3S209AdeltaSTART_CHIR99021/20221104_MCF7STARD3S209AdeltaSTART_GSK3i_1_SR_w1SPI 491 GFP.TIF]

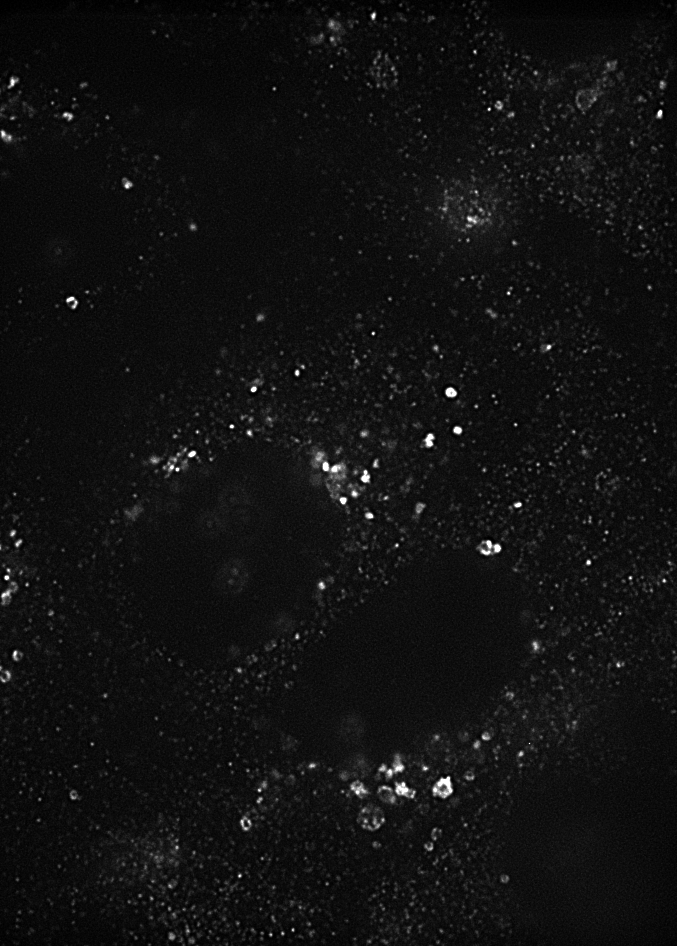

Supplement: Supplementary file 24 — Figure EV4-2 Source Data [file 44318_2026_705_MOESM24_ESM.zip › Figure EV4-2/H/STARD3S209AdeltaSTART_CHIR99021/20221104_MCF7STARD3S209AdeltaSTART_GSK3i_1_SR_w2SPI 561 mCherry.TIF]

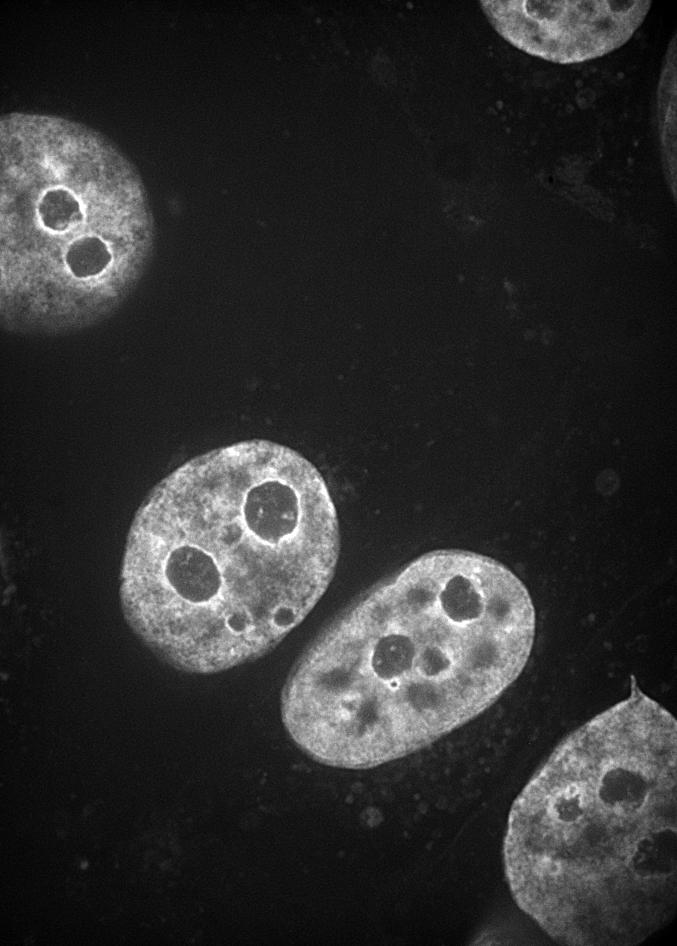

Supplement: Supplementary file 24 — Figure EV4-2 Source Data [file 44318_2026_705_MOESM24_ESM.zip › Figure EV4-2/H/STARD3S209AdeltaSTART_CHIR99021/20221104_MCF7STARD3S209AdeltaSTART_GSK3i_1_SR_w3SPI 405 DAPI.TIF]

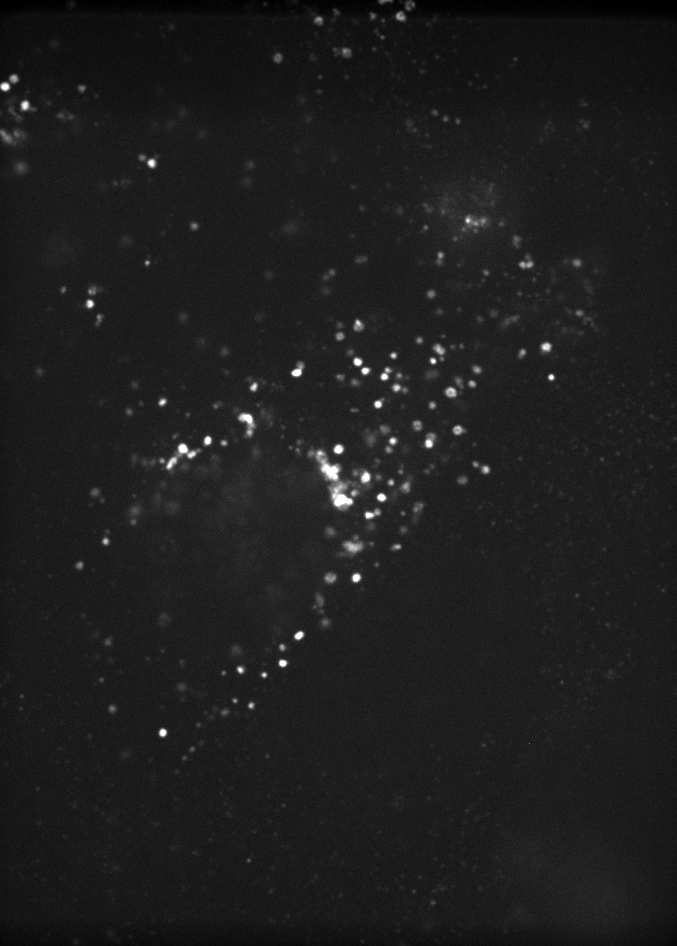

Supplement: Supplementary file 24 — Figure EV4-2 Source Data [file 44318_2026_705_MOESM24_ESM.zip › Figure EV4-2/H/STARD3S209AdeltaSTART_CHIR99021/20221104_MCF7STARD3S209AdeltaSTART_GSK3i_1_w1SPI 491 GFP.TIF]

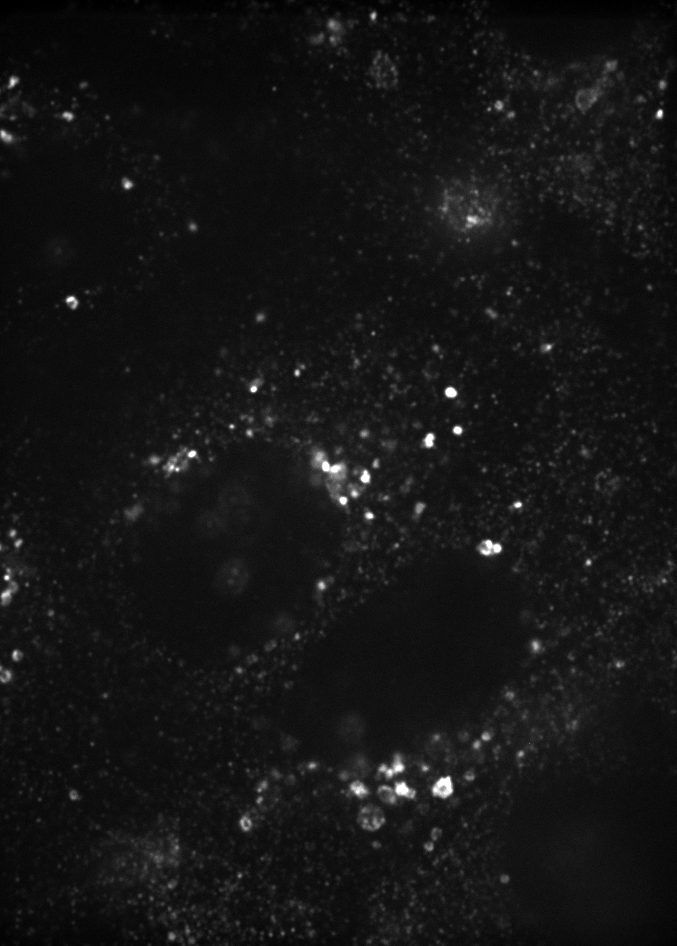

Supplement: Supplementary file 24 — Figure EV4-2 Source Data [file 44318_2026_705_MOESM24_ESM.zip › Figure EV4-2/H/STARD3S209AdeltaSTART_CHIR99021/20221104_MCF7STARD3S209AdeltaSTART_GSK3i_1_w2SPI 561 mCherry.TIF]

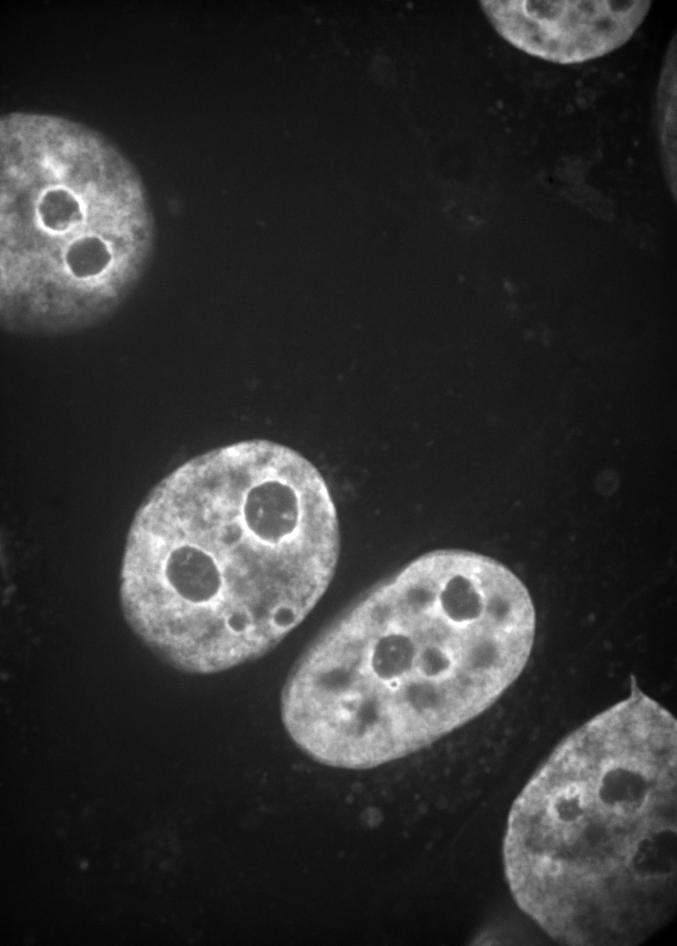

Supplement: Supplementary file 24 — Figure EV4-2 Source Data [file 44318_2026_705_MOESM24_ESM.zip › Figure EV4-2/H/STARD3S209AdeltaSTART_CHIR99021/20221104_MCF7STARD3S209AdeltaSTART_GSK3i_1_w3SPI 405 DAPI.TIF]

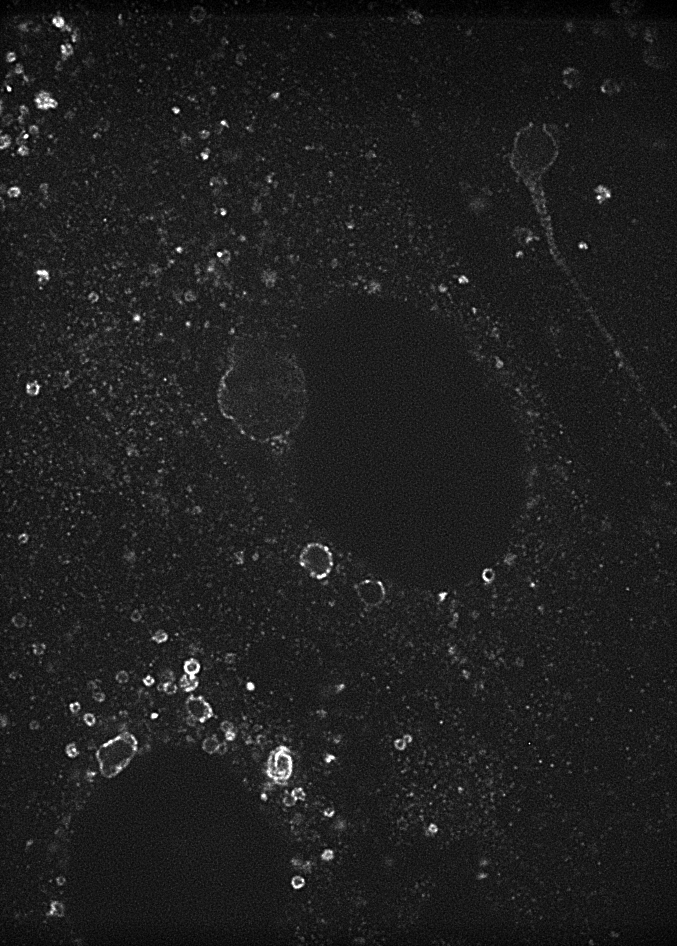

Supplement: Supplementary file 24 — Figure EV4-2 Source Data [file 44318_2026_705_MOESM24_ESM.zip › Figure EV4-2/H/STARD3S209AdeltaSTART_NT/20221212_MCF7STARD3S209AdeltaSTART_NT_1_SR_w1SPI 491 GFP.TIF]

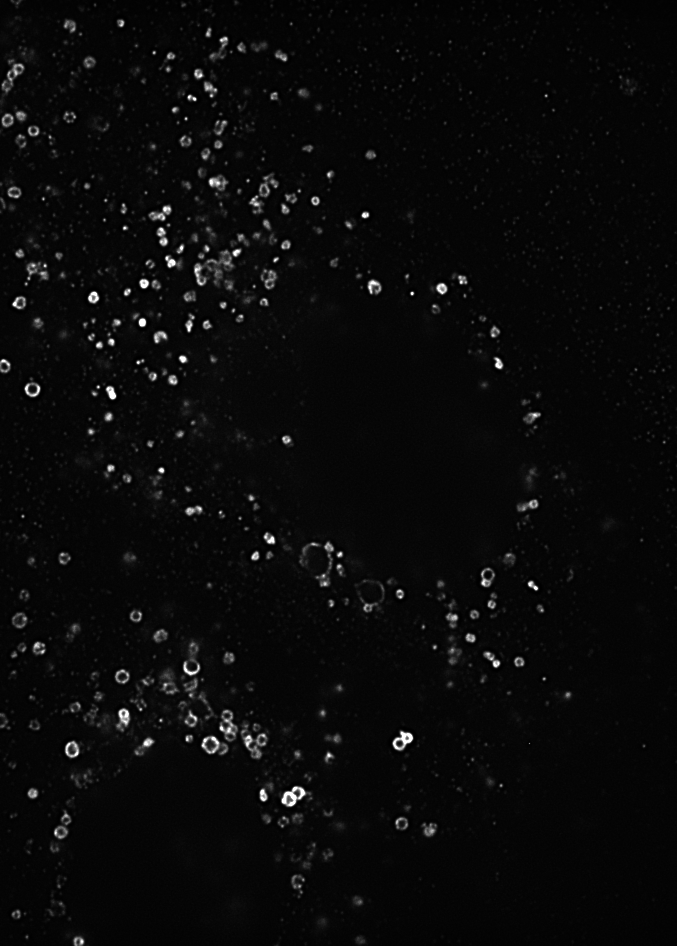

Supplement: Supplementary file 24 — Figure EV4-2 Source Data [file 44318_2026_705_MOESM24_ESM.zip › Figure EV4-2/H/STARD3S209AdeltaSTART_NT/20221212_MCF7STARD3S209AdeltaSTART_NT_1_SR_w2SPI 561 mCherry.TIF]

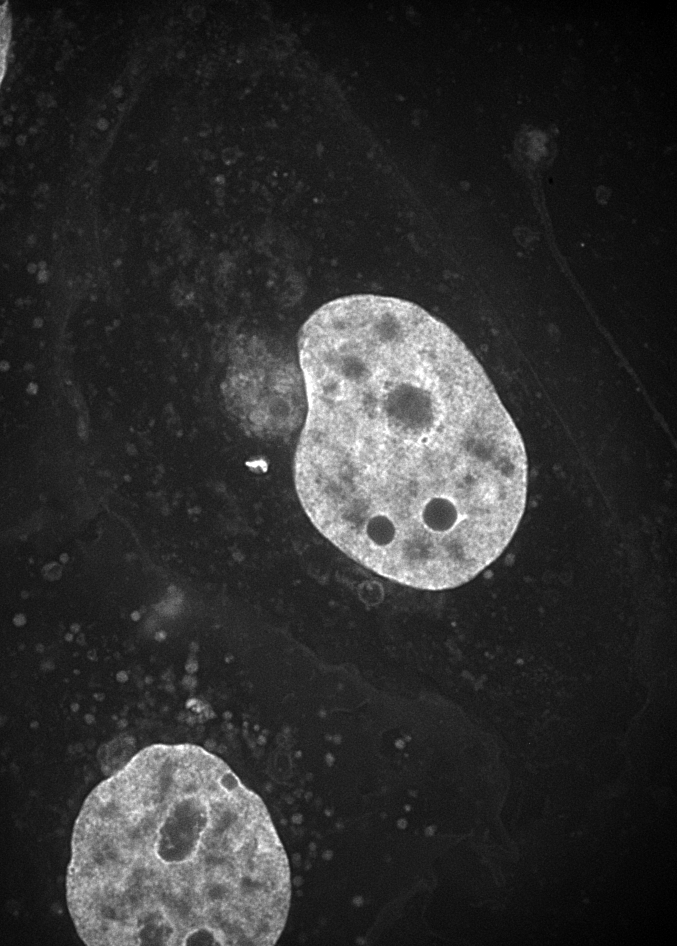

Supplement: Supplementary file 24 — Figure EV4-2 Source Data [file 44318_2026_705_MOESM24_ESM.zip › Figure EV4-2/H/STARD3S209AdeltaSTART_NT/20221212_MCF7STARD3S209AdeltaSTART_NT_1_SR_w3SPI 405 DAPI.TIF]

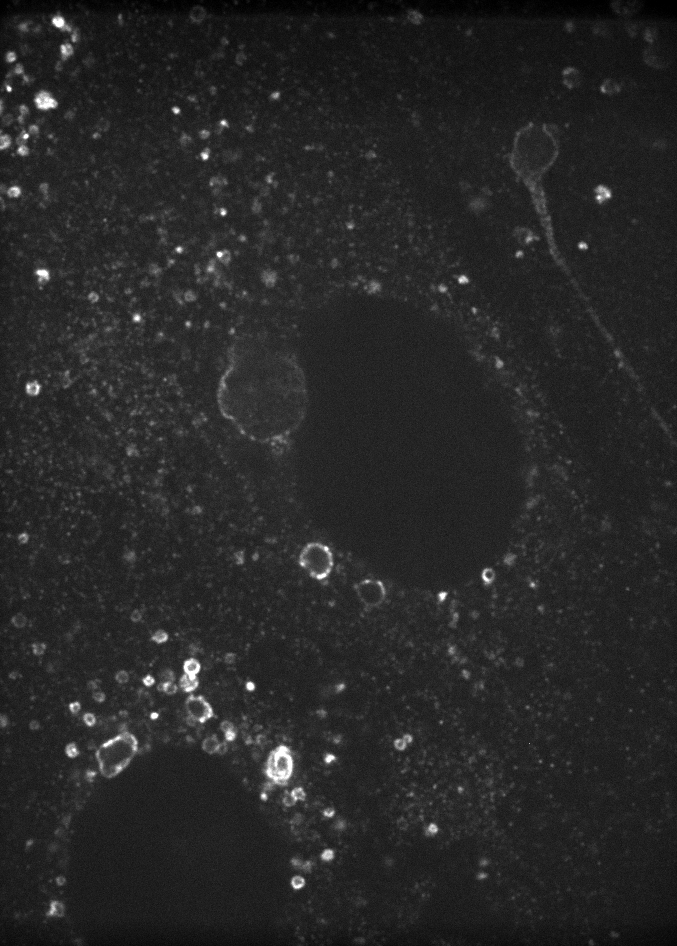

Supplement: Supplementary file 24 — Figure EV4-2 Source Data [file 44318_2026_705_MOESM24_ESM.zip › Figure EV4-2/H/STARD3S209AdeltaSTART_NT/20221212_MCF7STARD3S209AdeltaSTART_NT_1_w1SPI 491 GFP.TIF]

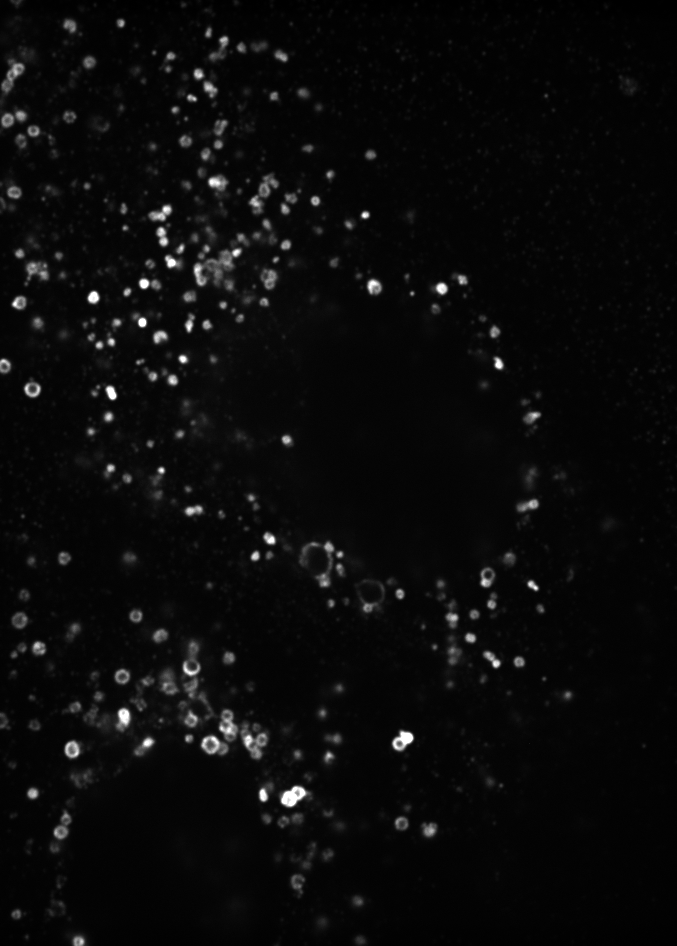

Supplement: Supplementary file 24 — Figure EV4-2 Source Data [file 44318_2026_705_MOESM24_ESM.zip › Figure EV4-2/H/STARD3S209AdeltaSTART_NT/20221212_MCF7STARD3S209AdeltaSTART_NT_1_w2SPI 561 mCherry.TIF]
