# Supplementary figures and images for: STARD3 regulates lysosome positioning and contacts via a GSK3-controlled phosphorylation switch (part 6 of 7)
Source: EMBO J. 2026 Feb 25;45(7):2239–77. doi: 10.1038/s44318-026-00705-3 (PMC13044316; doi:10.1038/s44318-026-00705-3)

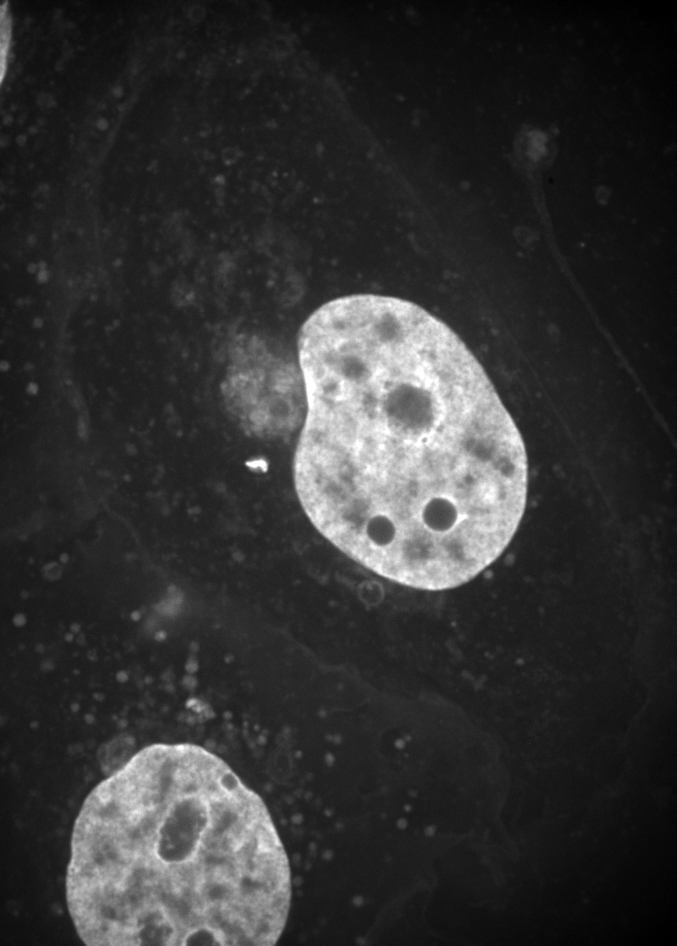

Supplement: Supplementary file 24 — Figure EV4-2 Source Data [file 44318_2026_705_MOESM24_ESM.zip › Figure EV4-2/H/STARD3S209AdeltaSTART_NT/20221212_MCF7STARD3S209AdeltaSTART_NT_1_w3SPI 405 DAPI.TIF]

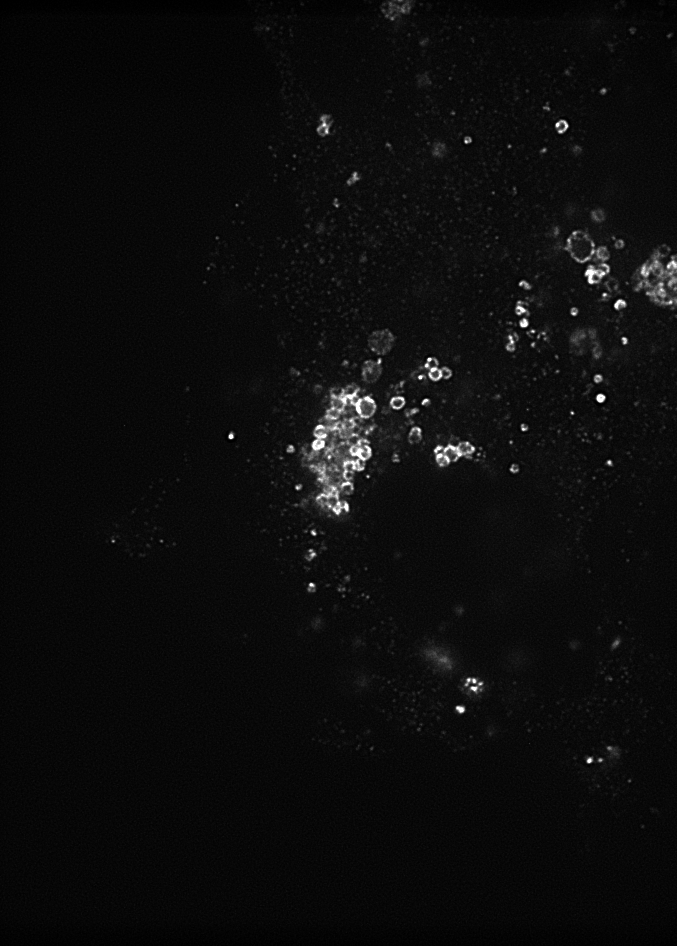

Supplement: Supplementary file 25 — Figure EV4-3 Source Data [file 44318_2026_705_MOESM25_ESM.zip › Figure EV4-3/I/MCF7_STARD3S213A/20230414_MCF7STARD3S213A_NT_5_SR_w1SPI 491 GFP.TIF]

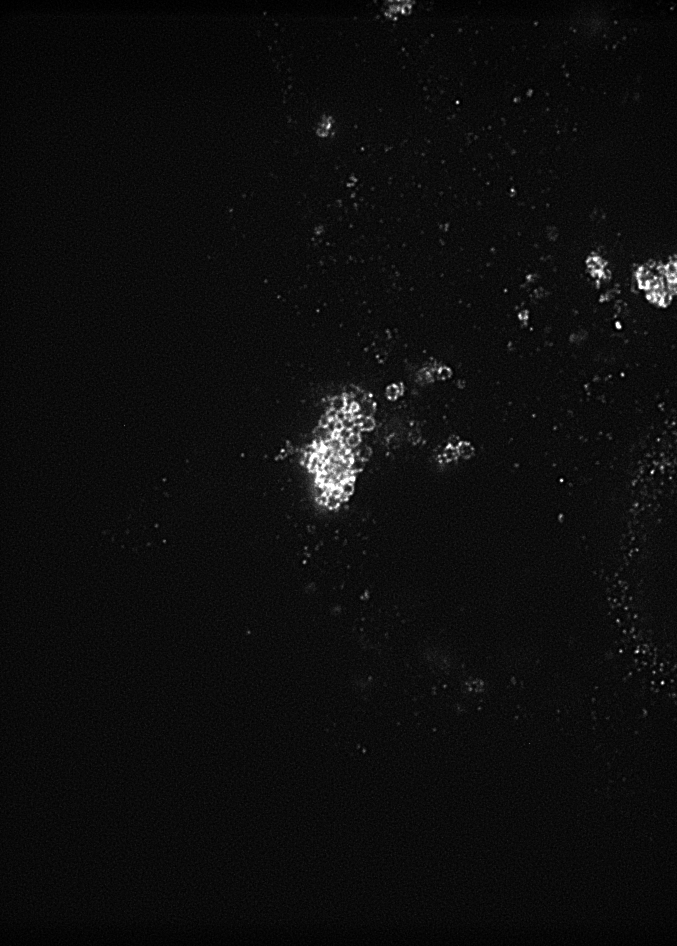

Supplement: Supplementary file 25 — Figure EV4-3 Source Data [file 44318_2026_705_MOESM25_ESM.zip › Figure EV4-3/I/MCF7_STARD3S213A/20230414_MCF7STARD3S213A_NT_5_SR_w2SPI 561 mCherry.TIF]

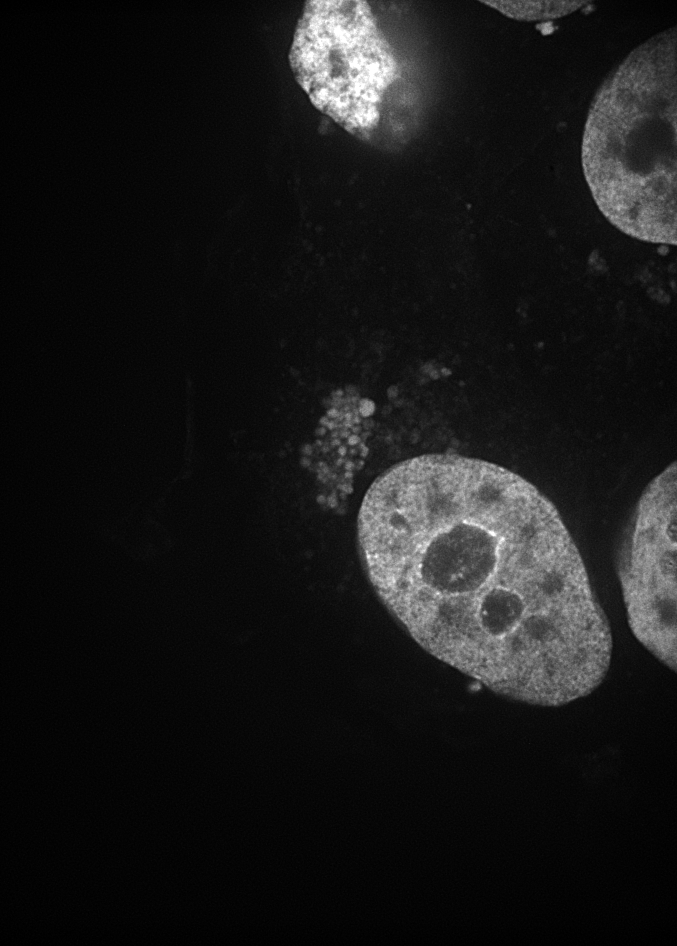

Supplement: Supplementary file 25 — Figure EV4-3 Source Data [file 44318_2026_705_MOESM25_ESM.zip › Figure EV4-3/I/MCF7_STARD3S213A/20230414_MCF7STARD3S213A_NT_5_SR_w3SPI 405 DAPI.TIF]

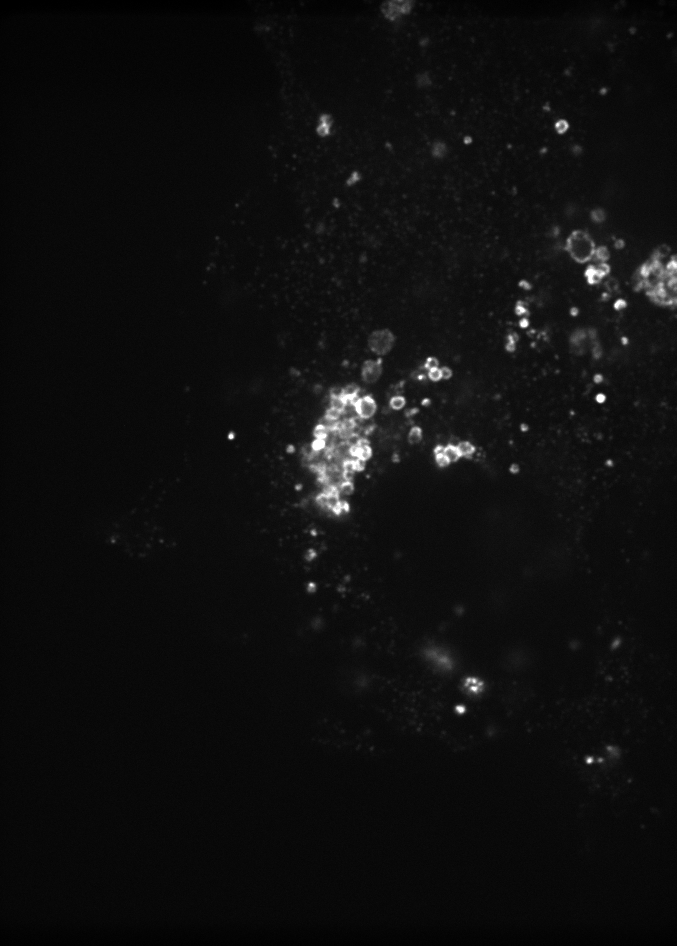

Supplement: Supplementary file 25 — Figure EV4-3 Source Data [file 44318_2026_705_MOESM25_ESM.zip › Figure EV4-3/I/MCF7_STARD3S213A/20230414_MCF7STARD3S213A_NT_5_w1SPI 491 GFP.TIF]

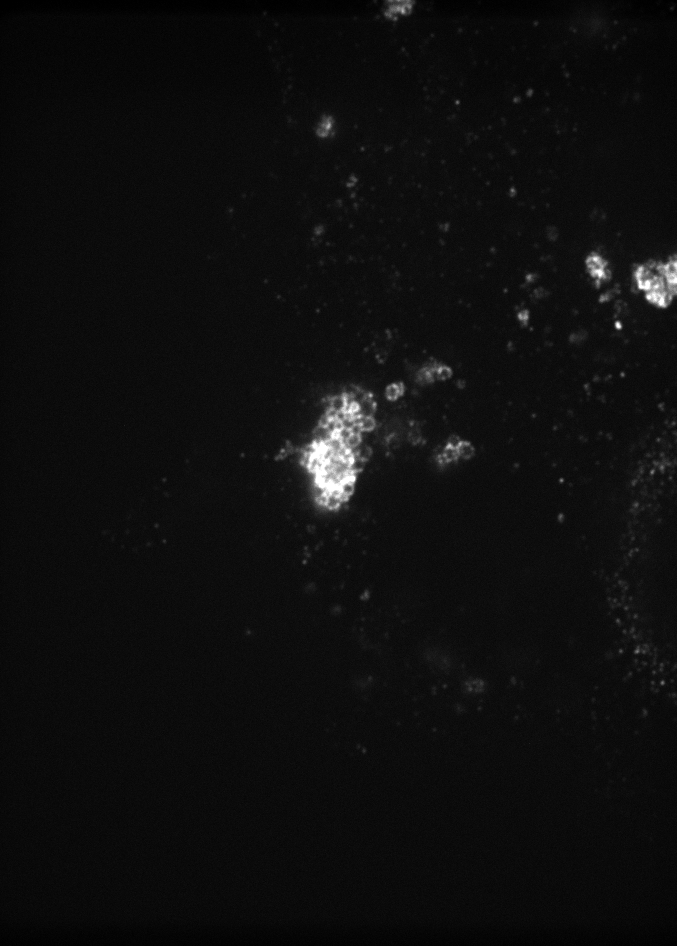

Supplement: Supplementary file 25 — Figure EV4-3 Source Data [file 44318_2026_705_MOESM25_ESM.zip › Figure EV4-3/I/MCF7_STARD3S213A/20230414_MCF7STARD3S213A_NT_5_w2SPI 561 mCherry.TIF]

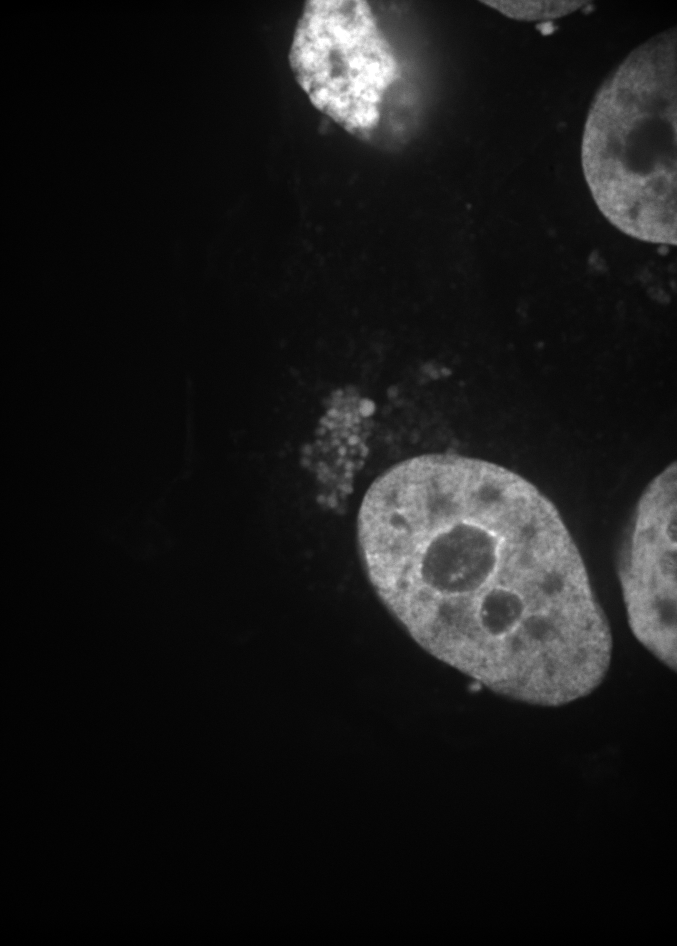

Supplement: Supplementary file 25 — Figure EV4-3 Source Data [file 44318_2026_705_MOESM25_ESM.zip › Figure EV4-3/I/MCF7_STARD3S213A/20230414_MCF7STARD3S213A_NT_5_w3SPI 405 DAPI.TIF]

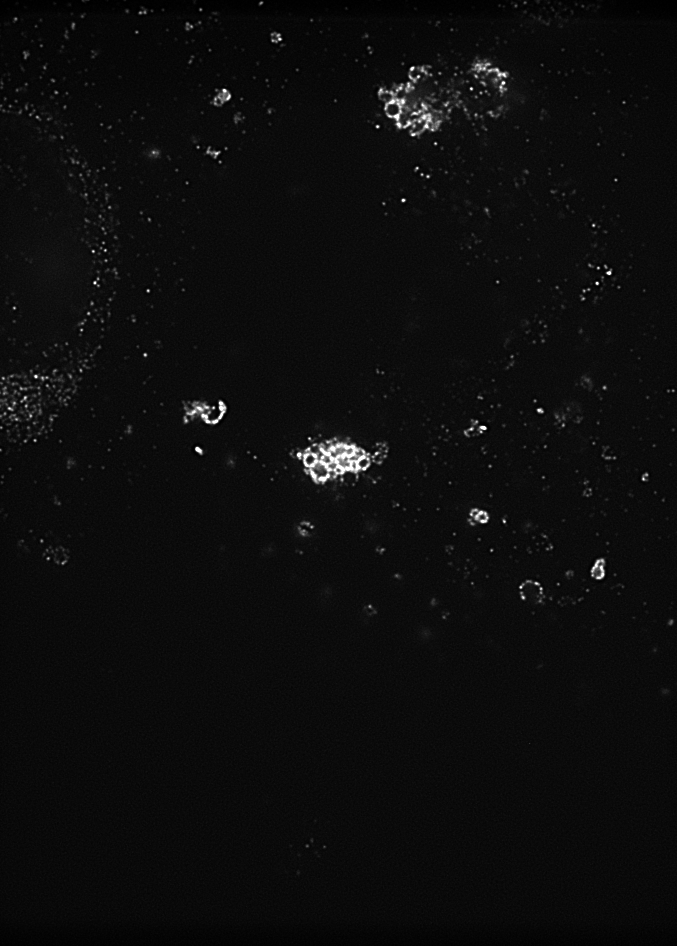

Supplement: Supplementary file 25 — Figure EV4-3 Source Data [file 44318_2026_705_MOESM25_ESM.zip › Figure EV4-3/I/MCF7_STARD3S213AS217AS221A/20230127_MCF7_STARD3S3A_NT_2_SR_w1SPI 491 GFP.TIF]

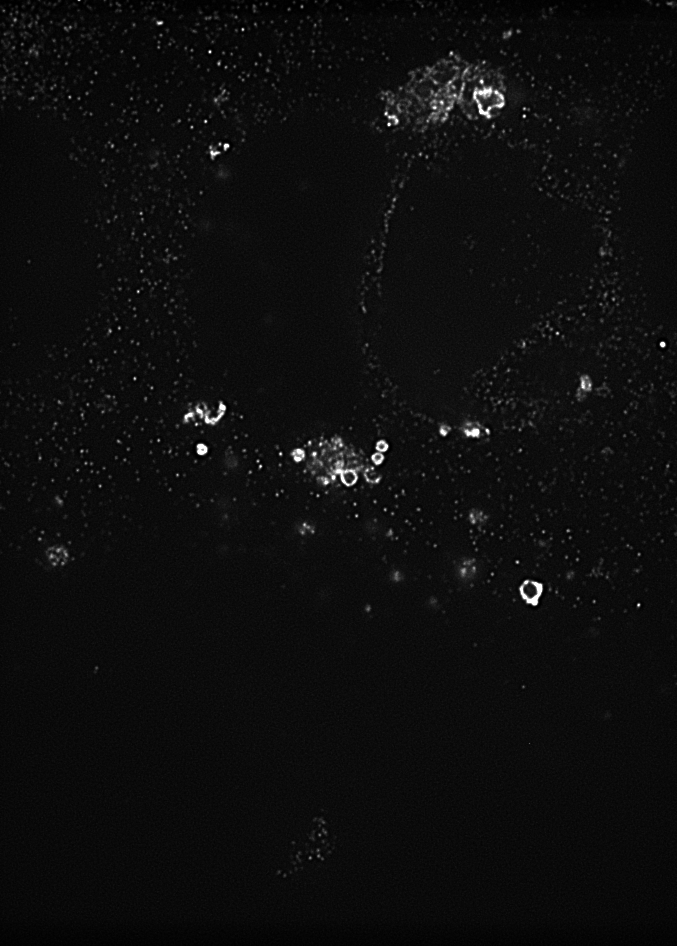

Supplement: Supplementary file 25 — Figure EV4-3 Source Data [file 44318_2026_705_MOESM25_ESM.zip › Figure EV4-3/I/MCF7_STARD3S213AS217AS221A/20230127_MCF7_STARD3S3A_NT_2_SR_w2SPI 561 mCherry.TIF]

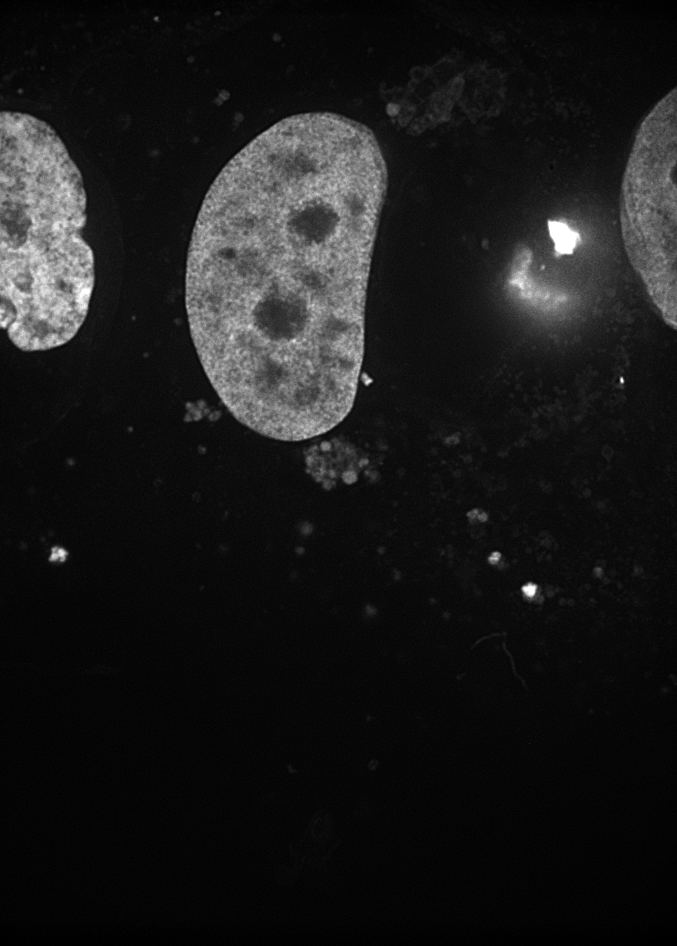

Supplement: Supplementary file 25 — Figure EV4-3 Source Data [file 44318_2026_705_MOESM25_ESM.zip › Figure EV4-3/I/MCF7_STARD3S213AS217AS221A/20230127_MCF7_STARD3S3A_NT_2_SR_w3SPI 405 DAPI.TIF]

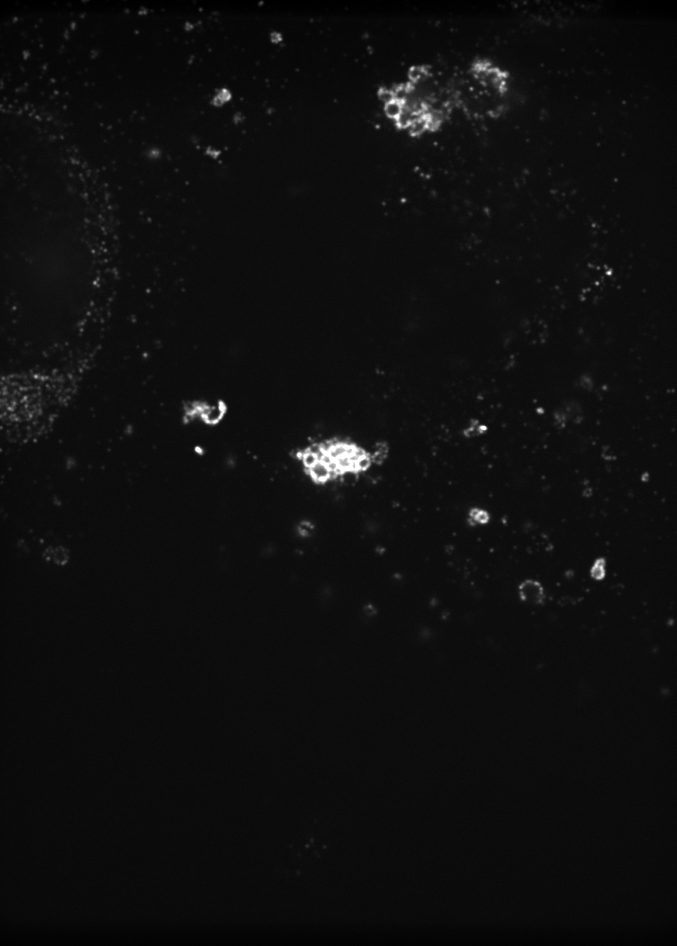

Supplement: Supplementary file 25 — Figure EV4-3 Source Data [file 44318_2026_705_MOESM25_ESM.zip › Figure EV4-3/I/MCF7_STARD3S213AS217AS221A/20230127_MCF7_STARD3S3A_NT_2_w1SPI 491 GFP.TIF]

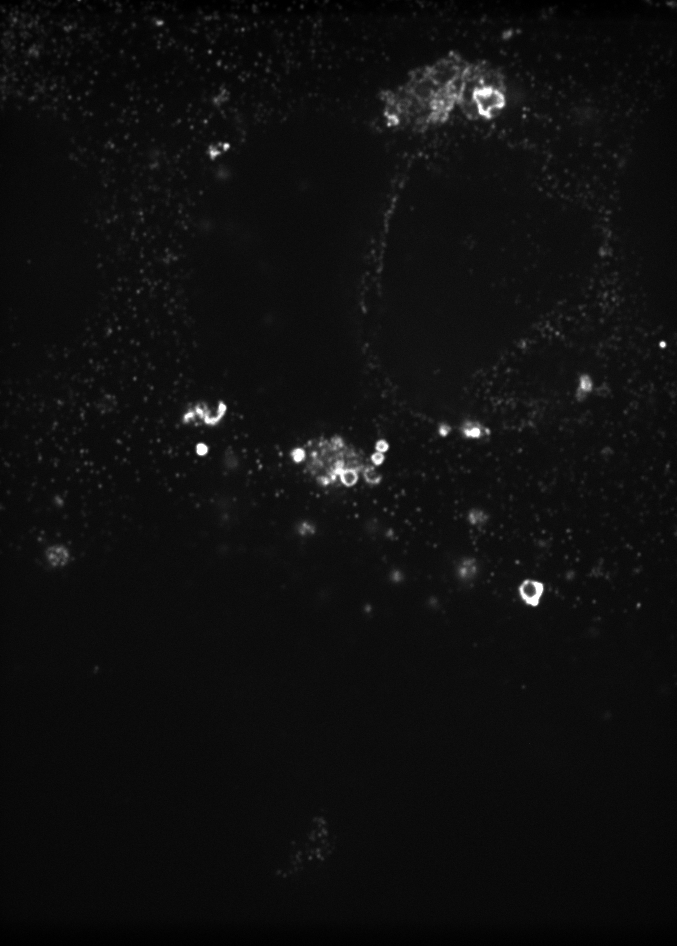

Supplement: Supplementary file 25 — Figure EV4-3 Source Data [file 44318_2026_705_MOESM25_ESM.zip › Figure EV4-3/I/MCF7_STARD3S213AS217AS221A/20230127_MCF7_STARD3S3A_NT_2_w2SPI 561 mCherry.TIF]

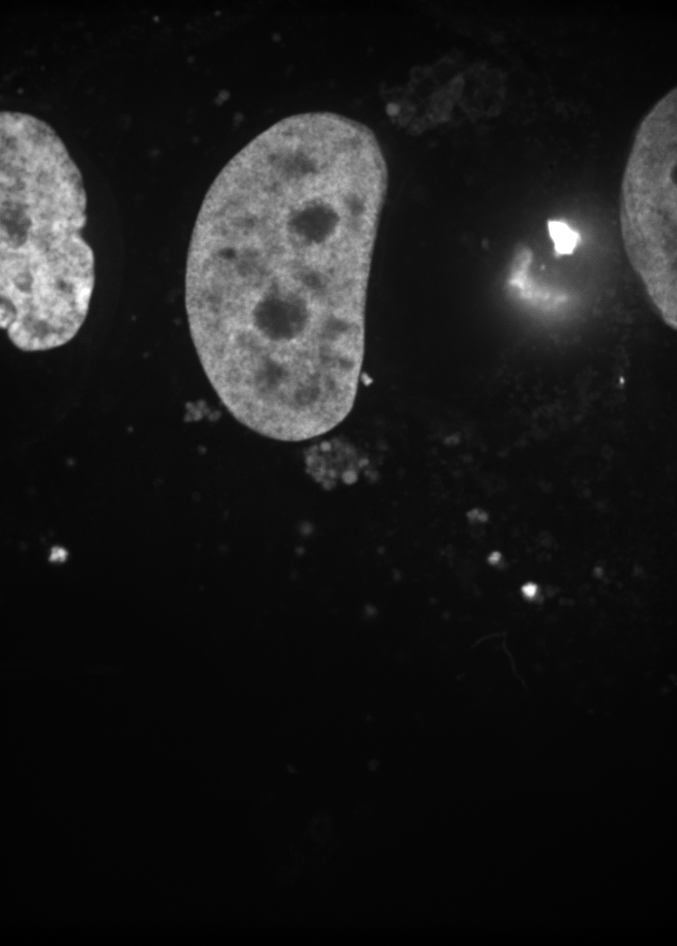

Supplement: Supplementary file 25 — Figure EV4-3 Source Data [file 44318_2026_705_MOESM25_ESM.zip › Figure EV4-3/I/MCF7_STARD3S213AS217AS221A/20230127_MCF7_STARD3S3A_NT_2_w3SPI 405 DAPI.TIF]

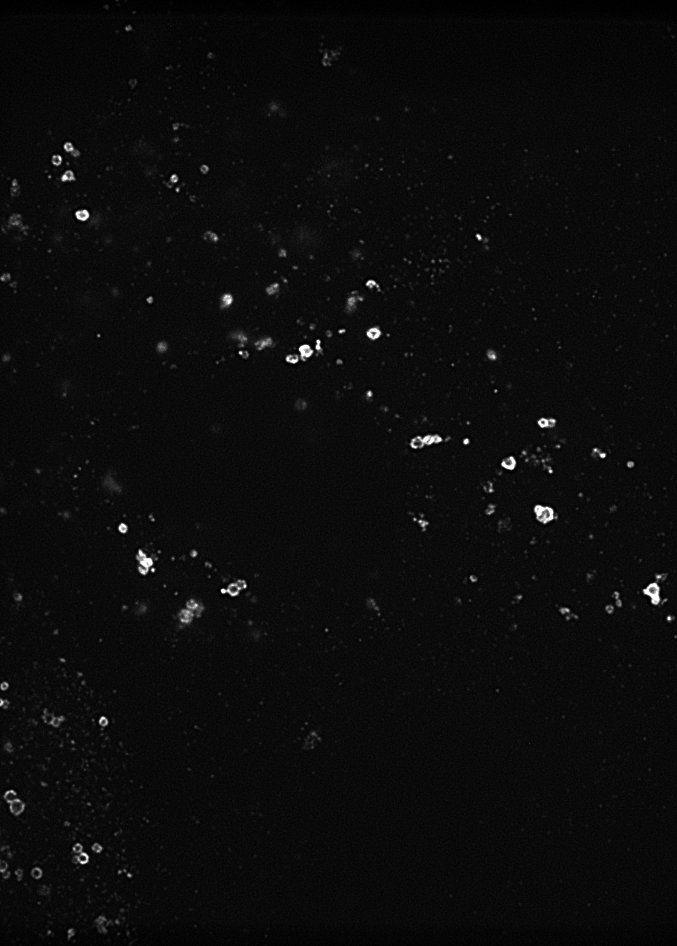

Supplement: Supplementary file 25 — Figure EV4-3 Source Data [file 44318_2026_705_MOESM25_ESM.zip › Figure EV4-3/I/MCF7_STARD3S217A/20230428_MCF7STARD3217A_NT_3_SR_w1SPI 491 GFP.TIF]

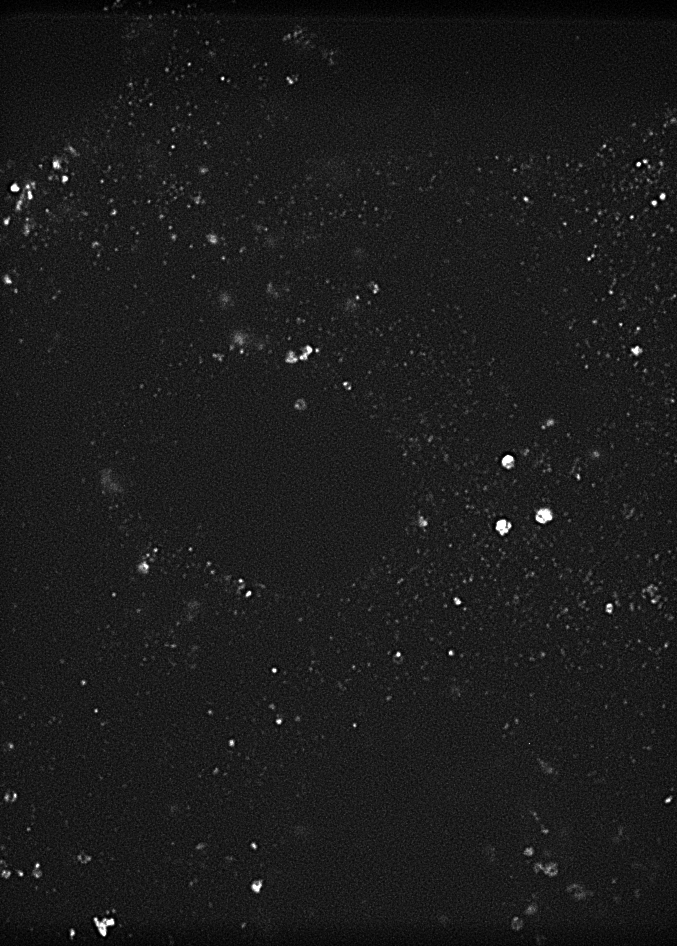

Supplement: Supplementary file 25 — Figure EV4-3 Source Data [file 44318_2026_705_MOESM25_ESM.zip › Figure EV4-3/I/MCF7_STARD3S217A/20230428_MCF7STARD3217A_NT_3_SR_w2SPI 561 mCherry.TIF]

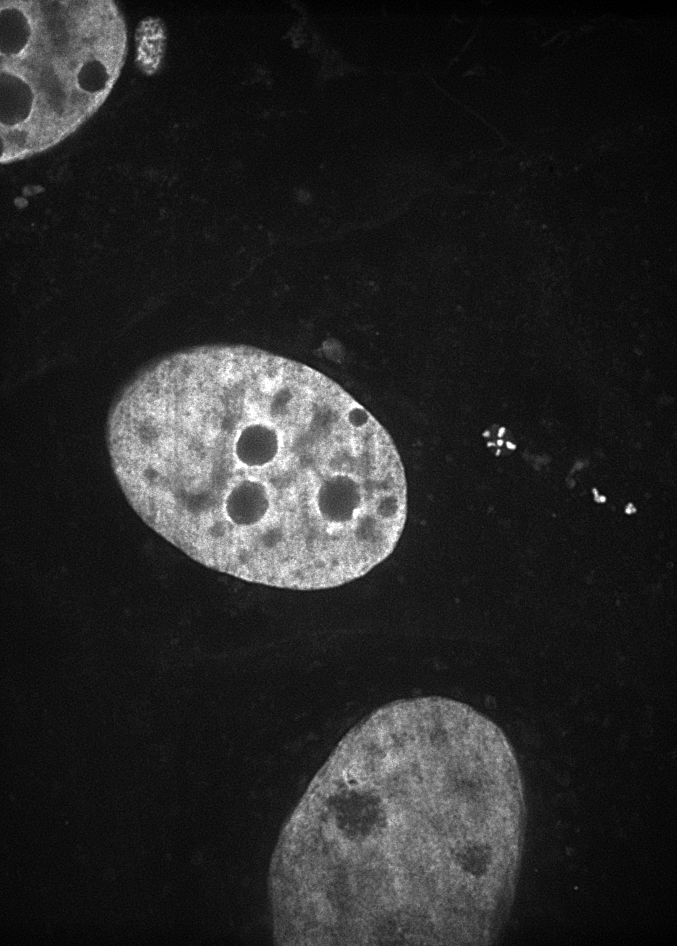

Supplement: Supplementary file 25 — Figure EV4-3 Source Data [file 44318_2026_705_MOESM25_ESM.zip › Figure EV4-3/I/MCF7_STARD3S217A/20230428_MCF7STARD3217A_NT_3_SR_w3SPI 405 DAPI.TIF]

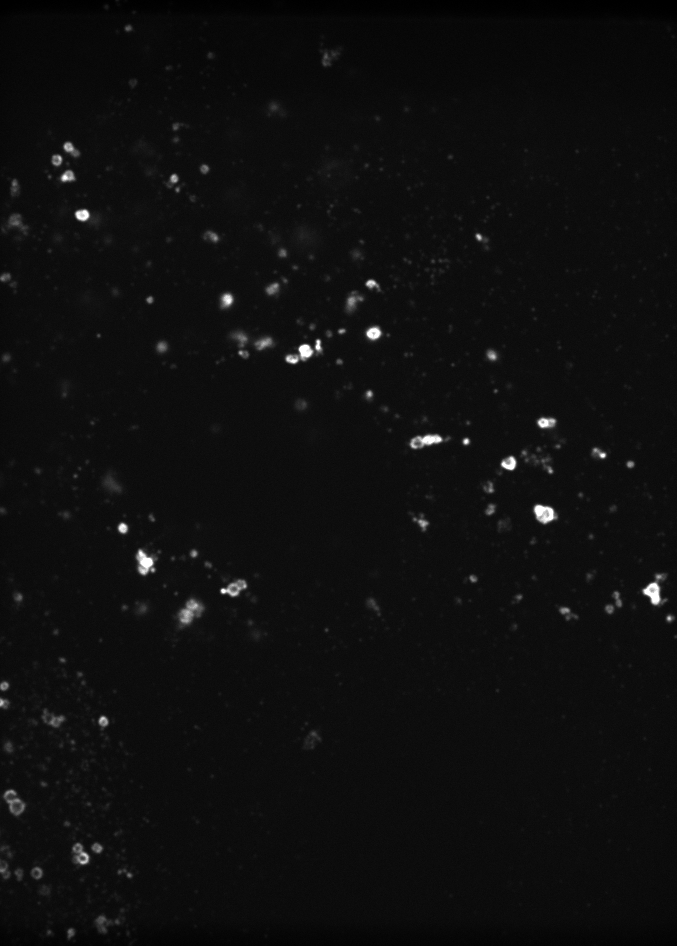

Supplement: Supplementary file 25 — Figure EV4-3 Source Data [file 44318_2026_705_MOESM25_ESM.zip › Figure EV4-3/I/MCF7_STARD3S217A/20230428_MCF7STARD3217A_NT_3_w1SPI 491 GFP.TIF]

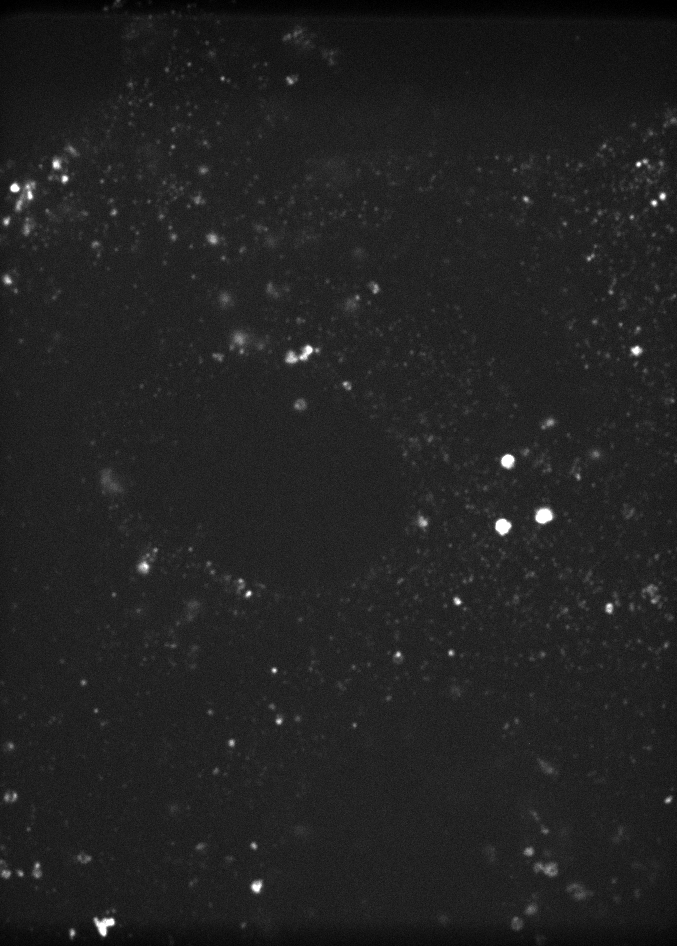

Supplement: Supplementary file 25 — Figure EV4-3 Source Data [file 44318_2026_705_MOESM25_ESM.zip › Figure EV4-3/I/MCF7_STARD3S217A/20230428_MCF7STARD3217A_NT_3_w2SPI 561 mCherry.TIF]

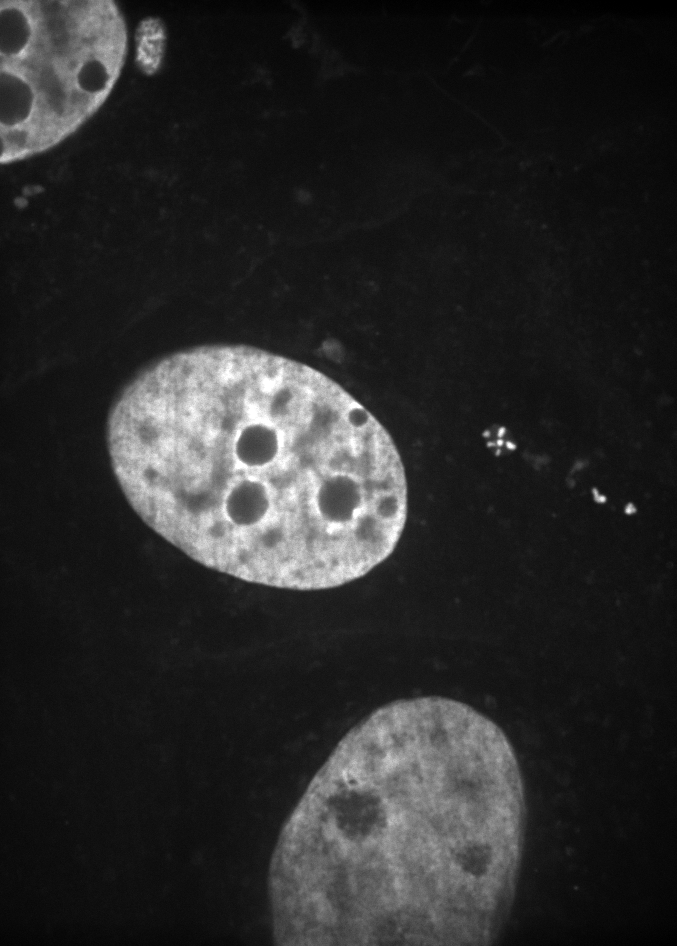

Supplement: Supplementary file 25 — Figure EV4-3 Source Data [file 44318_2026_705_MOESM25_ESM.zip › Figure EV4-3/I/MCF7_STARD3S217A/20230428_MCF7STARD3217A_NT_3_w3SPI 405 DAPI.TIF]

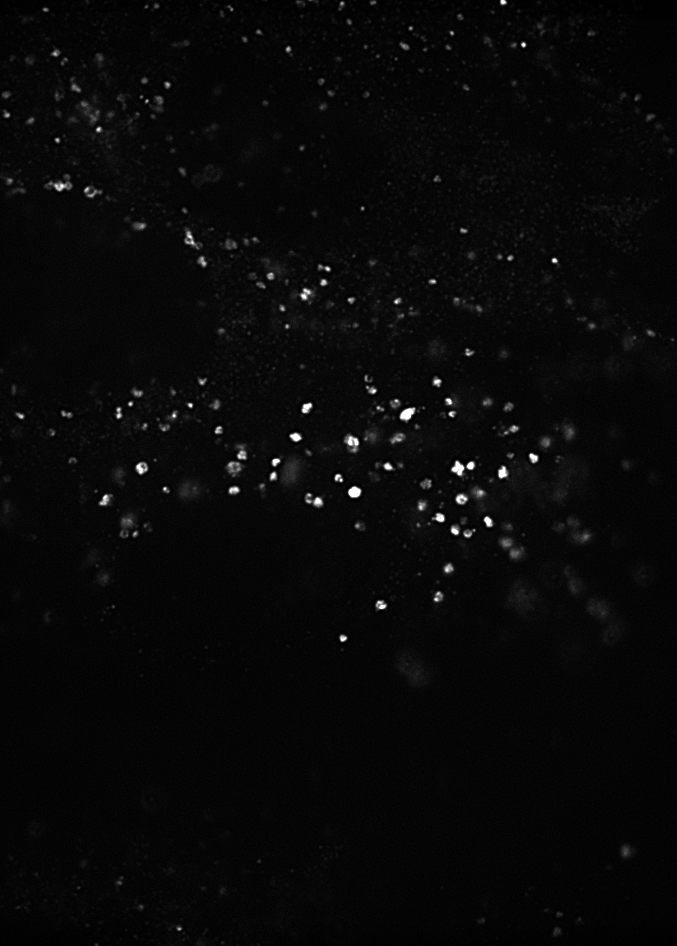

Supplement: Supplementary file 25 — Figure EV4-3 Source Data [file 44318_2026_705_MOESM25_ESM.zip › Figure EV4-3/I/MCF7_STARD3S221A/20230414_MCF7STARD3S221A_NT_2_SR_w1SPI 491 GFP.TIF]

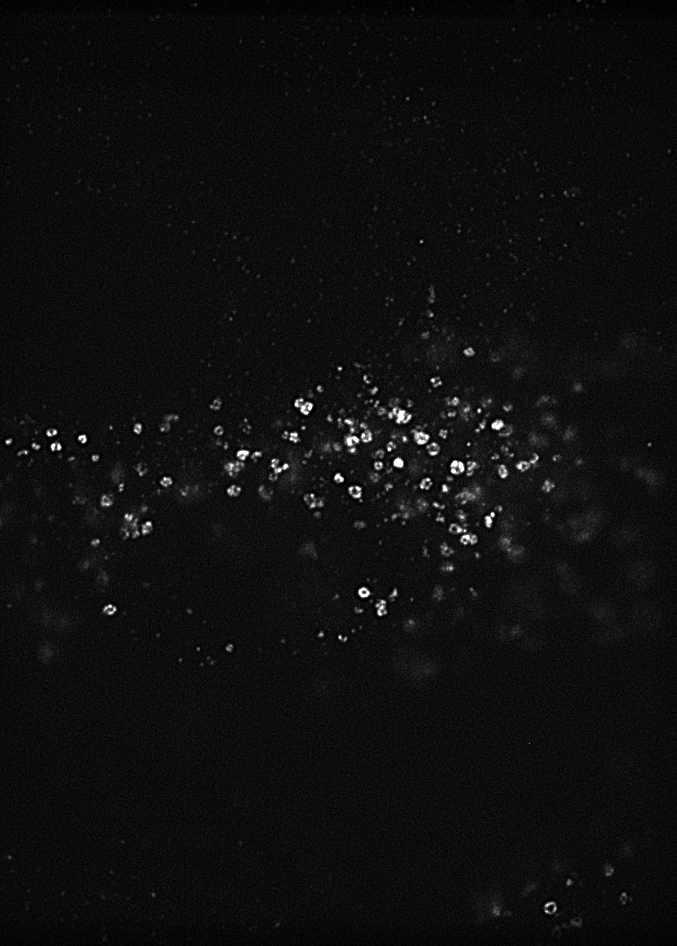

Supplement: Supplementary file 25 — Figure EV4-3 Source Data [file 44318_2026_705_MOESM25_ESM.zip › Figure EV4-3/I/MCF7_STARD3S221A/20230414_MCF7STARD3S221A_NT_2_SR_w2SPI 561 mCherry.TIF]

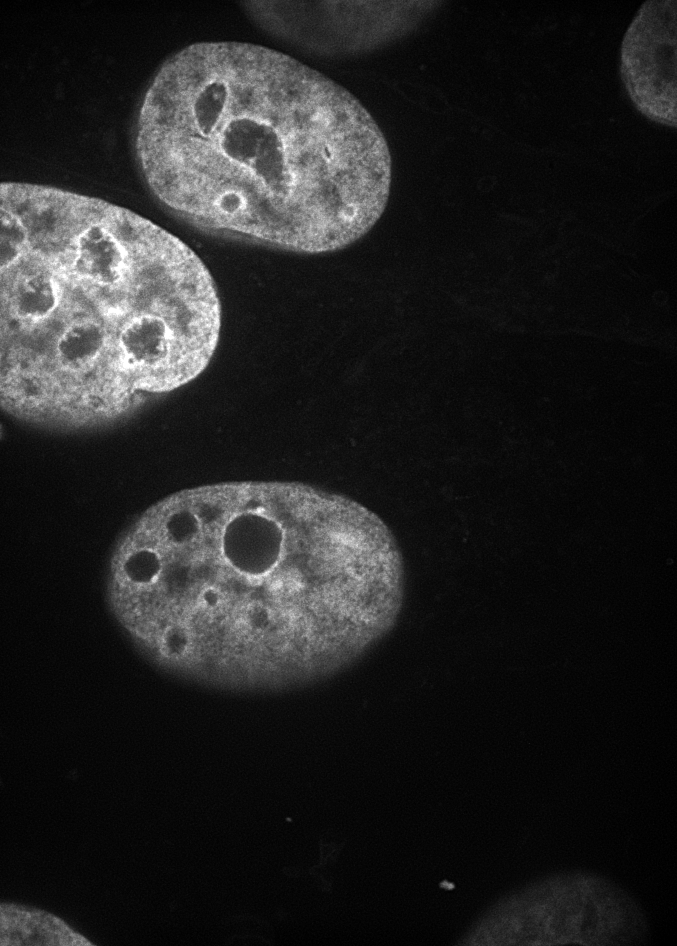

Supplement: Supplementary file 25 — Figure EV4-3 Source Data [file 44318_2026_705_MOESM25_ESM.zip › Figure EV4-3/I/MCF7_STARD3S221A/20230414_MCF7STARD3S221A_NT_2_SR_w3SPI 405 DAPI.TIF]

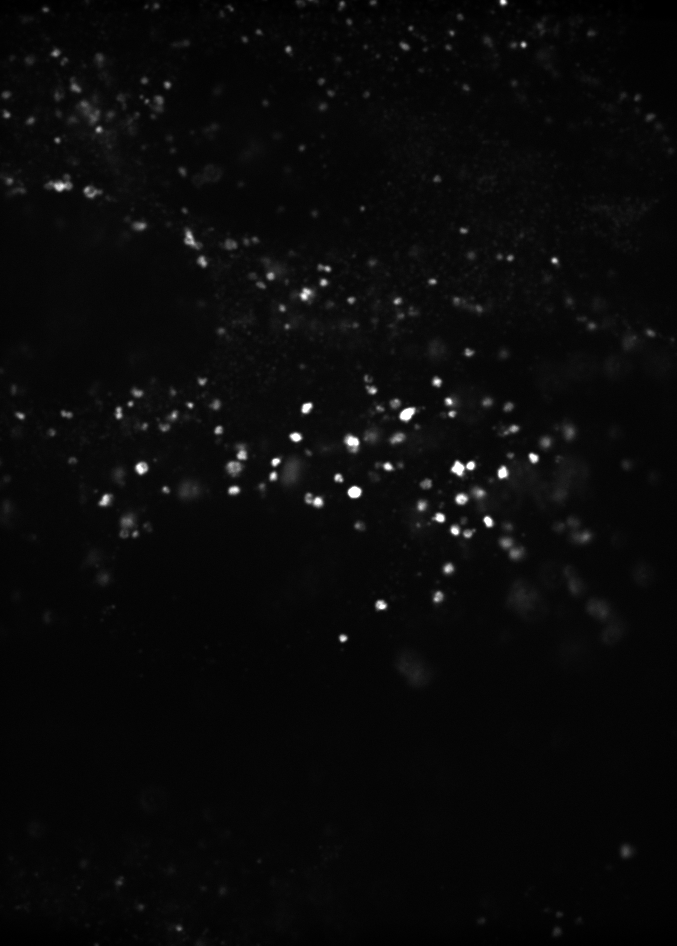

Supplement: Supplementary file 25 — Figure EV4-3 Source Data [file 44318_2026_705_MOESM25_ESM.zip › Figure EV4-3/I/MCF7_STARD3S221A/20230414_MCF7STARD3S221A_NT_2_w1SPI 491 GFP.TIF]

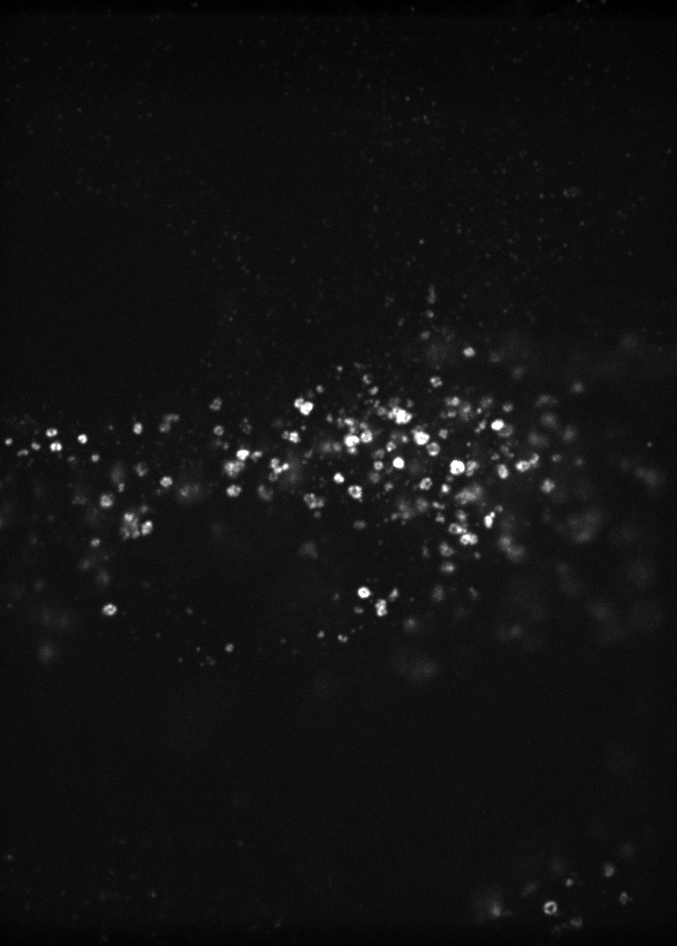

Supplement: Supplementary file 25 — Figure EV4-3 Source Data [file 44318_2026_705_MOESM25_ESM.zip › Figure EV4-3/I/MCF7_STARD3S221A/20230414_MCF7STARD3S221A_NT_2_w2SPI 561 mCherry.TIF]

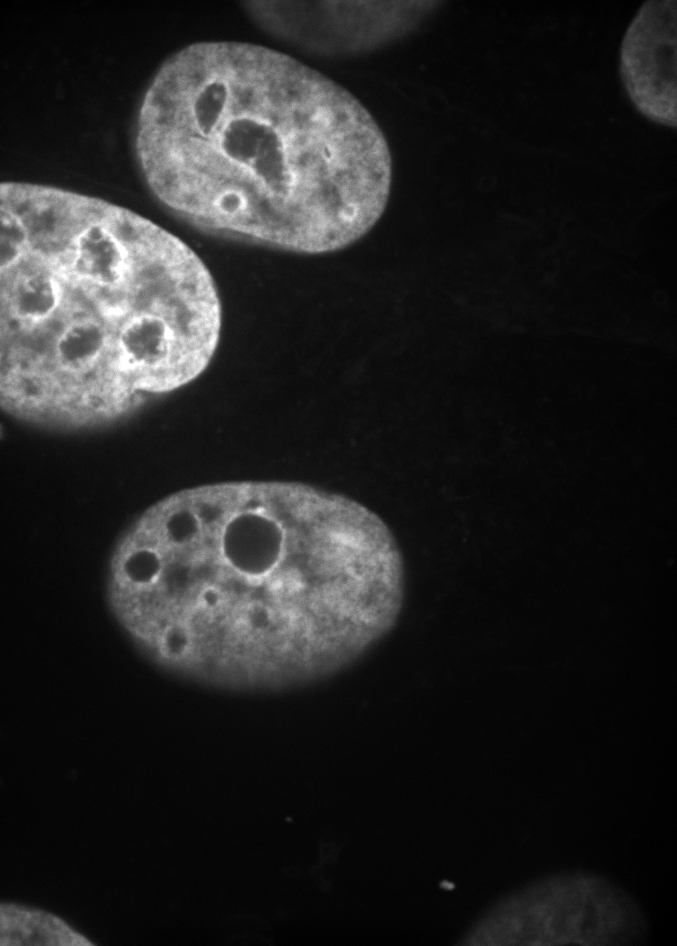

Supplement: Supplementary file 25 — Figure EV4-3 Source Data [file 44318_2026_705_MOESM25_ESM.zip › Figure EV4-3/I/MCF7_STARD3S221A/20230414_MCF7STARD3S221A_NT_2_w3SPI 405 DAPI.TIF]

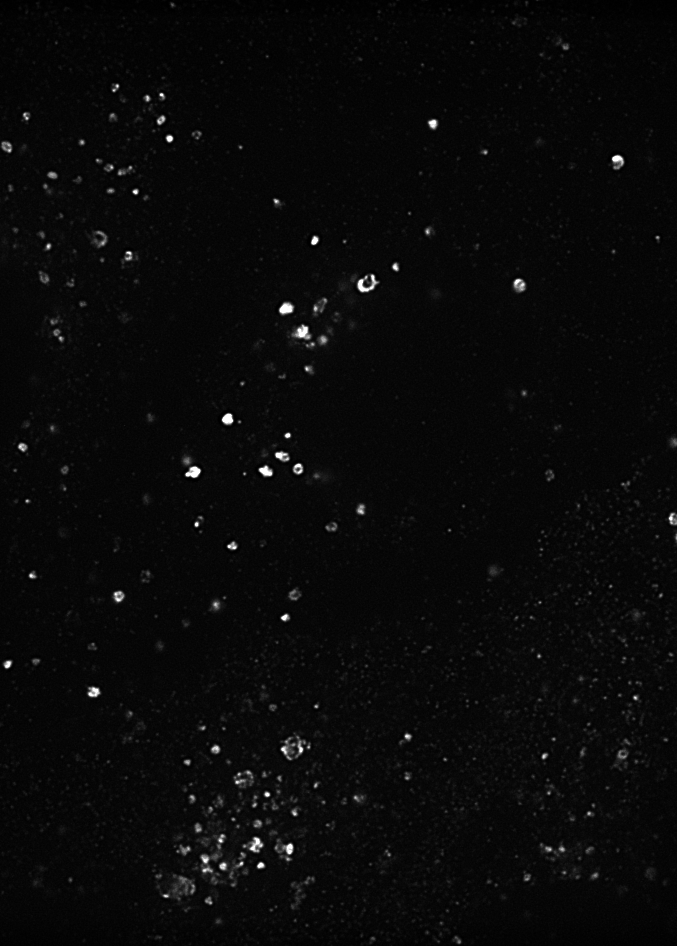

Supplement: Supplementary file 25 — Figure EV4-3 Source Data [file 44318_2026_705_MOESM25_ESM.zip › Figure EV4-3/I/MCF7_STARD3WT/20230414_MCF7STARD3WT_NT_5_SR_w1SPI 491 GFP.TIF]

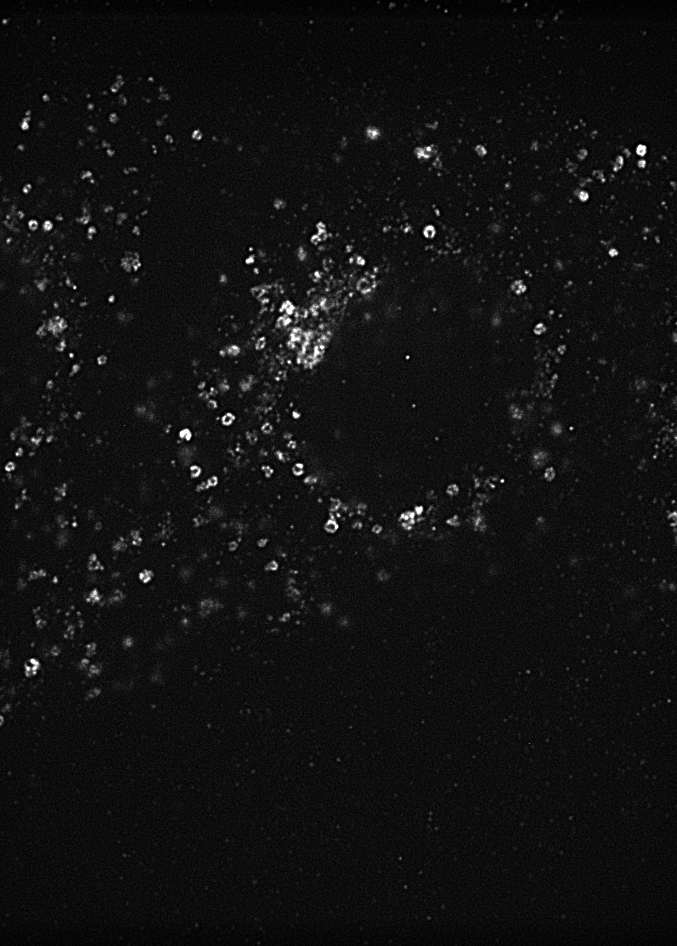

Supplement: Supplementary file 25 — Figure EV4-3 Source Data [file 44318_2026_705_MOESM25_ESM.zip › Figure EV4-3/I/MCF7_STARD3WT/20230414_MCF7STARD3WT_NT_5_SR_w2SPI 561 mCherry.TIF]

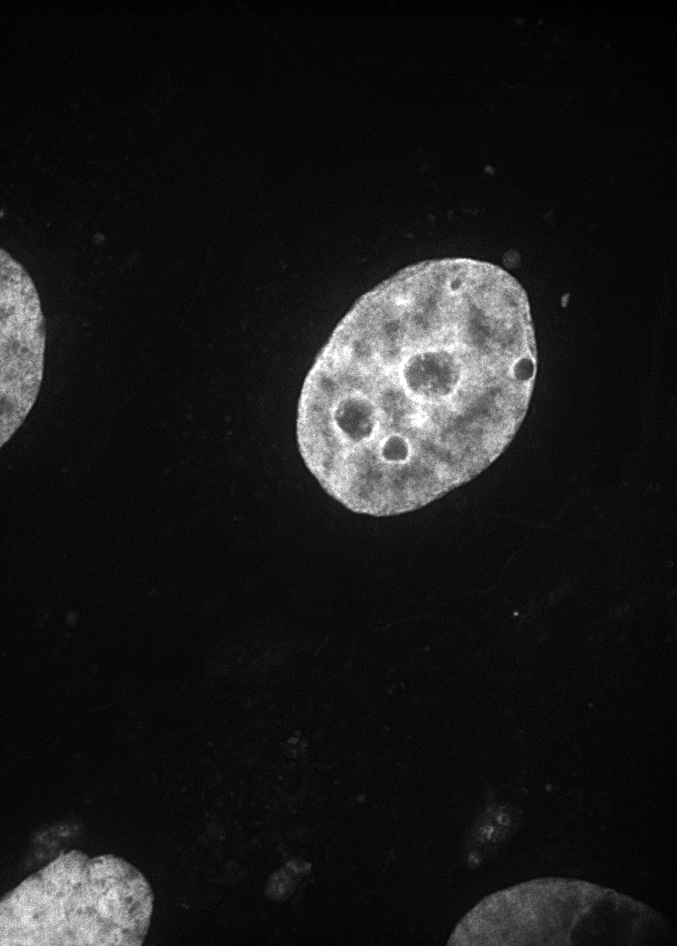

Supplement: Supplementary file 25 — Figure EV4-3 Source Data [file 44318_2026_705_MOESM25_ESM.zip › Figure EV4-3/I/MCF7_STARD3WT/20230414_MCF7STARD3WT_NT_5_SR_w3SPI 405 DAPI.TIF]

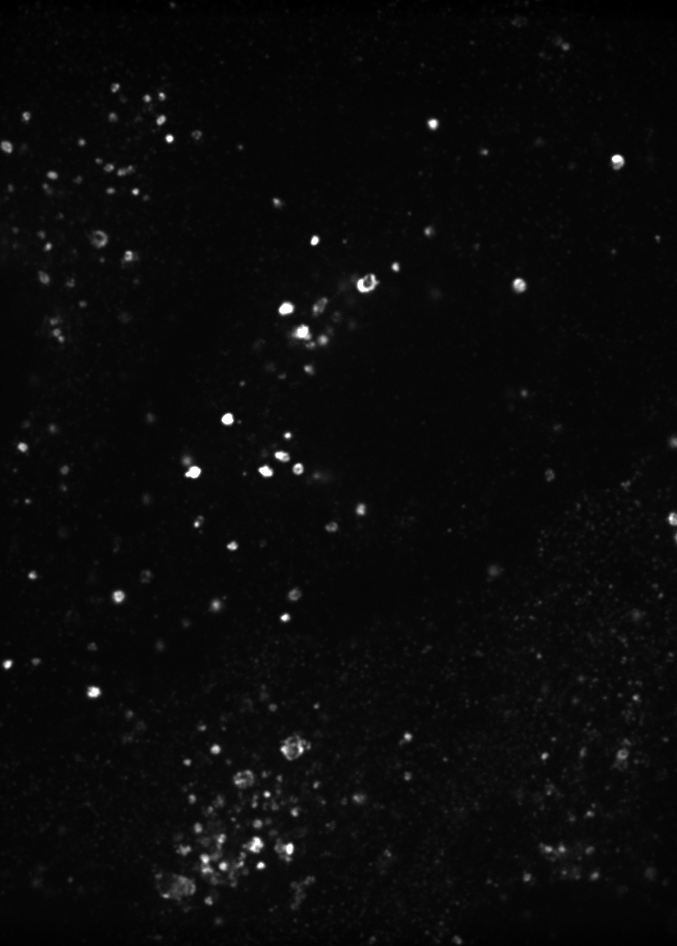

Supplement: Supplementary file 25 — Figure EV4-3 Source Data [file 44318_2026_705_MOESM25_ESM.zip › Figure EV4-3/I/MCF7_STARD3WT/20230414_MCF7STARD3WT_NT_5_w1SPI 491 GFP.TIF]

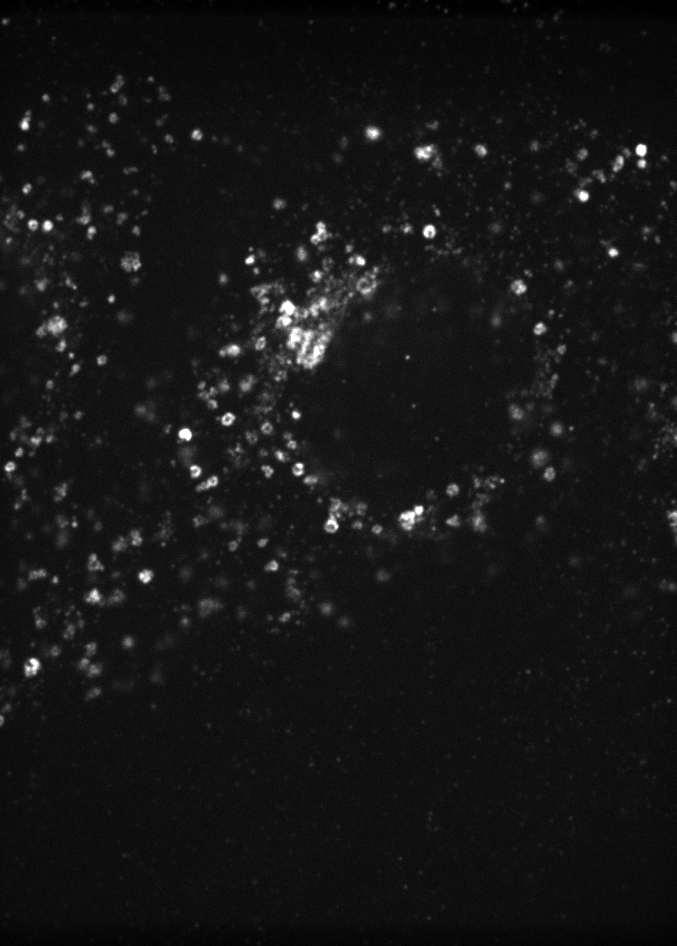

Supplement: Supplementary file 25 — Figure EV4-3 Source Data [file 44318_2026_705_MOESM25_ESM.zip › Figure EV4-3/I/MCF7_STARD3WT/20230414_MCF7STARD3WT_NT_5_w2SPI 561 mCherry.TIF]

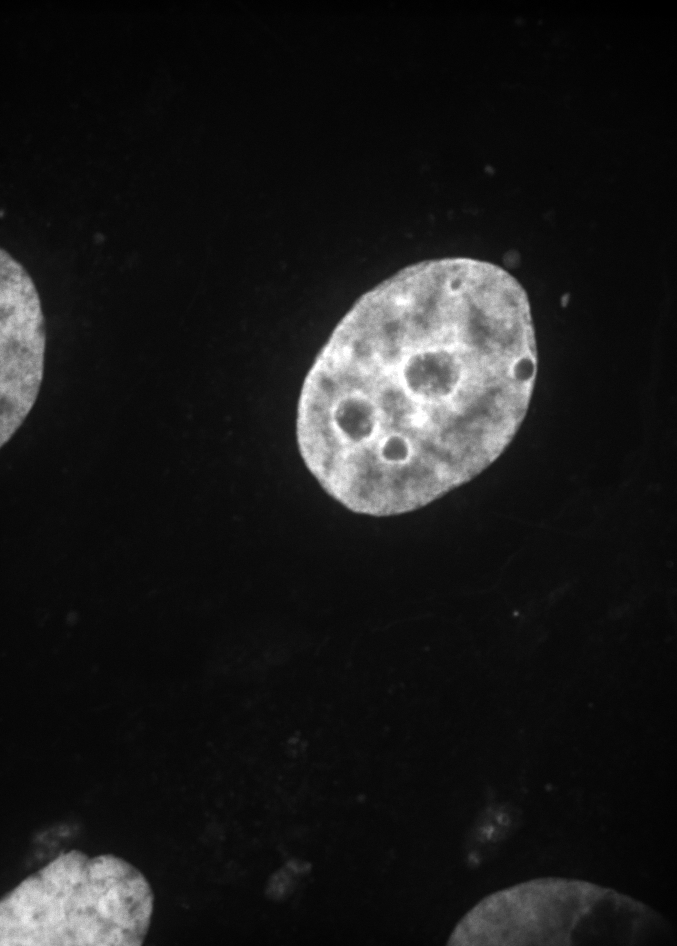

Supplement: Supplementary file 25 — Figure EV4-3 Source Data [file 44318_2026_705_MOESM25_ESM.zip › Figure EV4-3/I/MCF7_STARD3WT/20230414_MCF7STARD3WT_NT_5_w3SPI 405 DAPI.TIF]

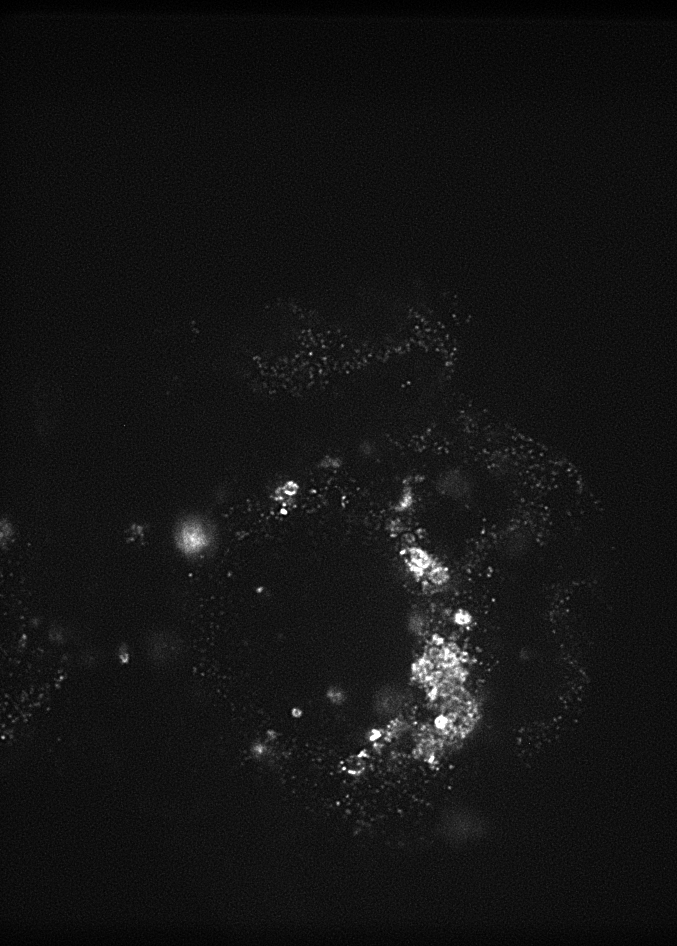

Supplement: Supplementary file 25 — Figure EV4-3 Source Data [file 44318_2026_705_MOESM25_ESM.zip › Figure EV4-3/I/MCF_STARD3S209A/20230414_MCF7STARD3S209A_NT_5_SR_w1SPI 491 GFP.TIF]

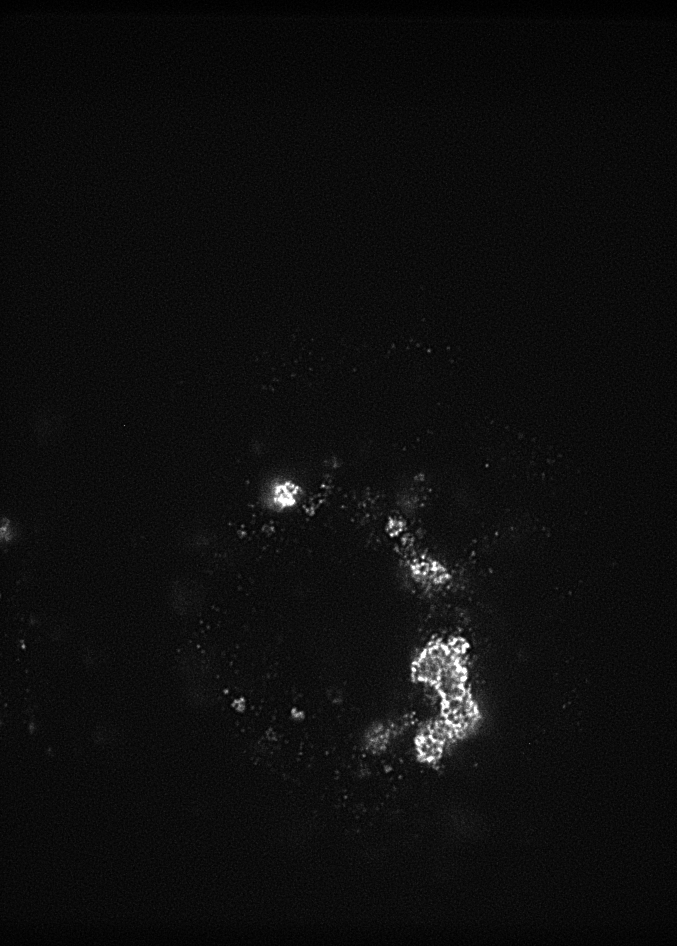

Supplement: Supplementary file 25 — Figure EV4-3 Source Data [file 44318_2026_705_MOESM25_ESM.zip › Figure EV4-3/I/MCF_STARD3S209A/20230414_MCF7STARD3S209A_NT_5_SR_w2SPI 561 mCherry.TIF]

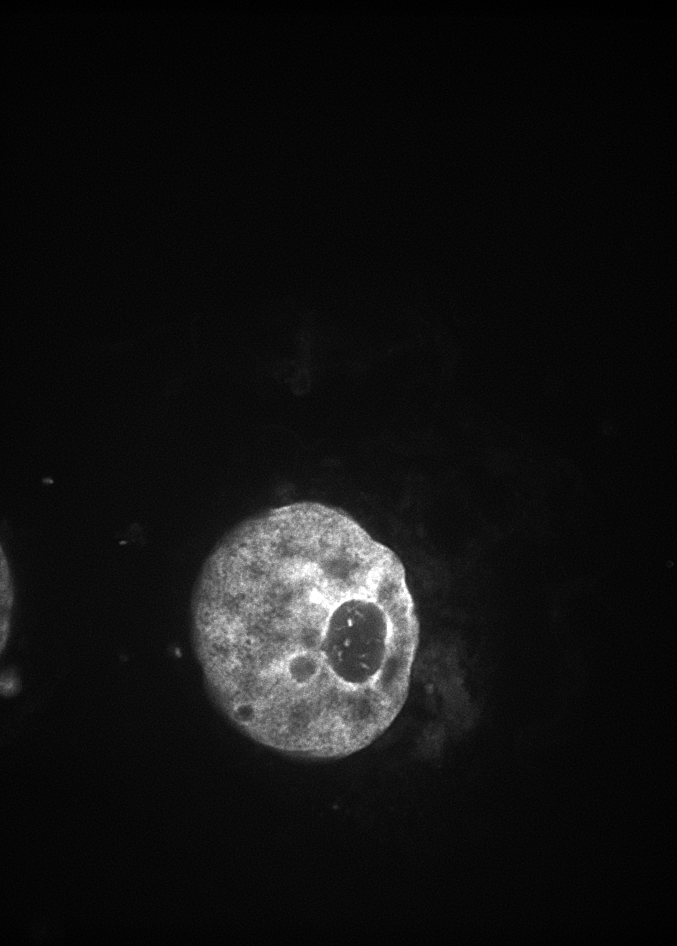

Supplement: Supplementary file 25 — Figure EV4-3 Source Data [file 44318_2026_705_MOESM25_ESM.zip › Figure EV4-3/I/MCF_STARD3S209A/20230414_MCF7STARD3S209A_NT_5_SR_w3SPI 405 DAPI.TIF]

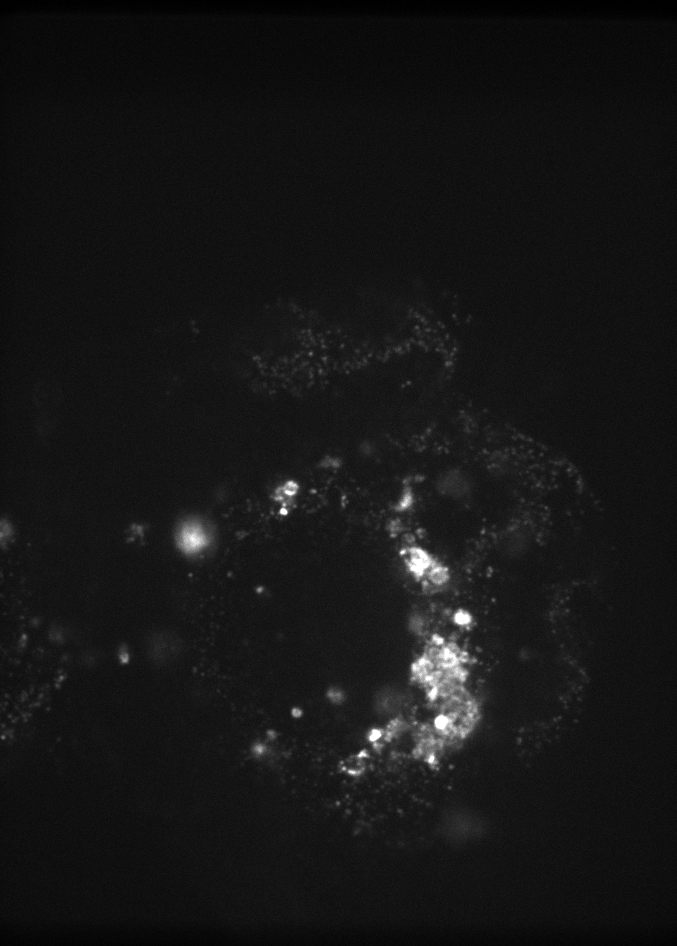

Supplement: Supplementary file 25 — Figure EV4-3 Source Data [file 44318_2026_705_MOESM25_ESM.zip › Figure EV4-3/I/MCF_STARD3S209A/20230414_MCF7STARD3S209A_NT_5_w1SPI 491 GFP.TIF]

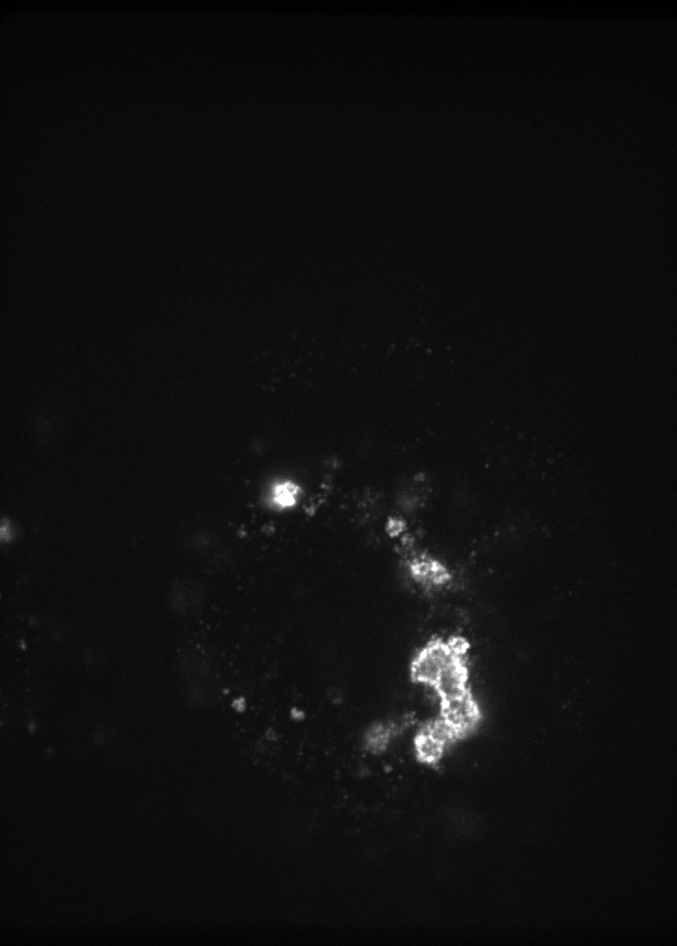

Supplement: Supplementary file 25 — Figure EV4-3 Source Data [file 44318_2026_705_MOESM25_ESM.zip › Figure EV4-3/I/MCF_STARD3S209A/20230414_MCF7STARD3S209A_NT_5_w2SPI 561 mCherry.TIF]

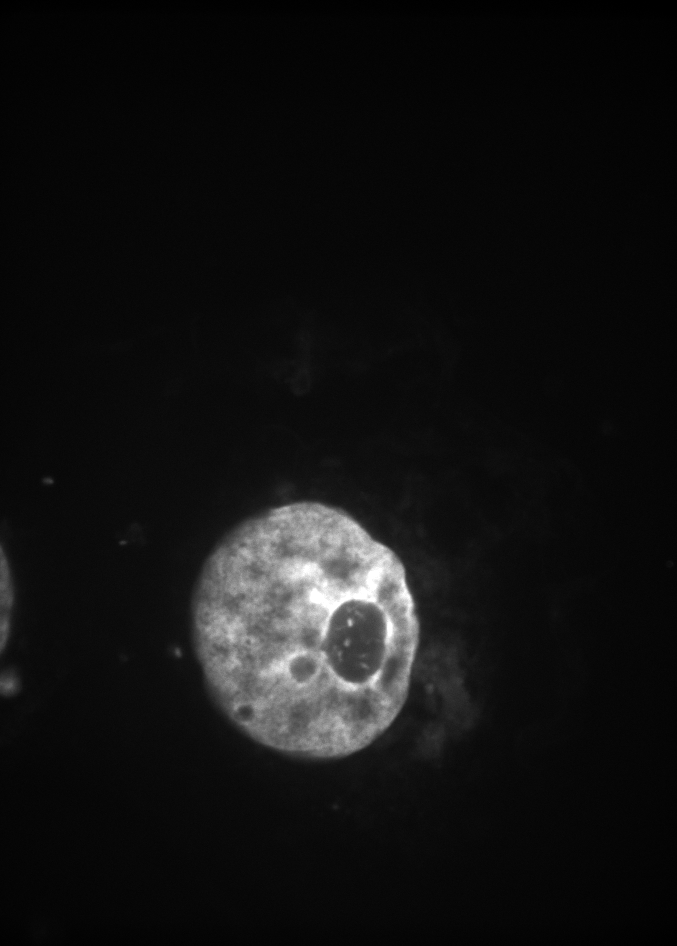

Supplement: Supplementary file 25 — Figure EV4-3 Source Data [file 44318_2026_705_MOESM25_ESM.zip › Figure EV4-3/I/MCF_STARD3S209A/20230414_MCF7STARD3S209A_NT_5_w3SPI 405 DAPI.TIF]

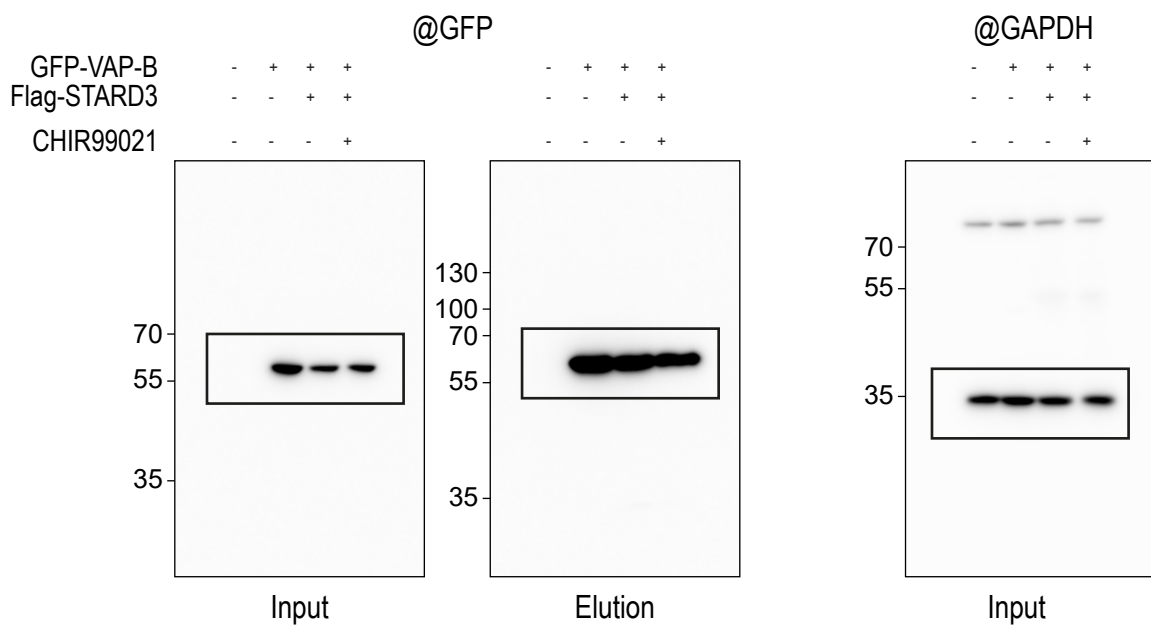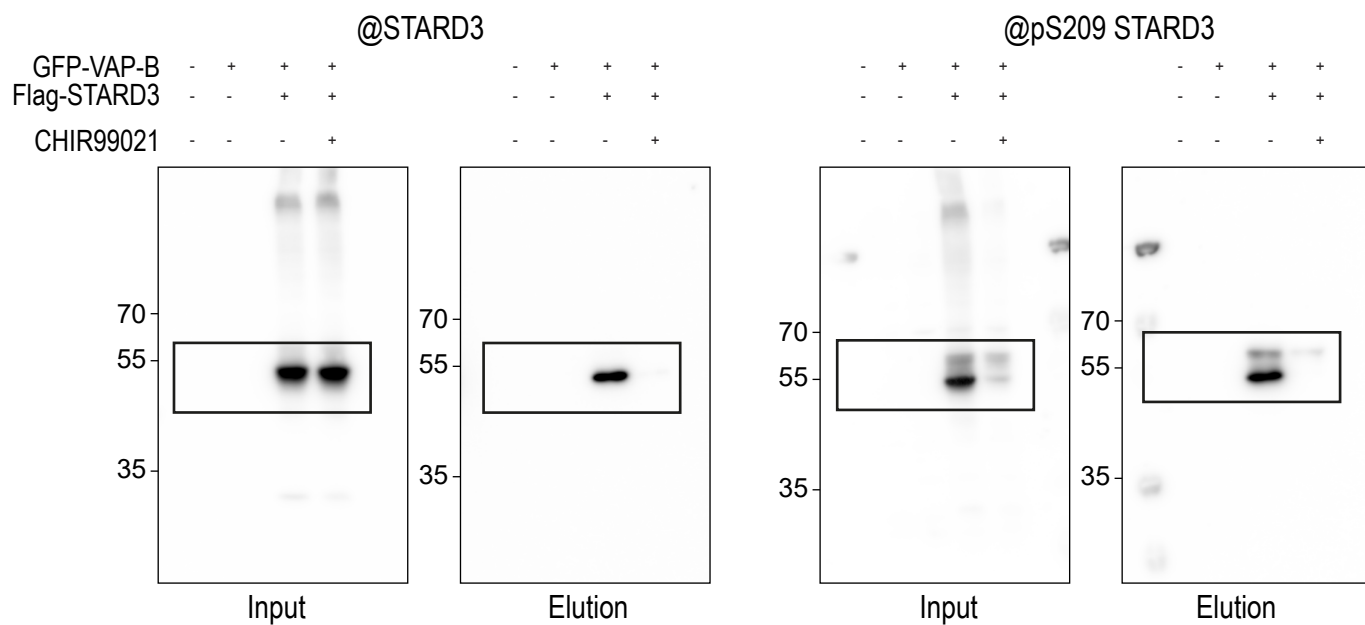

Supplement: Supplementary file 27 — Appendix Fig. S2-1 Source Data [file 44318_2026_705_MOESM27_ESM.zip › Appendix Figure S2-1/B/GFP Trap-VAPB_WB.pdf]

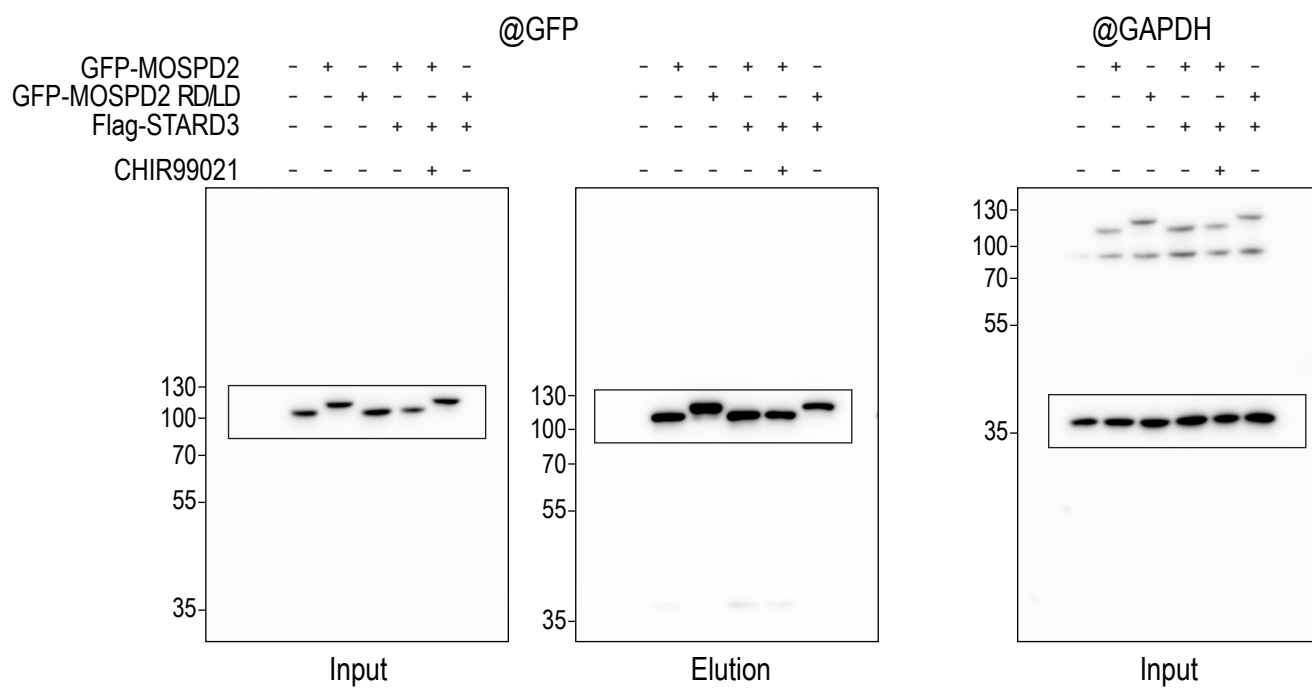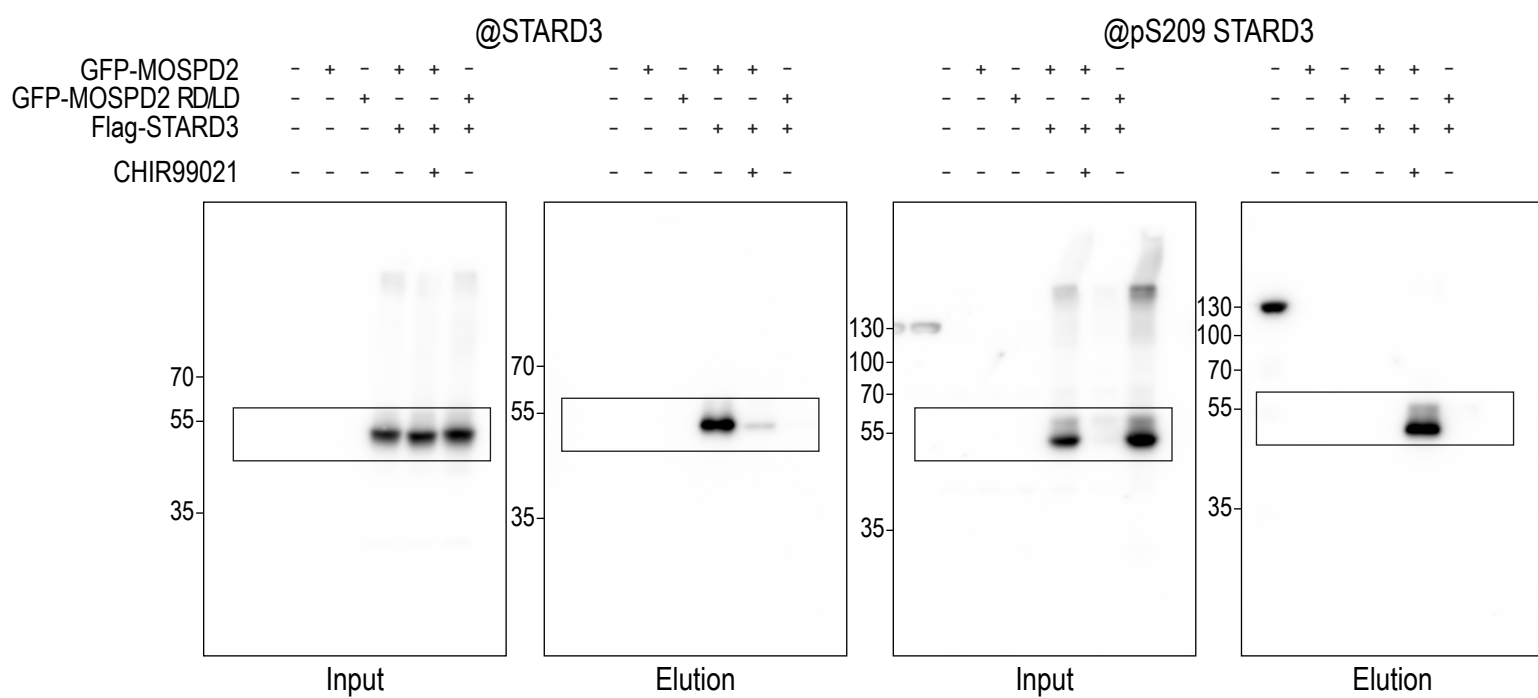

Supplement: Supplementary file 27 — Appendix Fig. S2-1 Source Data [file 44318_2026_705_MOESM27_ESM.zip › Appendix Figure S2-1/C/GFP Trap-MOSPD2_WB.pdf]

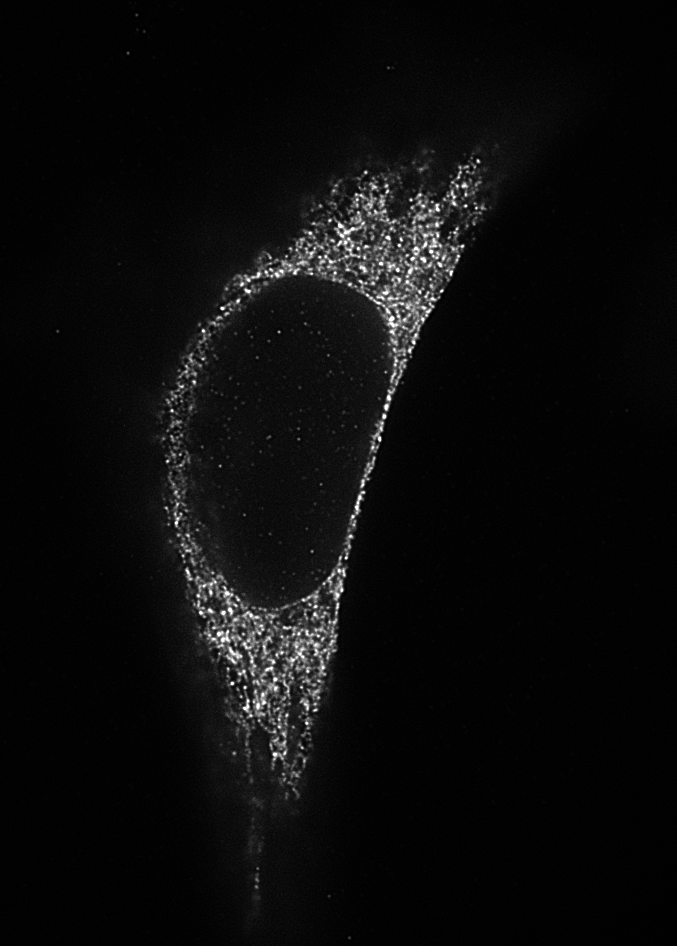

Supplement: Supplementary file 27 — Appendix Fig. S2-1 Source Data [file 44318_2026_705_MOESM27_ESM.zip › Appendix Figure S2-1/D/20240506_HeLaGFPVAPA_NT_3_SR_w1SPI 491 GFP.TIF]

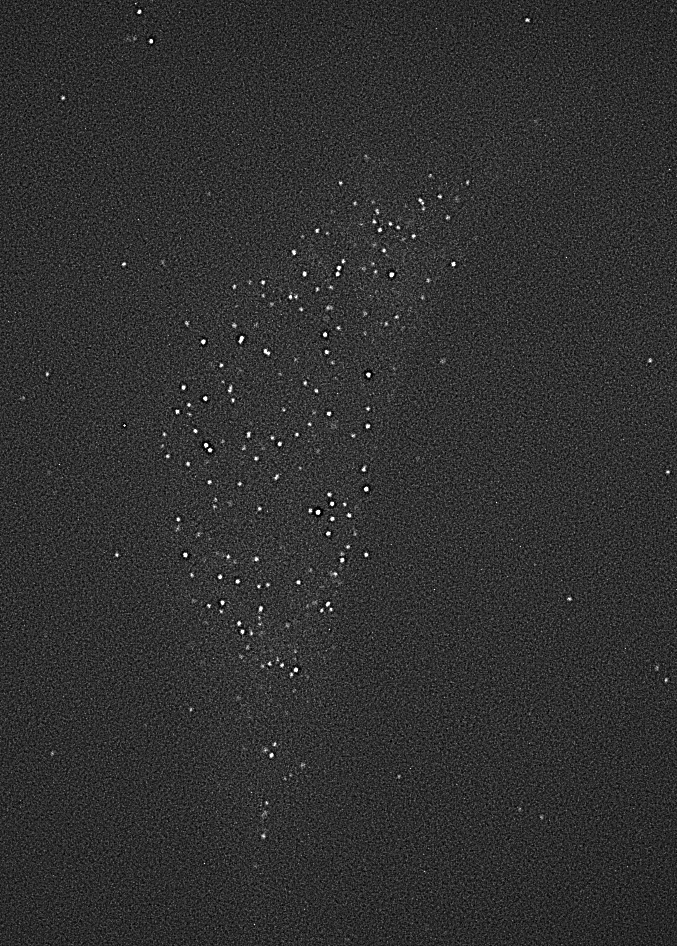

Supplement: Supplementary file 27 — Appendix Fig. S2-1 Source Data [file 44318_2026_705_MOESM27_ESM.zip › Appendix Figure S2-1/D/20240506_HeLaGFPVAPA_NT_3_SR_w2SPI 561 mCherry.TIF]

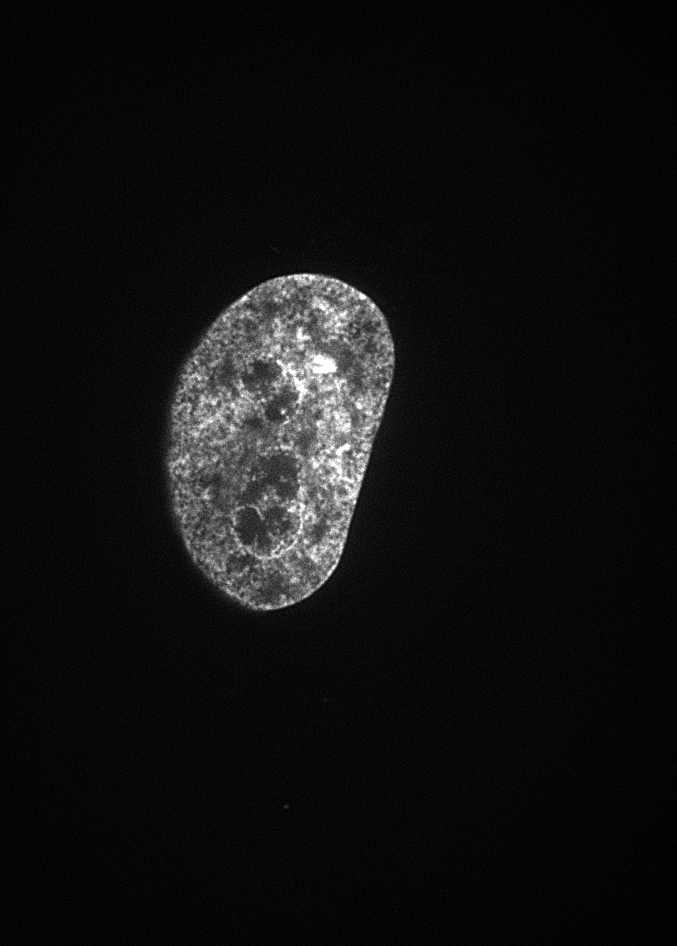

Supplement: Supplementary file 27 — Appendix Fig. S2-1 Source Data [file 44318_2026_705_MOESM27_ESM.zip › Appendix Figure S2-1/D/20240506_HeLaGFPVAPA_NT_3_SR_w3SPI 405 DAPI.TIF]

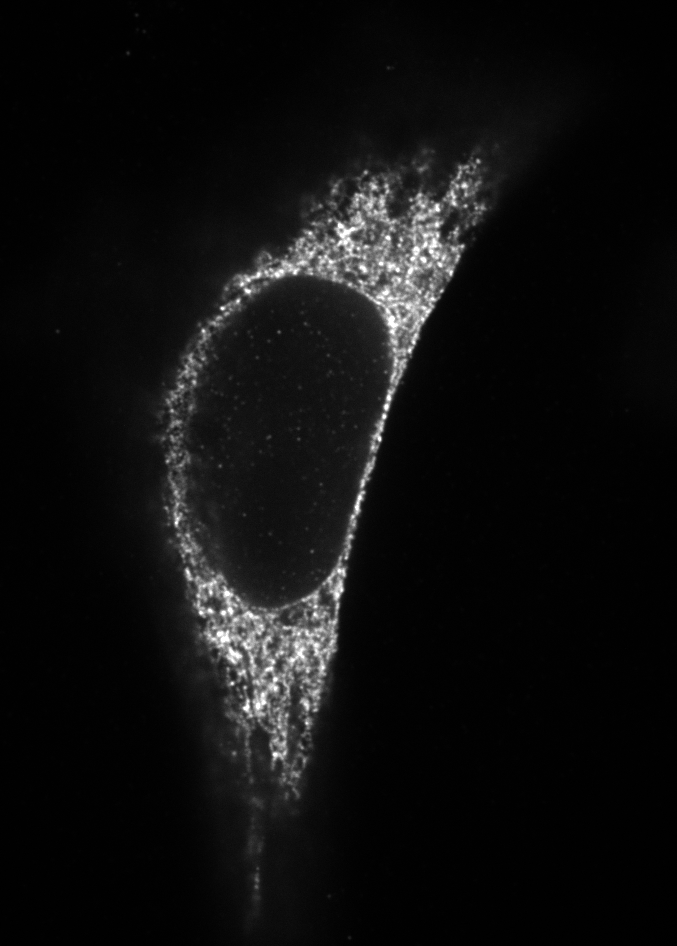

Supplement: Supplementary file 27 — Appendix Fig. S2-1 Source Data [file 44318_2026_705_MOESM27_ESM.zip › Appendix Figure S2-1/D/20240506_HeLaGFPVAPA_NT_3_w1SPI 491 GFP.TIF]

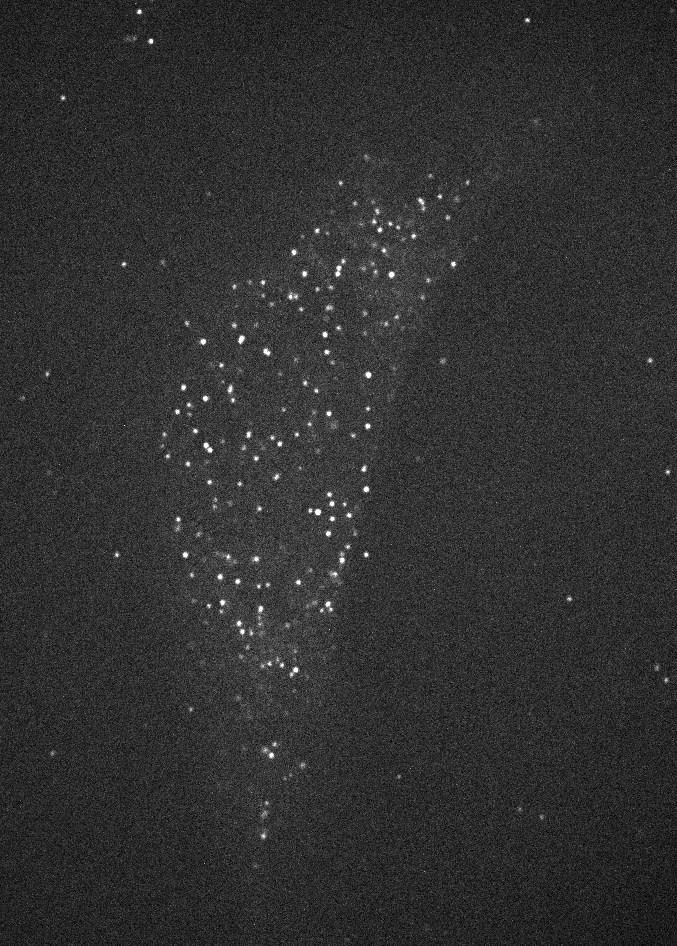

Supplement: Supplementary file 27 — Appendix Fig. S2-1 Source Data [file 44318_2026_705_MOESM27_ESM.zip › Appendix Figure S2-1/D/20240506_HeLaGFPVAPA_NT_3_w2SPI 561 mCherry.TIF]

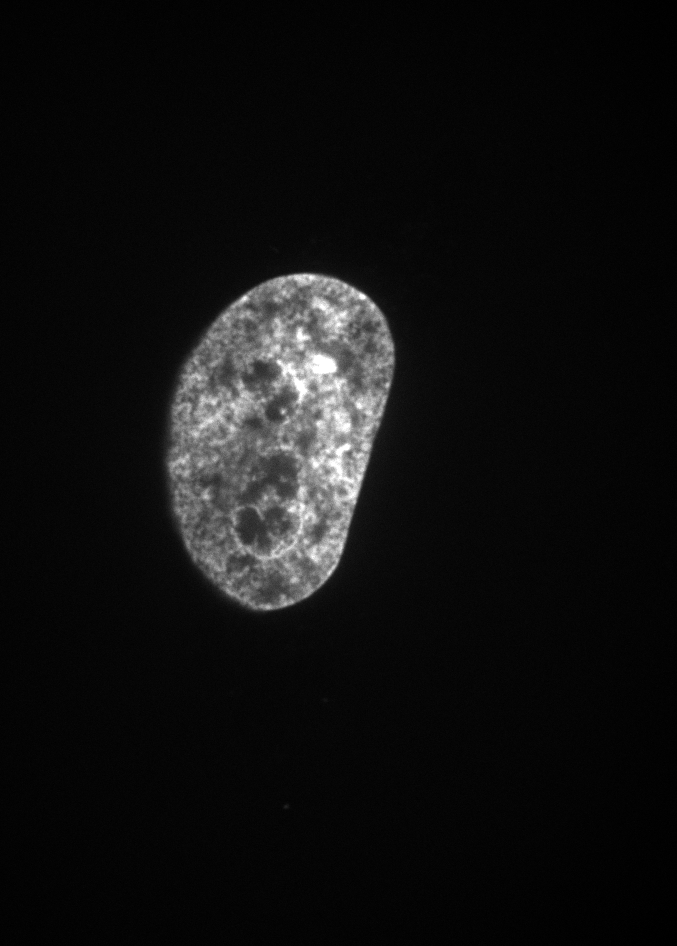

Supplement: Supplementary file 27 — Appendix Fig. S2-1 Source Data [file 44318_2026_705_MOESM27_ESM.zip › Appendix Figure S2-1/D/20240506_HeLaGFPVAPA_NT_3_w3SPI 405 DAPI.TIF]

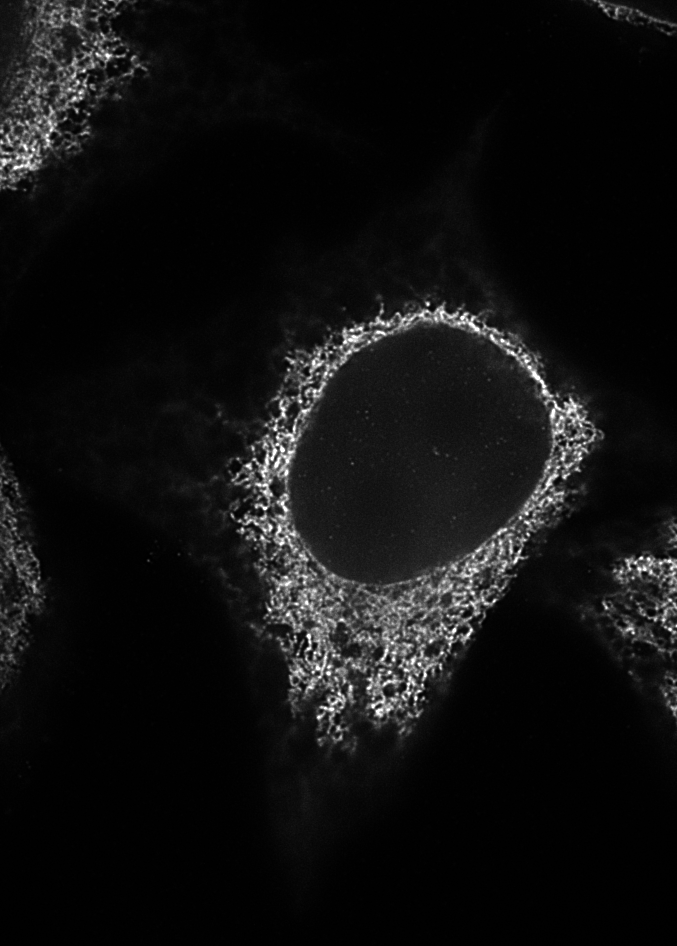

Supplement: Supplementary file 27 — Appendix Fig. S2-1 Source Data [file 44318_2026_705_MOESM27_ESM.zip › Appendix Figure S2-1/E/20240506_HeLaGFPVAPA_CHIR_3_SR_w1SPI 491 GFP.TIF]

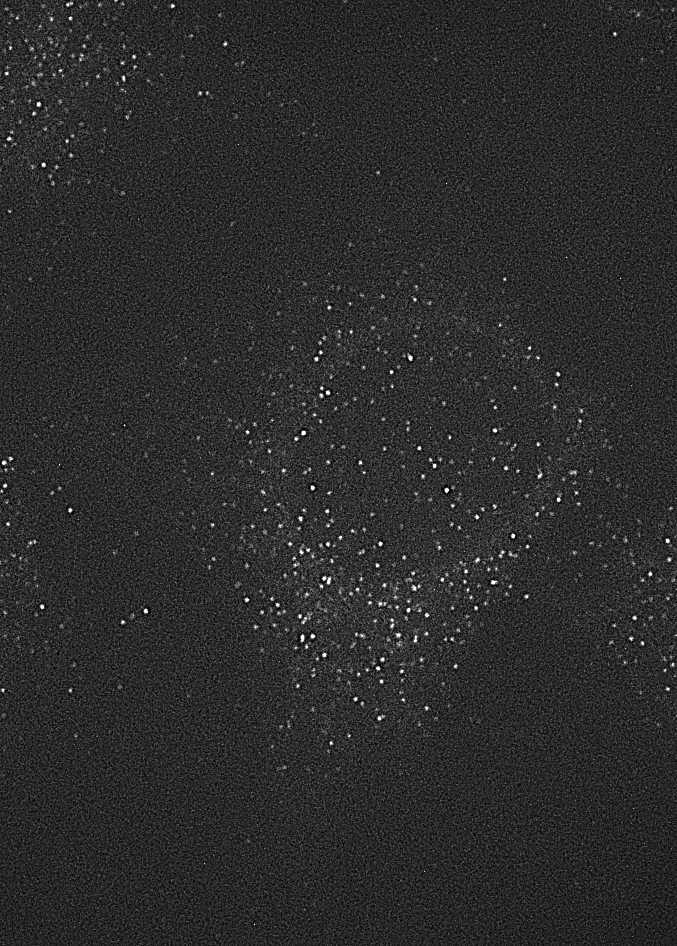

Supplement: Supplementary file 27 — Appendix Fig. S2-1 Source Data [file 44318_2026_705_MOESM27_ESM.zip › Appendix Figure S2-1/E/20240506_HeLaGFPVAPA_CHIR_3_SR_w2SPI 561 mCherry.TIF]

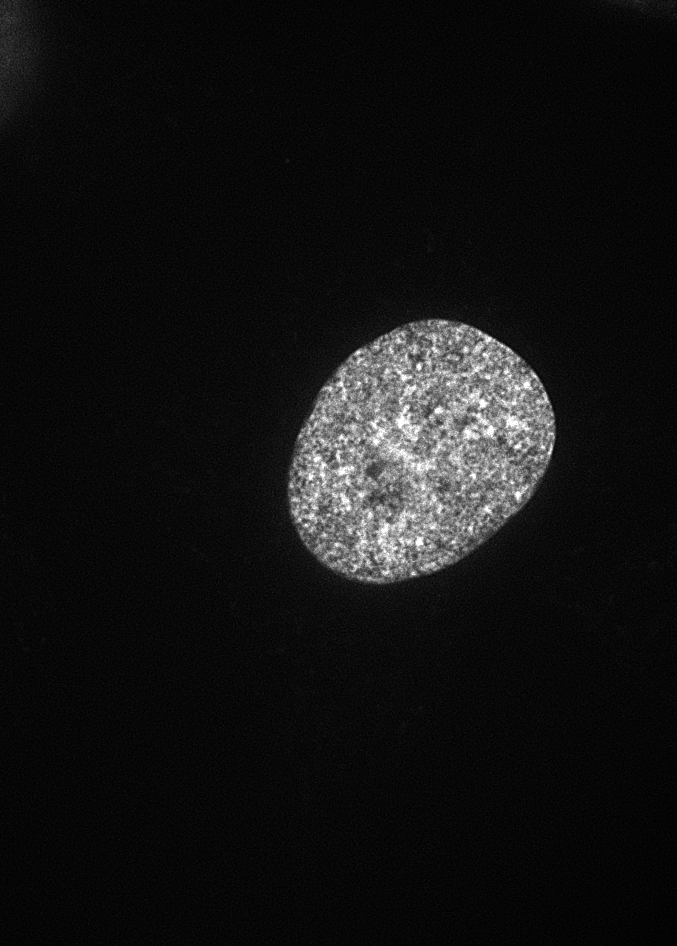

Supplement: Supplementary file 27 — Appendix Fig. S2-1 Source Data [file 44318_2026_705_MOESM27_ESM.zip › Appendix Figure S2-1/E/20240506_HeLaGFPVAPA_CHIR_3_SR_w3SPI 405 DAPI.TIF]

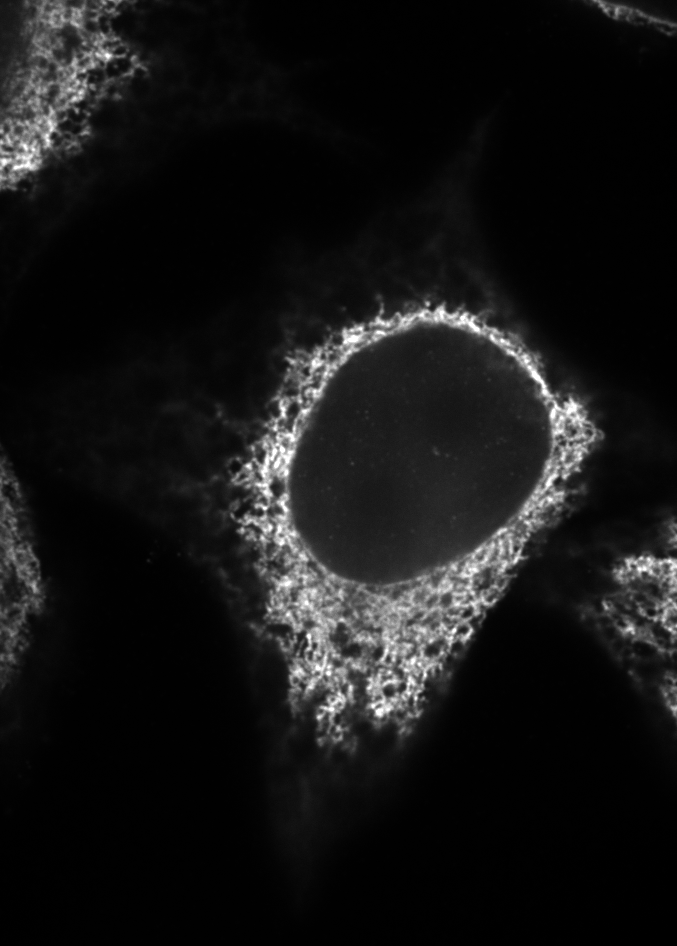

Supplement: Supplementary file 27 — Appendix Fig. S2-1 Source Data [file 44318_2026_705_MOESM27_ESM.zip › Appendix Figure S2-1/E/20240506_HeLaGFPVAPA_CHIR_3_w1SPI 491 GFP.TIF]

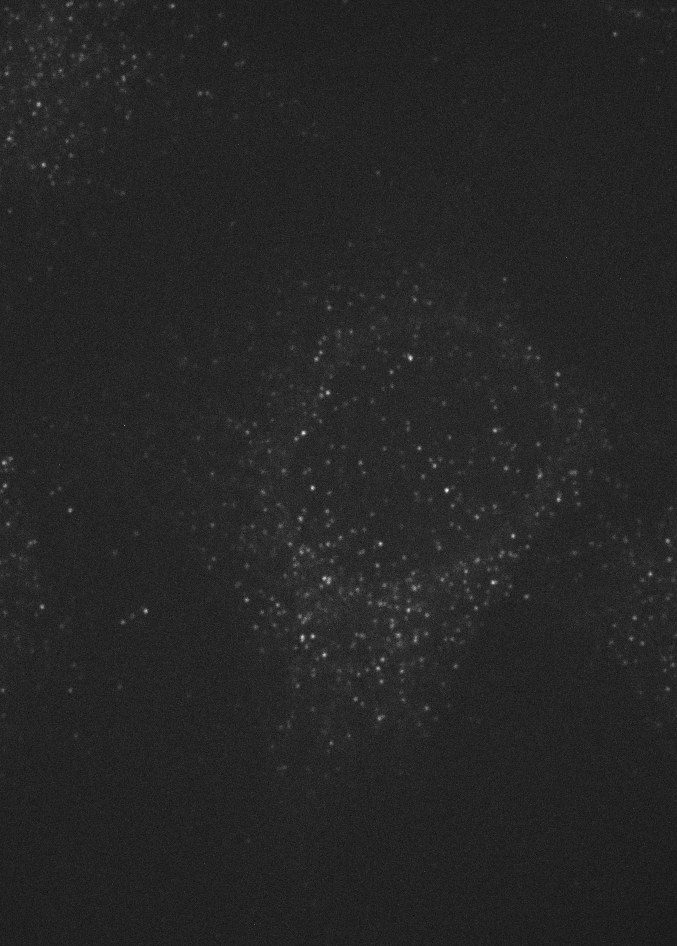

Supplement: Supplementary file 27 — Appendix Fig. S2-1 Source Data [file 44318_2026_705_MOESM27_ESM.zip › Appendix Figure S2-1/E/20240506_HeLaGFPVAPA_CHIR_3_w2SPI 561 mCherry.TIF]

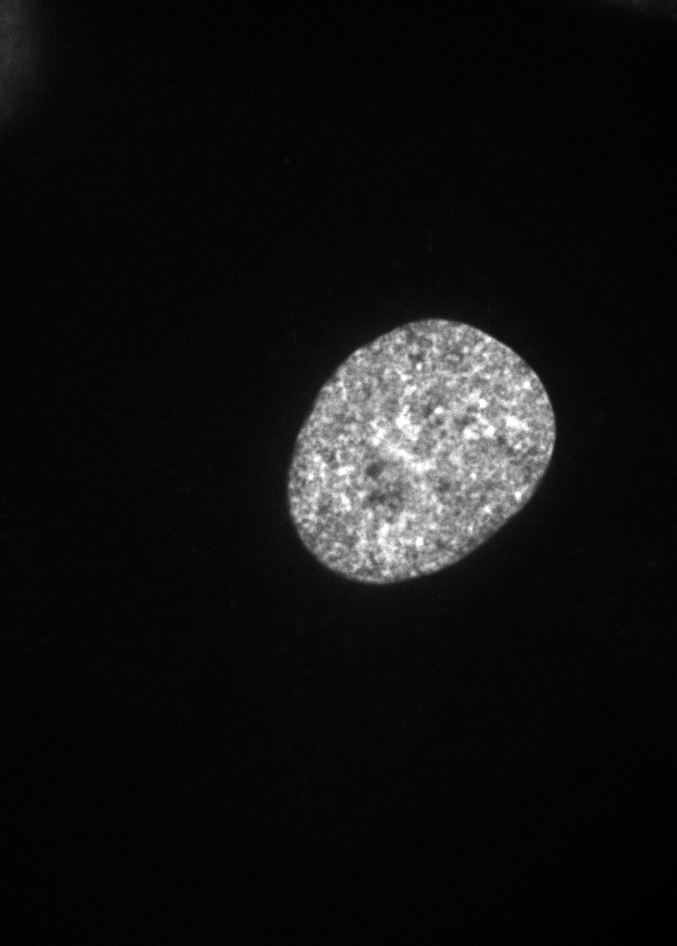

Supplement: Supplementary file 27 — Appendix Fig. S2-1 Source Data [file 44318_2026_705_MOESM27_ESM.zip › Appendix Figure S2-1/E/20240506_HeLaGFPVAPA_CHIR_3_w3SPI 405 DAPI.TIF]

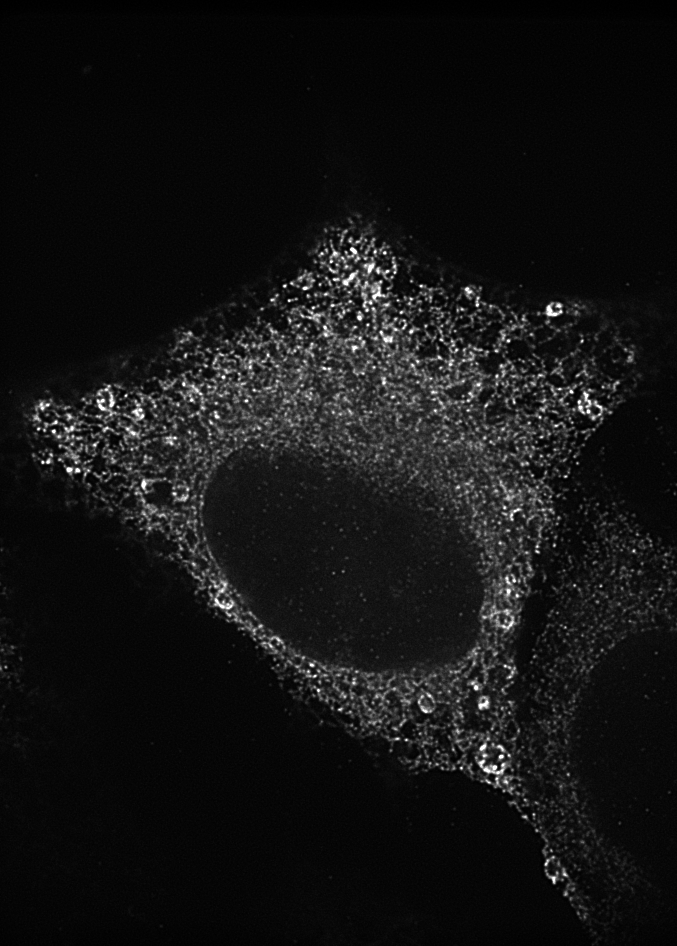

Supplement: Supplementary file 27 — Appendix Fig. S2-1 Source Data [file 44318_2026_705_MOESM27_ESM.zip › Appendix Figure S2-1/F/20240112_HeLaGFPVAPAantiGFP_STARD3WT_NT_9_SR_w1SPI 491 GFP.TIF]

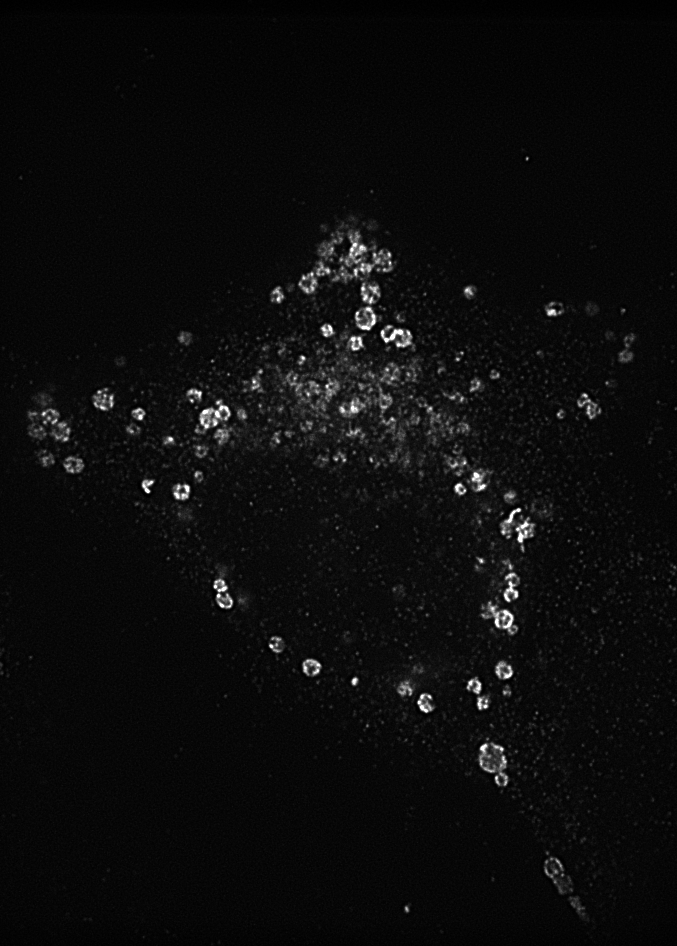

Supplement: Supplementary file 27 — Appendix Fig. S2-1 Source Data [file 44318_2026_705_MOESM27_ESM.zip › Appendix Figure S2-1/F/20240112_HeLaGFPVAPAantiGFP_STARD3WT_NT_9_SR_w2SPI 561 mCherry.TIF]

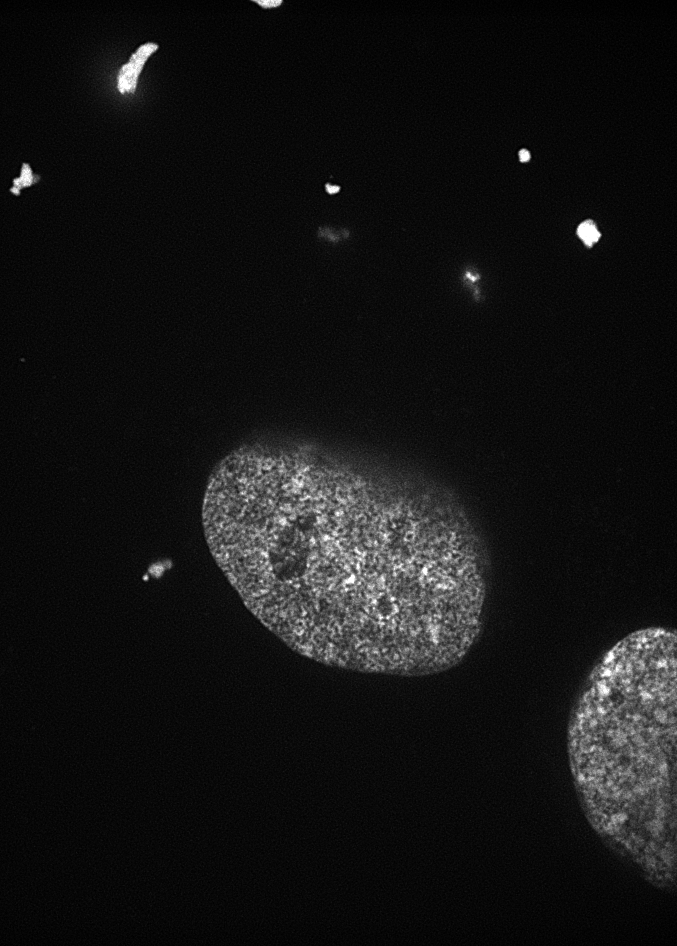

Supplement: Supplementary file 27 — Appendix Fig. S2-1 Source Data [file 44318_2026_705_MOESM27_ESM.zip › Appendix Figure S2-1/F/20240112_HeLaGFPVAPAantiGFP_STARD3WT_NT_9_SR_w3SPI 405 DAPI.TIF]

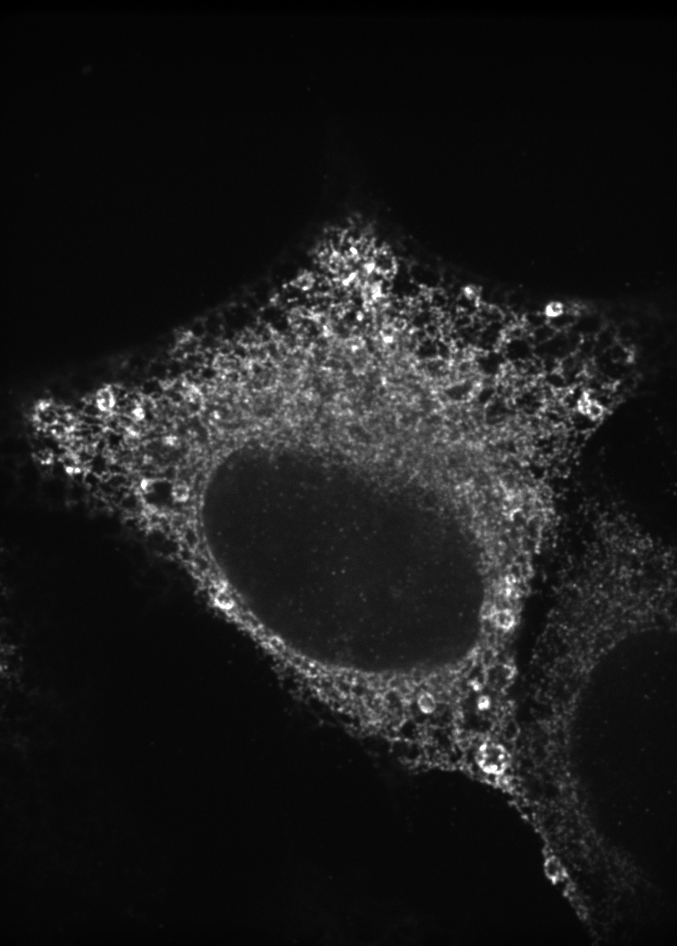

Supplement: Supplementary file 27 — Appendix Fig. S2-1 Source Data [file 44318_2026_705_MOESM27_ESM.zip › Appendix Figure S2-1/F/20240112_HeLaGFPVAPAantiGFP_STARD3WT_NT_9_w1SPI 491 GFP.TIF]

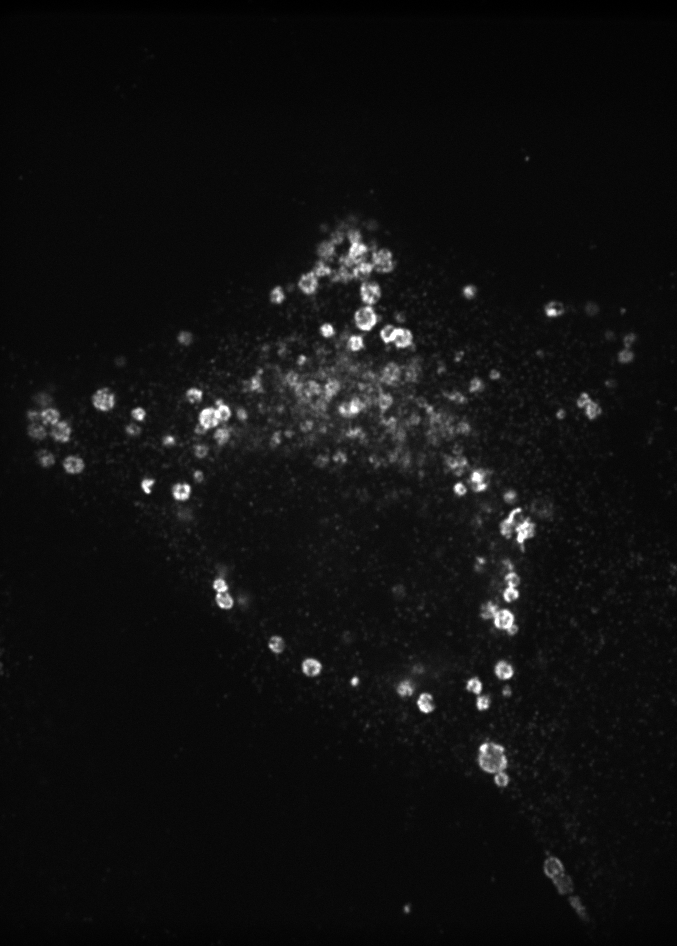

Supplement: Supplementary file 27 — Appendix Fig. S2-1 Source Data [file 44318_2026_705_MOESM27_ESM.zip › Appendix Figure S2-1/F/20240112_HeLaGFPVAPAantiGFP_STARD3WT_NT_9_w2SPI 561 mCherry.TIF]

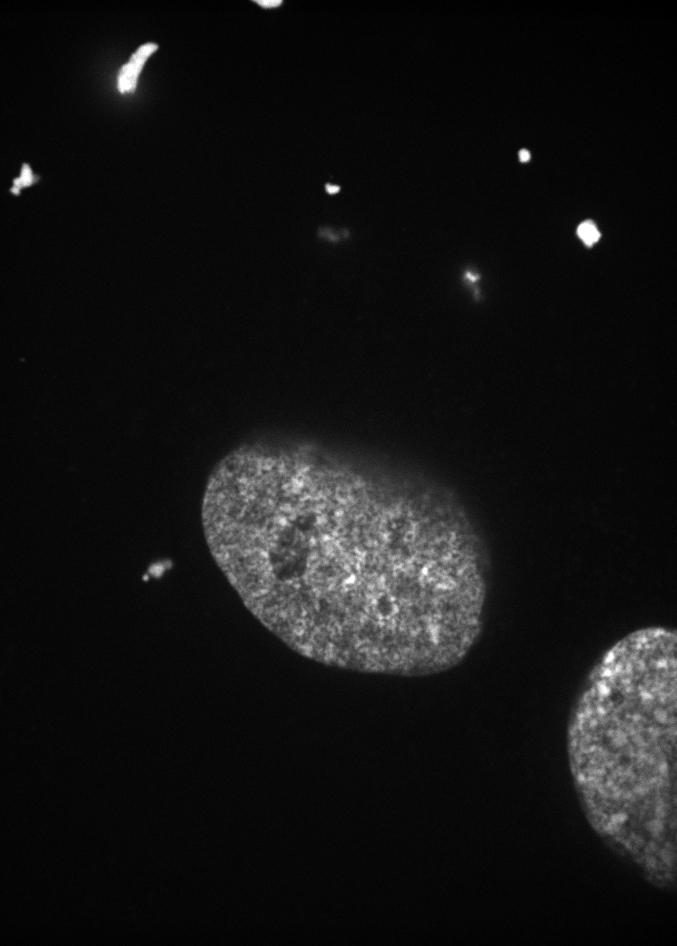

Supplement: Supplementary file 27 — Appendix Fig. S2-1 Source Data [file 44318_2026_705_MOESM27_ESM.zip › Appendix Figure S2-1/F/20240112_HeLaGFPVAPAantiGFP_STARD3WT_NT_9_w3SPI 405 DAPI.TIF]

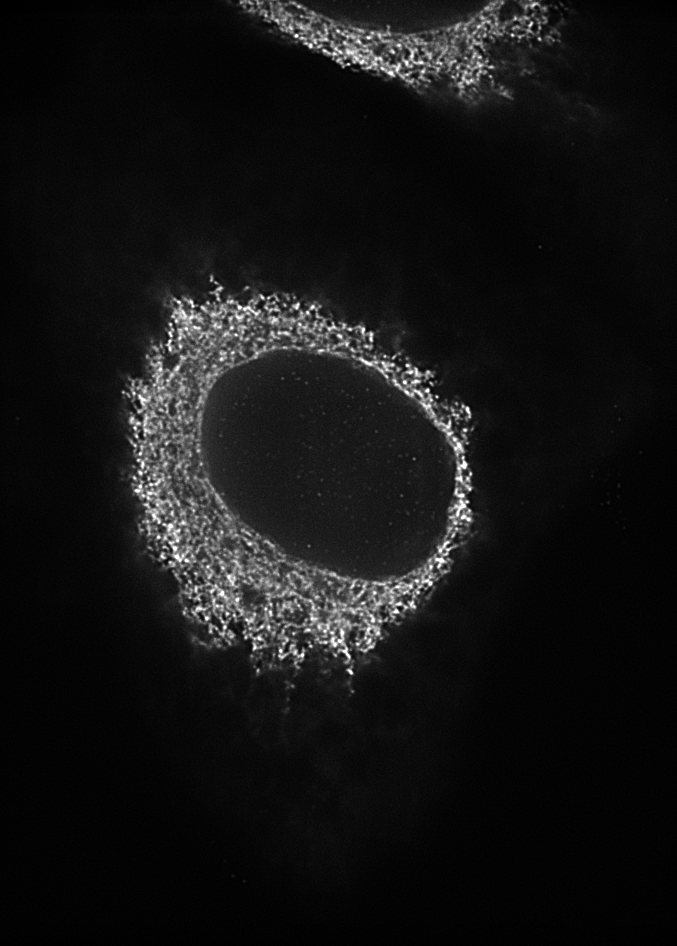

Supplement: Supplementary file 27 — Appendix Fig. S2-1 Source Data [file 44318_2026_705_MOESM27_ESM.zip › Appendix Figure S2-1/G/20240112_HeLaGFPVAPAantiGFP_STARD3S209Avrai_NT_2_SR_w1SPI 491 GFP.TIF]

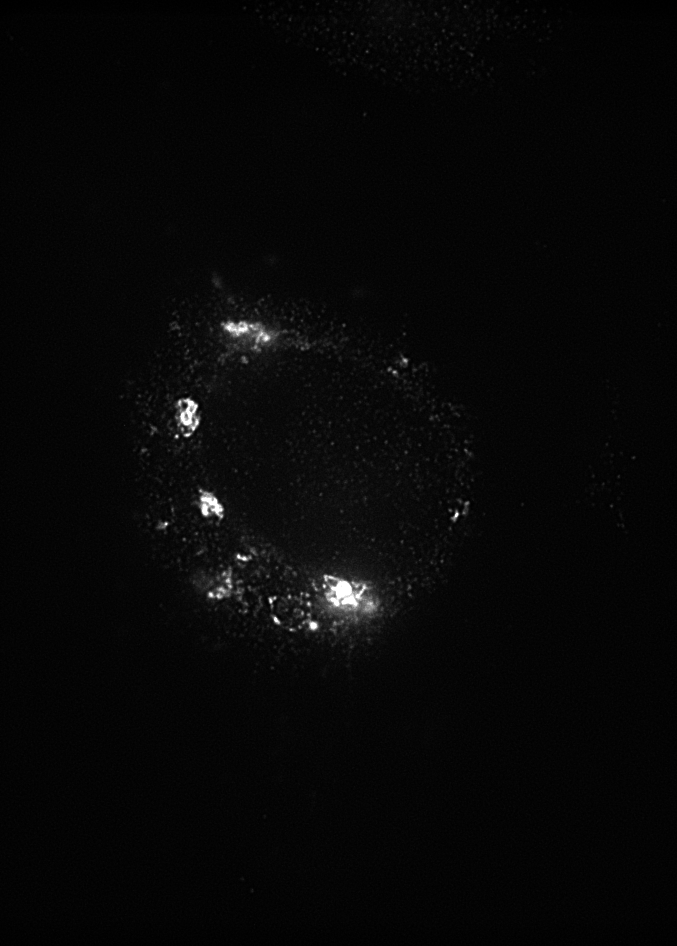

Supplement: Supplementary file 27 — Appendix Fig. S2-1 Source Data [file 44318_2026_705_MOESM27_ESM.zip › Appendix Figure S2-1/G/20240112_HeLaGFPVAPAantiGFP_STARD3S209Avrai_NT_2_SR_w2SPI 561 mCherry.TIF]

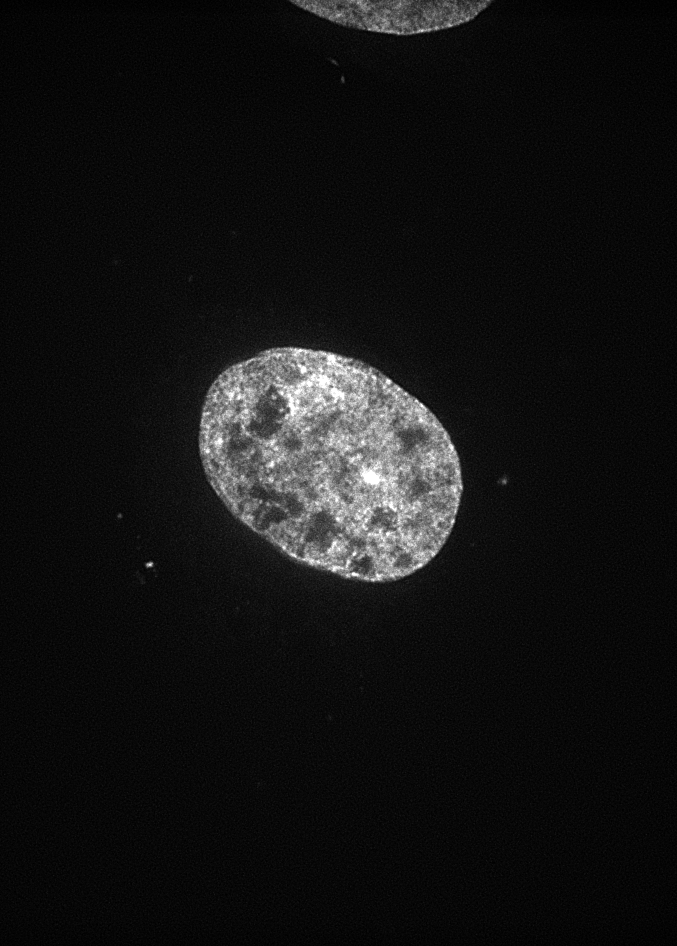

Supplement: Supplementary file 27 — Appendix Fig. S2-1 Source Data [file 44318_2026_705_MOESM27_ESM.zip › Appendix Figure S2-1/G/20240112_HeLaGFPVAPAantiGFP_STARD3S209Avrai_NT_2_SR_w3SPI 405 DAPI.TIF]

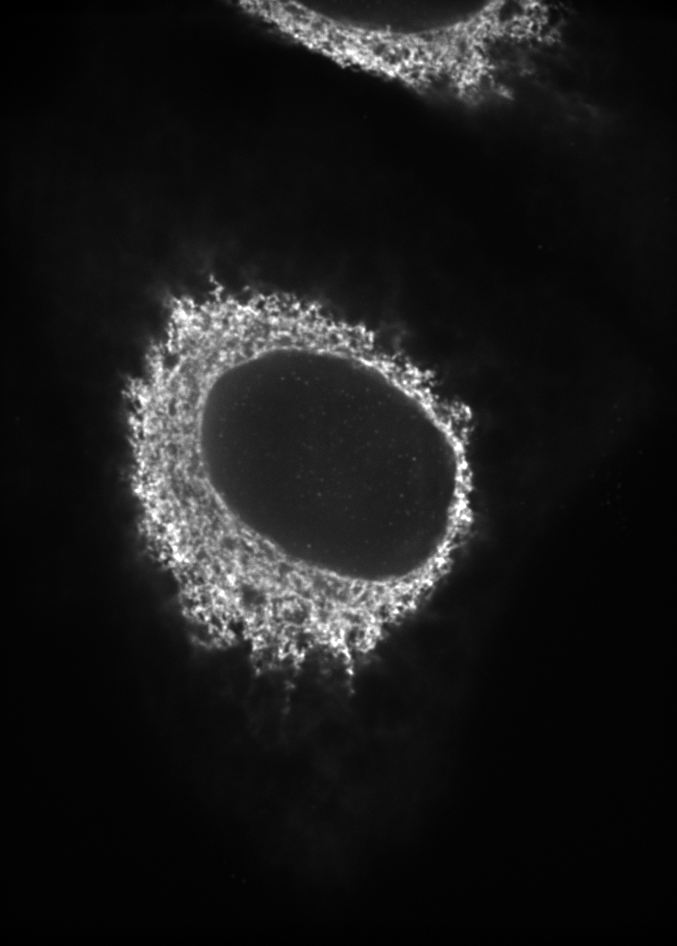

Supplement: Supplementary file 27 — Appendix Fig. S2-1 Source Data [file 44318_2026_705_MOESM27_ESM.zip › Appendix Figure S2-1/G/20240112_HeLaGFPVAPAantiGFP_STARD3S209Avrai_NT_2_w1SPI 491 GFP.TIF]

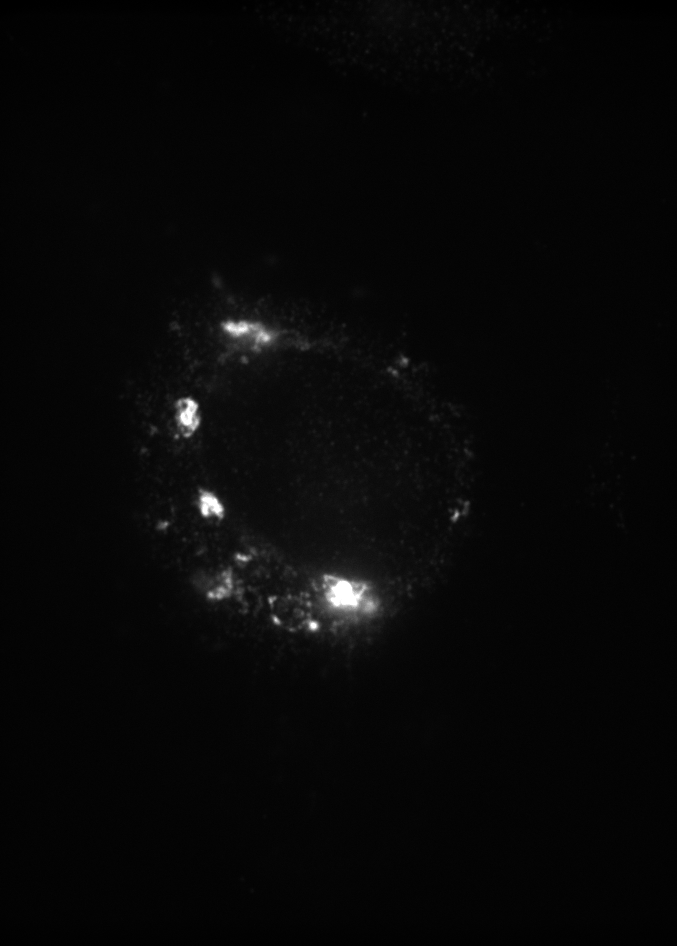

Supplement: Supplementary file 27 — Appendix Fig. S2-1 Source Data [file 44318_2026_705_MOESM27_ESM.zip › Appendix Figure S2-1/G/20240112_HeLaGFPVAPAantiGFP_STARD3S209Avrai_NT_2_w2SPI 561 mCherry.TIF]

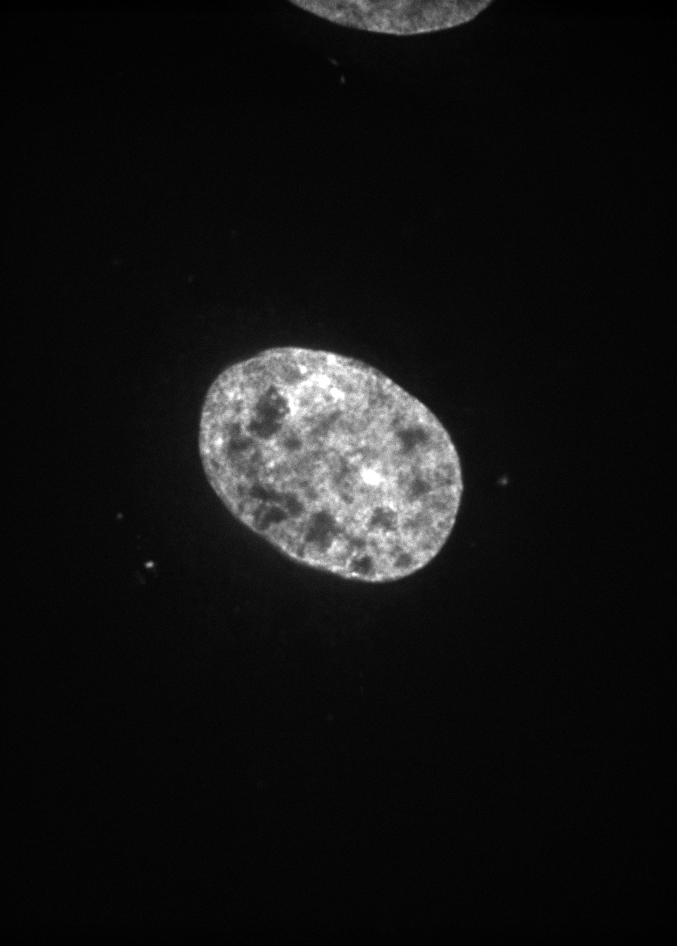

Supplement: Supplementary file 27 — Appendix Fig. S2-1 Source Data [file 44318_2026_705_MOESM27_ESM.zip › Appendix Figure S2-1/G/20240112_HeLaGFPVAPAantiGFP_STARD3S209Avrai_NT_2_w3SPI 405 DAPI.TIF]

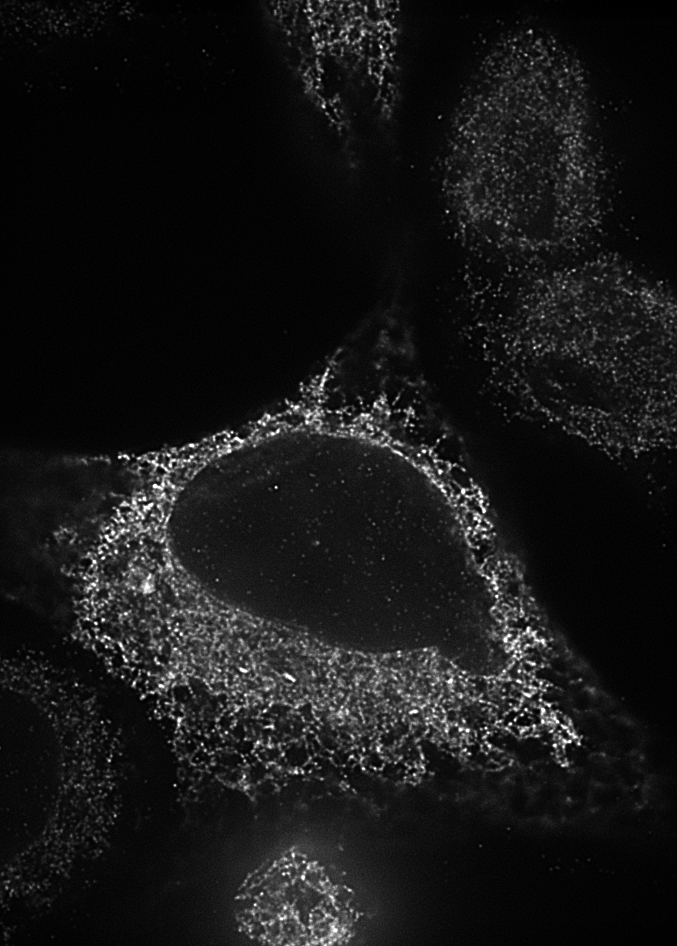

Supplement: Supplementary file 28 — Appendix Fig. S2-2 Source Data [file 44318_2026_705_MOESM28_ESM.zip › Appendix Figure S2-2/H/20240208_HeLaVAPKDMD_STARD3WT_8_SR_w1SPI 491 GFP.TIF]

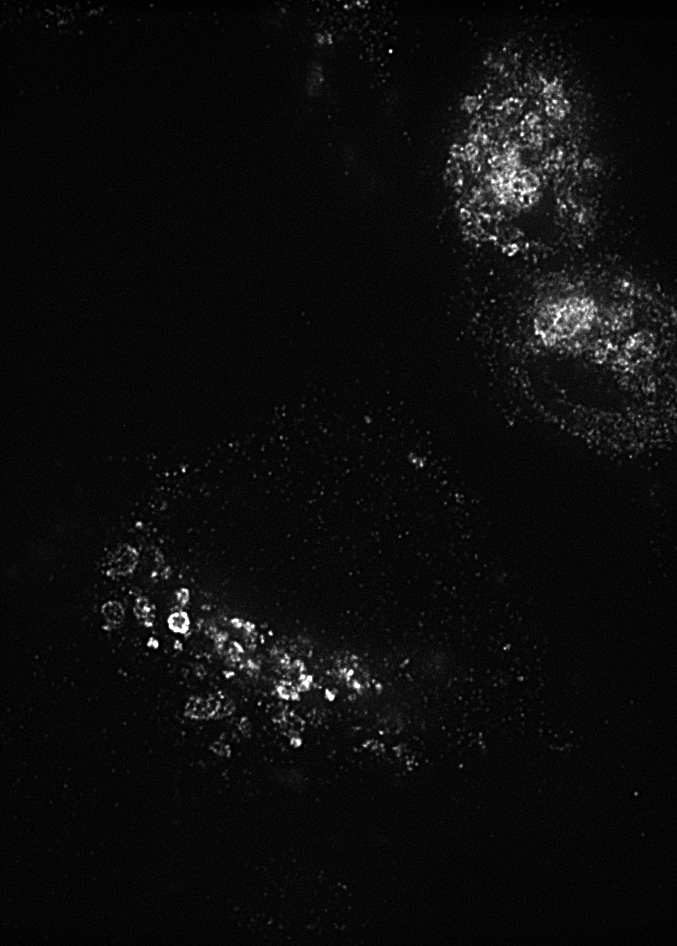

Supplement: Supplementary file 28 — Appendix Fig. S2-2 Source Data [file 44318_2026_705_MOESM28_ESM.zip › Appendix Figure S2-2/H/20240208_HeLaVAPKDMD_STARD3WT_8_SR_w2SPI 561 mCherry.TIF]

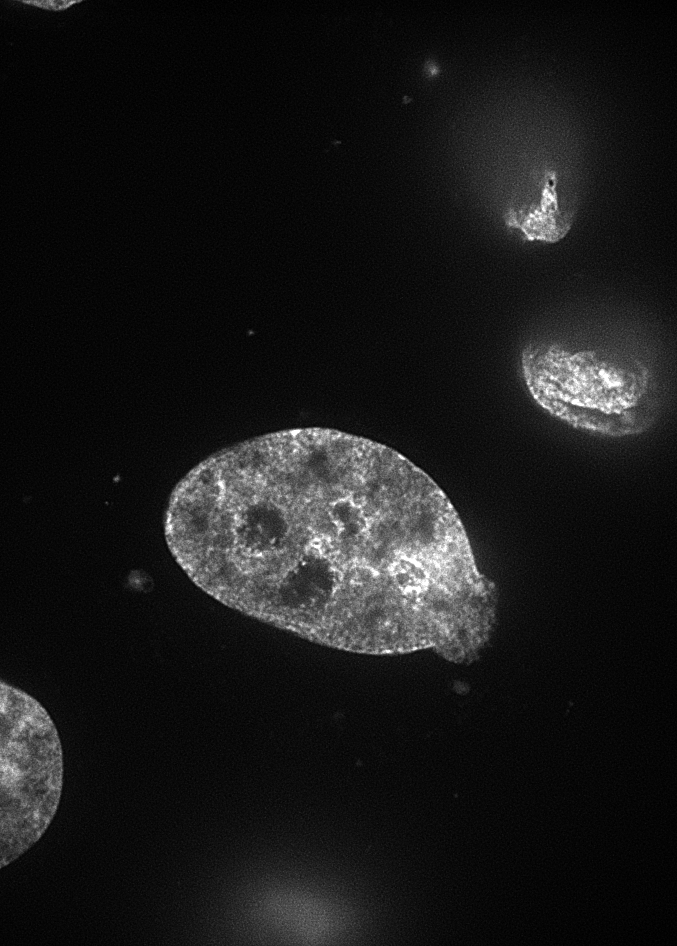

Supplement: Supplementary file 28 — Appendix Fig. S2-2 Source Data [file 44318_2026_705_MOESM28_ESM.zip › Appendix Figure S2-2/H/20240208_HeLaVAPKDMD_STARD3WT_8_SR_w3SPI 405 DAPI.TIF]

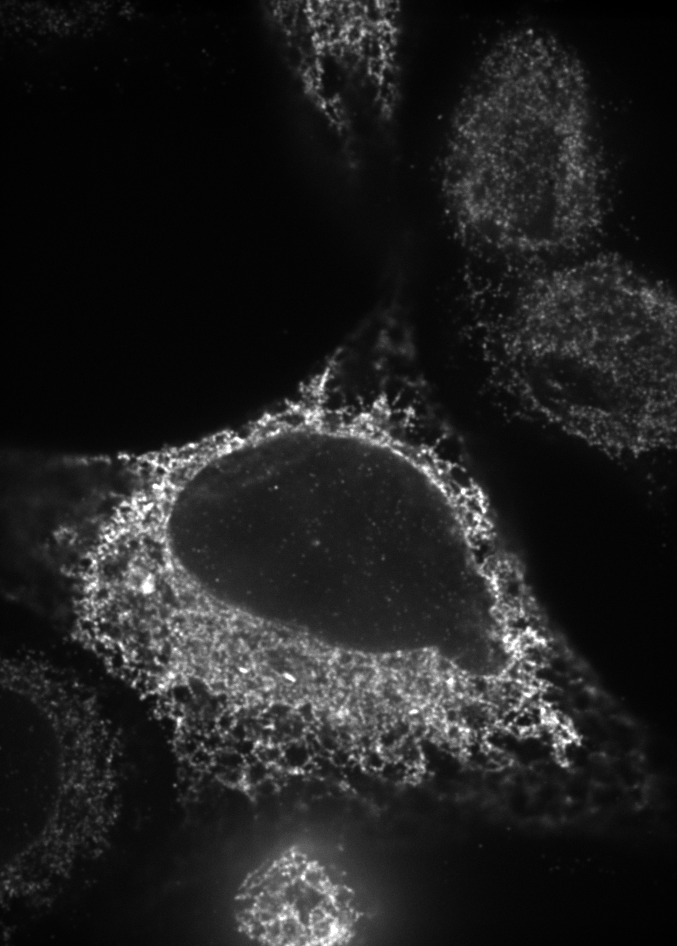

Supplement: Supplementary file 28 — Appendix Fig. S2-2 Source Data [file 44318_2026_705_MOESM28_ESM.zip › Appendix Figure S2-2/H/20240208_HeLaVAPKDMD_STARD3WT_8_w1SPI 491 GFP.TIF]

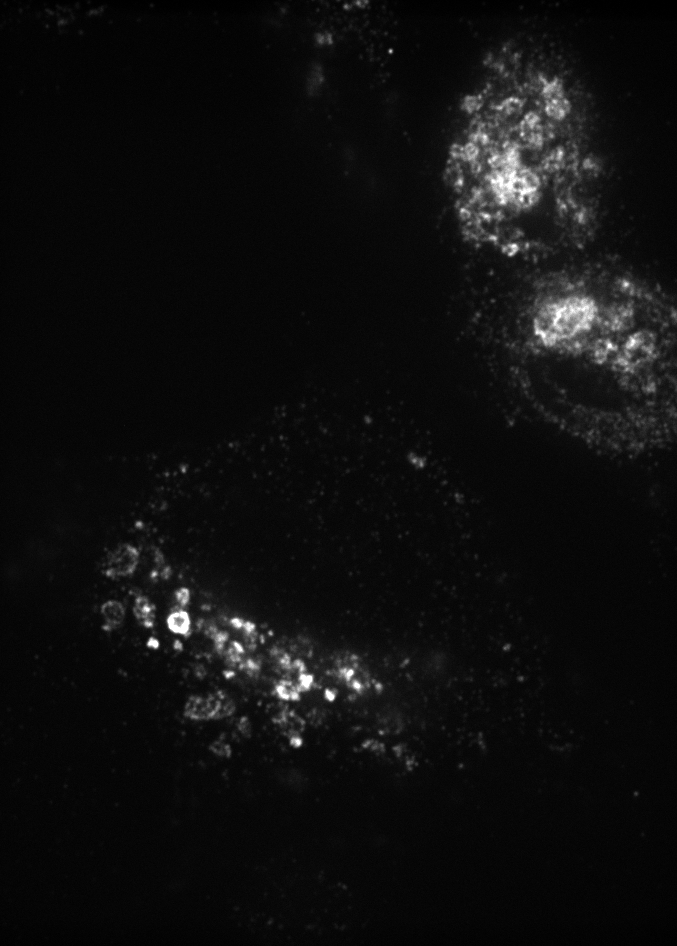

Supplement: Supplementary file 28 — Appendix Fig. S2-2 Source Data [file 44318_2026_705_MOESM28_ESM.zip › Appendix Figure S2-2/H/20240208_HeLaVAPKDMD_STARD3WT_8_w2SPI 561 mCherry.TIF]

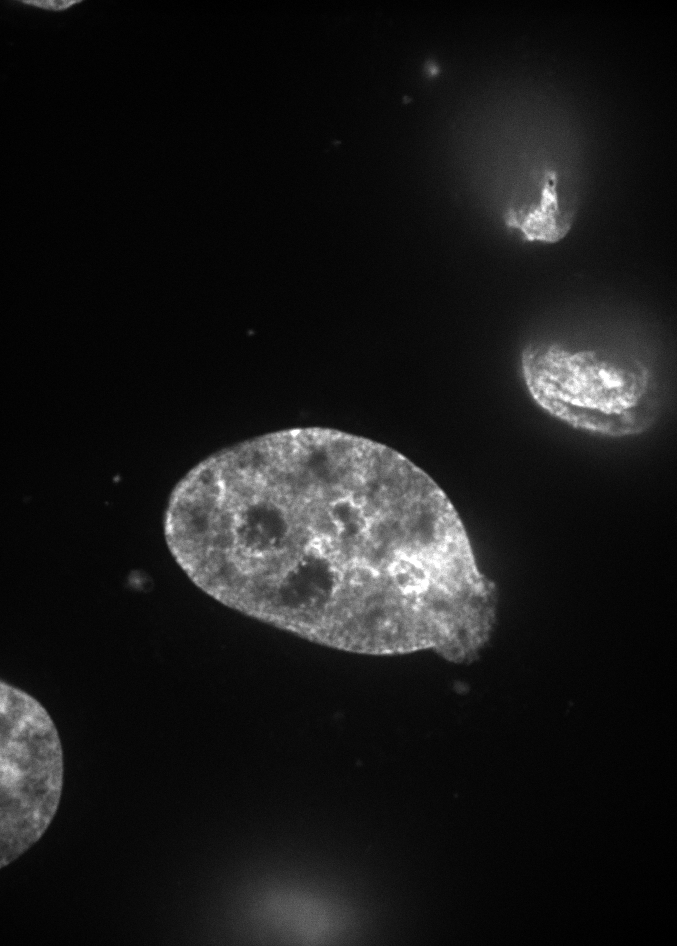

Supplement: Supplementary file 28 — Appendix Fig. S2-2 Source Data [file 44318_2026_705_MOESM28_ESM.zip › Appendix Figure S2-2/H/20240208_HeLaVAPKDMD_STARD3WT_8_w3SPI 405 DAPI.TIF]

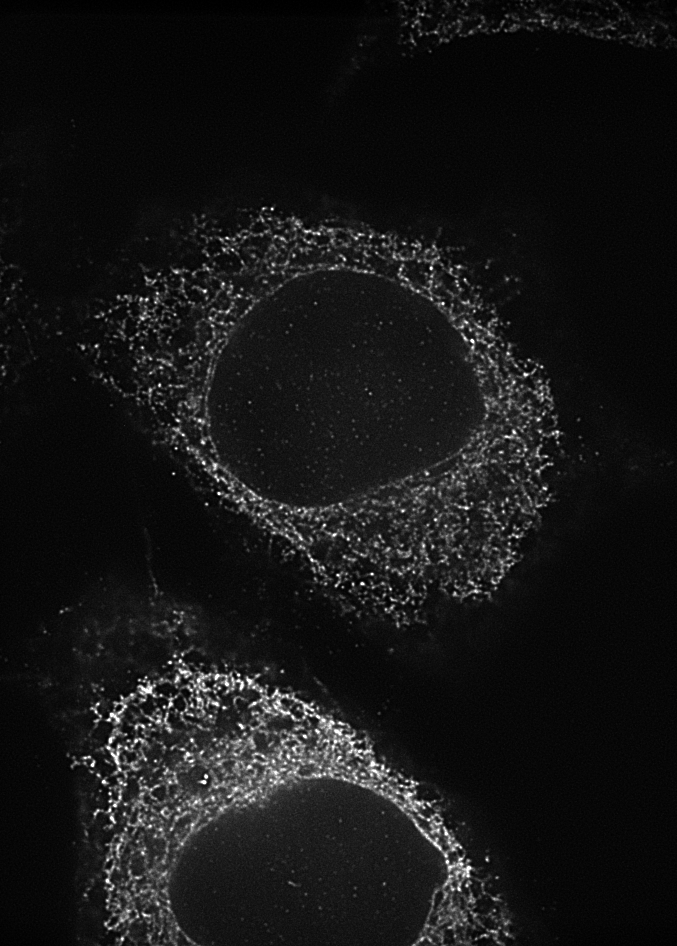

Supplement: Supplementary file 28 — Appendix Fig. S2-2 Source Data [file 44318_2026_705_MOESM28_ESM.zip › Appendix Figure S2-2/I/20240112_HeLaGFPVAPAantiGFP_STARD3_CHIR_8_SR_w1SPI 491 GFP.TIF]

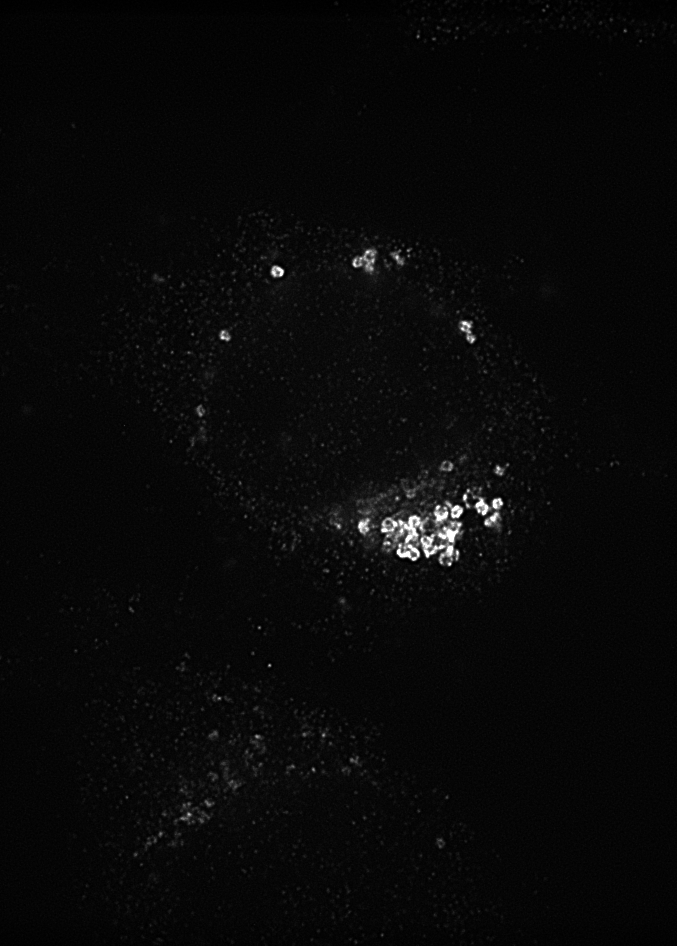

Supplement: Supplementary file 28 — Appendix Fig. S2-2 Source Data [file 44318_2026_705_MOESM28_ESM.zip › Appendix Figure S2-2/I/20240112_HeLaGFPVAPAantiGFP_STARD3_CHIR_8_SR_w2SPI 561 mCherry.TIF]

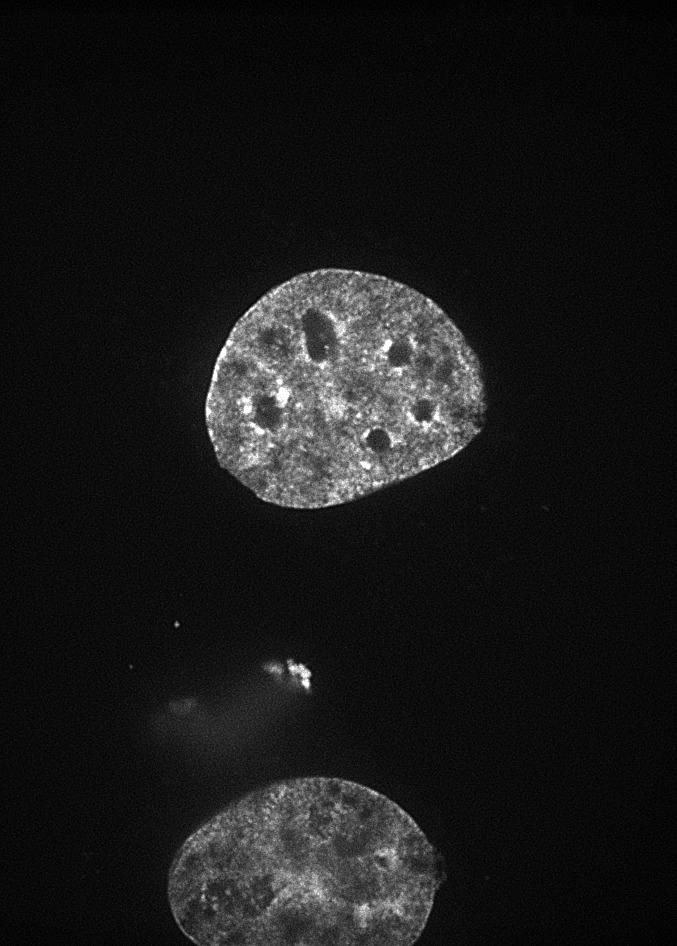

Supplement: Supplementary file 28 — Appendix Fig. S2-2 Source Data [file 44318_2026_705_MOESM28_ESM.zip › Appendix Figure S2-2/I/20240112_HeLaGFPVAPAantiGFP_STARD3_CHIR_8_SR_w3SPI 405 DAPI.TIF]

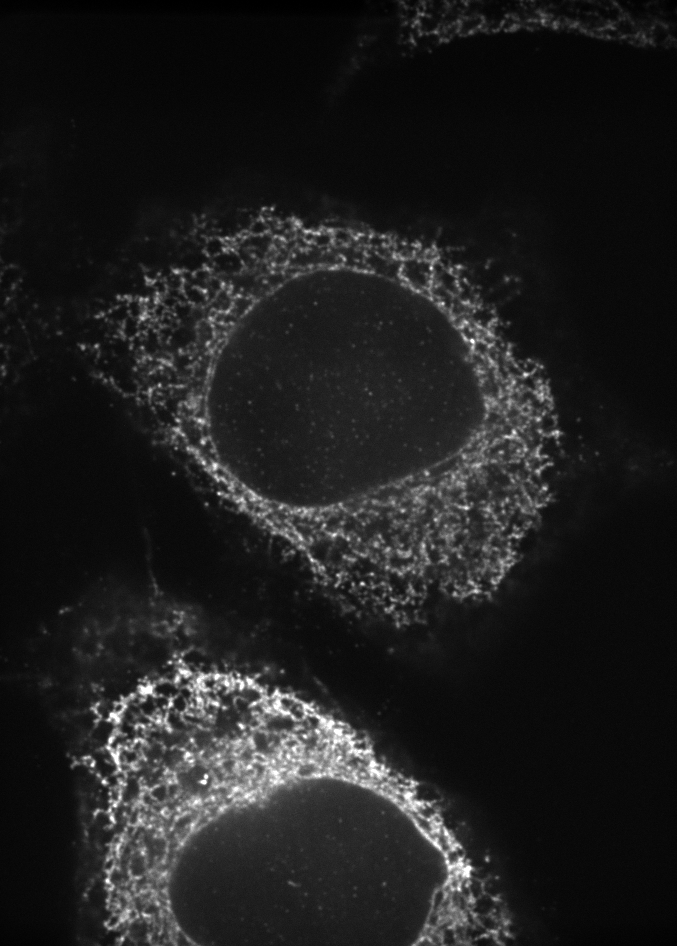

Supplement: Supplementary file 28 — Appendix Fig. S2-2 Source Data [file 44318_2026_705_MOESM28_ESM.zip › Appendix Figure S2-2/I/20240112_HeLaGFPVAPAantiGFP_STARD3_CHIR_8_w1SPI 491 GFP.TIF]

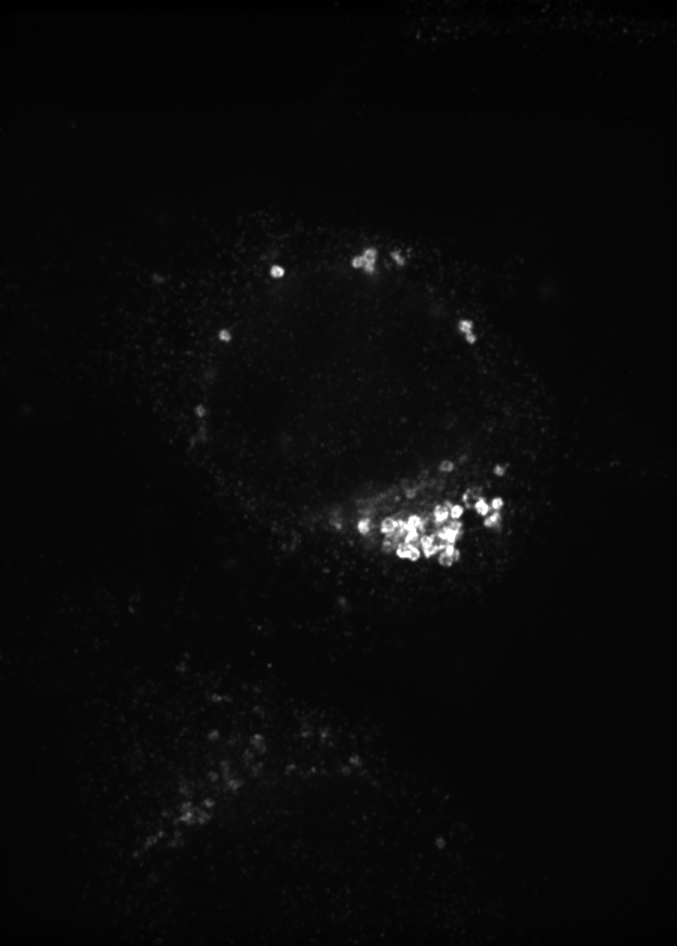

Supplement: Supplementary file 28 — Appendix Fig. S2-2 Source Data [file 44318_2026_705_MOESM28_ESM.zip › Appendix Figure S2-2/I/20240112_HeLaGFPVAPAantiGFP_STARD3_CHIR_8_w2SPI 561 mCherry.TIF]

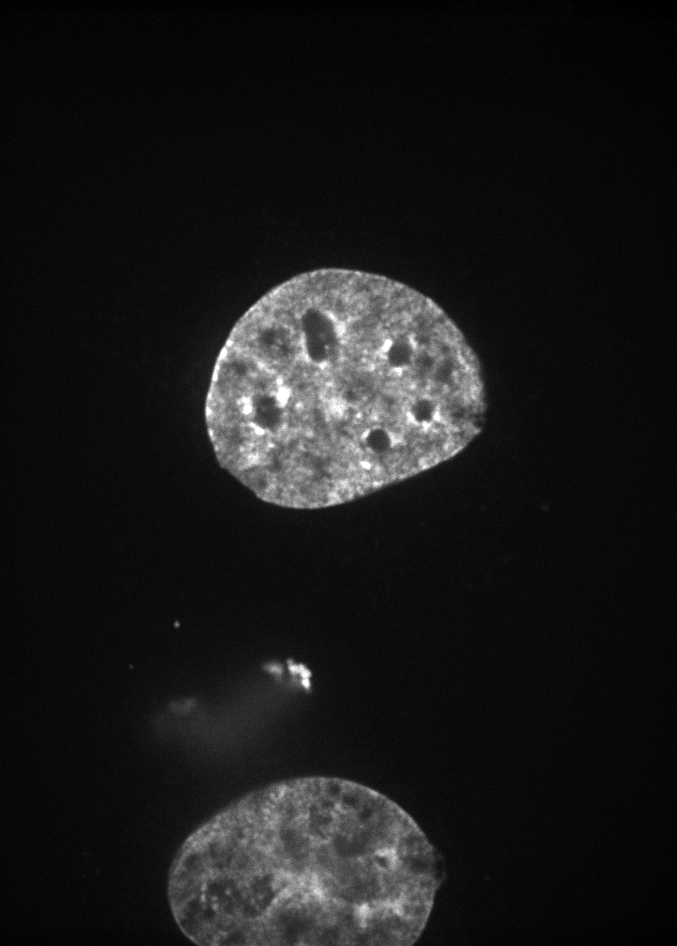

Supplement: Supplementary file 28 — Appendix Fig. S2-2 Source Data [file 44318_2026_705_MOESM28_ESM.zip › Appendix Figure S2-2/I/20240112_HeLaGFPVAPAantiGFP_STARD3_CHIR_8_w3SPI 405 DAPI.TIF]

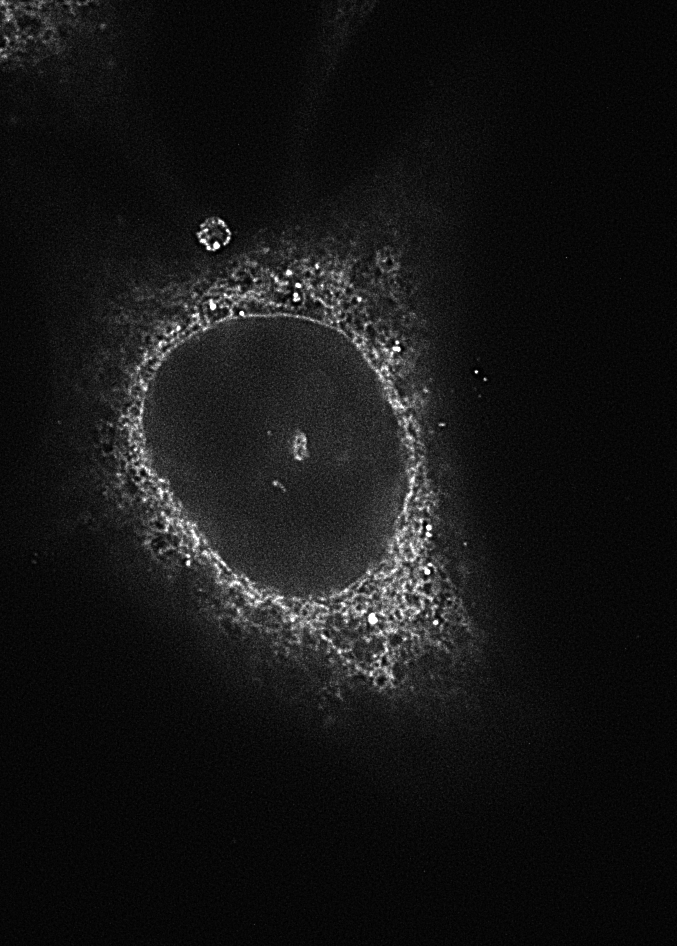

Supplement: Supplementary file 28 — Appendix Fig. S2-2 Source Data [file 44318_2026_705_MOESM28_ESM.zip › Appendix Figure S2-2/K/20241112_HeLaGFPVAPBg_STARD3r_NT_noexp_1_SR_w1SPI 491 GFP.TIF]

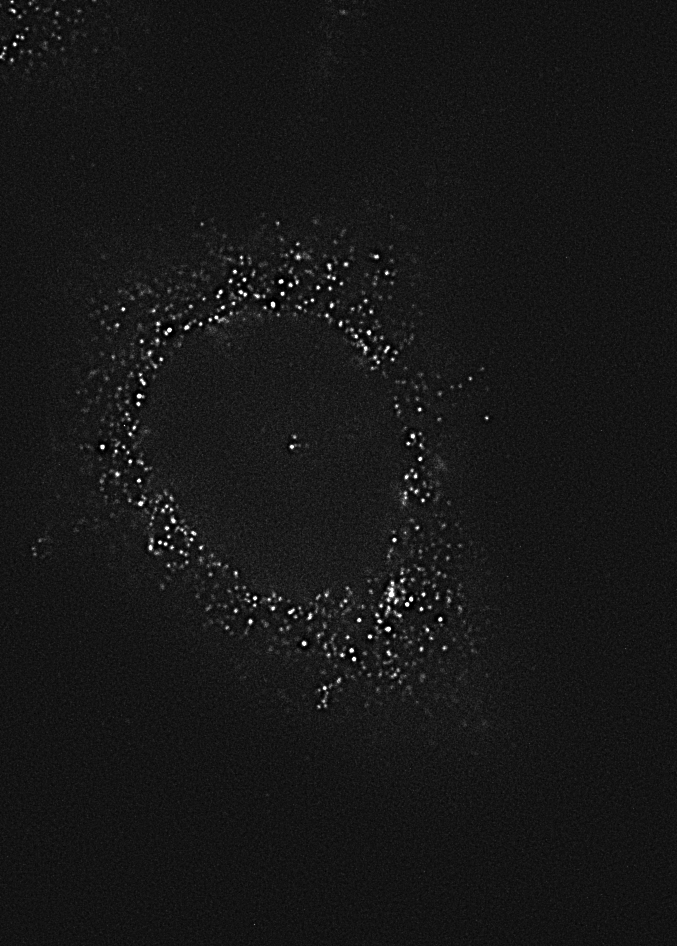

Supplement: Supplementary file 28 — Appendix Fig. S2-2 Source Data [file 44318_2026_705_MOESM28_ESM.zip › Appendix Figure S2-2/K/20241112_HeLaGFPVAPBg_STARD3r_NT_noexp_1_SR_w2SPI 561 mCherry.TIF]

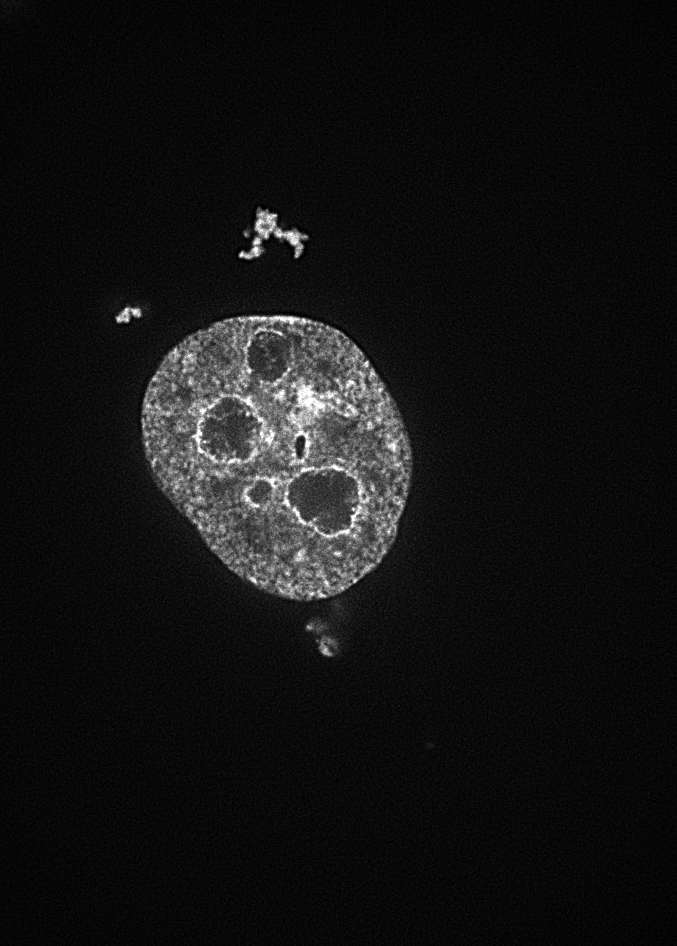

Supplement: Supplementary file 28 — Appendix Fig. S2-2 Source Data [file 44318_2026_705_MOESM28_ESM.zip › Appendix Figure S2-2/K/20241112_HeLaGFPVAPBg_STARD3r_NT_noexp_1_SR_w3SPI 405 DAPI.TIF]

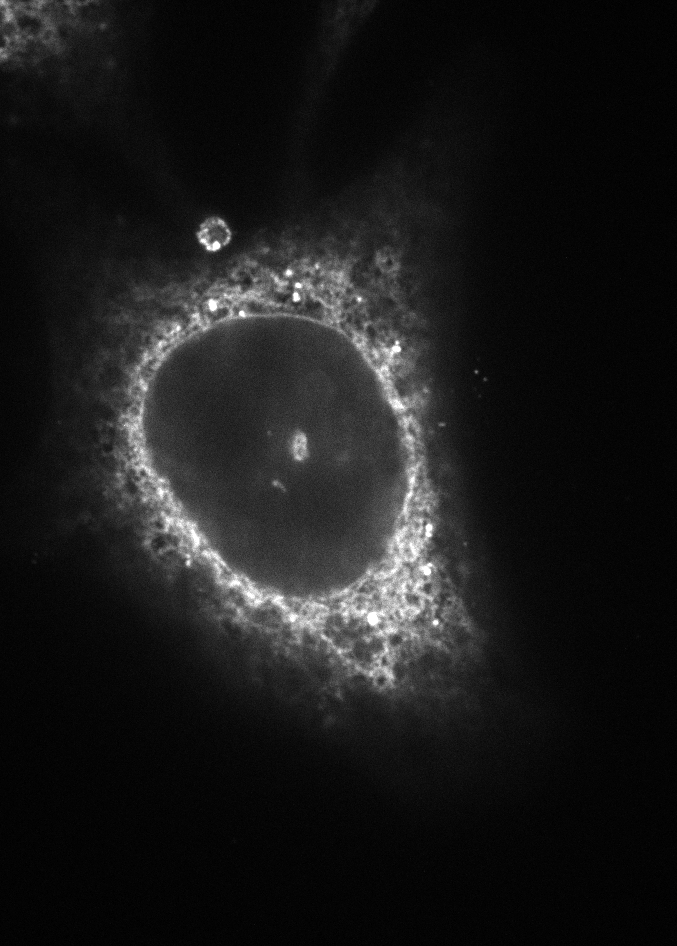

Supplement: Supplementary file 28 — Appendix Fig. S2-2 Source Data [file 44318_2026_705_MOESM28_ESM.zip › Appendix Figure S2-2/K/20241112_HeLaGFPVAPBg_STARD3r_NT_noexp_1_w1SPI 491 GFP.TIF]

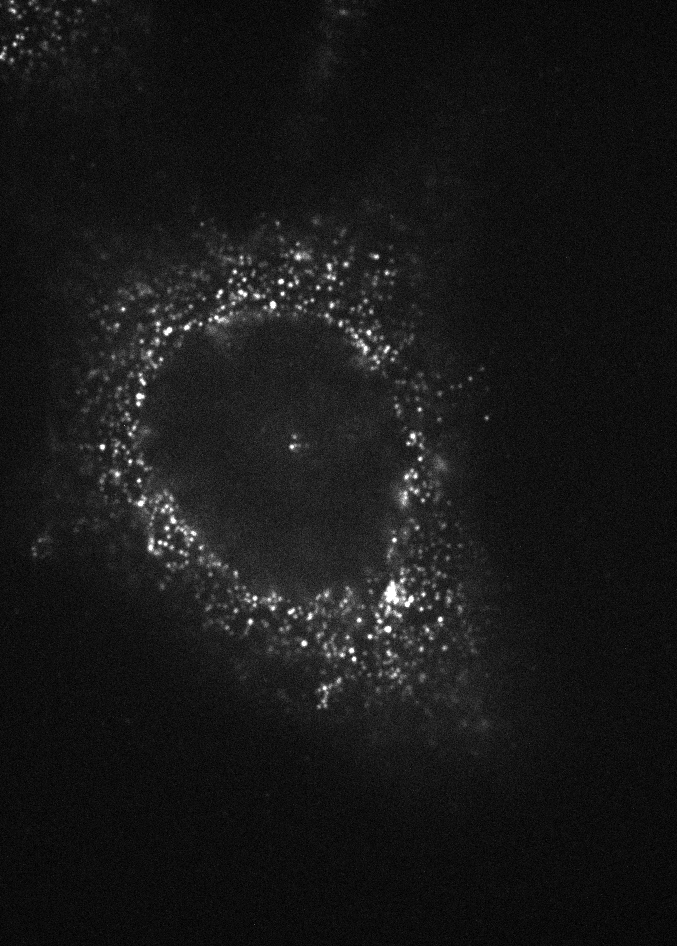

Supplement: Supplementary file 28 — Appendix Fig. S2-2 Source Data [file 44318_2026_705_MOESM28_ESM.zip › Appendix Figure S2-2/K/20241112_HeLaGFPVAPBg_STARD3r_NT_noexp_1_w2SPI 561 mCherry.TIF]

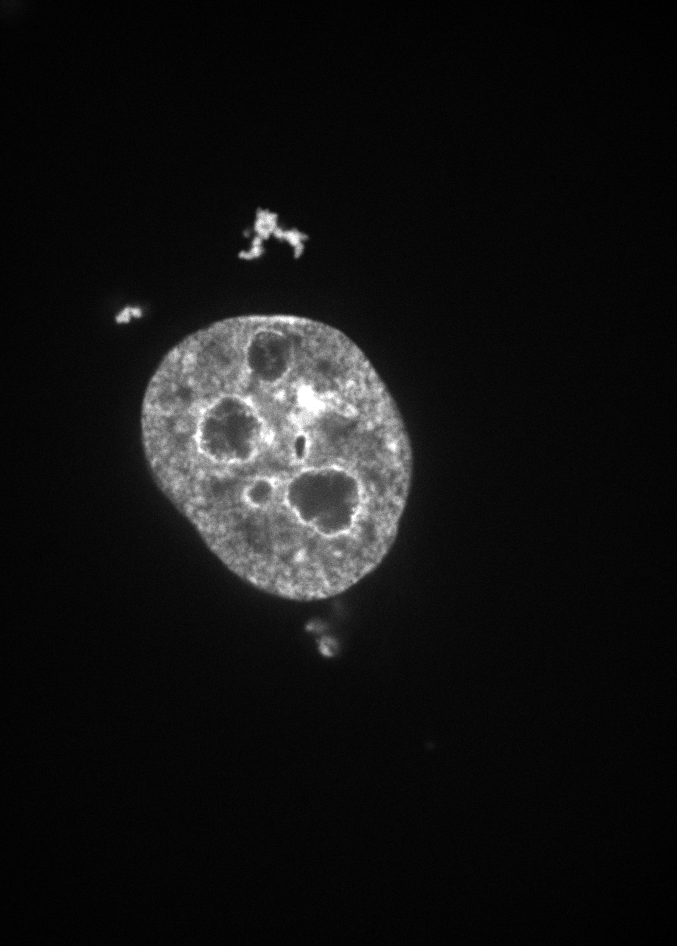

Supplement: Supplementary file 28 — Appendix Fig. S2-2 Source Data [file 44318_2026_705_MOESM28_ESM.zip › Appendix Figure S2-2/K/20241112_HeLaGFPVAPBg_STARD3r_NT_noexp_1_w3SPI 405 DAPI.TIF]

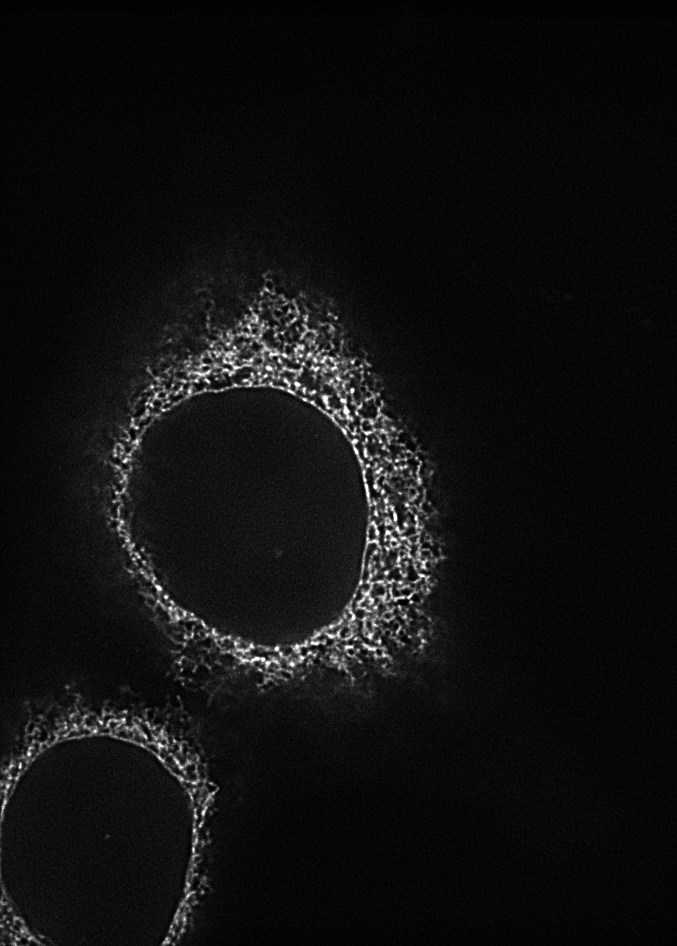

Supplement: Supplementary file 28 — Appendix Fig. S2-2 Source Data [file 44318_2026_705_MOESM28_ESM.zip › Appendix Figure S2-2/L/20241113_HeLaGFPVAPBg_STARD3r_CHIR_noexp_1_SR_w1SPI 491 GFP.TIF]

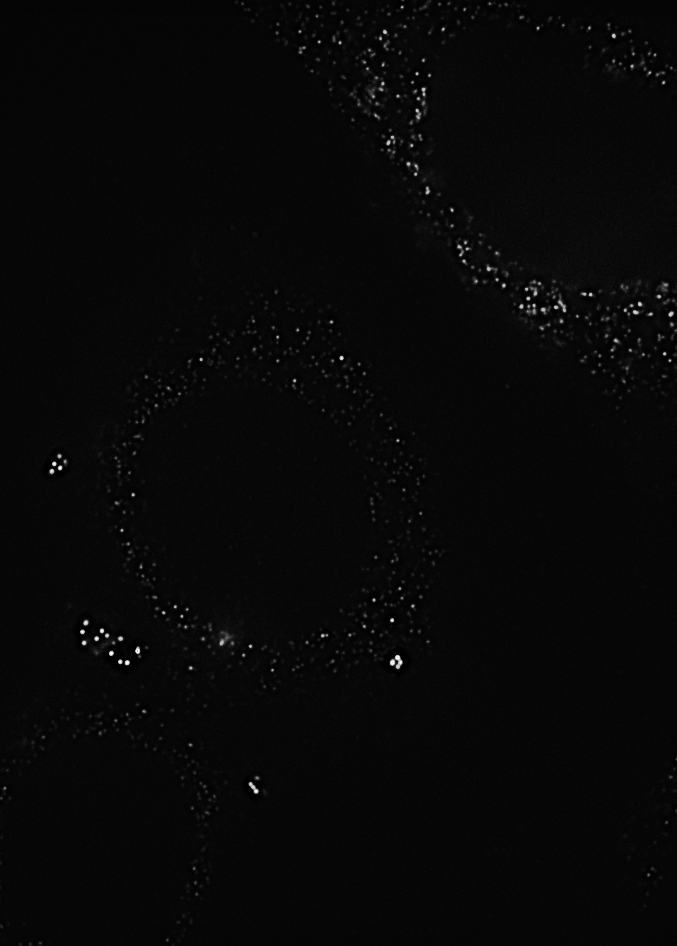

Supplement: Supplementary file 28 — Appendix Fig. S2-2 Source Data [file 44318_2026_705_MOESM28_ESM.zip › Appendix Figure S2-2/L/20241113_HeLaGFPVAPBg_STARD3r_CHIR_noexp_1_SR_w2SPI 561 mCherry.TIF]

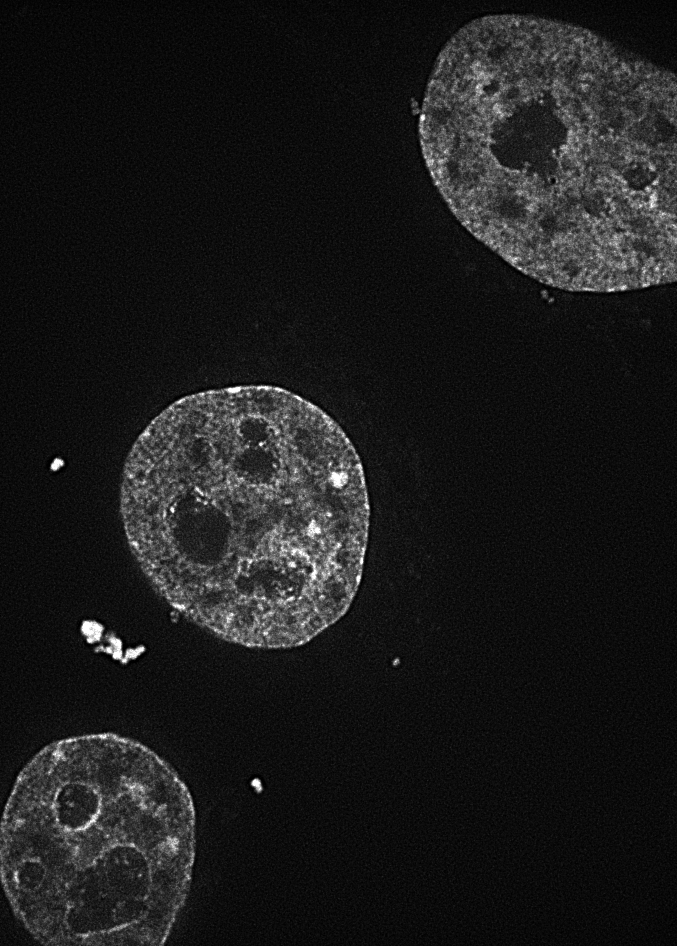

Supplement: Supplementary file 28 — Appendix Fig. S2-2 Source Data [file 44318_2026_705_MOESM28_ESM.zip › Appendix Figure S2-2/L/20241113_HeLaGFPVAPBg_STARD3r_CHIR_noexp_1_SR_w3SPI 405 DAPI.TIF]

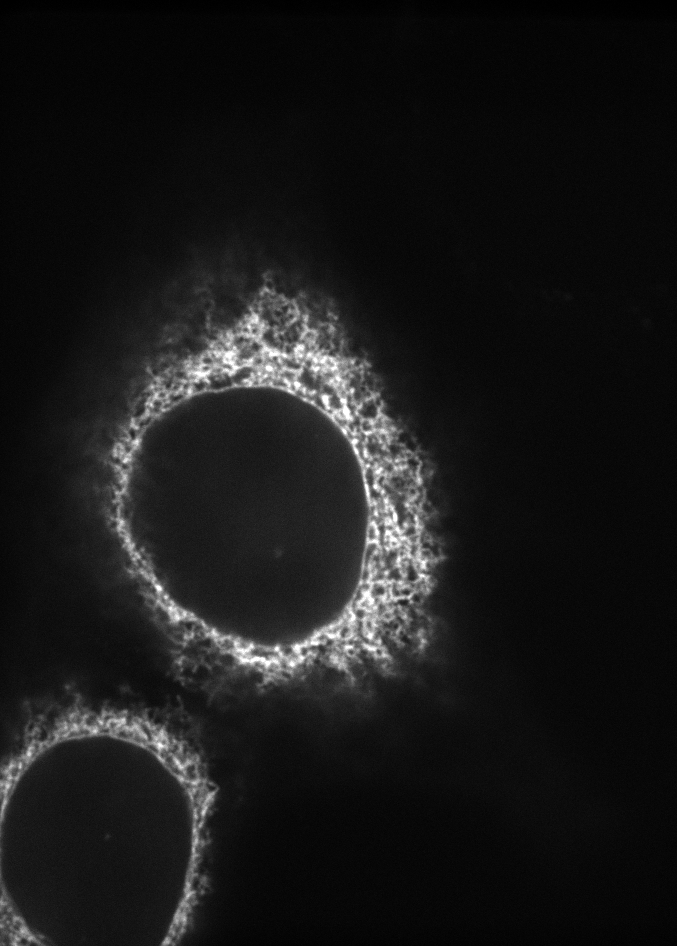

Supplement: Supplementary file 28 — Appendix Fig. S2-2 Source Data [file 44318_2026_705_MOESM28_ESM.zip › Appendix Figure S2-2/L/20241113_HeLaGFPVAPBg_STARD3r_CHIR_noexp_1_w1SPI 491 GFP.TIF]

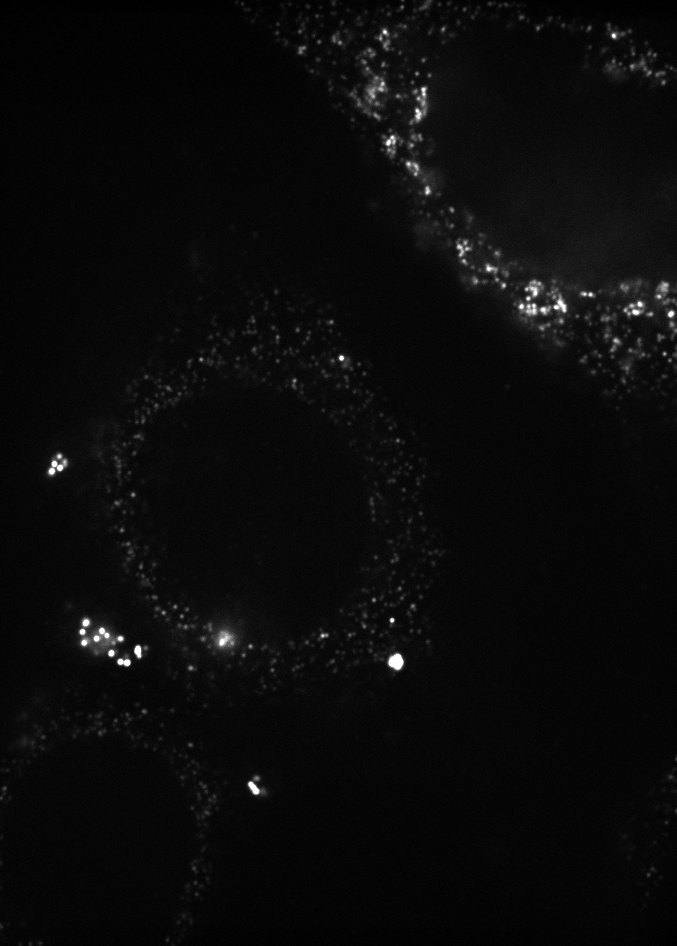

Supplement: Supplementary file 28 — Appendix Fig. S2-2 Source Data [file 44318_2026_705_MOESM28_ESM.zip › Appendix Figure S2-2/L/20241113_HeLaGFPVAPBg_STARD3r_CHIR_noexp_1_w2SPI 561 mCherry.TIF]

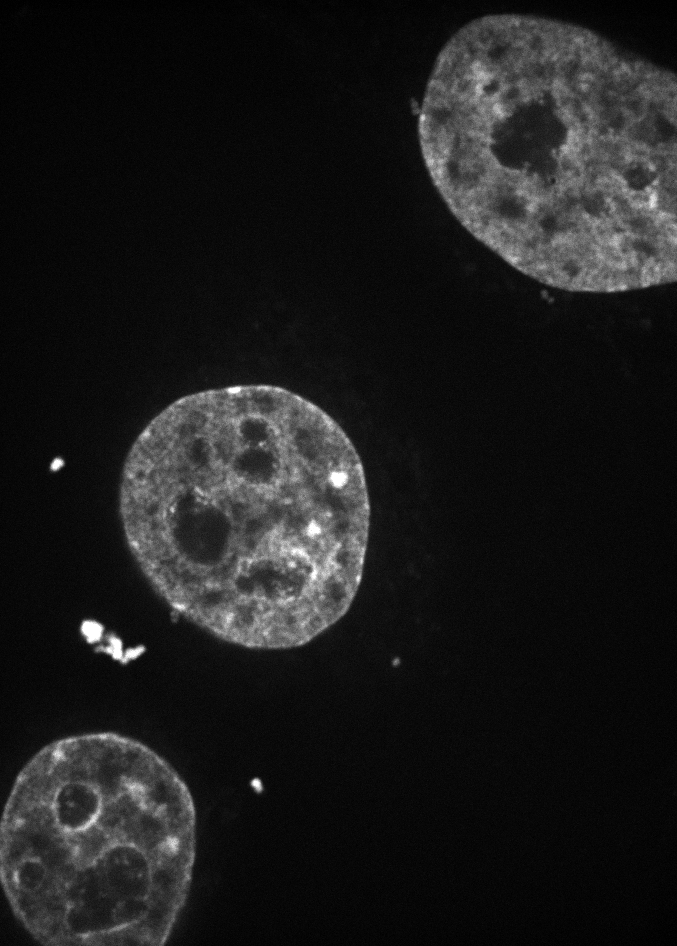

Supplement: Supplementary file 28 — Appendix Fig. S2-2 Source Data [file 44318_2026_705_MOESM28_ESM.zip › Appendix Figure S2-2/L/20241113_HeLaGFPVAPBg_STARD3r_CHIR_noexp_1_w3SPI 405 DAPI.TIF]

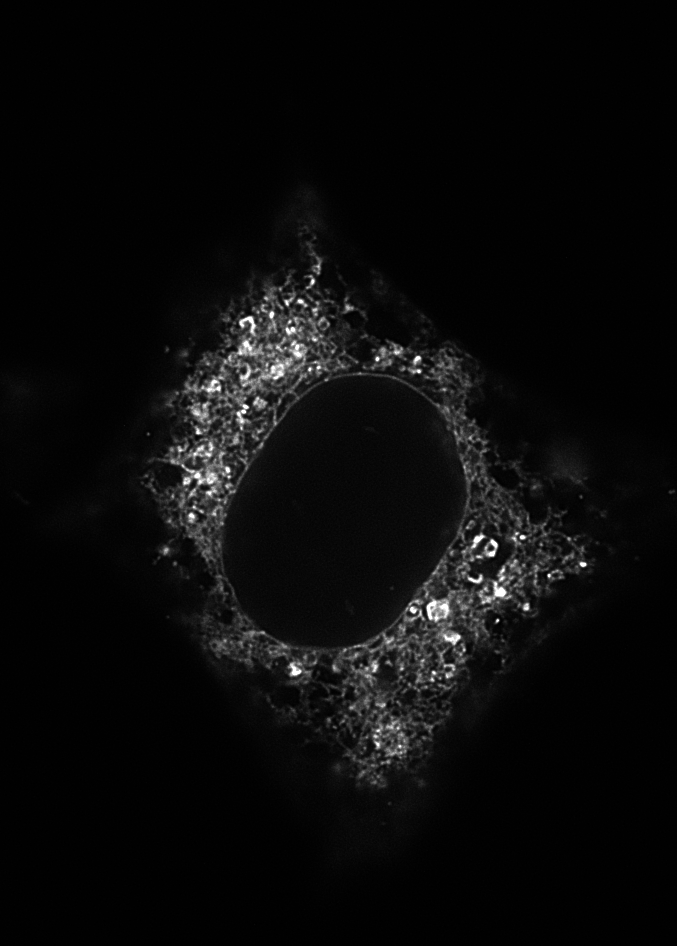

Supplement: Supplementary file 29 — Appendix Fig. S2-3 Source Data [file 44318_2026_705_MOESM29_ESM.zip › Appendix Figure S2-3/M/20241029_HeLaGFPVAPBg_STARD3r_NT_2_SR_w1SPI 491 GFP.TIF]

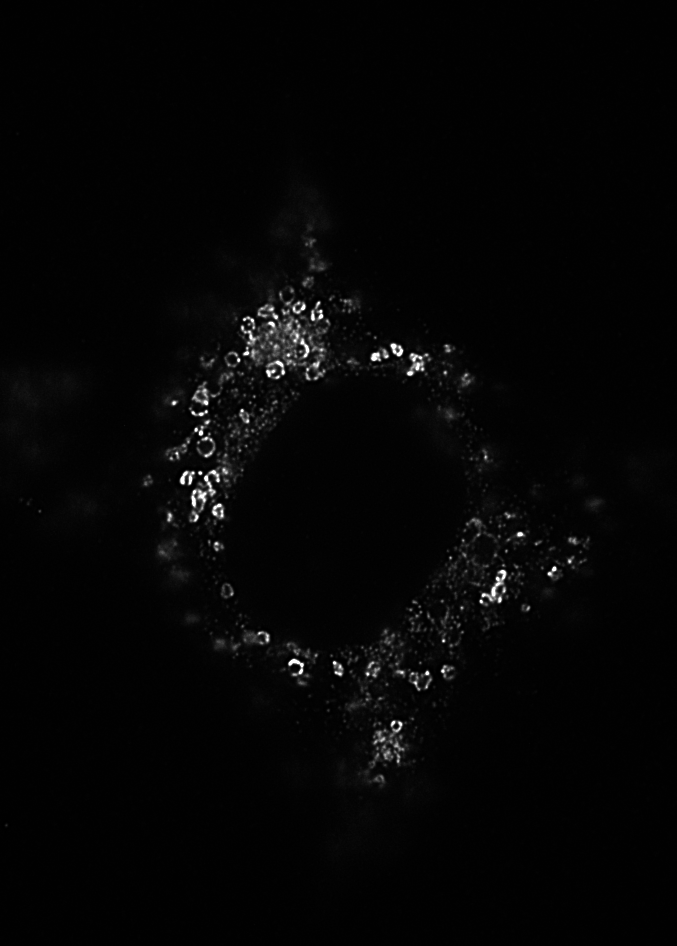

Supplement: Supplementary file 29 — Appendix Fig. S2-3 Source Data [file 44318_2026_705_MOESM29_ESM.zip › Appendix Figure S2-3/M/20241029_HeLaGFPVAPBg_STARD3r_NT_2_SR_w2SPI 561 mCherry.TIF]

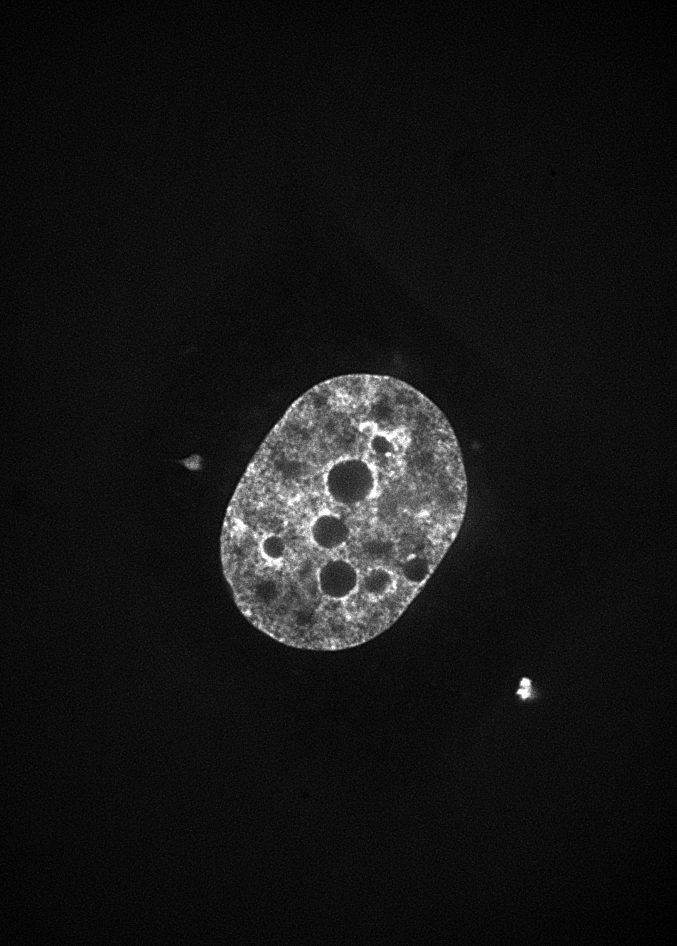

Supplement: Supplementary file 29 — Appendix Fig. S2-3 Source Data [file 44318_2026_705_MOESM29_ESM.zip › Appendix Figure S2-3/M/20241029_HeLaGFPVAPBg_STARD3r_NT_2_SR_w3SPI 405 DAPI.TIF]

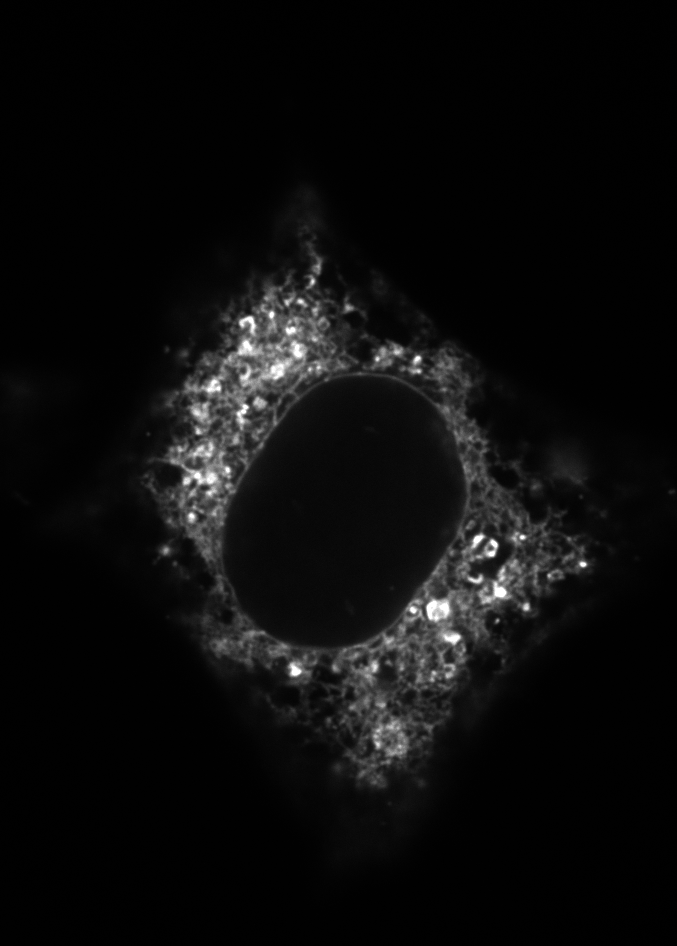

Supplement: Supplementary file 29 — Appendix Fig. S2-3 Source Data [file 44318_2026_705_MOESM29_ESM.zip › Appendix Figure S2-3/M/20241029_HeLaGFPVAPBg_STARD3r_NT_2_w1SPI 491 GFP.TIF]

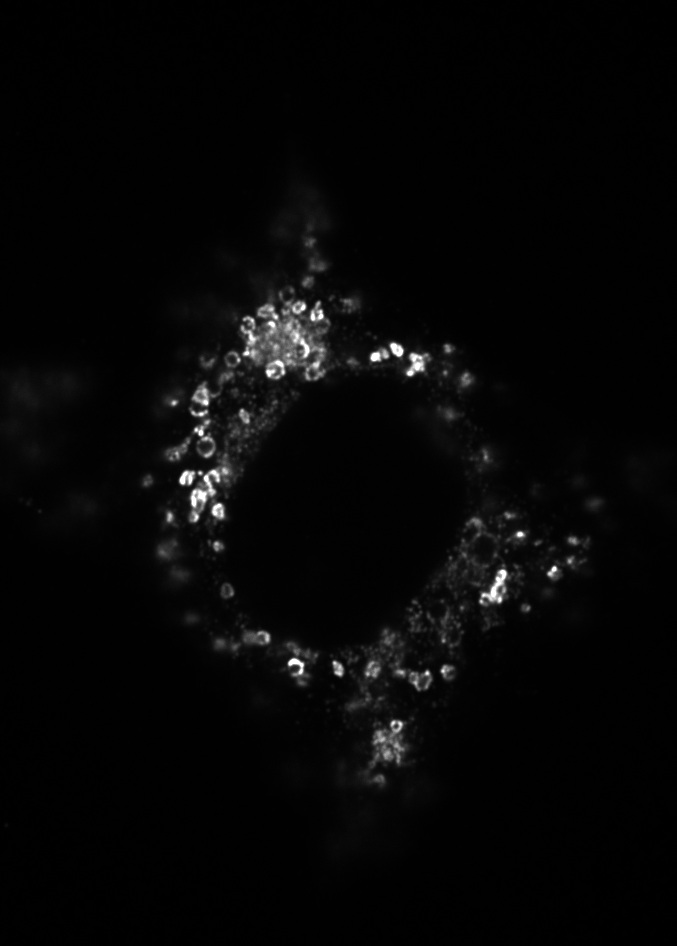

Supplement: Supplementary file 29 — Appendix Fig. S2-3 Source Data [file 44318_2026_705_MOESM29_ESM.zip › Appendix Figure S2-3/M/20241029_HeLaGFPVAPBg_STARD3r_NT_2_w2SPI 561 mCherry.TIF]

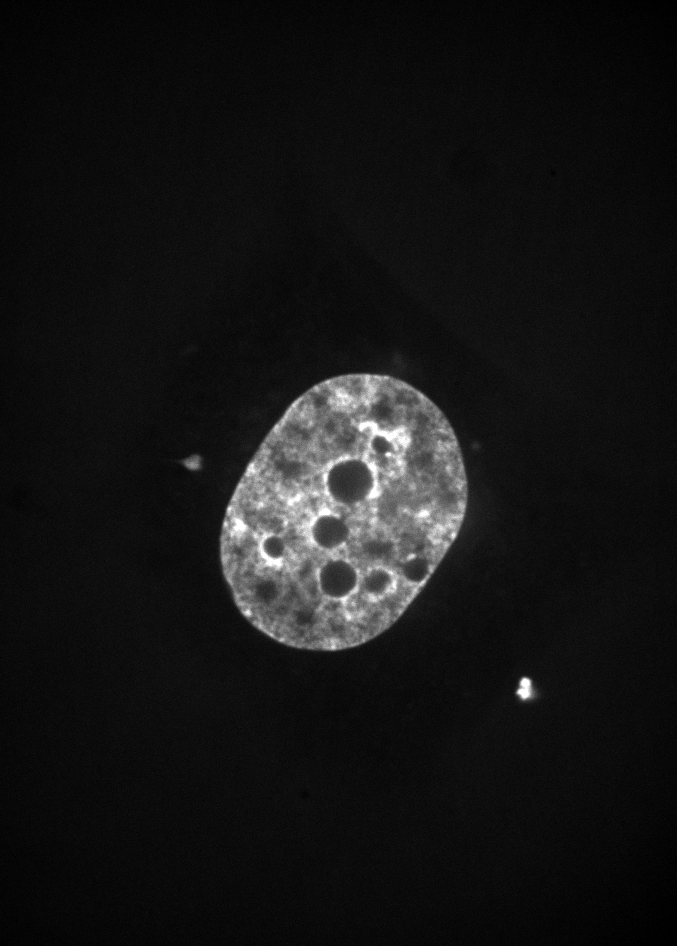

Supplement: Supplementary file 29 — Appendix Fig. S2-3 Source Data [file 44318_2026_705_MOESM29_ESM.zip › Appendix Figure S2-3/M/20241029_HeLaGFPVAPBg_STARD3r_NT_2_w3SPI 405 DAPI.TIF]

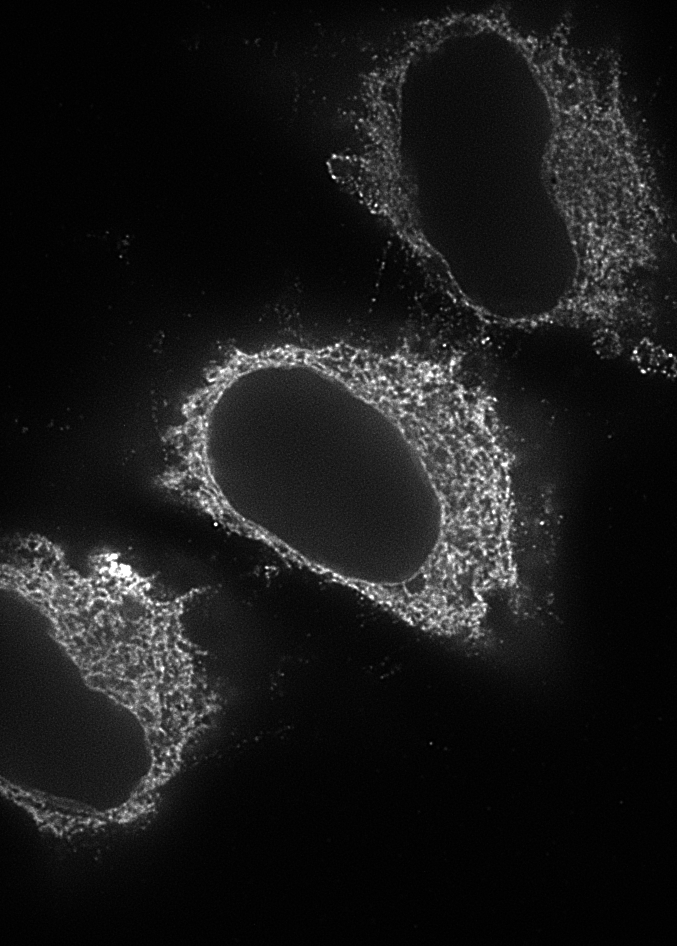

Supplement: Supplementary file 29 — Appendix Fig. S2-3 Source Data [file 44318_2026_705_MOESM29_ESM.zip › Appendix Figure S2-3/N/20241028_HeLaGFPVAPBg_STARD3r_CHIR_2_SR_w1SPI 491 GFP.TIF]

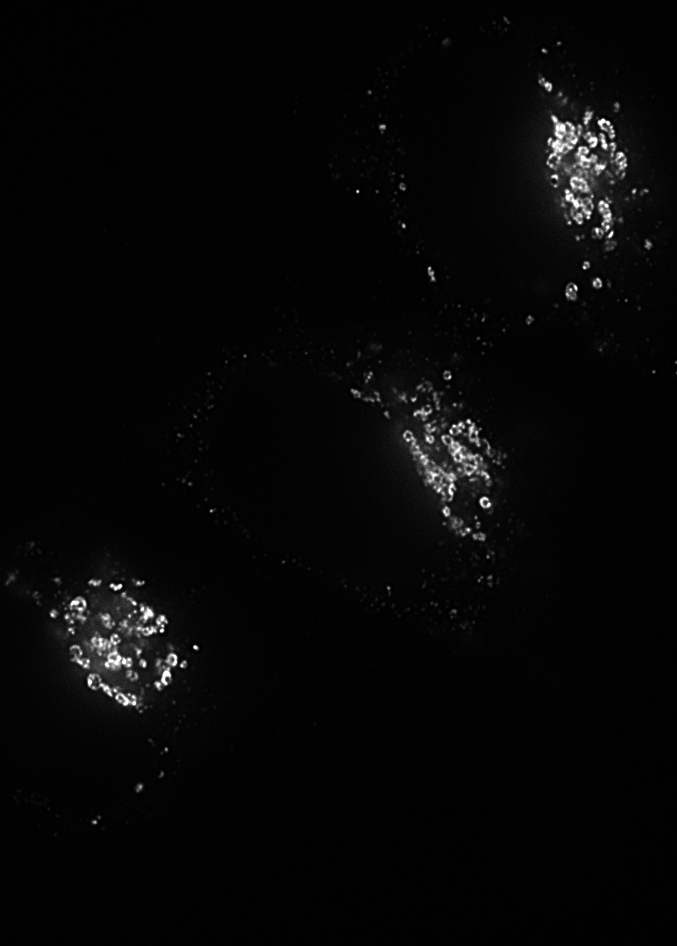

Supplement: Supplementary file 29 — Appendix Fig. S2-3 Source Data [file 44318_2026_705_MOESM29_ESM.zip › Appendix Figure S2-3/N/20241028_HeLaGFPVAPBg_STARD3r_CHIR_2_SR_w2SPI 561 mCherry.TIF]

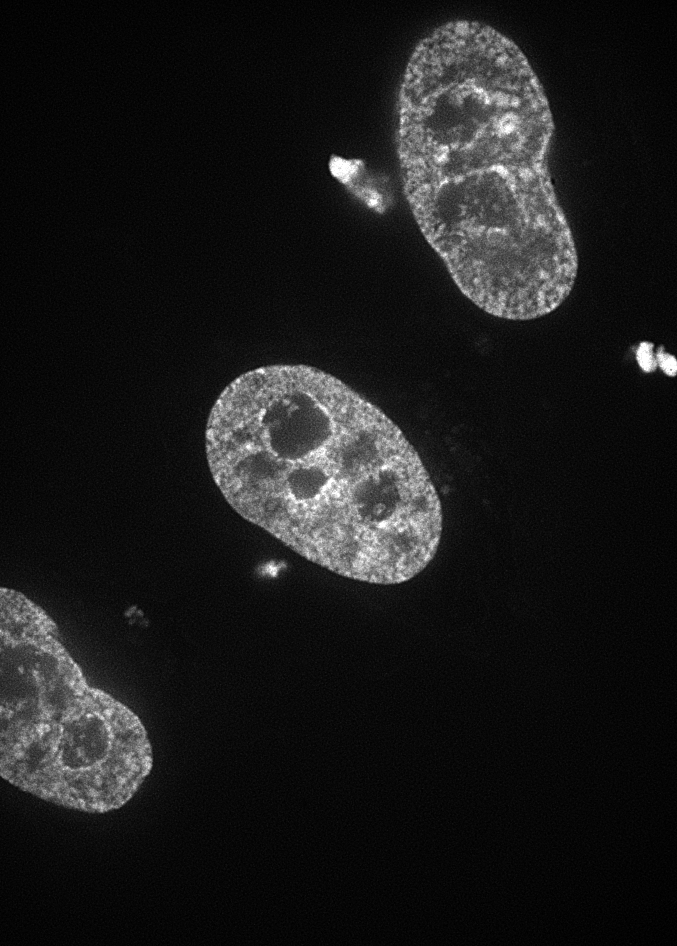

Supplement: Supplementary file 29 — Appendix Fig. S2-3 Source Data [file 44318_2026_705_MOESM29_ESM.zip › Appendix Figure S2-3/N/20241028_HeLaGFPVAPBg_STARD3r_CHIR_2_SR_w3SPI 405 DAPI.TIF]

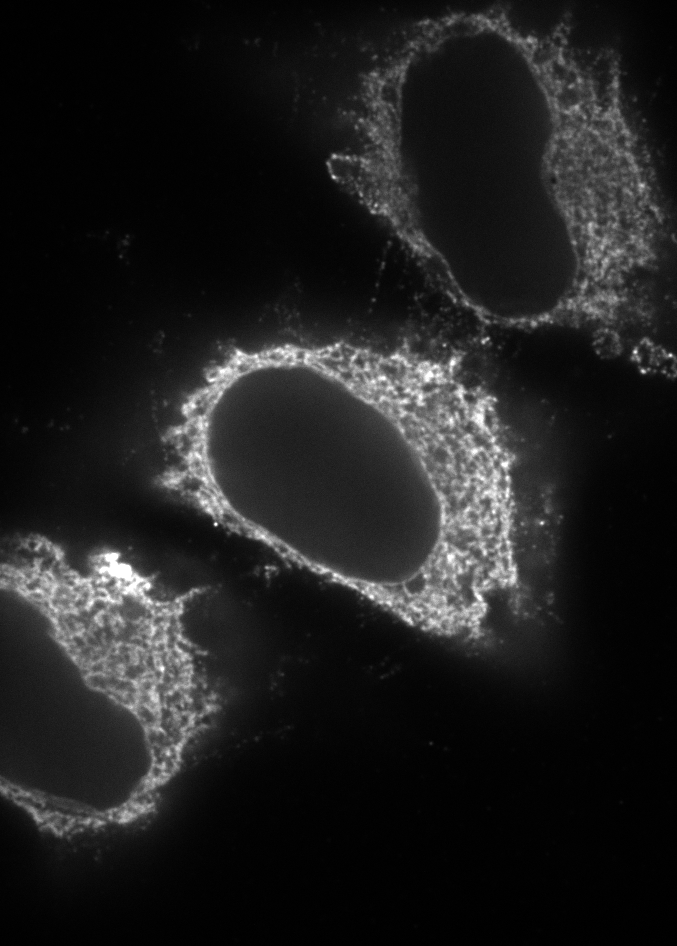

Supplement: Supplementary file 29 — Appendix Fig. S2-3 Source Data [file 44318_2026_705_MOESM29_ESM.zip › Appendix Figure S2-3/N/20241028_HeLaGFPVAPBg_STARD3r_CHIR_2_w1SPI 491 GFP.TIF]

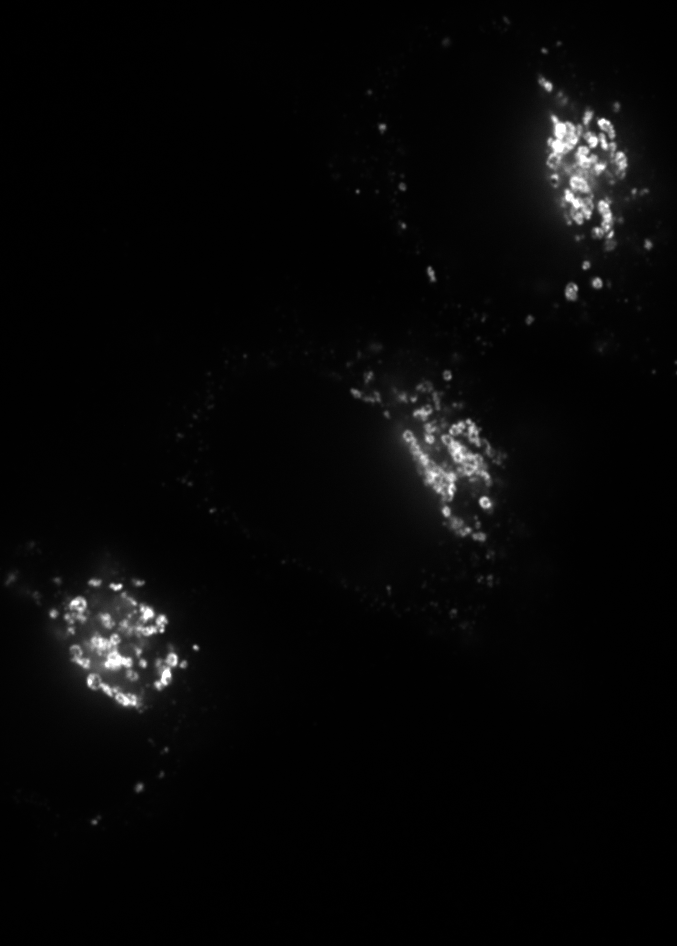

Supplement: Supplementary file 29 — Appendix Fig. S2-3 Source Data [file 44318_2026_705_MOESM29_ESM.zip › Appendix Figure S2-3/N/20241028_HeLaGFPVAPBg_STARD3r_CHIR_2_w2SPI 561 mCherry.TIF]

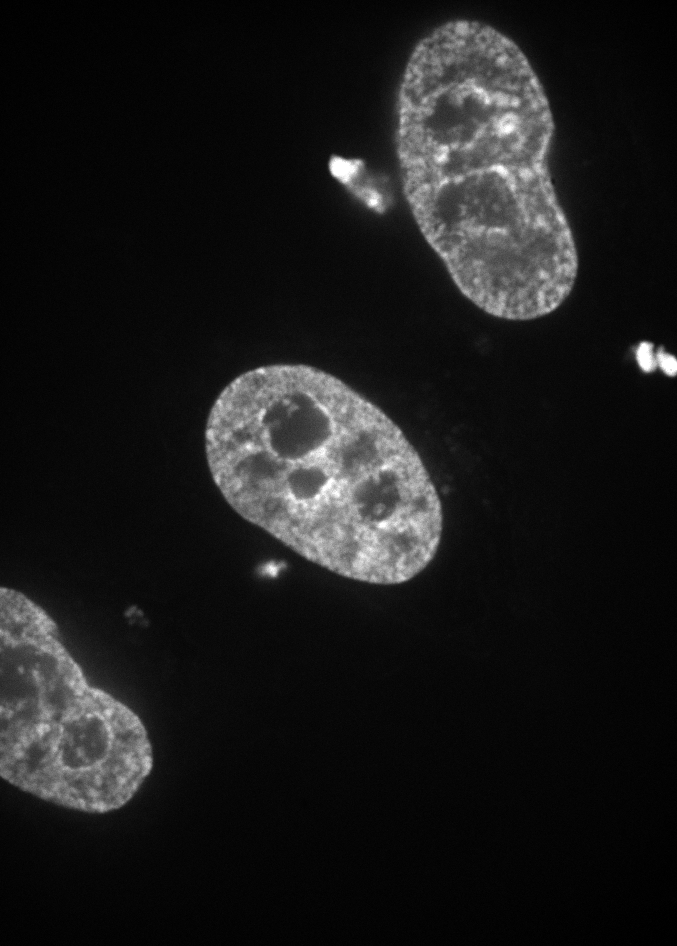

Supplement: Supplementary file 29 — Appendix Fig. S2-3 Source Data [file 44318_2026_705_MOESM29_ESM.zip › Appendix Figure S2-3/N/20241028_HeLaGFPVAPBg_STARD3r_CHIR_2_w3SPI 405 DAPI.TIF]

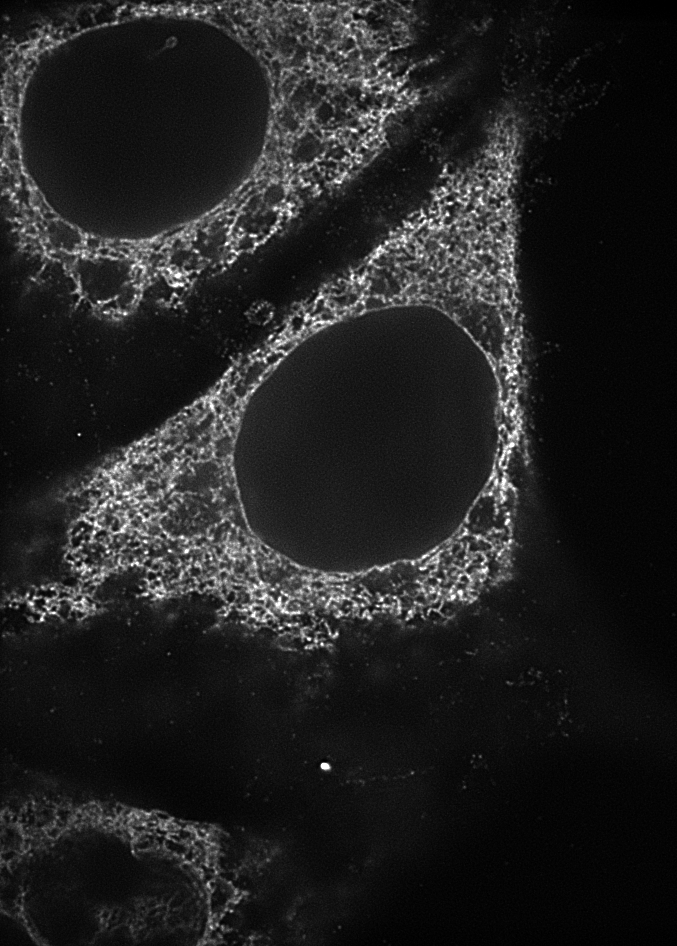

Supplement: Supplementary file 29 — Appendix Fig. S2-3 Source Data [file 44318_2026_705_MOESM29_ESM.zip › Appendix Figure S2-3/O/20241029_HeLaGFPVAPBg_STARD3S209Ar_NT_8_SR_w1SPI 491 GFP.TIF]
