# Supplementary material for: STARD3 regulates lysosome positioning and contacts via a GSK3-controlled phosphorylation switch
Source: EMBO J. 2026 Feb 25;45(7):2239–77. doi: 10.1038/s44318-026-00705-3 (PMC13044316; doi:10.1038/s44318-026-00705-3)
Supplement: Supplementary file 27 — Appendix Fig. S2-1 Source Data [file 44318_2026_705_MOESM27_ESM.zip › Appendix Figure S2-1/A/GFP Trap-VAPA_WB.pdf]

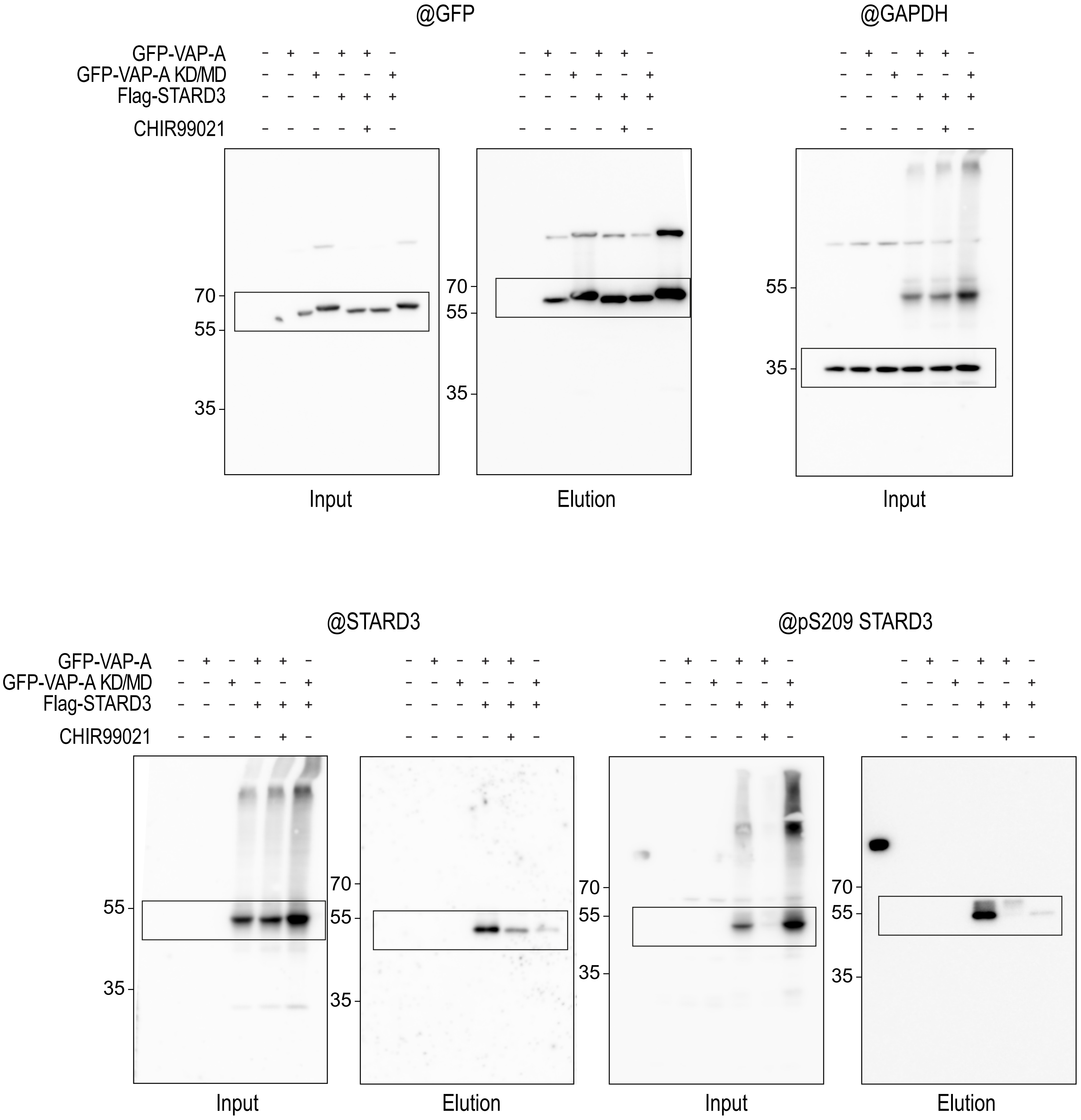

@GFP
@GAPDH
GFP-VAP-A
GFP-VAP-A KD/MD
Flag-STARD3
CHIR99021
70
55
35
Input
Elution
Input
@STARD3
@pS209 STARD3
GFP-VAP-A
GFP-VAP-A KD/MD
Flag-STARD3
CHIR99021
Input
Elution
Input
Elution
