# Supplementary figures and images for: STARD3 regulates lysosome positioning and contacts via a GSK3-controlled phosphorylation switch (part 7 of 7)
Source: EMBO J. 2026 Feb 25;45(7):2239–77. doi: 10.1038/s44318-026-00705-3 (PMC13044316; doi:10.1038/s44318-026-00705-3)

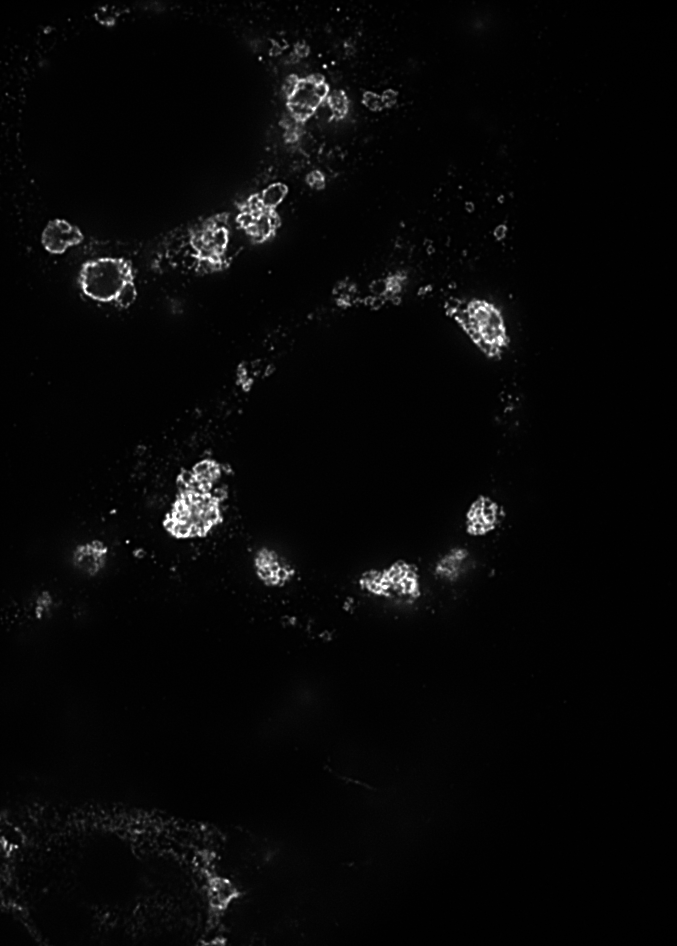

Supplement: Supplementary file 29 — Appendix Fig. S2-3 Source Data [file 44318_2026_705_MOESM29_ESM.zip › Appendix Figure S2-3/O/20241029_HeLaGFPVAPBg_STARD3S209Ar_NT_8_SR_w2SPI 561 mCherry.TIF]

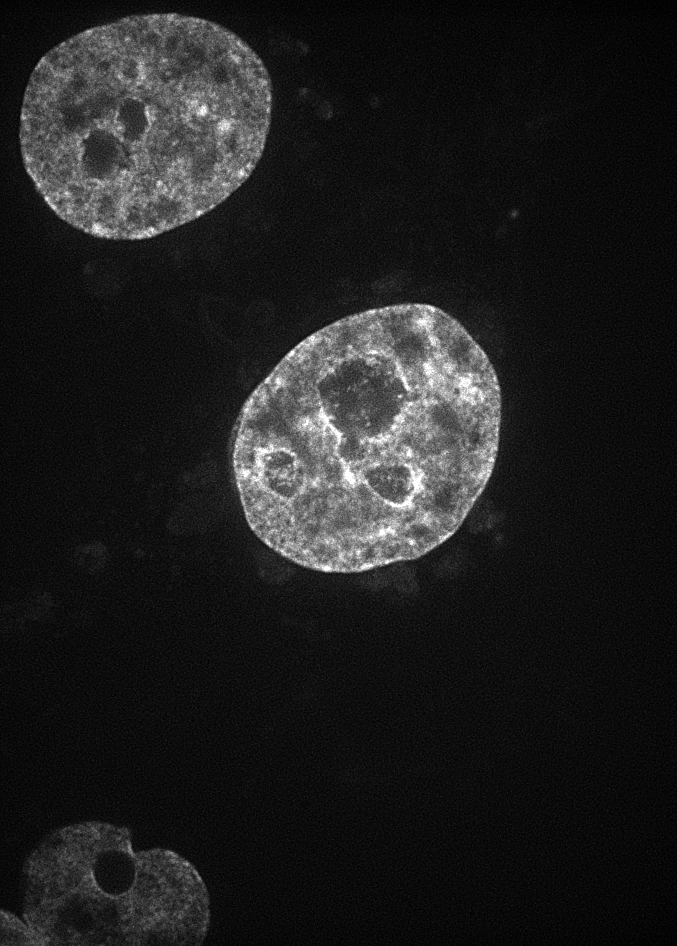

Supplement: Supplementary file 29 — Appendix Fig. S2-3 Source Data [file 44318_2026_705_MOESM29_ESM.zip › Appendix Figure S2-3/O/20241029_HeLaGFPVAPBg_STARD3S209Ar_NT_8_SR_w3SPI 405 DAPI.TIF]

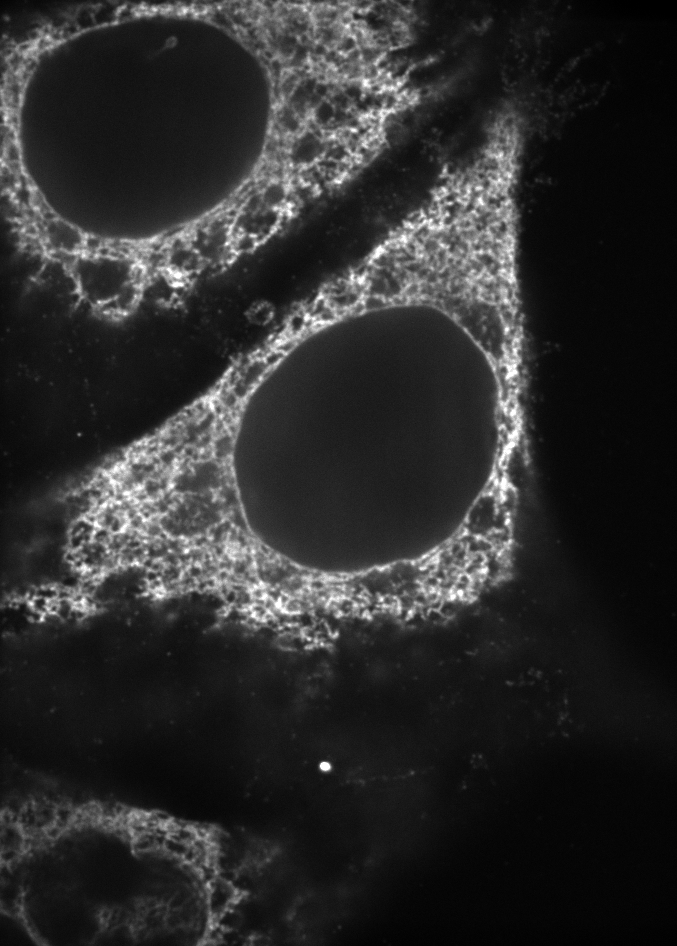

Supplement: Supplementary file 29 — Appendix Fig. S2-3 Source Data [file 44318_2026_705_MOESM29_ESM.zip › Appendix Figure S2-3/O/20241029_HeLaGFPVAPBg_STARD3S209Ar_NT_8_w1SPI 491 GFP.TIF]

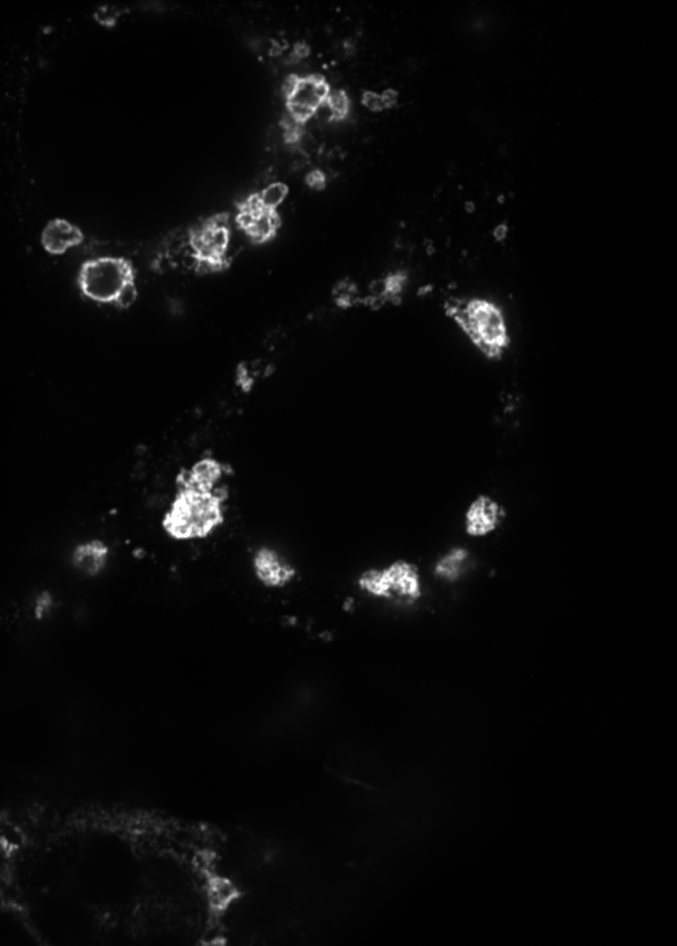

Supplement: Supplementary file 29 — Appendix Fig. S2-3 Source Data [file 44318_2026_705_MOESM29_ESM.zip › Appendix Figure S2-3/O/20241029_HeLaGFPVAPBg_STARD3S209Ar_NT_8_w2SPI 561 mCherry.TIF]

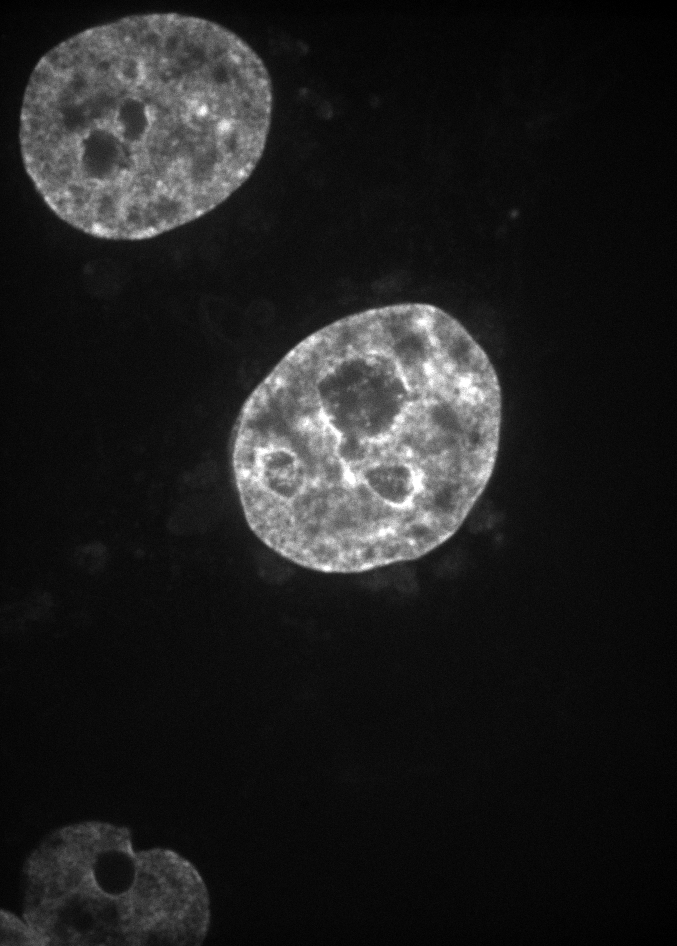

Supplement: Supplementary file 29 — Appendix Fig. S2-3 Source Data [file 44318_2026_705_MOESM29_ESM.zip › Appendix Figure S2-3/O/20241029_HeLaGFPVAPBg_STARD3S209Ar_NT_8_w3SPI 405 DAPI.TIF]

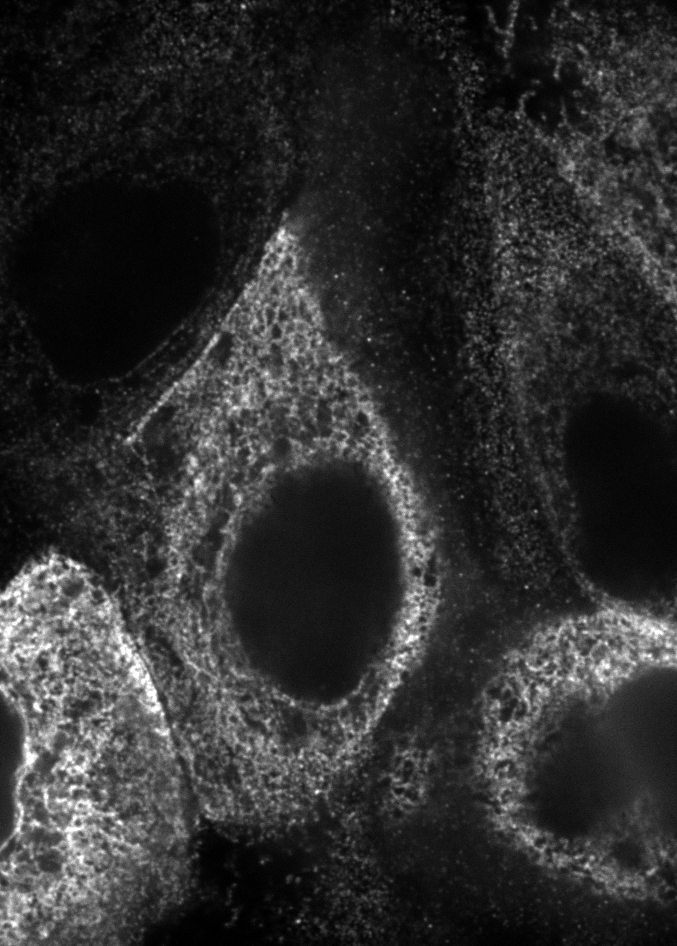

Supplement: Supplementary file 30 — Appendix Fig. S3 Source Data [file 44318_2026_705_MOESM30_ESM.zip › Appendix Figure S3/A/20250818_MCF7GFPVAPB_NT_4_w1SPI 491 GFP.TIF]

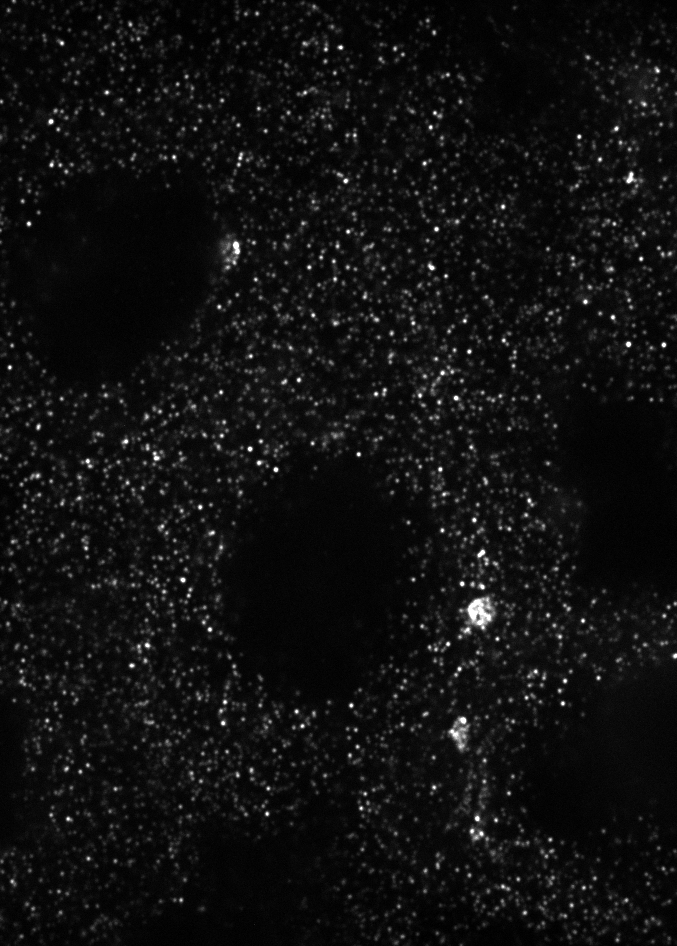

Supplement: Supplementary file 30 — Appendix Fig. S3 Source Data [file 44318_2026_705_MOESM30_ESM.zip › Appendix Figure S3/A/20250818_MCF7GFPVAPB_NT_4_w2SPI 561 mCherry.TIF]

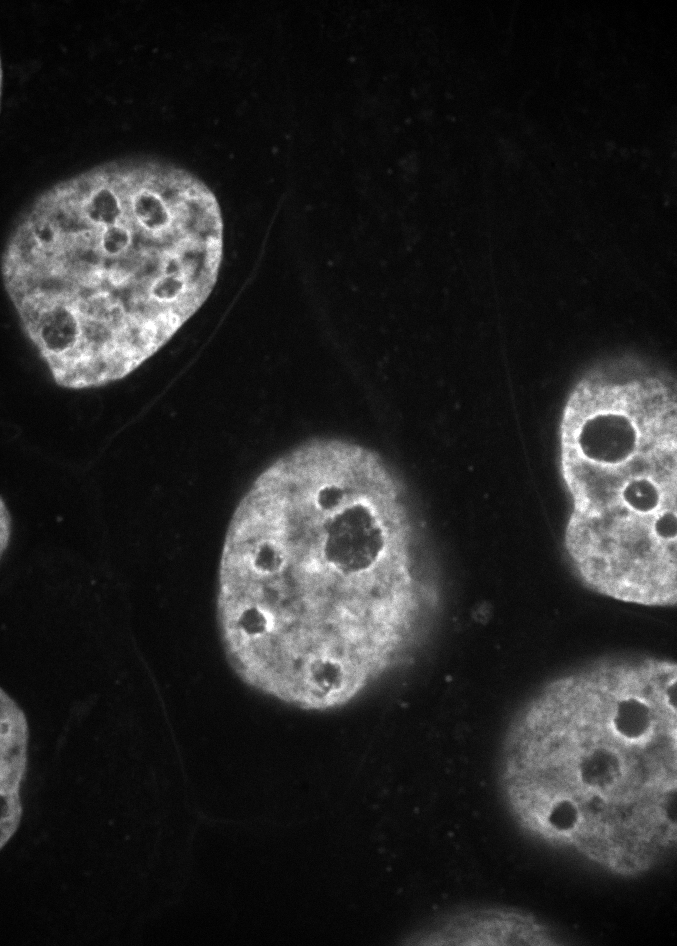

Supplement: Supplementary file 30 — Appendix Fig. S3 Source Data [file 44318_2026_705_MOESM30_ESM.zip › Appendix Figure S3/A/20250818_MCF7GFPVAPB_NT_4_w3SPI 405 DAPI.TIF]

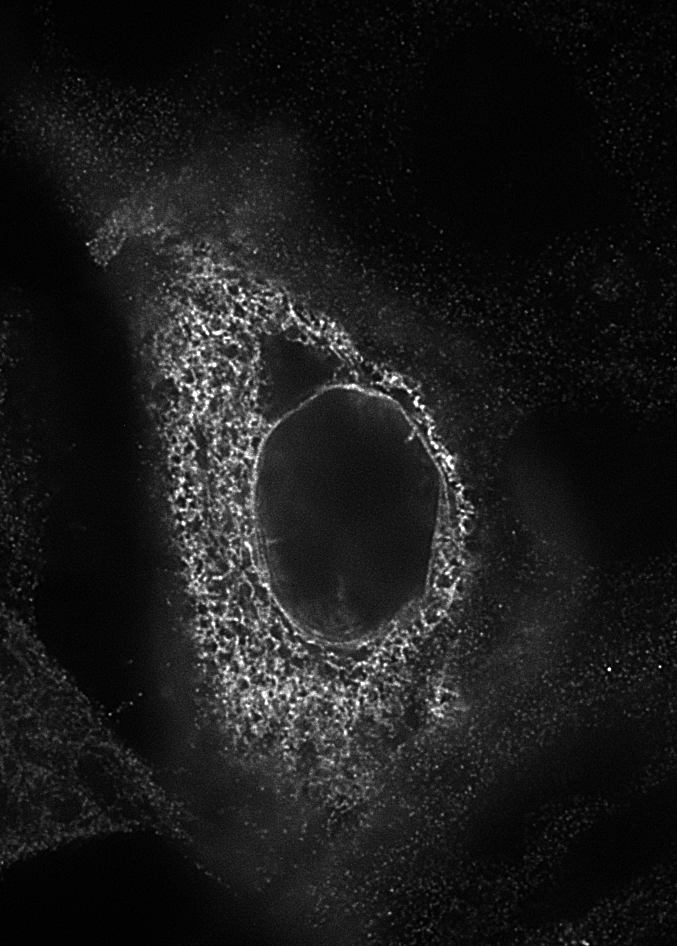

Supplement: Supplementary file 30 — Appendix Fig. S3 Source Data [file 44318_2026_705_MOESM30_ESM.zip › Appendix Figure S3/B/20250818_MCF7GFPVAPB_CHIR_1_SR_w1SPI 491 GFP.TIF]

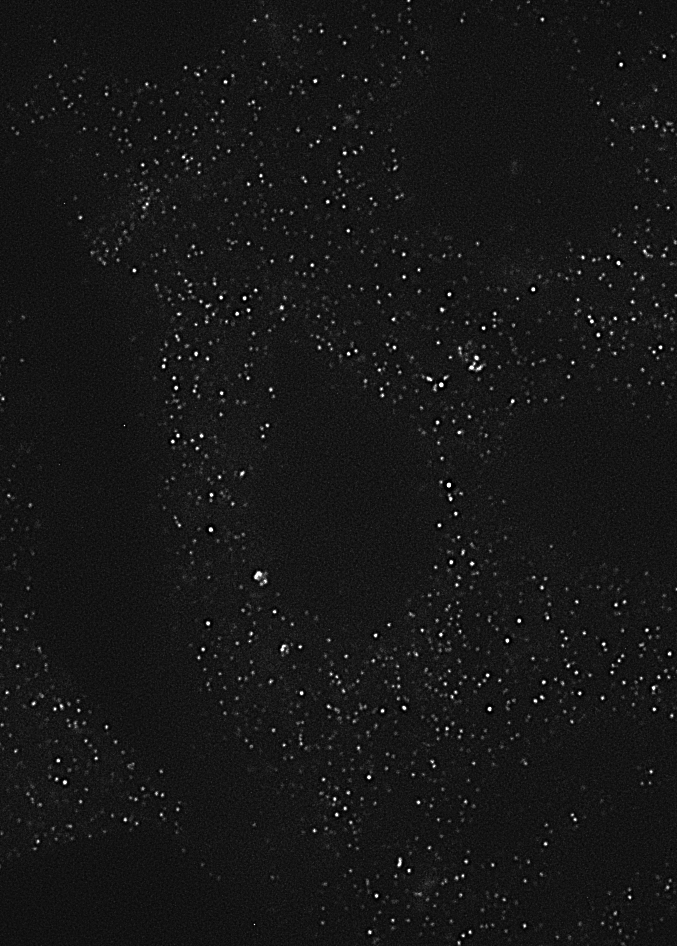

Supplement: Supplementary file 30 — Appendix Fig. S3 Source Data [file 44318_2026_705_MOESM30_ESM.zip › Appendix Figure S3/B/20250818_MCF7GFPVAPB_CHIR_1_SR_w2SPI 561 mCherry.TIF]

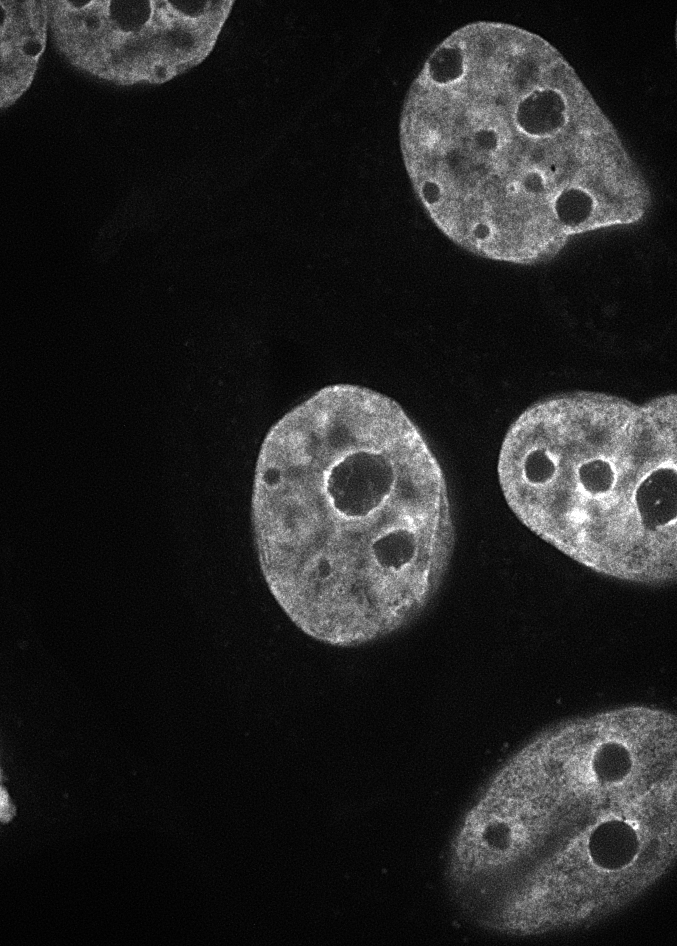

Supplement: Supplementary file 30 — Appendix Fig. S3 Source Data [file 44318_2026_705_MOESM30_ESM.zip › Appendix Figure S3/B/20250818_MCF7GFPVAPB_CHIR_1_SR_w3SPI 405 DAPI.TIF]

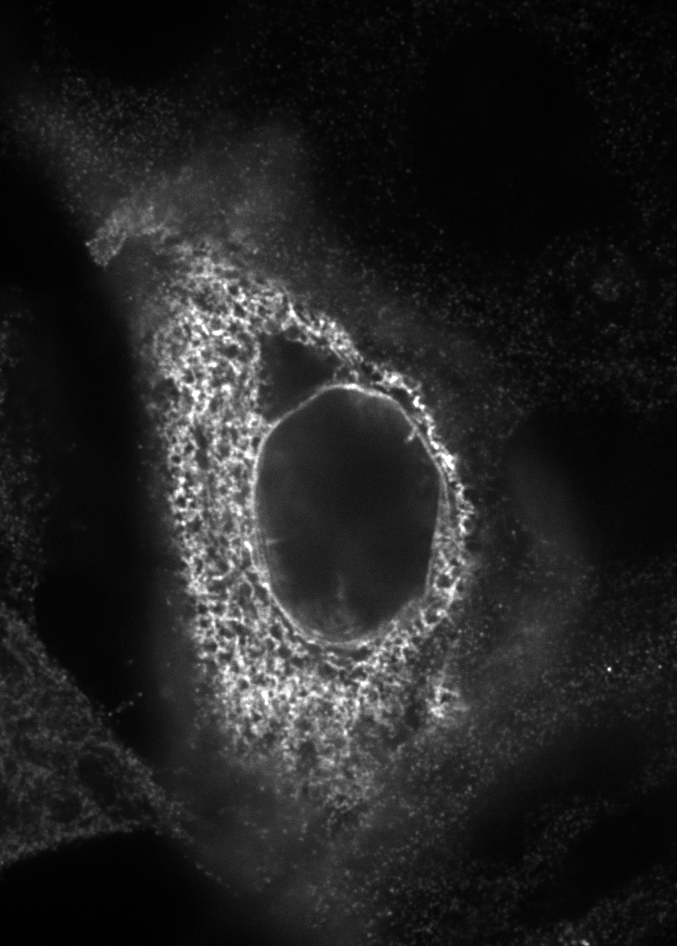

Supplement: Supplementary file 30 — Appendix Fig. S3 Source Data [file 44318_2026_705_MOESM30_ESM.zip › Appendix Figure S3/B/20250818_MCF7GFPVAPB_CHIR_1_w1SPI 491 GFP.TIF]

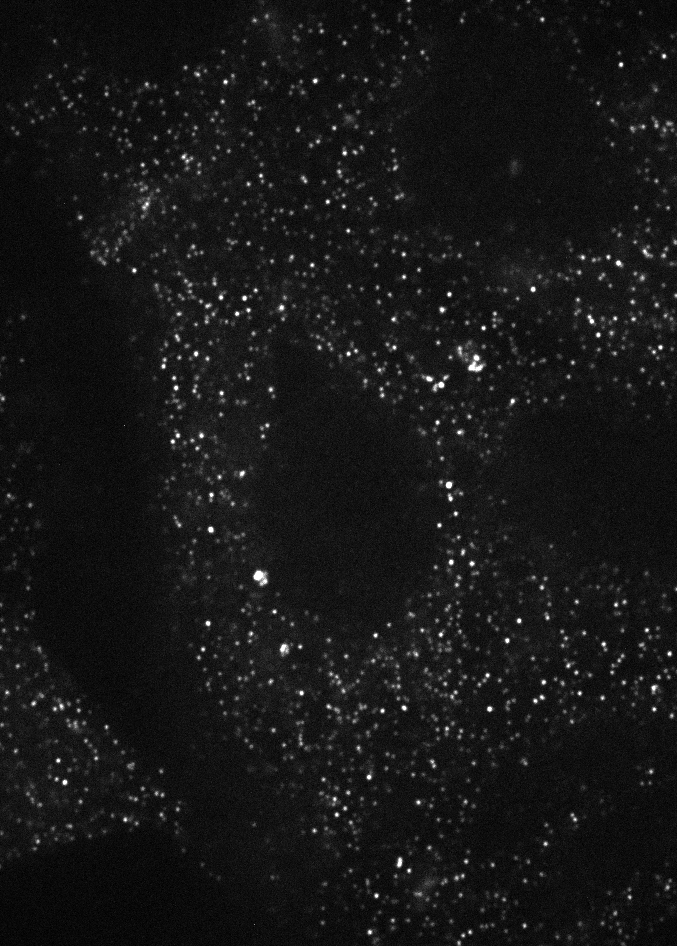

Supplement: Supplementary file 30 — Appendix Fig. S3 Source Data [file 44318_2026_705_MOESM30_ESM.zip › Appendix Figure S3/B/20250818_MCF7GFPVAPB_CHIR_1_w2SPI 561 mCherry.TIF]

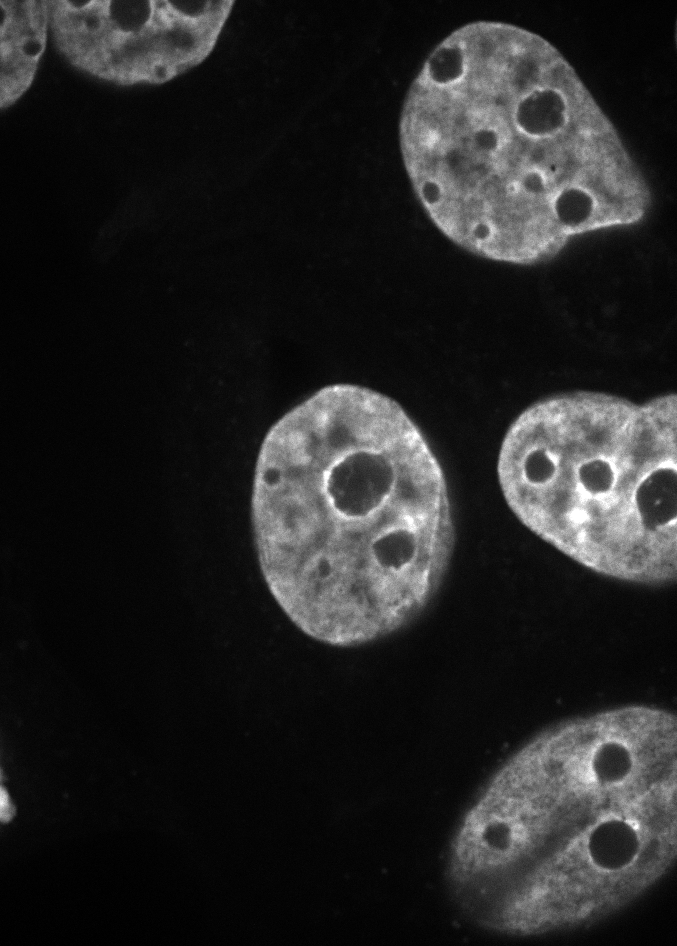

Supplement: Supplementary file 30 — Appendix Fig. S3 Source Data [file 44318_2026_705_MOESM30_ESM.zip › Appendix Figure S3/B/20250818_MCF7GFPVAPB_CHIR_1_w3SPI 405 DAPI.TIF]

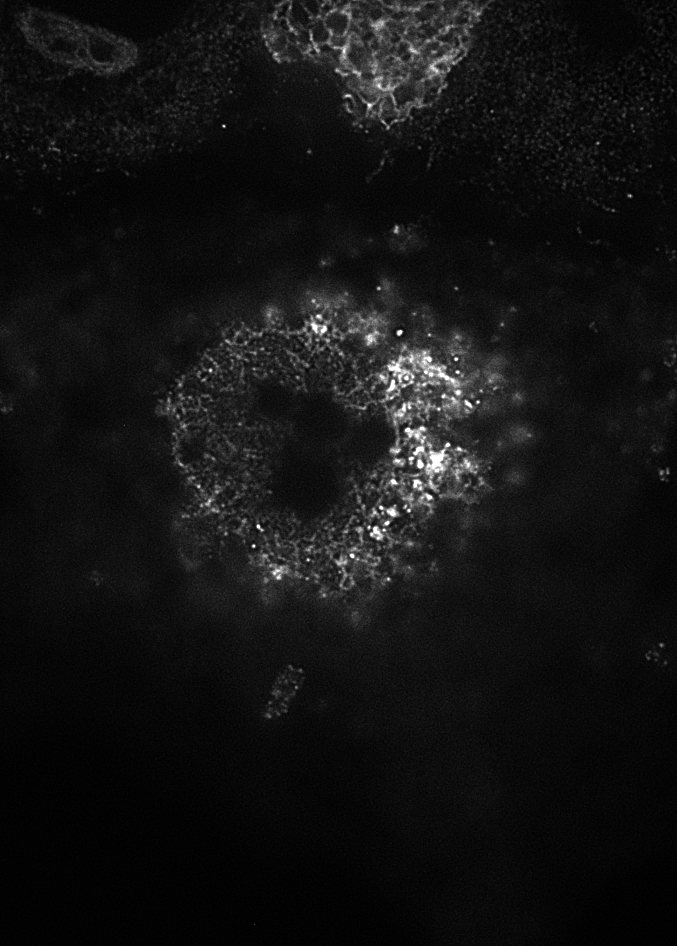

Supplement: Supplementary file 30 — Appendix Fig. S3 Source Data [file 44318_2026_705_MOESM30_ESM.zip › Appendix Figure S3/C/20250820_MCF7VAPBGFPSTARD3_NT_1_SR_w1SPI 491 GFP.TIF]

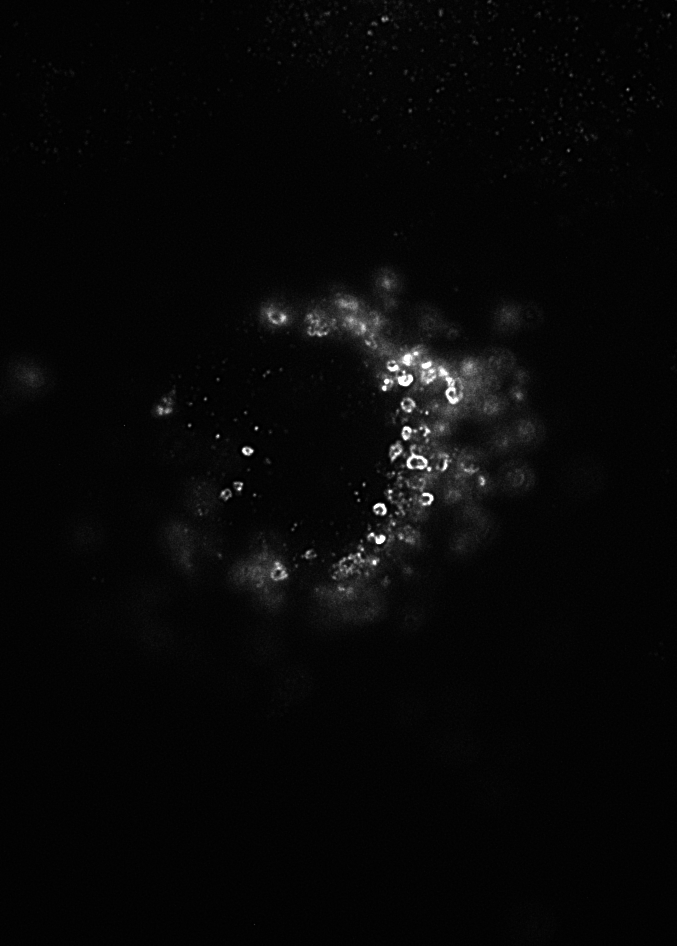

Supplement: Supplementary file 30 — Appendix Fig. S3 Source Data [file 44318_2026_705_MOESM30_ESM.zip › Appendix Figure S3/C/20250820_MCF7VAPBGFPSTARD3_NT_1_SR_w2SPI 561 mCherry.TIF]

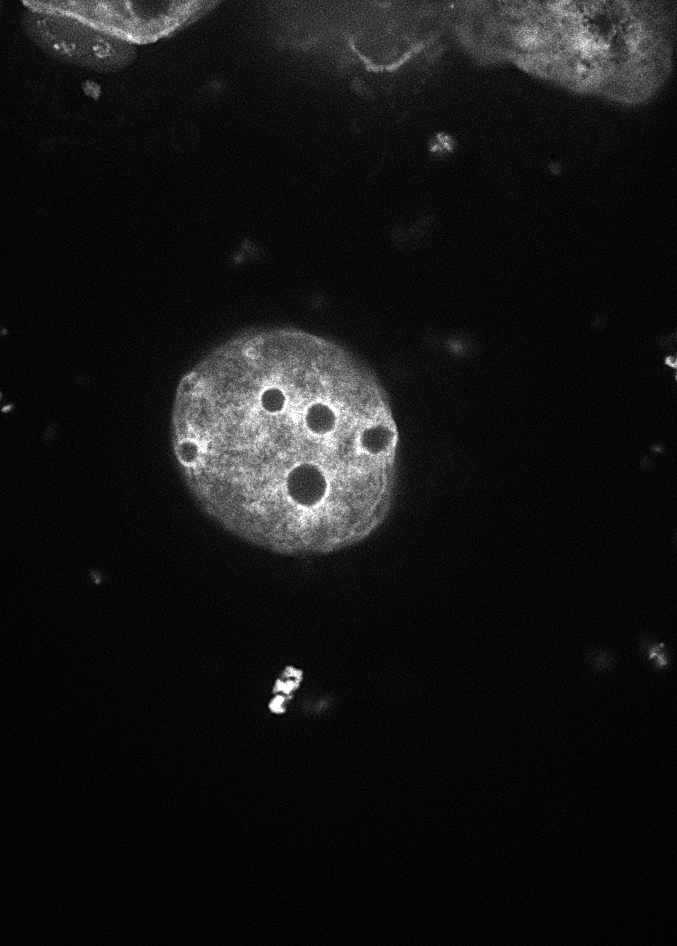

Supplement: Supplementary file 30 — Appendix Fig. S3 Source Data [file 44318_2026_705_MOESM30_ESM.zip › Appendix Figure S3/C/20250820_MCF7VAPBGFPSTARD3_NT_1_SR_w3SPI 405 DAPI.TIF]

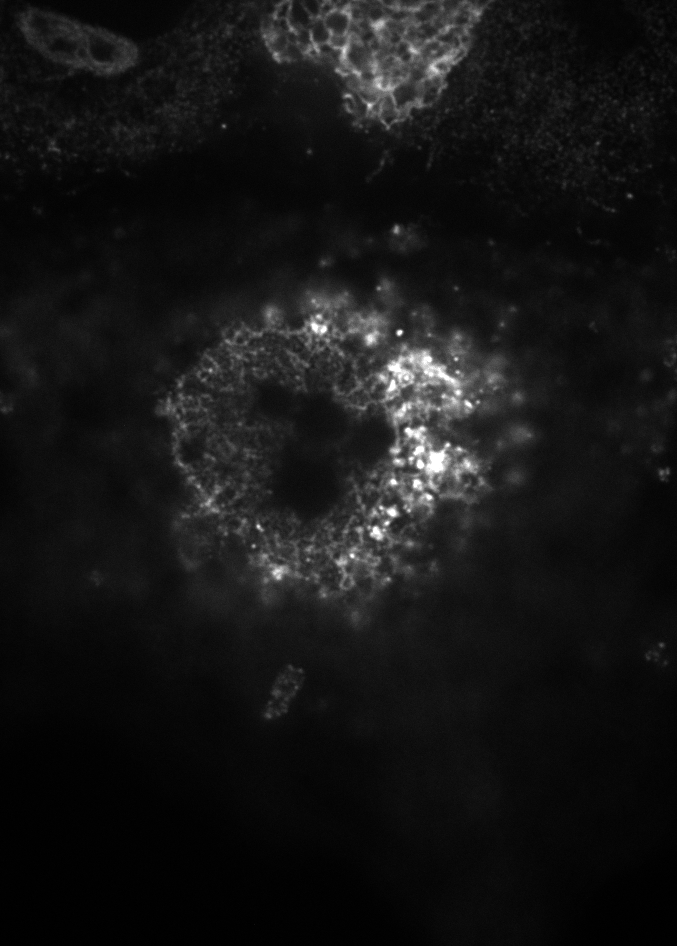

Supplement: Supplementary file 30 — Appendix Fig. S3 Source Data [file 44318_2026_705_MOESM30_ESM.zip › Appendix Figure S3/C/20250820_MCF7VAPBGFPSTARD3_NT_1_w1SPI 491 GFP.TIF]

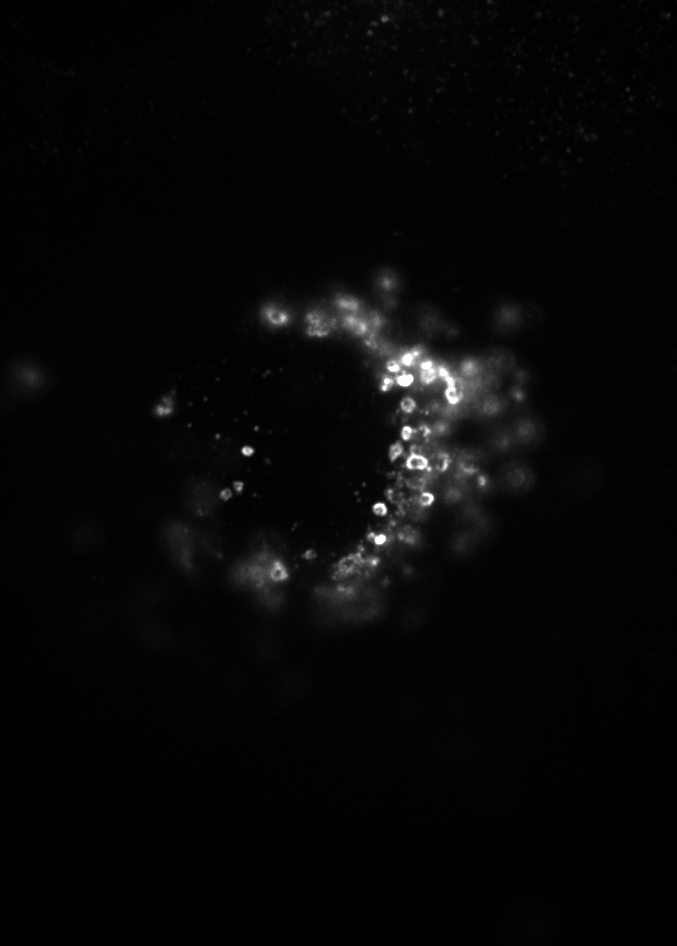

Supplement: Supplementary file 30 — Appendix Fig. S3 Source Data [file 44318_2026_705_MOESM30_ESM.zip › Appendix Figure S3/C/20250820_MCF7VAPBGFPSTARD3_NT_1_w2SPI 561 mCherry.TIF]

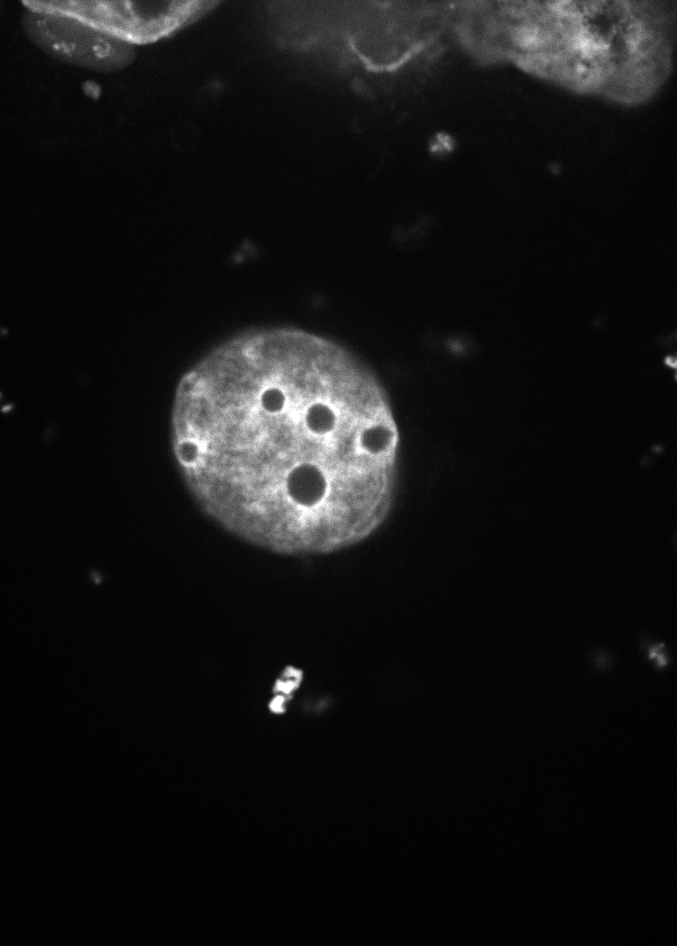

Supplement: Supplementary file 30 — Appendix Fig. S3 Source Data [file 44318_2026_705_MOESM30_ESM.zip › Appendix Figure S3/C/20250820_MCF7VAPBGFPSTARD3_NT_1_w3SPI 405 DAPI.TIF]

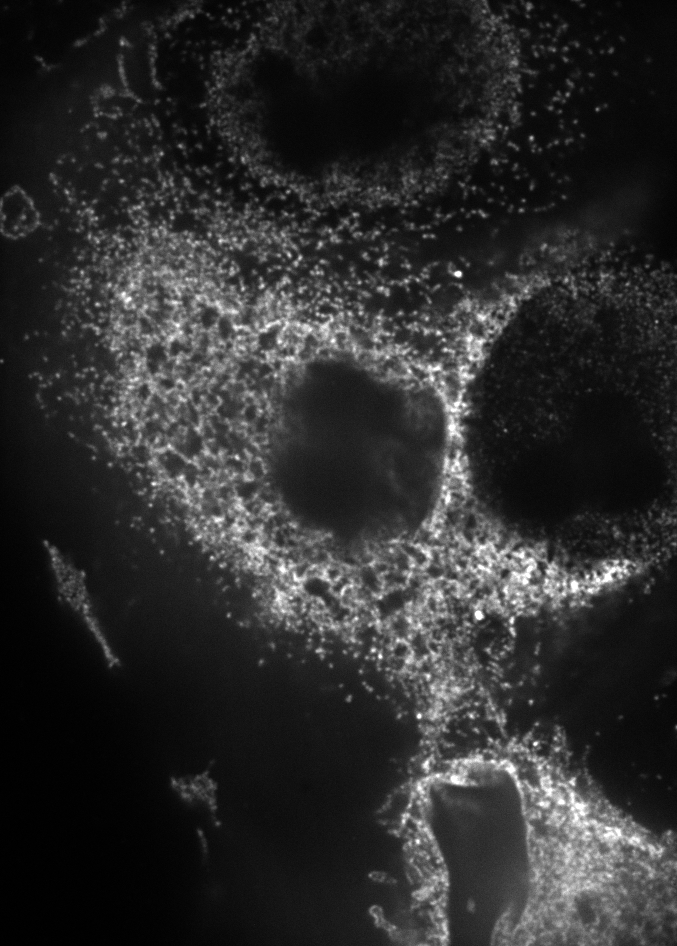

Supplement: Supplementary file 30 — Appendix Fig. S3 Source Data [file 44318_2026_705_MOESM30_ESM.zip › Appendix Figure S3/D/20250818_MCF7GFPVAPBSTARD3S209A_NT_1_w1SPI 491 GFP.TIF]

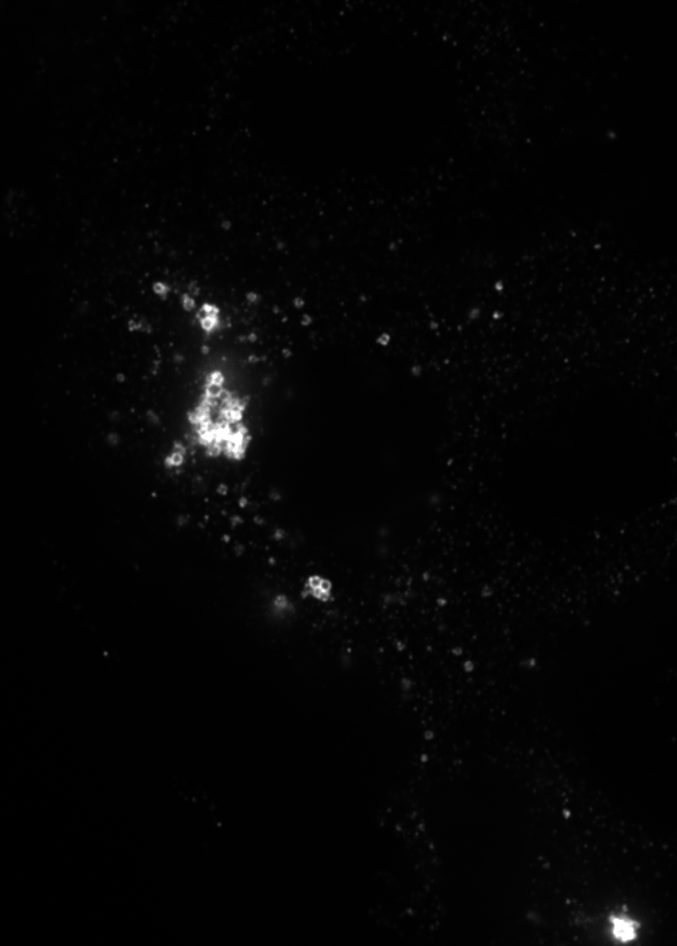

Supplement: Supplementary file 30 — Appendix Fig. S3 Source Data [file 44318_2026_705_MOESM30_ESM.zip › Appendix Figure S3/D/20250818_MCF7GFPVAPBSTARD3S209A_NT_1_w2SPI 561 mCherry.TIF]

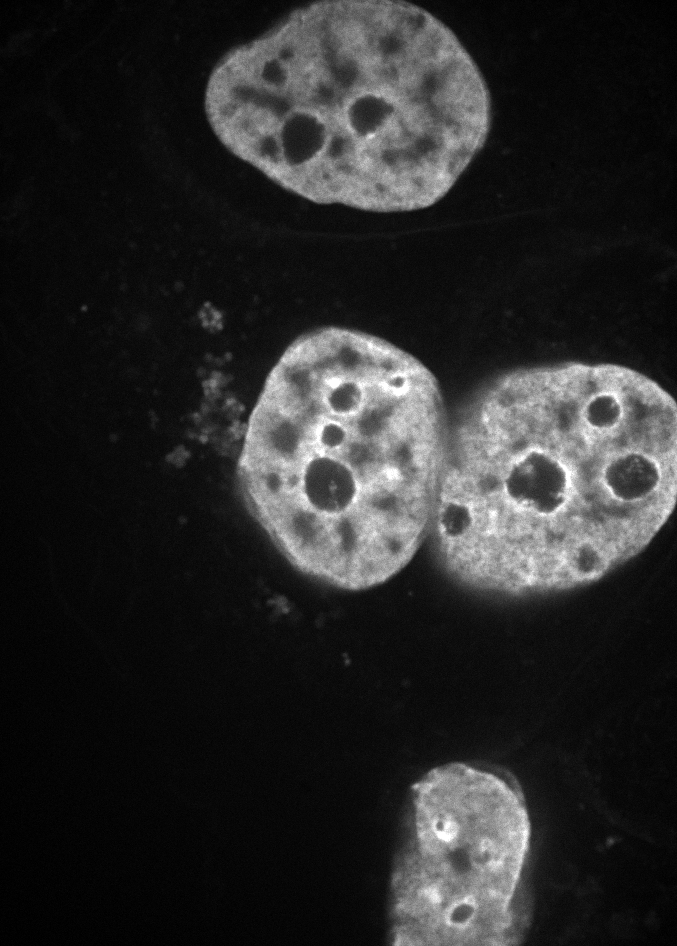

Supplement: Supplementary file 30 — Appendix Fig. S3 Source Data [file 44318_2026_705_MOESM30_ESM.zip › Appendix Figure S3/D/20250818_MCF7GFPVAPBSTARD3S209A_NT_1_w3SPI 405 DAPI.TIF]

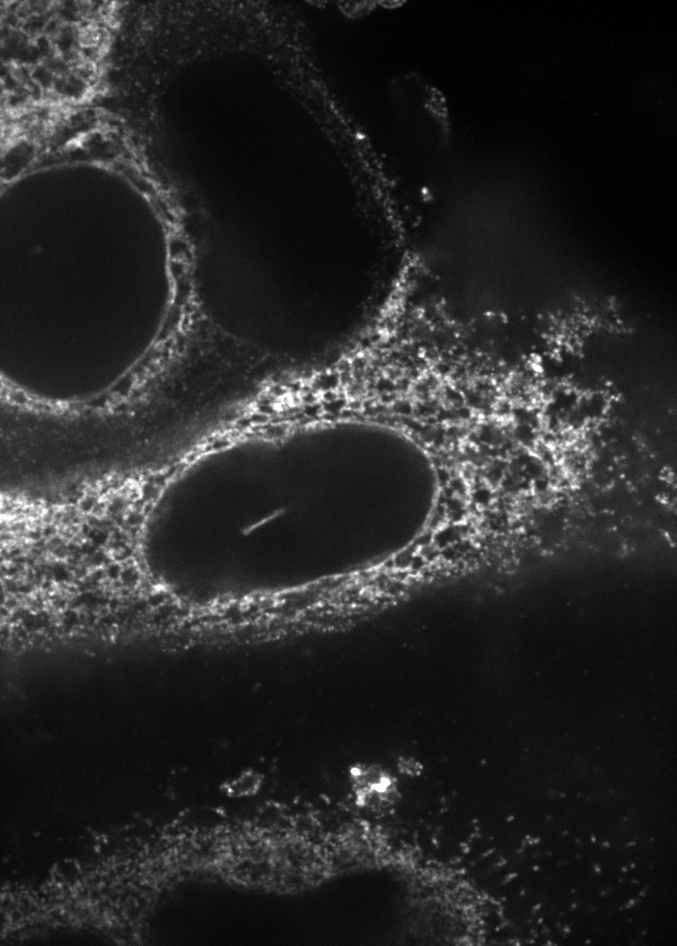

Supplement: Supplementary file 30 — Appendix Fig. S3 Source Data [file 44318_2026_705_MOESM30_ESM.zip › Appendix Figure S3/E/20250818_MCF7GFPVAPBSTARD3_CHIR_2_w1SPI 491 GFP.TIF]

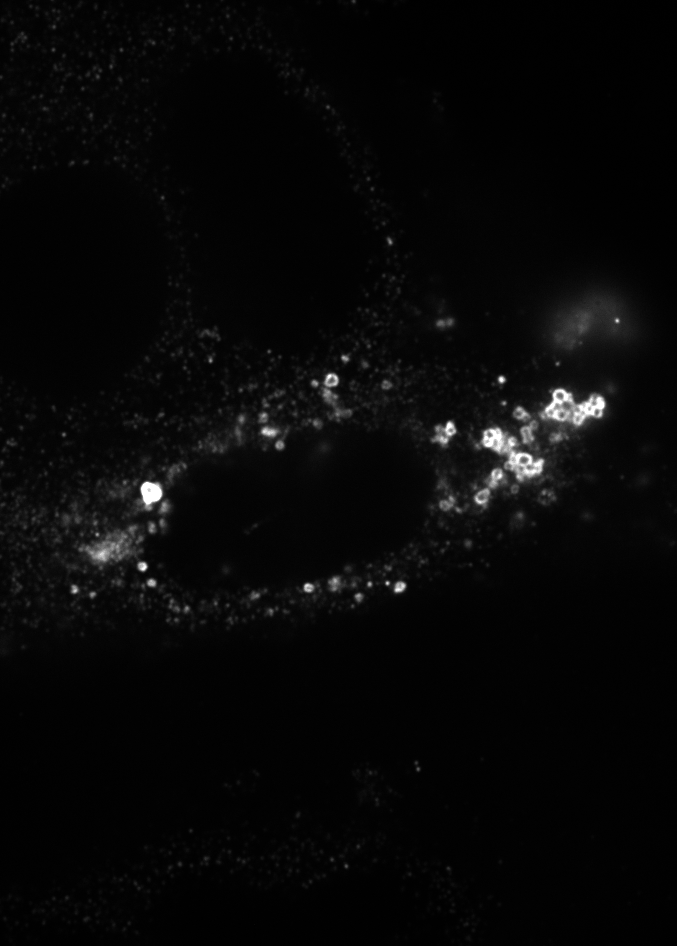

Supplement: Supplementary file 30 — Appendix Fig. S3 Source Data [file 44318_2026_705_MOESM30_ESM.zip › Appendix Figure S3/E/20250818_MCF7GFPVAPBSTARD3_CHIR_2_w2SPI 561 mCherry.TIF]

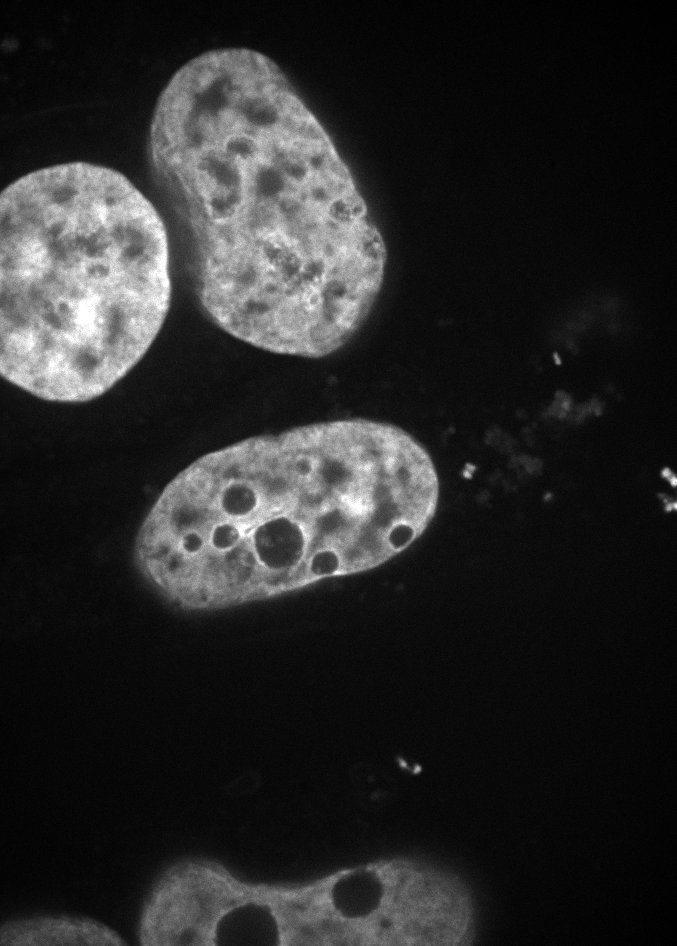

Supplement: Supplementary file 30 — Appendix Fig. S3 Source Data [file 44318_2026_705_MOESM30_ESM.zip › Appendix Figure S3/E/20250818_MCF7GFPVAPBSTARD3_CHIR_2_w3SPI 405 DAPI.TIF]

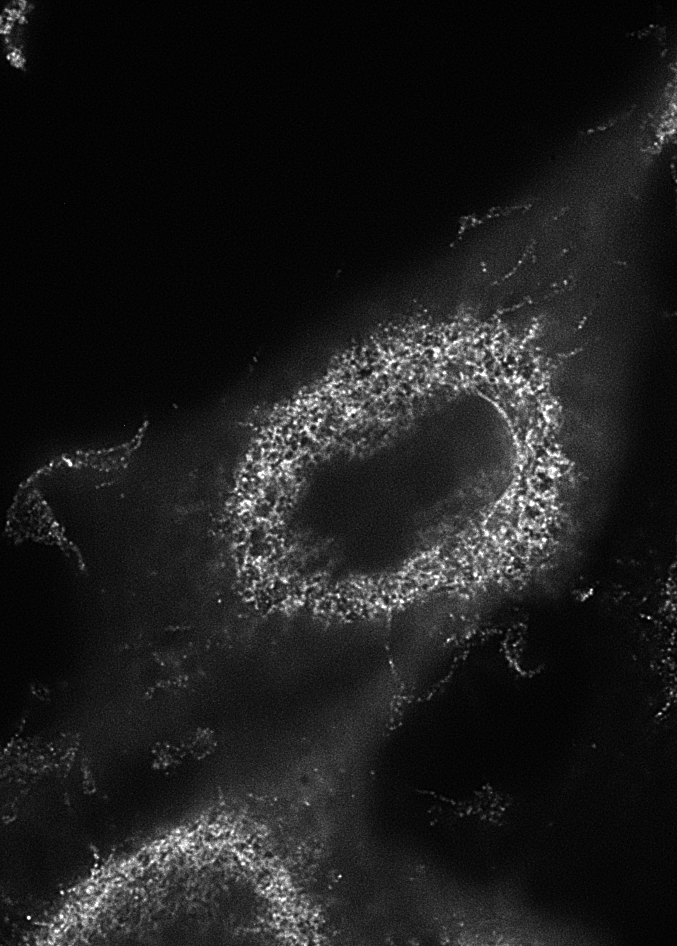

Supplement: Supplementary file 30 — Appendix Fig. S3 Source Data [file 44318_2026_705_MOESM30_ESM.zip › Appendix Figure S3/G/20250821_MCF7VAPAGFP_NT_3_SR_w1SPI 491 GFP.TIF]

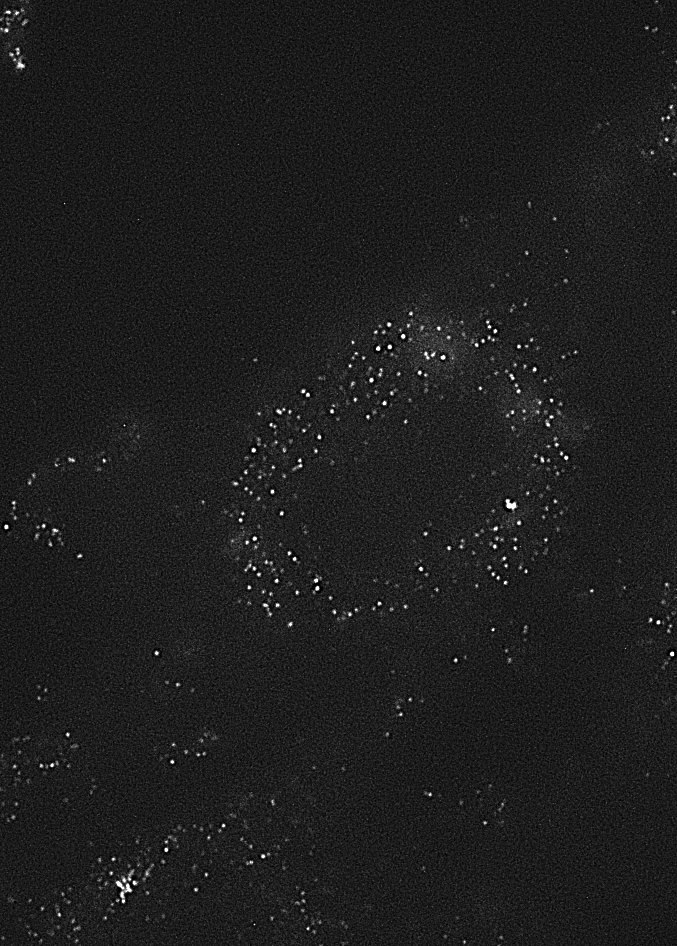

Supplement: Supplementary file 30 — Appendix Fig. S3 Source Data [file 44318_2026_705_MOESM30_ESM.zip › Appendix Figure S3/G/20250821_MCF7VAPAGFP_NT_3_SR_w2SPI 561 mCherry.TIF]

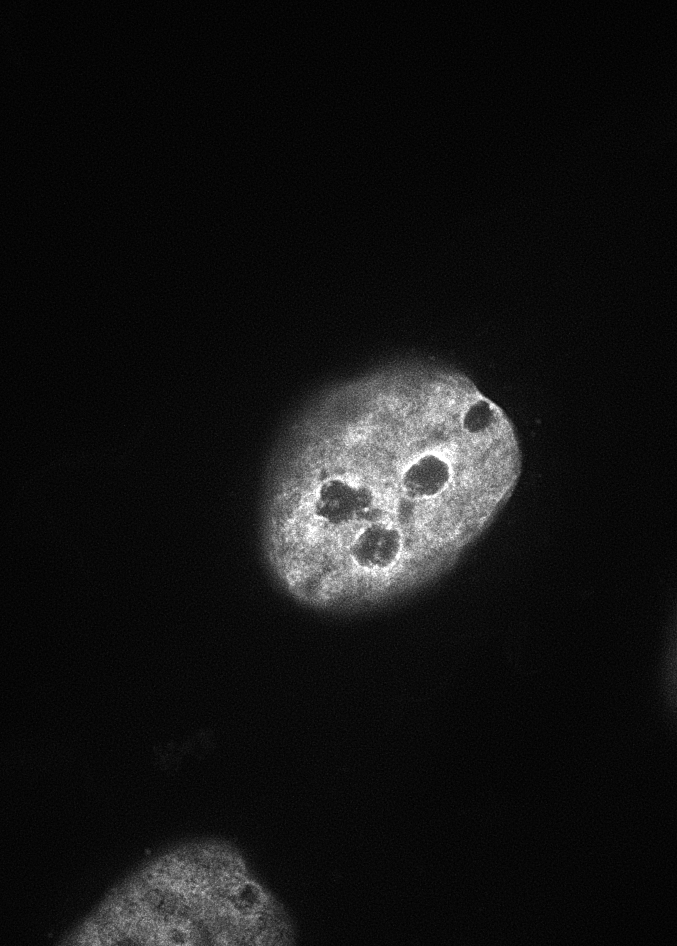

Supplement: Supplementary file 30 — Appendix Fig. S3 Source Data [file 44318_2026_705_MOESM30_ESM.zip › Appendix Figure S3/G/20250821_MCF7VAPAGFP_NT_3_SR_w3SPI 405 DAPI.TIF]

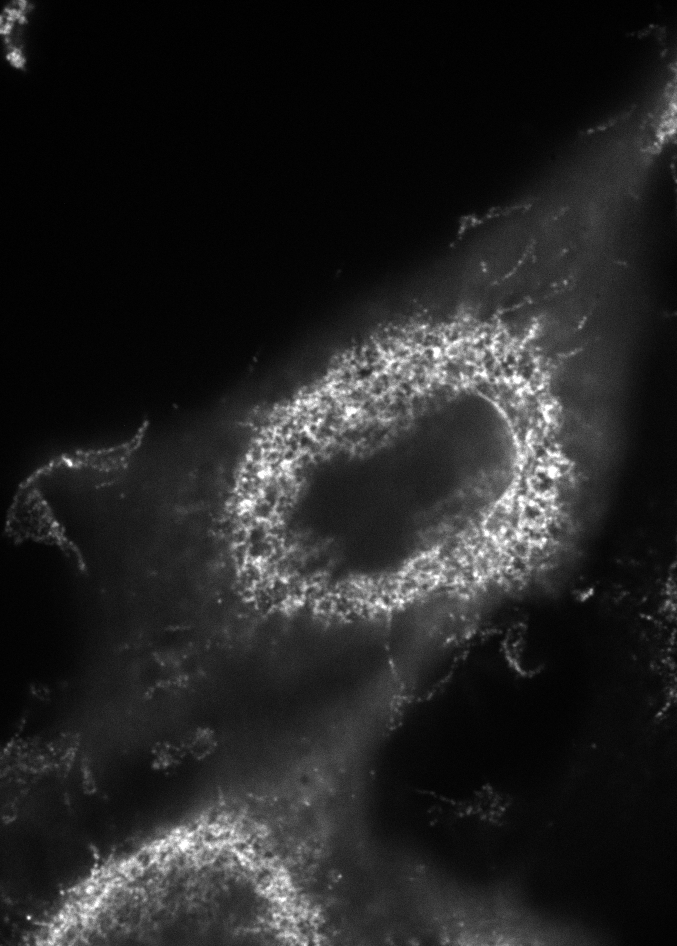

Supplement: Supplementary file 30 — Appendix Fig. S3 Source Data [file 44318_2026_705_MOESM30_ESM.zip › Appendix Figure S3/G/20250821_MCF7VAPAGFP_NT_3_w1SPI 491 GFP.TIF]

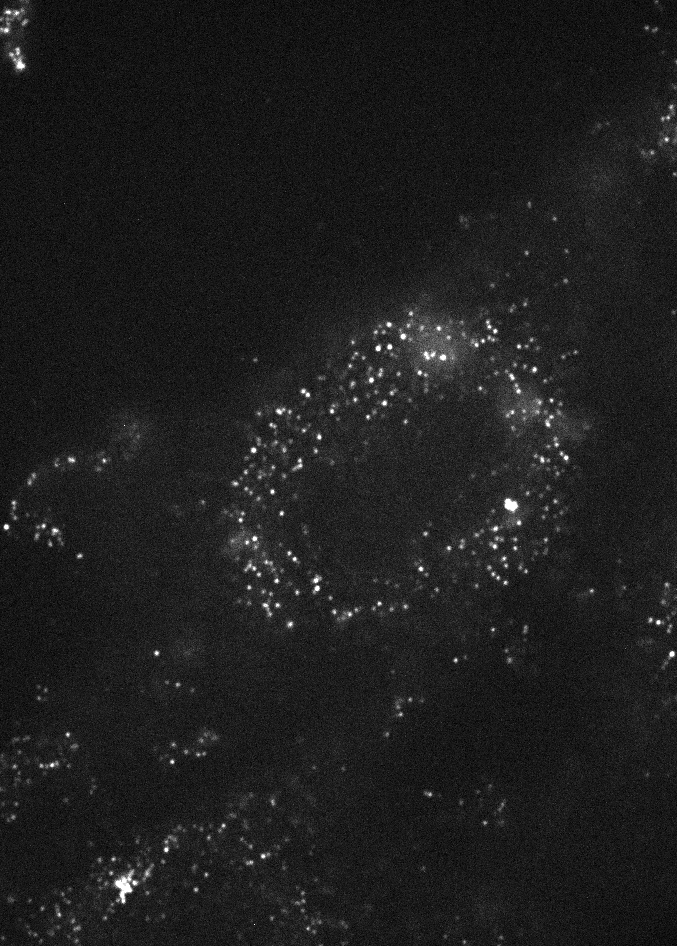

Supplement: Supplementary file 30 — Appendix Fig. S3 Source Data [file 44318_2026_705_MOESM30_ESM.zip › Appendix Figure S3/G/20250821_MCF7VAPAGFP_NT_3_w2SPI 561 mCherry.TIF]

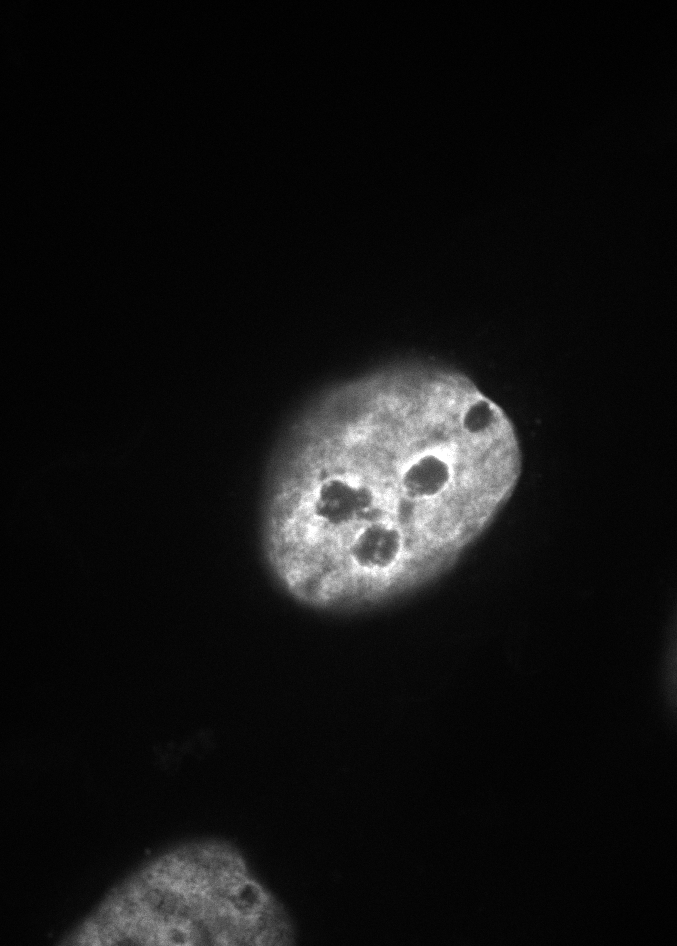

Supplement: Supplementary file 30 — Appendix Fig. S3 Source Data [file 44318_2026_705_MOESM30_ESM.zip › Appendix Figure S3/G/20250821_MCF7VAPAGFP_NT_3_w3SPI 405 DAPI.TIF]

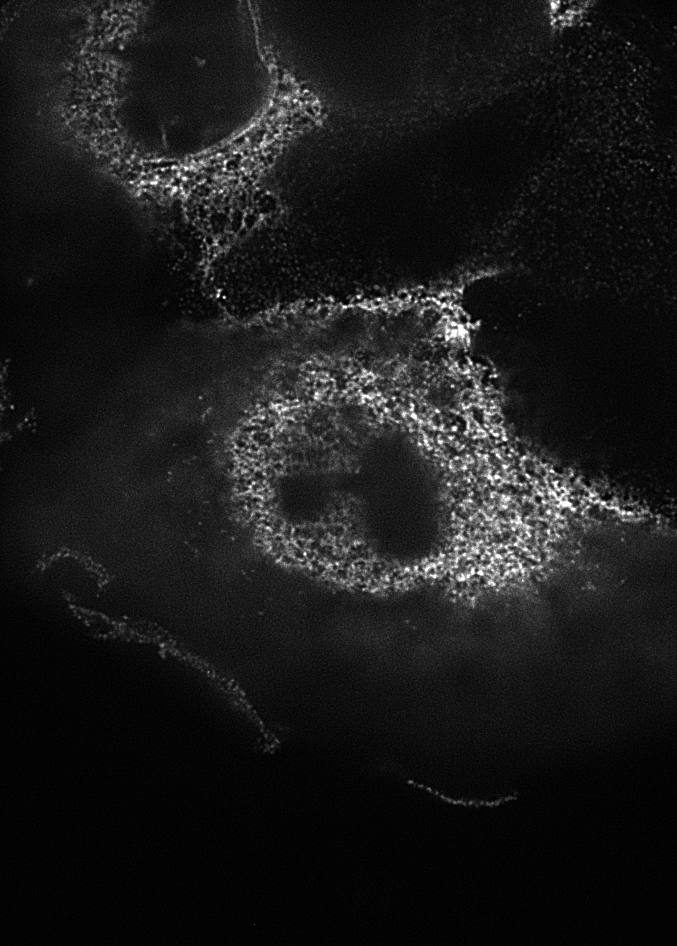

Supplement: Supplementary file 30 — Appendix Fig. S3 Source Data [file 44318_2026_705_MOESM30_ESM.zip › Appendix Figure S3/H/20250821_MCF7VAPAGFP_CHIR_3_SR_w1SPI 491 GFP.TIF]

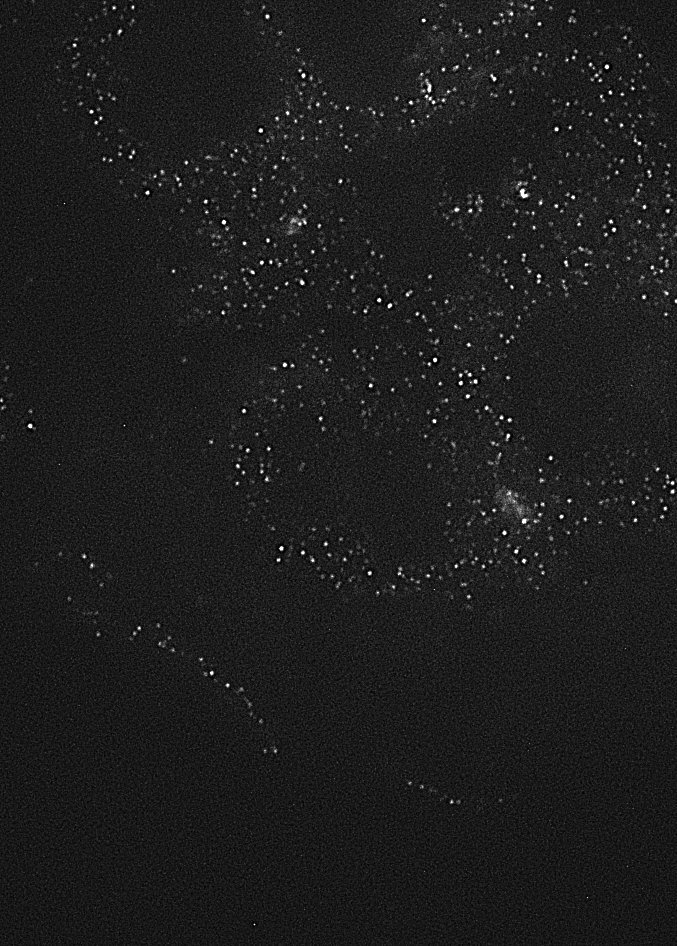

Supplement: Supplementary file 30 — Appendix Fig. S3 Source Data [file 44318_2026_705_MOESM30_ESM.zip › Appendix Figure S3/H/20250821_MCF7VAPAGFP_CHIR_3_SR_w2SPI 561 mCherry.TIF]

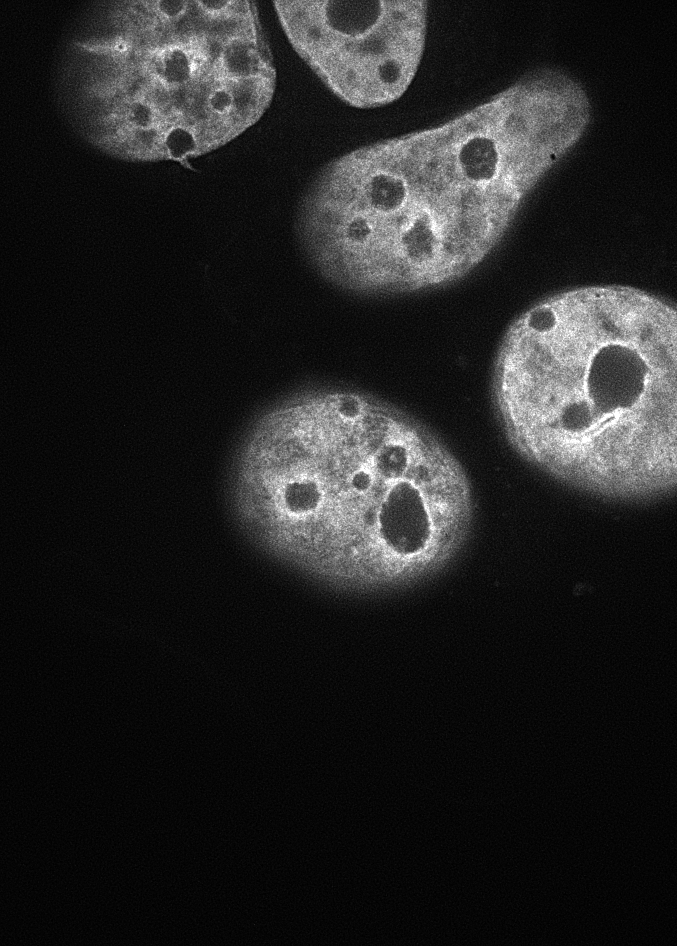

Supplement: Supplementary file 30 — Appendix Fig. S3 Source Data [file 44318_2026_705_MOESM30_ESM.zip › Appendix Figure S3/H/20250821_MCF7VAPAGFP_CHIR_3_SR_w3SPI 405 DAPI.TIF]

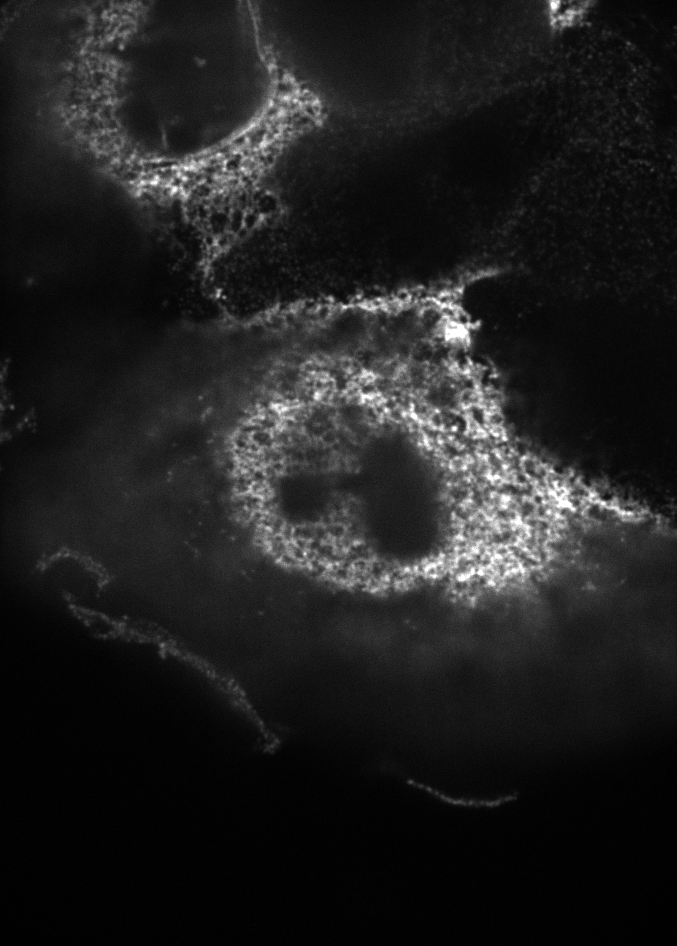

Supplement: Supplementary file 30 — Appendix Fig. S3 Source Data [file 44318_2026_705_MOESM30_ESM.zip › Appendix Figure S3/H/20250821_MCF7VAPAGFP_CHIR_3_w1SPI 491 GFP.TIF]

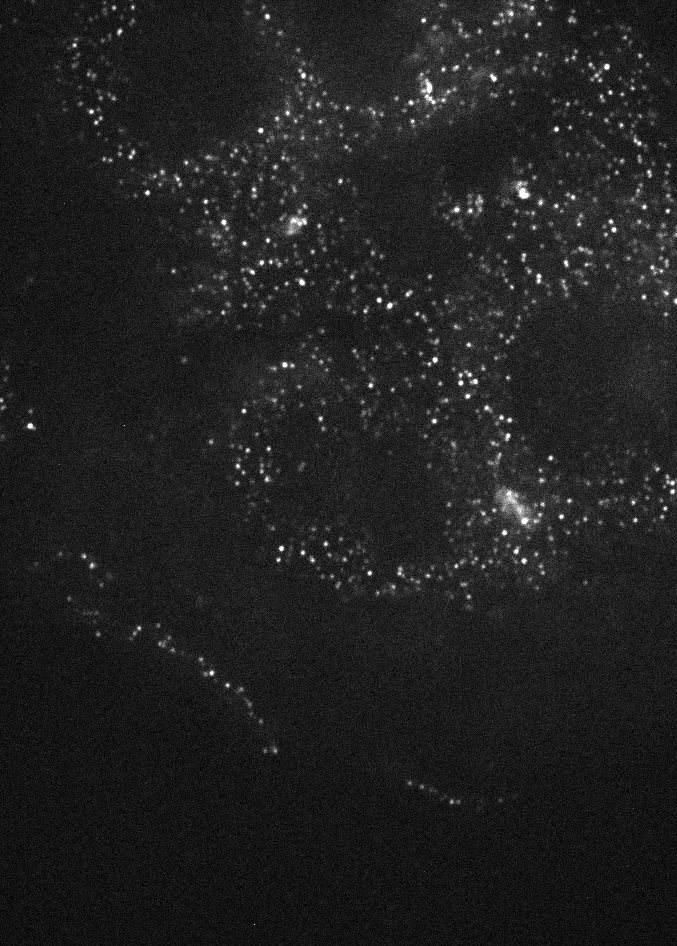

Supplement: Supplementary file 30 — Appendix Fig. S3 Source Data [file 44318_2026_705_MOESM30_ESM.zip › Appendix Figure S3/H/20250821_MCF7VAPAGFP_CHIR_3_w2SPI 561 mCherry.TIF]

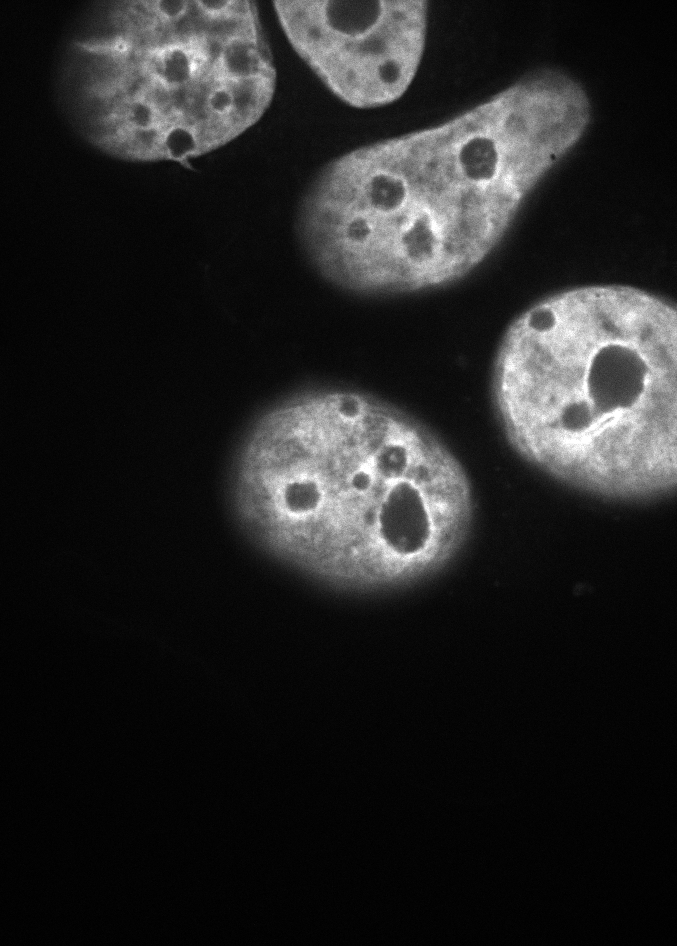

Supplement: Supplementary file 30 — Appendix Fig. S3 Source Data [file 44318_2026_705_MOESM30_ESM.zip › Appendix Figure S3/H/20250821_MCF7VAPAGFP_CHIR_3_w3SPI 405 DAPI.TIF]

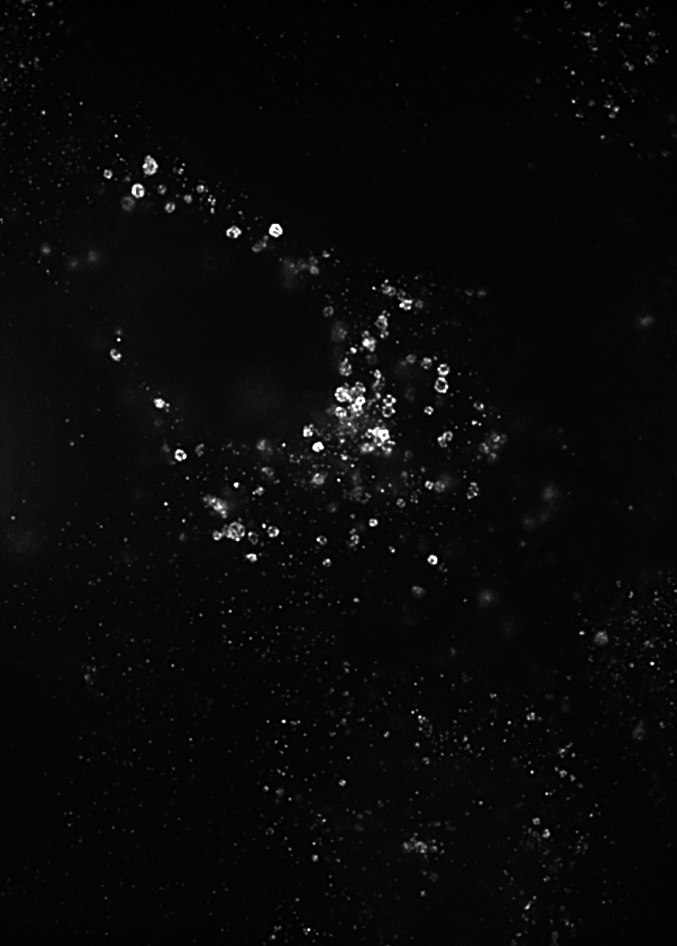

Supplement: Supplementary file 31 — Appendix Fig. S4 Source Data [file 44318_2026_705_MOESM31_ESM.zip › Appendix Figure S4/20230210_MCF7STARD3WT_NT_6_SR_w1SPI 491 GFP.TIF]

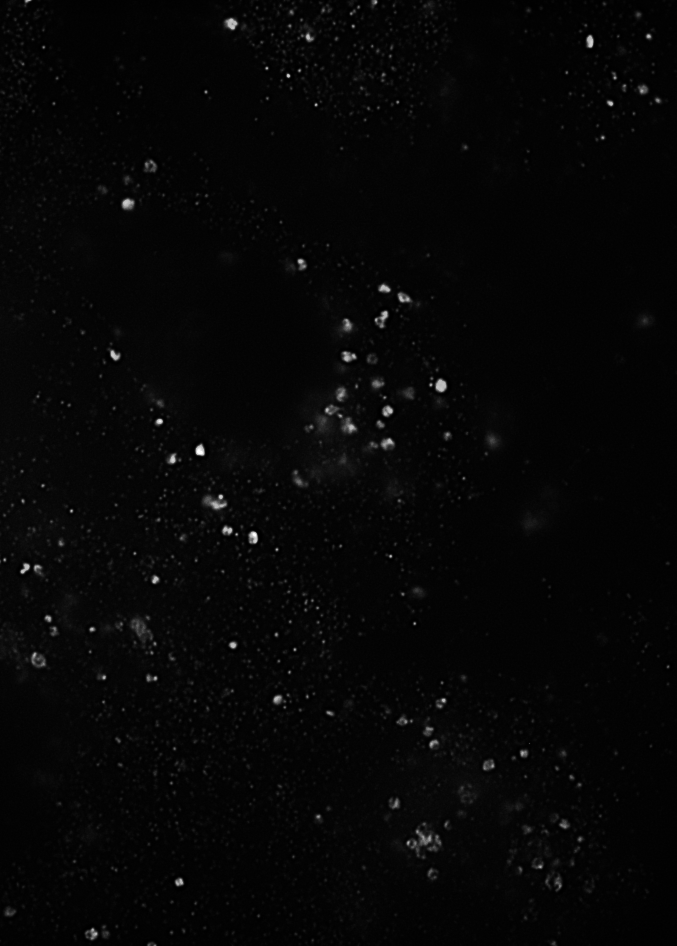

Supplement: Supplementary file 31 — Appendix Fig. S4 Source Data [file 44318_2026_705_MOESM31_ESM.zip › Appendix Figure S4/20230210_MCF7STARD3WT_NT_6_SR_w2SPI 561 mCherry.TIF]

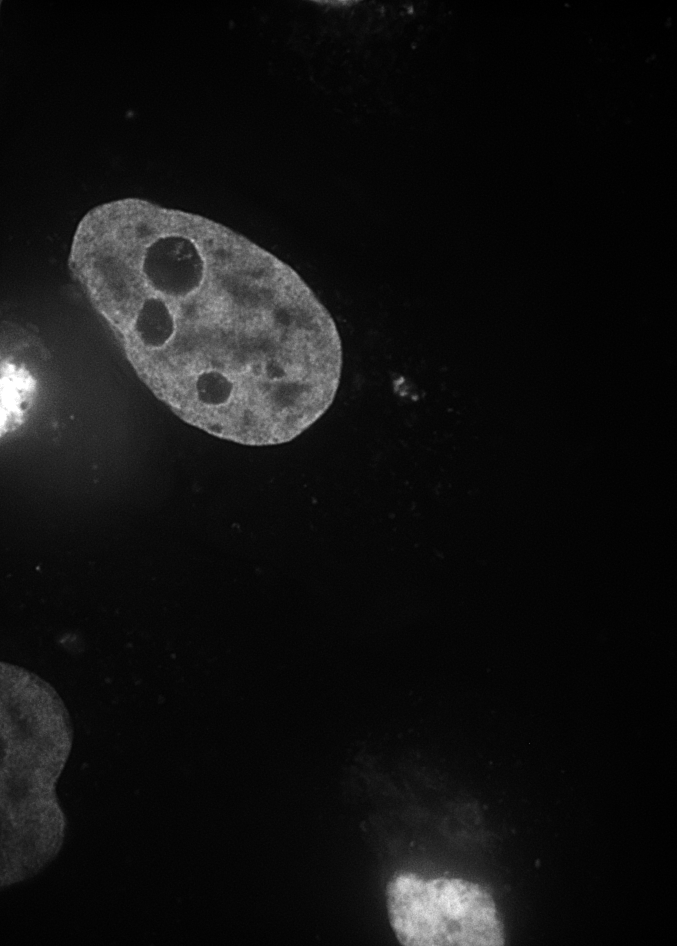

Supplement: Supplementary file 31 — Appendix Fig. S4 Source Data [file 44318_2026_705_MOESM31_ESM.zip › Appendix Figure S4/20230210_MCF7STARD3WT_NT_6_SR_w3SPI 405 DAPI.TIF]

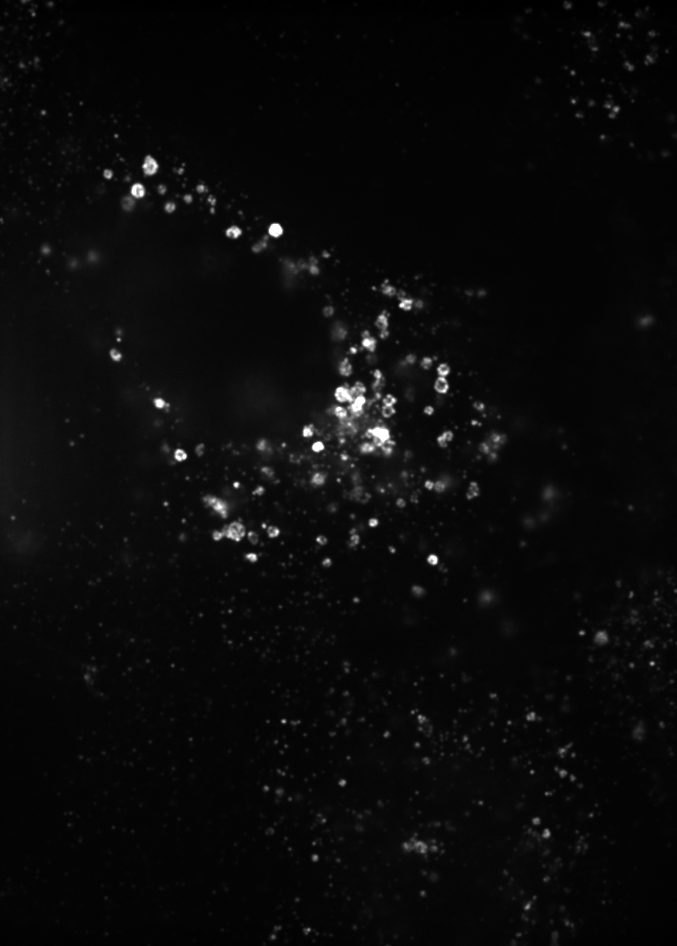

Supplement: Supplementary file 31 — Appendix Fig. S4 Source Data [file 44318_2026_705_MOESM31_ESM.zip › Appendix Figure S4/20230210_MCF7STARD3WT_NT_6_w1SPI 491 GFP.TIF]

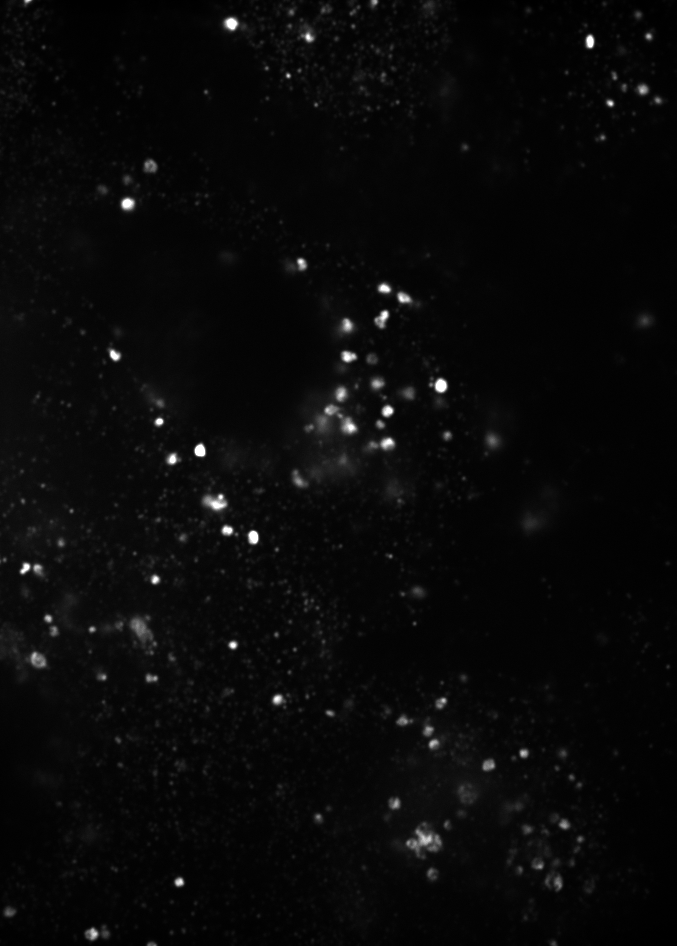

Supplement: Supplementary file 31 — Appendix Fig. S4 Source Data [file 44318_2026_705_MOESM31_ESM.zip › Appendix Figure S4/20230210_MCF7STARD3WT_NT_6_w2SPI 561 mCherry.TIF]

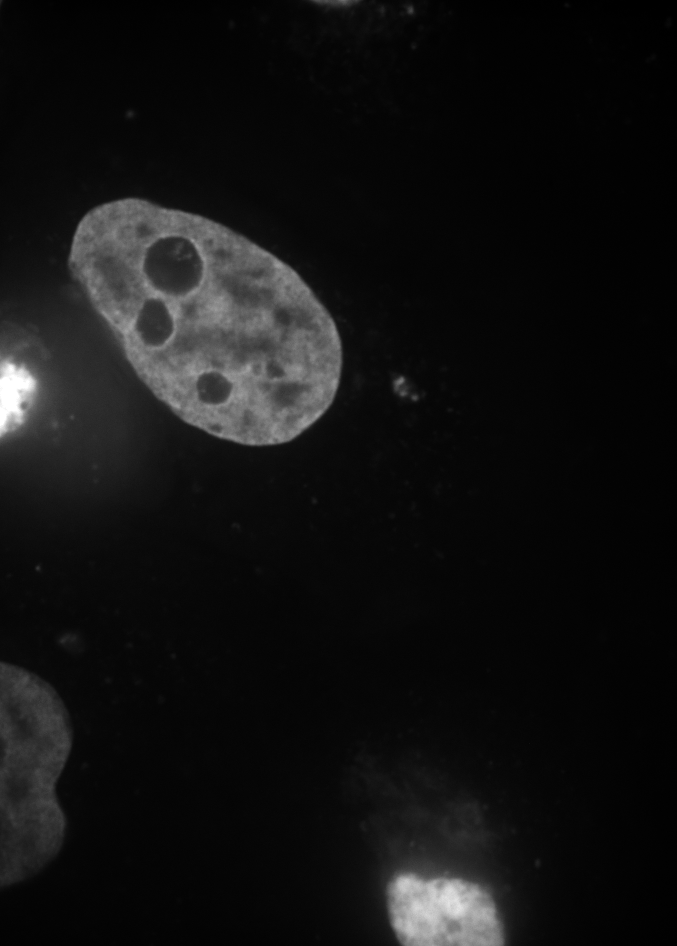

Supplement: Supplementary file 31 — Appendix Fig. S4 Source Data [file 44318_2026_705_MOESM31_ESM.zip › Appendix Figure S4/20230210_MCF7STARD3WT_NT_6_w3SPI 405 DAPI.TIF]

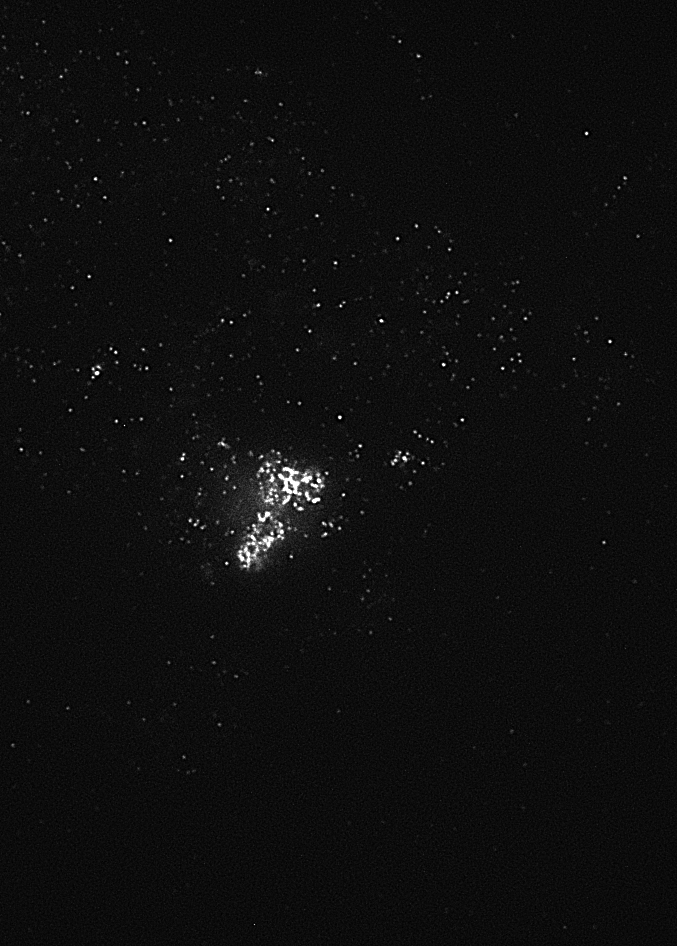

Supplement: Supplementary file 33 — Appendix Fig. S6 Source Data [file 44318_2026_705_MOESM33_ESM.zip › Appendix Figure S6/A/STARD3WT_CHIR/20250210_STARD3WT605_CHIR_8_SR_w1SPI 491 GFP.TIF]

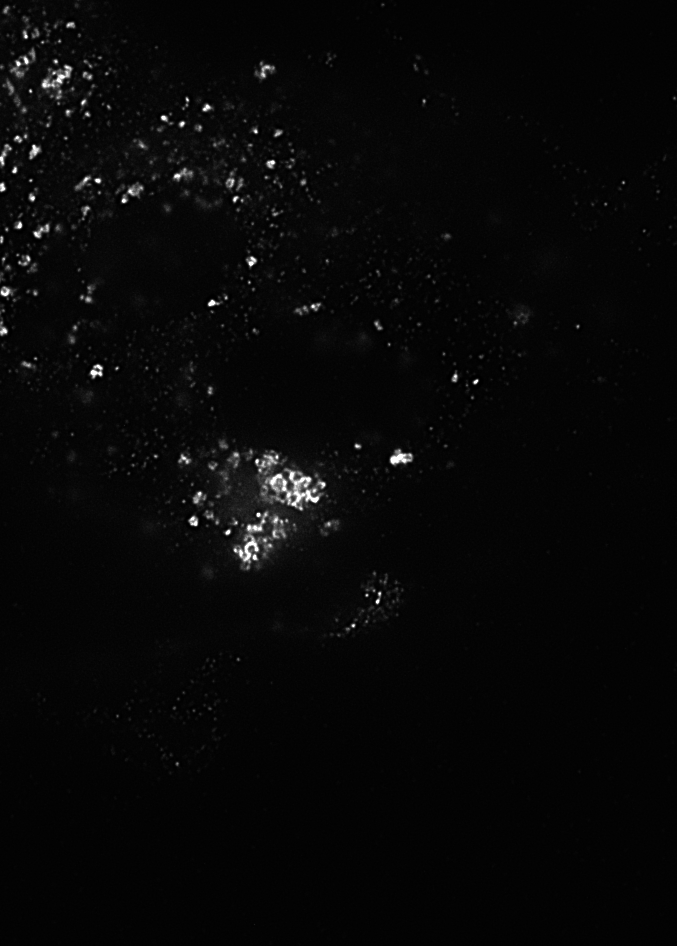

Supplement: Supplementary file 33 — Appendix Fig. S6 Source Data [file 44318_2026_705_MOESM33_ESM.zip › Appendix Figure S6/A/STARD3WT_CHIR/20250210_STARD3WT605_CHIR_8_SR_w2SPI 561 mCherry.TIF]

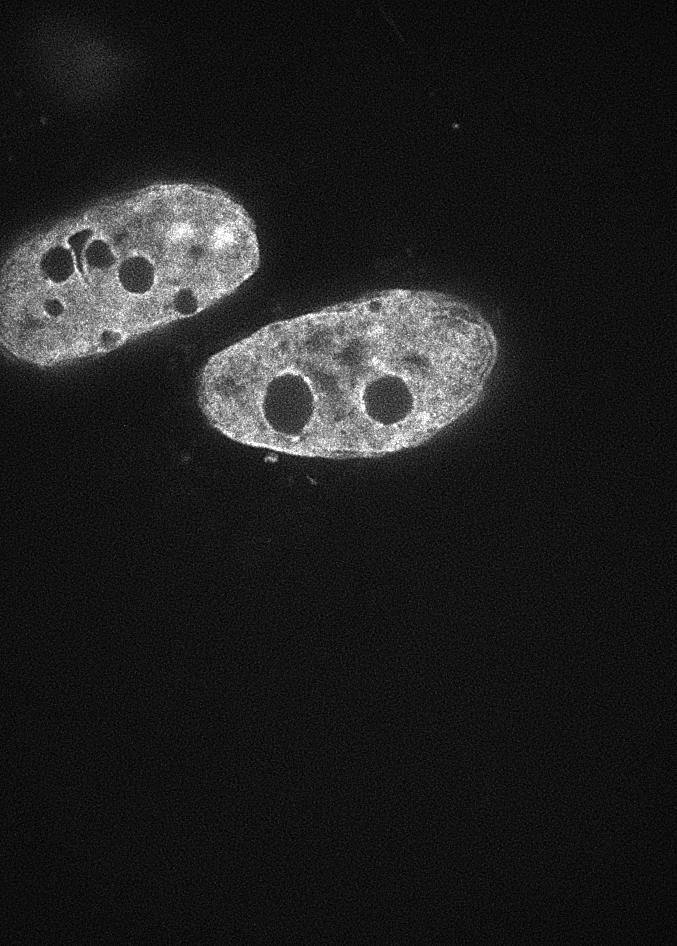

Supplement: Supplementary file 33 — Appendix Fig. S6 Source Data [file 44318_2026_705_MOESM33_ESM.zip › Appendix Figure S6/A/STARD3WT_CHIR/20250210_STARD3WT605_CHIR_8_SR_w3SPI 405 DAPI.TIF]

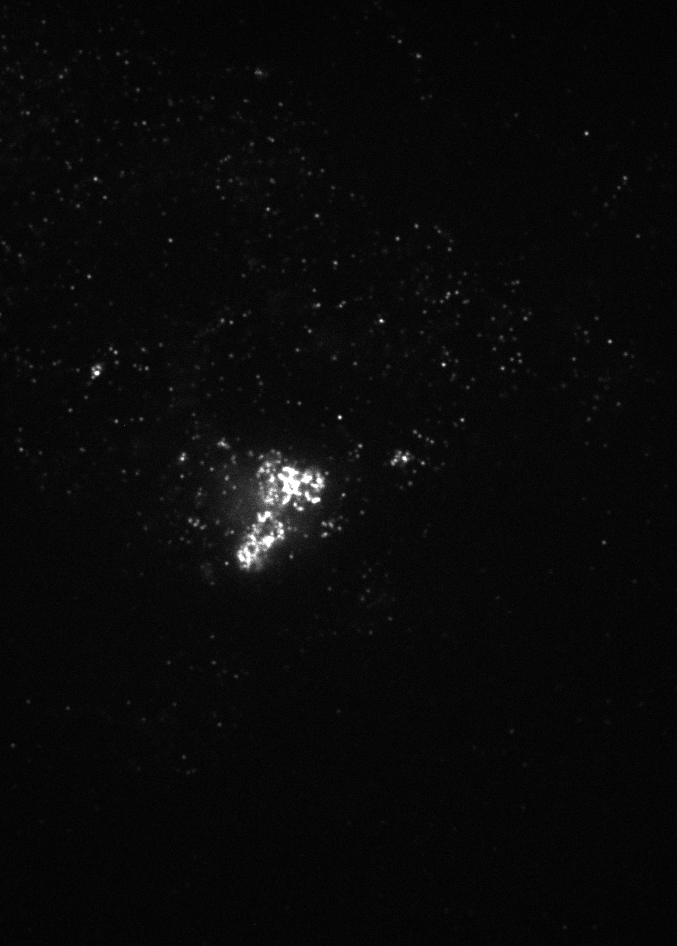

Supplement: Supplementary file 33 — Appendix Fig. S6 Source Data [file 44318_2026_705_MOESM33_ESM.zip › Appendix Figure S6/A/STARD3WT_CHIR/20250210_STARD3WT605_CHIR_8_w1SPI 491 GFP.TIF]

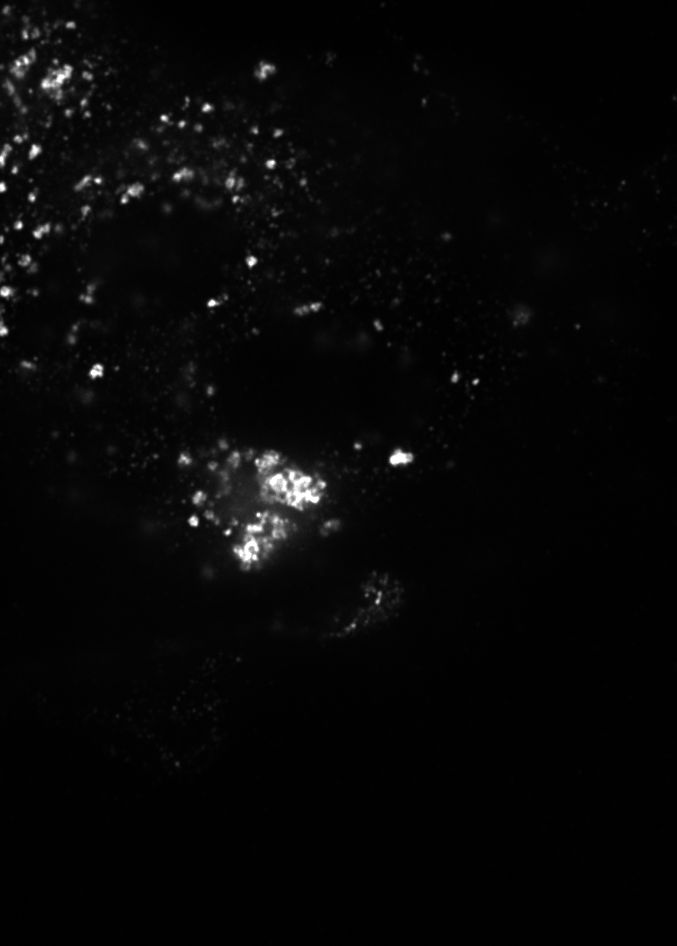

Supplement: Supplementary file 33 — Appendix Fig. S6 Source Data [file 44318_2026_705_MOESM33_ESM.zip › Appendix Figure S6/A/STARD3WT_CHIR/20250210_STARD3WT605_CHIR_8_w2SPI 561 mCherry.TIF]

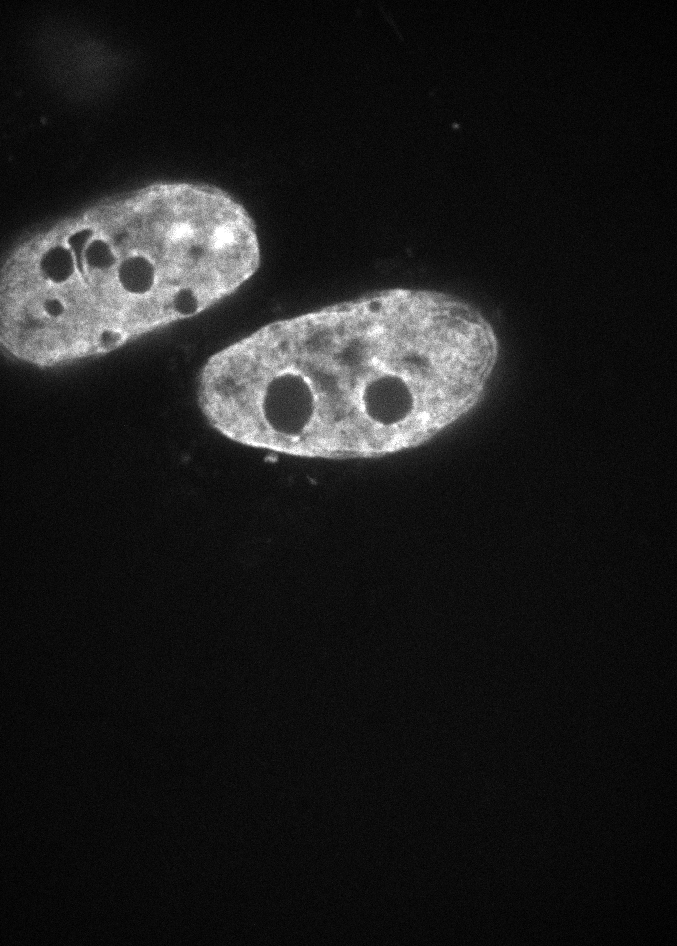

Supplement: Supplementary file 33 — Appendix Fig. S6 Source Data [file 44318_2026_705_MOESM33_ESM.zip › Appendix Figure S6/A/STARD3WT_CHIR/20250210_STARD3WT605_CHIR_8_w3SPI 405 DAPI.TIF]

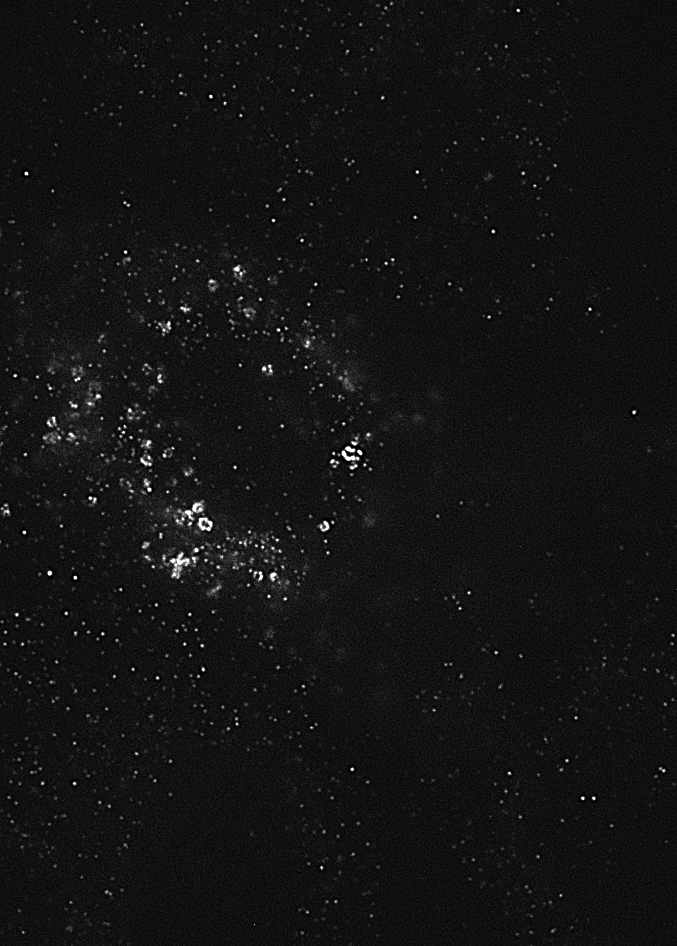

Supplement: Supplementary file 33 — Appendix Fig. S6 Source Data [file 44318_2026_705_MOESM33_ESM.zip › Appendix Figure S6/A/STARD3WT_NT/20250210_STARD3WT605_NT_1_SR_w1SPI 491 GFP.TIF]

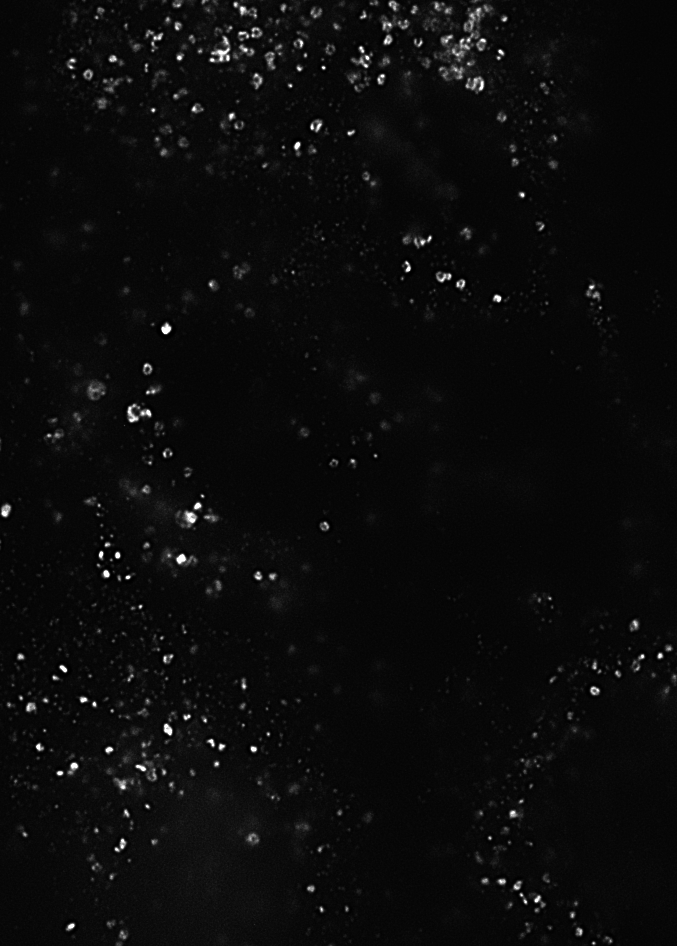

Supplement: Supplementary file 33 — Appendix Fig. S6 Source Data [file 44318_2026_705_MOESM33_ESM.zip › Appendix Figure S6/A/STARD3WT_NT/20250210_STARD3WT605_NT_1_SR_w2SPI 561 mCherry.TIF]

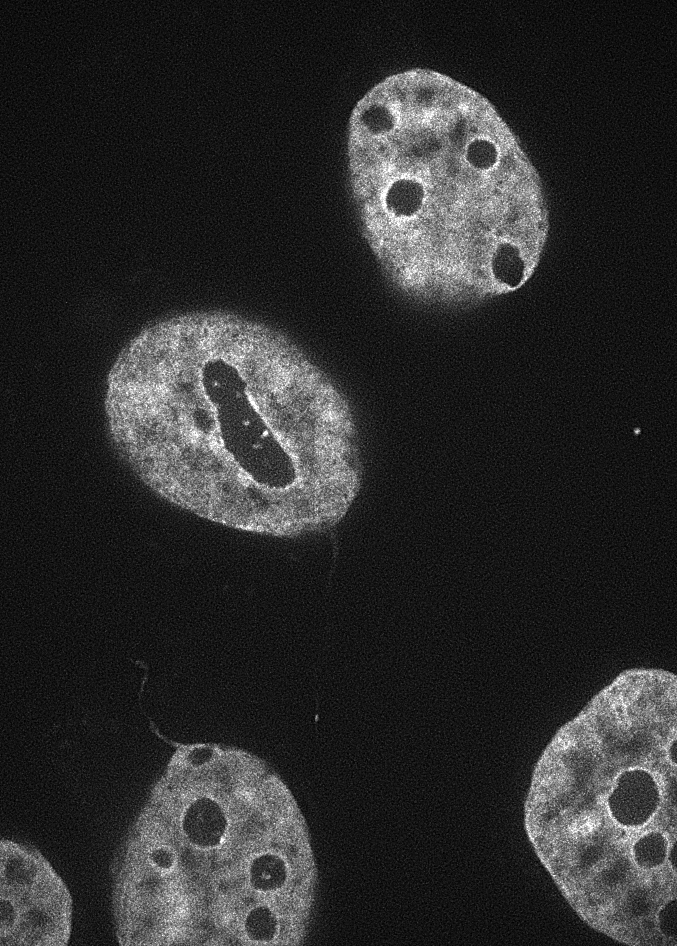

Supplement: Supplementary file 33 — Appendix Fig. S6 Source Data [file 44318_2026_705_MOESM33_ESM.zip › Appendix Figure S6/A/STARD3WT_NT/20250210_STARD3WT605_NT_1_SR_w3SPI 405 DAPI.TIF]

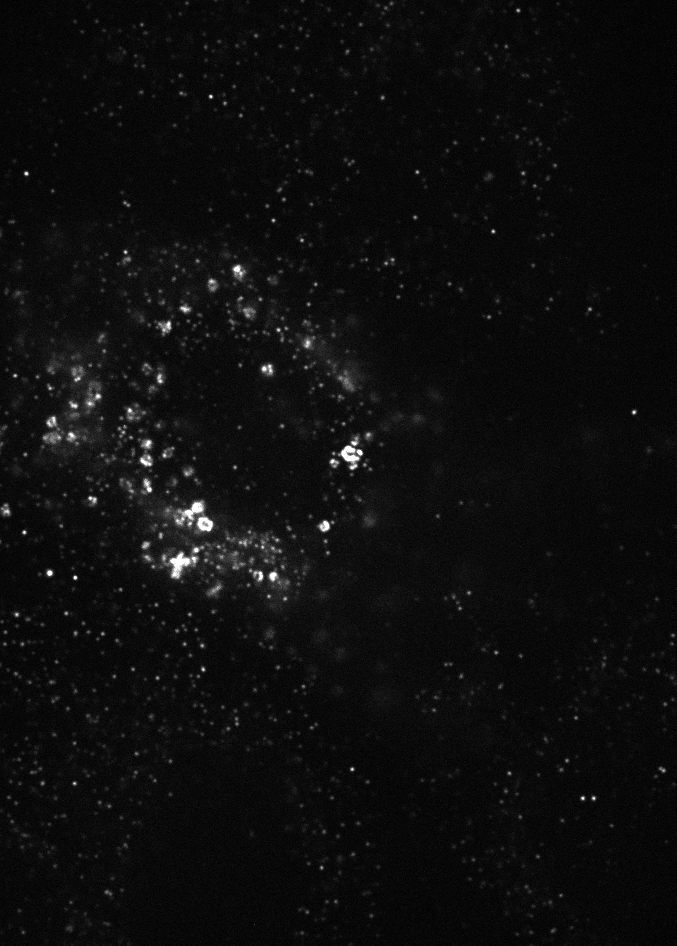

Supplement: Supplementary file 33 — Appendix Fig. S6 Source Data [file 44318_2026_705_MOESM33_ESM.zip › Appendix Figure S6/A/STARD3WT_NT/20250210_STARD3WT605_NT_1_w1SPI 491 GFP.TIF]

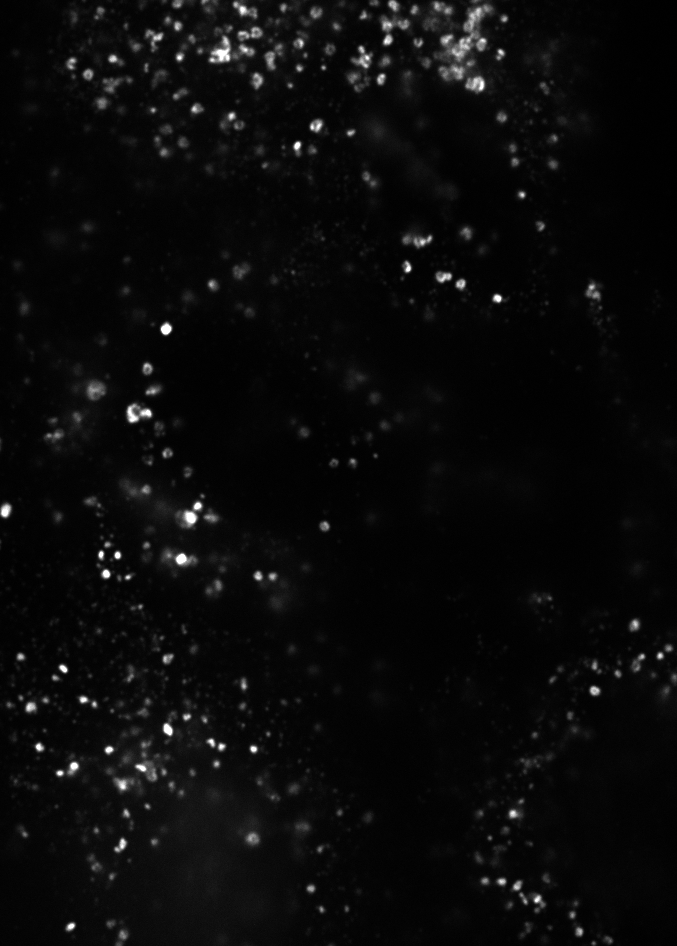

Supplement: Supplementary file 33 — Appendix Fig. S6 Source Data [file 44318_2026_705_MOESM33_ESM.zip › Appendix Figure S6/A/STARD3WT_NT/20250210_STARD3WT605_NT_1_w2SPI 561 mCherry.TIF]

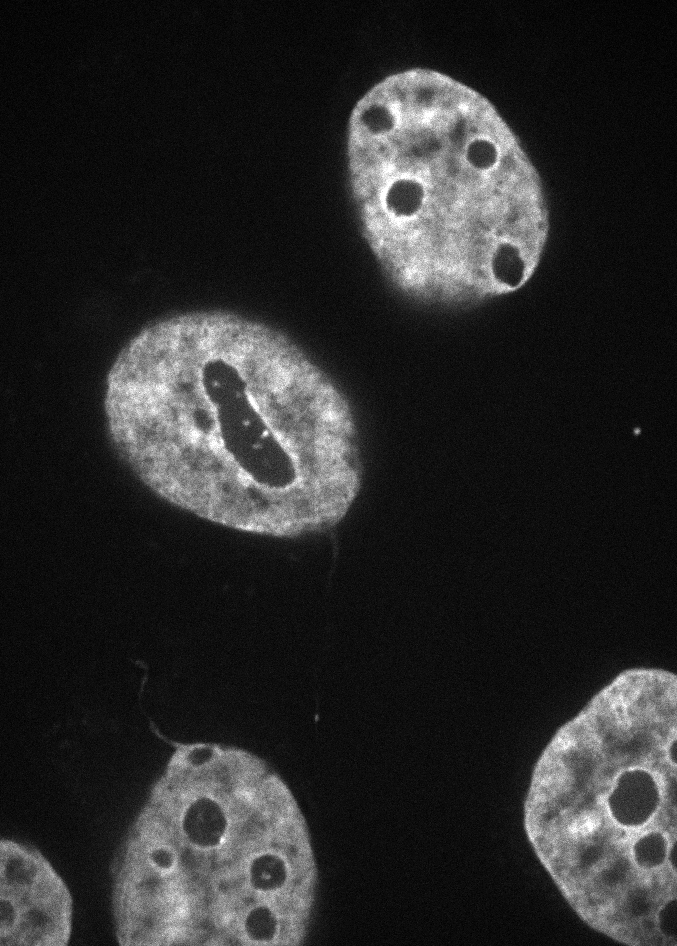

Supplement: Supplementary file 33 — Appendix Fig. S6 Source Data [file 44318_2026_705_MOESM33_ESM.zip › Appendix Figure S6/A/STARD3WT_NT/20250210_STARD3WT605_NT_1_w3SPI 405 DAPI.TIF]

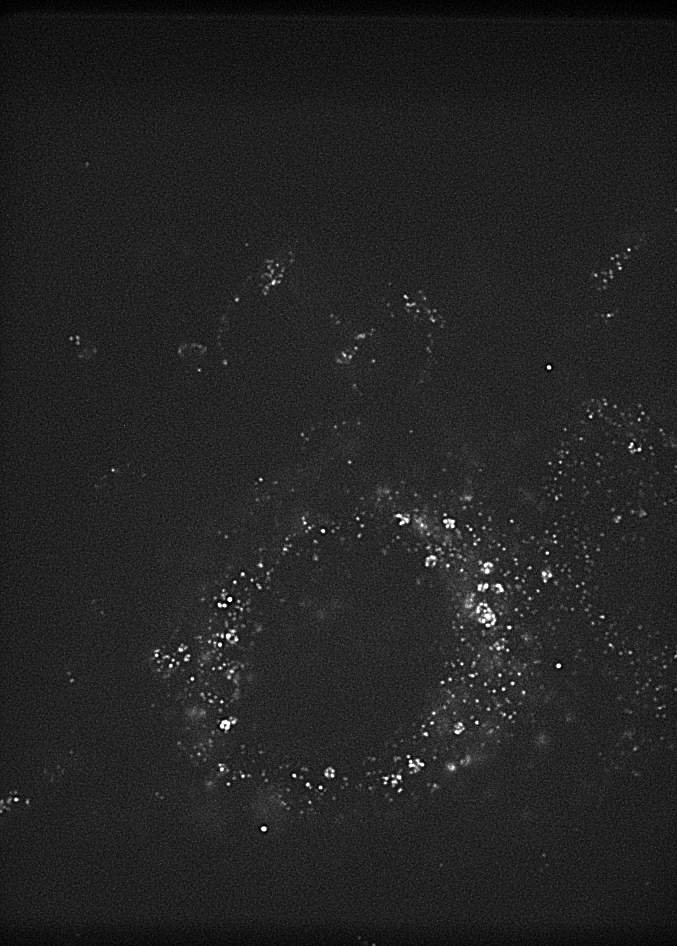

Supplement: Supplementary file 33 — Appendix Fig. S6 Source Data [file 44318_2026_705_MOESM33_ESM.zip › Appendix Figure S6/B/20220825_STARD3NLSTART_SR_w1SPI 491 GFP.TIF]

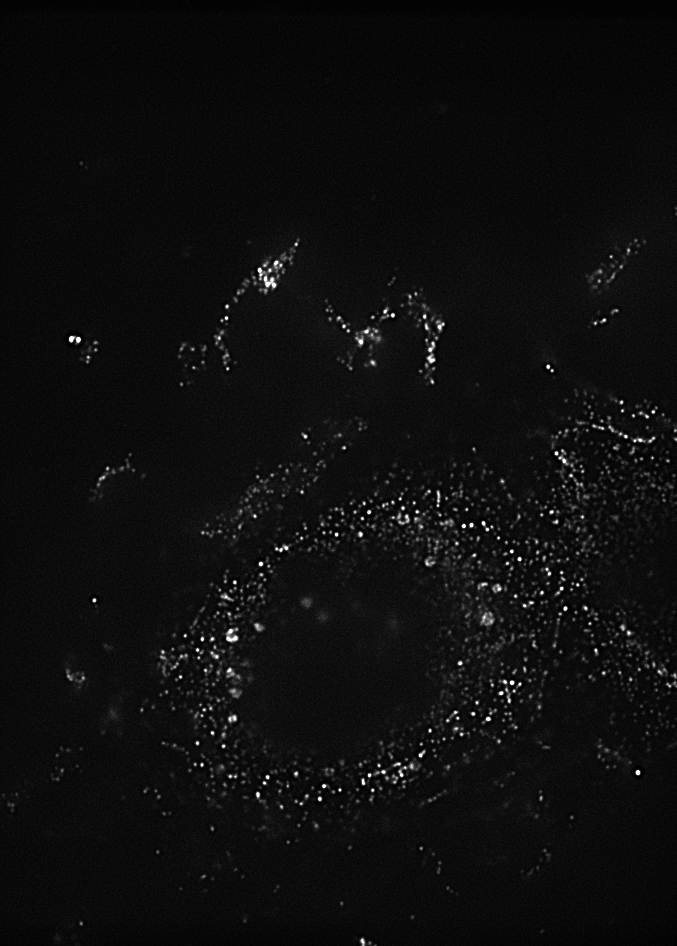

Supplement: Supplementary file 33 — Appendix Fig. S6 Source Data [file 44318_2026_705_MOESM33_ESM.zip › Appendix Figure S6/B/20220825_STARD3NLSTART_SR_w2SPI 561 mCherry.TIF]

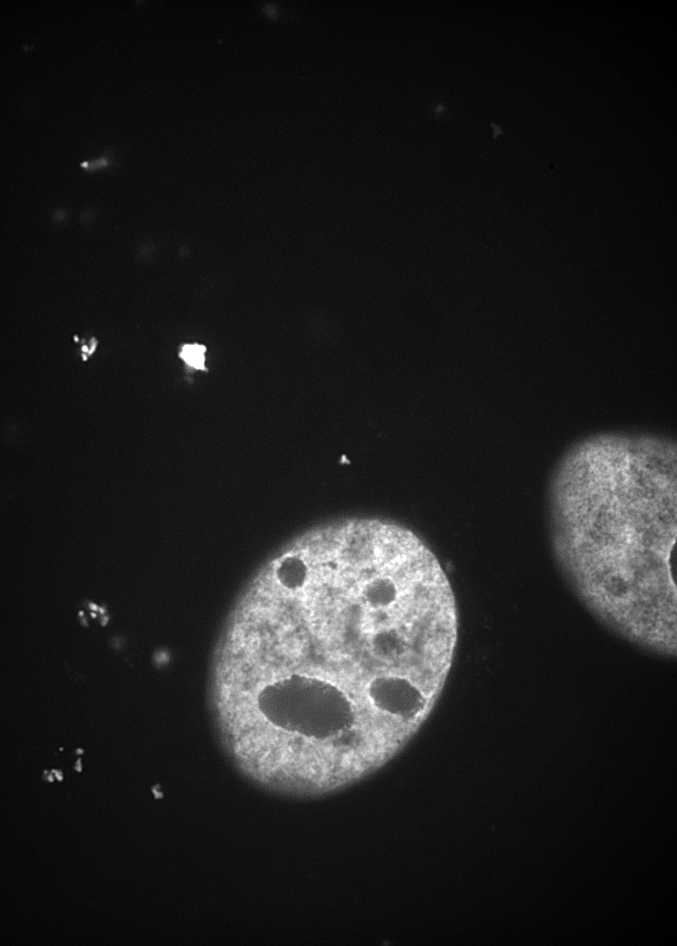

Supplement: Supplementary file 33 — Appendix Fig. S6 Source Data [file 44318_2026_705_MOESM33_ESM.zip › Appendix Figure S6/B/20220825_STARD3NLSTART_SR_w3SPI 405 DAPI.TIF]

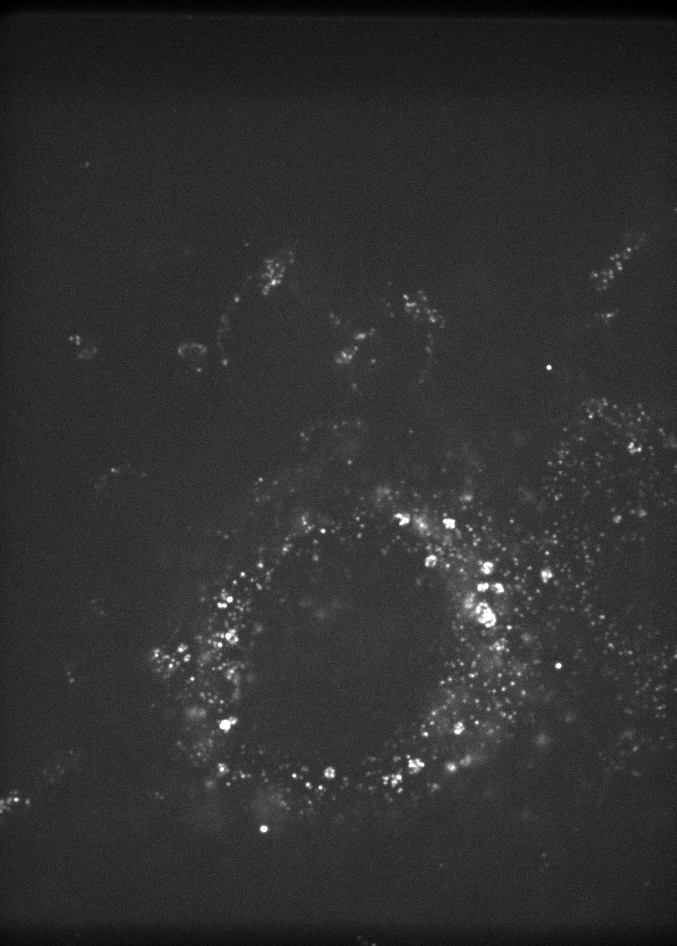

Supplement: Supplementary file 33 — Appendix Fig. S6 Source Data [file 44318_2026_705_MOESM33_ESM.zip › Appendix Figure S6/B/20220825_STARD3NLSTART_w1SPI 491 GFP.TIF]

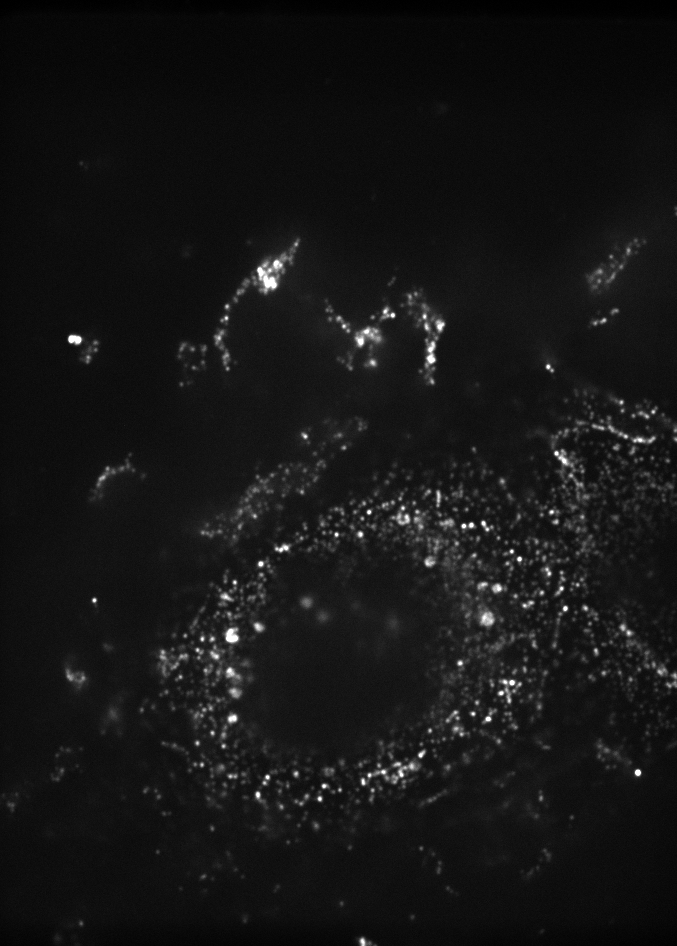

Supplement: Supplementary file 33 — Appendix Fig. S6 Source Data [file 44318_2026_705_MOESM33_ESM.zip › Appendix Figure S6/B/20220825_STARD3NLSTART_w2SPI 561 mCherry.TIF]

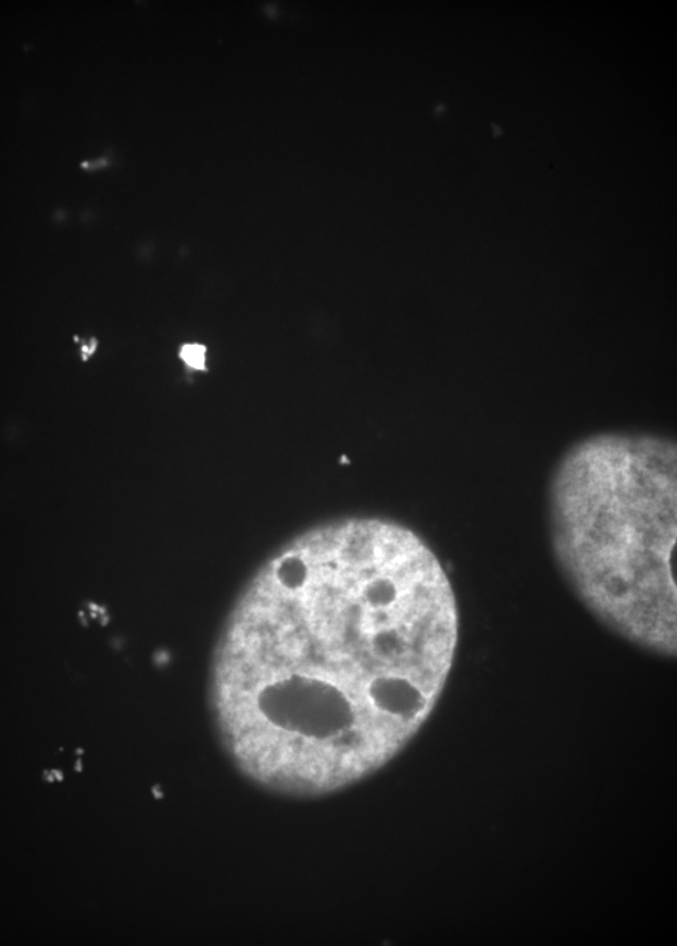

Supplement: Supplementary file 33 — Appendix Fig. S6 Source Data [file 44318_2026_705_MOESM33_ESM.zip › Appendix Figure S6/B/20220825_STARD3NLSTART_w3SPI 405 DAPI.TIF]

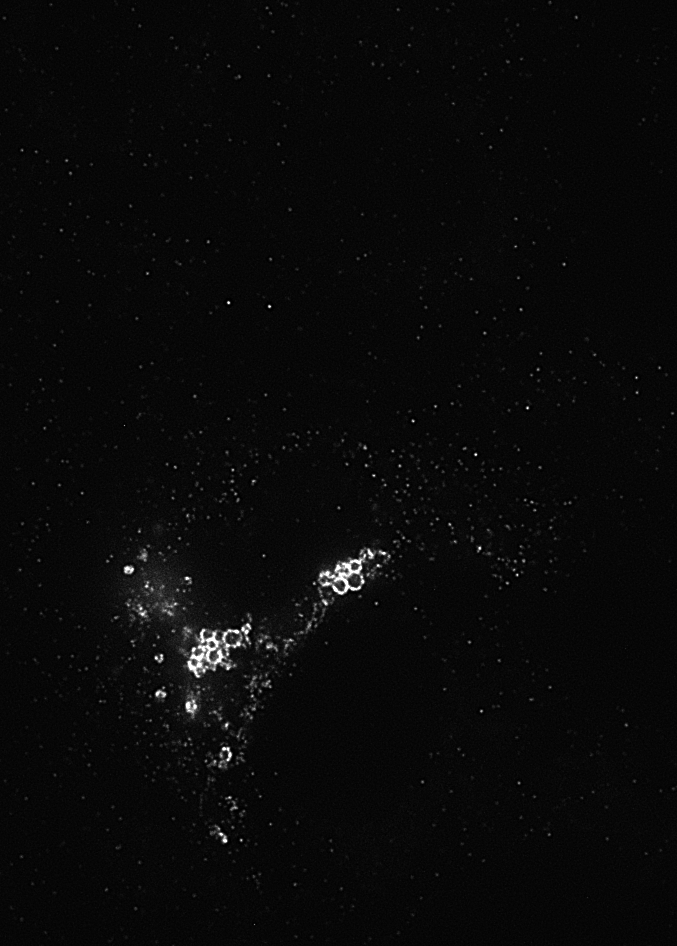

Supplement: Supplementary file 33 — Appendix Fig. S6 Source Data [file 44318_2026_705_MOESM33_ESM.zip › Appendix Figure S6/C/TMEM192START_CHIR/20250228_MCF7TMEMSTART_CHIR_2_SR_w1SPI 491 GFP.TIF]

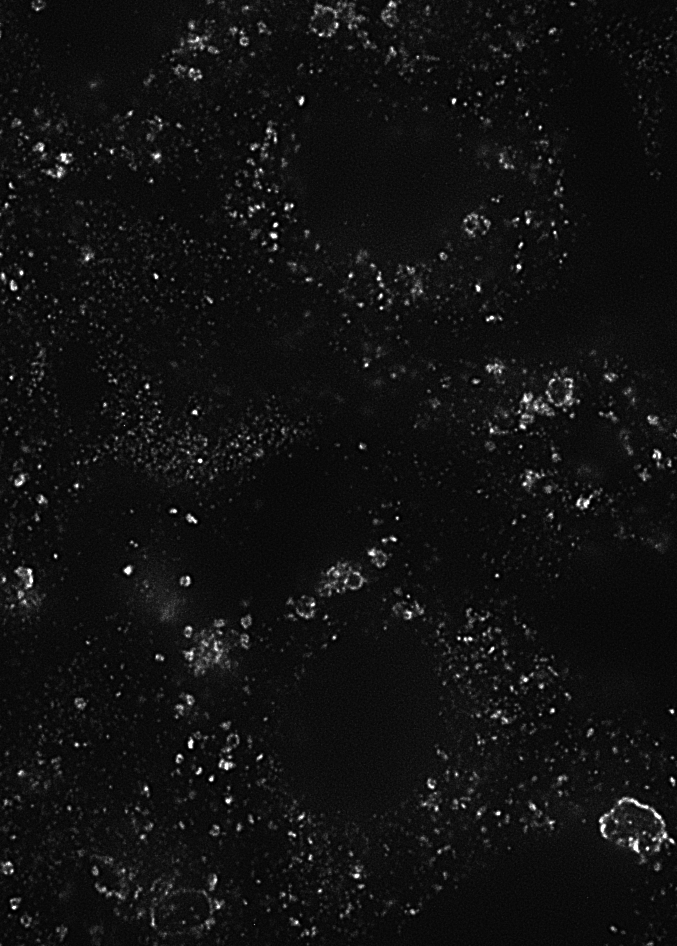

Supplement: Supplementary file 33 — Appendix Fig. S6 Source Data [file 44318_2026_705_MOESM33_ESM.zip › Appendix Figure S6/C/TMEM192START_CHIR/20250228_MCF7TMEMSTART_CHIR_2_SR_w2SPI 561 mCherry.TIF]

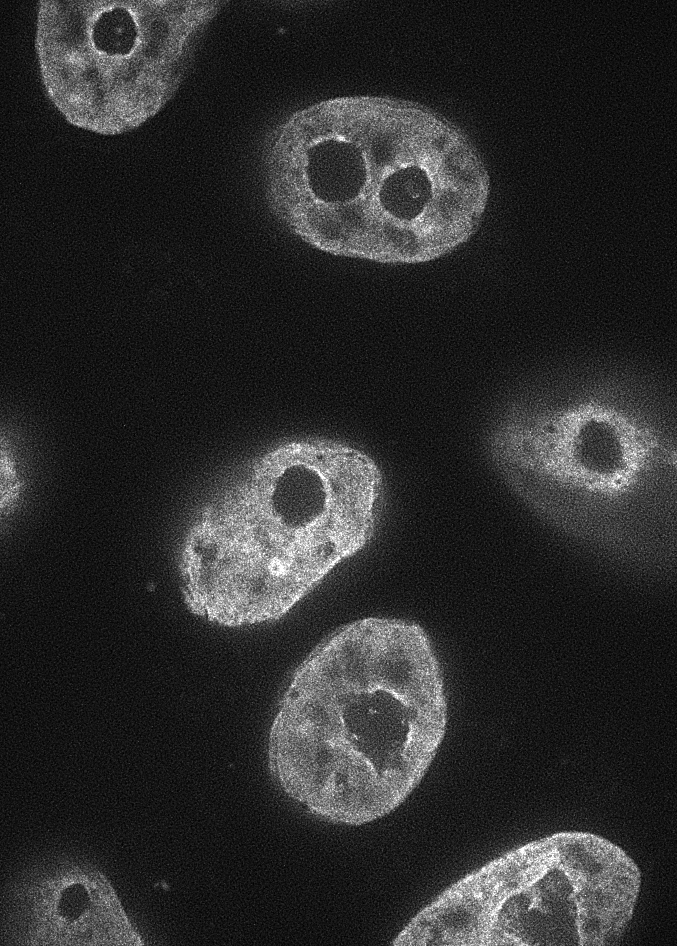

Supplement: Supplementary file 33 — Appendix Fig. S6 Source Data [file 44318_2026_705_MOESM33_ESM.zip › Appendix Figure S6/C/TMEM192START_CHIR/20250228_MCF7TMEMSTART_CHIR_2_SR_w3SPI 405 DAPI.TIF]

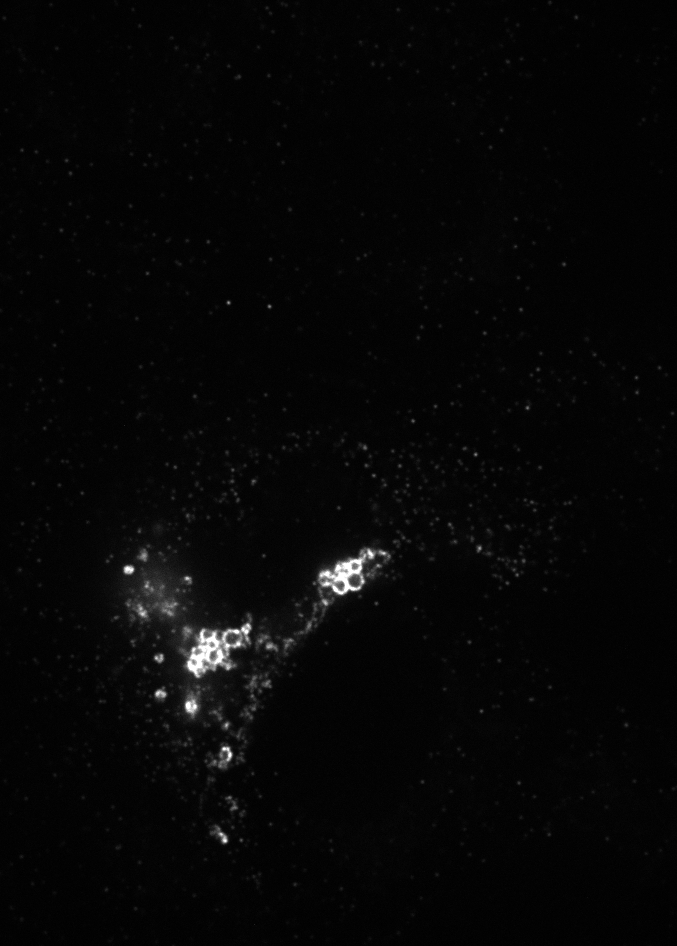

Supplement: Supplementary file 33 — Appendix Fig. S6 Source Data [file 44318_2026_705_MOESM33_ESM.zip › Appendix Figure S6/C/TMEM192START_CHIR/20250228_MCF7TMEMSTART_CHIR_2_w1SPI 491 GFP.TIF]

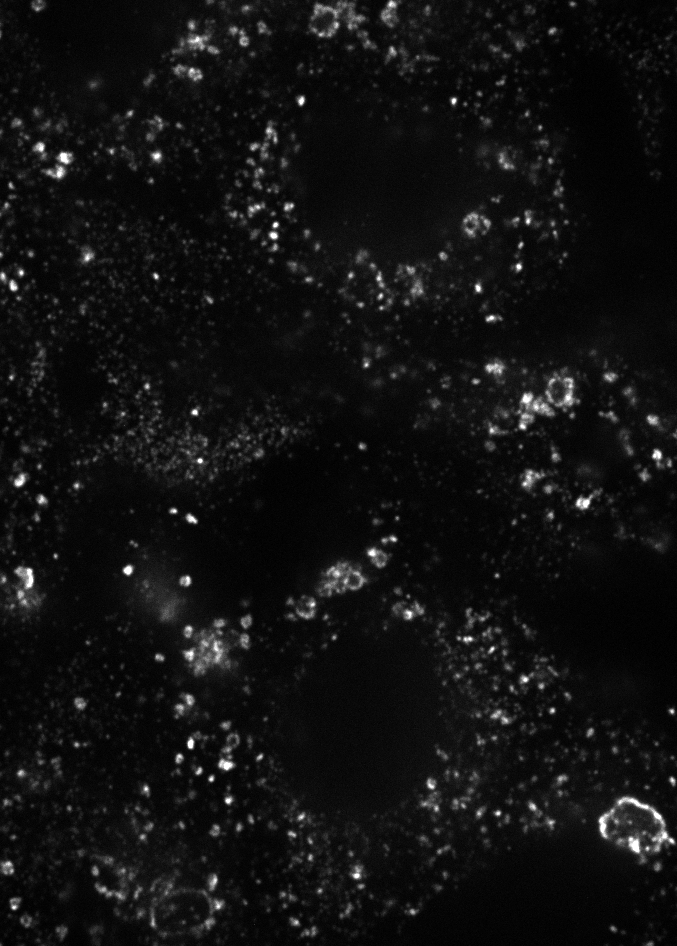

Supplement: Supplementary file 33 — Appendix Fig. S6 Source Data [file 44318_2026_705_MOESM33_ESM.zip › Appendix Figure S6/C/TMEM192START_CHIR/20250228_MCF7TMEMSTART_CHIR_2_w2SPI 561 mCherry.TIF]

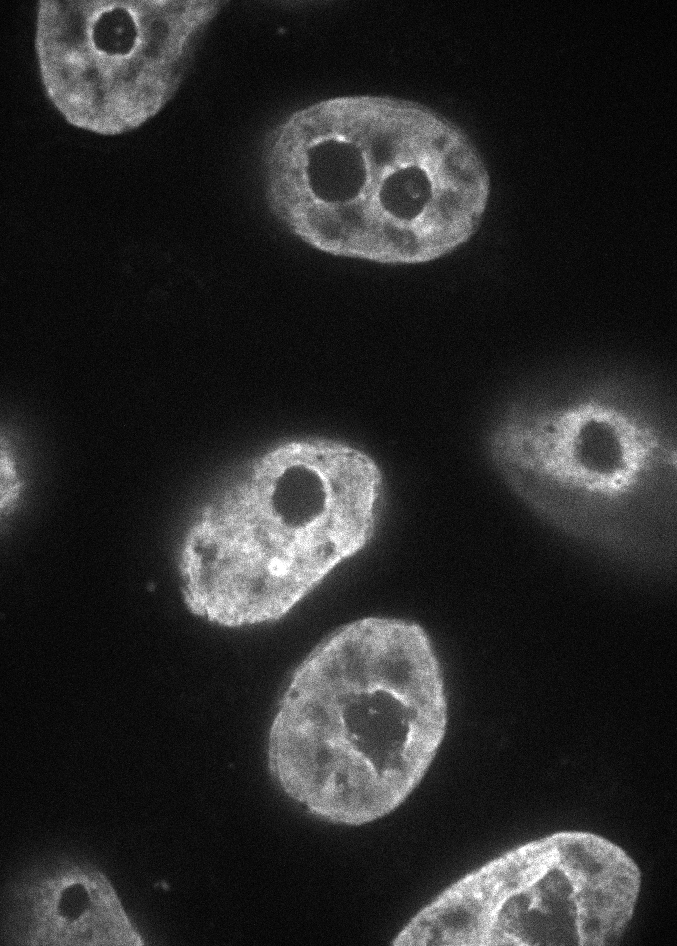

Supplement: Supplementary file 33 — Appendix Fig. S6 Source Data [file 44318_2026_705_MOESM33_ESM.zip › Appendix Figure S6/C/TMEM192START_CHIR/20250228_MCF7TMEMSTART_CHIR_2_w3SPI 405 DAPI.TIF]

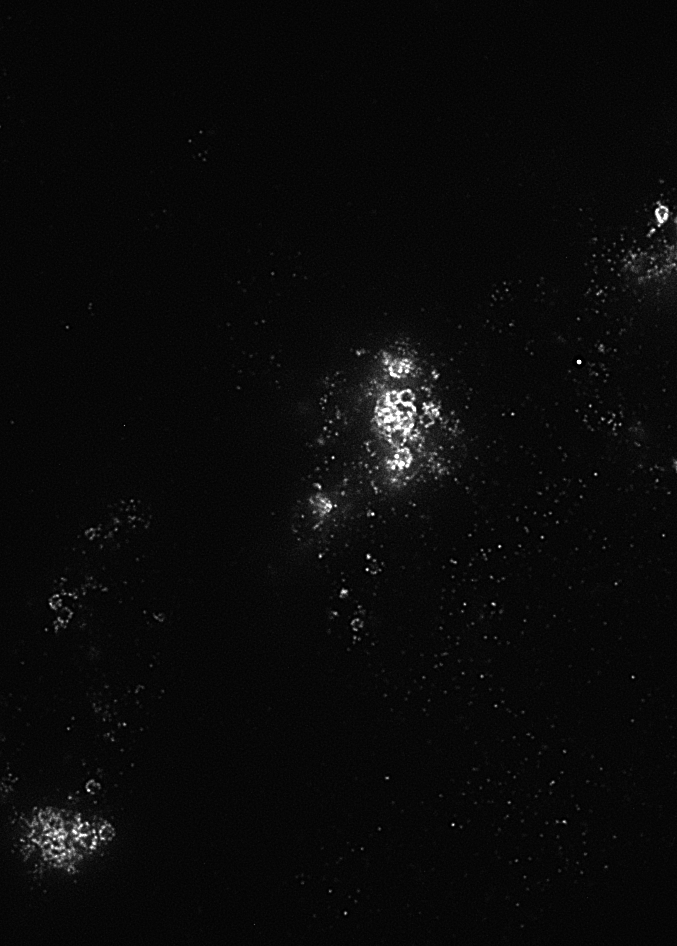

Supplement: Supplementary file 33 — Appendix Fig. S6 Source Data [file 44318_2026_705_MOESM33_ESM.zip › Appendix Figure S6/C/TMEM192START_NT/20250211_TMEMSTART_NT_1_SR_w1SPI 491 GFP.TIF]

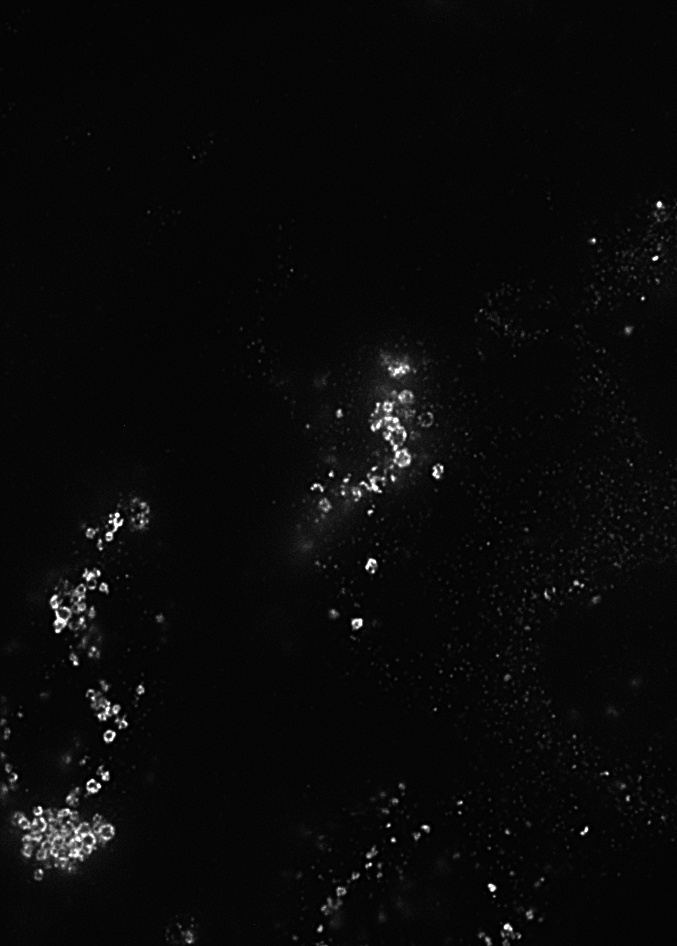

Supplement: Supplementary file 33 — Appendix Fig. S6 Source Data [file 44318_2026_705_MOESM33_ESM.zip › Appendix Figure S6/C/TMEM192START_NT/20250211_TMEMSTART_NT_1_SR_w2SPI 561 mCherry.TIF]

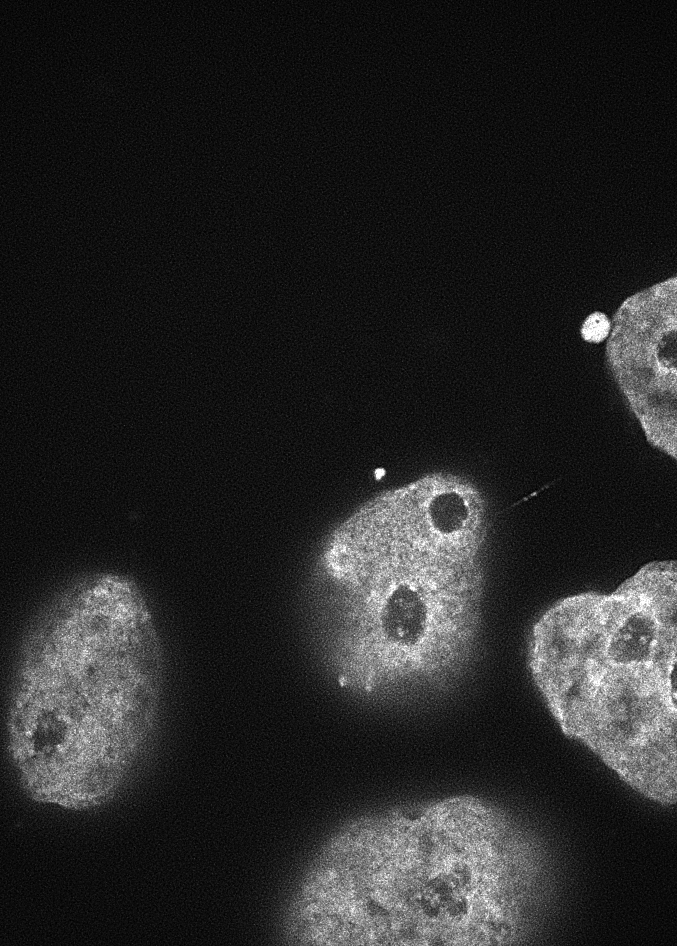

Supplement: Supplementary file 33 — Appendix Fig. S6 Source Data [file 44318_2026_705_MOESM33_ESM.zip › Appendix Figure S6/C/TMEM192START_NT/20250211_TMEMSTART_NT_1_SR_w3SPI 405 DAPI.TIF]

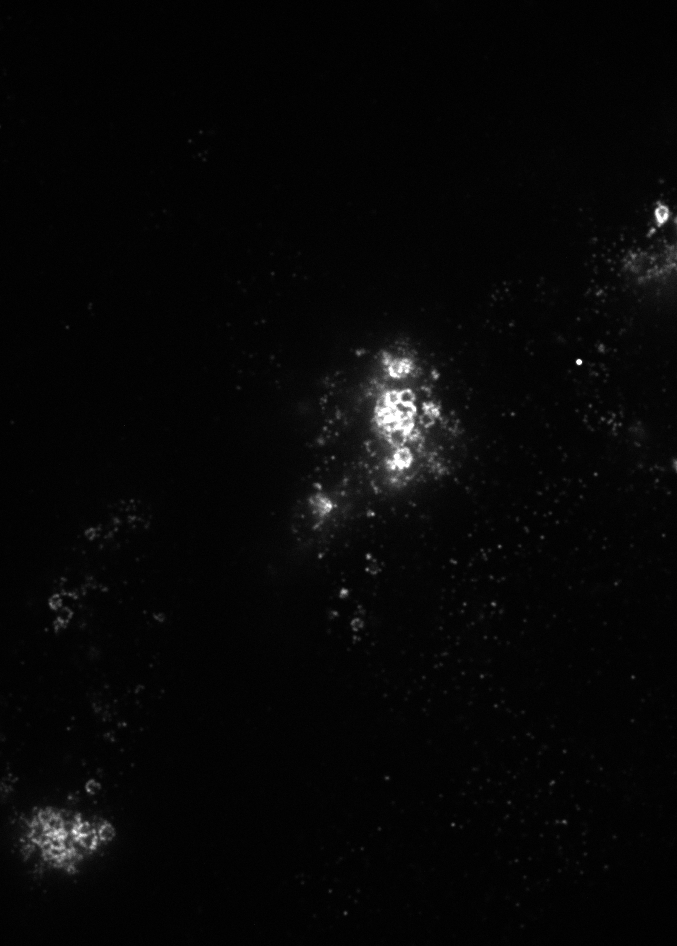

Supplement: Supplementary file 33 — Appendix Fig. S6 Source Data [file 44318_2026_705_MOESM33_ESM.zip › Appendix Figure S6/C/TMEM192START_NT/20250211_TMEMSTART_NT_1_w1SPI 491 GFP.TIF]

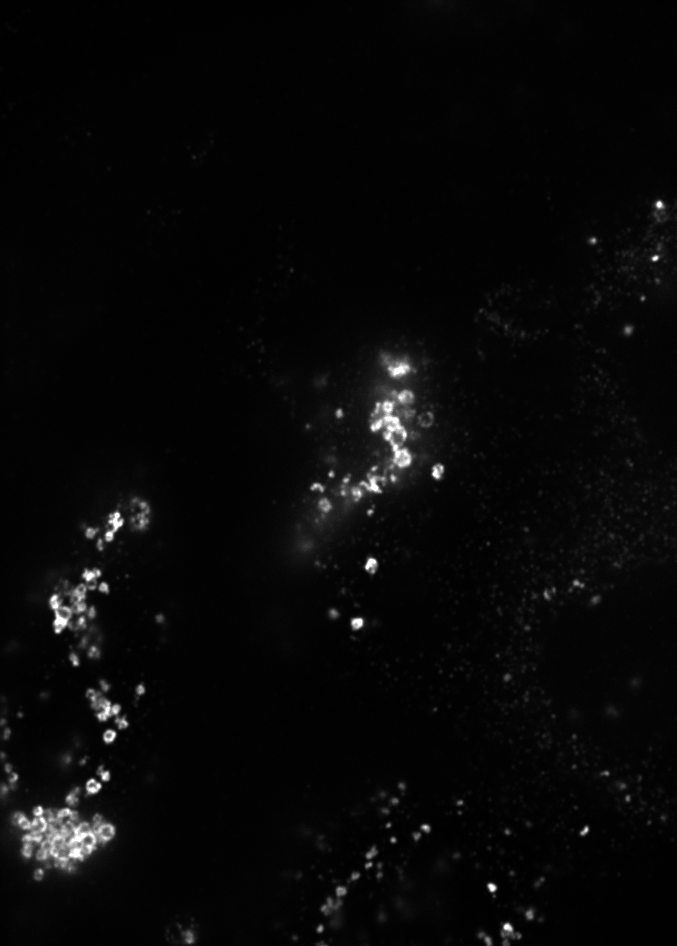

Supplement: Supplementary file 33 — Appendix Fig. S6 Source Data [file 44318_2026_705_MOESM33_ESM.zip › Appendix Figure S6/C/TMEM192START_NT/20250211_TMEMSTART_NT_1_w2SPI 561 mCherry.TIF]

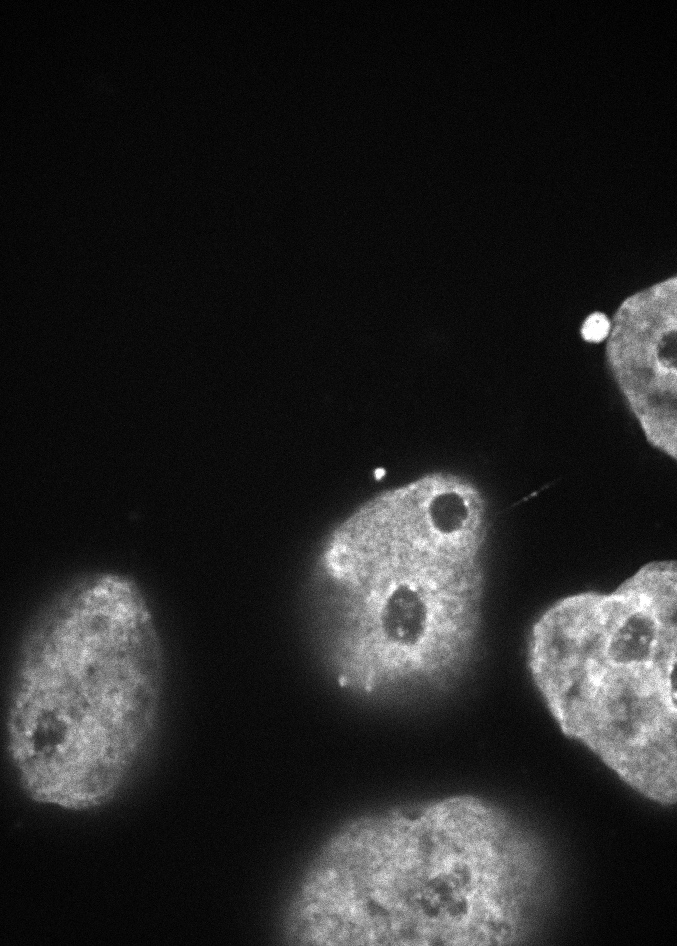

Supplement: Supplementary file 33 — Appendix Fig. S6 Source Data [file 44318_2026_705_MOESM33_ESM.zip › Appendix Figure S6/C/TMEM192START_NT/20250211_TMEMSTART_NT_1_w3SPI 405 DAPI.TIF]

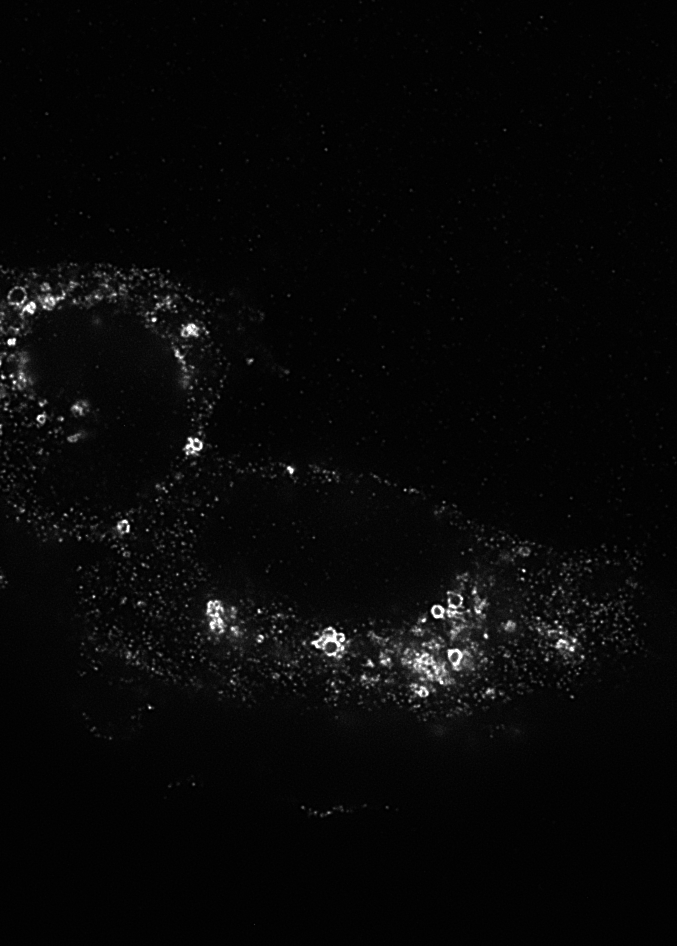

Supplement: Supplementary file 33 — Appendix Fig. S6 Source Data [file 44318_2026_705_MOESM33_ESM.zip › Appendix Figure S6/D/LysoSTART_CHIR/20250228_MCF7STARD3LAMTOR_CHIR_4_SR_w1SPI 491 GFP.TIF]

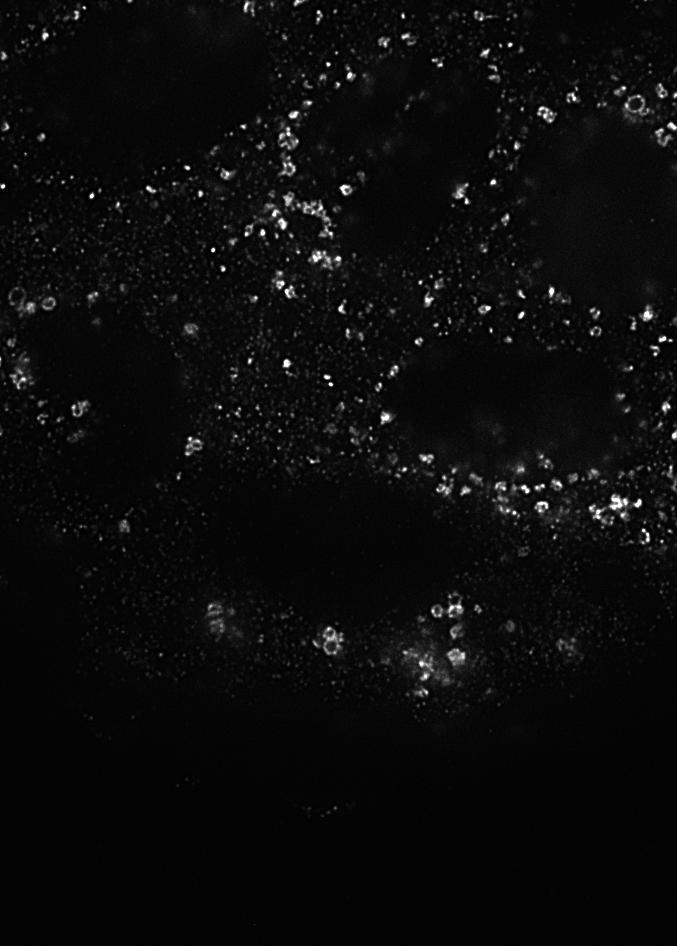

Supplement: Supplementary file 33 — Appendix Fig. S6 Source Data [file 44318_2026_705_MOESM33_ESM.zip › Appendix Figure S6/D/LysoSTART_CHIR/20250228_MCF7STARD3LAMTOR_CHIR_4_SR_w2SPI 561 mCherry.TIF]

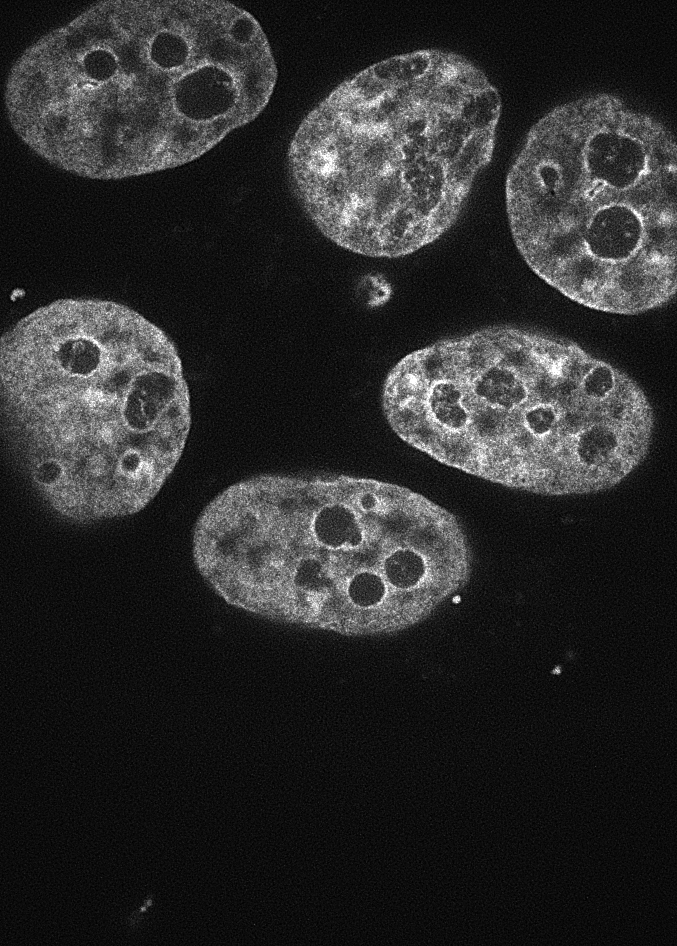

Supplement: Supplementary file 33 — Appendix Fig. S6 Source Data [file 44318_2026_705_MOESM33_ESM.zip › Appendix Figure S6/D/LysoSTART_CHIR/20250228_MCF7STARD3LAMTOR_CHIR_4_SR_w3SPI 405 DAPI.TIF]

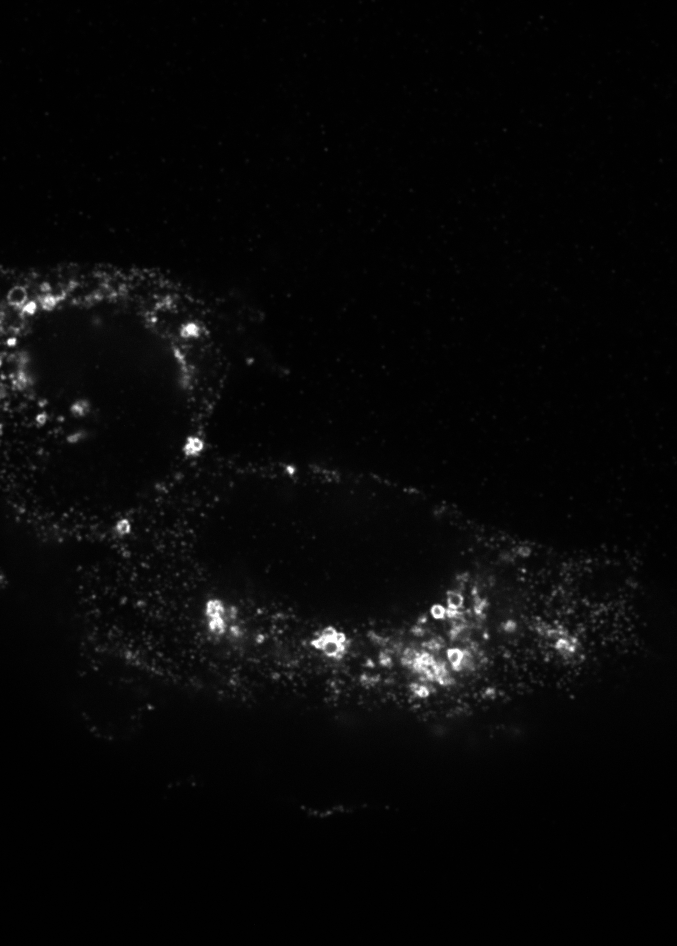

Supplement: Supplementary file 33 — Appendix Fig. S6 Source Data [file 44318_2026_705_MOESM33_ESM.zip › Appendix Figure S6/D/LysoSTART_CHIR/20250228_MCF7STARD3LAMTOR_CHIR_4_w1SPI 491 GFP.TIF]

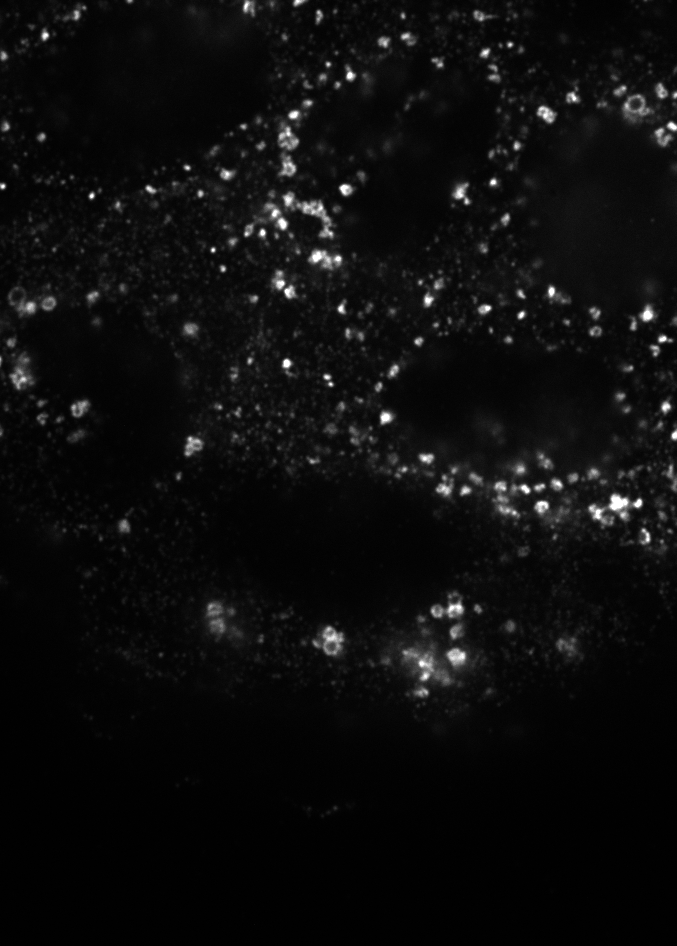

Supplement: Supplementary file 33 — Appendix Fig. S6 Source Data [file 44318_2026_705_MOESM33_ESM.zip › Appendix Figure S6/D/LysoSTART_CHIR/20250228_MCF7STARD3LAMTOR_CHIR_4_w2SPI 561 mCherry.TIF]

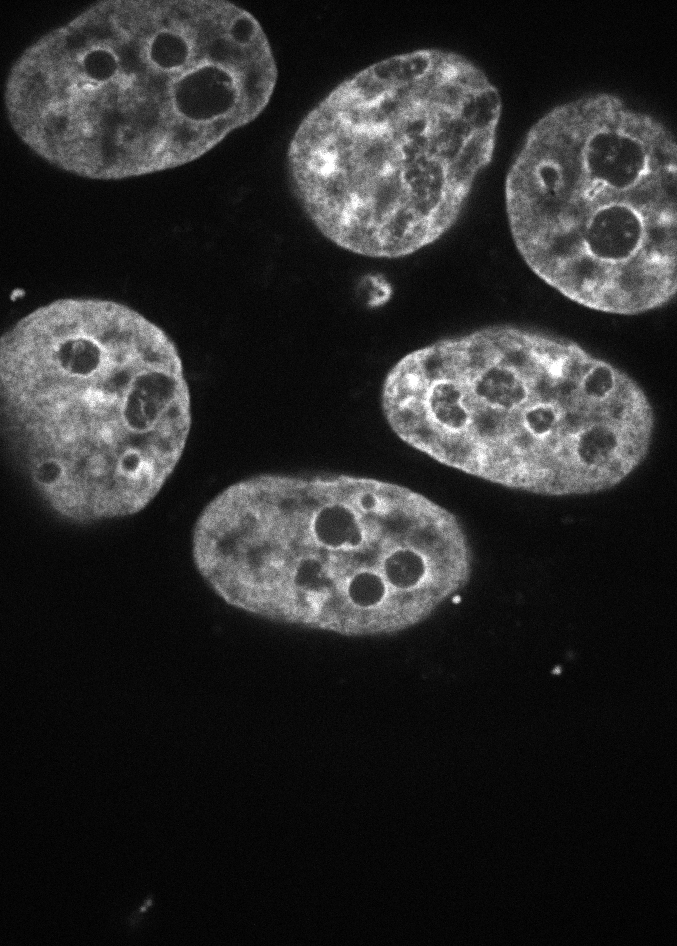

Supplement: Supplementary file 33 — Appendix Fig. S6 Source Data [file 44318_2026_705_MOESM33_ESM.zip › Appendix Figure S6/D/LysoSTART_CHIR/20250228_MCF7STARD3LAMTOR_CHIR_4_w3SPI 405 DAPI.TIF]

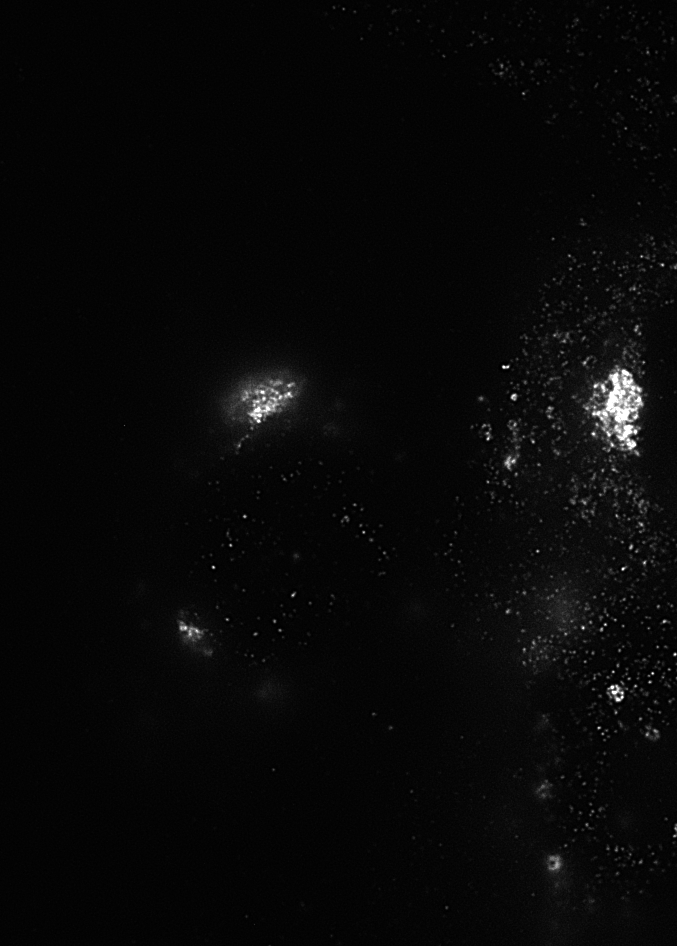

Supplement: Supplementary file 33 — Appendix Fig. S6 Source Data [file 44318_2026_705_MOESM33_ESM.zip › Appendix Figure S6/D/LysoSTART_NT/20250210_LAMTORSTART605_NT_1_SR_w1SPI 491 GFP.TIF]

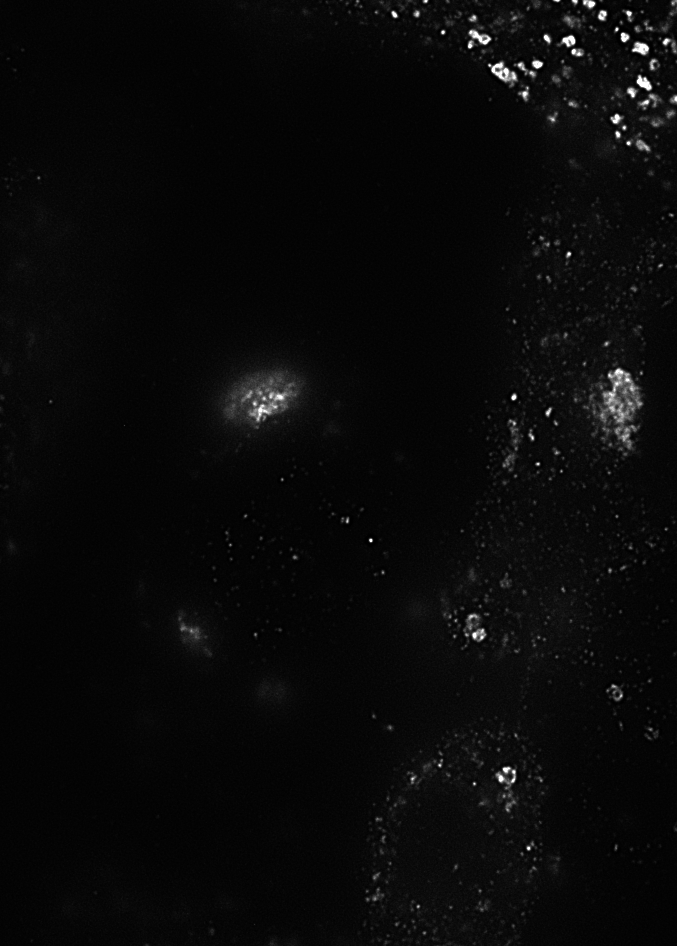

Supplement: Supplementary file 33 — Appendix Fig. S6 Source Data [file 44318_2026_705_MOESM33_ESM.zip › Appendix Figure S6/D/LysoSTART_NT/20250210_LAMTORSTART605_NT_1_SR_w2SPI 561 mCherry.TIF]

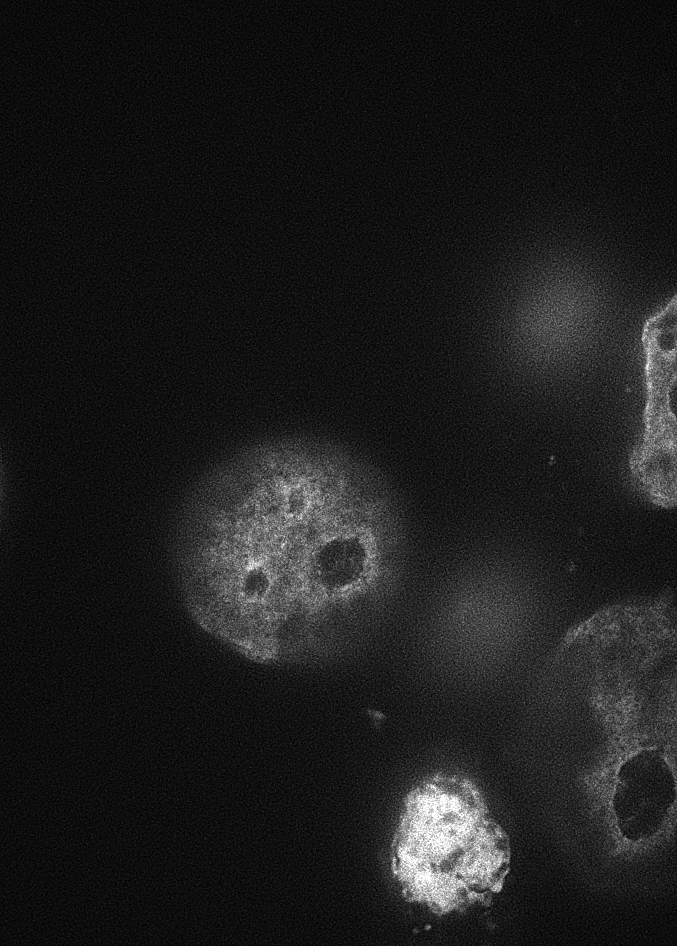

Supplement: Supplementary file 33 — Appendix Fig. S6 Source Data [file 44318_2026_705_MOESM33_ESM.zip › Appendix Figure S6/D/LysoSTART_NT/20250210_LAMTORSTART605_NT_1_SR_w3SPI 405 DAPI.TIF]

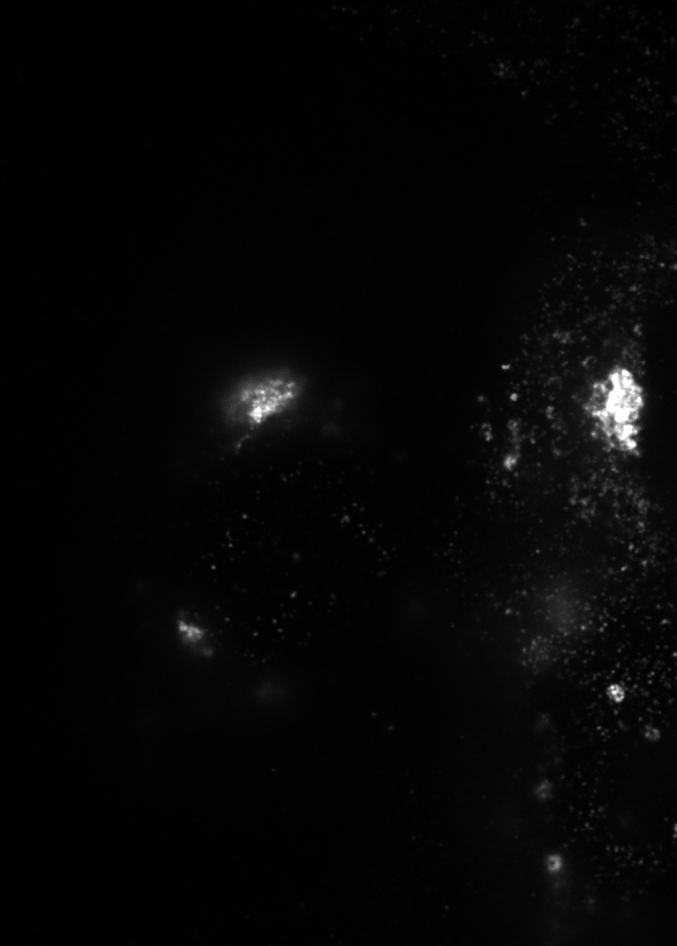

Supplement: Supplementary file 33 — Appendix Fig. S6 Source Data [file 44318_2026_705_MOESM33_ESM.zip › Appendix Figure S6/D/LysoSTART_NT/20250210_LAMTORSTART605_NT_1_w1SPI 491 GFP.TIF]

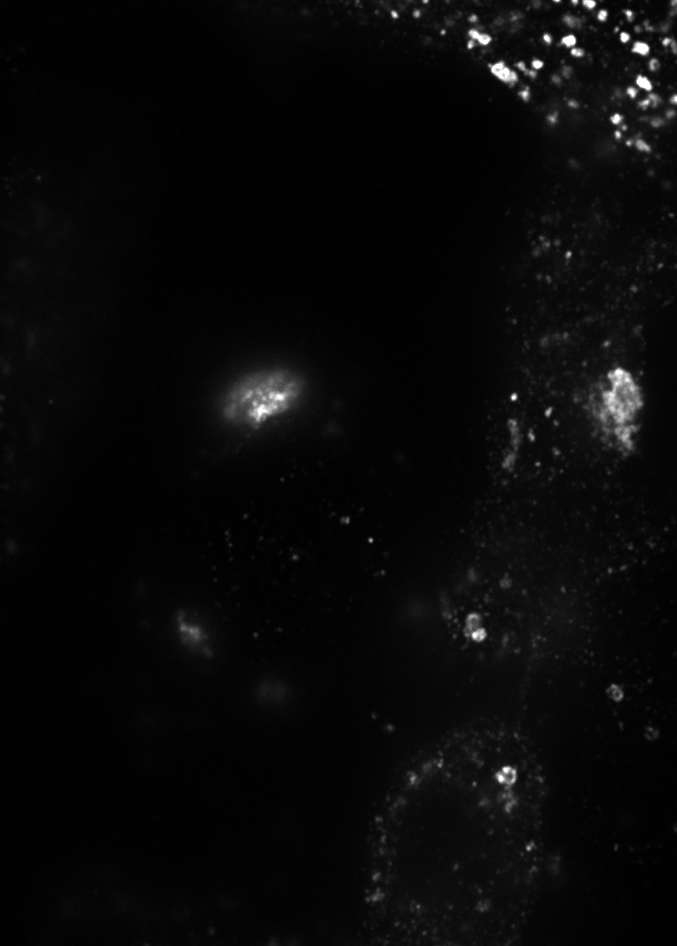

Supplement: Supplementary file 33 — Appendix Fig. S6 Source Data [file 44318_2026_705_MOESM33_ESM.zip › Appendix Figure S6/D/LysoSTART_NT/20250210_LAMTORSTART605_NT_1_w2SPI 561 mCherry.TIF]

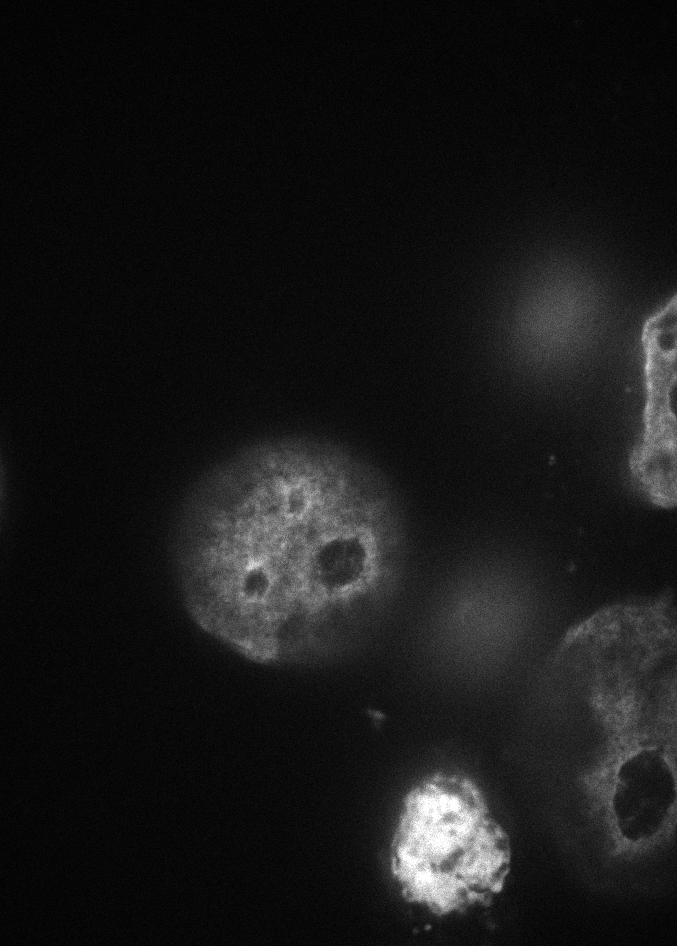

Supplement: Supplementary file 33 — Appendix Fig. S6 Source Data [file 44318_2026_705_MOESM33_ESM.zip › Appendix Figure S6/D/LysoSTART_NT/20250210_LAMTORSTART605_NT_1_w3SPI 405 DAPI.TIF]
