# Supplementary material for: STARD3 regulates lysosome positioning and contacts via a GSK3-controlled phosphorylation switch
Source: EMBO J. 2026 Feb 25;45(7):2239–77. doi: 10.1038/s44318-026-00705-3 (PMC13044316; doi:10.1038/s44318-026-00705-3)
Supplement: Supplementary file 34 — Expanded View Figures [file 44318_2026_705_MOESM34_ESM.pdf]

Expanded View Figures

Figure EV1. Dose and time-dependent effects of GSK3 inhibition on STARD3 phosphorylation.

(**A**) Western blot analysis of MCF7 cells overexpressing TIP60 treated or not with the GSK3 inhibitor CHIR99021 (5 µM; overnight). TIP60 protein levels (Total) and S_{586} phosphorylation (pS_{586}) were analyzed. (**B**) Western blot analysis of HeLa/STARD3 cells treated overnight with different concentration of CHIR99021 (1, 2 and 5 µM) or left untreated. STARD3 protein level (Total) and S_{209} phosphorylation (pS_{209}) were analyzed. (**C**) (a) Western blot analysis of HCC1954 cells treated with 5 µM of CHIR99021 for 2, 4, 6, 8 and 16 h, or left untreated. STARD3 protein levels (Total) and S_{209} phosphorylation (pS_{209}) were analyzed. (b) Quantification of relative S_{209} phosphorylation levels. Means ± SD. One-way ANOVA with Dunnett's multiple comparison test (*, $P < 0.05$; ***, $P < 0.001$; $n = 3$ independent experiments; 0 h vs 2 h, $P = 0.33$; 0 h vs 4 h, $P = 0.06$; 0 h vs 6 h, $P = 2.3 \times 10^{-2}$; 0 h vs 8 h, $P = 1.9 \times 10^{-2}$; 0 h vs 16 h, $P = 8 \times 10^{-4}$). (c) Representative images of HCC1954 cells expressing WT STARD3 and treated with the GSK3 inhibitor CHIR99021 for 2, 4, 6, 8 and 16 h, or left untreated. Cells were labeled with an anti-LAMP1 antibody (magenta), an anti-STARD3 antibody (green) and Hoechst for nuclei (blue). (**D**, **E**) Quantification of relative GSK3α and GSK3β in HCC1954 (**D**) and MCF7/STARD3 (**E**) cells transfected with control siRNAs (siCtrl) or siRNAs targeting GSK3α (siGSK3α), GSK3β (siGSK3β), or both (siGSK3α + siGSK3β) (see Fig. 1E,F). Means ± SD. One-way ANOVA with Dunnett's multiple comparison test (*, $P < 0.05$; **, $P < 0.01$; ****, $P < 0.0001$, $n = 3$–4 independent experiments; D, GSK3α: siCtrl vs WT, $P = 0.76$; GSK3α, $P = 9 \times 10^{-3}$; siCtrl vs siGSK3β, $P = 0.52$; siCtrl vs siGSK3α + siGSK3β, $P = 1.1 \times 10^{-2}$; (**D**), GSK3 β: siCtrl vs WT, $P = 0.5$; GSK3α, $P = 0.99$; siCtrl vs siGSK3β, $P = 3.6 \times 10^{-3}$; siCtrl vs siGSK3α + siGSK3β, $P = 1.6 \times 10^{-3}$; (**E**), GSK3α: siCtrl vs WT, $P = 0.94$; GSK3α, $P = 1.3 \times 10^{-2}$; siCtrl vs siGSK3β, $P = 0.97$; siCtrl vs siGSK3α + siGSK3β, $P = 8 \times 10^{-3}$; (**E**), GSK3 β: siCtrl vs WT, $P = 0.93$; GSK3α, $P = 0.28$; siCtrl vs siGSK3β, $P < 10^{-4}$; siCtrl vs siGSK3α + siGSK3β, $P < 10^{-4}$). Source data are available online for this figure.

© The Author(s)

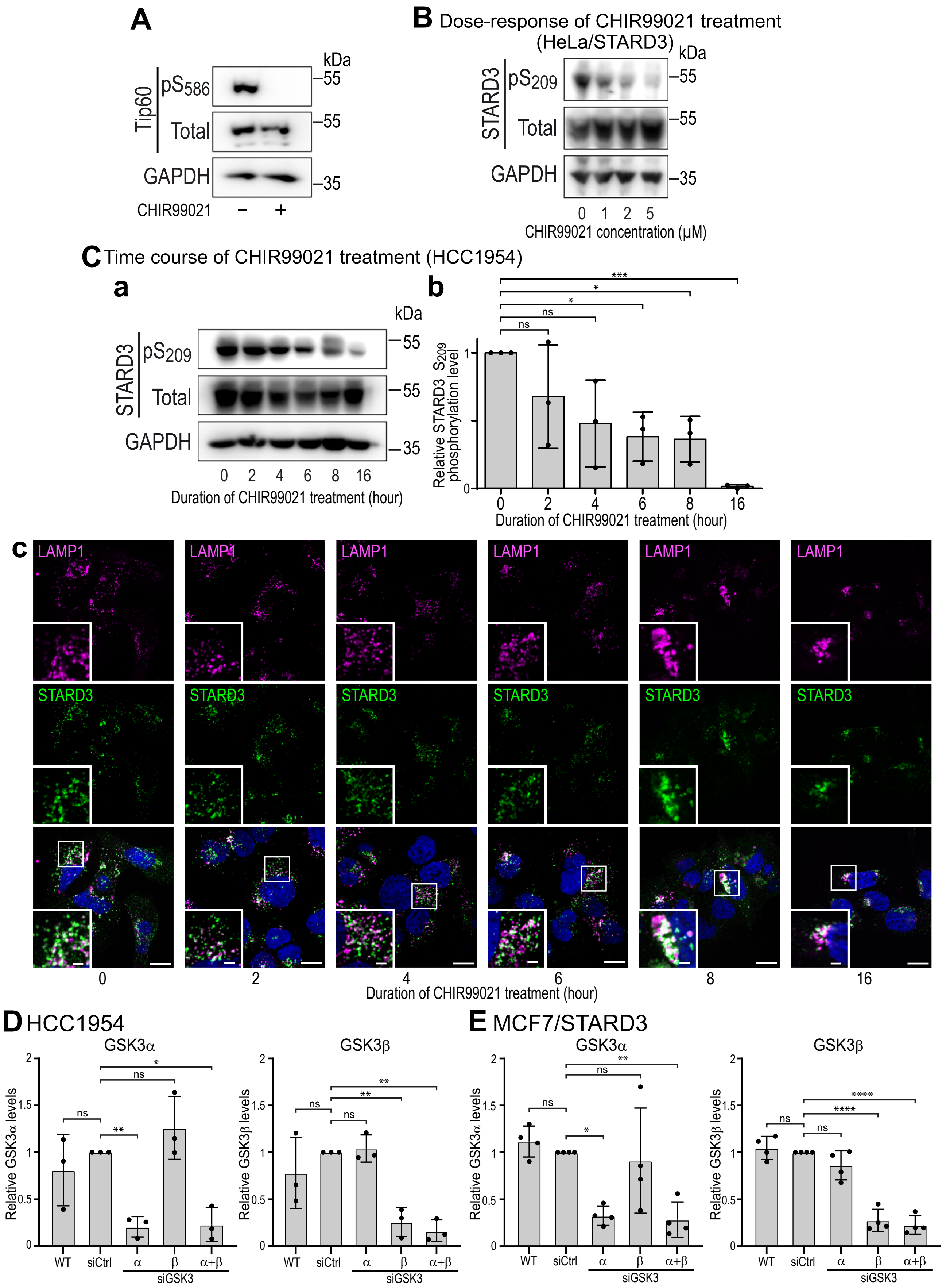
A
Tip60
pS586
Total
GAPDH
kDa
55
55
35
CHIR99021 - +
B Dose-response of CHIR99021 treatment (HeLa/STARD3)
STARD3
pS209
Total
GAPDH
kDa
55
55
35
0 1 2 5
CHIR99021 concentration (µM)
C Time course of CHIR99021 treatment (HCC1954)
a
STARD3
pS209
Total
GAPDH
kDa
55
55
35
0 2 4 6 8 16
Duration of CHIR99021 treatment (hour)
b
Relative STARD3 S209 phosphorylation level
Duration of CHIR99021 treatment (hour)
ns
ns
*
*
***
c
LAMP1
STARD3
0 2 4 6 8 16
Duration of CHIR99021 treatment (hour)
D HCC1954
GSK3α
Relative GSK3α levels
GSK3β
Relative GSK3β levels
WT siCtrl α β α+β
siGSK3
E MCF7/STARD3
GSK3α
Relative GSK3α levels
GSK3β
Relative GSK3β levels
WT siCtrl α β α+β
siGSK3

© The Author(s)

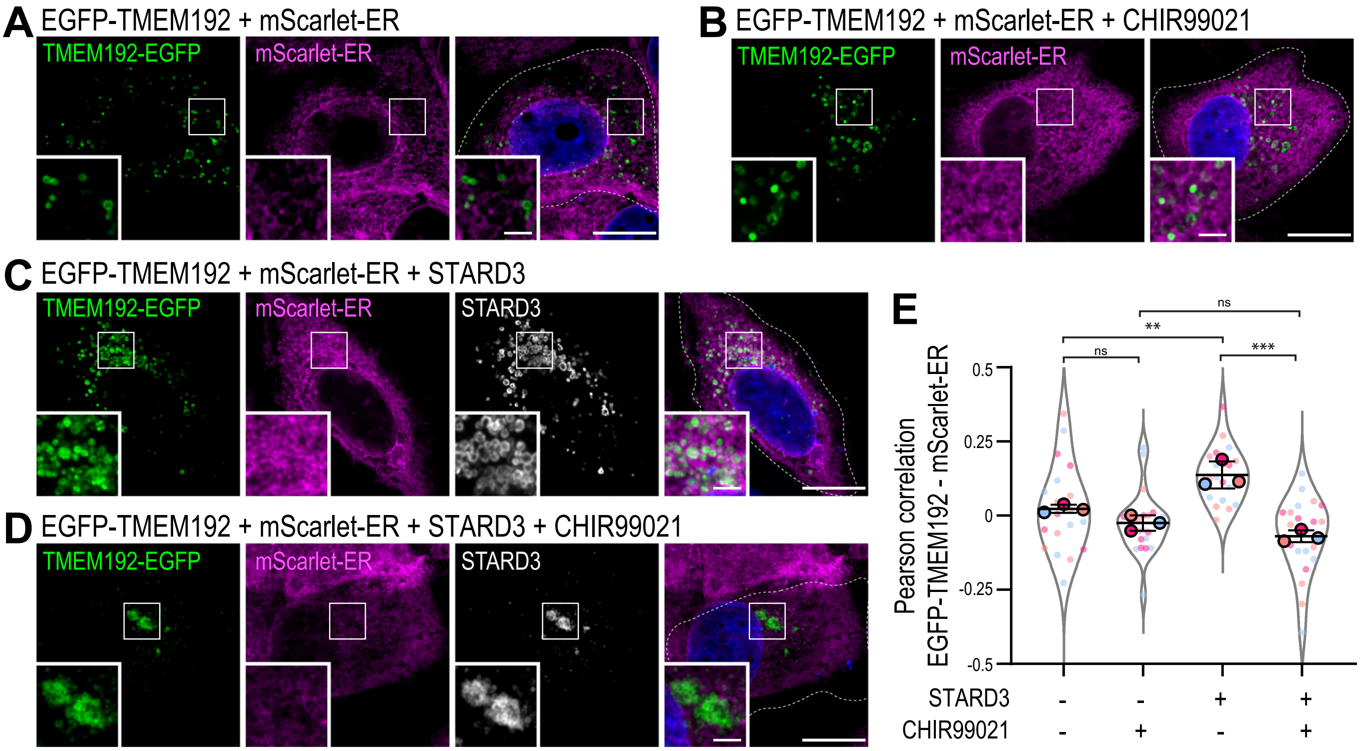

Figure EV2. In vivo, GSK3 activity governs the establishment of STARD3-mediated ER-endosome contacts.

(**A–D**) MCF7 cells expressing mScarlet-ER (magenta) were transfected with EGFP-TMEM192 (green) (**A–D**) and STARD3 WT (**C**, **D**). Cells were left untreated (**A**, **C**) or treated with CHIR99021 (**B**, **D**). STARD3 was labeled using anti-STARD3 antibodies (gray), and nuclei were stained with Hoechst (blue). Insets show higher magnification images of the areas outlined in white. Scale bars: 10 µm. Inset scale bars: 2 µm. Overlay panels show merged green, magenta and blue channels. In (**A**, **B**), endogenous STARD3 levels were too low to be detected with anti-STARD3 antibodies. (**E**) Pearson's correlation coefficients between EGFP-TMEM192 and mScarlet-ER in cells with or without STARD3 expression and with or without CHIR99021 treatment. Data are displayed as Superplots showing the clustering index per cell (small dots) and its mean per independent experiment (large dots). Number of cells: mScarlet-ER / EGFP-TMEM192: 21; mScarlet-ER / EGFP-TMEM192 treated with CHIR99021: 22; mScarlet-ER / EGFP-TMEM192 / STARD3: 22, mScarlet-ER / EGFP-TMEM192 / STARD3 treated with CHIR99021: 22, from three independent experiments). Means and error bars (SD) are shown. ANOVA with Tukey's multiple comparison test (**, $P < 0.01$; ***, $P < 0.001$; mScarlet-ER / EGFP-TMEM192 vs mScarlet-ER / EGFP-TMEM192-CHIR99021: $P = 0.26$; mScarlet-ER / EGFP-TMEM192 vs mScarlet-ER / EGFP-TMEM192 / STARD3: $P = 6 \times 10^{-3}$; mScarlet-ER / EGFP-TMEM192-CHIR99021 vs mScarlet-ER / EGFP-TMEM192 / STARD3-CHIR99021: $P = 0.31$; mScarlet-ER / EGFP-TMEM192 / STARD3 vs mScarlet-ER / EGFP-TMEM192 / STARD3-CHIR99021: $P = 1.9 \times 10^{-4}$). Source data are available online for this figure.

 © The Author(s)

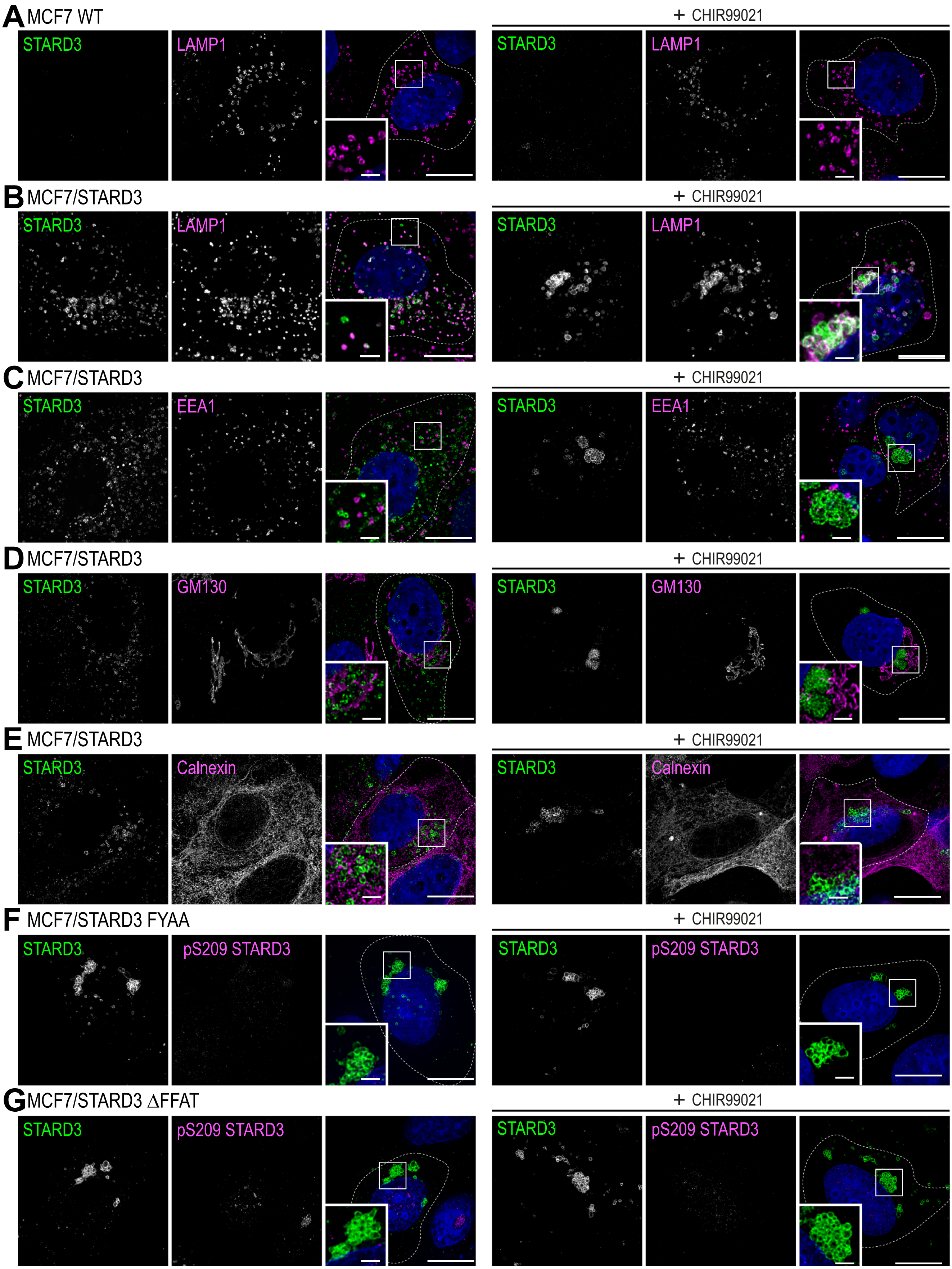
A MCF7 WT
+ CHIR99021
STARD3
LAMP1
B MCF7/STARD3
+ CHIR99021
STARD3
LAMP1
C MCF7/STARD3
+ CHIR99021
STARD3
EEA1
D MCF7/STARD3
+ CHIR99021
STARD3
GM130
E MCF7/STARD3
+ CHIR99021
STARD3
Calnexin
F MCF7/STARD3 FYAA
+ CHIR99021
STARD3
pS209 STARD3
G MCF7/STARD3 ΔFFAT
+ CHIR99021
STARD3
pS209 STARD3

Figure EV3. STARD3 mediates the formation of LE/Lys clusters but does not affect other organelles.

WT MCF7 cells (**A**), MCF7 cells expressing STARD3 WT (**B–F**) or FFAT-motif deficient mutants (STARD3 FYAA (**F**), STARD3 ΔFFAT (**G**)) were left untreated (left) or treated with CHIR99021 (5 µM, overnight; right). Cells were labeled with anti-STARD3 antibodies (green) (**A–G**) and in magenta with: anti-LAMP1 antibodies (**A**, **B**) to label LE/Lys, anti-EEA1 antibodies (**C**) to label early endosomes, anti-GM130 antibodies (**D**) to label the Golgi apparatus, anti-calnexin antibodies (**E**) to label the ER or phospho-specific antibodies (pS_{209} STARD3, **F**, **G**). Nuclei were stained with Hoechst (blue). Subpanels show higher magnification images of the area outlined in white. Scale bars: 10 µm. Inset scale bars: 2 µm. Source data are available online for this figure.

 © The Author(s)

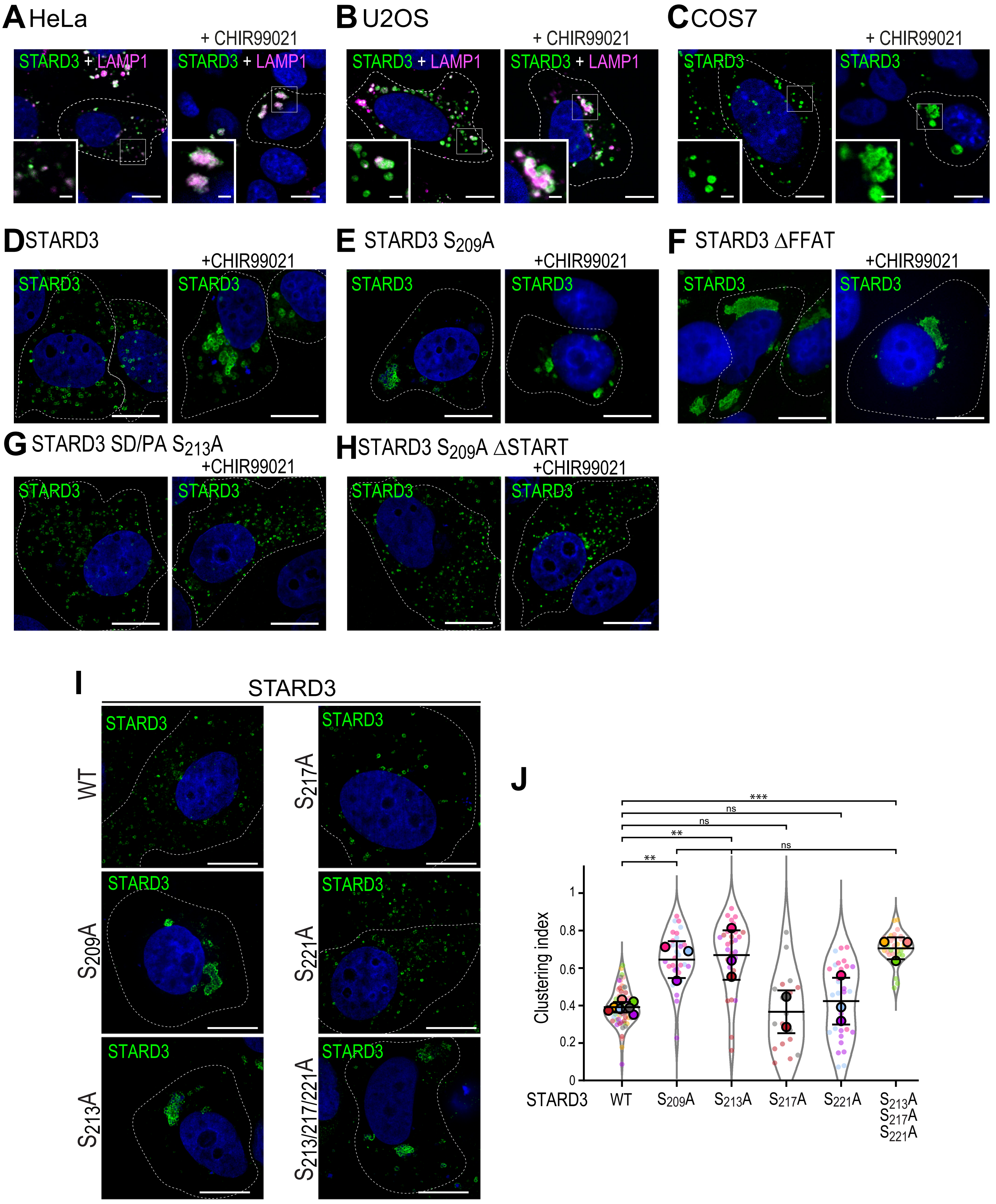
A HeLa
+ CHIR99021
STARD3 + LAMP1
B U2OS
C COS7
STARD3
D STARD3
+CHIR99021
E STARD3 S209A
F STARD3 ΔFFAT
G STARD3 SD/PA S213A
H STARD3 S209A ΔSTART
I
WT
S209A
S213A
S217A
S221A
S213/217/221A
J
Clustering index
***
ns
**
0
0.2
0.4
0.6
0.8
1
WT
S209A
S213A
S217A
S221A
S213A S217A S221A

Figure EV4. Characterization of STARD3-induced LE/Lys clusters.

(**A–C**) HeLa cells (**A**), U2OS cells (**B**) and COS-7 cells (**C**) expressing STARD3 WT were left untreated (left) or treated with CHIR99021 (5 µM, overnight) (right). Cells were labeled with anti-STARD3 antibodies (green) and anti-LAMP1 antibody (magenta). Nuclei were stained with Hoechst (blue). Subpanels show higher magnification images of the area outlined in white. Images were acquired with a SP8-UV confocal microscope. Scale bars: 10 µm. Inset scale bars: 2 µm. (**D–H**) MCF7 (**A**) expressing STARD3 WT (**D**), STARD3 S_{209}A (**E**), STARD3 ΔFFAT (**F**), STARD3 SD/PA S_{213}A (**G**) or STARD3 S_{209}A ΔSTART (**H**) were left untreated (left) or treated with CHIR99021 (5 µM, overnight; right). Cells were labeled with anti-STARD3 antibodies (green). Nuclei were stained with Hoechst (blue). Scale bars: 10 µm. Inset scale bars: 2 µm. (**I**, **J**) Representative images of MCF7 cells expressing STARD3 WT, STARD3 S_{209}A, STARD3 S_{213}A, STARD3 S_{217}A, STARD3 S_{221}A, or STARD3 S_{213}A-S_{217}A-S_{221}A. Cells were labeled with anti-STARD3 antibodies (green) and Hoechst (blue). Scale bars: 10 µM. (**E**) Quantification of LE/Lys clustering in cells shown in (**D**). Data are displayed as Superplots showing the clustering index per cell (small dots) and its mean per independent experiment (large dots). Number of cells: MCF7-STARD3: 70, MCF7-STARD3 S_{209}A: 31, MCF7-STARD3 S_{213}A: 31, MCF7-STARD3 S_{217}A: 18, MCF7-STARD3 S_{221}A: 32, MCF7-STARD3 S_{213}A-S_{217}A-S_{221}A: 31, from seven independent experiments). Independent experiments are color-coded. Means and error bars (SD) are shown as black bars. One-way ANOVA with Tukey's multiple comparison test (**, $P < 0.01$; ***, $P < 0.001$; $n = 3$–7 independent experiments; WT vs S_{209}A, $P = 6.8 \times 10^{-3}$; WT vs S_{213}A, $P = 3 \times 10^{-3}$; WT vs S_{217}A, $P = 0.99$; WT vs S_{221}A, $P = 0.99$; WT vs S_{213}A-S_{217}A-S_{221}A, $P = 10^{-3}$; S_{209}A vs S_{213}A, $P = 0.99$; S_{209}A vs S_{213}A-S_{217}A-S_{221}A, $P = 0.95$; S_{213}A vs S_{213}A-S_{217}A-S_{221}A, $P = 0.99$). Source data are available online for this figure.

 © The Author(s)

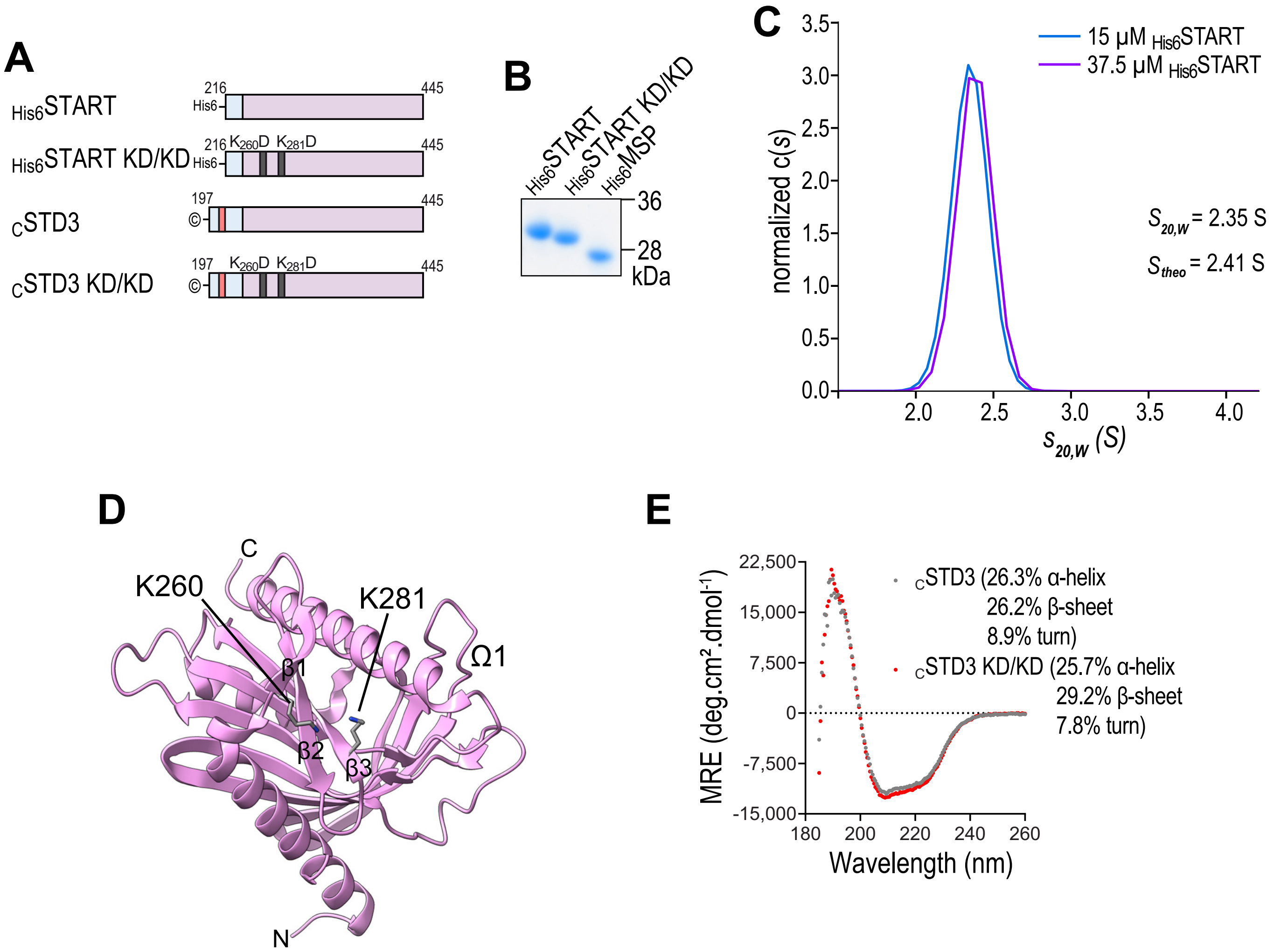

Figure EV5. The START domain is a monomer in solution and contains positively charged surface patches.

(**A**) Schematic representation of the different recombinant proteins used. (**B**) Coomassie blue staining of the recombinant START domain of STARD3 (WT and KD/KD mutant) and the MSP domain of VAP-B proteins after SDS-PAGE. (**C**) Superimposition of sedimentation coefficient distributions c(s) obtained from sedimentation velocity (SV) experiments with either 15 μM (blue) or 37.5 μM (purple) of the recombinant START domain of STARD3. The experimental average sedimentation coefficient ($S_{20,w}$) of 2.35 S is close to the theoretical value (S_{theo}) of 2.41 S, indicating that the protein remains in a stable monomeric form at both concentrations. (**D**) Ribbon diagram of the START domain of STARD3. The positions of the two mutated residues, K260 and K281, are highlighted. Key structural features, including omega loop 1 (Ω1) and three beta strands (β1, β2, β3), are also indicated (PDB ID: 1EM2) (Tsujishita and Hurley, 2000). (**E**) Far-UV CD spectrum of purified $_C$STD3 and $_C$STD3 KD/KD constructs in 20 mM Tris pH 7.4, 120 mM NaF buffer at room temperature. Source data are available online for this figure.

© The Author(s)
